# Annotated catalogue of the Tachinidae (Insecta, Diptera) of the Afrotropical Region, with the description of seven new genera

**DOI:** 10.3897/zookeys.575.6072

**Published:** 2016-03-31

**Authors:** James E. O’Hara, Pierfilippo Cerretti

**Affiliations:** 1 Canadian National Collection of Insects, Agriculture and Agri-Food Canada, 960 Carling Avenue, Ottawa, Ontario, Canada, K1A 0C6; 2 DAFNAE-Entomologia, Università degli Studi di Padova, Viale dell’Università 16, I 35020 Legnaro (Padova), Italy; 3 Dipartimento di Biologia e Biotecnologie “Charles Darwin”, Sapienza Università di Roma, Piazzale A. Moro 5, 00185, Rome, Italy

**Keywords:** Afrotropical Region, parasitoids, classification, distribution, zoological nomenclature, systematics, new taxa

## Abstract

The Tachinidae of the Afrotropical Region are catalogued and seven genera and eight species are newly described. There are 237 genera and 1126 species recognized, of which 101 genera and 1043 species are endemic to the region. The catalogue is based on examination of the primary literature comprising about 525 references as well as numerous name-bearing types and other specimens housed in collections. Taxa are arranged hierarchically and alphabetically under the categories of subfamily, tribe, genus, subgenus (where recognized), species, and rarely subspecies. Nomenclatural information is provided for all genus-group and species-group names, including lists of synonyms (mostly restricted to Afrotropical taxa) and name-bearing type data. Species distributions are recorded by country within the Afrotropical Region and by larger geographical divisions outside the region. Additional information is given in the form of notes, numbering about 300 in the catalogue section. Seven genera and eight species are described as new: *Afrophylax* Cerretti & O’Hara with type species *Sturmia aureiventris* Villeneuve, 1910, **gen. n.** (Exoristinae, Eryciini); *Austrosolieria* Cerretti & O’Hara with type species *Austrosolieria londti* Cerretti & O’Hara, **gen. n.** and **sp. n.** (South Africa) and *Austrosolieria freidbergi* Cerretti & O’Hara, **sp. n.** (Malawi) (Tachininae, Leskiini); *Carceliathrix* Cerretti & O’Hara with type species *Phorocera crassipalpis* Villeneuve, 1938, **gen. n.** (Exoristinae, Eryciini); *Filistea* Cerretti & O’Hara with type species *Viviania aureofasciata* Curran, 1927, **gen. n.** and *Filistea verbekei* Cerretti & O’Hara, **sp. n.** (Cameroon, D.R. Congo, Uganda) (Exoristinae, Blondeliini); *Mesnilotrix* Cerretti & O’Hara with type species *Dexiotrix empiformis* Mesnil, 1976, **gen. n.** (Dexiinae, Dexiini); *Myxophryxe* Cerretti & O’Hara with type species *Phorocera longirostris* Villeneuve, 1938, **gen. n.**, *Myxophryxe murina* Cerretti & O’Hara, **sp. n.** (South Africa), *Myxophryxe regalis* Cerretti & O’Hara, **sp. n.** (South Africa), and *Myxophryxe satanas* Cerretti & O’Hara, **sp. n.** (South Africa) (Exoristinae, Goniini); and *Stiremania* Cerretti & O’Hara with type species *Stiremania karoo* Cerretti & O’Hara, **gen. n.** and **sp. n.** (South Africa), and *Stiremania robusta* Cerretti & O’Hara, **sp. n.** (South Africa) (Exoristinae, Goniini). *Paraclara* Bezzi, 1908 is transferred from the Cylindromyiini to the Hermyini, **comb. n.** *Sarrorhina* Villeneuve, 1936 is transferred from the Minthoini to the Graphogastrini, **comb. n.** Three genera are newly recorded from the Afrotropical Region: *Madremyia* Townsend, 1916 (Eryciini); *Paratrixa* Brauer & Bergenstamm, 1891 (Blondeliini); and *Simoma* Aldrich, 1926 (Goniini). Three genera previously recorded from the Afrotropical Region are no longer recognized from the region: *Calozenillia* Townsend, 1927 (Palaearctic, Oriental and Australasian regions); *Eurysthaea* Robineau-Desvoidy, 1863 (Palaearctic, Oriental and Australasian regions); and *Trixa* Meigen, 1824 (Palaearctic and Oriental regions). Two species are newly recorded from the Afrotropical Region: *Amnonia carmelitana* Kugler, 1971 (Ethiopia, Kenya); and *Simoma grahami* Aldrich, 1926 (Namibia). Three species previously recorded from the Afrotropical Region are no longer recognized from the region: *Euthera peringueyi* Bezzi, 1925 (Oriental Region); *Hamaxia incongrua* Walker, 1860 (Palaearctic, Oriental and Australasian regions); *Leucostoma tetraptera* (Meigen, 1824) (Palaearctic Region). New replacement names are proposed for five preoccupied names of Afrotropical species: *Billaea rubida* O’Hara & Cerretti for *Phorostoma rutilans* Villeneuve, 1916, preoccupied in the genus *Billaea* Robineau-Desvoidy, 1830 by *Musca rutilans* Fabricius, 1781, **nom. n.**; *Cylindromyia braueri* O’Hara & Cerretti for *Ocyptera nigra* Villeneuve, 1918, preoccupied in the genus *Cylindromyia* Meigen, 1803 by *Glossidionophora nigra* Bigot, 1885, **nom. n.**; *Cylindromyia rufohumera* O’Hara & Cerretti for *Ocyptera scapularis* Villeneuve, 1944, preoccupied in the genus *Cylindromyia* Meigen, 1803 by *Ocyptera scapularis* Loew, 1845, **nom. n.**; *Phytomyptera longiarista* O’Hara & Cerretti for *Phytomyzoneura aristalis* Villeneuve, 1936, preoccupied in the genus *Phytomyptera* Rondani, 1845 by *Phasiostoma aristalis* Townsend, 1915, **nom. n.**; and Siphona (Siphona) pretoriana O’Hara & Cerretti for *Siphona laticornis* Curran, 1941, preoccupied in the genus *Siphona* Meigen, 1803 by *Actia laticornis* Malloch, 1930, **nom. n.** New type species fixations are made under the provisions of Article 70.3.2 of the ICZN *Code* for two genus-group names: *Lydellina* Villeneuve, 1916, type species newly fixed as *Lydellina villeneuvei* Townsend, 1933 (valid genus name); and *Sericophoromyia* Austen, 1909, type species newly fixed as *Tachina quadrata* Wiedemann, 1830 (synonym of *Winthemia* Robineau-Desvoidy, 1830). Lectotypes are designated for the following nine nominal species based on examination of one or more syntypes of each: *Degeeria crocea* Villeneuve, 1950; *Degeeria semirufa* Villeneuve, 1950; *Erycia brunnescens* Villeneuve, 1934; *Exorista oculata* Villeneuve, 1910; *Kiniatilla tricincta* Villeneuve, 1938; *Myxarchiclops caffer* Villeneuve, 1916; *Ocyptera linearis* Villeneuve, 1936; *Peristasisea luteola* Villeneuve, 1934; and *Phorocera crassipalpis* Villeneuve, 1938. The following four genus-group names that were previously treated as junior synonyms or subgenera are recognized as valid generic names: *Bogosiella* Villeneuve, 1923, **status revived**; *Dyshypostena* Villeneuve, 1939, **status revived**; *Perlucidina* Mesnil, 1952, **status revived**; and *Thelymyiops* Mesnil, 1950, **status n.** The following six species-group names that were previously treated as junior synonyms are recognized as valid species names: *Besseria fossulata* Bezzi, 1908, **status revived**; *Degeeria cinctella* Villeneuve, 1950, **status revived** (as *Medina cinctella* (Villeneuve)); *Nemoraea miranda intacta* Villeneuve, 1916, **status revived** (as *Nemoraea intacta* Villeneuve); *Succingulum exiguum* Villeneuve, 1935, **status revived** (as *Trigonospila exigua* (Villeneuve)); *Wagneria rufitibia abbreviata* Mesnil, 1950, **status n.** (as *Periscepsia abbreviata* (Mesnil)); and *Wagneria rufitibia nudinerva* Mesnil, 1950, **status n.** (as *Periscepsia nudinerva* (Mesnil)). The following 25 new or revived combinations are proposed: *Afrophylax aureiventris* (Villeneuve, 1910), **comb. n.**; *Blepharella orbitalis* (Curran, 1927), **comb. n.**; *Bogosiella pomeroyi* Villeneuve, 1923, **comb. revived**; *Brachychaetoides violacea* (Curran, 1927), **comb. n.**; *Carceliathrix crassipalpis* (Villeneuve, 1938), **comb. n.**; *Charitella whitmorei* (Cerretti, 2012), **comb. n.**; *Dyshypostena edwardsi* (van Emden, 1960), **comb. n.**; *Dyshypostena tarsalis* Villeneuve, 1939, **comb. revived**; *Estheria buccata* (van Emden, 1947), **comb. n.**; *Estheria surda* (Curran, 1933), **comb. n.**; *Filistea aureofasciata* (Curran, 1927), **comb. n.**; *Madremyia setinervis* (Mesnil, 1968), **comb. n.**; *Mesnilotrix empiformis* (Mesnil, 1976), **comb. n.**; *Myxophryxe longirostris* (Villeneuve, 1938), **comb. n.**; *Nealsomyia chloronitens* (Mesnil, 1977), **comb. n.**; *Nealsomyia clausa* (Curran, 1940), **comb. n.**; *Nilea longicauda* (Mesnil, 1970), **comb. n.**; *Paratrixa aethiopica* Mesnil, 1952, **comb. revived**; *Paratrixa stammeri* Mesnil, 1952, **comb. revived**; *Perlucidina africana* (Jaennicke, 1867), **comb. n.**; *Perlucidina perlucida* (Karsch, 1886), **comb. revived**; *Prolophosia retroflexa* (Villeneuve, 1944), **comb. n.**; *Sturmia profana* (Karsch, 1888), **comb. n.**; additionally, *Ceromasia rufiventris* Curran, 1927 is treated as an unplaced species of Goniini, **comb. n.** and *Hemiwinthemia stuckenbergi* Verbeke, 1973 is treated as an unplaced species of Leskiini, **comb. n.** New or revived generic and specific synonymies are proposed for the following nine names: *Afrosturmia* Curran, 1927 with *Blepharella* Macquart, 1851, **syn. n.**; *Archiphania* van Emden, 1945 with *Catharosia* Rondani, 1868, **syn. revived**; *Besseria longicornis* Zeegers, 2007 with *Besseria fossulata* Bezzi, 1908 (current name *Besseria fossulata*), **syn. n.**; *Dexiomera* Curran, 1933 with *Estheria* Robineau-Desvoidy, 1830, **syn. n.**; *Hemiwinthemia francoisi* Verbeke, 1973 with *Nemoraea capensis* Schiner, 1868 (current name *Smidtia capensis*), **syn. n.**; *Kinangopana* van Emden, 1960 with *Dyshypostena* Villeneuve, 1939, **syn. n.**; *Metadrinomyia* Shima, 1980 with *Charitella* Mesnil, 1957, **syn. n.**; *Phorocera majestica* Curran, 1940 with *Phorocera longirostris* Villeneuve, 1938 (current name *Myxophryxe longirostris*), **syn. n.**; and *Podomyia discalis* Curran, 1939 with *Antistasea fimbriata* Bischof, 1904 (current name *Antistasea fimbriata*), **syn. n.**

## Introduction

The Tachinidae are a large cosmopolitan family of flies that are parasitoids of other arthropods, primarily other insects (Stireman et al. 2006). The Afrotropical fauna of the Tachinidae was last catalogued 35 years ago by Crosskey (1980b), who had previously prepared conspecti of the tachinids of Australia and the Oriental Region (Crosskey 1973b, 1976). His catalogue and the keys that followed four years later to the tachinid genera of southern and tropical Africa (Crosskey 1984) continue to this day as the main sources of information on the classification and identification of Afrotropical Tachinidae. Crosskey prefaced his catalogue with a review of the “scanty” knowledge of the biology and hosts of the tachinids of the region, and briefly summarized the unsettled state of the classification. He noted the difficulty of delimiting taxa at all levels and blamed the problem at the species level on the “wealth of intangibly varying characters” (Crosskey 1980b: 822). This has been a familiar lament among taxonomists throughout the world who have attempted to classify regional faunas of the family.

Crosskey (1984) reviewed in some detail the history of tachinid taxonomy in the Afrotropical Region. He unflatteringly portrayed the most prolific of the early taxonomists, Villeneuve and Curran, as failing to bring order to the fauna at the supraspecific level and of leaving a legacy of species largely unidentifiable without study of the types. Van Emden, following in the wake of such workers in the middle part of the 1900s, began the formidable task of revising the Afrotropical fauna subfamily by subfamily (van Emden 1945, 1947, 1960) but died before the project could be completed and with the largest and most difficult subfamily, the Exoristinae, untouched. Mesnil was active too during this time and described a significant number of Afrotropical genera and species even though his primary goal was to revise the entire tachinid fauna of the Palaearctic Region. Verbeke, in a series of papers in the 1960s and 70s, was the last taxonomist of note to advance tachinid classification within the Afrotropics prior to Crosskey’s synthesis of the fauna in his catalogue and keys.

Crosskey’s exemplary skills as a taxonomist, nomenclaturalist and bibliographer ensured that his Afrotropical catalogue and keys were virtually free of errors in their presentation of factual information. His higher classification of the Tachinidae, however, was little changed from his earlier conspecti and in this respect was not progressive. Nevertheless, it suited Crosskey’s desire to construct keys that would first separate tribes and then genera within tribes. His classification was already at odds with the advances being made in tachinid relationships by Mesnil, Herting and Verbeke (O’Hara 2013), but it was the publication of Herting’s (1984) catalogue of Palaearctic Tachinidae that was most influential in galvanizing support for a more phylogenetic classification of the family.

Crosskey’s (1980b) catalogue differed from his conspecti of the Australian and Oriental faunas (Crosskey 1973b, 1976) in lacking information about name-bearing types. This information has been included in the present catalogue based on the examination of all original descriptions and relevant subsequent literature. The major works that have been published on Afrotropical Tachinidae since Crosskey’s catalogue are reviewed below and our revised classification is discussed in light of recent studies on tachinid evolution and conflicting phylogenetic interpretations.

The main impetus for preparing this catalogue was the announcement in 2010 during the *7^th^ International Congress of Dipterology* in San José, Costa Rica, of an international effort to publish a *Manual of Afrotropical Diptera* (A.H. Kirk-Spriggs and B.J. Sinclair, editors, in prep.). The Tachinidae are by far the largest family of Afrotropical Diptera in terms of genera and the *Manual* chapter detailing this diversity is recognized as a considerable challenge by the authors (P. Cerretti, J.E. O’Hara, J.O. Stireman and D.M. Wood, in prep.). This catalogue is intended as both a companion volume to the *Manual* chapter and a resource for the chapter authors as they prepare a key to genera and evaluate the diversity, biology and biogeography of the tachinid fauna.

The geographic limits of the Afrotropical Region for the purposes of this catalogue have been changed slightly from those of Crosskey (1980a) to conform to the limits recognized by the *Manual of Afrotropical Diptera*. As such, Oman and United Arab Emirates, both formerly included within the Palaearctic Region, are treated here as part of the Afrotropical Region.

Numerous specimens of Afrotropical Tachinidae were examined during the preparation of this catalogue. This has led to taxonomic changes within the catalogue and also revealed numerous new species and a smaller number of new genera. Described herein are seven new genera that are well characterized and worthy of formal recognition in this catalogue and by such treatment will be available for inclusion in the key to tachinid genera in the *Manual*.

The *Catalogue of the Diptera of the Afrotropical Region* recognized 95 families, 2020 genera and 16,550 species (Crosskey 1980a; including additional genera and species listed in the appendix). The Tachinidae were the dominant family in terms of genera with 210, or 10.4% of all genera of Afrotropical Diptera. The number of tachinid species was proportionally smaller but still high at 996, or 6.0% of all dipteran species.

The number of Afrotropical tachinid genera and species has risen modestly over the past 35 years due to taxonomic activity, an expansion of the region’s boundaries, and the new taxa described herein. The present catalogue records 237 genera, of which 101 (43%) are endemic to the region. Of the 1126 species recorded, a total of 1043 (93%) are endemic. The current numbers represent an increase since 1980 of 29 genera and 130 species. Despite these advances, the tachinid fauna of the region remains understudied and many new taxa await discovery and description.

## Materials and methods

### Format

This catalogue is arranged in a similar manner to the one on the Tachinidae of China by O’Hara et al. (2009). The sections here under Format are little changed from the same sections in that work but are given here as a convenient guide and have been modified to apply to the Afrotropical Tachinidae. Any changes in format or interpretation of nomenclatural matters compared to O’Hara et al. (2009) are noted.

### General

This catalogue cites all nominal species in their original combinations, provides details about name-bearing types, gives known distributions, and is based on the examination of all but a very few of the approximately 525 publications listed in the References.

Valid names are arranged hierarchically and alphabetically according to the categories of subfamily, tribe, genus, subgenus, species, and subspecies. Synonyms are given for valid names of genera, subgenera, and species and are listed chronologically. Synonymic lists comprise taxa described from the Afrotropical Region, synonyms that have been used as valid names in the literature on Afrotropical Tachinidae, and (where known) misidentifications (given last in synonymic lists).

Each genus-group name is listed with the following information: genus name in italics and capital letters (and additionally in bold if valid, unless misidentified from the Afrotropics), author, year (with letter if applicable), page, note in parentheses if applicable (e.g., junior homonym, proposed as subgenus), type species with author and date, form of type fixation, and region of origin of type species in square brackets if not the Afrotropics. Each type species is cited in its original binomen (Recommendation 67B of the *Code*, ICZN 1999), and if that name is a synonym then it is followed by the valid name of the species in parentheses. We have invoked Article 70.3.2 of the *Code* (ICZN 1999) to fix the intended species as the type species for generic names that were based on misidentified type species. This maintains the concepts of these generic names as currently accepted and in prevailing usage. The genera so affected are listed below under “Summary of new taxonomic and nomenclatural changes”.

Type species were fixed by original designation, monotypy, subsequent designation, or in a few instances subsequent monotypy, except for type species newly fixed here for nominal genera based on misidentified type species. Fixation by original designation requires an explicit designation of a type species (Article 68.2 of the *Code*, ICZN 1999), so a new genus “proposed for” or “erected for” a single species has its type species fixed by monotypy. A new genus proposed before 1931 for a single species and accompanied by the expression “gen. n., sp. n.” or an equivalent also has its type species fixed by monotypy (Article 68.2.1). If, on the other hand, the new genus is proposed for more than one new species and the expression “gen. n., sp. n.” or an equivalent is applied to only one of the new species, then that species is fixed as type species by original designation (Article 68.2.1).

Species are listed by valid name followed by the available name(s) associated with it; i.e., the available name of the valid name plus synonyms. The valid name is represented by the valid specific epithet in bold and italics (in italics only if questionably recorded or misidentified from the Afrotropics) followed by the author, date (no letter), and known distribution. Author and date are enclosed in parentheses if the species has moved from its original genus. The distribution is given first for the Afrotropical Region and then for other regions as explained under “Geographic divisions” and “Distributional data”. Each available name is given in italics in its original combination and spelling followed by author, year (with letter if applicable to match a publication listed in the References), page, and a note in parentheses if applicable (e.g., junior homonym, subsequent spelling). A questionable synonym is preceded by a question mark (e.g., “? *Ocyptera cribrata* Villeneuve”). Given next is name-bearing type information consisting of status (holotype, lectotype, neotype, or syntypes), sex (of single type, or number and sex of syntypes), type depository (in parentheses), and type locality. If a neotype or lectotype was designated then a citation is given to the designation. Additional information may be given in parentheses with the type depository to cite the number and sex of syntypes existing in a collection if that number is different from the information given in the original description, or if the original description did not provide details about the type series; also, a reference may be cited wherein information can be found about the name-bearing type.

A subsequent spelling of a generic or specific name can be an incorrect subsequent spelling (which is not an available name) or an unjustified emendation (which is an available name with its own author and date). Incorrect subsequent spellings encountered during this study are cited but there are certainly others that escaped our notice. In a departure from the catalogue of O’Hara et al. (2009), an unjustified emendation is cited with an author and date (name only given in the prior catalogue except in rare cases).

The following acronyms are used in this work:

Code
*International Code of Zoological Nomenclature*, specifically the fourth edition published by the International Commission on Zoological Nomenclature in 1999; cited as ICZN 1999 


ICZN
 International Commission on Zoological Nomenclature 


JEOH
 James E. O’Hara 


PC
 Pierfilippo Cerretti 


### Name-bearing types

We follow the same method developed by O’Hara et al. (2009) for citing name-bearing type information for species described without a holotype designation in the original publication or without a subsequent lectotype or neotype designation. Details are provided about name-bearing types based on the content of an original description and are not biased by existing type material in collections (that information being given in parentheses with the type depository). Our format for citing published data on name-bearing types other than a designated holotype, lectotype or neotype is explained below.

Type(s), male: One or more males. This citation is used for a species described from the male sex without indication of whether a single male (i.e., a holotype) or more than one male (i.e., syntypes) comprised the type series.

Type(s), female: One or more females. See “Type(s), male”.

Type(s), unspecified sex: One or more specimens with no indication of sex.

Syntypes, [number] male[s] and [number] female[s] (e.g., “Syntypes, 3 males and 2 females”): Species described from an indicated number of males and females.

Syntypes, males and females: Species described from both sexes but the number of each sex was not given.

Syntypes, males: Species described from more than one male but without indication of the number of males.

Syntypes, females: Species described from more than one female but without indication of the number of females.

Syntypes, unspecified number and sex: Species described from more than one specimen but without indication of sex or number of specimens.

### Avoidance of assumption of holotype

In following the foregoing format we have complied with Recommendation 73F of the *Code* (ICZN 1999), “Avoidance of assumption of holotype”, which states: “Where no holotype or syntype was fixed for a nominal species-group taxon established before 2000, and when it is possible that the nominal species-group taxon was based on more than one specimen, an author should proceed as though syntypes may exist and, where appropriate, should designate a lectotype rather than assume a holotype (see also Article 74.6)”. See O’Hara et al. (2009: 9–10) for a further discussion of this issue.

By following Recommendation 73F of the *Code*, assumed holotypes take on the status of syntypes. The recommendation favors “where appropriate” the designation of lectotypes. We have combined the spirit of Recommendation 73F and the provisions of Article 74.5 of the *Code* (ICZN 1999) to recognize certain published statements (as discussed in next section) about assumed holotypes as lectotype fixations. This follows O’Hara et al. (2009) and is in our opinion the best way to reconcile assumed holotypes with the modern rules of nomenclature, while also giving credit of lectotype fixations to the authors who assumed holotypes (e.g., van Emden 1960, Crosskey 1976).

### Lectotypifications

There are two types of lectotypification in zoological nomenclature, explicit and implicit. In the former, a single syntype in a type series is designated as lectotype; in the latter, there is some form of statement that can be construed as the selection of a single name-bearing type. We follow O’Hara et al. (2009) in using the term “lectotype designation” for an explicit lectotypification and “lectotype fixation” for an implicit lectotypification. There is good reason to distinguish between the two because implicit lectotypifications are open to some interpretation, especially with respect to Article 74.5 of the *Code* (ICZN 1999: 82–83) that deals in part (see also Article 74.6) with lectotype designations before 2000:

“In a lectotype designation made before 2000, either the term ‘lectotype’, or an exact translation or equivalent expression (e.g. ‘the type’), must have been used or the author must have unambiguously selected a particular syntype to act as the unique name-bearing type of the taxon. When the original work reveals that the taxon had been based on more than one specimen, a subsequent use of the term ‘holotype’ does not constitute a valid lectotype designation unless the author, when wrongly using that term, explicitly indicated that he or she was selecting from the type series that particular specimen to serve as the name-bearing type”.

What constitutes a valid lectotypification (or lectotype fixation in our terminology) in the foregoing is largely dependent on how one interprets the passage about an author explicitly indicating “that he or she was selecting from the type series that particular specimen to serve as the name-bearing type”. At one end of the spectrum is the mere mention of a “holotype” or “type” by a subsequent author when the original type series clearly consisted of two or more syntypes. This statement does not constitute a lectotype fixation because the “holotype” is not distinguishable from other syntypes. At the other end of the spectrum is the mention of a “holotype” or “type” with accompanying details about its labelling, features, damage, etc. that clearly distinguishes that specimen from other syntypes; or perhaps there is only one type specimen in a collection and it is an “assumed holotype” (see section above) for a species described from an unspecified number of specimens. We considered these latter statements about a single type to qualify as lectotype fixations under Article 74.5 because they contain an explicit indication that an author accepted the cited “holotype” as the name-bearing type and restricted the term to a single recognizable specimen in a collection. We encountered many “holotype” statements that were not so easily interpretable as the aforementioned ones. For these, we adopted the criteria that there had to be reasonable grounds to believe the information provided would permit the “holotype” or “type” to be recognized in a collection, and we generally required some additional data beyond the mere mention of a “holotype” or “type”, for a statement to qualify as a lectotype fixation.

O’Hara et al. (2009) chose not to recognize lectotype fixations in Townsend’s *Manual of Myiology* [Parts I–XII, 1934–1942]. They argued that Townsend consistently used the term “Ht” (holotype) for the name-bearing type of a type species of a nominal genus whether or not a holotype had been designated in the original publication or the “Ht” had been personally examined. This approach was adopted to avoid certain pitfalls that would follow from a universal acceptance of these cited holotypes (see O’Hara et al. 2009: 11). We have reconsidered this matter and have elected to follow Crosskey (1969: 88, 1971: 255) and O’Hara and Wood (2004: 4) in accepting the mention of a “Ht” (when accompanied by information about type locality and type depository) in *Manual of Myiology* as a lectotype fixation if the specimen can be recognized in the cited depository or has a strong possibility of being so recognized. We could not, for practical reasons, examine all putative lectotypes to verify that they can be recognized in their cited depositories. We consider the verification of such putative lectotypes to be a “work in progress” and a task for us and future researchers to be mindful of when dealing with nominal species for which Townsend or other authors may have fixed lectotypes.

### Type localities

Type localities are cited first by country and then by location within that country from larger to smaller geographic area or place. Spellings of geographic areas and places largely follow *The Times Comprehensive Atlas of the World* (Times Books 2007), if found in that work. Modern names and spellings are given where these have been determined. Country and province names (the latter generally given only for D.R. Congo, Madagascar and South Africa) are given only in their modern equivalents. For locality names that have changed since they were first published, the modern spelling is given first followed by the original spelling in square brackets and quotes; e.g., Kisangani [as “Stanleyville”]. Elevations are cited in metres (m) or feet (ft) as given by the author. Coordinates given in an original publication are cited in parentheses after the type locality and in their original format; e.g. Kenya, Western, Kakamega Forest, 1600m (0°13′37.2″N 34°52′49.8″E). Coordinates are included for many type localities that we had difficulty locating. These are given in square brackets (generally in degrees and minutes without seconds) after the locality to distinguish them from coordinates provided by an author; e.g., Rwanda, south of Volcan Karisimbi, Rivière Bikwi, 3100m [ca. 1°32′S, 29°30′E]. Localities that we could not find are given in quotes; e.g., Madagascar, “Ambalamalakana” [not located]. A variety of resources were used to locate type localities including atlases, maps, and literature, often found through Internet searches for the locality and/or collector. Two especially useful sources were: 1) the map in de Witte (1937) detailing the mountainous region between Lake Edward and Lake Kivu on the borders of D.R. Congo, Uganda and Rwanda, and 2) the maps in Scott (1958) of northern Ethiopia.

The type localities of almost 30 nominal species were published as the Rwenzori (often published as “Ruwenzori”) Range on the border of D.R. Congo and Uganda, frequently with additional data. Crosskey (1980b) placed some of these localities in D.R. Congo and others in Uganda. Other earlier authors cited only Uganda, and in the absence of evidence to the contrary we have cited all type localities associated with “Ruwenzori” as in Uganda.

Criteria for citing type localities from Sweden, and for nominal species described by Meigen, are explained in O’Hara et al. (2009: 11).

### Collections housing name-bearing types

The location of the name-bearing type (holotype, lectotype, neotype, or syntypes) is cited for each nominal species, where known. The collections housing these name-bearing types are listed below with the acronyms used in the text. We largely accepted as accurate the statements about the deposition of name-bearing types given in the original literature unless we had reason to doubt the information given (e.g., types known to have been relocated or are presumed lost). We personally examined many of the types cited in AMNH, BMNH, CNC, IRSNB, MCSN, MRAC, MSNM, MZF, MZUR, NHMW, NMB, NMDA, SAMC, SANC, SMNS, TAU, USNM, ZMHB and ZMUC.

The acronyms of collections cited in this work are as follows:

AMNH
American Museum of Natural History, New York, USA 


BMNH
Natural History Museum [formerly British Museum (Natural History)], London, United Kingdom 


CNC
 Canadian National Collection of Insects, Agriculture and Agri-Food Canada, Ottawa, Canada 


ETHZ
 Eidgenössische Technische Hochschule, Zürich, Switzerland 


FMNHH
Finnish Museum of Natural History, Zoological Museum, University of Helsinki, Helsinki, Finland 


HUJI
Hebrew University, Jerusalem, Israel 


IRSNB
Institut Royal des Sciences Naturelles de Belgique, Bruxelles [Brussels], Belgium 


JOS
 Private collection of J.O. Stireman, Dayton, Ohio, USA 


MCSN
Museo Civico di Storia Naturale, Genova [Genoa], Italy 


MHNG
Muséum d’Histoire Naturelle, Genève [Geneva], Switzerland 


MHNL
 Musée d’Histoire Naturelle de Lille, Lille, France 


MNCN
Museo Nacional de Ciencias Naturales, Madrid, Spain (including the collection of the former Instituto Español de Entomología) 


MNHN
Muséum National d’Histoire Naturelle, Paris, France 


MRAC
 Musée Royal de 1’Afrique Centrale, Tervuren, Belgium 


MSNM
Museo Civico di Storia Naturale, Milano [Milan], Italy 


MZF
 Museo Zoologico “La Specola”, Firenze [Florence], Italy 


MZLU
Museum of Zoology, Lund University, Lund, Sweden 


MZUR
Museum of Zoology, Università di Roma “La Sapienza”, Roma [Rome], Italy 


NHMB
 Naturhistorisches Museum Basel, Basel, Switzerland 


NHMW
Naturhistorisches Museum Wien, Wien [Vienna], Austria 


NHRS
Naturhistoriska Riksmuseet [Swedish Museum of Natural History], Stockholm, Sweden 


NMB
National Museum, Bloemfontein, South Africa 


NMBA
Naturhistorisches Museum der Benediktiner-Abtei Admont, Admont, Austria 


NMBZ
Natural History Museum of Zimbabwe, Bulawayo, Zimbabwe [formerly National Museum of Southern Rhodesia] 


NMDA
 Department of Arthropoda, KwaZulu-Natal Museum, Pietermaritzburg, South Africa 


NMCL
Naturkunde-Museum Coburg, Coburg, Germany 


NMNW
National Museum of Namibia, Windhoek, Namibia 


RMNH
Naturalis Biodiversity Center, Leiden, Netherlands [formerly Nationaal Natuurhistorisch Museum and before that Rijksmuseum van Natuurlijke Historie]. The Zoölogisch Museum of the University of Amsterdam [as ZMAN] closed recently and the collections were merged with those of RMNH



SAMC
 Iziko South African Museum, Cape Town, South Africa 


SANC
South African National Collection of Insects, ARC, Plant Protection Research Institute, Pretoria, South Africa [former acronym as PPRI] 


SDEI
Senckenberg Deutsches Entomologisches Institut, Leibniz-Zentrums für Agrarlandschaftsforschung, Müncheberg, Germany 


SMF
 Forschungsinstitut und Naturmuseum Senckenberg, Frankfurt am Main, Germany 


SMNS
Staatliches Museum für Naturkunde, Stuttgart, Germany 


TAU
Tel Aviv University, Tel Aviv, Israel 


USNM
 National Museum of Natural History [formerly United States National Museum], Smithsonian Institution, Washington, USA 


ZIN
Zoological Institute, Russian Academy of Sciences, St. Petersburg, Russia 


ZMHB
Museum für Naturkunde [formerly associated with Humboldt-Universität], Leibniz-Institut für Evolutions- und Biodiversitätsforschung, Berlin, Germany 


ZMUC
Zoological Museum, Natural History Museum of Denmark, University of Copenhagen, Copenhagen, Denmark 


ZMUH
Zoologisches Institut und Zoologisches Museum, Universität von Hamburg, Germany 


ZMUK
 Zoologisches Museum der Christian-Albrechts-Universität zu Kiel, Kiel, Germany 


ZMUM
Zoological Museum, Moscow State University, Moscow, Russia 


### Geographic divisions

The known distribution of each tachinid species recorded from the Afrotropical Region is given next to the valid name in the following order: Afrotropical Region, Palaearctic Region, Oriental Region, Australasian and Oceanian regions [cited as Australasian for brevity], Nearctic Region, and Neotropical Region. Each of these regions is subdivided according to the scheme explained below. Areas close to the Afrotropical Region are subdivided more finely than those that are distant from it. Spellings of countries and areas within countries follow, with few exceptions, *The Times Comprehensive Atlas of the World* (Times Books 2007). The abbreviations and names given below are those used for the distributions given in the Catalogue section.

#### Afrotropical Region (Fig. 1)

**Figure 1.**
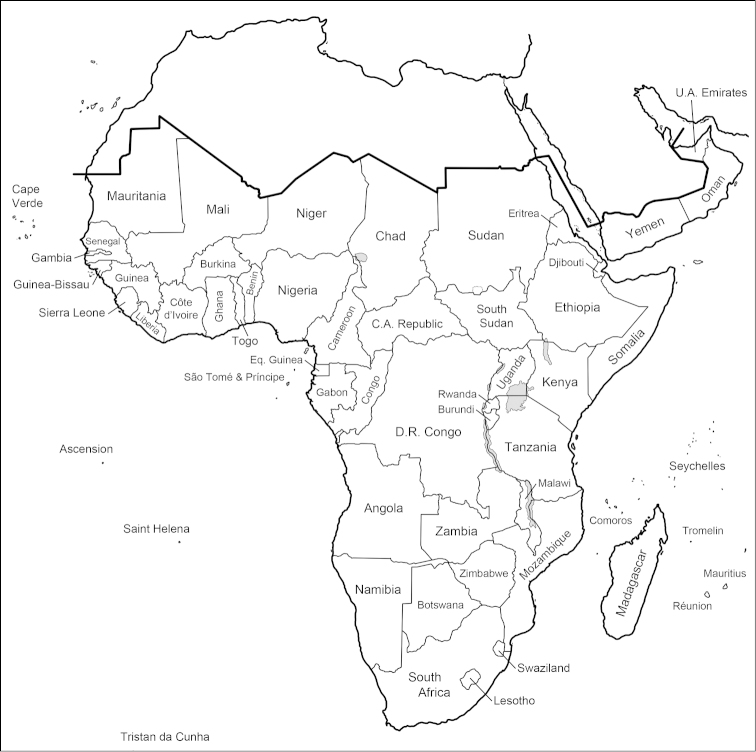
Countries and major islands of the Afrotropical Region. These are used for distributions within the Afrotropical Region and are listed (with annotations) under Geographic Divisions in the Materials and methods section.

The geographic limits of the Afrotropical Region follow Crosskey (1980a) except for the addition of Oman and United Arab Emirates (formerly part of the Palaearctic Region) to conform to the *Manual of Afrotropical Diptera* that is currently in preparation (A.H. Kirk-Spriggs and B.J. Sinclair, editors).

The names of countries and islands listed below and shown in Fig. 1 have in a few instances changed from those given in Crosskey (1980a). These changes are noted in the following list. Two new countries have formed since the last catalogue: Eritrea (formerly part of Ethiopia) and South Sudan (formerly part of Sudan). It is not possible to divide old distribution records from “Sudan” into the present countries of Sudan and South Sudan and hence Sudan is used in the sense of both countries in the Catalogue section. There are several nominal species with type localities in this greater Sudan and in all cases these localities are in the present country of Sudan; i.e., no species was described from South Sudan.

Angola.

Ascension (an island dependency of the United Kingdom Overseas Territory of Saint Helena).

Benin.

Botswana.

Burkina [Burkina Faso] (as Upper Volta in Crosskey 1980a).

Burundi.

Cameroon (as Cameroun in Crosskey 1980a).

Cape Verde [Cape Verde Islands].

C.A. Republic [Central African Republic].

Chad.

Comoros [Comoros Islands].

Congo.

Côte d’Ivoire [or Ivory Coast].

Djibouti.

D.R. Congo [Democratic Republic of the Congo] (as Zaire in Crosskey 1980a).

Eq. Guinea [Equatorial Guinea] (including Annobón and Bioco [as “Fernando Póo”] islands of Crosskey 1980a).

Eritrea (new country since Crosskey 1980a [formerly part of Ethiopia]).

Ethiopia.

Gabon.

Gambia [The Gambia].

Ghana.

Guinea.

Guinea-Bissau.

Kenya.

Lesotho.

Liberia.

Madagascar.

Malawi.

Mali.

Mauritania.

Mauritius (including Cargados Carajos and Rodrigues islands of Crosskey 1980a).

Mozambique.

Namibia.

Niger.

Nigeria.

Oman (not included in Afrotropical Region of Crosskey 1980a).

Réunion (France).

Rwanda.

Saint Helena (United Kingdom Overseas Territory).

São Tomé & Príncipe (treated separately in Crosskey 1980a).

Senegal.

Seychelles (including Aldabra, Amirante, Astove, Coëtivy, and Cosmoledo islands of Crosskey 1980a).

Sierra Leone.

Somalia.

South Africa.

South Sudan (see note for Sudan; new country since Crosskey 1980a [formerly part of Sudan]).

Sudan (including, for distributional purposes, South Sudan).

Swaziland.

Tanzania.

Togo.

Tristan da Cunha (an island dependency of the United Kingdom Overseas Territory of Saint Helena).

Tromelin (disputed island territory of France).

U.A. Emirates [United Arab Emirates] (not included in Afrotropical Region of Crosskey 1980a)

Uganda.

Yemen (including South Yemen and Suquţrá [as Socotra] of Crosskey 1980a).

Zambia.

Zimbabwe (as Rhodesia in Crosskey 1980a).

#### Palaearctic Region (Fig. 2)

**Figure 2.**
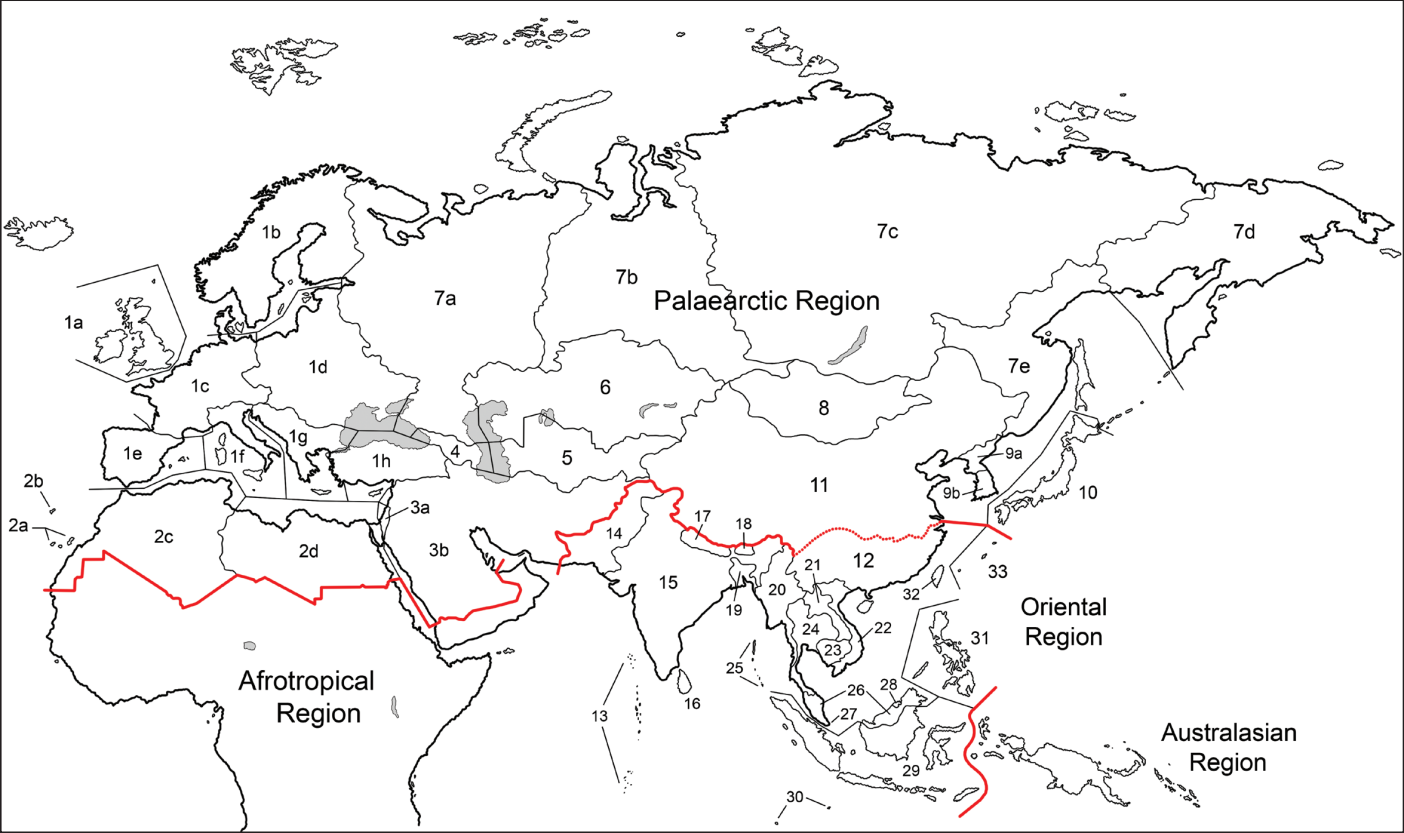
Subdivisions of the Palaearctic and Oriental regions used for distributions outside the Afrotropical Region. The numbers correspond to the countries or areas listed under Geographic Divisions in the Materials and methods section.

The traditional limits of the Palaearctic Region are recognized except that Oman and United Arab Emirates are assigned to the Afrotropical Region to conform with the upcoming *Manual of Afrotropical Diptera* and the boundary with the Oriental Region through China is as newly defined under Oriental China (area 12). The subdivisions of the Palaearctic Region are explained below and are shown in Fig. 2, where they are labelled according to the following numbering scheme.

1. Europe.

a. British Is. [British Isles].—United Kingdom and Republic of Ireland.

b. Scand. [Scandinavia].—Iceland, Denmark (excluding Greenland), Norway, Sweden, and Finland.

c. W. Eur. [Western Europe].—Austria, Belgium, Channel Islands, France (excluding Corse), Germany, Liechtenstein, Luxembourg, Netherlands, and Switzerland.

d. E. Eur. [Eastern Europe].—Belarus, Czech Republic, Estonia, Hungary, Kaliningradskaya [or Kaliningrad] Oblast’ (Russia), Latvia, Lithuania, Moldova, Poland, Romania, Slovakia, and Ukraine.

e. SW. Eur. [Southwestern Europe].—Andorra, Portugal (including Azores, excluding Madeira), and Spain (excluding Canary Islands).

f. SC. Eur. [Southcentral Europe].—Corse (France), Italy, Malta, Monaco, and San Marino.

g. SE. Eur. [Southeastern Europe].—Albania, Bosnia and Herzegovina, Bulgaria, Croatia, Greece, Montenegro, Macedonia, Serbia, and Slovenia.

h. Turkey.—Cyprus and Turkey.

2. N. Africa [North Africa].

a. Canary Is. [Canary Islands].—Canary Islands (Spain).

b. Madeira.—Madeira (Portugal).

c. NW. Africa [Northwestern Africa].—Algeria, Morocco, Tunisia, and Western Sahara.

d. NE. Africa [Northeastern Africa].—Egypt and Libya.

3. M. East [Middle East].

a. Israel (treated as a separate division because the Tachinidae are significantly better known from Israel than from the other countries of the Middle East).

b. M. East [Middle East] (excluding Israel).—Afghanistan, Bahrain, Iran, Iraq, Jordan, Kuwait, Lebanon, Oman, [Occupied] Palestinian territories, Qatar, Saudi Arabia, and Syria.

4. Transcaucasia.—Armenia, Azerbaijan, and Georgia.

5. C. Asia [Central Asia].—Kyrgyzstan, Tajikistan, Turkmenistan, and Uzbekistan.

6. Kazakhstan.

7. Russia [or Russian Federation].

a. W. Russia [Western Russia, excluding Kaliningradskaya Oblast’].—Bordering Scandinavia and Eastern Europe to the west, Transcaucasia to the south, Ural Mountains to the east, and Kazakhstan to the southeast.

b. W. Siberia [Western Siberia].—Bordering Western Russia to the west, Kazakhstan and Mongolia to the south, and Yenisey River to the east.

c. E. Siberia [Eastern Siberia].—Bordering Western Siberia to the west, Mongolia and China to the south, and Russian administrative divisions of Chukotskiy [or Chukotka] Avtonomnyy Okrug, Magadanskaya [or Magadan] Oblast’, Khabarovskiy [or Khabarovsk] Kray, and Amurskaya [or Amur] Oblast’ to the east.

d-e. Far East [Russian Far East].—Bordering Eastern Siberia to the west, China and North Korea to the south, and Japan to the southeast.

d. N. Far East [Northern Russian Far East].—Russian administrative divisions of Chukotskiy Avtonomnyy Okrug, Magadanskaya Oblast’, and Kamchatskiy [or Kamchatka] Kray.

e. S. Far East [Southern Russian Far East].—Russian administrative divisions of Khabarovskiy Kray, Amurskaya Oblast’, Yevreyskaya [or Jewish] Avtonomnaya Oblast’, and Sakhalinskaya [or Sakhalin] Oblast’ (including Kuril Islands).

8. Mongolia.

9. Korea.—North and South Korea. Cited as Korea when more detailed distributional data is not available.

a. N. Korea [North Korea].

b. S. Korea [South Korea].

10. Japan (excluding Ryukyu I.).

11. Pal. China [Palaearctic China]. North of the dotted line in Fig. 2, comprising that part of China not listed for Oriental China.

#### Oriental Region (Fig. 2)

The Oriental Region is bounded on the south by Weber’s Line (following Evenhuis 1989) and on the north and west by the Palaearctic Region. The subdivisions of the Oriental Region are explained below and are shown on Fig. 2, where they are labelled according to the following numbering scheme.

12. Orien. China [Oriental China]. The Oriental portion of China is newly defined here as comprising the southern half of Chongqing, Fujian, Guangdong, Guangxi, Guizhou, Hainan, Hong Kong, Hunan, Jiangxi, Macau, Shanghai, southern half of Sichuan, most of Yunnan except for the extreme northwest portion, and Zhejiang. A species recorded from Palaearctic China and additionally Sichuan and/or Yunnan, with no other records from Oriental China, is recorded only from Palaearctic China; e.g., *Periscepsia carbonaria* (Panzer).

13. Maldives etc.—Maldives, Lakshadweep (India), British Indian Ocean Territory [or Chagos Archipelago] (United Kingdom Overseas Territory).

14. Pakistan.

15. India.

16. Sri Lanka.

17. Nepal.

18. Bhutan.

19. Bangladesh.

20. Myanmar [or Burma].

21. Laos.

22. Vietnam.

23. Cambodia.

24. Thailand.

25. Andaman & Nicobar Is.—Andaman and Nicobar Islands (India).

26. Malaysia.

27. Singapore.

28. Brunei.

29. Indonesia (Oriental part as delimited by Weber’s Line; mainly Borneo, Jawa [or Java], Lesser Sunda Islands, Sulawesi [or Celebes], and Sumatera [or Sumatra]).

30. Christmas & Cocos Is.—Territories of Christmas Island and Cocos [or Keeling] Islands (Australia).

31. Philippines.

32. Taiwan.

33. Ryukyu Is.—Ryukyu Islands [or Nansei-shotō] (Japan).

#### Australasian and Oceanian regions

These regions are combined under the title of Australasian Region for the purposes of this catalogue. The combined region is bounded on the north by the Oriental Region (Weber’s Line) and is subdivided as follows.

N. Australasian.—Indonesia (Australasian part as delimited by Weber’s Line; mainly Maluku [or Moluccas] Islands, Western New Guinea [or Irian Jaya], and Papua New Guinea (including Bismarck Archipelago).

Australia.

Hawaii.—Hawaiian Islands (USA).

Melanesia.—Melanesia (excluding Papua New Guinea and Bismarck Archipelago, listed as part of N. Australasian), principally Fiji, New Caledonia (France), Solomon Islands, and Vanuatu.

Micronesia.—Federated States of Micronesia, principally Guam (USA), Kiribati, Marshall Islands, Nauru, Northern Mariana Islands (USA), and Palau.

New Zealand.

Polynesia.—Polynesia (excluding New Zealand and Hawaii, each listed separately), principally American Samoa (USA), Cook Islands (New Zealand), Easter Island (Chile), French Polynesia (France), Niue (New Zealand), Pitcairn Islands (United Kingdom), Samoa, Tokelau (New Zealand), Tonga, Tuvalu, and Wallis and Futuna (France).

#### Nearctic Region

The Nearctic Region is arbitrarily defined as America north of Mexico for the purposes of this catalogue, including Greenland (Denmark) and Bermuda (United Kingdom Overseas Territory) but not Hawaii (USA) and the West Indies (following O’Hara and Wood 2004). The Nearctic Region is not subdivided in this catalogue but individual distributions are given for species recorded from the region.

#### Neotropical Region

This region is bounded on the north by the Nearctic Region. There are only three species recorded from the region in this catalogue: *Leucostoma simplex* (Fallén), *Trichopoda giacomellii* (Blanchard) (introduced into South Africa and establishment unknown), and *Voria ruralis* (Fallén).

### Sample distribution

A species recorded from all regions and subdivisions recognized here would be cited with the following distribution:

Afrotropical: Angola, Ascension, Benin, Botswana, Burkina, Burundi, Cameroon, Cape Verde, C.A. Republic, Chad, Comoros, Congo, Côte d’Ivoire, Djibouti, D.R. Congo, Eq. Guinea, Eritrea, Ethiopia, Gabon, Gambia, Ghana, Guinea, Guinea-Bissau, Kenya, Lesotho, Liberia, Madagascar, Malawi, Mali, Mauritania, Mauritius, Mozambique, Namibia, Niger, Nigeria, Oman, Réunion, Rwanda, Saint Helena, São Tomé & Príncipe, Senegal, Seychelles, Sierra Leone, Somalia, South Africa, Sudan, Swaziland, Tanzania, Togo, Tristan da Cunha, Tromelin, U.A. Emirates, Uganda, Yemen, Zambia, Zimbabwe. Palaearctic: C. Asia, Europe (British Is., Scand., W. Eur., E. Eur., SW. Eur., SC. Eur., SE. Eur., Turkey) [or Europe (all), if recorded from all subdivisions], Japan, Kazakhstan, Korea (N. Korea, S. Korea), M. East (Israel, M. East) [or M. East (all)], Mongolia, N. Africa (Canary Is., Madeira, NW. Africa, NE. Africa) [or N. Africa (all)], Pal. China, Russia (W. Russia, W. Siberia, E. Siberia, N. Far East, S. Far East) [or Russia (all)], Transcaucasia. Oriental: Andaman & Nicobar Is., Bangladesh, Bhutan, Brunei, Cambodia, Christmas & Cocos Is., India, Indonesia, Laos, Malaysia, Maldives etc., Myanmar, Nepal, Orien. China, Pakistan, Philippines, Ryukyu Is., Singapore, Sri Lanka, Taiwan, Thailand, Vietnam. Australasian: Australia, Hawaii, Melanesia, Micronesia, N. Australasian, New Zealand, Polynesia. Nearctic: [individual distribution]. Neotropical: [individual distribution].

### Distributional data

#### Distributions within the Afrotropical Region

Distributions are cited for each species based on published records, examination of specimens in collections, and material collected by the authors (primarily PC) or made available to us by colleagues (see Acknowledgements). The principle source for published records was Crosskey (1980b), which was generally the starting point for the distributions cited here. Crosskey’s distributions were augmented or revised based on subsequent literature. As well, his generalized ranges given for widespread species were further detailed to the country level, as much as possible, using earlier literature. In such instances we cited Crosskey’s generalized range (e.g., “widespread trop. Afr. & sthn Afr.” for *Hermya diabolus*) and followed this with a list of countries for which we found records.

#### Distributions outside the Afrotropical Region

The primary sources for extralimital distributions were Herting (1984) and Herting and Dely-Draskovits (1993) for the Palaearctic Region, *Fauna Europaea* (Tschorsnig et al. 2004) for Europe, Tschorsnig and Báez (2002) for Spain, Portugal and Andorra (and associated islands), Cerretti and Freidberg (2009) for Israel, Cerretti (2010) for Italy, Dawah (2011) for Saudi Arabia, Zeegers (2007) for Yemen, Zeegers (2010) for United Arab Emirates, Richter (2004) for the Russian Far East, Shima (2014) for Japan, O’Hara et al. (2009) for China, Crosskey (1976) for the Oriental Region, Cantrell and Crosskey (1989) for the Australasian and Oceanian regions, *Australian Faunal Directory* (Ginn 2012) for Australia, O’Hara and Wood (2004) for America north of Mexico (herein as Nearctic Region), and Guimarães (1971) for America south of the United States (herein as Neotropical Region). Other literature on the Tachinidae published after the foregoing sources supplemented these primary references. See notes in O’Hara et al. (2009: 20) regarding the interpretation of records given as Mongolia and West and East Siberia in Herting and Dely-Draskovits (1993).

## Classification

The classification adopted here recognizes the usual four subfamilies—Dexiinae, Exoristinae, Phasiinae and Tachininae—a classificatory scheme that has been generally accepted since the time of Herting’s (1984) Palaearctic catalogue (e.g., Wood 1987, Tschorsnig and Richter 1998, Ziegler 1998, O’Hara and Wood 2004, Richter 2004, O’Hara et al. 2009, Cerretti 2010, and Shima 2014). Crosskey (1980b) recognized five subfamilies, the aforementioned four (the Exoristinae as Goniinae) plus the Dufouriinae. The last was recognized by Verbeke (1962a) in his detailed study of tachinid male terminalia but most authors since Herting (1984) have afforded this taxon tribal status within the Dexiinae.

At the tribal level, the greatest difference between the classification of Crosskey (1980b) and modern works was in the treatment of what Crosskey (1973b, 1976) called the “Goniini-Carceliini-Sturmiini-Eryciini complex”. In general terms, the Goniini and Sturmiini of this complex are now united under Goniini and the other two are united under Eryciini (see O’Hara 2013 for a historical review). Females of the Goniini produce microtype eggs and those of the Eryciini produce macrotype eggs, but adult external morphology does not always separate them (e.g., Cerretti et al. 2015). This continues to be a problem for some Afrotropical taxa of Goniini–Erycini of unknown reproductive habit and “ambiguous” external morphology.

A few tribes have been moved to other subfamilies since Crosskey (1980b). The Neaerini have been split into the Graphogastrini and Neaerini and transferred, along with the Siphonini, from the Exoristinae (as Goniinae) to the Tachininae. The genus *Sarrorhina* Villeneuve is moved herein from its placement by Crosskey (1980b) in the Minthoini (Tachininae) to the Graphogastrini, **comb. n.** The tribe Acemyini is retained in the Exoristinae where most authors, including Crosskey (1980b), have placed it. O’Hara and Wood (2004) and O’Hara et al. (2009) treated it under Tachininae but the recent phylogenetic studies of Cerretti et al. (2014) and Winkler et al. (2015) strongly support its placement in the Exoristinae.

The Campylochetini, Thelairiini, Voriini and Wagneriini were recognized as distinct tribes within the Tachininae by Crosskey (1980b). They have since been moved to the Dexiinae but there has been no consensus on how to treat them tribally with the exception of Wagneriini being included within the Voriini. O’Hara and Wood (2004) recognized the Campylochetini, Thelairiini and Voriini as distinct tribes, O’Hara et al. (2009) recognized only the Campylochetini and Voriini (with Thelairiini included in the latter), and other authors placed all of these taxa in the Voriini (e.g., Herting 1984, Tschorsnig and Herting 1994, Ziegler 1998, Cerretti 2010). We have elected to recognize the single tribe Voriini, which is consistent with the phylogenetic analysis of Cerretti et al. (2014).

The Eloceriini, Linnaemyini, and Loewiini were recognized as tribes (within the Tachininae) by Crosskey (1980b) and subsequent authors have treated them in various ways. These taxa, along with the Polideini (a non-Afrotropical tribe) and Bigonichetini (formerly the Triarthriini) are related in a manner that is not yet clear (O’Hara 2002, Cerretti et al. 2014). For present purposes we recognize two tribes in the Afrotropics, the Ernestiini (including Eloceriini, Linnaemyini and Loewiini) and Bigonichetini (represented by *Trichactia* Stein). *Trichactia* was the only member of the Eloceriini recognized from the Afrotropics by Crosskey (1980b) and is treated herein as the only member of the Bigonichetini.

The Eutherini, a small tribe with one of its two genera (*Euthera* Loew) present in the Afrotropics, have the distinction of being one of only two tribes in recent decades to have been treated in the Phasiinae by some authors (e.g., Crosskey 1980b, Herting 1984, Ziegler 1998, Richter 2004) and in the Dexiinae by others (e.g., Shima 1989, O’Hara and Wood 2004, Cerretti 2010). We follow the latter placement and consider the tribe as possibly a basal member of the subfamily, as first suggested by Shima (1989) and recently supported by Winkler et al. (2014, 2015).

The Imitomyiini have also been treated in the Phasiinae by some authors (e.g., Herting 1984, Richter 2004) and in the Dexiinae by others (e.g., O’Hara and Wood 2004, Cerretti 2010), but were placed by Crosskey (1980b) in the Dufouriinae following Verbeke (1962a). The tribe is placed in the Phasiinae herein as supported by the morphological analysis of Cerretti et al. (2014). *Imitomyia* Townsend with *Diplopota* Bezzi in synonymy was recognized as the sole Afrotropical genus of the Imitomyiini by Crosskey (1980b, 1984). Herting (1984) treated both *Imitomyia* and *Diplopota* as valid but the synonymy of Crosskey is followed here, as it was by O’Hara (2014).

Another tribe of enigmatic placement, the Strongygastrini, is newly recorded from the Afrotropical Region. The tribe has typically been considered an unusual member of the Phasiinae (because it is ovolarviparous and not restricted to parasitizing heteropterans) (e.g., Verbeke 1962a, Herting 1984, Tschorsnig 1985, Shima 2014) but has been treated in the Tachininae by some recent authors (e.g., O’Hara and Wood 2004, O’Hara et al. 2009, Cerretti 2010). The morphological analysis of Cerretti et al. (2014) and molecular analyses of Winkler et al. (2014, 2015) support the former view and this placement of the Strongygastrini in Phasiinae is followed herein. The Strongygastrini are represented in the Afrotropical Region by a single genus, *Rondaniooestrus* Villeneuve. Our treatment of *Rondaniooestrus* and the non-Afrotropical genus *Strongygaster* Macquart in the Strongygastrini (in Phasiinae) was first advanced by Verbeke (1962a). Crosskey (1980b) placed *Rondaniooestrus* in the monotypic tribe Rondaniooestrini in the Tachininae, but Tschorsnig (1985) tentatively agreed with Verbeke (1962a) and preliminary unpublished data arising from the *Phylogeny and Evolution of World Tachinidae* project (see Stireman et al. 2013 for project overview) also supports a close relationship between *Rondaniooestrus* and *Strongygaster*.

Crosskey (1976) noted similarities between the Glaurocarini and Ormiini but retained the two as separate tribes in the Tachininae, as did Crosskey (1980b). Tschorsnig (1985) combined them under the Ormiini and Ziegler (1998) supported this grouping. We prefer to recognize the Glaurocarini and Ormiini as distinct tribes pending further study of their relationships.

Within the Phasiinae, the Cinochirini of Crosskey (1980b) have been included within Leucostomatini since Herting (1984) and this arrangement is followed herein. *Gymnosoma* Meigen was placed in Phasiini by Crosskey (1980b) and Herting (1984) but Tschorsnig (1985) recognized a monophyletic Gymnosomatini based on derived features of the male terminalia. Included within Gymnosomatini were the Afrotropical genera *Bogosia* Rondani and *Bogosiella* Villeneuve and the New World genus *Trichopoda* Berthold. Contemporaneous and subsequent authors have been split in their treatment of the *Gymnosoma* group, some continuing to include it in the Phasiini (e.g., Barraclough 1985a, Tschorsnig and Herting 1994, Richter 2004, O’Hara et al. 2009, Cerretti 2010, Shima 2014) and others recognizing it as a tribe (e.g., Ziegler 1998, O’Hara and Wood 2004). *Trichopoda* (of unconfirmed presence as an introduction in South Africa) and related genera have continued to be treated in the literature in the Trichopodini (O’Hara and Wood 2004, Cerretti 2010). However, the recent analysis of Cerretti (2014) and our own studies provide support for the Gymnosomatini *sensu* Tschorsnig (1985) and we recognize this tribe herein with the genera *Bogosia*, *Bogosiella*, *Gymnosoma* and *Trichopoda*. Based on our own research we transfer herein *Paraclara* Bezzi (as *Clara* Brauer & Bergenstamm in Crosskey 1980b) from the Cylindromyiini to Hermyini, **comb. n.**

With respect to the priority of family-group names, Sabrosky (1999) is followed unless otherwise noted. We recognize subfamilies, tribes (but not subtribes), genera, subgenera, species, and in rare instances (in deference to existing literature) subspecies (only for the three species *Trigonospila prasius* Mesnil, *Siphona fuliginea* Mesnil and *Siphona reducta* Mesnil).

### Review of generic changes since Crosskey’s Afrotropical catalogue

There has been no dramatic reappraisal of the Afrotropical genera of Tachinidae since Crosskey (1980b) but instead a steady succession of taxonomic papers that have introduced gradual changes to the generic classification over the past 35 years. The genera involved and associated references are listed in this section as a review of the changes that have shaped the current generic classification of Afrotropical Tachinidae. The section “Summary of new taxonomic and nomenclatural changes” gives the changes to the current classification that are introduced in this catalogue.

Three lists are given in this section. In the first list that follows are the genera and subgenera that have been described or recorded from the Afrotropical Region since Crosskey (1980b), or were treated under other generic names in that work and have since been recognized as valid names.

*Acemya* Robineau-Desvoidy, 1830 (Zeegers 2007, 2010).

*Amnonia* Kugler, 1971 (Zeegers 2010).

*Anomalostomyia* Cerretti & Barraclough, 2007.

*Apomorphomyia* Crosskey, 1984.

*Brachychaetoides* Mesnil, 1970 (treated as a synonym of *Chlorolydella* Townsend, 1933 by Crosskey (1980b) but reinstated as a valid name by Crosskey (1984)).

*Calliethilla* Shima, 1979 (Cerretti 2012).

*Calyptromyia* Villeneuve, 1915 (Dear 1981).

*Campylocheta* Rondani, 1859 (as *Elpe* Robineau-Desvoidy, 1863 in Crosskey (1980b), a genus subsequently synonymized with *Campylocheta*).

*Chryserycia* Mesnil, 1977 (described from Madagascar by Mesnil (1977b), a paper missing from Crosskey (1980b)).

*Clairvilliops* Mesnil, 1959 (treated as a synonym of *Dionaea* Robineau-Desvoidy, 1830 by Crosskey (1980b) and as a synonym of *Clairvillia* Robineau-Desvoidy, 1830 by Crosskey (1984) but subsequently recognized as a valid name).

*Clausicella* Rondani, 1856 (as *Istoglossa* Rondani, 1856 in Crosskey (1980b), a genus subsequently synonymized with *Clausicella*).

*Conopomima* Mesnil, 1978 (published too late to be included in Crosskey (1980b) but was listed in the simultaneously published Appendix of Crosskey (1980a)).

*Crassicornia* Kugler, 1980.

*Dionomelia* Kugler, 1978 (Zeegers 2010).

*Estheria* Robineau-Desvoidy, 1830 (as *Dolichodexia* Brauer & Bergenstamm, 1889 in Crosskey (1980b), a genus subsequently synonymized with *Estheria*).

*Eugaedioxenis* Cerretti, O’Hara & Stireman, 2015 (Cerretti et al. 2015).

Exoristella Herting, 1984 as subgenus of Exorista Meigen, 1803.

*Istocheta* Rondani, 1859 (as *Prosopofrontina* Townsend, 1926 in Crosskey (1980b), a genus subsequently synonymized with *Istocheta*).

*Kaiseriola* Mesnil, 1970 (treated as a synonym of *Diaprochaeta* Mesnil, 1970 by Crosskey (1980b) but reinstated as a valid name by Crosskey (1984)).

*Kuwanimyia* Townsend, 1916 (Cerretti 2009b).

*Lydella* Robineau-Desvoidy, 1830 (as *Metoposisyrops* Townsend, 1916 in Crosskey (1980b), a genus synonymized with *Lydella* by Woodley (1994)).

*Mediosetiger* Barraclough, 1983.

*Meigenia* Robineau-Desvoidy, 1830 (Zeegers 2007).

*Minthosoma* Zeegers, 2007.

*Montanothalma* Barraclough, 1996.

*Myxogaedia* Mesnil, 1956 (treated as a synonym of *Pretoriana* Curran, 1938 by Crosskey (1980b) and changed to the valid name of the genus by O’Hara (2011)).

*Nardia* Cerretti, 2009.

*Nealsomyia* Mesnil, 1939 (Cerretti 2005).

*Neophryxe* Townsend, 1916 (Cerretti 2012).

*Nilea* Robineau-Desvoidy, 1863 (recorded from Madagascar by Mesnil (1977b), a paper missing from Crosskey (1980b)).

*Ossidingia* Townsend, 1919 (treated as a synonym of *Nemorilla* Rondani, 1856 by Crosskey (1980b) but reinstated as a valid name by Crosskey (1984)).

*Paraclara* Bezzi, 1908 (treated as a synonym of *Clara* Brauer & Bergenstamm, 1889 by Crosskey (1980b) but corrected to the valid name of the genus in the simultaneously published Appendix of Crosskey (1980a)).

*Phasia* Latreille, 1804 (as *Alophora* Robineau-Desvoidy, 1830 in Crosskey (1980b), a genus subsequently synonymized with *Phasia*).

*Piligenoides* Barraclough, 1985.

*Pseudalsomyia* Mesnil, 1968 (Cerretti 2012).

Ptilotachina Brauer & Bergenstamm, 1891 as subgenus of Exorista Meigen, 1803.

*Ramonella* Kugler, 1980 (Zeegers 2007).

*Rhinophoroides* Barraclough, 2005.

*Rhynchogonia* Brauer & Bergenstamm, 1893 (Zeegers 2010).

*Rossimyiops* Mesnil, 1953 (Cerretti et al. 2009).

*Schembria* Rondani, 1861 (Crosskey 1984, Barraclough 1991).

Senometopia Macquart, 1834 was treated as a subgenus of Carcelia Robineau-Desvoidy, 1830 by Crosskey (1980b) but has subsequently been recognized as a separate genus.

*Smidtia* Robineau-Desvoidy, 1830 (as *Timavia* Robineau-Desvoidy, 1863 in Crosskey (1980b), a genus subsequently synonymized with *Smidtia*).

Spixomyia Crosskey, 1967 as subgenus of Exorista Meigen, 1803.

*Stomina* Robineau-Desvoidy, 1830 (undetermined species noted by Mesnil (1975a), Crosskey (1984) and Zeegers (2007)).

*Stylocarcelia* Zeegers, 2007.

*Thrixion* Brauer & Bergenstamm, 1889 (Zeegers 2007).

*Trichopoda* Berthold, 1827 (two species introduced into South Africa in the 1990s but no confirmation of establishment).

In the second list below are given genus-group names that have changed status since Crosskey (1980b). Some are treated as the valid names of subgenera in the current literature but none as the valid name of a genus. Names mentioned in the comments in the list above are not repeated here (i.e., *Alophora*, *Clara*, *Dolichodexia*, *Elpe*, *Istoglossa*, *Metoposisyrops*, *Pretoriana*, *Prosopofrontina*, and *Timavia*).

Alophorella Townsend, 1912 was treated as a subgenus of Alophora Robineau-Desvoidy, 1830 by Crosskey (1980b). It is currently recognized as a synonym of *Phasia* Latreille, 1804. Subgenera of *Phasia* are not recognized herein because the Afrotropical species have been insufficiently studied.

*Asiphona* Mesnil, 1954 was treated as a genus by Crosskey (1980b) but has subsequently been synonymized with Siphona subgenus Aphantorhaphopsis Townsend, 1926.

*Carcelita* Mesnil, 1975 was treated as a *nomen nudum* by Crosskey (1980b) but has subsequently been recognized as a subgenus of *Carcelia* Robineau-Desvoidy, 1830 (e.g., Cerretti and Freidberg 2009).

Caricelia Mesnil, 1975 was treated as a subgenus of Carcelia Robineau-Desvoidy, 1830 by Crosskey (1980b) but has subsequently been synonymized with Carcelia subgenus Carcelita Mesnil, 1975.

*Ceranthia* Robineau-Desvoidy, 1830 was treated as a genus by Crosskey (1980b) but has subsequently been recognized as a subgenus of *Siphona* Meigen, 1803.

*Cuphocera* Macquart, 1845 was treated as a genus by Crosskey (1980b) but has subsequently been synonymized with *Peleteria* Robineau-Desvoidy, 1830.

*Elfia* Robineau-Desvoidy, 1850 was treated as a genus by Crosskey (1980b) but has subsequently been synonymized with *Phytomyptera* Rondani, 1845 (a genus also recognized as valid by Crosskey 1980b).

*Mapolomyia* Verbeke, 1960 was treated as a genus by Crosskey (1980b) but has subsequently been synonymized with *Cahenia* Verbeke, 1960 by Crosskey (1984).

*Mormonomyia* Brauer & Bergenstamm, 1891 was treated as a subgenus of *Alophora* Robineau-Desvoidy, 1830 by Crosskey (1980b). It is currently recognized as a synonym of *Phasia* Latreille, 1804. Subgenera of *Phasia* are not recognized herein because the Afrotropical species have been insufficiently studied.

*Palexorista* Townsend, 1921 was treated as a genus by Crosskey (1980b) but has subsequently been recognized as a subgenus of *Drino* Robineau-Desvoidy, 1863.

*Phaniola* Mesnil, 1978 was listed as a genus by Crosskey (1981a) in the Appendix to the Afrotropical catalogue but was placed in synonymy with *Catapariprosopa* Townsend, 1927 by Crosskey (1984).

*Podotachina* Brauer & Bergenstamm, 1891 was treated as a synonym of *Exorista* Meigen, 1803 by Crosskey (1980b) but has subsequently been recognized as a subgenus of *Exorista*.

*Stomatomyia* Brauer & Bergenstamm, 1889 was treated as a genus by Crosskey (1980b) but has subsequently been recognized as a subgenus of *Chetogena* Rondani, 1856. Subgenera of *Chetogena* are not recognized herein because the Afrotropical species have been insufficiently studied.

*Tricoliga* Rondani, 1856 was treated as a synonym of *Exorista* Meigen, 1803 by Crosskey (1980b, spelled as *Thrycolyga*) but has subsequently been recognized as a subgenus of *Exorista*.

*Trypherosoma* Verbeke, 1962 was treated as a genus by Crosskey (1980b) but was subsequently synonymized with *Gynandromyia* Bezzi, 1923 by Crosskey (1984).

*Zelindomyia* Verbeke, 1962 was treated as a genus by Crosskey (1980b) but was subsequently synonymized with *Gynandromyia* Bezzi, 1923 by Crosskey (1984).

*Ziminiola* Mesnil, 1978 was treated as a genus by Crosskey (1980b) but is a junior homonym of *Ziminiola* Gerasimov, 1930 and has subsequently been replaced by the name *Mesnilus* Özdikmen, 2007.

*Zygobothria* Mik, 1891 was treated as a genus by Crosskey (1980b) but has subsequently been recognized as a subgenus of *Drino* Robineau-Desvoidy, 1863.

In the third list below are given the genus-group names that were treated as valid by Crosskey (1980b) and are still valid but the genera are no longer recognized from the Afrotropical Region.

*Clairvillia* Robineau-Desvoidy, 1830 was treated as a genus by Crosskey (1980b) but the single Afrotropical species assigned to it (*Clairvillia breviforceps* van Emden, 1954) has subsequently been placed under *Clairvilliops* Mesnil, 1959.

*Dexiotrix* Villeneuve, 1936 was treated as a genus by Crosskey (1980b) but the single Afrotropical species assigned to it (*Dexiotrix empiformis* Mesnil, 1976) was reassigned to *Trixa* Meigen, 1824 by Zhang and Shima 2005. *Dexiotrix empiformis* is reassigned to *Mesnilotrix* gen. n. herein.

*Dionaea* Robineau-Desvoidy, 1830 was treated as a genus by Crosskey (1980b) but the single Afrotropical species assigned to it (*Dionaea inermis* Mesnil, 1959) has subsequently been placed in synonymy with *Clairvilliops breviforceps* (van Emden, 1954).

*Gymnophryxe* Villeneuve, 1922 was treated as a genus by Crosskey (1980b) but the single Afrotropical species assigned to it (*Archiclops africanum* Mesnil, 1968) was moved to *Brachychaetoides* Mesnil, 1970 by Crosskey (1984).

### Recent family reassignment of copal inclusions from East Africa

Two copal inclusions from East Africa were believed to be Baltic amber fossils of Tachinidae until Grimaldi et al. (1994) corrected their age and geographic origin. After study of the inclusions, O’Hara et al. (2013) determined that *Paleotachina smithii* Townsend (1921: 134) is a junior synonym of *Aethiopomyia gigas* (Stein, 1906) in the family Muscidae and *Electrotachina smithii* Townsend (1938a: 166) is a species, possibly extant, belonging to the genus *Dolichotachina* Villeneuve (1913b: 112) in the family Sarcophagidae. Both *Paleotachina* and *Electrotachina* were described as monotypic genera with *Paleotachina smithii* and *Electrotachina smithii* as their type species, respectively. Neither inclusion was thought to be of East African origin at the time of Crosskey (1980a, b).

### Summary of new taxonomic and nomenclatural changes

#### New genera and species

*Afrophylax* Cerretti & O’Hara. Type species: *Sturmia aureiventris* Villeneuve, 1910, by designation herein. **Gen. n.**

*Austrosolieria* Cerretti & O’Hara. Type species: *Austrosolieria londti* Cerretti & O'Hara, **sp. n.**, by designation herein. **Gen. n.**

*Austrosolieria freidbergi* Cerretti & O’Hara. **Sp. n.** (Malawi).

*Austrosolieria londti* Cerretti & O’Hara. **Sp. n.** (South Africa).

*Carceliathrix* Cerretti & O’Hara. Type species: *Phorocera crassipalpis* Villeneuve, 1938, by designation herein. **Gen. n.**

*Filistea* Cerretti & O’Hara. Type species: *Viviania aureofasciata* Curran, 1927, by designation herein. **Gen. n.**

*Filistea verbekei* Cerretti & O’Hara. **Sp. n.** (Cameroon, D.R. Congo, Uganda).

*Mesnilotrix* Cerretti & O’Hara. Type species: *Dexiotrix empiformis* Mesnil, 1976, by designation herein. **Gen. n.**

*Myxophryxe* Cerretti & O’Hara. Type species: *Phorocera longirostris* Villeneuve, 1938, by designation herein. **Gen. n.**

*Myxophryxe murina* Cerretti & O’Hara. **Sp. n.** (South Africa).

*Myxophryxe regalis* Cerretti & O’Hara. **Sp. n.** (South Africa).

*Myxophryxe satanas* Cerretti & O’Hara. **Sp. n.** (South Africa).

*Stiremania* Cerretti & O’Hara. Type species: *Stiremania karoo* Cerretti & O’Hara, **sp. n.**, by designation herein. **Gen. n.**

*Stiremania karoo* Cerretti & O’Hara. **Sp. n.** (South Africa).

*Stiremania robusta* Cerretti & O’Hara. **Sp. n.** (South Africa).

#### Genera newly recorded from the Afrotropical Region

The following genera are newly recorded from the Afrotropical Region based on species that were placed in other genera by Crosskey (1980b).

*Madremyia* Townsend, 1916 (one species placed in *Phryxe* Robineau-Desvoidy, 1830 by Crosskey (1980b)). **New record.**

*Paratrixa* Brauer & Bergenstamm, 1891 (two species placed in *Medina* Robineau-Desvoidy, 1830 by Crosskey (1980b)). **New record.**

The following genus is newly recorded from the Afrotropical Region based on a described species not previously reported from the region.

*Simoma* Aldrich, 1926 (based on new record of *Simoma grahami* Aldrich). **New record.**

#### Genera no longer recognized from the Afrotropical Region

The following genera, which are currently recorded from the Afrotropical Region in the literature (e.g., O’Hara 2014), are no longer recognized from the region.

*Calozenillia* Townsend, 1927 [Oriental; also Australasian and Palaearctic]. The two species placed under *Calozenillia* by Crosskey (1980b: 869, as new combinations) are moved herein to the reinstated genus *Perlucidina* Mesnil, 1952 (treated as a synonym of *Calozenillia* by Crosskey (1980b)).

*Eurysthaea* Robineau-Desvoidy, 1863 [Palaearctic; also Oriental and Australasian]. The single species recognized under *Eurysthaea* by Crosskey (1980b: 878), *Ceromasia rufiventris* Curran, 1927, is moved herein to “Unplaced species of Goniini”.

*Trixa* Meigen, 1824 [Palaearctic; also Oriental]. *Dexiotrix empiformis* Mesnil, 1976 from Madagascar was transferred to *Trixa* by Zhang and Shima (2005: 59), resulting in the first record of the genus from the Afrotropical Region. *Dexiotrix empiformis* is reassigned to *Mesnilotrix* gen. n. herein.

#### Species newly recorded from the Afrotropical Region

The following species are newly recorded from the Afrotropical Region. New country records for Afrotropical species are noted in the Catalogue section.

*Amnonia carmelitana* Kugler, 1971 (Ethiopia, Kenya).

*Simoma grahami* Aldrich, 1926 (Namibia).

#### Species misidentified or misrecorded from the Afrotropical Region

Species that are newly recognized as misidentified or misrecorded from the Afrotropical Region are listed here.

*Euthera peringueyi* Bezzi, 1925 [Oriental]. The type locality was originally given as “Chabra, Congo” and on this basis *Euthera peringueyi* was recorded from “Congo: Chabra” by van Emden (1960: 383), from “‘Congo’ [? Zaire]: Chabra” and India by Crosskey (1976: 175), and from “‘Congo’” and India by Crosskey (1980b: 829). The type locality is recognized herein as Chapra in West Bengal, India and *Euthera peringueyi* is no longer recorded from the Afrotropical Region.

*Hamaxia incongrua* Walker, 1860 [Australasian; also Oriental and Palaearctic]. Recorded from Tanzania by Verbeke (1960: 335) and from “? E. Africa” by Crosskey (1976: 184); not listed in Crosskey (1980b). Treated herein as misidentified from the Afrotropical Region.

*Leucostoma tetraptera* (Meigen, 1824) [Palaearctic]. Recorded from Botswana, Nigeria and South Africa by Crosskey (1980b: 829), probably based on misidentifications.

#### New replacement names

Five new names are proposed for preoccupied names of Afrotropical species. Preoccupied names that are currently recognized as junior synonyms are not renamed in this work.

*Billaea rubida* O’Hara & Cerretti is proposed as a *nomen novum* for *Phorostoma rutilans* Villeneuve, 1916, a name preoccupied in the genus *Billaea* Robineau-Desvoidy, 1830 by *Musca rutilans* Fabricius, 1781 [Nearctic]. **Nom. n.**

*Cylindromyia braueri* O’Hara & Cerretti is proposed as a *nomen novum* for *Ocyptera nigra* Villeneuve, 1918, a name preoccupied in the genus *Cylindromyia* Meigen, 1803 by *Glossidionophora nigra* Bigot, 1885 [Neotropical]. **Nom. n.**

*Cylindromyia rufohumera* O’Hara & Cerretti is proposed as a *nomen novum* for *Ocyptera scapularis* Villeneuve, 1944, a junior primary homonym of *Ocyptera scapularis* Loew, 1845 [Palaearctic]. **Nom. n.**

*Phytomyptera longiarista* O’Hara & Cerretti is proposed as a *nomen novum* for *Phytomyzoneura aristalis* Villeneuve, 1936, a name preoccupied in the genus *Phytomyptera* Rondani, 1845 by *Phasiostoma aristalis* Townsend, 1915 [Nearctic]. **Nom. n.**

Siphona (Siphona) pretoriana O’Hara & Cerretti is proposed as a *nomen novum* for *Siphona laticornis* Curran, 1941, a name preoccupied in the genus *Siphona* Meigen, 1803 by *Actia laticornis* Malloch, 1930 [Oriental]. **Nom. n.**

#### New type species fixations

Article 70.3.2 of the *Code* (ICZN 1999) allows the type species of a nominal genus to be fixed as the species intended by the original author if the type species designated by that author was misidentified. We have invoked Article 70.3.2 for the two instances of misidentified type species in this catalogue that had not been dealt with previously (e.g., O’Hara and Wood 2004, O’Hara et al. 2009) to preserve the current concepts of the genera involved. Type species are fixed for the following nominal genera (see Catalogue section for further details).

*Lydellina* Villeneuve, 1916c: 490. Type species newly fixed as *Lydellina villeneuvei* Townsend, 1933. Valid generic name.

*Sericophoromyia* Austen, 1909: 95. Type species newly fixed as *Tachina quadrata* Wiedemann, 1830. Synonym of *Winthemia* Robineau-Desvoidy, 1830.

#### Lectotype designations

Lectotypes are designated for the following nominal species (see Lectotype Designations section).

*Degeeria crocea* Villeneuve, 1950. This is a valid name in the genus *Medina* Robineau-Desvoidy, 1830, as *Medina crocea* (Villeneuve).

*Degeeria semirufa* Villeneuve, 1950. This is a valid name in the genus *Medina* Robineau-Desvoidy, 1830, as *Medina semirufa* (Villeneuve).

*Erycia brunnescens* Villeneuve, 1934. This is a valid name in the genus *Thelairosoma* Villeneuve, 1916, as *Thelairosoma brunnescens* (Villeneuve).

*Exorista oculata* Villeneuve, 1910. This is a valid name in the genus Carcelia Robineau-Desvoidy, 1830 (subgenus Carcelita Mesnil, 1975), as Carcelia (Carcelita) oculata (Villeneuve).

*Kiniatilla tricincta* Villeneuve, 1938. This is a valid name in the genus *Kiniatilla* Villeneuve, 1938.

*Myxarchiclops caffer* Villeneuve, 1916. This is a valid name in the genus *Myxarchiclops* Villeneuve, 1916.

*Ocyptera linearis* Villeneuve, 1936. This is a junior synonym in the genus *Cylindromyia* Meigen, 1803. The valid name of the species is *Cylindromyia soror* (Wiedemann, 1830).

*Peristasisea luteola* Villeneuve, 1934. This is a valid name in the genus *Peristasisea* Villeneuve, 1934.

*Phorocera crassipalpis* Villeneuve, 1938. This valid name is designated as the type species of *Carceliathrix* Cerretti & O’Hara, gen. n.

#### New and revived status

Changes to genus-group names

*Bogosiella* Villeneuve, 1923, which was synonymized with *Phasia* Latreille, 1804 by Sun and Marshall (2003: 19), is reinstated as a valid name. **Status revived.**

*Dyshypostena* Villeneuve, 1939, which was treated as a junior synonym of *Sumpigaster* Macquart, 1855 by Crosskey (1980b: 842, 1984: 252), is reinstated as a valid name. **Status revived.**

*Perlucidina* Mesnil, 1952, which was synonymized with *Calozenillia* Townsend, 1927 by Crosskey (1980b: 869) and retained in synonymy by Crosskey (1984: 199), is reinstated as a valid name. **Status revived.**

*Thelymyiops* Mesnil, 1950, which was originally proposed as a subgenus of *Carcelia* Robineau-Desvoidy, 1830 and was treated as such by Crosskey (1980b: 866, 1984: 279), is removed from *Carcelia* and elevated to full genus status. **Status n.**

#### Changes to species-group names

*Besseria fossulata* Bezzi, 1908, which was treated as a junior synonym of *Actia zonaria* Loew, 1847 in the genus *Besseria* Robineau-Desvoidy by Crosskey (1980b: 826), is elevated to valid name *Besseria fossulata* Bezzi. **Status revived.**

*Degeeria cinctella* Villeneuve, 1950, which was treated as a junior synonym of *Degeeria lateralis* Villeneuve, 1950 in the genus *Medina* Robineau-Desvoidy by Crosskey (1980b: 857), is elevated to valid name *Medina cinctella* (Villeneuve). **Status revived.**

*Nemoraea miranda intacta* Villeneuve, 1916, which was treated as a valid name by Curran (1936: 14) and later as a junior synonym of *Nemoraea miranda* Villeneuve, 1916 by Crosskey (1980b: 843), is elevated to valid name *Nemoraea intacta* Villeneuve. **Status revived.**

*Succingulum exiguum* Villeneuve, 1935, which was treated as a junior synonym of *Succingulum mista* Villeneuve, 1913 in the genus *Trigonospila* Pokorny by Crosskey (1980b: 858), is elevated to valid name *Trigonospila exigua* (Villeneuve). **Status revived.**

*Wagneria rufitibia abbreviata* Mesnil, 1950, which was treated as a junior synonym of *Wagneria rufitibia* Villeneuve, 1938 in the genus *Periscepsia* Gistl by Crosskey (1980b: 839), is elevated to valid name *Periscepsia abbreviata* (Mesnil). **Status n.**

*Wagneria rufitibia nudinerva* Mesnil, 1950, which was treated as a junior synonym of *Wagneria rufitibia* Villeneuve, 1938 in the genus *Periscepsia* Gistl by Crosskey (1980b: 839), is elevated to valid name *Periscepsia nudinerva* (Mesnil). **Status n.**

#### New and revived combinations

New and revived combinations proposed in this work are listed below. These are based on the study of type material, authoritatively identified specimens, and/or descriptions and figures in the literature by PC.

*Afrosturmia orbitalis* Curran, 1927 (type species of *Afrosturmia* Curran) is moved from its original placement in *Afrosturmia* to *Blepharella* Macquart (with *Afrosturmia* in synonymy). **Comb. n.**

*Alsomyia chloronitens* Mesnil, 1977, which was published too late to be included in Crosskey (1980b), is moved to *Nealsomyia* Mesnil. **Comb. n.**

*Bogosiella pomeroyi* Villeneuve, 1923 (type species of *Bogosiella* Villeneuve) is returned to *Bogosiella* from its placement in *Phasia* Latreille by Sun and Marshall (2003: 19). **Comb. revived.**

*Campylochaeta violacea* Curran, 1927 is moved to *Brachychaetoides* Mesnil from its placement in *Chlorolydella* Townsend by Crosskey (1980b: 877, 1984: 286). **Comb. n.**

*Ceromasia rufiventris* Curran, 1927 is moved to Goniini, and treated as an unplaced species within the tribe, from its placement in *Eurysthaea* Robineau-Desvoidy by Crosskey (1980b: 878, 1984: 295). **Comb. n.**

*Degeeria profana* Karsch, 1888 is moved to *Sturmia* Robineau-Desvoidy from its placement in “Unplaced species of Goniinae” by Crosskey (1980b: 881). **Comb. n.**

*Dexia buccata* van Emden, 1947 is moved to *Estheria* Robineau-Desvoidy from its treatment as a “species of uncertain generic affiliation” by Crosskey (1984: 240). **Comb. n.**

*Dexiomera surda* Curran, 1933 (type species of *Dexiomera* Curran) is moved from its original placement in *Dexiomera* to *Estheria* Robineau-Desvoidy (with *Dexiomera* in synonymy). **Comb. n.**

*Dexiotrix empiformis* Mesnil, 1976 is moved to *Mesnilotrix* gen. n. from its placement in *Trixa* Meigen by Zhang and Shima (2005: 59). **Comb. n.**

*Dyshypostena tarsalis* Villeneuve, 1939 (type species of *Dyshypostena* Villeneuve) is returned to *Dyshypostena* Villeneuve from its placement in *Sumpigaster* Macquart by Crosskey (1980b: 842, 1984: 252). **Comb. revived.**

*Exorista africana* Jaennicke, 1867 is moved to *Perlucidina* Mesnil from its placement in *Calozenillia* Townsend by Crosskey (1980b: 869, 1984: 281). **Comb. n.**

*Exorista perlucida* Karsch, 1886 (type species of *Perlucidina* Mesnil) is returned to *Perlucidina* from its placement in *Calozenillia* Townsend by Crosskey (1980b: 869, 1984: 281). **Comb. revived.**

*Hemiwinthemia stuckenbergi* Verbeke, 1973 is moved to Leskiini, and treated as an unplaced species within the tribe, from its original placement in *Hemiwinthemia* Verbeke. **Comb. n.**

*Kinangopana edwardsi* van Emden, 1960 (type species of *Kinangopana* van Emden) is moved from its original placement in *Kinangopana* to *Dyshypostena* Villeneuve (with *Kinangopana* in synonymy). **Comb. n.**

*Metadrinomyia whitmorei* Cerretti, 2012 is moved to *Charitella* Mesnil from its original placement in *Metadrinomyia* Shima. **Comb. n.**

*Ocyptera retroflexa* Villeneuve, 1944 is moved to *Prolophosia* Townsend from its placement in *Cylindromyia* Meigen by Crosskey (1980b: 827). **Comb. n.**

*Paratrixa aethiopica* Mesnil, 1952 is returned to *Paratrixa* Brauer & Bergenstamm from its placement in *Medina* Robineau-Desvoidy by Crosskey (1980b: 857). **Comb. revived.**

*Paratrixa stammeri* Mesnil, 1952 is returned to *Paratrixa* Brauer & Bergenstamm from its placement in *Medina* Robineau-Desvoidy by Crosskey (1980b: 857). **Comb. revived.**

*Phorocera clausa* Curran, 1940 is moved to *Nealsomyia* Mesnil from its placement in “Unplaced species of Goniinae” by Crosskey (1980b: 881). **Comb. n.**

*Phorocera crassipalpis* Villeneuve, 1938 is moved to *Carceliathrix* gen. n. (and designated as its type species) from its placement in “Unplaced species of Carceliini” by Crosskey (1980b: 867). **Comb. n.**

*Phorocera longirostris* Villeneuve, 1938 is moved to *Myxophryxe* gen. n. (and designated as its type species) from its placement in *Pretoriana* Curran, 1938 by Crosskey (1980b: 879). **Comb. n.**

*Phryxe setinervis* Mesnil, 1968 is moved to *Madremyia* Townsend from its original placement in *Phryxe* Robineau-Desvoidy. **Comb. n.**

*Sturmia aureiventris* Villeneuve, 1910 is moved to *Afrophylax* gen. n. (and designated as its type species) from its placement in “Unplaced species of Carceliini” by Crosskey (1980b: 867). **Comb. n.**

*Sturmia longicauda* Mesnil, 1970 is moved to *Nilea* Robineau-Desvoidy from its original placement in *Sturmia* Robineau-Desvoidy. **Comb. n.**

*Viviania aureofasciata* Curran, 1927 is moved to *Filistea* gen. n. (and designated as its type species) from its placement in “Unplaced species of Tachinidae” by Crosskey (1980b: 881). **Comb. n.**

#### New and revived synonymies

New and revived generic and specific synonymies are proposed for the names below. As with the new and revived combinations listed above, they result from the study of type material, authoritatively identified specimens, and/or descriptions and figures in the literature by PC.

*Afrosturmia* Curran, 1927, which was treated as a genus by Crosskey (1980b: 867, 1984: 283), is synonymized with *Blepharella* Macquart, 1851. **Syn. n.**

*Archiphania* van Emden, 1945 was treated as a genus by Crosskey (1980b) but was synonymized with *Catharosia* Rondani, 1868 by Crosskey (1984). Zeegers (2007) recognized *Archiphania* as a genus but we follow Crosskey (1984) in treating it as a synonym of *Catharosia*. **Syn. revived.**

*Besseria longicornis* Zeegers, 2007 is synonymized with *Besseria fossulata* Bezzi, 1908. The current combination is *Besseria fossulata* Bezzi. **Syn. n.**

*Dexiomera* Curran, 1933, which was treated as a genus by Crosskey (1980b: 832, 1984: 239), is synonymized with *Estheria* Robineau-Desvoidy, 1830. **Syn. n.**

*Hemiwinthemia francoisi* Verbeke, 1973, which was overlooked by Crosskey (1980b) and later treated as a species of *Hemiwinthemia* Villeneuve by Crosskey (1984: 201), is synonymized with *Nemoraea capensis* Schiner, 1868. The current combination is *Smidtia capensis* (Schiner). **Syn. n.**

*Kinangopana* van Emden, 1960, which was treated as a genus by Crosskey (1980b: 841, 1984: 252), is synonymized with *Dyshypostena* Villeneuve, 1939. **Syn. n.**

*Metadrinomyia* Shima, 1980 is synonymized with *Charitella* Mesnil, 1957. **Syn. n.**

*Phorocera majestica* Curran, 1940 is synonymized with *Phorocera longirostris* Villeneuve, 1938. The current combination is *Myxophryxe longirostris* (Villeneuve). **Syn. n.**

*Podomyia discalis* Curran, 1939 is synonymized with *Antistasea fimbriata* Bischof, 1904. The current combination is *Antistasea fimbriata* Bischof. **Syn. n.**

## Catalogue

### Subfamily DEXIINAE (Fig. 3)

**Figure 3.**
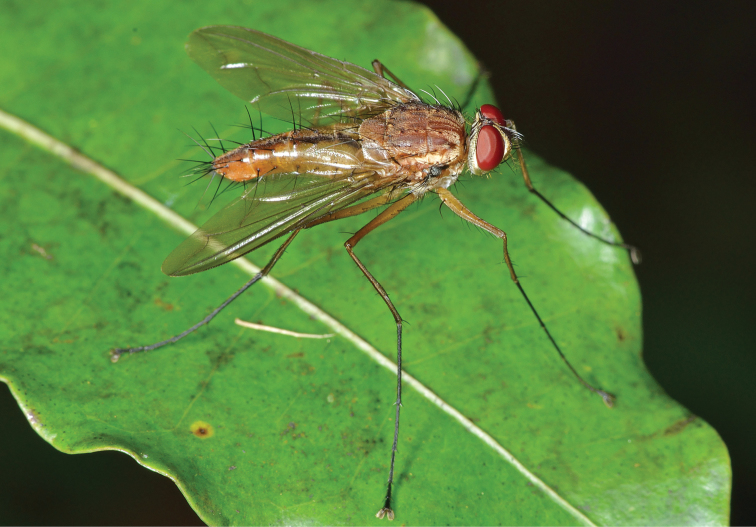
Live specimen of *Chaetodexia* sp. (Dexiini, Dexiinae) from Andasibe, Madagascar (image courtesy of S.A. Marshall).

#### Tribe DEXIINI

##### Genus *BILLAEA* Robineau-Desvoidy, 1830

***BILLAEA*** Robineau-Desvoidy, 1830: 328. Type species: *Billaea grisea* Robineau-Desvoidy, 1830 (= *Dexia pectinata* Meigen, 1826), by monotypy [Palaearctic].

*OMALOGASTER* Macquart, 1834: 51 [also 1834: 187]. Type species: *Billaea grisea* Robineau-Desvoidy, 1830 (= *Dexia pectinata* Meigen, 1826), by subsequent designation of Townsend (1916b: 8) [Palaearctic].

*GIGAMYIA* Macquart, 1844: 115 [also 1844: 272]. Type species: *Stomoxys gigantea* Wiedemann, 1824, by original designation.

*HOMALOGASTER* Agassiz, 1846b: 184. Unjustified emendation of *Omalogaster* Macquart, 1834.

*PARAPROSENA* Brauer & Bergenstamm, 1889: 127 [also 1890: 59]. Type species: *Paraprosena waltlii* Brauer & Bergenstamm, 1889 (= *Dexia marmorata* Meigen, 1838), by monotypy [Palaearctic].

*GYMNODEXIA* Brauer & Bergenstamm, 1891: 364 [also 1891: 60]. Type species: *Dexia triangulifera* Zetterstedt, 1844, by subsequent designation of Brauer (1893: 505) [Palaearctic].

*AMPHIBOLIOPSIS* Townsend, 1926b: 538. Type species: *Gymnostylia minor* Villeneuve, 1913, by original designation.

*CHAETOBILLAEA* Mesnil, 1976: 44 (as subgenus of *Billaea* Robineau-Desvoidy, 1830). Type species: Billaea (Chaetobillaea) communis Mesnil, 1976, by original designation.

***africana*** (Villeneuve, 1935).—Afrotropical: D.R. Congo, Ethiopia, Kenya, South Africa, Tanzania.

*Paraprosena marmorata africana* Villeneuve, 1935a: 138. Syntypes, 5 males (possibly 1 male in CNC). Type locality: Kenya.

*Billaea neavei* van Emden, 1947: 643. Holotype male (BMNH). Type locality: Kenya, Marsabit [as “Marsabit, Rendili Njoro, N. Frontier District”].

Note: Cooper and O’Hara (1996: 58) recorded a male in CNC as a syntype of *Paraprosena marmorata africana* Villeneuve, 1935. The specimen is from “Ilesha, S. Nigeria”, which is not the type locality of “l’Afrique orientale anglaise” [= Kenya] given by Villeneuve (1935a: 138). However, the CNC specimen bears a handwritten Villeneuve type label and is perhaps one of the five males mentioned in the original description.

***capensis*** van Emden, 1947.—Afrotropical: South Africa.

*Billaea capensis* van Emden, 1947: 645. Holotype male (BMNH). Type locality: South Africa, Western Cape, 40 miles from Cape Town, Viljoen’s Pass [as “Viljoeus Pass”, ca. 34°5′S 19°3′E].

***communis*** Mesnil, 1976.—Afrotropical: Madagascar.

Billaea (Chaetobillaea) communis Mesnil, 1976: 45. Holotype male (MNHN). Type locality: Madagascar, Antananarivo, Manjakatompo [ca. 19°21′S 47°18′E].

***decisa*** (Curran, 1927).—Afrotropical: D.R. Congo.

*Gymnodexia decisa* Curran, 1927a: 7. Holotype male (AMNH). Type locality: D.R. Congo, Orientale, Kisangani [as “Stanleyville”].

***edwardsi*** (van Emden, 1947).—Afrotropical: Uganda.

*Paraprosena edwardsi* van Emden, 1947: 658. Holotype female (BMNH). Type locality: Uganda, Rwenzori Range [as “Ruwenzori”], Mobuku Valley, 7300ft.

***gigantea*** (Wiedemann, 1824).—Afrotropical: South Africa.

*Stomoxys gigantea* Wiedemann, 1824: 41. Type(s), female (1 syntype in ZMUC, Zimsen 1954: 21). Type locality: South Africa, Western Cape, Cape of Good Hope [as “Prom. bon. sp.” = “Promontorium Bonae Spei”].

***grandis*** Mesnil, 1976.—Afrotropical: Madagascar.

Billaea (Chaetobillaea) grandis Mesnil, 1976: 46. Holotype male (MNHN). Type locality: Madagascar, Toliara, “Andohahelo” [presumably Parc National d’Andohahela], 1800m.

***interrupta*** (Curran, 1927).—Afrotropical: D.R. Congo.

*Gymnodexia interrupta* Curran, 1927a: 8. Holotype male (AMNH). Type locality: D.R. Congo, Orientale, Kisangani [as “Stanleyville”].

Note: *Billaea interrupta* (Curran, 1927) is a senior secondary homonym of *Billaea interrupta* (Curran, 1929), a name currently treated as valid in the Nearctic Region (O’Hara and Wood 2004: 23). The junior homonym, *Billaea interrupta* (Curran, 1929), is not renamed here but will be dealt with in a future publication on Nearctic Tachinidae.

***lateralis*** (Curran, 1927).—Afrotropical: D.R. Congo.

*Gymnodexia lateralis* Curran, 1927a: 6. Holotype male (AMNH). Type locality: D.R. Congo, Orientale, Kisangani [as “Stanleyville”].

***lativentris*** van Emden, 1947.—Afrotropical: Kenya.

*Billaea lativentris* van Emden, 1947: 646. Holotype male (BMNH). Type locality: Kenya, Mt. Elgon, 10,500–11,500ft.

***minor*** (Villeneuve, 1913).—Afrotropical: D.R. Congo, Ethiopia, Kenya, South Africa, Uganda.

*Gymnostylia minor* Villeneuve, 1913c: 37. Lectotype male (SAMC, examined by JEOH), by fixation of Townsend (1938b: 316) (mention of “Ht male” from Natal in Rambouillet [Villeneuve’s personal collection, since dispersed] is regarded as a lectotype fixation). Type locality: South Africa, KwaZulu-Natal (Newcastle according to label data).

Note: *Gymnostylia minor* Villeneuve, 1913 was described from two males, one from “Natal” (South Africa) and the other from Kericho (Kenya) and dated “27-XI-1911”. Villeneuve (1913c: 37) gave the depository for the syntype from Kericho as BMNH but the specimen there is dated “20.I.1913” (D. Whitmore, pers. comm.). The specimen collected on the correct date is in CNC (Brooks et al. 2007: 33) and it is accepted here as the paralectotype. Arnaud (1982: 12) cited a “type” from Kenya in MSNM but did not provide data supporting its status as an original syntype. The male in SAMC labelled “Natal, Newcastle” is assumed to be the lectotype fixed by Townsend (1938b: 316).

***orbitalis*** van Emden, 1947.—Afrotropical: South Africa.

*Billaea orbitalis* van Emden, 1947: 644. Holotype male (BMNH). Type locality: South Africa, Western Cape, Malgas [as “Malagas”].

***ovata*** Mesnil, 1976.—Afrotropical: Madagascar.

Billaea (Chaetobillaea) ovata Mesnil, 1976: 45. Holotype male (MNHN). Type locality: Madagascar, Fianarantsoa, Ranohira.

***rhingiaeformis*** van Emden, 1959.—Afrotropical: Ethiopia.

*Billaea rhingiaeformis* van Emden, 1959: 186. Holotype male (BMNH). Type locality: Ethiopia, Simien Mountains, Lori, 11,500ft [ca. 13°17′N 38°12′E, see map in Scott (1958, inserted between pp. 58–59)].

***rubida*** O’Hara & Cerretti, **nom. n.**—Afrotropical: South Africa.

*Phorostoma rutilans* Villeneuve, 1916c: 504 (junior secondary homonym of *Musca rutilans* Fabricius, 1781). Syntypes, males (1 male in CNC, MSNM [1 “cotype” according to Arnaud 1982: 13], 7 males in SAMC [examined by JEOH]). Type locality: South Africa, KwaZulu-Natal.

*Billaea rubida* O’Hara & Cerretti, **nom. n.** for *Phorostoma rutilans* Villeneuve, 1916.

Note: *Phorostoma rutilans* Villeneuve, 1916 is a junior secondary homonym of *Musca rutilans* Fabricius, 1781, the valid name of a Nearctic species of *Billaea* (O’Hara and Wood 2004: 23). We hereby propose the new name *Billaea rubida* to replace the preoccupied name *Phorostoma rutilans* Villeneuve. The same type material applies to the new name. The specific epithet *rubida* is formed from the Latin *rubidus*, meaning reddish, alluding to the reddish portions of the abdomen mentioned in the original description and which presumably inspired Villeneuve’s name *rutilans*.

***setosa*** (Macquart, 1844).—Afrotropical: South Africa.

*Gymnostylia setosa* Macquart, 1844: 88 [also 1844: 245]. Syntypes, males and females (lost, Crosskey 1971: 271). Type locality: South Africa, Western Cape, Cape of Good Hope [as “Cap”].

***sjostedti*** Speiser, 1910.—Afrotropical: Ethiopia, Kenya, Tanzania, Uganda.

*Billaea sjostedti* Speiser, 1910: 146 (as “*sjöstedti*”). Lectotype male (NHRS), by fixation of Villeneuve (1914a: 439) (mention of “type (♂)” in NHRS is regarded as a lectotype fixation). Type locality: Tanzania, Mt. Kilimanjaro [as “Kilimandjaro”].

Note: *Billaea sjostedti* Speiser, 1910 was described from two males from the area of “Kilimandjaro”, with one male further restricted to “Kibonoto” [now Kibongoto] at 1000m. Villeneuve (1914a: 439) did not specify which of the two males is the “type (♂)” that he examined, but it is presumed to be identifiable (and distinguishable from the other syntype, if still extant) in NHRS as the syntype accepted here as lectotype.

***solivaga*** Mesnil, 1976.—Afrotropical: Madagascar.

Billaea (Chaetobillaea) solivaga Mesnil, 1976: 46. Holotype male (MNHN). Type locality: Madagascar, Toamasina, Périnet [ca. 18°55′S 48°25′E].

***vanemdeni*** Fennah, 1959.—Afrotropical: Ghana.

*Billaea vanemdeni* Fennah, 1959: 682. Holotype male (BMNH). Type locality: Ghana, Tafo, West African Cacao Research Institute.

***velutina*** Mesnil, 1976.—Afrotropical: Madagascar.

*Billaea velutina* Mesnil, 1976: 42. Holotype male (MNHN). Type locality: Madagascar, Toamasina, south of Moramanga, Ampetameloka, 840m.

***versicolor*** (Curran, 1927).—Afrotropical: D.R. Congo.

*Gymnodexia versicolor* Curran, 1927a: 7. Holotype male (AMNH). Type locality: D.R. Congo, Orientale, Kisangani [as “Stanleyville”].

***villeneuvei*** (Curran, 1927).—Afrotropical: D.R. Congo.

*Gymnodexia villeneuvei* Curran, 1927a: 5. Holotype male (AMNH). Type locality: D.R. Congo, Orientale, Kisangani [as “Stanleyville”].

***vitripennis*** Mesnil, 1950.—Afrotropical: Zimbabwe.

Billaea (Homalogaster) vitripennis Mesnil, 1950d: 116. Syntypes, males and females (“Plusieurs exemplaires”) (1 male in CNC). Type locality: Zimbabwe, Hurungwe [as “Urungwe”], Gota Gota.

##### Genus *CHAETODEXIA* Mesnil, 1976

***CHAETODEXIA*** Mesnil, 1976: 49. Type species: *Chaetodexia keiseri* Mesnil, 1976, by original designation.

***keiseri*** Mesnil, 1976.—Afrotropical: Madagascar.

*Chaetodexia keiseri* Mesnil, 1976: 50. Holotype male (MNHN). Type locality: Madagascar, Antsiranana, Joffreville.

***nigrescens*** Mesnil, 1976.—Afrotropical: Madagascar.

*Chaetodexia keiseri nigrescens* Mesnil, 1976: 50. Holotype male (MNHN). Type locality: Madagascar, Toamasina, Périnet [ca. 18°55′S 48°25′E].

***pallida*** Mesnil, 1976.—Afrotropical: Madagascar.

*Chaetodexia pallida* Mesnil, 1976: 50. Holotype male (MNHN). Type locality: Madagascar, Toliara, Ambatolahy [ca. 19°54′S 45°23′E].

***trilineata*** Mesnil, 1976.—Afrotropical: Madagascar.

*Chaetodexia trilineata* Mesnil, 1976: 51. Holotype male (MNHN). Type locality: Madagascar, Fianarantsoa, Vohiparara [within Parc National de Ranomafana, which is located at ca. 21°13′S 47°26′E].

##### Genus *DEXIA* Meigen, 1826

***DEXIA*** Meigen, 1826: 33. Type species: *Musca rustica* Fabricius, 1775, by designation under the Plenary Powers of ICZN (1988: 74) [Palaearctic].

*DEXILLA* Westwood, 1840: 140. Type species: *Musca rustica* Fabricius, 1775, by original designation [Palaearctic].

***aurohumera*** van Emden, 1947.—Afrotropical: Mozambique.

*Dexia aurohumera* van Emden, 1947: 634. Holotype male (BMNH). Type locality: Mozambique, Maputo [as “Lorenzo Marques”].

***capensis*** Robineau-Desvoidy, 1830.—Afrotropical: Kenya, South Africa, Tanzania.

*Dexia capensis* Robineau-Desvoidy, 1830: 314. Type(s), unspecified sex (MNHN or lost). Type locality: South Africa, Western Cape, Cape of Good Hope [as “Cap de Bonne-Espérance”].

*Dexia afra* Curran, 1927f: 104. Holotype male (BMNH). Type locality: South Africa, KwaZulu-Natal, Durban.

***cuthbertsoni*** (Curran, 1941).—Afrotropical: Kenya, Liberia, Nigeria, Sierra Leone, Zimbabwe.

*Dexilla cuthbertsoni* Curran, 1941: 1. Holotype female (AMNH). Type locality: Zimbabwe, Vumba Mountains.

*Dexilla bequaerti* Curran, 1941: 2. Holotype male (AMNH). Type locality: Liberia, Du River Camp No. 3.

Note: The relative priority of *Dexilla cuthbertsoni* Curran, 1941 and *Dexilla bequaerti* Curran, 1941, when the two are treated as synonyms, was established by van Emden (1947: 637), as the First Reviser (Article 24.2.2 of the *Code*, ICZN 1999).

***inappendiculata*** Austen, 1909.—Afrotropical: D.R. Congo, Uganda.

*Dexia inappendiculata* Austen, 1909: 97. Syntypes, 2 males (BMNH). Type locality: Uganda, Rwenzori Range [as “Ruwenzori”], 7000–8000ft.

*Dexia monticola* Villeneuve, 1935a: 137. Holotype male (CNC). Type locality: Uganda, Rwenzori Range [as “Ruwenzori”], 1900m.

***orphne*** Curran, 1927.—Afrotropical: Kenya.

*Dexia orphne* Curran, 1927f: 105. Holotype male (BMNH). Type locality: Kenya, Amboseli National Park [as “Southern Masai Reserve”].

***pollinosa*** Villeneuve, 1943.—Afrotropical: Nigeria, Tanzania.

*Dexia pollinosa* Villeneuve, 1943b: 94. Syntypes, 2 males (1 male in CNC). Type locality: northern Nigeria, Abinsi.

***rhodesia*** (Curran, 1941).—Afrotropical: Ghana, Mozambique, Tanzania, Zimbabwe.

*Dexilla rhodesia* Curran, 1941: 2. Holotype female (AMNH). Type locality: Zimbabwe, Harare [as “Salisbury”].

***torneutopoda*** (Speiser, 1914).—Afrotropical: Cameroon, Nigeria.

*Dolichodexia torneutopoda* Speiser, 1914: 10. Syntypes, 2 males (1 syntype in SDEI, Rohlfien and Ewald 1974: 145). Type locality: Cameroon.

*Dexia venusta* Curran, 1927f: 105. Holotype male (SDEI). Type locality: southern Nigeria [as “N. Cameroons”; Northern Cameroons became part of Nigeria in 1961].

***uelensis*** van Emden, 1954.—Afrotropical: D.R. Congo.

*Dexia uelensis* van Emden, 1954: 551. Holotype male (MRAC). Type locality: D.R. Congo, Orientale, Uele, Bambesa.

***uniseta*** Curran, 1927.—Afrotropical: Kenya, Malawi, South Africa, Tanzania, Uganda.

*Dexia uniseta* Curran, 1927f: 105. Holotype female (BMNH). Type locality: South Africa, KwaZulu-Natal, Weenen [ca. 28°51′S 30°4′E].

***varivittata*** Curran, 1927.—Afrotropical: Cameroon, Kenya, Tanzania.

*Dexia varivittata* Curran, 1927f: 106. Holotype male (SDEI). Type locality: Cameroon (not Nigeria as published, Crosskey 1980b: 832), Buea [ca. 4°10′N 9°14′E].

##### Genus *DINERA* Robineau-Desvoidy, 1830

***DINERA*** Robineau-Desvoidy, 1830: 307. Type species: *Dinera grisea* Robineau-Desvoidy, 1830 (= *Musca carinifrons* Fallén, 1817), by subsequent designation of Townsend (1916b: 6) [Palaearctic].

*PHOROSTOMA* Robineau-Desvoidy, 1830: 326. Type species: *Phorostoma subrotunda* Robineau-Desvoidy, 1830 (= *Musca ferina* Fallén, 1817), by monotypy [Palaearctic].

*MYOCERA* Robineau-Desvoidy, 1830: 328. Type species: *Myocera longipes* Robineau-Desvoidy, 1830 (= *Musca ferina* Fallén, 1817), by subsequent designation of Townsend (1916b: 8) [Palaearctic].

*MYIOCERA* Rondani, 1868b: 597. Unjustified emendation of *Myocera* Robineau-Desvoidy, 1830 (see O’Hara et al. 2011: 123).

*MYOCEROPS* Townsend, 1916c: 178. Type species: *Musca carinifrons* Fallén, 1816, by original designation [Palaearctic].

*AFRICODEXIA* Townsend, 1933: 462. Type species: *Dexia lugens* Wiedemann, 1830, by original designation.

Note: We have not determined who, as the First Reviser (Article 24.2.2 of the *Code*, ICZN 1999), established the relative priority of *Dinera* Robineau-Desvoidy, 1830, *Phorostoma* Robineau-Desvoidy, 1830 and *Myocera* Robineau-Desvoidy, 1830 when the three are treated as synonyms.

***femoralis*** (van Emden, 1947).—Afrotropical: Ethiopia, Kenya.

*Paraprosena femoralis* van Emden, 1947: 659. Holotype male (BMNH). Type locality: Kenya, Lake Naivasha [as “Lake Naivasha, Masai Reserve”], 6000ft.

***fulvotestacea*** (Villeneuve, 1943).—Afrotropical: South Africa.

*Myiocera fulvotestacea* Villeneuve, 1943b: 95 (as “*fulvo-testacea*”). Holotype male (not located). Type locality: South Africa, KwaZulu-Natal, Durban.

***latigena*** (van Emden, 1947).—Afrotropical: Malawi.

*Paraprosena latigena* van Emden, 1947: 663. Holotype male (BMNH). Type locality: Malawi, plateau on Mt. Mulanje [as “Mlanje Mt.”], 6000–7000ft.

***lugens*** (Wiedemann, 1830).—Afrotropical: Kenya, South Africa, Zimbabwe.

*Dexia lugens* Wiedemann, 1830: 374. Type(s), male (not located). Type locality: South Africa, Western Cape, Cape of Good Hope [as “Kap”].

***palliventris*** (van Emden, 1947).—Afrotropical: Kenya, Uganda.

*Paraprosena palliventris* van Emden, 1947: 661. Holotype male (BMNH). Type locality: Uganda, Rwenzori Range [as “Ruwenzori”], Kilembe, 4500ft.

***spinosa*** (Walker, 1858).—Afrotropical: South Africa.

*Dexia spinosa* Walker, 1858: 204. Type(s), male (BMNH). Type locality: South Africa, KwaZulu-Natal, Durban [as “Port Natal”].

***suffulva*** (Villeneuve, 1943).—Afrotropical: D.R. Congo, Zimbabwe.

*Myiocera suffulva* Villeneuve, 1943b: 96. Syntypes, 3 males (1 male in CNC). Type localities: D.R. Congo (Sud-Kivu, Kalembelembe to Baraka) and Zimbabwe (Hurungwe [as “Urungwe”], Gota Gota).

##### Genus *ESTHERIA* Robineau-Desvoidy, 1830

***ESTHERIA*** Robineau-Desvoidy, 1830: 305. Type species: *Estheria imperatoriae* Robineau-Desvoidy, 1830 (= *Dexia cristata* Meigen, 1826), by subsequent designation of Townsend (1916a: 7) [Palaearctic].

*DEXIMORPHA* Rondani, 1856: 84. Type species: *Deximorpha marittima* Rondani, 1856 (as “*Dexia marittima* Macq:”) (= *Dexia picta* Meigen, 1826), by original designation (see O’Hara et al. 2011: 72) [Palaearctic].

*DOLICHODEXIA* Brauer & Bergenstamm, 1889: 118 [also 1890: 50]. Type species: *Dolichodexia rufipes* Brauer & Bergenstamm, 1889 (= *Dinera pallicornis* Loew, 1873), by original designation [Palaearctic].

*DEXIOMERA* Curran, 1933: 164. Type species: *Dexiomera surda* Curran, 1933, by original designation. **Syn. n.**

***buccata*** (van Emden, 1947).—Afrotropical: Mozambique. **Comb. n.**

*Dexia buccata* van Emden, 1947: 633. Holotype female (BMNH). Type locality: Mozambique, Maputo [as “Lorenzo Marques”].

Note: Crosskey (1984: 240) left *Dexia buccata* van Emden, 1947 unplaced, noting “Species of uncertain generic affiliation but not a *Dexia*”. This species is moved here to *Estheria* Robineau-Desvoidy, 1830.

*capensis* (Brauer & Bergenstamm, 1891).

*Deximorpha capensis* Brauer & Bergenstamm, 1891: 417 [also 1891: 113] (as “*capensis* S. litt. Cap. [Cape of Good Hope]”). *Nomen nudum*.

Note: Although *Deximorpha capensis* Brauer & Bergenstamm, 1891 is an unavailable name, there are seven specimens labelled as *capensis* from “Cap” [= Cape of Good Hope] and “Coll. Winthem” in NHMW (examined by JEOH). Based on these specimens, *Deximorpha capensis* is moved here from Crosskey’s (1980b: 835) “Unplaced species and names of Dexiini”. This change is not treated as a new combination because *Deximorpha capensis* is an unavailable name.

***notopleuralis*** (van Emden, 1947).—Afrotropical: South Africa.

*Dexiomera notopleuralis* van Emden, 1947: 639. Holotype male (BMNH). Type locality: South Africa, KwaZulu-Natal, Willow Grange.

***surda*** (Curran, 1933).—Afrotropical: South Africa. **Comb. n.**

*Dexiomera surda* Curran, 1933: 165. Holotype male (formerly in ZMUH but destroyed according to Crosskey 1984: 239). Type locality: South Africa, Eastern Cape, Algoa Bay.

Note: *Dexiomera surda* Curran, 1933 is the type species of *Dexiomera* Curran, 1933. Crosskey (1980b: 832) treated both genus and species names as valid, but the species (and hence the genus) is moved here to *Estheria* Robineau-Desvoidy, 1830.

***turneri*** (van Emden, 1947).—Afrotropical: South Africa.

*Dexiomera turneri* van Emden, 1947: 638. Holotype male (BMNH). Type locality: South Africa, Eastern Cape, Somerset East.

##### Genus *EUPODODEXIA* Villeneuve, 1915

***EUPODODEXIA*** Villeneuve, 1915b: 200. Type species: *Eupododexia festiva* Villeneuve, 1915, by subsequent designation of Townsend (1936a: 140).

*HOMOTRIXODES* Townsend, 1926b: 529. Type species: *Eupododexia diaphana* Villeneuve, 1915, by original designation.

***amoena*** Mesnil, 1976.—Afrotropical: Madagascar.

*Eupododexia amoena* Mesnil, 1976: 42. Holotype male (NHMB [“to be returned to MNHN”, O’Hara 1996: 132]). Type locality: Madagascar, Antananarivo, Ambatolampy [ca. 19°23′S 47°26′E].

***diaphana*** Villeneuve, 1915.—Afrotropical: Madagascar.

*Eupododexia diaphana* Villeneuve, 1915b: 202. Holotype male (CNC). Type locality: Madagascar, Antananarivo, Antananarivo [as “Tananarive”].

***festiva*** Villeneuve, 1915.—Afrotropical: Madagascar.

*Eupododexia festiva* Villeneuve, 1915b: 201. Lectotype male (NHMW), by fixation of Townsend (1938b: 335) (mention of “Ht male” from Andrangoloaka in NHMW is regarded as a lectotype fixation). Type locality: Madagascar, Antananarivo, Andrangoloaka [ca. 19°2′S 47°55′E].

***gigantea*** Mesnil, 1976.—Afrotropical: Madagascar.

*Eupododexia gigantea* Mesnil, 1976: 41. Holotype male (IRSNB). Type locality: Madagascar, “Ahitsitondrona” [not located].

***picta*** Mesnil, 1976.—Afrotropical: Madagascar.

*Eupododexia picta* Mesnil, 1976: 40. Holotype female (MNHN). Type locality: Madagascar, “Ambalamalakana” [not located].

##### Genus *FRONTODEXIA* Mesnil, 1976

***FRONTODEXIA*** Mesnil, 1976: 51. Type species: *Frontodexia lutea* Mesnil, 1976, by original designation.

***lutea*** Mesnil, 1976.—Afrotropical: Madagascar.

*Frontodexia lutea* Mesnil, 1976: 51. Holotype male (MNHN). Type locality: Madagascar, Fianarantsoa, Vohiparara [within Parc National de Ranomafana, which is located at ca. 21°13′S 47°26′E].

##### Genus *MESNILOTRIX* Cerretti & O’Hara, gen. n.

***MESNILOTRIX*** Cerretti & O’Hara, **gen. n.** Type species: *Dexiotrix empiformis* Mesnil, 1976, by present designation.

Note: This new genus is described in the New Taxa of Afrotropical Tachinidae section.

***empiformis*** (Mesnil, 1976).—Afrotropical: Madagascar. **Comb. n.**

*Dexiotrix empiformis* Mesnil, 1976: 48. Holotype male (MNHN). Type locality: Madagascar, Antananarivo, Ambohitantely [Réserve Spéciale, ca. 18°10′S 47°17′E], 1600m.

Note: *Dexiotrix empiformis* Mesnil, 1976 was treated in the genus *Dexiotrix* Villeneuve, 1936 by Crosskey (1980b: 832). It was later reassigned to *Trixa* Meigen, 1824 when *Dexiotrix* was synonymized with *Trixa* by Zhang and Shima (2005: 59). This species is moved here to *Mesnilotrix* gen. n. and is redescribed in the New Taxa of Afrotropical Tachinidae section. *Dexiotrix* was no longer recorded from the Afrotropical Region as a result of the taxonomic change of Zhang and Shima (2005) and *Trixa* is similarly no longer recorded from the region as a result of the reassignment here of *Dexiotrix empiformis* to *Mesnilotrix*.

##### Genus *PILIGENA* van Emden, 1947

***PILIGENA*** van Emden, 1947: 666. Type species: *Piligena mackieae* van Emden, 1947, by monotypy.

***mackieae*** van Emden, 1947.—Afrotropical: South Africa, Zimbabwe (**new record**, CNC).

*Piligena mackieae* van Emden, 1947: 667. Holotype male (BMNH). Type locality: South Africa, Western Cape, Bot River.

Undescribed sp.: South Africa (Limpopo Province) (MZUR, examined by PC).

##### Genus *PILIGENOIDES* Barraclough, 1985

***PILIGENOIDES*** Barraclough, 1985b: 268. Type species: *Piligenoides vittata* Barraclough, 1985, by original designation.

***vittata*** Barraclough, 1985.—Afrotropical: South Africa.

*Piligenoides vittata* Barraclough, 1985b: 269. Holotype male (NMDA). Type locality: South Africa, KwaZulu-Natal, St Lucia Nature Reserve.

##### Genus *PLATYDEXIA* van Emden, 1954

***PLATYDEXIA*** van Emden, 1954: 550. Type species: *Platydexia maynei* van Emden, 1954, by original designation.

***maynei*** van Emden, 1954.—Afrotropical: D.R. Congo.

*Platydexia maynei* van Emden, 1954: 551 (as “*maynéi*”). Holotype male (MRAC). Type locality: D.R. Congo, Sud-Kivu, Kalembelembe to Baraka.

##### Genus *PODODEXIA* Brauer & Bergenstamm, 1889

***PODODEXIA*** Brauer & Bergenstamm, 1889: 117 [also 1890: 49]. Type species: *Pododexia arachna* Brauer & Bergenstamm, 1889, by monotypy.

***arachna*** Brauer & Bergenstamm, 1889.—Afrotropical: Madagascar.

*Pododexia arachna* Brauer & Bergenstamm, 1889: 117, 166 [also 1890: 49, 98]. Type(s), published as male (7 males and 4 females in NHMW). Type locality: Madagascar.

Note: *Pododexia arachna* Brauer & Bergenstamm, 1889 was described from an unspecified number of males from Madagascar. There are seven males and four females in NHMW, most collected by Sikora (or “Sicora”) and two from the locality of Andrangoloaka in Antananarivo Province [ca. 19°2′S 47°55′E], and most identified as *arachna* by “B. B.” (examined by JEOH). Although the female sex was not mentioned in the original description it seems likely that the four females recorded here were part of the original series of specimens examined by Brauer and Bergenstamm.

***hirtipleura*** Mesnil, 1976.—Afrotropical: Madagascar.

*Pododexia hirtipleura* Mesnil, 1976: 39. Holotype male (MNHN). Type locality: Madagascar, Antananarivo, Ambatolampy [ca. 19°23′S 47°26′E], “Andranotobaka” [not located], 1400m.

***similis*** Mesnil, 1976.—Afrotropical: Madagascar.

*Pododexia similis* Mesnil, 1976: 39. Holotype male (MNHN). Type locality: Madagascar, Antananarivo, Ambatolampy [ca. 19°23′S 47°26′E], “Andranotobaka” [not located], 1400m.

##### Genus *PRETORIAMYIA* Curran, 1927

***PRETORIAMYIA*** Curran, 1927f: 106. Type species: *Pretoriamyia munroi* Curran, 1927, by original designation.

***anacrostichalis*** van Emden, 1947.—Afrotropical: Kenya.

*Pretoriamyia anacrostichalis* van Emden, 1947: 653. Holotype female (BMNH). Type locality: Kenya, Mt. Elgon, 8500ft.

***munroi*** Curran, 1927.—Afrotropical: D.R. Congo (**new record**, IRSNB [PC]), Kenya (**new record**, MZUR [PC]), South Africa, Tanzania, Yemen.

*Pretoriamyia munroi* Curran, 1927f: 107. Holotype male (SANC). Type locality: South Africa, Gauteng, Pretoria.

***ogilviei*** van Emden, 1947.—Afrotropical: South Africa.

*Pretoriamyia ogilviei* van Emden, 1947: 650. Holotype male (BMNH). Type locality: South Africa, Free State, Norvalspont [as “Norvals Pont”], “North Bank Halt” [not located but presumably north of the Orange River in Free State, across the river from Norvalspont in Northern Cape].

***plumicornis*** van Emden, 1947.—Afrotropical: South Africa.

*Pretoriamyia plumicornis* van Emden, 1947: 651. Holotype female (BMNH). Type locality: South Africa, Eastern Cape, Graaf-Reinet.

***sellifera*** van Emden, 1947.—Afrotropical: South Africa.

*Pretoriamyia sellifera* van Emden, 1947: 652. Holotype female (BMNH). Type locality: South Africa, Western Cape, Doring [as “Doorn”] River.

***somereni*** van Emden, 1947.—Afrotropical: D.R. Congo (**new record**, IRSNB [PC]), Uganda.

*Pretoriamyia somereni* van Emden, 1947: 655. Holotype female (BMNH). Type locality: Uganda, Semliki National Park [as “Bwamba Valley”, ca. 0°49′N 30°3′E].

##### Genus *PROSENA* Lepeletier & Serville, 1828

*CALIRRHOE* Meigen, 1800: 39. Name suppressed by ICZN (1963: 339).

***PROSENA*** Lepeletier & Serville *in* Latreille et al., 1828: 499, 500. Type species: *Stomoxys siberita* Fabricius, 1775, by original designation.

***siberita*** (Fabricius, 1775).—Afrotropical: Mozambique. Palaearctic: C. Asia, Europe (all except Turkey), Japan, Mongolia, Pal. China, Russia (W. Russia, W. Siberia, E. Siberia, S. Far East), Transcaucasia. Oriental: India, Indonesia, Malaysia, Myanmar, Nepal, Philippines, Ryukyu Is., Sri Lanka, Taiwan. Australasian: Australia, ?Melanesia. Nearctic: introduced and established in United States.

*Stomoxys siberita* Fabricius, 1775: 798. Type(s), unspecified sex (ZMUC, destroyed and only name label remaining according to Zimsen 1964: 485; originally in ZMUK). Type locality: Denmark, Copenhagen [as “Havniae”].

*Stomoxys flavipennis* Wiedemann, 1819: 20. Lectotype male (ZMUC), by designation of Crosskey (1966a: 668). Type locality: Indonesia, Jawa.

*Prosena longirostris* Egger, 1860: 798. Syntypes, males and females (NHMW). Type locality: Austria, including Mödling near Wien.

*Prosena sybarita* Rondani, 1861a: 280. Unjustified emendation of *Stomoxys siberita* Fabricius, 1775.

*Calirrhoe malayana* Townsend, 1926c: 25. Lectotype male (RMNH), by designation of Crosskey (1969: 91). Type locality: Indonesia, Sumatera, Bukittinggi [as “Fort de Kock”] 920m.

*Prosena brevirostris* van Emden, 1947: 630. Holotype male (BMNH). Type locality: Mozambique, Maputo [as “Lorenzo Marques”].

*sibirita*. Incorrect subsequent spelling of *siberita* Fabricius, 1775 (e.g., Aldrich 1928: 130).

Note: Herting (1984: 143) reported the sex of the type(s) of *Stomoxys siberita* Fabricius, 1775 as male, but on what basis is unknown.

There are likely syntypes of *Prosena longirostris* Egger, 1860 among the specimens of *Prosena siberita* (Fabricius) in NHMW (examined by JEOH) but they are not labelled as types and are not easily recognized. Specimens identified by Egger from Austria are labelled as *siberita*. The only specimens from Austria labelled as *longirostris* from “Coll. Egger” were identified by Schiner. No specimen is labelled as collected from Mödling (cf. Herting 1974b: 131).

Wiedemann (1819: 20) gave the sex of the type(s) of *Stomoxys flavipennis* as female, but Crosskey (1966a: 668) found only two males in ZMUC and designated one of them as lectotype. A female in NHMW labelled as *flavipennis* from “Java” and “Coll. Winthem” is possibly a paralectotype.

##### Genus *PROSENOIDES* Brauer & Bergenstamm, 1891

***PROSENOIDES*** Brauer & Bergenstamm, 1891: 370 [also 1891: 66]. Type species: *Prosenoides papilio* Brauer & Bergenstamm, 1891 (as “*Prosena papilio* S. litt.”) (= *Prosena curvirostris* Bigot, 1889), by monotypy [Neotropical].

*NEOPROSENA* Townsend, 1927a: 221. Type species: *Neoprosena haustellata* Townsend, 1927, by original designation [Neotropical].

*PERIPROSENA* Villeneuve, 1938c: 14. Type species: *Periprosena dispar* Villeneuve, 1938, by monotypy.

***cytorus*** (Walker, 1849).—Afrotropical: South Africa, “West Africa”.

*Stomyxys cytorus* Walker, 1849: 1160 (as “Stomyxys? cytorus”, with “*Stomyxys*” as an error for *Stomoxys*). Type(s), unspecified sex (1 male in BMNH according to BMNH database). Type locality: “West Africa”.

***dispar*** (Villeneuve, 1938).—Afrotropical: D.R. Congo.

*Periprosena dispar* Villeneuve, 1938c: 14. Holotype female (CNC). Type locality: D.R. Congo, Nord-Kivu, Mokoto [ca. 1°15′S 29°00′E].

***longilingua*** (Villeneuve, 1943).—Afrotropical: D.R. Congo.

*Myiocera longilingua* Villeneuve, 1943b: 95. Holotype male (not located). Type locality: D.R. Congo, Nord-Kivu, Kibati [ca. 1°36′S 29°16′E].

Note: Villeneuve (1943b: 95) gave the type locality of *Myiocera longilingua* as “Kibati”. Van Emden (1947: 665) was unsure of the location of Kibati and wrote “‘Kibati’ (?Uganda: Kibate River)”. Crosskey (1980b: 834) placed the locality in Tanzania. Villeneuve (1938a: 5) cited “N. Kivu, Kibati” for *Wagneria fratella* Villeneuve and this locality, in Nord-Kivu of D.R. Congo, is assumed to be the same Kibati as cited for the type locality of *Myiocera longilingua*.

***tenuipes*** (van Emden, 1947).—Afrotropical: Uganda.

*Paraprosena tenuipes* van Emden, 1947: 665. Holotype male (BMNH). Type locality: Uganda, Rwenzori Range [as “Ruwenzori”], Namwamba Valley, 6500ft.

##### Genus *PSEUDODINERA* Brauer & Bergenstamm, 1891

***PSEUDODINERA*** Brauer & Bergenstamm, 1891: 378 [also 1891: 74]. Type species: *Pseudodinera nigripes* Brauer & Bergenstamm, 1891, by monotypy.

***nigripes*** Brauer & Bergenstamm, 1891.—Afrotropical: South Africa.

*Pseudodinera nigripes* Brauer & Bergenstamm, 1891: 379 [also 1891: 75] (as “*nigripes* Wd. Coll. Winth. litt.”). Type(s), male (2 males in NHMW). Type locality: South Africa, Western Cape, Cape of Good Hope [as “Cap b. sp.” = “Cap Bonae Spei”].

Note: *Pseudodinera nigripes* Brauer & Bergenstamm, 1891 was described from an unspecified number of males. There are two male syntypes in NHMW, both from “Cap.” [= Cape of Good Hope] and “Coll. Winthem” (examined by JEOH). Townsend (1938b: 369) mentioned “Ht male” from Cape of Good Hope in NHMW but did not restrict the term “Ht” to a single male among the two males in NHMW, and hence did not fix a lectotype.

***spinigera*** (Thomson, 1869).—Afrotropical: South Africa.

*Dinera spinigera* Thomson, 1869: 531. Type(s), male (NHRS). Type locality: South Africa, Western Cape, Cape of Good Hope [as “Promont. bonae spei”].

##### Genus *ZELIOMIMA* Mesnil, 1976

***ZELIOMIMA*** Mesnil, 1976: 37. Type species: *Zeliomima caudata* Mesnil, 1976, by original designation.

***caudata*** Mesnil, 1976.—Afrotropical: Madagascar.

*Zeliomima caudata* Mesnil, 1976: 39. Holotype male (MNHN). Type locality: Madagascar, Toamasina, Périnet, 1000m [ca. 18°55′S 48°25′E].

***chaetosa*** Mesnil, 1976.—Afrotropical: Madagascar.

*Zeliomima chaetosa* Mesnil, 1976: 39. Holotype male (MNHN). Type locality: Madagascar, Mahajanga, Antsalova [District], Forêt Antsingy, Andobo, 190m [not located but likely within Réserve naturelle intégrale du Tsingy de Bemaraha].

##### Genus *ZEUXIOTRIX* Mesnil, 1976

***ZEUXIOTRIX*** Mesnil, 1976: 46. Type species: *Zeuxiotrix atra* Mesnil, 1976, by original designation.

***atra*** Mesnil, 1976.—Afrotropical: Madagascar.

*Zeuxiotrix atra* Mesnil, 1976: 48. Holotype male (MNHN). Type locality: Madagascar, Antananarivo, Ambohitantely [Réserve Spéciale, ca. 18°10′S 47°17′E].

***cinerosa*** Mesnil, 1976.—Afrotropical: Madagascar.

*Zeuxiotrix cinerosa* Mesnil, 1976: 47. Holotype male (MNHN). Type locality: Madagascar, Antananarivo, Ambohitantely [Réserve Spéciale, ca. 18°10′S 47°17′E].

##### Unplaced species of Dexiini

***brunnicornis*** Macquart, 1844.—Afrotropical: Réunion.

*Dexia brunnicornis* Macquart, 1844: 86 [also 1844: 243]. Lectotype male (MNHN), by fixation of Crosskey (1971: 265) (examination of “Holotype ♂” from Réunion in MNHN is regarded as a lectotype fixation). Type locality: Réunion [as “Bourbon”].

***crassipalpis*** Mesnil, 1950.—Afrotropical: Zimbabwe.

*Dinera crassipalpis* Mesnil, 1950d: 115. Syntypes, 3 females (not located). Type locality: Zimbabwe, Hurungwe [as “Urungwe”], Gota Gota.

#### Tribe DUFOURIINI

##### Genus *CHETOPTILIA* Rondani, 1862

***CHETOPTILIA*** Rondani, 1862: 166. Type species: *Ptilops puella* Rondani, 1862, by monotypy [Palaearctic].

*CHAETOPTILIA* Bezzi & Stein, 1907: 402. Unjustified emendation of *Chetoptilia* Rondani, 1862 (see O’Hara et al. 2011: 55, 259).

*PARAPTILOPS* Mesnil, 1975a: 1358. Type species: *Chaetoptilia angustifrons* Mesnil, 1953, by original designation [Oriental].

***cyanea*** Mesnil, 1968.—Afrotropical: Madagascar.

*Chaetoptilia cyanea* Mesnil, 1968a: 53. Holotype male (BMNH). Type locality: Madagascar, Toamasina, Toamasina [as “Tamatave”].

***metallica*** Mesnil, 1968.—Afrotropical: Madagascar.

*Chaetoptilia metallica* Mesnil, 1968a: 54. Holotype male (MNHN). Type locality: Madagascar, Toliara, Morondava [District], forest south of Befasy [ca. 20°35′S 44°22′E].

***plumicornis*** Villeneuve, 1942.—Afrotropical: Uganda.

*Chaetoptilia plumicornis* Villeneuve, 1942a: 53. Holotype male (not located). Type locality: Uganda, Kampala.

##### Genus *MESNILANA* van Emden, 1945

***MESNILANA*** van Emden, 1945: 413. Type species: *Mesnilana bevisi* van Emden, 1945, by monotypy.

Note: We follow van Emden (1945: 413) and Crosskey (1980b: 829) in placing *Mesnilana* van Emden, 1945 in Dufouriini but we are uncertain whether this genus belongs here.

***bevisi*** van Emden, 1945.—Afrotropical: South Africa.

*Mesnilana bevisi* van Emden, 1945: 414. Holotype female (BMNH). Type locality: South Africa, KwaZulu-Natal, Greenwood Park [suburb of Durban].

##### Genus *PANDELLEIA* Villeneuve, 1907

***PANDELLEIA*** Villeneuve, 1907: 392. Type species: *Etheria sexpunctata* Pandellé, 1896, by monotypy [Palaearctic].

*AFROPHASIA* Curran, 1939: 1. Type species: *Afrophasia dimorphia* Curran, 1939, by original designation.

***dimorphia*** (Curran, 1939).—Afrotropical: Burundi, D.R. Congo, Kenya, Lesotho, South Africa, Tanzania, Uganda.

*Afrophasia dimorphia* Curran, 1939: 1. Holotype male (SANC). Type locality: South Africa, Eastern Cape, East London.

*Pandelleia francoisi* Mesnil, 1952a: 2 (as “*françoisi*”). Holotype male (IRSNB). Type locality: Burundi, Bururi, 1950m.

***translucens*** (Mesnil, 1959).—Afrotropical: Tanzania.

*Rondania translucens* Mesnil, 1959: 27. Holotype female (SMNS). Type locality: Tanzania, Pare Mountains, Usangi.

##### Genus *RHINOPHOROIDES* Barraclough, 2005

***RHINOPHOROIDES*** Barraclough, 2005: 381. Type species: *Rhinophoroides minutus* Barraclough, 2005, by original designation.

Note: *Rhinophoroides* Barraclough, 2005 is possibly a junior synonym of *Mesnilana* van Emden, 1945.

***minutus*** Barraclough, 2005.—Afrotropical: South Africa.

*Rhinophoroides minutus* Barraclough, 2005: 382. Holotype male (NMDA). Type locality: South Africa, KwaZulu-Natal, Merrivale, Tshwalabenyoni, 1000m (29°31′S 20°15′E).

#### Tribe EUTHERINI

##### Genus *EUTHERA* Loew, 1866

***EUTHERA*** Loew, 1866: 46, 47. Type species: *Euthera tentatrix* Loew, 1866, by monotypy [Nearctic].

*EUTHEROPSIS* Townsend, 1916c: 178. Type species: *Euthera mannii* Mik, 1889 (= *Ocyptera fascipennis* Loew, 1854), by original designation.

*PREUTHERA* Townsend, 1933: 452. Type species: Euthera (Eutheropsis) peringueyi Bezzi, 1925, by original designation [Oriental].

***fascipennis*** (Loew, 1854).—Afrotropical: Malawi, Tanzania, Yemen. Palaearctic: C. Asia, Europe (SW. Eur., SC. Eur., SE. Eur., Turkey). Oriental: India, Taiwan.

*Ocyptera fascipennis* Loew, 1854: 20. Type(s), male (1 male in ZMHB). Type locality: Greece, Crete [or Kriti], Heraklion [as “Candia”].

*Euthera mannii* Mik, 1889: 132. Lectotype female (NHMW), by fixation of Townsend (1931: 391) (examination of “Female Ht” from “Brussa” in NHMW is regarded as a lectotype fixation). Type locality: Turkey, Bursa [as “Brussa”].

*Euthera burtti* van Emden, 1960: 383. Holotype male (BMNH). Type locality: Tanzania, Old Shinyanga.

*manni*. Incorrect subsequent spelling of *mannii* Mik, 1889 (e.g., Herting 1984: 162, Zeegers 2007: 404).

*peringueyi* Bezzi, 1925.—Not Afrotropical [Oriental].

Euthera (Eutheropsis) peringueyi Bezzi, 1925a: 280 (as “*péringueyi*”).

Note: Bezzi (1925a: 280) was in error in citing the type locality of his new species *Euthera peringueyi* as “Chabra, Congo”. Arnaud (1982: 13) noted that the holotype in MSNM is labelled “Chapra/Mackenzie” and commented: “Bezzi stated the type was from the ‘Congo,’ but could this be in error for India?” We have determined that this is indeed the case, as Mackenzie collected in Chapra in West Bengal, India, not in the African “Congo” [e.g., Distant (1912) and Banks (1913)]. Van Emden (1960: 383) treated *Euthera peringueyi* as a species from “Congo”, Crosskey (1976: 175) recorded it from “‘Congo’ [? Zaire]: Chabra” and India, and Crosskey (1980b: 829) recorded it from “‘Congo’” and India. It is, based on present evidence, a strictly Oriental species.

***tuckeri*** Bezzi, 1925.—Afrotropical: Botswana (**new record**, NMDA [PC]), Ghana, Kenya (**new record**, MZUR [PC]), Malawi, Mozambique (**new record**, MZUR [PC]), South Africa, Sudan, U.A. Emirates, Uganda, Zambia (**new record**, NMDA [PC]). Palaearctic: Japan. Oriental: Pakistan [also questionably from Sri Lanka according to Crosskey (1976: 175) but this country not listed by Crosskey (1980b: 829)].

Euthera (Eutheropsis) tuckeri Bezzi, 1925a: 279. Holotype male (SAMC). Type locality: South Africa, Mpumalanga, Kaapmuiden [as “Koopmuiden”, ca. 25°33′S 31°20′E].

#### Tribe VORIINI

##### Genus *ALLOTHELAIRA* Villeneuve, 1915

***ALLOTHELAIRA*** Villeneuve, 1915c: 226. Type species: *Allothelaira diaphana* Villeneuve, 1915, by monotypy.

***diaphana*** Villeneuve, 1915.—Afrotropical: Cameroon, D.R. Congo, Ghana, Nigeria, Sierra Leone, Tanzania.

*Allothelaira diaphana* Villeneuve, 1915c: 226. Lectotype male (BMNH), by designation of van Emden (1960: 377). Type locality: Ghana, Aburi.

Note: *Allothelaira diaphana* Villeneuve, 1915 was described from five males and two females, including two males from Aburi (Ghana). Townsend (1939b: 8) mentioned a “Ht” from Ghana in Rambouillet (Villeneuve’s personal collection, since dispersed) but did not restrict the term “Ht” to a single male among the two males from Ghana in the type series, and hence did not fix a lectotype.

##### Genus *CAMPYLOCHETA* Rondani, 1859

***CAMPYLOCHETA*** Rondani, 1859: 157, 169. Type species: *Tachina praecox* Meigen, 1824, by fixation of O’Hara and Wood (2004: 18) under Article 70.3.2 of the *Code* (ICZN 1999), misidentified as *Tachina schistacea* Meigen, 1824 in the original designation by Rondani (1859) [Palaearctic].

*ELPE* Robineau-Desvoidy, 1863a: 488. Type species: *Tachina inepta* Meigen, 1824, by original designation [Palaearctic].

*MYXACTIA* Villeneuve, 1915b: 197. Type species: *Myxactia inclinata* Villeneuve, 1915, by monotypy.

*CAMPYLOCHAETA* Bezzi & Stein, 1907: 305. Unjustified emendation of *Campylocheta* Rondani, 1859 (see O’Hara et al. 2011: 46, 259).

*CHAETOPHLEPSIS* Townsend, 1915b: 422. Type species: *Chaetophlepsis tarsalis* Townsend, 1915, by original designation [Neotropical].

***inclinata*** (Villeneuve, 1915).—Afrotropical: Madagascar.

*Myxactia inclinata* Villeneuve, 1915b: 197. Holotype male (NHMW). Type locality: Madagascar.

Note: Townsend (1939a: 370) gave the type locality of *Myxactia inclinata* Villeneuve, 1915 as “Sikora, Madagascar”, but Sikora was the collector. No type locality within Madagascar was given by Villeneuve (1915b) or appears on the data label of the holotype (examined by JEOH).

***keiseri*** Mesnil, 1978.—Afrotropical: Madagascar.

*Campylochaeta keiseri* Mesnil, 1978b: 284. Holotype male (MNHN). Type locality: Madagascar, Toamasina, Périnet [ca. 18°55′S 48°25′E].

***plumbea*** (Mesnil, 1952).—Afrotropical: D.R. Congo, Rwanda (**new record**, IRSNB [PC]).

*Frivaldszkia plumbea* Mesnil, 1952a: 8. Holotype male (not located). Type locality: D.R. Congo, Nord-Kivu, Bweza, Tshamugussa, 2250m [ca. 1°20′S 29°31′E].

***risbeci*** (Mesnil, 1944).—Afrotropical: Mali, Nigeria, Senegal, Uganda.

*Frivaldzkia risbeci* Mesnil, 1944: 16. Type(s), unspecified sex (MNHN). Type locality: Senegal, Bambey.

***vansomereni*** van Emden, 1960.—Afrotropical: Kenya.

*Campylochaeta vansomereni* van Emden, 1960: 352. Holotype male (BMNH). Type locality: Kenya, Meru.

##### Genus *CYRTOPHLOEBA* Rondani, 1856

***CYRTOPHLOEBA*** Rondani, 1856: 207. Type species: *Tachina ruricola* Meigen, 1824, by original designation [Palaearctic].

*CYRTHOPLAEBA* Rondani, 1857: 13. Unjustified emendation of *Cyrtophloeba* Rondani, 1856 (see O’Hara et al. 2011: 69).

*STACKELBERGULA* Richter, 1967: 478. Type species: *Stackelbergula eremophila* Richter, 1967, by original designation.

*CYRTHOPHLAEBA*. Incorrect subsequent spelling of *Cyrtophloeba* Rondani, 1856 (Rondani 1859: 235) (see O’Hara et al. 2011: 68).

*CYRTHOPHLEBA*. Incorrect subsequent spelling of *Cyrtophloeba* Rondani, 1856 (Rondani 1857: 13) (see O’Hara et al. 2011: 68).

*CYRTOPHLEBA*. Incorrect original spelling of *Cyrtophloeba* Rondani, 1856 (Rondani 1856: 68) (see O’Hara et al. 2011: 69).

***arabica*** Zeegers, 2007.—Afrotropical: Yemen.

Cyrtophleba (Stackelbergula) arabica Zeegers, 2007: 374. Holotype male (RMNH). Type locality: Yemen, Laḩij [as “Lahj”] (13°03′28″N 44°53′02″E).

***eremophila*** (Richter, 1967).—Afrotropical: U.A. Emirates. Palaearctic: C. Asia, Mongolia.

*Stackelbergula eremophila* Richter, 1967: 479. Holotype male (ZIN). Type locality: Uzbekistan, Kyzylkum [Desert], 40km east of Dzhingel’dy, Ayakguzhumdy [ca. 40°44′N 63°45′E].

Undescribed spp.: Kenya (Crosskey 1980b: 837), “two new undescribed species from tropical Africa (BMNH)” (Crosskey 1984: 245), and Mozambique (MZUR, examined by PC).

##### Genus *HYLEORUS* Aldrich, 1926

***HYLEORUS*** Aldrich, 1926a: 16. Type species: *Hyleorus furcatus* Aldrich, 1926, by monotypy [Australasian].

*STEINIOMYIA* Townsend, 1932: 54. Type species: *Plagia elata* Meigen, 1838, by monotypy [Palaearctic].

*NEUROPLAGIA* Townsend, 1933: 479. Type species: *Plagia elata nudinerva* Villeneuve, 1920, by original designation.

*AFROPLAGIA* Curran, 1938: 6. Type species: *Afroplagia fasciata* Curran, 1938, by original designation.

***fasciatus*** (Curran, 1938).—Afrotropical: Ghana, South Africa, Uganda.

*Afroplagia fasciata* Curran, 1938: 6. Holotype male (SAMC, not located by JEOH). Type locality: South Africa, KwaZulu-Natal, Wartburg.

***nudinerva*** (Villeneuve, 1920).—Afrotropical: Yemen. Palaearctic: Europe (SW. Eur.), M. East (Israel).

*Plagia elata nudinerva* Villeneuve, 1920b: 200. Holotype, unspecified sex [female, examined by PC] (IRSNB). Type locality: Spain (Sierra de Albarracín [as “Sierra Albarracin”] according to label data).

##### Genus *HYSTRICOVORIA* Townsend, 1928

***HYSTRICOVORIA*** Townsend, 1928: 395. Type species: *Hystricovoria bakeri* Townsend, 1928, by original designation.

*AFROVORIA* Curran, 1938: 5. Type species: *Afrovoria munroi* Curran, 1938 (= *Hystricovoria bakeri* Townsend, 1928), by original designation.

*ANAVORIA* Mesnil, 1953b: 170 (as subgenus of *Voria* Robineau-Desvoidy, 1830). Type species: Voria (Anavoria) indica Mesnil, 1953 (= *Hystricovoria bakeri* Townsend, 1928), by monotypy.

***bakeri*** Townsend, 1928.—Afrotropical: Botswana, Ghana, Kenya, South Africa, Yemen. Australasian: ?Australia. Oriental: India, Orien. China, Philippines.

*Hystricovoria bakeri* Townsend, 1928: 395. Holotype male (USNM). Type locality: Philippines, Luzon, Mt. Makiling [as “Mount Maquiling”].

*Afrovoria munroi* Curran, 1938: 6. Holotype male (SANC). Type locality: South Africa, Mpumalanga, Barberton.

Voria (Anavoria) indica Mesnil, 1953b: 170. Holotype female (BMNH). Type locality: India, Uttarakhand, Dehra Dun.

##### Genus *NARDIA* Cerretti, 2009

***NARDIA*** Cerretti, 2009a: 108. Type species: *Plagiomima rufolateralis* Crosskey, 1984, by original designation.

***rufolateralis*** (Crosskey, 1984).—Afrotropical: Botswana, Namibia.

*Plagiomima rufolateralis* Crosskey, 1984: 302. Holotype male (BMNH). Type locality: Botswana, South-East, Sebele [as “Bakgatla, Sebele”; 24°34′S 25°58′E according to Cerretti 2009a: 113].

***tsavo*** Cerretti, 2009.—Afrotropical: Kenya.

*Nardia tsavo* Cerretti, 2009a: 114. Holotype female (MZUR). Type locality: Kenya, Coast, Tsavo East National Park, Ndara Plains, Aruba Lodge, 444m.

##### Genus *PERISCEPSIA* Gistel, 1848

*SCOPOLIA* Robineau-Desvoidy, 1830: 268 (junior homonym of *Scopolia* Hübner, 1825). Type species: *Musca carbonaria* Panzer, 1798, by subsequent designation of Zetterstedt (1844: 1239).

***PERISCEPSIA*** Gistel, 1848: x (unnecessary *nomen novum* for *Scopolia* Robineau-Desvoidy, 1830) (see O’Hara et al. 2011: 143).

*PHORICHETA* Rondani, 1861b: 8 (*nomen novum* for *Scopolia* Robineau-Desvoidy, 1830).

*RAMONDA* Robineau-Desvoidy, 1863a: 790. Type species: *Ramonda fasciata* Robineau-Desvoidy, 1863 (= *Tachina spathulata* Fallén, 1820), by original designation [Palaearctic].

*PHORICHAETA* Brauer & Bergenstamm, 1889: 106 [also 1890: 38]. Unjustified emendation of *Phoricheta* Rondani, 1861 (see O’Hara et al. 2011: 143, 265).

*WAGNERIA* of authors (e.g., Mesnil 1950a, van Emden 1960), not Robineau-Desvoidy, 1930. Misidentification, “on current generic limits” (Crosskey 1980b: 838).

Note: Subgenera of *Periscepsia* Gistel, 1848 are not recognized here because the subgeneric placements of the Afrotropical species require more study.

***abbreviata*** (Mesnil, 1950).—Afrotropical: D.R. Congo. **Status n.**

*Wagneria rufitibia abbreviata* Mesnil, 1950a: 1. Holotype, unspecified sex [male, examined by PC] (IRSNB). Type locality: D.R. Congo, Nord-Kivu, Volcan Mikeno, near Rweru [as “Bweru”], 2400m [ca. 1°29′S 29°24′E].

Note: *Wagneria rufitibia abbreviata* Mesnil, 1950 was treated as a synonym of *Wagneria rufitibia* Villeneuve, 1938 by Crosskey (1980b: 839) but is recognized here as a distinct species based on examination of the holotype by PC.

Mesnil (1950a: 1) gave the type locality of *Wagneria rufitibia abbreviata* as “volcan Mikeno, vers Bweru, 2.400m”. The map of Parc National Albert published by de Witte (1937) shows Rweru at 2799m within D.R. Congo about 2km north of the Rwandan border (Volcan Mikeno is 2–3km further north). Without evidence to the contrary, this type locality is treated as within D.R. Congo. Crosskey (1980b: 839) gave the country as Rwanda and this was followed by O’Hara (1996: 130); these authors treated the same locality as within Rwanda for one other species and within D.R. Congo (as “Zaire”) for two others.

***amicula*** (Mesnil, 1950).—Afrotropical: D.R. Congo, South Africa.

*Wagneria amicula* Mesnil, 1950a: 1. Holotype male (MRAC). Type locality: D.R. Congo, Nord-Kivu, Kabasha Escarpment, 1500m [ca. 0°44′S 29°13′E].

***canina*** (Mesnil, 1950).—Afrotropical: D.R. Congo, Ethiopia, Rwanda, South Africa.

*Wagneria canina* Mesnil, 1950a: 2. Holotype, unspecified sex (MRAC). Type locality: Rwanda, Volcan Sabyinyo [as “Sabinyo”], Rwebeya Valley, 3000m [ca. 1°24′S 29°36′E].

***carbonaria*** (Panzer, 1798).—Afrotropical: “widespread n.-e. Afr. to sthn Afr.” (Crosskey 1980b: 839), including D.R. Congo, Kenya, Malawi, South Africa, Sudan, Yemen, Zimbabwe. Palaearctic: Europe (all), M. East (all), Pal. China, Russia (W. Russia), Transcaucasia. Oriental: India, Pakistan.

*Musca carbonaria* Panzer, 1798: 15 (and coloured figure on unnumbered facing plate). Type(s), unspecified sex [sex cannot be determined from the figure] (lost). Type locality: Austria (Thompson and Pont 1994: 58).

*Dexia nigrans* Meigen, 1826: 40. Syntypes, published as females (male(s) in MNHN, Herting 1972: 10). Type locality: not given (Europe, from “Baumhauerischen und Wiedemannischen Museum [= collections]”).

Note: *Periscepsia carbonaria* (Panzer, 1798) of current authors is likely a species complex but is treated here as a single species pending further study.

***caviceps*** (van Emden, 1960).—Afrotropical: Zimbabwe.

*Wagneria caviceps* van Emden, 1960: 336. Holotype male (BMNH). Type locality: Zimbabwe, Harare [as “Salisbury”].

***decolor*** (van Emden, 1960).—Afrotropical: Ethiopia, Kenya, South Africa, Uganda.

*Wagneria decolor* van Emden, 1960: 347. Holotype male (BMNH). Type locality: Uganda, Nyakasura [ca. 0°40′N 30°13′E].

***fratella*** (Villeneuve, 1938).—Afrotropical: D.R. Congo, Kenya, Uganda.

*Wagneria fratella* Villeneuve, 1938a: 5. Holotype, unspecified sex (MRAC). Type locality: D.R. Congo, Nord-Kivu, Kibati [ca. 1°36′S 29°16′E].

***glossinicornis*** (van Emden, 1960).—Afrotropical: Kenya, South Africa.

*Wagneria glossinicornis* van Emden, 1960: 337. Holotype male (BMNH). Type locality: Kenya, Chyulu Hills, 6000ft.

***guttipennis*** (van Emden, 1960).—Afrotropical: Kenya.

*Wagneria guttipennis* van Emden, 1960: 345. Holotype male (BMNH). Type locality: Kenya, Naivasha.

***kirbyiformis*** (van Emden, 1960).—Afrotropical: D.R. Congo.

*Wagneria kirbyiformis* van Emden, 1960: 344. Holotype male (MRAC). Type locality: D.R. Congo, Orientale, “Kibali-Ituri”, Kilo [ca. 1°48′N 30°14′E].

***lindneri*** (Mesnil, 1959).—Afrotropical: Tanzania.

*Wagneria lindneri* Mesnil, 1959: 25. Holotype male (SMNS). Type locality: Tanzania, west side of Mt. Kibo [one of the three peaks of Mt. Kilimanjaro], 3500–4500m.

***natalica*** (van Emden, 1960).—Afrotropical: Ethiopia, Kenya, South Africa.

*Wagneria natalica* van Emden, 1960: 339. Holotype male (BMNH). Type locality: South Africa, KwaZulu-Natal, “Winzinto River” [not located].

*Wagneria laniventris* van Emden, 1960: 339. Holotype male (BMNH). Type locality: Kenya, Ngong.

*Wagneria nubilipennis* van Emden, 1960: 341. Holotype female (BMNH). Type locality: Kenya, Meru.

*Wagneria z-fuscum* van Emden, 1960: 340. Holotype female (BMNH). Type locality: South Africa, KwaZulu-Natal, Weenen [ca. 28°51′S 30°4′E].

Note: Mesnil (1978b: 284–285) synonymized the four simultaneously published van Emden (1960) names, and as First Reviser selected *Wagneria natalica* van Emden, 1960 as the senior synonym (Article 24.2.2 of the *Code*, ICZN 1999). The specific epithet in *Wagneria z-fuscum* van Emden, 1960 is assumed to refer to the brown patterning in the wing of the nominal species and therefore “*z-fuscum*” does not change to “*zfuscum*” (Article 32.5.2.4.3 of the *Code*, ICZN 1999).

***nudinerva*** (Mesnil, 1950).—Afrotropical: D.R. Congo. **Status n.**

*Wagneria rufitibia nudinerva* Mesnil, 1950a: 1. Holotype, unspecified sex [female, examined by PC] (IRSNB). Type locality: D.R. Congo, Nord-Kivu, Rutshuru, 1285m.

Note: *Wagneria rufitibia nudinerva* Mesnil, 1950 was treated as a synonym of *Wagneria rufitibia* Villeneuve, 1938 by Crosskey (1980b: 839) but is recognized here as a distinct species based on examination of the holotype by PC.

***pallidipennis*** (van Emden, 1960).—Afrotropical: D.R. Congo, Kenya.

*Wagneria pallidipennis* van Emden, 1960: 349. Holotype male (BMNH). Type locality: Kenya, Naivasha.

***propleuralis*** (van Emden, 1960).—Afrotropical: South Africa, Uganda.

*Wagneria propleuralis* van Emden, 1960: 343. Holotype female (BMNH). Type locality: Uganda, Semliki National Park [as “Bwamba Valley, Ruwenzori”, ca. 0°49′N 30°3′E].

***rufitibia*** (Villeneuve, 1938).—Afrotropical: D.R. Congo, Kenya, South Africa, Tanzania, Uganda.

*Wagneria rufitibia* Villeneuve, 1938a: 4. Holotype, unspecified sex [male, see van Emden 1960: 350] (BMNH). Type locality: South Africa, KwaZulu-Natal, Wartburg.

***salti*** (van Emden, 1960).—Afrotropical: Tanzania.

*Wagneria salti* van Emden, 1960: 348. Holotype male (BMNH). Type locality: Tanzania, Mt. Kilimanjaro, Shira Plateau, 12,450ft [ca. 3°0′S 37°14′E].

***vidua*** (Mesnil, 1950).—Afrotropical: Kenya, Rwanda, Uganda.

*Wagneria vidua* Mesnil, 1950a: 3. Holotype, unspecified sex (MRAC). Type locality: Rwanda, Volcans Gahinga–Sabyinyo [latter as “Sabinyo”], “Kundhuru ya Tshuve”, 2600m [ca. 1°23′S 29°38′E].

##### Genus *PROSHELIOMYIA* Brauer & Bergenstamm, 1891

***PROSHELIOMYIA*** Brauer & Bergenstamm, 1891: 375 [also 1891: 71]. Type species: *Prosheliomyia nietneri* Brauer & Bergenstamm, 1891, by monotypy [Oriental].

###### Subgenus *THRIXIONELLUS* Mesnil, 1968

*THRIXIONELLUS* Mesnil, 1968a: 45 (as subgenus of *Prosheliomyia* Brauer & Bergenstamm, 1891). Type species: Prosheliomyia (Thrixionellus) mirabilis Mesnil, 1968, by original designation.

***mirabilis*** Mesnil, 1968.—Afrotropical: Madagascar.

Prosheliomyia (Thrixionellus) mirabilis Mesnil, 1968a: 45. Holotype male (NHMB [“to be returned to MNHN”, O’Hara 1996: 148]). Type locality: Madagascar, Antsiranana, Joffreville.

***nigricornis*** Mesnil, 1968.—Afrotropical: Madagascar.

Prosheliomyia (Thrixionellus) nigricornis Mesnil, 1968a: 47. Holotype male (NHMB [“to be returned to MNHN”, O’Hara 1996: 149]). Type locality: Madagascar, Fianarantsoa, Vohiparara [within Parc National de Ranomafana, which is located at ca. 21°13′S 47°26′E].

***pallida*** Mesnil, 1968.—Afrotropical: Madagascar.

Prosheliomyia (Thrixionellus) pallida Mesnil, 1968a: 48. Holotype male (NHMB [“to be returned to MNHN”, O’Hara 1996: 151]). Type locality: Madagascar, Antsiranana, Ambanoro [ca. 13°24′S 48°18′E].

##### Genus *REICHARDIA* Karsch, 1886

***REICHARDIA*** Karsch, 1886a: 137. Type species: *Reichardia insignis* Karsch, 1886, by monotypy.

***insignis*** Karsch, 1886.—Afrotropical: Tanzania.

*Reichardia insignis* Karsch, 1886a: 137. Type(s), unspecified sex (1 male in ZMHB). Type locality: Tanzania, east of Lake Tanganyika, “Kawende” [not located].

Undescribed sp.: Ethiopia (MZUR, examined by PC).

##### Genus *STOMINA* Robineau-Desvoidy, 1830

***STOMINA*** Robineau-Desvoidy, 1830: 411. Type species: *Stomina rubricornis* Robineau-Desvoidy, 1830 (= *Musca tachinoides* Fallén, 1817), by monotypy [Palaearctic].

Undetermined sp(p).—Afrotropical: Malawi (TAU, examined by PC), Namibia, South Africa, Yemen.

Note: Undetermined specimens of this genus were recorded from the Afrotropical Region by Mesnil (1975a: 1329, South Africa), Crosskey (1984: 255, Namibia) and Zeegers (2007: 375, Yemen). An undetermined male from Pretoria (South Africa) in NMDA was examined by PC.

##### Genus *SUBFISCHERIA* Villeneuve, 1937

***SUBFISCHERIA*** Villeneuve, 1937a: 210. Type species: *Subfischeria flavogrisea* Villeneuve, 1937, by monotypy.

***flavogrisea*** Villeneuve, 1937.—Afrotropical: Botswana, Malawi, Namibia, South Africa.

*Subfischeria flavogrisea* Villeneuve, 1937a: 211 (as “*flavo-grisea*”). Holotype female (CNC). Type locality: South Africa, “Colonie du Cap” ([former Cape Province], “Windsaxton Grigualand” according to label data, Cooper and O’Hara 1996: 73).

##### Genus *THELAIRA* Robineau-Desvoidy, 1830

***THELAIRA*** Robineau-Desvoidy, 1830: 214 (as “*Thelaïra*”). Type species: *Thelaira abdominalis* Robineau-Desvoidy, 1830 (= *Musca solivagus* Harris, 1780), by subsequent designation of Townsend (1916b: 9) [Palaearctic].

*THELAIRIA*. Incorrect subsequent spelling of *Thelaira* Robineau-Desvoidy, 1830 (Coquillett 1910: 614).

***altoplani*** Speiser, 1914.—Afrotropical: Angola, Cameroon, D.R. Congo, Eritrea, Ghana, Lesotho, Madagascar, Malawi, Mozambique, Nigeria, Sierra Leone, South Africa, Sudan, Tanzania, Uganda, Zimbabwe.

*Thelaira altoplani* Speiser, 1914: 12. Holotype male (not located). Type locality: Cameroon, Dschang.

*Thelaira palliventris* Curran, 1928b: 378. Holotype male (AMNH). Type locality: D.R. Congo, Orientale, Parc National de la Garamba [as “Garamba, Congo”; coordinates on label given as 29°40′E 40°10′N, by Arnaud (1963: 130)].

*Musca nigripes* of authors (e.g., Bezzi 1908b: 61, Villeneuve 1913c: 37, both as “*Thelaira nigripes*”), not Fabricius, 1794. Misidentification (Crosskey 1980b: 840).

***aurofasciata*** van Emden, 1960.—Afrotropical: Ghana, Nigeria.

*Thelaira aurofasciata* van Emden, 1960: 374. Holotype male (BMNH). Type locality: Ghana, Obuasi, Ashanti.

***luteiventris*** van Emden, 1960.—Afrotropical: Nigeria, Sudan.

*Thelaira luteiventris* van Emden, 1960: 376. Holotype male (BMNH). Type locality: Nigeria, Azare.

***madecassa*** Mesnil, 1978.—Afrotropical: Madagascar.

*Thelaira madecassa* Mesnil, 1978b: 285. Holotype male (MNHN). Type locality: Madagascar, Antananarivo, Antananarivo [as “Tananarive”].

##### Genus *VORIA* Robineau-Desvoidy, 1830

***VORIA*** Robineau-Desvoidy, 1830: 195. Type species: *Voria latifrons* Robineau-Desvoidy, 1830 (= *Tachina ruralis* Fallén, 1810), by monotypy [Palaearctic].

*PLAGIA* Meigen, 1838: 201. Type species: *Tachina verticalis* Meigen, 1824 (= *Tachina ruralis* Fallén, 1810), by subsequent designation of Rondani (1856: 69) [Palaearctic].

***capensis*** Villeneuve, 1935.—Afrotropical: Ghana, Kenya, Mozambique (**new record**, MZUR [PC]), Nigeria, South Africa.

*Plagia setosa* Brauer & Bergenstamm, 1891: 409, 439 [also 1891: 105, 135] (as “*setosa* Wd. litt. Cap. [Cape of Good Hope]”). *Nomen nudum*.

*Voria capensis* Villeneuve, 1935a: 138. Holotype male (not located). Type locality: South Africa.

***ruralis*** (Fallén, 1810).—Afrotropical: “Kenya to South Africa, South Yemen [part of present-day Yemen]” (Crosskey 1980b: 838). Palaearctic: C. Asia, Europe (all except Turkey), Japan, M. East (Israel), Mongolia, N. Africa (Madeira), Pal. China, Russia (W. Russia, W. Siberia, E. Siberia, S. Far East), Transcaucasia. Oriental: India, Nepal, Orien. China, Pakistan, Ryukyu Is., Taiwan. Australasian: Australia, N. Australasian. Nearctic: widespread. Neotropical: probably widespread.

*Tachina ruralis* Fallén, 1810: 265. Lectotype male (NHRS), by designation of Crosskey (1973b: 163). Type locality: Sweden, Skåne, Äsperöd [as “Esperöd”].

### Subfamily EXORISTINAE (Fig. 4)

**Figure 4.**
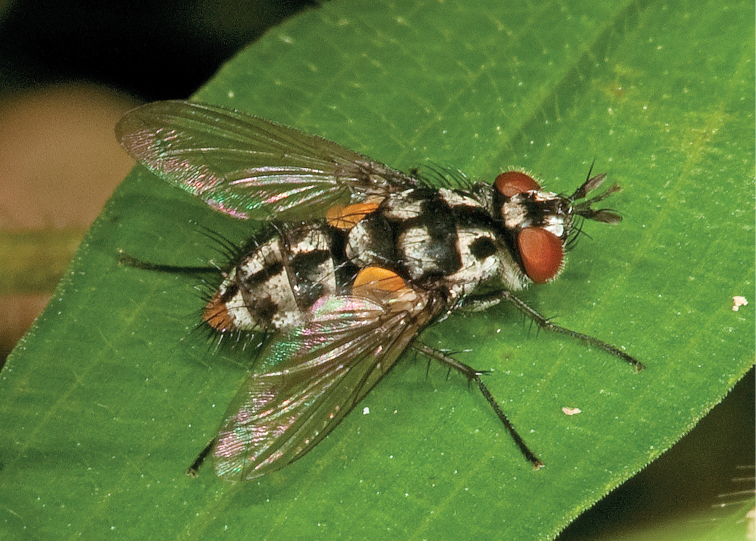
Live specimen of *Ossidingia cruciata* (Wiedemann) (Winthemiini, Exoristinae) from Magombera Forest near Mangula, Tanzania (image courtesy of S.A. Marshall).

#### Tribe ACEMYINI

##### Genus *ACEMYA* Robineau-Desvoidy, 1830

***ACEMYA*** Robineau-Desvoidy, 1830: 202. Type species: *Acemya oblonga* Robineau-Desvoidy, 1830 (= *Tachina acuticornis* Meigen, 1824), by subsequent designation of Desmarest *in* d’Orbigny (1849a: 318) (see Evenhuis and Thompson 1990: 232) [Palaearctic].

*ACOMYIA* Agassiz, 1846b: 3, 5. Unjustified emendation of *Acemya* Robineau-Desvoidy, 1830 (see Evenhuis et al. 2010: 33).

*ACEMYIA* Schiner, 1861: 472. Unjustified emendation of *Acemya* Robineau-Desvoidy, 1830 (see Evenhuis et al. 2010: 33).

***fishelsoni*** Kugler, 1968.—Afrotropical: Yemen. Palaearctic: M. East (Israel), Mongolia, Pal. China.

*Acemyia fishelsoni* Kugler, 1968: 65. Holotype female (TAU). Type locality: Israel, Metula.

***pyrrhocera*** Villeneuve, 1922.—Afrotropical: U.A. Emirates. Palaearctic: C. Asia, Europe (W. Eur., SW. Eur., SC. Eur.), Mongolia, Russia (E. Siberia), Transcaucasia.

*Acomyia pyrrhocera* Villeneuve, 1922c: 342. Syntypes, 1 male and 2 females (not located). Type localities: France, Digne-les-Bains [as “Digne”] and “sud de la France”.

##### Genus *ATLANTOMYIA* Crosskey, 1977

***ATLANTOMYIA*** Crosskey, 1977: 145. Type species: *Atlantomyia nitida* Crosskey, 1977, by original designation.

***nitida*** Crosskey, 1977.—Afrotropical: Saint Helena.

*Atlantomyia nitida* Crosskey, 1977: 147. Holotype male (MRAC). Type locality: Saint Helena, Prosperous Bay Plain, 900–1000ft.

##### Genus *CERACIA* Rondani, 1865

***CERACIA*** Rondani, 1865: 221. Type species: *Ceracia mucronifera* Rondani, 1865, by monotypy [Palaearctic].

*MYOTHYRIA* van der Wulp, 1890: 208. Type species: *Myothyria majorina* van der Wulp, 1890, by subsequent designation of Coquillett (1910: 573) [Neotropical].

*MYIOTHYRIA*. Incorrect subsequent spelling of *Myothyria* van der Wulp, 1890 (e.g., Herting 1958: 4, Mesnil 1962: 790).

Note: Herting (1984: 34) gave the type species of *Myothyria* van der Wulp, 1890 as *Myothyria majorina* van der Wulp, 1890, by subsequent designation of Brauer and Bergenstamm (1891: 358 [also 1891: 54]). Brauer and Bergenstamm (1891: 358) wrote “*Myothyria* v. d. Wp. mit der Art *majorina* v. d. Wp.”) but did not refer to *Myothyria majorina* as the type species of *Myothyria*. Crosskey (1980b: 851) correctly cited the type species as *Myothyria majorina*, by subsequent designation of Coquillett (1910: 573).

***africana*** (Mesnil, 1959).—Afrotropical: Nigeria, South Africa, Tanzania, Uganda.

*Myothyria africana* Mesnil, 1959: 19. Holotype male (SMNS). Type locality: Tanzania, Dar es Salaam.

*Ceracia burtti* van Emden, 1960: 370. Holotype female (BMNH). Type locality: Tanzania, Old Shinyanga.

***freyi*** (Herting, 1958).—Afrotropical: Cape Verde.

*Myiothyria freyi* Herting, 1958: 4. Holotype male (FMNHH). Type locality: Cape Verde Islands, São Nicolau, Ribeira da Pulga [as “S. Nicolau: Rib. Pulga”].

***mucronifera*** Rondani, 1865.—Afrotropical: Yemen. Palaearctic: C. Asia, Europe (W. Eur., SW. Eur., SC. Eur., SE. Eur.), M. East (Israel), N. Africa (Canary Is., NW. Africa), Transcaucasia. Oriental: Orien. China [Hunan].

*Ceracia mucronifera* Rondani, 1865: 222. Syntypes, 2 males (MZF). Type locality: Italy, Apennines, near Parma.

***murina*** Mesnil, 1977.—Afrotropical: Madagascar.

*Ceracia murina* Mesnil, 1977d: 326. Holotype female (MNHN). Type locality: Madagascar, Antananarivo, Antananarivo [as “Tananarive”].

##### Genus *METACEMYIA* Herting, 1969

***METACEMYIA*** Herting, 1969: 197. Type species: *Acemyia calloti* Séguy, 1936, by original designation.

*CERACIA* of Mesnil (1962: 788), not Rondani, 1865. Misidentification (Herting 1969: 196–197).

***aartseni*** Zeegers, 2007.—Afrotropical: U.A. Emirates, Yemen. Palaearctic: M. East (Israel).

*Metacemyia aartseni* Zeegers, 2007: 388. Holotype female (RMNH). Type locality: Yemen, 12km northwest of Manākhah (15°04′19″N 43°44′27″E).

***calloti*** (Séguy, 1936).—Afrotropical: Senegal, Tanzania, U.A. Emirates, Yemen, Zambia, Zimbabwe. Palaearctic: Europe (W. Eur., SW. Eur., SC. Eur., Turkey), M. East (Israel), N. Africa (NW. Africa)

*Acemyia calloti* Séguy, 1936: 324. Holotype female (not located). Type locality: Tunisia, El Aouina.

*Ceracia nomadacridis* van Emden, 1960: 369. Holotype male (BMNH). Type locality: Tanzania, Rukwa District, Nkamba-Kati.

*Ceracia mucronifera* of authors (e.g., Mesnil 1962: 789), not Rondani, 1865. Misidentification (Herting 1969: 196–197).

***setosa*** Crosskey, 1973.—Afrotropical: Malawi.

*Metacemyia setosa* Crosskey, 1973a: 376. Holotype male (BMNH). Type locality: Malawi, Southern Region, Chambe Plateau.

***uncinata*** (Thomson, 1869).—Afrotropical: Botswana, D.R. Congo, South Africa.

*Myobia uncinata* Thomson, 1869: 526. Lectotype male (NHRS), by fixation of Crosskey (1973a: 379) (examination of “holotype” from Cape of Good Hope in NHRS is regarded as a lectotype fixation). Type locality: South Africa, Western Cape, Cape of Good Hope [as “Promont. bonae spei”].

Note: A record of *Myobia uncinata* Thomson, 1869 from Israel by Kugler (1963: 26, 32, as “*Ceracia uncinata*”) was questioned by Crosskey (1973a: 380, 1980b: 851). This species was not recorded from Israel by Cerretti and Freidberg (2009).

#### Tribe ANACAMPTOMYIINI

##### Genus *ANACAMPTOMYIA* Bischof, 1904

***ANACAMPTOMYIA*** Bischof, 1904: 79. Type species: *Anacamptomyia africana* Bischof, 1904, by monotypy.

*ROUBAUDIA* Villeneuve, 1910a: 249. Type species: *Roubaudia rufescens* Villeneuve, 1910, by monotypy (not by original designation as given by Zeegers 2014: 96).

*PARAROUBAUDIA* Roubaud & Villeneuve, 1914: 122, 124 (as subgenus of *Roubaudia* Villeneuve, 1910). Type species: Roubaudia (Pararoubaudia) bisetosa Roubaud & Villeneuve, 1914, by monotypy.

Note: A key to the African species of *Anacamptomyia* Bischof, 1904 was published by Mesnil (1950b: 22–24). A key to the species of *Anacamptomyia* from Madagascar was given by Zeegers (2014: 97).

***africana*** Bischof, 1904.—Afrotropical: D.R. Congo, Kenya, ?Madagascar, Mozambique, Nigeria, Senegal, South Africa, Tanzania.

*Anacamptomyia africana* Bischof, 1904: 81. Lectotype female (NHMW), by fixation of Townsend (1940: 8) (mention of “Ht female” from Algoa Bay in NHMW is regarded as a lectotype fixation for the only syntype from Algoa Bay, a female that also bears a blue handwritten “Typus” label [examined by JEOH]). Type locality: South Africa, Eastern Cape, Algoa Bay.

***aurifrons*** Zeegers, 2014.—Afrotropical: Madagascar.

*Anacamptomyia aurifrons* Zeegers, 2014: 97. Holotype male (RMNH). Type locality: Madagascar, Antananarivo, [near] Ambatolampy, Ankaratra Mountains, Manjakatompo, 2000m [ca. 19°21′S 47°18′E].

***bisetosa*** (Roubaud & Villeneuve, 1914).—Afrotropical: Benin, Cameroon, D.R. Congo, Ghana, Nigeria, Senegal, Sierra Leone, Zimbabwe.

Roubaudia (Pararoubaudia) bisetosa Roubaud & Villeneuve, 1914: 125. Syntypes, males and females (1 female in MRAC). Type localities: Senegal (Dakar) and unspecified localities from Benin [as “Dahomey”] to Senegal.

Note: *Roubaudia bisetosa* Roubaud & Villeneuve, 1914 was described from an unspecified number of males and females. The only specific locality mentioned was Dakar (the locality where the syntype in MRAC was collected) but the range of the species was given as Senegal to Benin. Townsend (1940: 13) mentioned a “Ht male” from Accra (Ghana) in Rambouillet (Villeneuve’s personal collection, since dispersed) but a specimen from that locality has not been located. Unless a male from Accra is found, or is proven to have existed, Townsend’s “Ht male” cannot legitimately be accepted as a lectotype fixation for *Roubaudia bisetosa*.

***blommersi*** Zeegers, 2014.—Afrotropical: Madagascar.

*Anacamptomyia blommersi* Zeegers, 2014: 99. Holotype male (RMNH). Type locality: Madagascar, Antananarivo [as “Tananarive”], 1300m.

***gymnops*** Zeegers, 2007.—Afrotropical: Yemen.

*Anacamptomyia gymnops* Zeegers, 2007: 376. Holotype female (RMNH). Type locality: Yemen, Wādī Lahīmah [as “Al Lahima”] (15°24′N 43°32′E).

***obscurella*** Mesnil, 1950.—Afrotropical: “toute Afrique tropicale et australe” (Mesnil 1950b: 24, Crosskey 1980b: 867), including D.R. Congo and presumably South Africa.

*Anacamptomyia pallida obscurella* Mesnil, 1950b: 24. Syntypes, males and females (1 male and possibly other unrecognized syntypes in CNC). Type localities: Africa, “toute Afrique tropicale et australe” (CNC syntype from D.R. Congo, Équateur, Eala).

***pallida*** (Roubaud & Villeneuve, 1914).—Afrotropical: Benin, Cameroon, D.R. Congo, Ghana, Malawi, Nigeria, Senegal, Sudan, Tanzania, Zambia, Zimbabwe.

*Roubaudia rufescens pallida* Roubaud & Villeneuve, 1914: 124. Syntypes, only the male sex specifically mentioned (2 females in MRAC). Type localities: D.R. Congo, Nigeria, Senegal [including MRAC syntypes from Satadougou], and Zimbabwe.

Note: A male in CNC treated as a syntype of *Roubaudia rufescens pallida* Roubaud & Villeneuve, 1914 by Cooper and O’Hara (1996: 68) was collected from “M fongosi Zulu L.” (label data; the faded lettering was misinterpreted as “M fongoss Zulu L.” by Cooper and O’Hara 1996: 68). Mfongosi is in KwaZulu-Natal, South Africa [ca. 28°43′S 30°49′E]. South Africa was not listed as a type locality by Roubaud and Villeneuve (1914) and therefore this specimen is not considered part of the original type series.

***pruinosa*** (Roubaud & Villeneuve, 1914).—Afrotropical: Nigeria, Senegal, Uganda, Zimbabwe.

*Roubaudia pruinosa* Roubaud & Villeneuve, 1914: 123. Syntypes, male(s) and female(s) (1 female in CNC, 2 males and 1 female in MRAC). Type locality: Senegal, Satadougou [as “Satadougou (Haute-Gambie)”].

***rufescens*** (Villeneuve, 1910).—Afrotropical: Benin, Nigeria.

*Roubaudia rufescens* Villeneuve, 1910a: 249. Lectotype male (CNC), by fixation of Townsend (1940: 14) (mention of “Ht male” from Dahomey in Rambouillet [Villeneuve’s personal collection, since dispersed] is regarded as a lectotype fixation for the single male syntype in CNC). Type locality: Benin [as “Dahomey”] (country not Congo as given by Crosskey 1980b: 867).

##### Genus *LEUCOCARCELIA* Villeneuve, 1921

***LEUCOCARCELIA*** Villeneuve, 1921: 30. Type species: *Leucocarcelia argyrata* Villeneuve, 1921, by monotypy.

***argyrata*** Villeneuve, 1921.—Afrotropical: Malawi.

*Leucocarcelia argyrata* Villeneuve, 1921: 30. Holotype male (BMNH). Type locality: Malawi, Mt. Mulanje [as “Mont Mlanje”].

Undescribed spp.: D.R. Congo (MRAC, examined by PC), Nigeria (BMNH, Crosskey 1984: 276).

##### Genus *PARAPALES* Mesnil, 1950

*PARAPALES* Mesnil, 1949b: 102 (as subgenus of *Ctenophorocera* Brauer & Bergenstamm, 1891). *Nomen nudum* (proposed after 1930 without designation of type species; no included species) (see Evenhuis and O’Hara 2008: 66).

***PARAPALES*** Mesnil, 1950c: 122 (as subgenus of *Ctenophorocera* Brauer & Bergenstamm, 1891). Type species: Ctenophorocera (Parapales) pallidula Mesnil, 1950, by original designation (see Evenhuis and O’Hara 2008: 67).

***brevicornis*** Mesnil, 1977.—Afrotropical: Madagascar.

*Parapales brevicornis* Mesnil, 1977b: 192. Holotype male (MNHN). Type locality: Madagascar, Toamasina, road from Anosibe An’ Ala [as “Anosibe”] to Moramanga, 840m.

***brunnea*** Mesnil, 1977.—Afrotropical: Madagascar.

*Parapales brunnea* Mesnil, 1977b: 192. Holotype female (MNHN). Type locality: Madagascar, Antananarivo, Manjakatompo [ca. 19°21′S 47°18′E].

***luteicornis*** Mesnil, 1977.—Afrotropical: Madagascar.

*Parapales luteicornis* Mesnil, 1977b: 192. Holotype female (MNHN). Type locality: Madagascar, Toamasina, Périnet [ca. 18°55′S 48°25′E].

***micronychia*** Mesnil, 1977.—Afrotropical: Madagascar.

*Parapales micronychia* Mesnil, 1977b: 191. Holotype male (MNHN). Type locality: Madagascar, Antsiranana, Joffreville.

***pallidula*** (Mesnil, 1950).—Afrotropical: Madagascar.

Ctenophorocera (Parapales) pallidula Mesnil, 1950c: 123. Holotype male (CNC). Type locality: Madagascar, Toliara, Bekily.

***pectinipes*** Mesnil, 1977.—Afrotropical: Madagascar.

*Parapales pectinipes* Mesnil, 1977b: 192. Holotype male (MNHN). Type locality: Madagascar, Antsiranana, Nosy Bé, Fascène [ca. 13°19′S 48°19′E].

#### Tribe BLONDELIINI

##### Genus *AFROLIXA* Curran, 1939

***AFROLIXA*** Curran, 1939: 4. Type species: *Afrolixa macula* Curran, 1939, by original designation.

***macula*** Curran, 1939.—Afrotropical: Malawi, Mozambique, South Africa.

*Afrolixa macula* Curran, 1939: 4. Holotype male (SANC). Type locality: Mozambique, Maputo [as “Lourenco Marquis”].

Undescribed sp.: Côte d’Ivoire, Sudan, Uganda (BMNH, Crosskey 1984: 267).

##### Genus *ANOMALOSTOMYIA* Cerretti & Barraclough, 2007

***ANOMALOSTOMYIA*** Cerretti & Barraclough, 2007: 102. Type species: *Anomalostomyia namibica* Cerretti & Barraclough, 2007, by original designation.

Note: Cerretti and Barraclough (2007: 104) considered *Anomalostomyia* as congeneric with Crosskey’s (1984: 289) “Undetermined genus”, which was based on a single male from Angola. That specimen, originally in BMNH, cannot be located (Cerretti and Barraclough 2007). Crosskey (1984: 289) treated the genus in Eryciini (and “apparently allied to the *Erythrocera*-group of genera”) but it has been provisionally placed in Blondeliini by Cerretti and Barraclough (2007) based on the species listed here from Namibia.

***namibica*** Cerretti & Barraclough, 2007.—Afrotropical: Namibia.

*Anomalostomyia namibica* Cerretti & Barraclough, 2007: 103. Holotype male (NMNW). Type locality: Namibia, Brandberg Mountain, Sonusib Ravine, 1435m (21°04.546′S 14°36.958′E).

##### Genus *BLONDELIA* Robineau-Desvoidy, 1830

***BLONDELIA*** Robineau-Desvoidy, 1830: 122. Type species: *Blondelia nitida* Robineau-Desvoidy, 1830 (= *Tachina nigripes* Fallén, 1810), by subsequent designation of Duponchel *in* d’Orbigny (1842: 609) (see Evenhuis and Thompson 1990: 233) [Palaearctic].

***tibialis*** Mesnil, 1962.—Afrotropical: Burundi (**new record**, MZUR [PC]), D.R. Congo, South Africa.

*Blondelia tibialis* Mesnil, 1962: 753. Holotype male (IRSNB [not MRAC as published]). Type locality: D.R. Congo, Nord-Kivu, Kibati [ca. 1°36′S 29°16′E].

##### Genus *CHARITELLA* Mesnil, 1957

***CHARITELLA*** Mesnil, 1957: 31. Type species: *Charitella gracilis* Mesnil, 1957, by monotypy [Oriental].

*METADRINOMYIA* Shima, 1980: 259. Type species: *Metadrinomyia proclinata* Shima, 1980, by original designation [Palaearctic]. **Syn. n.**

Note: *Metadrinomyia* Shima, 1980 was first recognized from the Afrotropical Region by Cerretti (2012: 325). It is here placed in synonymy with *Charitella* Mesnil, 1957.

***nigrescens*** Mesnil, 1977.—Afrotropical: ?Madagascar, Malawi.

*Charitella nigrescens* Mesnil, 1977d: 325. Holotype female (CNC). Type locality: Malawi, Mt. Mulanje [as “Mt. Mlanje”].

***whitmorei*** (Cerretti, 2012).—Afrotropical: Burundi, D.R. Congo. **Comb. n.**

*Metadrinomyia whitmorei* Cerretti, 2012: 325. Holotype male (MZUR). Type locality: Burundi, Kayanza [Province], Parc National de la Kibira, 2200m (2°53′25.9″S 29°27′25.4″E).

Note: The recently described *Metadrinomyia whitmorei* Cerretti, 2012 is moved here to *Charitella* Mesnil, 1957.

Undescribed sp. 1: Madagascar (TAU, examined by PC).

Undescribed sp. 2: Comoros (MNHN, examined by PC).

##### Genus *COMPSILURA* Bouché, 1834

***COMPSILURA*** Bouché, 1834: 58. Type species: *Tachina concinnata* Meigen, 1824, by subsequent designation of Mik (1894: 52–53).

***concinnata*** (Meigen, 1824).—Afrotropical: “widespread W. Afr. n.-e. Afr., E. Afr. & sthn Afr.” (Crosskey 1980b: 855), including Nigeria, South Africa. Palaearctic: C. Asia, Europe (all), Japan, M. East (all), N. Africa (Madeira), Pal. China, Russia (W. Russia, W. Siberia, E. Siberia), Transcaucasia. Oriental: India, Indonesia, Malaysia, Nepal, Orien. China, Philippines, Ryukyu Is., Taiwan, Thailand. Australasian: Australia, N. Australasian. Nearctic: introduced and widespread in northeast, also British Columbia to California.

*Tachina concinnata* Meigen, 1824: 412. Holotype female (NHMW, Herting 1972: 5). Type locality: not given (probably Germany, Hamburg [specimen from von Winthem]).

*Phorocera selecta* Curran, 1940: 6. Holotype male (SANC). Type locality: South Africa, KwaZulu-Natal.

***solitaria*** (Curran, 1940).—Afrotropical: Zimbabwe.

*Phorocera solitaria* Curran, 1940: 6. Holotype male (AMNH). Type locality: Zimbabwe, Harare [as “Salisbury”].

Undescribed sp.: Madagascar (TAU, examined by PC).

Undetermined sp.: Burundi (MZUR, examined by PC).

##### Genus *DOLICHOTARSINA* Mesnil, 1977

***DOLICHOTARSINA*** Mesnil, 1977d: 324. Type species: *Dolichotarsina gracilis* Mesnil, 1977, by original designation.

***gracilis*** Mesnil, 1977.—Afrotropical: Madagascar.

*Dolichotarsina gracilis* Mesnil, 1977d: 325. Holotype female (MNHN). Type locality: Madagascar, Toamasina, Périnet [ca. 18°55′S 48°25′E].

##### Genus *EOMEDINA* Mesnil, 1960

***EOMEDINA*** Mesnil, 1960b: 652. Type species: *Eomedina grisescens* Mesnil, 1960 (= *Degeeria apicalis* Curran, 1927), by original designation.

Note: See Cerretti and Wyatt (2006) for a diagnosis of *Eomedina* Mesnil, 1960 and a key to the two species.

***apicalis*** (Curran, 1927).—Afrotropical: D.R. Congo, Kenya, Nigeria, Sierra Leone, Tanzania (**new record**, TAU [PC]), Uganda (**new record**, TAU [PC]).

*Degeeria apicalis* Curran, 1927c: 8. Holotype male (AMNH). Type locality: D.R. Congo, Orientale, Kisangani [as “Stanleyville”].

*Eomedina grisescens* Mesnil, 1960b: 651. Holotype female (BMNH). Type locality: D.R. Congo [as “Südafrika”, in error], Katanga, Bukama.

***hamoyensis*** Cerretti & Wyatt, 2006.—Afrotropical: Namibia.

*Eomedina hamoyensis* Cerretti & Wyatt, 2006: 64. Holotype female (NMNW). Type locality: Namibia, Rundu District, Hamoye National Forest (18°12′S 19°43′E).

##### Genus *EOPHYLLOPHILA* Townsend, 1926

***EOPHYLLOPHILA*** Townsend, 1926c: 19. Type species: *Eophyllophila elegans* Townsend, 1926, by original designation [Oriental].

***africana*** Villeneuve, 1935.—Afrotropical: Angola, Burundi, Cameroon, Kenya, Malawi, Nigeria, Sierra Leone, Tanzania, Uganda.

*Eophyllophila africana* Villeneuve, 1935a: 136. Syntypes, 1 male and 1 female (not located). Type localities: Nigeria (Oshogbo) and Uganda (west Rwenzori Range [as “W. Ruwenzori”], 1800m).

Undescribed spp.: Kenya, Malawi, Uganda (all in TAU, examined by PC).

##### Genus *ERYNNIOLA* Mesnil, 1977

***ERYNNIOLA*** Mesnil, 1977c: 179. Type species: *Erynniola atricolor* Mesnil, 1977, by original designation.

***atricolor*** Mesnil, 1977.—Afrotropical: Madagascar.

*Erynniola atricolor* Mesnil, 1977c: 181. Holotype male (MNHN). Type locality: Madagascar, Toamasina, Périnet [ca. 18°55′S 48°25′E].

***russipes*** Mesnil, 1977.—Afrotropical: Madagascar.

*Erynniola russipes* Mesnil, 1977c: 181. Holotype female (MNHN). Type locality: Madagascar, Toamasina, Périnet [ca. 18°55′S 48°25′E].

##### Genus *FILISTEA* Cerretti & O’Hara, gen. n.

***FILISTEA*** Cerretti & O’Hara, **gen. n.** Type species: *Viviania aureofasciata* Curran, 1927, by present designation.

Note: This new genus is described in the New Taxa of Afrotropical Tachinidae section.

***aureofasciata*** (Curran, 1927).—Afrotropical: Cameroon (**new record**, ZMHB [PC]), D.R. Congo, Nigeria, Uganda. **Comb. n.**

*Viviania aureofasciata* Curran, 1927c: 8. Holotype male (AMNH). Type locality: D.R. Congo, Orientale, Kisangani [as “Stanleyville”].

***verbekei*** Cerretti & O’Hara, **sp. n.**—Afrotropical: Cameroon, D.R. Congo, Nigeria, Uganda.

*Filistea verbekei* Cerretti & O’Hara, **sp. n.** Holotype male (ZMHB). Type locality: Cameroon, Kumba [as “Johann-Albrechtshöhe”] (4°38′N 9°28′E).

Note: This new species is described in the New Taxa of Afrotropical Tachinidae section.

##### Genus *ISTOCHETA* Rondani, 1859

*FALLENIA* Meigen, 1838: 265 (junior homonym of *Fallenia* Meigen, 1820). Type species: *Tachina longicornis* Fallén, 1810, by subsequent designation of Coquillett (1910: 544) [Palaearctic].

***ISTOCHETA*** Rondani, 1859: 157, 171. Type species: *Istocheta frontosa* Rondani, 1859 (as “Sp. Typ. nova *Frontalis* Mihi”, incorrect original spelling, see O’Hara et al. 2011: 101) (= *Phorocera cinerea* Macquart, 1850), by original designation [Palaearctic].

*ISTOCHAETA* Marschall, 1873: 334. Unjustified emendation of *Istocheta* Rondani, 1859 (see O’Hara et al. 2011: 101, 262).

*HISTOCHAETA* Brauer & Bergenstamm, 1891: 445 [also 1891: 141]. Unjustified emendation of *Istocheta* Rondani, 1859 (see O’Hara et al. 2011: 101).

*PROSOPOFRONTINA* Townsend, 1926c: 33. Type species: *Prosopofrontina pulchra* Townsend, 1926, by original designation [Oriental].

*UROPHYLLINA* Villeneuve, 1937c: 5 (as subgenus of *Urophylloides* Brauer & Bergenstamm, 1893). Type species: Urophylloides (Urophyllina) rufipes Villeneuve, 1937, by monotypy [Oriental].

*ANUROPHYLLINA* Mesnil, 1961: 693 (as subgenus of *Urophyllina* Villeneuve, 1937). *Nomen nudum* (proposed after 1930 without designation of type species from four included species) (see note below and Evenhuis et al. 2008: 6).

*ANUROPHYLLINA* Mesnil, 1977d: 322 (as subgenus of *Urophyllina* Villeneuve, 1937). Type species: *Urophylloides bicolor* Villeneuve, 1937, by original designation [Oriental].

Note: Herting (1984: 24) accepted *Anurophyllina* Mesnil, 1961 as an available name and designated *Urophylloides bicolor* Villeneuve, 1937 as type species. The availability of *Anurophyllina* Mesnil, 1961 vs. *Anurophyllina* Mesnil, 1977 was properly cited by O’Hara (1996: 121) and Evenhuis et al. (2008: 6) but O’Hara et al. (2009: 48) inadvertently followed Herting (1984).

***cerina*** (Mesnil, 1977).—Afrotropical: Madagascar.

Urophyllina (Anurophyllina) cerina Mesnil, 1977d: 322. Holotype female (MNHN). Type locality: Madagascar, Antsiranana, Montagne d’Ambre [Parc National, ca. 12°36′S 49°8′E].

***conifrons*** (Villeneuve, 1950).—Afrotropical: Uganda.

*Degeeria conifrons* Villeneuve, 1950: 2. Holotype male (IRSNB). Type locality: Uganda, Entebbe.

***crucigera*** (Mesnil, 1977).—Afrotropical: Madagascar.

Urophyllina (Anurophyllina) crucigera Mesnil, 1977d: 322. Holotype male (MNHN). Type locality: Madagascar, Toamasina, Périnet [ca. 18°55′S 48°25′E].

***flava*** (Curran, 1927).—Afrotropical: Kenya, Nigeria, Sierra Leone.

*Viviania flava* Curran, 1927f: 108. Holotype male (BMNH). Type locality: Sierra Leone, Njala [ca. 8°14′N 12°1'W].

*Degeeria frontosa* Villeneuve, 1950: 3. Holotype female (IRSNB). Type locality: Kenya, west side of Mt. Kenya, Ngare Rungai, 2000m.

##### Genus *KINIATILIOPS* Mesnil, 1955

***KINIATILIOPS*** Mesnil, 1955: 365. Type species: *Kiniatiliops elegans* Mesnil, 1955 (= *Lomatacantha nigrapex* Mesnil, 1952), by monotypy.

***bilineatus*** (Mesnil, 1952).—Afrotropical: D.R. Congo.

*Lomatacantha bilineata* Mesnil, 1952a: 11. Holotype female (not located). Type locality: D.R. Congo, Nord-Kivu, Kamatembe, 2100m [ca. 1°19′S 29°6′E].

***nigrapex*** (Mesnil, 1952).—Afrotropical: D.R. Congo, Ethiopia, Kenya, Rwanda, Tanzania, Zambia.

*Lomatacantha nigrapex* Mesnil, 1952a: 13. Holotype male (MRAC). Type locality: D.R. Congo, Nord-Kivu, Rutshuru, 1285m.

*Kiniatiliops elegans* Mesnil, 1955: 365. Holotype male (MRAC). Type locality: Rwanda, Byumba [as “terr. Biumba”, a former territory], “Gatsibu” [probably Gatsibo, ca. 1°35′S 30°15′E], 1800m.

***trispina*** Mesnil, 1959.—Afrotropical: Kenya.

*Kiniatiliops trispina* Mesnil, 1959: 14. Holotype female (SMNS). Type locality: Kenya, Lake Jipe.

##### Genus *KINIATILLA* Villeneuve, 1938

***KINIATILLA*** Villeneuve, 1938c: 10. Type species: *Kiniatilla tricincta* Villeneuve, 1938, by original designation.

*KINIATILIA*. Incorrect subsequent spelling of *Kiniatilla* Villeneuve, 1938 (Mesnil 1952a: 14).

***brevipalpis*** Mesnil, 1952.—Afrotropical: Burundi, D.R. Congo.

*Kiniatilia brevipalpis* Mesnil, 1952a: 14. Holotype male (MRAC). Type locality: D.R. Congo, Nord-Kivu, Beni to Lesse [Lesse is located northeast of Beni at ca. 0°45′N 29°46′E].

***tricincta*** Villeneuve, 1938.—Afrotropical: Burundi, D.R. Congo, Rwanda, Uganda.

*Kiniatilla tricincta* Villeneuve, 1938c: 11. Lectotype female (IRSNB), by designation herein (see Lectotype Designations section). Type locality: D.R. Congo, Bas-Congo, Mayumbé [a highland area west of Rivière Congo], Kiniati.

##### Genus *LATIGINELLA* Villeneuve, 1936

***LATIGINELLA*** Villeneuve, 1936a: 4. Type species: *Latiginella rufogrisea* Villeneuve, 1936, by monotypy.

***handeni*** Verbeke, 1963.—Afrotropical: Malawi (**new record**, NMDA [PC]), Mozambique, Tanzania.

*Latiginella handeni* Verbeke, 1963: 176. Holotype female (MRAC). Type locality: Tanzania, Handeni, 350m.

***rufogrisea*** Villeneuve, 1936.—Afrotropical: D.R. Congo, Kenya, Nigeria.

*Latiginella rufogrisea* Villeneuve, 1936a: 4. Holotype female (IRSNB). Type locality: Kenya, Ikutha.

##### Genus *LINDNERIOLA* Mesnil, 1959

***LINDNERIOLA*** Mesnil, 1959: 17. Type species: *Lindneriola paradoxa* Mesnil, 1959, by monotypy.

***paradoxa*** Mesnil, 1959.—Afrotropical: Tanzania, Uganda.

*Lindneriola paradoxa* Mesnil, 1959: 17. Holotype female (SMNS). Type locality: Tanzania, “Ngaruka” [probably Engaruka, ca. 3°0′S 35°58′E].

Undescribed sp. 1: South Africa (NMB, examined by PC).

Undescribed sp. 2: Tanzania (TAU, examined by PC).

##### Genus *MAURITIODORIA* Townsend, 1932

***MAURITIODORIA*** Townsend, 1932: 52. Type species: *Medoria spinicosta* Thomson, 1869, by original designation.

*GASTROLEPTINA* Villeneuve, 1938c: 6. Type species: *Gastroleptina discolor* Villeneuve, 1938 (= *Medoria spinicosta* Thomson, 1869), by monotypy.

***spinicosta*** (Thomson, 1869).—Afrotropical: Mauritius, Réunion.

*Medoria spinicosta* Thomson, 1869: 522. Lectotype male (NHRS), by fixation of Townsend (1932: 52) (examination of “Male Ht” from Mauritius in NHRS is regarded as a lectotype fixation). Type locality: Mauritius.

*Clytia spinicosta* Thomson, 1869: 523 (junior secondary homonym of *Medoria spinicosta* Thomson, 1869). Type(s), male (NHRS). Type locality: Mauritius.

*Gastroleptina discolor* Villeneuve, 1938c: 7. Syntypes, 1 male and 1 female (BMNH). Type locality: Mauritius.

Note: The relative priority of *Medoria spinicosta* Thomson, 1869 and *Clytia spinicosta* Thomson, 1869, when both are placed in the same genus, was established by Crosskey (1980b: 856), as the First Reviser (Article 24.2.2 of the *Code*, ICZN 1999). Townsend (1932: 52) was probably mistaken when he referred to the “male Pt” of *Medoria spinicosta* Thomson as bearing the label “*Clytia spinicosta*, Th”; this specimen is likely the name-bearing type of *Clytia spinicosta* Thomson, 1869.

##### Genus *MEDINA* Robineau-Desvoidy, 1830

***MEDINA*** Robineau-Desvoidy, 1830: 138. Type species: *Medina cylindrica* Robineau-Desvoidy, 1830 (= *Tachina collaris* Fallén, 1820), by subsequent designation of Coquillett (1910: 565) [Palaearctic].

*DEGEERIA* Meigen, 1838: 249. Type species: *Tachina collaris* Fallén, 1820, by subsequent designation of Rondani (1856: 72) [Palaearctic].

***carbonata*** Mesnil, 1968.—Afrotropical: Madagascar, South Africa, Tanzania.

*Medina carbonata* Mesnil, 1968b: 8. Holotype male (SMNS). Type locality: Tanzania, Makoa [probably near Moshi, ca. 3°21′S 37°19′E].

***cinctella*** (Villeneuve, 1950).—Afrotropical: Malawi. **Status revived.**

*Degeeria cinctella* Villeneuve, 1950: 7. Holotype male (IRSNB). Type locality: Malawi, Mt. Mulanje [as “Mt. Mlanje”].

Note: *Degeeria cinctella* Villeneuve, 1950 was treated as a synonym of *Medina lateralis* (Villeneuve, 1950) by Verbeke (1964: 181) and Crosskey (1980b: 857) but is recognized here as a distinct species based on examination of the holotype by PC. The relative priority of *Degeeria lateralis* Villeneuve, 1950 and *Degeeria cinctella* Villeneuve, 1950, when the two are treated as synonyms, was established by Verbeke (1964: 181), as the First Reviser (Article 24.2.2 of the *Code*, ICZN 1999).

***crocea*** (Villeneuve, 1950).—Afrotropical: Kenya, Malawi.

*Degeeria crocea* Villeneuve, 1950: 3. Lectotype male (IRSNB), by designation herein (see Lectotype Designations section). Type locality: Malawi, Mt. Mulanje [as “Mt. Mlanje”]).

***decellei*** Verbeke, 1964.—Afrotropical: Côte d’Ivoire.

*Medina decellei* Verbeke, 1964: 169. Holotype male (MRAC). Type locality: Côte d’Ivoire, Parc du Banco [as “Réserve du Banco”; near Abidjan].

***denticulata*** (Villeneuve, 1950).—Afrotropical: Madagascar, Nigeria.

*Degeeria denticulata* Villeneuve, 1950: 6. Holotype female (IRSNB). Type locality: Nigeria, Ilesha.

***egregia*** (Villeneuve, 1950).—Afrotropical: Nigeria, Zambia, Zimbabwe.

*Degeeria egregia* Villeneuve, 1950: 4. Holotype male (IRSNB). Type locality: Nigeria, Oshogbo.

***lateralis*** (Villeneuve, 1950).—Afrotropical: Burundi, D.R. Congo (**new record**, IRSNB [PC]), Rwanda, South Africa, Tanzania.

*Degeeria lateralis* Villeneuve, 1950: 7. Holotype male (IRSNB). Type locality: South Africa, Western Cape, Cape of Good Hope [as “Cap”].

***mira*** Mesnil, 1977.—Afrotropical: Madagascar.

*Medina mira* Mesnil, 1977c: 185. Holotype male (MNHN). Type locality: Madagascar, Mahajanga, Ambato Boeni.

***nigra*** Mesnil, 1968.—Afrotropical: Angola, Madagascar, South Africa.

*Medina nigra* Mesnil, 1968b: 8. Holotype male (SMNS). Type locality: South Africa, Western Cape, Cape Town.

***pectinifera*** Mesnil, 1977.—Afrotropical: Madagascar.

*Medina pectinifera* Mesnil, 1977c: 187. Holotype female (MNHN). Type locality: Madagascar, Antsiranana, Montagne d’Ambre [Parc National, ca. 12°36′S 49°8′E].

***rubricosa*** (Villeneuve, 1913).—Afrotropical: Nigeria.

*Lydella rubricosa* Villeneuve, 1913c: 30. Holotype female (BMNH). Type locality: Nigeria, Oshogbo.

Note: Villeneuve’s (1913c: 30–31) description of a single female of *Lydella rubricosa* from Nigeria was followed by a brief description of a male from Benin (as “Dahomey”). It is not clear whether this male was thought to be conspecific with *Lydella rubricosa* and hence part of the type series of this nominal species. We have inferred that the male was not positively associated with the female and is therefore not a syntype of *Lydella rubricosa*, and have followed Crosskey (1980b: 857) in excluding Benin from the distribution of *Lydella rubricosa* (Villeneuve).

***semirufa*** (Villeneuve, 1950).—Afrotropical: Kenya, Malawi.

*Degeeria semirufa* Villeneuve, 1950: 6. Lectotype female (IRSNB), by designation herein (see Lectotype Designations section). Type locality: Malawi, Mt. Mulanje [as “Mt. Mlanje”].

***setosella*** (Villeneuve, 1950).—Afrotropical: Burundi (**new record**, IRSNB [PC]), Cameroon, D.R. Congo (**new record**, IRSNB [PC]), Uganda.

*Degeeria setosella* Villeneuve, 1950: 5. Holotype male (IRSNB). Type locality: northwest Cameroon, Dschang [as “Dchang”] Plateau.

***sopha*** Mesnil, 1977.—Afrotropical: Madagascar.

*Medina sopha* Mesnil, 1977c: 184. Holotype male (MNHN). Type locality: Madagascar, Toamasina, Périnet, 1000m [ca. 18°55′S 48°25′E].

***spinulifera*** Mesnil, 1968.—Afrotropical: Tanzania.

*Medina spinulifera* Mesnil, 1968b: 9. Holotype female (SMNS). Type locality: Tanzania, Makoa [probably near Moshi, ca. 3°21′S 37°19′E].

***succuba*** Mesnil, 1977.—Afrotropical: Madagascar.

*Medina succuba* Mesnil, 1977c: 186. Holotype male (MNHN). Type locality: Madagascar, Toamasina, Moramanga.

***vidua*** Mesnil, 1977.—Afrotropical: Madagascar.

*Medina vidua* Mesnil, 1977c: 187. Holotype female (MNHN). Type locality: Madagascar, Toamasina, Périnet [ca. 18°55′S 48°25′E].

Possibly undescribed sp.: Nigeria (Crosskey 1984: 265).

##### Genus *MEIGENIA* Robineau-Desvoidy, 1830

***MEIGENIA*** Robineau-Desvoidy, 1830: 198. Type species: *Meigenia cylindrica* Robineau-Desvoidy, 1830, by subsequent designation of Desmarest *in* d’Orbigny (1849a: 318, as “*Tachina* [*Tachina*] *cylindrica*”) (see Evenhuis and Thompson 1990: 237) [Palaearctic].

Note: *Meigenia cylindrica* Robineau-Desvoidy, 1830 is accepted as the type species of *Meigenia* Robineau-Desvoidy, 1830, following Evenhuis and Thompson (1990: 237). This name was treated as a *nomen dubium* under *Meigenia* by Herting and Dely-Draskovits (1993: 147). Despite this treatment of the type species of *Meigenia* as a *nomen dubium*, the concept of *Meigenia* is well-established and no useful purpose would be served by calling it into question over the dubious identity of *Meigenia cylindrica*.

Undetermined sp.: Yemen (Zeegers 2007: 388).

##### Genus *MEDINOSPILA* Mesnil, 1977

***MEDINOSPILA*** Mesnil, 1977d: 322. Type species: *Medinospila nigella* Mesnil, 1977, by original designation.

***nigella*** Mesnil, 1977.—Afrotropical: Madagascar.

*Medinospila nigella* Mesnil, 1977d: 323. Holotype male (MNHN). Type locality: Madagascar, Toamasina, Périnet [ca. 18°55′S 48°25′E].

##### Genus *PARARONDANIA* Villeneuve, 1916

***PARARONDANIA*** Villeneuve, 1916c: 498. Type species: *Pararondania multipunctata* Villeneuve, 1916, by monotypy.

***multipunctata*** Villeneuve, 1916.—Afrotropical: South Africa.

*Pararondania multipunctata* Villeneuve, 1916c: 498. Holotype female (CNC [not SAMC as published]). Type locality: South Africa, “Cape Colony” (“S.W. Distr. Cape Col.” according to label data, Cooper and O’Hara 1996: 58; possibly referring to present-day Western Cape, Cape of Good Hope).

##### Genus *PARATRIXA* Brauer & Bergenstamm, 1891

***PARATRIXA*** Brauer & Bergenstamm, 1891: 357 [also 1891: 53]. Type species: *Paratrixa polonica* Brauer & Bergenstamm, 1891, by monotypy [Palaearctic]. **New record.**

Note: Mesnil (1952a) described the two Afrotropical species below in *Paratrixa* Brauer & Bergenstamm, 1891. Crosskey (1980b: 857) did not recognize *Paratrixa* and placed these two species in *Medina* Robineau-Desvoidy, 1830. *Paratrixa* is treated as a genus in the Palaearctic Region (e.g., Herting and Dely-Draskovits 1993: 153, Cerretti 2010: 128) and is reinstated here as an Afrotropical genus with these same two species.

***aethiopica*** Mesnil, 1952.—Afrotropical: D.R. Congo, Rwanda, South Africa. **Comb. revived.**

*Paratrixa aethiopica* Mesnil, 1952a: 10. Holotype female (not located). Type locality: Rwanda, Ruhengeri [1°30′S 29°38′E], “sources Kirii” [not located], 1800–1825m.

***stammeri*** Mesnil, 1952.—Afrotropical: D.R. Congo, South Africa (**new record**, IRSNB [PC]). **Comb. revived.**

*Paratrixa stammeri* Mesnil, 1952a: 9. Holotype male (not located). Type locality: D.R. Congo, Nord-Kivu, Rutshuru, 1285m.

##### Genus *PELASHYRIA* Villeneuve, 1935

***PELASHYRIA*** Villeneuve, 1935a: 138. Type species: *Pelashyria grisescens* Villeneuve, 1935, by monotypy.

***grisescens*** Villeneuve, 1935.—Afrotropical: D.R. Congo.

*Pelashyria grisescens* Villeneuve, 1935a: 139. Syntypes, 1 male and 1 female (IRSNB). Type locality: D.R. Congo, Nord-Kivu, Mukule, 1800m [ca. 1°20′S 29°15′E].

##### Genus *PRODEGEERIA* Brauer & Bergenstamm, 1894

***PRODEGEERIA*** Brauer & Bergenstamm, 1894: 617 [also 1895: 81]. Type species: *Prodegeeria javana* Brauer & Bergenstamm, 1894, by monotypy [Oriental].

*MYXHYPOSTENA* Villeneuve, 1939: 6. Type species: *Myxhypostena consobrina* Villeneuve, 1939, by original designation.

Note: Villeneuve (1939: 6) wrote about his new genus *Myxhypostena*: “le scutellum du type à 4 soies marginales”. This statement is accepted as a type species designation for *Myxhypostena* of the single included species, *Myxhypostena consobrina* Villeneuve.

***consobrina*** (Villeneuve, 1939).—Afrotropical: D.R. Congo, Ghana, Nigeria.

*Myxhypostena consobrina* Villeneuve, 1939: 6. Syntypes, 1 male and 1 female (IRSNB). Type localities: D.R. Congo (“Agangula” [not located]) and Nigeria (Oshogbo).

***straeleni*** Mesnil, 1952.—Afrotropical: D.R. Congo, Uganda.

*Prodegeeria straeleni* Mesnil, 1952a: 14. Holotype male (IRSNB). Type locality: D.R. Congo, Équateur, Eala.

##### Genus *PROSUCCINGULUM* Mesnil, 1959

***PROSUCCINGULUM*** Mesnil, 1959: 16. Type species: *Prosuccingulum aberrans* Mesnil, 1959, by monotypy.

***aberrans*** Mesnil, 1959.—Afrotropical: Tanzania.

*Prosuccingulum aberrans* Mesnil, 1959: 16. Holotype female (SMNS). Type locality: Tanzania, west side of Mt. Kibo [one of the three peaks of Mt. Kilimanjaro], 2800m.

Undescribed sp.: Malawi (NMB, examined by PC).

##### Genus *RIOTERIA* Herting, 1973

***RIOTERIA*** Herting, 1973: 3. Type species: *Rioteria submacula* Herting, 1973, by monotypy [Palaearctic].

***flava*** Zeegers, 2007.—Afrotropical: Yemen.

*Rioteria flava* Zeegers, 2007: 395. Holotype male (RMNH). Type locality: Yemen, 12km northwest of Manākhah (15°04′19″N 43°44′27″E).

***rufitibia*** (Mesnil, 1959).—Afrotropical: Nigeria, Tanzania.

*Tachinophytopsis rufitibia* Mesnil, 1959: 14. Holotype male (SMNS). Type locality: Tanzania, “Ngaruka” [probably Engaruka, ca. 3°0′S 35°58′E].

Undescribed sp. 1: South Africa (NMB, examined by PC).

Undescribed sp. 2: Burkina (MZUR, examined by PC).

##### Genus *TRIGONOSPILA* Pokorny, 1886

***TRIGONOSPILA*** Pokorny, 1886: 191. Type species: *Trigonospila picta* Pokorny, 1886 (= *Tachina ludio* Zetterstedt, 1849), by monotypy [Palaearctic].

*SUCCINGULUM* Pandellé, 1894: 52. Type species: *Succingulum transvittatum* Pandellé, 1896, by subsequent monotypy of Pandellé (1896: 148) [Palaearctic].

***bimaculata*** (Villeneuve, 1935).—Afrotropical: Ghana, Malawi, Mozambique, Nigeria, Sudan, Uganda.

*Succingulum bimaculatum* Villeneuve, 1935a: 142. Holotype female (IRSNB). Type locality: Malawi.

Note: Villeneuve (1935a: 142) cited a second female of *Succingulum bimaculatum* seen by W.S. Patton but it was not examined by Villeneuve (as evidenced from his statement, “La tarière est exserte sur l’unique ♀ que j’ai vue”) and hence is not a syntype.

***exigua*** (Villeneuve, 1935).—Afrotropical: South Africa. **Status revived.**

*Succingulum exiguum* Villeneuve, 1935a: 142. Holotype male (IRSNB). Type locality: South Africa.

Note: *Succingulum exiguum* Villeneuve, 1935 was treated as a synonym of *Trigonospila mista* (Villeneuve, 1913) by Crosskey (1980b: 858) but is recognized here as a distinct species based on examination of the holotype by PC.

***integra*** (Villeneuve, 1935).—Afrotropical: “Afrique”. Oriental: India, Myanmar.

*Succingulum integrum* Villeneuve, 1935a: 142. Holotype male (possibly lost, Crosskey 1976: 218). Type locality: Africa [as “Afrique (région?)”].

***mista*** (Villeneuve, 1913).—Afrotropical: Angola, D.R. Congo, Kenya, Malawi, ?South Africa, Tanzania, Uganda.

*Succingulum mista* Villeneuve, 1913c: 39. Holotype female (IRSNB). Type locality: D.R. Congo, Katanga, Sankisia.

***prasius*** Mesnil, 1977.

***prasiusprasius*** Mesnil, 1977.—Afrotropical: Madagascar.

*Trigonospila prasius prasius* Mesnil, 1977c: 181, 183. Holotype male (MNHN). Type locality: Madagascar, Toamasina, Périnet [ca. 18°55′S 48°25′E].

***prasius trifida*** Mesnil, 1977.—Afrotropical: Madagascar.

*Trigonospila prasius trifidus* Mesnil, 1977c: 183. Holotype male (MNHN). Type locality: Madagascar, Antananarivo, Antananarivo [as “Tananarive”].

##### Unplaced species of Blondeliini

***triquetra*** Macquart, 1844.—Afrotropical: Réunion.

*Dexia triquetra* Macquart, 1844: 86 [also 1844: 243]. Lectotype male (MNHN), by fixation of Crosskey (1971: 267) (examination of “Holotype ♂” from Réunion in MNHN is regarded as a lectotype fixation). Type locality: Réunion [as “Bourbon”].

#### Tribe ERYCIINI

##### Genus *AFROPHYLAX* Cerretti & O’Hara, gen. n.

***AFROPHYLAX*** Cerretti & O’Hara, **gen. n.** Type species: *Sturmia aureiventris* Villeneuve, 1910, by present designation.

Note: This new genus is described in the New Taxa of Afrotropical Tachinidae section.

***aureiventris*** (Villeneuve, 1910).—Afrotropical: Cameroon (**new record**, ZMHB [PC]), D.R. Congo, Nigeria, Sierra Leone, Tanzania, Uganda. **Comb. n.**

*Sturmia aureiventris* Villeneuve, 1910a: 252. Holotype male (MRAC). Type locality: D.R. Congo (as “Congo”, p. 249).

Note: Villeneuve (1910a) described four species from “Congo”. Curran (1927f: 122) treated one of them (*Sturmia aureiventris* Villeneuve, 1910) as described from D.R. Congo (as “Belgian Congo”), and used “Belgian Congo” and “Congo” interchangeably in this work and some others. We think it likely that Villeneuve (1910a), like Curran, used “Congo” in the sense of present-day D.R. Congo. However, Crosskey (1980b) interpreted Villeneuve’s Congo as the present-day country of Congo. Crosskey (1980b: 867, 1984: 277) treated *Sturmia aureiventris* Villeneuve as an unplaced species in the “Carceliini”.

##### Genus *ANTISTASEA* Bischof, 1904

***ANTISTASEA*** Bischof, 1904: 82. Type species: *Antistasea fimbriata* Bischof, 1904, by monotypy.

***fimbriata*** Bischof, 1904.—Afrotropical: Kenya (**new record**, TAU [PC]), South Africa, Zimbabwe.

*Antistasea fimbriata* Bischof, 1904: 83. Lectotype male (NHMW), by fixation of Townsend (1941: 235) (mention of “Ht male” from Algoa Bay in NHMW is regarded as a lectotype fixation). Type locality: South Africa, Eastern Cape, Algoa Bay.

*Podomyia discalis* Curran, 1939: 2. Holotype male (AMNH). Type locality: Zimbabwe, Harare [as “Salisbury”]. **Syn. n.**

Note: Crosskey (1984: 289) commented that *Podomyia discalis* Curran, 1939 is “almost certainly synonymous” with *Antistasea fimbriata* Bischof, 1904. We confirm from examination of the name-bearing types that these names are synonyms.

***mutans*** Mesnil, 1970.—Afrotropical: Botswana, South Africa.

*Antistasea mutans* Mesnil, 1970b: 106. Holotype male (CNC). Type locality: South Africa, KwaZulu-Natal, Mfongosi [ca. 28°43′S 30°49′E].

##### Genus *APLOMYA* Robineau-Desvoidy, 1830

***APLOMYA*** Robineau-Desvoidy, 1830: 184. Type species: *Aplomya zonata* Robineau-Desvoidy, 1830 (= *Tachina confinis* Fallén, 1820), by subsequent designation of Robineau-Desvoidy (1863a: 459, 460) (as *confinis*, with *zonata* in synonymy) [Palaearctic].

*APLOMYIA* Agassiz, 1846a: 3. Unjustified emendation of *Aplomya* Robineau-Desvoidy, 1830 (see Evenhuis et al. 2010: 39).

*HAPLOMYIA* Agassiz, 1846b: 172. Unjustified emendation of *Aplomya* Robineau-Desvoidy, 1830 (see Evenhuis et al. 2010: 39).

*PROZENILLIA* Villeneuve, 1916c: 487. Type species: *Prozenillia distans* Villeneuve, 1916, by monotypy.

*WIEDEMANNIOMYIA* Townsend, 1933: 469. Type species: *Tachina metallica* Wiedemann, 1824, by original designation.

*APLOMYIELLA* Mesnil, 1939d: 31. Type species: *Tricholyga impexa* Villeneuve, 1916 (= *Tachina metallica* Wiedemann, 1824), by original designation.

*ATRICHOLYGA* Villeneuve, 1939: 9. Type species: *Tricholyga impexa* Villeneuve, 1916 (= *Tachina metallica* Wiedemann, 1824), by monotypy.

***confinis*** (Fallén, 1820).—Afrotropical: ?Malawi, Yemen. Palaearctic: C. Asia, Europe (all), Japan, M. East (all), Mongolia, N. Africa (Canary Is., Madeira), Pal. China, Russia (W. Russia, W. Siberia, E. Siberia, S. Far East), Transcaucasia. Oriental: Orien. China.

*Tachina confinis* Fallén, 1820: 32. Syntypes, males and females (NHRS and/or MZLU). Type locality: Sweden, Gotland.

Note: *Tachina confinis* Fallén, 1820 was recorded from Malawi by Villeneuve (1913c: 32) but not by Crosskey (1980b: 876). The presence of this species in Malawi needs confirmation.

***distans*** (Villeneuve, 1916).—Afrotropical: Nigeria, South Africa, Sudan, Uganda.

*Prozenillia distans* Villeneuve, 1916c: 488. Lectotype male (SAMC, not located by JEOH), by fixation of Townsend (1940: 311) (mention of “Ht male” from Durban in SAMC is regarded as a lectotype fixation, if type can be found in SAMC). Type locality: South Africa, KwaZulu-Natal, Durban.

***latimana*** Villeneuve, 1934.—Afrotropical: D.R. Congo, Kenya, Uganda.

*Aplomyia latimana* Villeneuve, 1934c: 409. Holotype female (CNC). Type locality: Uganda, Rwenzori Range [as “Ruwenzori”], 1800m.

***lycaena*** (Curran, 1927).—Afrotropical: Ethiopia, Senegal, South Africa.

*Zenillia lycaena* Curran, 1927d: 333. Holotype male (SANC). Type locality: South Africa, Free State, Bloemfontein.

***metallica*** (Wiedemann, 1824).—Afrotropical: “W. Afr. to n.-e. Afr., E. Afr. & sthn Afr.” (Crosskey 1980b: 876), including D.R. Congo, Mozambique, South Africa, Sudan, U.A. Emirates, Yemen. Palaearctic: Japan, M. East (Israel), Pal. China. Oriental: India, Indonesia, Orien. China, Ryukyu Is., Taiwan. Australasian: N. Australasian.

*Tachina metallica* Wiedemann, 1824: 46. Lectotype male (ZMUC), by fixation of Townsend (1933: 470) (examination of “Male holotype” from East Indies in ZMUC is regarded as a lectotype fixation). Type locality: “India orient.” (i.e., “East Indies”).

*Tachina nigriventris* Wiedemann, 1824: 43. Lectotype male (ZMUC), by fixation of Townsend (1933: 470) (examination of “male holotype” from East Indies in ZMUC is regarded as a lectotype fixation). Type locality: “India orient.” (i.e., “East Indies”).

*Tachina notata* Wiedemann, 1830: 653. Type(s), male (SMF or lost). Type locality: Nubia region [as “Nubien”, a region in southern Egypt and northern Sudan, recorded here as Sudan following Crosskey 1980b: 876].

*Tachina socia* Wiedemann, 1830: 654. Type(s), female (SMF or lost). Type locality: not given (likely Nubia region).

*Phorocera eucalypta* Loew, 1852: 659 [also 1862: 19, full description]. Type(s), unspecified sex (1 male in ZMHB). Type locality: Mozambique (Tete [as “Tette”] according to Loew 1862: 20).

*Parexorista laeviventris* van der Wulp, 1893: 173. Lectotype male (RMNH), by designation of Crosskey (1966a: 674–675) (see also Crosskey 1969: 105). Type locality: Indonesia, Jawa.

*Tricholyga impexa* Villeneuve, 1916c: 494. Syntypes, 2 males (1 male in NHMW). Type localities: D.R. Congo [as “Congo”, but received from Bequaert and presumably collected from D.R. Congo] and South Africa (Eastern Cape, Uitenhage).

Note: The relative priority of *Tachina metallica* Wiedemann, 1824 and *Tachina nigriventris* Wiedemann, 1824, when the two are treated as synonyms, was established by Townsend (1933: 470), as the First Reviser (Article 24.2.2 of the *Code*, ICZN 1999). *Tachina notata* Wiedemann, 1830 and *Tachina socia* Wiedemann, 1830 were synonymized with *Tachina metallica* by Crosskey (1980b: 876); their relative priority has not been established by a First Reviser and such action is unnecessary while they are invalid names.

The male syntype of *Tricholyga impexa* Villeneuve, 1916 in NHMW was collected from Uitenhage, South Africa, on 15 November 1896 and not on 15 December 1896 as given by Villeneuve (1916c: 494) (examined by JEOH).

***poultoni*** (Villeneuve, 1922).—Afrotropical: Kenya, Nigeria, South Africa.

*Exorista poultoni* Villeneuve, 1922a: 518. Holotype male (not located). Type locality: Nigeria, near Ibadan, Moor Plantation.

***seyrigi*** Mesnil, 1954.—Afrotropical: Madagascar.

Aplomyia (Aplomyiella) seyrigi Mesnil, 1954: 330. Holotype male (MNHN). Type locality: Madagascar, Toliara, Bekily.

***versicolor*** (Curran, 1927).—Afrotropical: South Africa, Uganda.

*Zenillia versicolor* Curran, 1927d: 334. Holotype male (SANC). Type locality: South Africa, Eastern Cape, East London.

##### Genus *CADURCIELLA* Villeneuve, 1927

***CADURCIELLA*** Villeneuve, 1927: 120. Type species: *Cadurciella rufipalpis* Villeneuve, 1927, by monotypy.

***rufipalpis*** Villeneuve, 1927.—Afrotropical: Namibia, South Africa, Zimbabwe. Palaearctic: M. East (Israel).

*Cadurciella rufipalpis* Villeneuve, 1927: 120. Lectotype male (not located), by fixation of Townsend (1941: 248) (mention of “Ht male” from Salisbury in Rambouillet [Villeneuve’s personal collection, since dispersed] is regarded as a lectotype fixation for the single male in the type series from this locality). Type locality: Zimbabwe, Harare [as “Salisbury”].

***uniseta*** (Curran, 1933).—Afrotropical: South Africa, Zimbabwe.

*Zenillia uniseta* Curran, 1933: 166. Holotype male (BMNH). Type locality: Zimbabwe.

Undetermined sp.: U.A. Emirates, as “cf. *Cadurciella* spec.” (Zeegers 2010: 681).

##### Genus *CARCELIA* Robineau-Desvoidy, 1830

###### Subgenus *CARCELIA* Robineau-Desvoidy, 1830

***CARCELIA*** Robineau-Desvoidy, 1830: 176. Type species: *Carcelia bombylans* Robineau-Desvoidy, 1830, by subsequent designation of Coquillett (1910: 518) (see Evenhuis et al. 2010: 52) [Palaearctic].

*CARCELLIA*. Incorrect subsequent spelling of *Carcelia* Robineau-Desvoidy, 1830 (Rondani 1859: 103, Stackelberg 1943: 163) (see O’Hara et al. 2011: 46).

***nudioculata*** Villeneuve, 1938.—Afrotropical: D.R. Congo, Rwanda, Uganda.

*Carcelia nudioculata* Villeneuve, 1938c: 4. Holotype male (not located). Type locality: D.R. Congo, Maniema, Lubutu.

###### Subgenus *CARCELITA* Mesnil, 1975

***CARCELITA*** Mesnil, 1975a: 1384. Type species: *Carcelia peraequalis* Mesnil, 1950, by monotypy.

*CARICELIA* Mesnil, 1975a: 1384. *Nomen nudum* (proposed after 1930 without designation of type species; no included species).

*CARICELIA* Mesnil, 1975b: 1388. Type species: *Carcelia obliterata* Mesnil, 1950, by original designation.

Note: See O’Hara (1996: 122) for an explanation of the nomenclatural history of *Caricelia* Mesnil and *Carcelita* Mesnil.

***abrelicta*** Mesnil, 1950.—Afrotropical: Burundi, D.R. Congo, South Africa, Tanzania, Uganda.

*Carcelia abrelicta* Mesnil, 1950b: 16. Syntypes, males and females (1 female in CNC). Type localities: D.R. Congo and South Africa (Western Cape, Cape Town).

***aequalis*** Villeneuve, 1939.—Afrotropical: Nigeria, South Africa, Tanzania, Zimbabwe.

*Carcelia aequalis* Villeneuve, 1939: 1. Syntypes, males (“plusieurs individus”) (1 male in CNC, 1 male in SAMC). Type locality: South Africa, KwaZulu-Natal.

Note: One male of *Carcelia aequalis* Villeneuve, 1939 in IRSNB from “Stella B” [former Stella Bush near Durban] (examined by PC) with a Villeneuve determination label is likely an unmarked syntype.

***angulicornis*** Villeneuve, 1916.—Afrotropical: Ghana (**new record**, CNC), Malawi, Nigeria, Sierra Leone, South Africa.

*Carcelia angulicornis* Villeneuve, 1916c: 481. Syntypes, males and females (BMNH, CNC). Type localities: Malawi (Mulanje [as “Mlange”]), Nigeria (Oshogbo), and South Africa.

***argyriceps*** (Curran, 1927).—Afrotropical: Uganda.

*Zenillia argyriceps* Curran, 1927d: 328. Holotype male (BMNH). Type locality: Uganda, [Kanungu District in southwestern Uganda], Kinkizi County, “Kizazi” [not located].

*Zenillia hargreavesi* Curran, 1928a: 238. Holotype male (BMNH). Type locality: Uganda, Kampala.

***atricans*** Mesnil, 1955.—Afrotropical: Burundi (**new record**, CNC, MZUR [PC]), ?Cape Verde, Kenya (**new record**, CNC), Rwanda, Tanzania.

*Carcelia atricans* Mesnil, 1955: 362. Holotype male (MRAC). Type locality: Rwanda, eastern foothills of Volcan Muhabura [as “Muhavura”], 2100m [ca. 1°23′S 29°44′E].

***bigoti*** (Jaennicke, 1867).—Afrotropical: Ethiopia.

*Exorista bigoti* Jaennicke, 1867: 384 [also 1868: 76]. Type(s), female (SMF). Type locality: Ethiopia, “Simen” (probably the Simien Mountains area).

***forcipata*** Mesnil, 1977.—Afrotropical: Madagascar.

Carcelia (Carcelita) forcipata Mesnil, 1977b: 178. Holotype male (MNHN). Type locality: Madagascar, Antananarivo, Ampefy.

***inusta*** Mesnil, 1950.—Afrotropical: Malawi (**new record**, CNC), South Africa.

*Carcelia inusta* Mesnil, 1950b: 11. Syntypes, males and females (1 male in CNC). Type locality: South Africa, KwaZulu-Natal, “Stella” [former Stella Bush near Durban].

***keiseri*** Mesnil, 1977.—Afrotropical: Madagascar.

Carcelia (Carcelita) keiseri Mesnil, 1977b: 176. Holotype male (MNHN). Type locality: Madagascar, Toamasina, Périnet, 1000m [ca. 18°55′S 48°25′E].

***lindneri*** Mesnil, 1959.—Afrotropical: South Africa (**new record**, CNC), Tanzania.

*Carcelia lindneri* Mesnil, 1959: 2. Holotype male (SMNS). Type locality: Tanzania, Msingi [ca. 4°20′S 34°34′E].

***lucidula*** Villeneuve, 1941.—Afrotropical: C.A. Republic (**new record**, CNC), D.R. Congo.

*Carcelia lucidula* Villeneuve, 1941b: 125. Syntypes, 2 males and 1 female (2 males in MRAC, 1 female in CNC). Type locality: D.R. Congo, Orientale, Uele, Dembia.

***normula*** (Curran, 1927).—Afrotropical: D.R. Congo, Ghana, Nigeria, Tanzania, Uganda.

*Zenillia normula* Curran, 1927d: 329. Holotype female (BMNH). Type locality: Uganda, “Rosaka” [not located].

***oblectanea*** Mesnil, 1950.—Afrotropical: D.R. Congo, Kenya, South Africa (**new record**, CNC).

*Carcelia oblectanea* Mesnil, 1950b: 15. Syntypes, males and females (1 female in CNC). Type locality: D.R. Congo.

***oblimata*** Mesnil, 1950.—Afrotropical: South Africa.

*Carcelia oblimata* Mesnil, 1950b: 14. Syntypes, males and females (1 female in CNC). Type locality: South Africa, Western Cape, Cape Town.

***obliterata*** Mesnil, 1950.—Afrotropical: Rwanda, South Africa.

*Carcelia obliterata* Mesnil, 1950b: 13. Lectotype female (CNC), by fixation of O’Hara (1996: 150). Type locality: South Africa (“Kransp.” according to label data, Cooper and O’Hara 1996: 21).

Note: O’Hara (1996: 150) accepted the specimen labelled as “TYPE” in CNC as the holotype of *Carcelia obliterata* Mesnil, 1950 under the assumption that the species was likely described from a single specimen. This assumption is contrary to Recommendation 73F, “Avoidance of assumption of holotype”, of the current *Code* (ICZN 1999). O’Hara’s (1996: 150) treatment of the “TYPE” in CNC as the holotype of *Carcelia obliterata* is regarded as a lectotype fixation.

***oculata*** (Villeneuve, 1910).—Afrotropical: D.R. Congo.

*Exorista oculata* Villeneuve, 1910a: 251. Lectotype male (IRSNB), by designation herein (see Lectotype Designations section). Type locality: D.R. Congo (as “Congo”, p. 249).

*occulata*. Incorrect subsequent spelling of *oculata* Villeneuve, 1910 (Curran 1927d: 335).

Note: Villeneuve (1910a) described four species from “Congo”. Curran (1927f: 122) treated one of them (*Sturmia aureiventris* Villeneuve, 1910) as described from D.R. Congo (as “Belgian Congo”), and used “Belgian Congo” and “Congo” interchangeably in this work and some others. We think it likely that Villeneuve (1910a), like Curran, used “Congo” in the sense of present-day D.R. Congo. However, Crosskey (1980b) interpreted Villeneuve’s Congo as the present-day country of Congo.

***orbitalis*** (Curran, 1927).—Afrotropical: South Africa, Zimbabwe.

*Zenillia orbitalis* Curran, 1927d: 330. Holotype male (SANC). Type locality: South Africa, Gauteng, Pretoria.

***patellata*** Mesnil, 1977.—Afrotropical: Madagascar.

Carcelia (Carcelita) patellata Mesnil, 1977b: 177. Holotype female (MNHN). Type locality: Madagascar, Antsiranana, Montagne d’Ambre [Parc National, ca. 12°36′S 49°8′E].

***pellex*** Mesnil, 1950.—Afrotropical: Kenya, South Africa, Uganda.

*Carcelia pellex* Mesnil, 1950b: 13. Type(s), unspecified sex (not located). Type locality: South Africa.

***peraequalis*** Mesnil, 1950.—Afrotropical: D.R. Congo, Kenya, Lesotho, Malawi, Rwanda, South Africa, Tanzania, Uganda, Zimbabwe.

*Carcelia peraequalis* Mesnil, 1950b: 18. Syntypes, males and females (possibly 1 male in CNC [O’Hara 1996: 152], 1 male in IRSNB). Type locality: Zimbabwe, Harare [as “Salisbury”].

***persimilis*** Mesnil, 1950.—Afrotropical: Madagascar, South Africa.

*Carcelia persimilis* Mesnil, 1950b: 17. Lectotype male (MNHN), by fixation of O’Hara (1996: 153) (treatment of a male labelled as “TYPE” from Fort-Dauphin in MNHN as the holotype is regarded as a lectotype fixation). Type locality: Madagascar, Toliara, Tôlanaro [also commonly known as Taolagnaro or Fort Dauphin and published as “Fort-Dauphin”].

Note: O’Hara (1996: 153) accepted the specimen labelled as “TYPE” in MNHN as the holotype of *Carcelia persimilis* Mesnil, 1950 under the assumption the species was likely described from a single specimen. However, Mesnil’s description (1950b: 17) clearly mentions both sexes, thus indicating syntypes. O’Hara’s (1996: 153) treatment of the “TYPE” in MNHN as the holotype of *Carcelia persimilis* is regarded as a lectotype fixation.

***vaga*** (Curran, 1927).—Afrotropical: Uganda.

*Zenillia vaga* Curran, 1927d: 332. Holotype male (BMNH). Type locality: Uganda, Kampala.

***vara*** (Curran, 1927).—Afrotropical: Ghana, Kenya, South Africa, Tanzania.

*Zenillia vara* Curran, 1927d: 331. Holotype male (BMNH). Type locality: Kenya, Kabete [ca. 1°16′S 36°43′E, near Nairobi].

***vexor*** (Curran, 1927).—Afrotropical: South Africa.

*Zenillia vexor* Curran, 1927d: 330. Holotype male (SANC). Type locality: South Africa, KwaZulu-Natal, Durban.

###### Subgenus *EURYCLEA* Robineau-Desvoidy, 1863

*EURYCLEA* Robineau-Desvoidy, 1863a: 290. Type species: *Euryclea tibialis* Robineau-Desvoidy, 1863, by original designation [Palaearctic].

***setifrons*** Mesnil, 1949.—Afrotropical: D.R. Congo, Nigeria (**new record**, CNC), Uganda.

Carcelia (Eucarcelia) setifrons Mesnil, 1949a: 90. Holotype male (MRAC). Type locality: D.R. Congo, Katanga, Lubumbashi [as “Elisabethville”].

Possibly undescribed spp.: Yemen, as “Carcelia (Caricelia) sp. 1 cf. vexor”, “Carcelia (Caricelia) sp. 2”, and “Carcelia (Caricelia) sp. 3” (Zeegers 2007: 378).

##### Genus *CARCELIATHRIX* Cerretti & O’Hara, gen. n.

***CARCELIATHRIX*** Cerretti & O’Hara, **gen. n.** Type species: *Phorocera crassipalpis* Villeneuve, 1938, by present designation.

Note: This new genus is described in the New Taxa of Afrotropical Tachinidae section.

***crassipalpis*** (Villeneuve, 1938).—Afrotropical: D.R. Congo. **Comb. n.**

*Phorocera crassipalpis* Villeneuve, 1938c: 2. Lectotype male (MRAC), by designation herein (see Lectotype Designations section). Type locality: D.R. Congo, Équateur, Bomputu.

*claripalpis*. Incorrect subsequent spelling of *crassipalpis* Villeneuve, 1938 (original usage not found but spelling listed by Crosskey 1980b: 867).

Note: Crosskey (1980b: 867) treated *Phorocera crassipalpis* Villeneuve, 1938 as an unplaced species in the “Carceliini”.

Undescribed sp. 1: Namibia (NNIC, examined by PC).

Undescribed sp. 2: South Africa (NMB, examined by PC).

##### Genus *CESTONIA* Rondani, 1861

***CESTONIA*** Rondani, 1861b: 105. Type species: *Cestonia cineraria* Rondani, 1861, by monotypy [Palaearctic].

***canariensis*** Villeneuve, 1936.—Afrotropical: U.A. Emirates. Palaearctic: N. Africa (Canary Is.), M. East (Israel).

*Cestonia canariensis* Villeneuve *in* Frey, 1936: 145. Syntypes, 1 male and 1 female (FMNHH). Type locality: Canary Islands, Gran Canaria, Las Palmas de Gran Canaria.

Note: *Cestonia canariensis* Villeneuve, 1936, was redescribed by Herting (1981: 3) from the original syntypes.

***harteni*** Zeegers, 2007.—Afrotropical: Yemen.

*Cestonia harteni* Zeegers, 2007: 381. Holotype female (RMNH). Type locality: Yemen, Suq Bani Mansour (15°05′15″N 43°52′10″E).

Note: Zeegers (2010: 677) recognized “Cestonia cf. harteni Zeegers” from U.A. Emirates.

***rufipes*** Zeegers, 2007.—Afrotropical: Yemen.

*Cestonia rufipes* Zeegers, 2007: 382. Holotype male (RMNH). Type locality: Yemen, Al Kawd [as “Al Kowd”] (15°14′52″N 43°15′16″E).

***rutilans*** Villeneuve, 1929.—Afrotropical: Senegal, Yemen. Palaearctic: N. Africa (NE. Africa).

*Cestonia rutilans* Villeneuve, 1929a: 102. Syntypes, 1 male and 1 female (not located). Type locality: Egypt, Al Qāhirah [as “Caire”].

##### Genus *CESTONIONERVA* Villeneuve, 1929

***CESTONIONERVA*** Villeneuve, 1929b: 43. Type species: *Conogaster petiolata* Villeneuve, 1910, by subsequent designation of Townsend (1936b: 137).

Note: *Cestonionerva* Villeneuve, 1929 was “Formé pour *Conogaster petiolata* Villen.” (Villeneuve 1929b: 43) and a new species of the genus was also described in the same paper. Crosskey (1980b: 876) and Herting and Dely-Draskovits (1993: 223) interpreted *Conogaster petiolata* as the type species of *Cestonionerva* by original designation. However, a fixation by original designation requires an explicit designation of a type species (Article 68.2 of the *Code*, ICZN 1999), which is lacking in this instance. The type species of *Cestonionerva* Villeneuve, 1929 was therefore fixed later by the subsequent designation of Townsend (1936b: 137).

***petiolata*** (Villeneuve, 1910).—Afrotropical: U.A. Emirates, Yemen. Palaearctic: C. Asia, M. East (Israel), Mongolia, N. Africa (Canary Is., NE. Africa), Pal. China.

*Conogaster petiolata* Villeneuve *in* Becker, 1910b: 144 [also 1910b: 14]. Holotype female (NHMW). Type locality: Yemen, Suquţrá [as “Sokótra”].

##### Genus *CHRYSERYCIA* Mesnil, 1977

***CHRYSERYCIA*** Mesnil, 1977b: 185. Type species: *Chryserycia fulviceps* Mesnil, 1977, by original designation.

***fulviceps*** Mesnil, 1977.—Afrotropical: Madagascar.

*Chryserycia fulviceps* Mesnil, 1977b: 186. Holotype female (MNHN). Type locality: Madagascar, Antsiranana, Montagne d’Ambre [Parc National, ca. 12°36′S 49°8′E].

##### Genus *DESCAMPSINA* Mesnil, 1956

***DESCAMPSINA*** Mesnil, 1956b: 76. Type species: *Descampsina sesamiae* Mesnil, 1956, by original designation.

***sesamiae*** Mesnil, 1956.—Afrotropical: Cameroon, D.R. Congo (**new record**, IRSNB [PC]), Nigeria.

*Descampsina sesamiae* Mesnil, 1956b: 76. Holotype, unspecified sex [male, examined by PC] (MNHN). Type locality: Cameroon, Garoua.

Note: Mesnil (1956b: 76–77) described *Descampsina sesamiae* from both sexes from Garoua (Cameroon) and wrote “Type dans ma collection”, without giving the sex. O’Hara (1996: 156) treated the type series as comprising syntypes in CNC and MNHN but this is incorrect because a holotype (as “Type”) was designated in the original description.

##### Genus *DIAPROCHAETA* Mesnil, 1970

***DIAPROCHAETA*** Mesnil, 1970b: 103. Type species: Diaprochaeta (Diaprochaeta) illustris Mesnil, 1970, by original designation.

***illustris*** Mesnil, 1970.—Afrotropical: Zimbabwe.

Diaprochaeta (Diaprochaeta) illustris Mesnil, 1970b: 105. Holotype male (CNC). Type locality: Zimbabwe, “Sankishya” [not located].

##### Genus *DRINO* Robineau-Desvoidy, 1863

###### Subgenus *DRINO* Robineau-Desvoidy, 1863

***DRINO*** Robineau-Desvoidy, 1863a: 250. Type species: *Drino volucris* Robineau-Desvoidy, 1863 (= *Tachina lota* Meigen, 1824), by original designation [Palaearctic].

*STURMIODORIA* Townsend, 1928: 391. Type species: *Sturmiodoria facialis* Townsend, 1928, by original designation.

***cordata*** (Curran, 1927).—Afrotropical: Burundi, D.R. Congo, Guinea, Malawi, Rwanda.

*Sturmia cordata* Curran, 1927a: 12. Holotype male (AMNH). Type locality: D.R. Congo, Orientale, Kisangani [as “Stanleyville”].

Note: *Sturmia cordata* Curran, 1927 is moved here from Drino subgenus Palexorista Townsend, 1921 based on examination of the holotype by PC.

***facialis*** (Townsend, 1928).—Afrotropical: D.R. Congo. Palaearctic: Pal. China. Oriental: India, Indonesia, Malaysia, Orien. China, Philippines, Sri Lanka, Taiwan, Thailand.

*Sturmiodoria facialis* Townsend, 1928: 392. Holotype female (USNM). Type locality: Philippines, Basilan.

Note: *Sturmiodoria facialis* Townsend, 1928 was recorded from Africa (D.R. Congo) by Verbeke (1962b: 51) but Crosskey (1984: 284) commented that “confirmation of identity in Africa [is] needed”.

***lota*** (Meigen, 1824).—Afrotropical: Tanzania. Palaearctic: Europe (all except SW. Eur., Turkey), Japan, Pal. China [Ningxia], Russia (W. Russia, W. Siberia, S. Far East). Oriental: Orien. China.

*Tachina lota* Meigen, 1824: 326. Lectotype male (MNHN), by designation of Herting (1972: 9). Type locality: not given (Europe).

Note: *Tachina lota* Meigen, 1824 was recorded from Africa (Tanzania) by Mesnil (1959: 8) but Crosskey (1984: 284) commented that “confirmation of identity in Africa [is] needed”.

###### Subgenus *PALEXORISTA* Townsend, 1921

*PALEXORISTA* Townsend, 1921: 134. Type species: *Tachina succini* Giebel, 1862 (as “*Tichina succini* Giebel”), by monotypy.

*PROSTURMIA* Townsend, 1927c: 69. Type species: *Prosturmia profana* Townsend, 1927 (= *Masicera solennis* Walker, 1858), by original designation [Oriental].

*PROSTURMINA* Mesnil, 1949b: 103 (as subgenus of *Drino* Robineau-Desvoidy, 1863). *Nomen nudum* (proposed after 1930 without designation of type species; no included species).

*PROSTURMINA* Mesnil, 1949c: 8, 32 (as subgenus of *Drino* Robineau-Desvoidy, 1863). *Nomen nudum* (proposed after 1930 without type designation from three included species).

*PROSTURMINA* Mesnil, 1951: 161 (as subgenus of *Drino* Robineau-Desvoidy, 1863). *Nomen nudum* (proposed after 1930 without type designation; no included species).

*PROSTURMINA* Mesnil, 1970b: 110 (as subgenus of *Drino* Robineau-Desvoidy, 1863). Type species: *Sturmia vigilans* Villeneuve, 1933 (= *Sturmia pulchra* Curran, 1927), by original designation.

Note: The nomenclatural history of *Prosturmina* Mesnil was discussed by O’Hara (1996: 128) and Evenhuis and O’Hara (2008: 67).

***amicula*** Mesnil, 1949.—Afrotropical: Cameroon, Ghana, Mozambique, Nigeria, Senegal, Tanzania.

Drino (Prosturmia) amicula Mesnil, 1949c: 30. Syntypes, males and females (1 male in CNC, 2 males in MNHN). Type localities: Mozambique (Rio Zambeze [Tambara according to label data, Cooper and O’Hara 1996: 30; ca. 16°43′S 34°15′E]) and Senegal (Bambey).

***ampliceps*** (Karsch, 1886).—Afrotropical: Angola.

Masicera (Blepharipa) ampliceps Karsch, 1886b: 340. Holotype, unspecified sex [female, examined by JEOH] (ZMHB). Type locality: Angola, Pungo Andongo.

***aureocincta*** Mesnil, 1977.—Afrotropical: Madagascar.

Drino (Prosturmia) aureocincta Mesnil, 1977b: 179. Holotype male (MNHN). Type locality: Madagascar, Toliara, Sakaraha.

***aureola*** Mesnil, 1970.—Afrotropical: Sierra Leone.

Drino (Prosturmina) aureola Mesnil, 1970b: 110. Holotype male (CNC). Type locality: Sierra Leone, Bafodia [as “Bafodea”, ca. 9°41′N 11°43′E].

***aurifera*** (Villeneuve, 1943).—Afrotropical: D.R. Congo.

*Sturmia aurifera* Villeneuve, 1943a: 36. Syntypes, males and females (2 males in CNC). Type localities: D.R. Congo, Équateur, Eala and Maniema, Lubutu.

***crassiseta*** Mesnil, 1968.—Afrotropical: South Africa.

*Drino crassiseta* Mesnil, 1968b: 5. Holotype male (SMNS). Type locality: South Africa, Western Cape, Cape Town, Kirstenbosch.

*curvipalpis* (van der Wulp, 1893).—Misidentification, not Afrotropical [known from Palaearctic, Oriental and Australasian regions].

Note: An unknown species was recorded as “*Drino* (*Prosturmia* T.T.) *unisetosa* Bar.” (originally described as Sturmia (Sturmia) unisetosa Baranov, 1932, currently a synonym of *Drino curvipalpis* (van der Wulp, 1893)) from Tanzania by Mesnil (1959: 7). Misidentification (Crosskey 1980b: 872).

***flavicans*** (Wiedemann, 1819).—Afrotropical: D.R. Congo, Malawi, South Africa, Uganda.

*Tachina flavicans* Wiedemann, 1819: 24. Type(s), female (not located). Type locality: South Africa, Western Cape, Cape of Good Hope [as “Prom. bon. sp.” = “Promontorium Bonae Spei”].

*Sturmia congolensis* Villeneuve, 1910a: 253. Syntypes, 3 females (not located). Type locality: D.R. Congo (as “Congo”, p. 249).

Note: Villeneuve (1910a) described four species from “Congo”. Curran (1927f: 122) treated one of them (*Sturmia aureiventris* Villeneuve, 1910) as described from D.R. Congo (as “Belgian Congo”), and used “Belgian Congo” and “Congo” interchangeably in this work and some others. We think it likely that Villeneuve (1910a), like Curran, used “Congo” in the sense of present-day D.R. Congo. However, Crosskey (1980b) interpreted Villeneuve’s Congo as the present-day country of Congo.

***flaviseta*** (Thomson, 1869).—Afrotropical: Mauritius.

*Masicera flaviseta* Thomson, 1869: 522. Type(s), unspecified sex (NHRS). Type locality: Mauritius.

*gilva* (Hartig, 1838).—Misidentification, not Afrotropical [known from Palaearctic Region].

Note: An unknown species was recorded as “*Sturmia gilva* Hartig” (originally described as *Tachina gilva* Hartig, 1838) from D.R. Congo by Curran (1927f: 116, 1928b: 393). Misidentifications (not recorded from the Afrotropical Region by Herting and Dely-Draskovits 1993: 207).

***gilvoides*** (Curran, 1927).—Afrotropical: D.R. Congo, South Africa.

*Sturmia gilvoides* Curran, 1927f: 117. Holotype male (SANC). Type locality: South Africa, Mpumalanga, Barberton.

***idonea*** (Brauer & Bergenstamm, 1891).—Afrotropical: ?Eritrea, Mozambique, South Africa.

*Argyrophylax idonea* Brauer & Bergenstamm, 1891: 344 [also 1891: 40]. Type(s), male (NHMW, not located by JEOH). Type locality: South Africa, Western Cape, Cape of Good Hope [as “Cap b. sp.” = “Cap Bonae Spei”].

*Sturmia partitor* Curran, 1927f: 116. Holotype male (SANC). Type locality: South Africa, Free State, Bloemfontein.

Note: Bezzi’s (1908b: 30) record of *Drino idonea* (Brauer & Bergenstamm, 1891) (as Sturmia (Argyrophylax) idonea) from Eritrea needs confirmation.

***imberbis*** (Wiedemann, 1830).—Afrotropical: D.R. Congo, Kenya, Malawi, South Africa, Sudan, Tanzania, U.A. Emirates, Uganda, Yemen. Palaearctic: C. Asia, Europe (SC. Eur., Turkey), M. East (all), N. Africa (Canary Is., NE. Africa), Transcaucasia.

*Tachina imberbis* Wiedemann, 1830: 317. Syntypes, 2 or more males (lost, Crosskey 1967b: 93, Ziegler 2011: 8). Type locality: Egypt.

*Sturmia zonata* Curran, 1927d: 336. Holotype male (BMNH). Type locality: Uganda, Entebbe.

Note: See Herting (1984: 193, note 142) and Ziegler (2011: 7–8) for a discussion of the identities of *Tachina imberbis* Wiedemann, 1830, *Sturmia zonata* Curran, 1927, and *Phorcida latigena* Mesnil, 1944. Zeegers (2007: 386, 2010: 679) treated *Tachina imberbis* as a *nomen dubium* and used *Sturmia zonata* as the valid name for this taxon.

*inconspicua* (Meigen, 1830).—Misidentification, not Afrotropical [known from Palaearctic and Oriental regions].

Note: An unknown species was recorded as “Sturmia (Sturmia) inconspicua” (originally described as *Tachina inconspicua* Meigen, 1830) from Tanzania by Speiser (1910: 140). The same or a similar species was recorded from Malawi and Uganda by Villeneuve (1913c: 29, as “*Sturmia inconspicua*”), from D.R. Congo and South Africa by Curran (1927f: 118, 1928b: 393, as “*Sturmia inconspicua*”) and from Seychelles by Barraclough (2009: 304, as “*Drino inconspicua*” but noting “Confirmation of identity required”). It was also recorded as “*Sturmia bimaculata*” (originally described as *Tachina bimaculata* Hartig, 1838, currently a synonym of *Drino inconspicua* (Meigen)) from D.R. Congo by Curran (1927f: 118, 1928b: 394). Misidentifications (Crosskey 1980b: 871, 872).

***iterata*** Mesnil, 1949.—Afrotropical: South Africa, Uganda.

Drino (Prosturmia) iterata Mesnil, 1949c: 31. Syntypes, males and females (1 male in CNC). Type localities: Uganda and South Africa.

***latigena*** (Mesnil, 1944).—Afrotropical: Djibouti, U.A. Emirates. Palaearctic: M. East (Israel), N. Africa (NE. Africa).

*Phorcida latigena* Mesnil, 1944: 15. Holotype male (MNHN). Type locality: Djibouti, Obock [as “Obok”].

*Tachina imberbis* of authors (e.g., Crosskey 1967b: 93–94, 1980b: 872 [in part], as “*Palexorista imberbis*”), not Wiedemann, 1830. Misidentification (Herting 1984: 193, note 142; Ziegler 2011: 7–8).

***lavinia*** (Curran, 1927).—Afrotropical: D.R. Congo, Uganda.

*Sturmia lavinia* Curran, 1927c: 14. Holotype male (AMNH). Type locality: D.R. Congo, Orientale, Kisangani [as “Stanleyville”].

***laxa*** (Curran, 1927).—Afrotropical: Botswana, Kenya, Malawi, South Africa, Sudan, Swaziland, Tanzania, Uganda, Zimbabwe. Oriental: India.

*Sturmia laxa* Curran, 1927d: 335. Holotype male (BMNH). Type locality: Tanzania, Morogoro.

***mayneana*** (Villeneuve, 1930).—Afrotropical: D.R. Congo.

*Sturmia mayneana* Villeneuve, 1930b: 59. Syntypes, males and females (“plusieurs individus”) (MRAC). Type locality: D.R. Congo, Équateur, Eala.

***melancholica*** Mesnil, 1949.—Afrotropical: Zimbabwe.

Drino (Prosturmia) melancholica Mesnil, 1949c: 16. Syntypes, 1 male and 1 female (CNC). Type locality: Zimbabwe, Harare [as “Salisbury”].

***nova*** Mesnil, 1949.—Afrotropical: Madagascar.

Drino (Prosturmia) nova Mesnil, 1949c: 27. Syntypes, males and females (1 male in CNC, MNHN). Type locality: Madagascar, Toliara, Bekily.

***obliterata*** Mesnil, 1949.—Afrotropical: Malawi, Senegal, South Africa.

Drino (Prosturmia) patruelis obliterata Mesnil, 1949c: 18. Syntypes, males and females (MNHN). Type localities: Malawi (Mt. Mulanje [as “Mt. Mlanje”]), Senegal (Bambey), and South Africa.

***parachrysops*** (Bezzi, 1925).—Afrotropical: Ghana, Kenya, Mali, Nigeria, Senegal, Yemen. Palaearctic: M. East (M. East [Saudi Arabia, Dawah 2011: 5]). Oriental: India, ?Indonesia, Malaysia, Sri Lanka.

*Sturmia parachrysops* Bezzi, 1925b: 114. Lectotype male (BMNH), by designation of Crosskey (1967b: 78). Type locality: Malaysia, Peninsular Malaysia, Kuala Lumpar.

***patruelis*** Mesnil, 1949.—Afrotropical: Malawi, South Africa, Tanzania, Uganda, Zimbabwe.

Drino (Prosturmia) patruelis Mesnil, 1949c: 17. Syntypes, males and probably females (“nombreux exemplaires”) (1 male and possibly other syntypes in CNC). Type localities: South Africa and Zimbabwe (Harare [as “Salisbury”]).

***pulchra*** (Curran, 1927).—Afrotropical: D.R. Congo, Uganda.

*Sturmia pulchra* Curran, 1927a: 16. Holotype male (BMNH). Type locality: Uganda, Entebbe.

*Sturmia vigilans* Villeneuve, 1933: 278. Holotype female (MRAC). Type locality: D.R. Congo, Équateur, Eala.

***quadrizonula*** (Thomson, 1869).—Afrotropical: widespread, including D.R. Congo, Ghana, Kenya, Saint Helena, São Tomé & Principe, Senegal, Seychelles, South Africa, Tanzania, Uganda, Zimbabwe (Crosskey 1977: 152, in part).

*Masicera quadrizonula* Thomson, 1869: 521. Lectotype female (NHRS), by designation of Crosskey (1970: 580). Type locality: Saint Helena.

Note: *Masicera quadrizonula* Thomson, 1869 was redescribed by Crosskey (1970: 580, 1977: 151).

***rufa*** Zeegers, 2007.—Afrotropical: Yemen.

*Drino rufa* Zeegers, 2007: 385. Holotype male (RMNH). Type locality: Yemen, Sana’a (15°21′17″N 44°12′24″E).

***salva*** (Wiedemann, 1830).—Afrotropical: South Africa.

*Tachina salva* Wiedemann, 1830: 340. Type(s), female (1 syntype in ZMUC, Zimsen 1954: 23). Type locality: South Africa [as “China”, in error according to Crosskey 1980b: 872].

***subaurata*** (Walker, 1853).—Afrotropical: Madagascar, South Africa.

*Tachina subaurata* Walker, 1853: 298. Type(s) female (BMNH). Type locality: South Africa, Western Cape, Cape of Good Hope [as “Cape”].

***succini*** (Giebel, 1862).—Afrotropical: ?Tanzania.

*Tachina succini* Giebel, 1862: 319. Holotype female (NMCL). Type locality: not given (in copal; “East Africa presumed”, Crosskey 1980b: 872).

Note: *Tachina succini* Giebel, 1862 was described from a copal inclusion originally thought to be an amber fossil (Crosskey 1966c: 133). Its provenance is unknown but likely East Africa, particularly Zanzibar, a popular source of copal since the early 1800s.

***tenella*** (Bezzi, 1911).—Afrotropical: South Africa.

Erycia (Bactromyia) tenella Bezzi, 1911: 60. Holotype female (USNM). Type locality: South Africa, Gauteng, Pretoria.

***terrosa*** Mesnil, 1949.—Afrotropical: Madagascar.

Drino (Prosturmia) terrosa Mesnil, 1949c: 20. Type(s), unspecified sex (MNHN). Type locality: Madagascar, Toliara, Bekily.

***ugandana*** (Curran, 1927).—Afrotropical: Burundi, D.R. Congo, Malawi, South Africa, Uganda, Zimbabwe.

*Sturmia ugandana* Curran, 1927c: 16. Holotype male (AMNH; not BMNH, see Arnaud and Owen 1981: 239). Type locality: Uganda, Entebbe.

###### Subgenus *ZYGOBOTHRIA* Mik, 1891

*ZYGOBOTHRIA* Mik, 1891: 193. Type species: *Sturmia atropivora* Robineau-Desvoidy, 1830, by original designation.

*FORMOSODORIA* Townsend, 1933: 475. Type species: Sturmia (Argyrophylax) dilabida Villeneuve, 1916 (= *Meigenia ciliata* van der Wulp, 1881), by original designation.

***atropivora*** (Robineau-Desvoidy, 1830).—Afrotropical: “widespread Afrotrop Reg.” (Crosskey 1980b: 874), including D.R. Congo, Ghana, Kenya, Madagascar, Malawi, Mauritius, Mozambique, Namibia, Sierra Leone, South Africa, Tanzania, U.A. Emirates, Uganda. Palaearctic: C. Asia, Europe (all except British Is., Scand.), Japan, M. East (all), N. Africa (Canary Is., NW. Africa), Pal. China, Russia (W. Russia), Transcaucasia. Oriental: India, Indonesia, Laos, Malaysia, Orien. China, Ryukyu Is., Sri Lanka. Australasian: Australia.

*Sturmia atropivora* Robineau-Desvoidy, 1830: 171. Syntypes, more than 80 males and females (lost, Herting 1974a: 24). Type locality: not given (France).

*Sturmia masakensis* Curran, 1927f: 117. Holotype male (BMNH). Type locality: Uganda, Masaka.

*masakesnsis*. Incorrect subsequent spelling of *masakensis* Curran, 1927 (Curran 1928b: 388).

***ciliata*** (van der Wulp, 1881).—Afrotropical: “widespread mainland Afrotrop. Reg.” (Crosskey 1980b: 874), including Ghana, Malawi, South Africa, U.A. Emirates, Uganda. Palaearctic: Pal. China. Oriental: India, Indonesia, Sri Lanka, Taiwan. Australasian: Australia, N. Australasian.

*Meigenia ciliata* van der Wulp, 1881: 38. Lectotype male (RMNH), by designation of Crosskey (1967c: 104). Type locality: Indonesia, Sumatera, Alahanpanjang [as “Alahan pandjang”].

Sturmia (Argyrophylax) dilabida Villeneuve, 1916c: 479. Type(s), unspecified number and including at least 1 male (SAMC, not located by JEOH). Type locality: South Africa, KwaZulu-Natal, Durban.

Note: *Sturmia dilabida* Villeneuve, 1916 was described from one or more specimens, at least one of which was male. The type locality was given as Durban and the depository as SAMC. There are several specimens in SAMC identified by Villeneuve as *Sturmia dilabida* but none from Durban. Unless type material of *Sturmia dilabida* is discovered in SAMC or is proven to have existed there, Townsend’s (1941: 270) mention of “Ht male” from Durban in SAMC cannot be accepted as a lectotype fixation. The identity of *Sturmia dilabida* was confused with that of *Sturmia convergens* (Wiedemann, 1824) by Villeneuve (1933: 280). Townsend (1932: 32, 1933: 475) erred in citing the type locality of *Sturmia dilabida* as Taiwan [as “Formosa”] and the type depository as SDEI [as “Berlin-Dahlem”]. Later, Townsend (1941: 270) correctly cited the type locality as Durban.

*Sturmia munroi* Curran, 1927c: 17. Holotype male (SANC). Type locality: South Africa, Eastern Cape, East London.

Sturmia (Sturmia) macrophallus Baranov, 1932: 76. Lectotype male (SDEI), by designation of Crosskey (1967c: 105). Type locality: Taiwan, P’ingtung Hsien, Changkou [as “Kankau”, near Hengch’un].

*Formosodoria foeda* Villeneuve, 1933: 280 (as “*Formosodoria foeda* T. T.”). *Nomen nudum* (proposed in synonymy [with *Sturmia dilabida* Villeneuve, 1916 and *Tachina convergens* Wiedemann, 1824, the latter misidentified] and not made available by subsequent usage before 1961).

*Tachina convergens* of Mesnil (1951: 169, as “*Drino convergens*”), not Wiedemann, 1824. Misidentification (Crosskey 1963: 77, 1980b: 874).

***grandicornis*** Mesnil, 1977.—Afrotropical: Madagascar.

Drino (Zygobothria) grandicornis Mesnil, 1977b: 178. Holotype male (MNHN). Type locality: Madagascar, Fianarantsoa, Mananjary.

##### Genus *EUGAEDIOXENIS* Cerretti, O’Hara & Stireman, 2015

***EUGAEDIOXENIS*** Cerretti, O’Hara & Stireman *in* Cerretti et al., 2015: 494. Type species: *Gaedioxenis haematodes* Villeneuve, 1937, by original designation.

***haematodes*** (Villeneuve, 1937).—Afrotropical: South Africa.

*Gaedioxenis haematodes* Villeneuve, 1937a: 207. Holotype male (CNC). Type locality: South Africa, “Colonie du Cap” ([former Cape Province], between Somerset West and Strand according to label data, Cooper and O’Hara 1996: 39).

***horridus*** Cerretti, O’Hara & Stireman, 2015.—Afrotropical: South Africa.

*Eugaedioxenis horridus* Cerretti, O’Hara & Stireman *in* Cerretti et al., 2015: 501. Holotype male (MZUR). Type locality: South Africa, Western Cape, Anysberg Nature Reserve, 840m (33°26′37.76″S 20°47′29.25″E).

##### Genus *HYPERSARA* Villeneuve, 1935

***HYPERSARA*** Villeneuve, 1935a: 139. Type species: *Hypersara argentata* Villeneuve, 1935, by monotypy.

***argentata*** Villeneuve, 1935.—Afrotropical: D.R. Congo, Nigeria.

*Hypersara argentata* Villeneuve, 1935a: 140. Holotype male (not located). Type locality: D.R. Congo, Nord-Kivu, Walikale [ca. 1°25′S 28°00′E].

Undescribed sp.: Ethiopia (TAU, examined by PC).

##### Genus *INTRAPALES* Villeneuve, 1938

***INTRAPALES*** Villeneuve, 1938c: 8. Type species: *Intrapales remotella* Villeneuve, 1938, by monotypy.

***hirsuta*** Mesnil, 1977.—Afrotropical: Madagascar.

*Intrapales hirsuta* Mesnil, 1977b: 185. Holotype male (MNHN). Type locality: Madagascar, Toamasina, Périnet [ca. 18°55′S 48°25′E].

***insularis*** Mesnil, 1977.—Afrotropical: Madagascar.

*Intrapales insularis* Mesnil, 1977b: 184. Holotype male (MNHN). Type locality: Madagascar, Fianarantsoa, Anosimparihy [ca. 21°30′S 47°59′E].

***remotella*** Villeneuve, 1938.—Afrotropical: D.R. Congo, Nigeria (**new record**, CNC), Sierra Leone, Tanzania.

*Intrapales remotella* Villeneuve, 1938c: 8. Syntypes, 2 males and 1 female (IRSNB). Type locality: D.R. Congo, Équateur, Eala.

##### Genus *KAISERIOLA* Mesnil, 1970

***KAISERIOLA*** Mesnil, 1970b: 105 (as subgenus of *Diaprochaeta* Mesnil, 1970). Type species: Diaprochaeta (Kaiseriola) aperta Mesnil, 1970, by original designation.

Note: *Kaiseriola* Mesnil, 1970 was treated as a synonym of *Diaprochaeta* Mesnil, 1970 by Crosskey (1980b: 877) but was later recognized as a genus by Crosskey (1984: 201, 294).

***aperta*** (Mesnil, 1970).—Afrotropical: Mozambique (**new record**, JOS [PC]), South Africa.

Diaprochaeta (Kaiseriola) aperta Mesnil, 1970b: 105. Holotype male (CNC). Type locality: South Africa, KwaZulu-Natal, Durban.

***obscura*** (Mesnil, 1970).—Afrotropical: Madagascar.

Diaprochaeta (Kaiseriola) obscura Mesnil, 1970b: 106. Holotype male (MNHN). Type locality: Madagascar, Toamasina, Moramanga.

##### Genus *LUBUTANA* Villeneuve, 1938

***LUBUTANA*** Villeneuve, 1938c: 10. Type species: *Lubutana divaricata* Villeneuve, 1938, by original designation.

***divaricata*** Villeneuve, 1938.—Afrotropical: D.R. Congo, Ethiopia, Ghana, Malawi, Nigeria, Sierra Leone, Uganda.

*Lubutana divaricata* Villeneuve, 1938c: 10. Syntypes, males (IRSNB). Type localities: D.R. Congo (Maniema, Lubutu; Nord-Kivu, Walikale [ca. 1°25′S 28°00′E]), Malawi (Mt. Mulanje [as “Mont Mlanje”]) and Nigeria (Degema).

***mayeri*** Mesnil, 1955.—Afrotropical: Nigeria.

*Lubutana mayeri* Mesnil, 1955: 363. Holotype female (CNC). Type locality: Nigeria, Oshogbo.

***perplexa*** Mesnil, 1955.—Afrotropical: D.R. Congo, Rwanda, Uganda.

*Lubutana perplexa* Mesnil, 1955: 362. Holotype female (MRAC). Type locality: Rwanda, eastern foothills of Volcan Muhabura [as “Muhavura”], 2100m [ca. 1°23′S 29°44′E].

##### Genus *LYDELLA* Robineau-Desvoidy, 1830

***LYDELLA*** Robineau-Desvoidy, 1830: 112. Type species: *Lydella grisescens* Robineau-Desvoidy, 1830, by subsequent designation of Robineau-Desvoidy (1863a: 855) [Palaearctic].

*METOPOSISYROPS* Townsend, 1916d: 320. Type species: *Metoposisyrops oryzae* Townsend, 1916, by original designation [Oriental].

Note: *Metoposisyrops* Townsend, 1916 was synonymized with *Lydella* Robineau-Desvoidy, 1830 by Woodley (1994: 135).

***sesamiae*** (Mesnil, 1968).—Afrotropical: D.R. Congo (**new record**, IRSNB [PC]), Mozambique (**new record**, MZUR [PC]), Namibia (**new record**, MZUR [PC]), Nigeria, Uganda.

*Metagonistylum sesamiae* Mesnil, 1968b: 4. Holotype female (CNC). Type locality: Uganda, Kidetok, Serere [ca. 1°30′N 33°33′E].

##### Genus *MADREMYIA* Townsend, 1916

***MADREMYIA*** Townsend, 1916d: 305. Type species: *Madremyia parva* Townsend, 1916 (= *Phorocera saundersii* Williston, 1889), by original designation [Neotropical]. **New record.**

Note: *Madremyia* Townsend, 1916 is newly recorded from the Afrotropical Region for a species previously placed in *Phryxe* Robineau-Desvoidy, 1830.

***setinervis*** (Mesnil, 1968).—Afrotropical: Tanzania. **Comb. n.**

*Phryxe setinervis* Mesnil, 1968b: 5. Holotype female (SMNS). Type locality: Tanzania, southwest side of Mt. Kilimanjaro [as “Kilimandjaro”], 3500m.

Note: *Phryxe setinervis* Mesnil, 1968 was treated as a species of *Phryxe* Robineau-Desvoidy, 1830 by Crosskey (1980b: 879) but is moved here to *Madremyia* Townsend, 1916.

##### Genus *MYXARCHICLOPS* Villeneuve, 1916

***MYXARCHICLOPS*** Villeneuve, 1916c: 494. Type species: *Myxarchiclops caffer* Villeneuve, 1916, by subsequent designation of Townsend (1936b: 222).

***caffer*** Villeneuve, 1916.—Afrotropical: South Africa.

*Myxarchiclops caffer* Villeneuve, 1916c: 495. Lectotype male (CNC), by designation herein (see Lectotype Designations section). Type locality: South Africa, Western Cape, Cape Town.

***major*** Villeneuve, 1930.—Afrotropical: South Africa.

*Myxarchiclops major* Villeneuve, 1930a: 353. Syntypes, 2 females (CNC). Type locality: South Africa, “Colonie du Cap” ([former Cape Province], Somerset West according to label data, Cooper and O’Hara 1996: 54).

##### Genus *NEOLYDELLA* Mesnil, 1939

***NEOLYDELLA*** Mesnil, 1939a: 209 (as subgenus of *Lydella* Robineau-Desvoidy, 1830). Type species: Lydella (Neolydella) pruinosa Mesnil, 1939, by monotypy.

***pruinosa*** (Mesnil, 1939).—Afrotropical: Madagascar.

Lydella (Neolydella) pruinosa Mesnil, 1939a: 209. Syntypes, 3 males (MNHN). Type locality: Madagascar, Toliara, Bekily, “région sud de l’Ile”.

##### Genus *NILEA* Robineau-Desvoidy, 1863

***NILEA*** Robineau-Desvoidy, 1863a: 275. Type species: *Nilea innoxia* Robineau-Desvoidy, 1863, by original designation [Palaearctic].

***longicauda*** (Mesnil, 1970).—Afrotropical: Madagascar. **Comb. n.**

*Sturmia longicauda* Mesnil, 1970b: 91. Holotype male (MNHN). Type locality: Madagascar, Toamasina, Moramanga.

Note: *Sturmia longicauda* Mesnil, 1970 was treated as a species of *Sturmia* Robineau-Desvoidy, 1830 by Crosskey (1980b: 874) but is moved here to *Nilea* Robineau-Desvoidy, 1863.

***perplexa*** Mesnil, 1977.—Afrotropical: Burundi (**new record**, MZUR [PC]), Madagascar, Mozambique (**new record**, MZUR [PC]), South Africa (**new record**, NMDA [PC]).

*Nilea perplexa* Mesnil, 1977b: 188. Holotype male (MNHN). Type locality: Madagascar, Toamasina, Foulpointe [ca. 17°41′S 49°31′E].

Undescribed sp.: Tanzania (TAU, examined by PC).

##### Genus *PARADRINO* Mesnil, 1949

***PARADRINO*** Mesnil, 1949b: 103 (as subgenus of *Drino* Robineau-Desvoidy, 1863). Type species: *Sturmia halli* Curran, 1939 (as “*Paradrino Halli* Curr.”, p. 100), by monotypy (see Evenhuis and O’Hara 2008: 66).

***halli*** (Curran, 1939).—Afrotropical: Botswana, Tanzania, Uganda, Zimbabwe.

*Sturmia halli* Curran, 1939: 2. Holotype male (AMNH). Type locality: Zimbabwe, Kadoma [as “Gatooma”].

*Sturmia rhodesiensis* Jones, 1939: 16. Syntypes, males and females (BMNH). Type locality: Zimbabwe, Mazoe.

Note: Jones (1939: 15) wrote in a footnote on the first page of his paper: “After the present manuscript had been sent to the printers, Curran published a description of this species under the name of *Sturmia halli* sp. n. (1939, Amer. Mus. Nov. 1022, pp. 2–3).” Since the name *Sturmia rhodesiensis* was not explicitly proposed in synonymy with *Sturmia halli*, it is treated as both an available name and a subjective synonym of *Sturmia halli*.

Undescribed species of “?*Paradrino*”: Yemen (Zeegers 2007: 392).

##### Genus *PHRYXE* Robineau-Desvoidy, 1830

***PHRYXE*** Robineau-Desvoidy, 1830: 158. Type species: *Phryxe athaliae* Robineau-Desvoidy, 1830 (= *Tachina vulgaris* Fallén, 1810), by subsequent designation of Robineau-Desvoidy (1863a: 329, 358) (as *vulgaris*, with *athaliae* in synonymy) [Palaearctic].

Note: The single species recognized in *Phryxe* Robineau-Desvoidy, 1830 by Crosskey (1980b: 879), *Phryxe setinervis* Mesnil, 1968, is moved herein to *Madremyia* Townsend, 1916. We record *Phryxe* in the Afrotropical Region from an undescribed species.

Undescribed sp.: Ethiopia (TAU, examined by PC).

##### Genus *PSEUDOPERICHAETA* Brauer & Bergenstamm, 1889

***PSEUDOPERICHAETA*** Brauer & Bergenstamm, 1889: 92 [also 1890: 24]. Type species: *Pseudoperichaeta major* Brauer & Bergenstamm, 1889 (= *Phryxe palesioidea* Robineau-Desvoidy, 1830), by monotypy [Palaearctic].

*ACHAETONEURILLA* Mesnil, 1939a: 210 (as subgenus of *Pseudoperichaeta* Brauer & Bergenstamm, 1889). Type species: Pseudoperichaeta (Achaetoneurilla) madecassa Mesnil, 1939, by monotypy.

***laevis*** Villeneuve, 1932.—Afrotropical: Nigeria, Tanzania, Uganda, Zimbabwe.

*Pseudoperichaeta laevis* Villeneuve, 1932: 285. Syntypes, males and females (not located). Type locality: Zimbabwe, Harare [as “Salisbury”].

*Phorocera bolyodes* Curran, 1933: 166. Holotype female (BMNH). Type locality: Zimbabwe, Harare [as “Salisbury”].

*bothyodes*. Incorrect subsequent spelling of *bolyodes* Curran, 1933 (original usage not found but spelling listed by Crosskey 1980b: 880).

***leo*** (Curran, 1941).—Afrotropical: Zimbabwe.

*Phorocera leo* Curran, 1941: 10. Holotype female (AMNH). Type locality: Zimbabwe, Mutare [as “Umtali”].

*Pseudoperichaeta pilosa* Villeneuve, 1942a: 52. Syntypes, 2 males (1 male in CNC). Type locality: Zimbabwe, Hurungwe [as “Urungwe”], Gota Gota.

***madecassa*** Mesnil, 1939.—Afrotropical: Madagascar.

Pseudoperichaeta (Achaetoneurilla) madecassa Mesnil, 1939a: 210. Syntypes, 12 males and females (3 males and 2 females in CNC, MNHN). Type locality: Madagascar, Toliara, Bekily, “région sud de l’Ile”.

***nestor*** (Curran, 1927).—Afrotropical: D.R. Congo, Nigeria, Tanzania.

*Phorocera nestor* Curran, 1927c: 12. Holotype male (AMNH). Type locality: D.R. Congo, Orientale, Kisangani [as “Stanleyville”].

***pacta*** Villeneuve, 1932.—Afrotropical: D.R. Congo, Mauritius, South Africa, Zimbabwe.

*Pseudoperichaeta pacta* Villeneuve, 1932: 285. Holotype female (not located). Type locality: South Africa, Western Cape, “région de Cape-Town”.

***sallax*** (Curran, 1927).—Afrotropical: D.R. Congo.

*Phorocera sallax* Curran, 1927c: 11. Holotype male (AMNH). Type locality: D.R. Congo, Orientale, Kisangani [as “Stanleyville”].

##### Genus *PTILOCATAGONIA* Mesnil, 1956

***PTILOCATAGONIA*** Mesnil, 1956b: 79 (as subgenus of *Sisyropa* Brauer & Bergenstamm, 1889). Type species: Sisyropa (Ptilocatagonia) viridescens Mesnil, 1956, by monotypy.

***viridescens*** (Mesnil, 1956).—Afrotropical: Sierra Leone, Tanzania, Zambia.

Sisyropa (Ptilocatagonia) viridescens Mesnil, 1956b: 79. Holotype male (SMNS). Type locality: Tanzania, Msingi [ca. 4°20′S 34°34′E].

Note: Mesnil (1956b: 79) described *Sisyropa viridescens* from a “Mâle capturé à Msingi (Ruwenzori)”. The type locality of Msingi is in Tanzania, whereas “Ruwenzori” refers to the Rwenzori Range on the border between D.R. Congo and Uganda. The country of the type locality was incorrectly cited as Uganda by Crosskey (1980b: 873) and O’Hara (1996: 160).

##### Genus *SENOMETOPIA* Macquart, 1834

***SENOMETOPIA*** Macquart, 1834: 160 [also 1834: 296]. Type species: *Carcelia aurifrons* Robineau-Desvoidy, 1830 (= *Tachina excisa* Fallén, 1820), by subsequent designation of Townsend (1916b: 8) (earlier type fixations set aside by ICZN 2012: 242; see Evenhuis and Thompson 1990: 237 and O’Hara and Evenhuis 2011: 61) [Palaearctic].

*STENOMETOPIA* Agassiz, 1846b: 351. Unjustified emendation of *Senometopia* Macquart, 1834.

*EOCARCELIA* Townsend, 1919b: 582. Type species: *Eocarcelia ceylanica* Townsend, 1919, by original designation [Oriental].

*EOCARCELIOPSIS* Townsend, 1928: 392. Type species: *Eocarceliopsis bakeri* Townsend, 1928, by original designation [Oriental].

*EUCARCELIA* Baranov, 1934: 393. Type species: *Tachina excisa* Fallén, 1820, by original designation [Palaearctic].

***albatella*** (Villeneuve, 1941).—Afrotropical: D.R. Congo, Malawi.

*Carcelia albatella* Villeneuve, 1941b: 125. Syntypes, 1 male and 1 female (MRAC). Type locality: D.R. Congo, Sud-Kivu, Kalembelembe to Baraka.

***evolans*** (Wiedemann, 1830).—Afrotropical: Côte d’Ivoire, Senegal, Sierra Leone, ?Yemen.

*Tachina evolans* Wiedemann, 1830: 321. Type(s), unspecified sex (not located). Type locality: Sierra Leone.

Note: *Tachina evolans* Wiedemann, 1830 has been misidentified from other places in the Afrotropical Region and from the Palaearctic Region, as noted by Crosskey (1980b: 865), Shima (2006: 64, 66) and O’Hara et al. (2009: 78). Given such a history of misidentifications, we treat the record from Yemen by Zeegers (2007: 396) as questionable. Curran (1927d: 327) examined the “type” of *Tachina evolans* but did not state where he had seen it or give any details about it.

***hectica*** (Speiser, 1910).—Afrotropical: Kenya, Tanzania, Uganda.

*Carcelia hectica* Speiser, 1910: 141. Holotype male (NHRS). Type locality: Tanzania, Mt. Kilimanjaro [as “Kilimandjaro”], valley at Kibongoto [as “Kibonoto”].

***illota*** (Curran, 1927).—Afrotropical: Nigeria, South Africa, Tanzania. Oriental: India, Laos, Orien. China. Australasian: Australia.

*Zenillia illota* Curran, 1927d: 328. Holotype male (BMNH). Type locality: Tanzania, Morogoro.

***judicabilis*** (Mesnil, 1949).—Afrotropical: D.R. Congo, Malawi, Zimbabwe.

Carcelia (Eucarcelia) evolans judicabilis Mesnil, 1949a: 90. Holotype, unspecified sex [male, examined by PC] (MRAC). Type locality: D.R. Congo, Katanga, Lubumbashi [as “Elisabethville”].

***laetifica*** (Mesnil, 1949).—Afrotropical: D.R. Congo, Ghana, Nigeria.

Carcelia (Eucarcelia) evolans laetifica Mesnil, 1949a: 89. Holotype male (MRAC). Type locality: D.R. Congo, Katanga, Lubumbashi [as “Elisabethville”].

***norma*** (Curran, 1927).—Afrotropical: Malawi, Tanzania, Uganda.

*Zenillia norma* Curran, 1927d: 329. Holotype male (BMNH). Type locality: Uganda, Bugoma Forest [ca. 1°16′N 30°57′E].

##### Genus *SISYROPA* Brauer & Bergenstamm, 1889

***SISYROPA*** Brauer & Bergenstamm, 1889: 163 [also 1890: 95]. Type species: *Tachina thermophila* Wiedemann, 1830, by monotypy [Oriental].

*STYLURODORIA* Townsend, 1933: 476. Type species: *Stylurodoria stylata* Townsend, 1933, by original designation.

*CTENOPHOROCEROPSIS* Baranov, 1938: 408. Type species: *Ctenophoroceropsis yerburyi* Baranov, 1938, by original designation.

*POUJADEA* Mesnil, 1949b: 102. *Nomen nudum* (proposed after 1930 without designation of type species; no included species) (see Evenhuis and O’Hara 2008: 67).

*EOCATAGONIA* Mesnil, 1949b: 103 (as subgenus *Sisyropa* Brauer & Bergenstamm, 1889). *Nomen nudum* (proposed after 1930 without designation of type species; no included species) (see Evenhuis and O’Hara 2008: 66).

*POUJADEA* Mesnil, 1950c: 108. Type species: *Zenillia insolita* Curran, 1927, by monotypy (see Evenhuis and O’Hara 2008: 67).

*EOCATAGONIA* Mesnil, 1950c: 148 (as subgenus *Sisyropa* Brauer & Bergenstamm, 1889). Type species: Sisyropa (Eocatagonia) argyrata Mesnil, 1950, by monotypy (see Evenhuis and O’Hara 2008: 66).

***argyrata*** Mesnil, 1950.—Afrotropical: Senegal.

Sisyropa (Eocatagonia) argyrata Mesnil, 1950c: 148. Holotype male (MNHN). Type locality: Senegal.

***boveyi*** Mesnil, 1958.—Afrotropical: Ghana, Guinea, Kenya, Nigeria, Tanzania.

Sisyropa (Catagonia) boveyi Mesnil, 1958: 252. Holotype male (ETHZ). Type locality: Guinea, Réserve de la Biosphère des Monts Nimba [as “Réserve du Mt Nimba”], foot of Mont Nimba.

***insolita*** (Curran, 1927).—Afrotropical: D.R. Congo.

*Zenillia insolita* Curran, 1927c: 5. Holotype male (AMNH). Type locality: D.R. Congo, Orientale, Kisangani [as “Stanleyville”].

*insoleta*. Incorrect subsequent spelling of *insolita* Curran, 1927 (Curran 1927d: 327).

***madecassa*** Mesnil, 1944.—Afrotropical: Madagascar.

*Sisyropa formosa madecassa* Mesnil, 1944: 14. Holotype male (MNHN). Type locality: Madagascar, Fianarantsoa, Ikongo-Ankarimbelo region, Forêt Tanala.

***negator*** (Curran, 1927).—Afrotropical: D.R. Congo.

*Sturmia negator* Curran, 1927c: 15. Holotype male (AMNH). Type locality: D.R. Congo, Orientale, Kisangani [as “Stanleyville”].

*negastor*. Incorrect subsequent spelling of *negator* Curran, 1927 (Curran 1928b: 391).

***stylata*** (Townsend, 1933).—Afrotropical: Ghana, Mali, Nigeria, Sierra Leone, Sudan. Oriental: India, Sri Lanka, Taiwan.

*Stylurodoria stylata* Townsend, 1933: 476. Holotype female (SDEI). Type locality: Taiwan, P’ingtung Hsien, Changkou [as “Kankau”, near Hengch’un].

***subdistincta*** (Villeneuve, 1916).—Afrotropical: Côte d’Ivoire, Ethiopia, Ghana, Senegal, South Africa, Tanzania.

*Catagonia subdistincta* Villeneuve, 1916c: 484. Syntypes, 2 males (SAMC, not located by JEOH). Type locality: South Africa, KwaZulu-Natal, Durban.

*Sisyropa cinerosa* Mesnil, 1944: 15. Holotype male (MNHN). Type locality: Senegal, Bambey.

***yerburyi*** (Baranov, 1938).—Afrotropical: Yemen.

*Ctenophoroceropsis yerburyi* Baranov, 1938: 409. Holotype male (BMNH, Sabrosky and Crosskey 1969: 39). Type locality: Yemen, ‘Adan [as “Aden”].

*yerburi*. Incorrect subsequent spelling of *yerburyi* Baranov, 1938 (original usage not found but spelling listed by Crosskey 1980b: 873).

Possibly undescribed spp.: Nigeria (BMNH, Crosskey 1984: 281).

##### Genus *STURMIOPSIS* Townsend, 1916

***STURMIOPSIS*** Townsend, 1916d: 313. Type species: *Sturmiopsis inferens* Townsend, 1916, by original designation.

*RHODESINA* Curran, 1939: 3 (junior homonym of *Rhodesina* Malloch, 1921). Type species: *Rhodesina parasitica* Curran, 1939, by original designation.

*CURRANOMYIA* Townsend *in* Cuthbertson & Munro, 1941: 115 (*nomen novum* for *Rhodesina* Curran, 1939).

Note: Barraclough (2004) published a review of *Sturmiopsis* Townsend, 1916 but was apparently unaware of the key to species of *Sturmiopsis* and the description of *Sturmiopsis setifrons* Mesnil, 1977 by Mesnil (1977b: 186–187).

***inferens*** Townsend, 1916.—Afrotropical: Madagascar (probably introduced, Barraclough 2004: 12). Oriental: Bangladesh, Bhutan, India, Indonesia, Malaysia, Orien. China, Nepal, Philippines.

*Sturmiopsis inferens* Townsend, 1916d: 313. Holotype female (USNM). Type locality: Indonesia, Jawa, Bogor [as “Buitenzorg”].

***parasitica*** (Curran, 1939).—Afrotropical: Benin, Ghana, Kenya, Nigeria, Senegal, Tanzania, Zimbabwe. Oriental: India (introduced, Barraclough 2004: 17).

*Rhodesina parasitica* Curran, 1939: 3. Holotype male (AMNH). Type locality: Zimbabwe, Harare [as “Salisbury”].

*Sturmiopsis angustifrons* Mesnil, 1959: 11. Holotype male (SMNS). Type locality: Tanzania, Kisangara.

***setifrons*** Mesnil, 1977.—Afrotropical: Madagascar.

*Sturmiopsis setifrons* Mesnil, 1977b: 187. Holotype male (MNHN). Type locality: Madagascar, Fianarantsoa, Ambalavao.

##### Genus *STYLOCARCELIA* Zeegers, 2007

***STYLOCARCELIA*** Zeegers, 2007: 396. Type species: *Stylocarcelia stylata* Zeegers, 2007, by original designation.

***stylata*** Zeegers, 2007.—Afrotropical: Yemen.

*Stylocarcelia stylata* Zeegers, 2007: 396. Holotype male (RMNH). Type locality: Yemen, Sana’a (15°21′17″N 44°12′24″E).

##### Genus *THECOCARCELIA* Townsend, 1933

***THECOCARCELIA*** Townsend, 1933: 471. Type species: *Argyrophylax pelmatoprocta* Brauer & Bergenstamm, 1891 (= *Masicera acutangulata* Macquart, 1851), by original designation [Palaearctic].

*THELYCARCELIA* Townsend, 1933: 475. Type species: *Thelycarcelia thrix* Townsend, 1933 (= *Sturmia sumatrana* Baranov, 1932), by original designation [Oriental].

Note: The relative priority of *Thecocarcelia* Townsend, 1933 and *Thelycarcelia* Townsend, 1933, when the two are treated as synonyms, was established by Mesnil (1950b: 20), as the First Reviser (Article 24.2.2 of the *Code*, ICZN 1999).

***acutangulata*** (Macquart, 1851).—Afrotropical: “W. Afr. to E. Afr. & sthn Afr.” (Crosskey 1980b: 866), including D.R. Congo, Madagascar. Palaearctic: Europe (all except Scand., Turkey), Japan, Transcaucasia.

*Masicera acutangulata* Macquart, 1851a: 478. Type(s), female (MHNL or lost). Type locality: Switzerland, Chur [as “Coire”].

*Masicera incedens* Rondani, 1861b: 22. Type(s), female (MZF, Herting 1969: 195; 1 female syntype and 1 male non-type in MZF [examined by PC]). Type locality: Italy, plain near Parma.

*Argyrophylax pelmatoprocta* Brauer & Bergenstamm, 1891: 344 [also 1891: 40]. Syntypes, males and females (2 males and 4 females in NHMW). Type locality: “M.-Europa”.

Note: *Argyrophylax pelmatoprocta* Brauer & Bergenstamm, 1891 was described from an unspecified number of males and females from “M.-Europa”. Herting (1974b: 140) reported on a female syntype from Bisamberg [near Wien, Austria] in NHMW but this collection includes an additional two males and three females identified as *pelmatoprocta* by “B. B.” or “Bergenst.” from other localities in Europe and these specimens are considered syntypes as well (examined by JEOH).

***ebenina*** Mesnil, 1950.—Afrotropical: D.R. Congo, South Africa.

*Thecocarcelia ebenina* Mesnil, 1950b: 21. Syntypes, males and possibly females (not located). Type locality: South Africa, KwaZulu-Natal.

***flavicosta*** Zeegers, 2007.—Afrotropical: Yemen.

*Thecocarcelia flavicosta* Zeegers, 2007: 398. Holotype male (RMNH). Type locality: Yemen, Laḩij [as “Lahj”] (13°03′28″N 44°53′02″E).

***latifrons*** Mesnil, 1949.—Afrotropical: Mozambique, South Africa, Uganda, Zimbabwe.

*Thecocarcelia latifrons* Mesnil, 1949b: 56. Holotype male (CNC). Type locality: Mozambique, Rio Zambezi, near Chemba, “Nova Choupanga”.

Note: Zeegers (2010: 681) recognized “Thecocarcelia cf. latifrons Mesnil” from U.A. Emirates.

***latimana*** Mesnil, 1950.—Afrotropical: South Africa.

*Thecocarcelia latimana* Mesnil, 1950b: 22. Syntypes, males and females (not located). Type locality: South Africa.

***pauciseta*** Mesnil, 1977.—Afrotropical: Madagascar.

*Thecocarcelia pauciseta* Mesnil, 1977b: 181. Holotype male (NHMB [“to be returned to MNHN”, O’Hara 1996: 152]). Type locality: Madagascar, Toamasina, 15km [south of] Mananara, Ivontaka [ca. 16°18′S 49°49′E].

***robusta*** Mesnil, 1950.—Afrotropical: D.R. Congo.

*Thecocarcelia robusta* Mesnil, 1950b: 22. Syntypes, males (1 male in CNC). Type locality: D.R. Congo, Équateur, Eala.

***trichops*** Herting, 1967.—Afrotropical: South Africa, Zambia. Palaearctic: Europe (W. Eur., SW. Eur., SC. Eur., SE. Eur.), Japan, Pal. China.

*Thecocarcelia trichops* Herting, 1967: 4. Holotype male (CNC). Type locality: France, Vaucluse, Lagnes.

Note: Specimens from the Afrotropical Region identified as *Thecocarcelia trichops* Herting, 1967 should be checked to confirm their identity.

***ventralis*** Mesnil, 1959.—Afrotropical: D.R. Congo, Ghana, Nigeria, Sierra Leone, Tanzania.

*Thecocarcelia ventralis* Mesnil, 1959: 2. Holotype male (SMNS). Type locality: Tanzania, “Torina” [not located].

***vibrissata*** Mesnil, 1977.—Afrotropical: Madagascar.

*Thecocarcelia vibrissata* Mesnil, 1977b: 181. Holotype male (MNHN). Type locality: Madagascar, Fianarantsoa, Ifanadiana [ca. 21°18′S 47°38′E].

##### Genus *THELAIRODRINO* Mesnil, 1954

***THELAIRODRINO*** Mesnil, 1954c: 470 (as subgenus of *Thelairosoma* Villeneuve, 1916). Type species: *Thelairosoma gracilis* Mesnil, 1952, by original designation [Oriental].

***anaphe*** (Curran, 1927).—Afrotropical: Cameroon, D.R. Congo, Kenya, Malawi, Nigeria, Tanzania, Zimbabwe.

*Sturmia anaphe* Curran, 1927e: 447. Holotype male (BMNH). Type locality: Tanzania, Morogoro.

Note: *Sturmia anaphe*, described by Curran (1927e: 447), was referred to as “*Sturmia anaphe*, sp. n.” by Curran (1927f: 126) with the accompanying note, “It will be described fully in the Entomologische Mitteilungen” (i.e., in Curran 1927e, which was published first).

***cardinalis*** (Mesnil, 1949).—Afrotropical: D.R. Congo.

*Drino cardinalis* Mesnil, 1949a: 91. Holotype, unspecified sex [male, examined by PC] (MRAC). Type locality: D.R. Congo, Katanga, Lubumbashi [as “Elisabethville”].

***potina*** (Curran, 1927).—Afrotropical: South Africa.

*Sturmia potina* Curran, 1927f: 118. Holotype male (SANC). Type locality: South Africa, KwaZulu-Natal, Port Shepstone.

##### Genus *THELAIROSOMA* Villeneuve, 1916

***THELAIROSOMA*** Villeneuve, 1916c: 499. Type species: *Thelairosoma fumosum* Villeneuve, 1916, by monotypy.

*SEYRIGOMYIA* Mesnil, 1944: 11. Type species: *Seyrigomyia fulvella* Mesnil, 1944, by original designation.

*LESPESIOPSIS* Mesnil, 1954c: 471 (as subgenus of *Thelairosoma* Villeneuve, 1916). Type species: Thelairosoma (Lespesiopsis) coerulescens Mesnil, 1954, by monotypy.

*THELAIROXENIS* Mesnil, 1954c: 472 (as subgenus of *Thelairosoma* Villeneuve, 1916). Type species: Thelairosoma (Thelairoxenis) pallidum Mesnil, 1954, by original designation.

***angustifrons*** (Villeneuve, 1916).—Afrotropical: D.R. Congo, Malawi, Mozambique, Nigeria, South Africa, Tanzania, Uganda.

Sturmia (Blepharipoda) angustifrons Villeneuve, 1916c: 478. Syntypes, 3 males and 1 female (1 male in SAMC). Type locality: South Africa, KwaZulu-Natal, Durban.

***atrum*** Mesnil, 1970.—Afrotropical: Madagascar.

Thelairosoma (Thelairosoma) atrum Mesnil, 1970b: 101. Holotype male (MNHN). Type locality: Madagascar, Toamasina, Périnet [ca. 18°55′S 48°25′E].

***brunnescens*** (Villeneuve, 1934).—Afrotropical: Rwanda, Uganda.

*Erycia brunnescens* Villeneuve, 1934d: 69. Lectotype female (IRSNB), by designation herein (see Lectotype Designations section). Type locality: Uganda, Rwenzori Range [as “Ruwenzori”], 2500m.

***carbonatum*** (Mesnil, 1944).—Afrotropical: Madagascar.

*Seyrigomyia carbonata* Mesnil, 1944: 13. Holotype male (MNHN). Type locality: Madagascar, Toliara, Bekily.

***coerulescens*** Mesnil, 1954.—Afrotropical: Burundi, D.R. Congo, Rwanda, Tanzania.

Thelairosoma (Lespesiopsis) coerulescens Mesnil, 1954c: 471. Holotype male (CNC). Type locality: northwest Tanzania, edge of virgin forest, 1800–2200m.

***comatum*** Villeneuve, 1938.—Afrotropical: Uganda.

*Thelairosoma comatum* Villeneuve, 1938b: 2. Holotype male (IRSNB). Type locality: Uganda, Rwenzori Range [as “Ruwenzori”], 2300m.

***diaphanum*** Mesnil, 1954.—Afrotropical: D.R. Congo.

Thelairosoma (Thelairoxenis) diaphanum Mesnil, 1954c: 472. Holotype male (IRSNB). Type locality: D.R. Congo, Équateur, Eala.

***flavipalpe*** Villeneuve, 1938.—Afrotropical: D.R. Congo.

*Thelairosoma flavipalpe* Villeneuve, 1938b: 3. Holotype male (IRSNB). Type locality: D.R. Congo, Nord-Kivu, Walikale [ca. 1°25′S 28°00′E].

***fulvellum*** (Mesnil, 1944).—Afrotropical: Madagascar.

*Seyrigomyia fulvella* Mesnil, 1944: 12. Holotype, unspecified sex (MNHN). Type locality: Madagascar, Toliara, Bekily.

***fumosum*** Villeneuve, 1916c: 500.—Afrotropical: D.R. Congo, Ghana, Malawi, Mozambique (**new record**, MZUR [PC]), South Africa, Tanzania.

*Thelairosoma fumosum* Villeneuve, 1916c: 500. Lectotype male (SAMC), by fixation of Townsend (1940: 98) (mention of “Ht male” from Durban in SAMC is regarded as a lectotype fixation for the male syntype in SAMC labelled by Villeneuve as “Typ.” [examined by JEOH]). Type locality: South Africa, KwaZulu-Natal, Durban.

***hybridum*** Mesnil, 1970.—Afrotropical: Madagascar.

Thelairosoma (Seyrigomyia) hybrida Mesnil, 1970b: 103. Holotype male (MNHN). Type locality: Madagascar, Antananarivo, Antananarivo [as “Tananarive”].

***ingrami*** Mesnil, 1970.—Afrotropical: Uganda.

Thelairosoma (Seyrigomyia) ingrami Mesnil, 1970b: 103. Holotype male (CNC). Type locality: Uganda, Serere [ca. 1°30′N 33°33′E].

***longicorne*** Mesnil, 1954.—Afrotropical: Zimbabwe.

Thelairosoma (Thelairoxenis) longicorne Mesnil, 1954c: 473. Holotype male (BMNH). Type locality: Zimbabwe, Harare [as “Salisbury”].

***lutescens*** Mesnil, 1954.—Afrotropical: Malawi, South Africa, Zimbabwe.

Thelairosoma (Seyrigomyia) lutescens Mesnil, 1954c: 474. Holotype, unspecified sex (BMNH, not located by D. Whitmore, pers. comm.). Type locality: South Africa.

***major*** Mesnil, 1970.—Afrotropical: Madagascar.

Thelairosoma (Seyrigomyia) major Mesnil, 1970b: 102. Holotype male (MNHN). Type locality: Madagascar, Antananarivo, Mandraka [near Antananarivo, not located].

***melancholicum*** Mesnil, 1970.—Afrotropical: Madagascar.

Thelairosoma (Seyrigomyia) melancholica Mesnil, 1970b: 102. Holotype male (MNHN). Type locality: Madagascar, Toamasina, Périnet [ca. 18°55′S 48°25′E].

***obversum*** Villeneuve, 1943.—Afrotropical: Zimbabwe.

*Thelairosoma obversum* Villeneuve, 1943a: 40. Syntypes, 3 males (not located). Type locality: Zimbabwe, Harare [as “Salisbury”].

***pallidum*** Mesnil, 1954.—Afrotropical: D.R. Congo, Malawi, Nigeria.

Thelairosoma (Thelairoxenis) pallidum Mesnil, 1954c: 472. Holotype male (MRAC). Type locality: D.R. Congo, Katanga, Lubumbashi [as “Elisabethville”].

***palposum*** Villeneuve, 1938.—Afrotropical: “W. Afr. to E. Afr. & sthn Afr.” (Crosskey 1980b: 881), including D.R. Congo, Gabon.

*Thelairosoma palposum* Villeneuve, 1938b: 2. Syntypes, 1 male and 1 female (1 male in IRSNB). Type localities: D.R. Congo, Nord-Kivu, Walikale [ca. 1°25′S 28°00′E] and Gabon, “Bas-Ogooué” [delta region of the Rivière Ogooué].

***pulchellum*** (Mesnil, 1944).—Afrotropical: Madagascar.

*Seyrigomyia pulchella* Mesnil, 1944: 13. Holotype, unspecified sex (MNHN). Type locality: Madagascar, central plateau of Fianarantsoa.

***quadriguttatum*** (Mesnil, 1944).—Afrotropical: Madagascar.

*Seyrigomyia quadriguttata* Mesnil, 1944: 12. Holotype, unspecified sex [male, see O’Hara 1996: 154] (MNHN). Type locality: Madagascar.

***rosatum*** Villeneuve, 1943.—Afrotropical: Malawi.

*Thelairosoma rosatum* Villeneuve, 1943a: 39. Holotype female (not located). Type locality: Malawi, Mt. Mulanje [as “Mt. Mlanje”].

***triste*** Mesnil, 1970.—Afrotropical: Madagascar.

Thelairosoma (Seyrigomyia) tristis Mesnil, 1970b: 102. Holotype male (CNC). Type locality: Madagascar, Toamasina, Périnet [ca. 18°55′S 48°25′E].

***varipes*** Villeneuve, 1943.—Afrotropical: Malawi.

*Thelairosoma varipes* Villeneuve, 1943a: 39. Syntypes, 3 males and 4 females (not located). Type locality: Malawi.

##### Genus *THELYCONYCHIA* Brauer & Bergenstamm, 1889

***THELYCONYCHIA*** Brauer & Bergenstamm, 1889: 89 [also 1890: 21]. Type species: Masicera (Ceromasia) solivaga Rondani, 1861, by monotypy.

*TORINAMYIA* Mesnil, 1959: 2. Type species: *Torinamyia delicatula* Mesnil, 1959, by monotypy.

***delicatula*** (Mesnil, 1959).—Afrotropical: Tanzania, Uganda.

*Torinamyia delicatula* Mesnil, 1959: 2. Holotype male (SMNS). Type locality: Tanzania, “Torina” [not located].

***solivaga*** (Rondani, 1861).—Afrotropical: Botswana, U.A. Emirates, Yemen. Palaearctic: C. Asia, Europe (all except British Is., Scand.), Japan, M. East (Israel), Pal. China, Russia (E. Siberia, S. Far East), Transcaucasia. Oriental: Pakistan.

Masicera (Ceromasia) solivaga Rondani, 1861b: 24. Type(s), male (MZF, Herting 1969: 201; 8 male syntypes and 6 female non-types in MZF [examined by PC]). Type locality: Italy, plain near Parma.

Note: *Thelyconychia solivaga* (Rondani, 1861) of current authors is likely a species complex but is treated here as a single species pending further study. Crosskey’s (1980b: 867) record of *Thelyconychia solivaga* from Canary Islands may have been based on a misidentification because the species was not recorded from there by Tschorsnig and Báez (2002) or Tschorsnig et al. (2004).

##### Genus *THELYMYIOPS* Mesnil, 1950

***THELYMYIOPS*** Mesnil, 1950b: 10 (as subgenus of *Carcelia* Robineau-Desvoidy, 1830). Type species: *Carcelia coniformis* Villeneuve, 1941, by monotypy. **Status n.**

Note: Thelymyiops Mesnil, 1950 was treated as a subgenus of Carcelia Robineau-Desvoidy, 1830 by Crosskey (1980b: 866). It is here raised to a genus and the characters that distinguish it will be given in the Tachinidae chapter of the *Manual of Afrotropical Diptera* (in prep.).

***coniformis*** (Villeneuve, 1941).—Afrotropical: D.R. Congo, Ghana, Tanzania, Uganda.

*Carcelia coniformis* Villeneuve, 1941b: 124. Holotype female (IRSNB). Type locality: D.R. Congo, Équateur, Eala.

##### Unplaced species of Eryciini

***varicornis*** Curran, 1940.—Afrotropical: Zambia, Zimbabwe.

*Phorocera varicornis* Curran, 1940: 7. Holotype female (AMNH). Type locality: border between Zambia and Zimbabwe, Victoria Falls.

Note: *Phorocera varicornis* Curran, 1940 was treated as an “Unplaced species of Goniinae” [= Exoristinae] by Crosskey (1980b: 881) and is moved here based on examination of the holotype by PC. It cannot be placed to genus at the present time.

#### Tribe ETHILLINI

##### Genus *AMNONIA* Kugler, 1971

***AMNONIA*** Kugler, 1971: 71. Type species: *Amnonia carmelitana* Kugler, 1971, by original designation.

***carmelitana*** Kugler, 1971.—Afrotropical: Ethiopia (**new record**, TAU [PC]), Kenya (**new record**, TAU [PC]). Palaearctic: M. East (Israel).

*Amnonia carmelitana* Kugler, 1971: 71. Holotype male (TAU). Type locality: Israel, Zikhron Ya’aqov.

Note: *Amnonia carmelitana* Kugler, 1971 is newly recorded from the Afrotropical Region.

***deemingi*** Zeegers, 2010.—Afrotropical: U.A. Emirates.

*Amnonia deemingi* Zeegers, 2010: 674. Holotype male (RMNH). Type locality: U.A. Emirates, 7km south of Jazīrat al Hamrā [as “al-Jazirat al-Hamra”] (25°39′N 55°45′E).

##### Genus *CALLIETHILLA* Shima, 1979

***CALLIETHILLA*** Shima, 1979: 147. Type species: *Calliethilla caerulea* Shima, 1979, by original designation [Oriental].

***hirta*** Cerretti, 2012.—Afrotropical: Uganda.

*Calliethilla hirta* Cerretti, 2012: 322. Holotype male (TAU). Type locality: Uganda, Rwenzori Range [as “Ruwenzori”], Itojo.

##### Genus *ETHILLA* Robineau-Desvoidy, 1863

***ETHILLA*** Robineau-Desvoidy, 1863a: 202. Type species: *Tachina aemula* Meigen, 1824, by original designation [Palaearctic].

*ETHYLLA* Mesnil, 1939d: 32. Unjustified emendation of *Ethilla* Robineau-Desvoidy, 1863 (see Evenhuis et al. 2010: 76).

***adiscalis*** Mesnil, 1977.—Afrotropical: Madagascar.

*Ethilla adiscalis* Mesnil, 1977b: 173. Holotype male (MNHN). Type locality: Madagascar, Antananarivo, Manjakatompo [ca. 19°21′S 47°18′E].

***tenor*** (Curran, 1927).—Afrotropical: ?Angola, D.R. Congo, ?Kenya, ?Malawi.

*Zenillia tenor* Curran, 1927c: 5. Holotype male (AMNH). Type locality: D.R. Congo, Orientale, Kisangani [as “Stanleyville”].

Note: Crosskey (1984: 270) recorded specimens in BMNH from Angola, Kenya and Malawi that are “probably” *Zenillia tenor* Curran, 1927.

Possibly undescribed sp.: South Africa (BMNH, Crosskey 1984: 270).

##### Genus *ETHYLLOIDES* Verbeke, 1970

***ETHYLLOIDES*** Verbeke, 1970: 286. Type species: *Ethylloides emdeni* Verbeke, 1970, by original designation.

***emdeni*** Verbeke, 1970.—Afrotropical: South Africa.

*Ethylloides emdeni* Verbeke, 1970: 288. Holotype male (MZLU). Type locality: South Africa, Western Cape, Cape Peninsula, Hout Bay, Skoorsteenkop.

##### Genus *GYNANDROMYIA* Bezzi, 1923

***GYNANDROMYIA*** Bezzi, 1923: 97. Type species: *Gynandromyia seychellensis* Bezzi, 1923, by original designation.

*ZENILLIANA* Curran, 1927c: 3 (as subgenus of *Zenillia* Robineau-Desvoidy, 1830). Type species: Zenillia (Zenilliana) devastator Curran, 1927 (= *Myxexorista habilis* Brauer & Bergenstamm, 1891), by monotypy.

*ZELINDOMYIA* Verbeke, 1962a: 166. Type species: *Zelindomyia grossa* Verbeke, 1962, by original designation.

*TRYPHEROSOMA* Verbeke, 1962a: 167. Type species: *Trypherosoma gilva* Verbeke, 1962, by original designation.

Note: *Trypherosoma* Verbeke, 1962 and *Zelindomyia* Verbeke, 1962 were synonymized with *Gynandromyia* Bezzi, 1923 by Crosskey (1984: 200, 271).

***bafwankei*** Verbeke, 1962.—Afrotropical: D.R. Congo.

*Gynandromyia bafwankei* Verbeke, 1962a: 172. Lectotype male (IRSNB), by fixation of Verbeke (1962b: 44) (examination of “Type, 1♂” from Bafwankei is regarded as a lectotype fixation). Type locality: D.R. Congo, Orientale, Bafwakei [as “Bafwankei”, ca. 1°41′N 27°02′E, near Bomili].

Note: The name *Gynandromyia bafwankei* was made available by Verbeke (1962a: 172) in a key that was apparently intended to precede the full description by Verbeke (1962b: 43, as “*Gynandromyia bafwankei* n. sp.”). No specimens were mentioned in the first work but two males, “Type” and “Paratype”, were cited in the second. We regard the “Type” as the lectotype of *Gynandromyia bafwankei* by fixation of Verbeke (1962b: 44).

***basilewskyi*** (Verbeke, 1960).—Afrotropical: Tanzania.

*Zenilliana basilewskyi* Verbeke, 1960: 337. Holotype male (MRAC). Type locality: Tanzania, Olkokola, Mt. Meru, towards northwest, 2500–2600m.

***crypta*** (Verbeke, 1962).—Afrotropical: D.R. Congo.

*Trypherosoma crypta* Verbeke, 1962a: 167, 168. Holotype male (IRSNB). Type locality: D.R. Congo, Orientale, Bafwakei [as “Bafwankei”, ca. 1°41′N 27°02′E, near Bomili].

***fumigata*** (Verbeke, 1962).—Afrotropical: D.R. Congo.

*Trypherosoma fumigata* Verbeke, 1962a: 167, 168. Holotype male (IRSNB). Type locality: D.R. Congo, Équateur, Eala.

***gilva*** (Verbeke, 1962).—Afrotropical: D.R. Congo.

*Trypherosoma gilva* Verbeke, 1962a: 167, 168. Holotype male (IRSNB). Type locality: D.R. Congo, Équateur, Eala.

***grossa*** (Verbeke, 1962).—Afrotropical: D.R. Congo.

*Zelindomyia grossa* Verbeke, 1962a: 167. Holotype male (IRSNB). Type locality: D.R. Congo, Orientale, Mapolo.

***habilis*** (Brauer & Bergenstamm, 1891).—Afrotropical: “widespread W. Afr., E. Afr. & sthn Afr.” (Crosskey 1980b: 861), including D.R. Congo, Malawi, South Africa.

*Myxexorista habilis* Brauer & Bergenstamm, 1891: 332 [also 1891: 28] (as “*habilis* Wd. litt. Coll. Wiedm.”). Type(s), male (NHMW, not located by JEOH). Type locality: South Africa, Western Cape, Cape of Good Hope [as “Cap b. sp.” = “Cap Bonae Spei”].

Zenillia (Zenilliana) devastator Curran, 1927c: 3. Holotype female (AMNH). Type locality: D.R. Congo, Orientale, Kisangani [as “Stanleyville”].

*Zenillia fuscicosta* Curran, 1927c: 4. Holotype male (AMNH). Type locality: D.R. Congo, Orientale, Kisangani [as “Stanleyville”].

***invaginata*** (Villeneuve, 1939).—Afrotropical: D.R. Congo.

*Zenilliana devastator invaginata* Villeneuve, 1939: 9. Syntypes, 2 females (not located). Type localities: D.R. Congo, Orientale, Bafwakei [as “Bafwankei”, ca. 1°41′N 27°02′E, near Bomili] and Équateur, Irebu.

***kibatiana*** Verbeke, 1962.—Afrotropical: D.R. Congo.

*Gynandromyia kibatiana* Verbeke, 1962a: 172. Lectotype male (IRSNB), by fixation of Verbeke (1962b: 41) (examination of “Type, 1♂” from Kibati is regarded as a lectotype fixation). Type locality: D.R. Congo, Nord-Kivu, Parc National des Virunga [as “P.N.A”, former Parc National Albert], foot of Mt. Nyiragongo [as “Nyaragongo”], Kibati [ca. 1°36′S 29°16′E].

Note: The name *Gynandromyia kibatiana* was made available by Verbeke (1962a: 172) in a key that was apparently intended to precede the full description by Verbeke (1962b: 39, as “*Gynandromyia kibatiana* n. sp.”). No specimens were mentioned in the first work but a male “Type” and two “Paratypes” (one male and one female) were cited in the second. We regard the “Type” as the lectotype of *Gynandromyia kibatiana* by fixation of Verbeke (1962b: 41).

***mesnili*** Verbeke, 1962.—Afrotropical: Burundi.

*Gynandromyia mesnili* Verbeke, 1962a: 172. Holotype male (MRAC). Type locality: Burundi, Bururi, 1800–2000m.

Note: The name *Gynandromyia mesnili* was made available by Verbeke (1962a: 172) in a key that was apparently intended to precede the full description by Verbeke (1962b: 38, as “*Gynandromyia mesnili* n. sp.”). No specimens were mentioned in the first work but a single specimen, a male “Type”, was cited in the second. Since the nominal species was clearly based on a single specimen, we regard the “Type” as the holotype of *Gynandromyia mesnili* by monotypy in Verbeke (1962a).

***prima*** Verbeke, 1962.—Afrotropical: Ghana, Kenya, Malawi, South Africa, Uganda, Zimbabwe.

*Gynandromyia prima* Verbeke, 1962a: 172. Lectotype male (IRSNB), by fixation of Verbeke (1962b: 37) (examination of “Type, 1♂” from Aburi is regarded as a lectotype fixation). Type locality: Ghana, Aburi.

Note: The name *Gynandromyia prima* was made available by Verbeke (1962a: 172) in a key that was apparently intended to precede the full description by Verbeke (1962b: 36, as “*Gynandromyia prima* n. sp.”). No specimens were mentioned in the first work but a male “Type” and a series of “Paratypes” (of both sexes) were cited in the second. We regard the “Type” as the lectotype of *Gynandromyia prima* by fixation of Verbeke (1962b: 37).

***saegeri*** Verbeke, 1962.—Afrotropical: D.R. Congo.

*Gynandromyia saegeri* Verbeke, 1962a: 171. Holotype male (MRAC). Type locality: D.R. Congo, Orientale, Parc National de la Garamba [as “P.N.G.”].

Note: The name *Gynandromyia saegeri* was made available by Verbeke (1962a: 171) in a key that was apparently intended to precede the full description by Verbeke (1962b: 44, as “*Gynandromyia saegeri* n. sp.”). No specimens were mentioned in the first work but a single specimen, a male “Type”, was cited in the second. Since the nominal species was clearly based on a single specimen, we regard the “Type” as the holotype of *Gynandromyia saegeri* by monotypy in Verbeke (1962a).

***seychellensis*** Bezzi, 1923.—Afrotropical: Seychelles.

*Gynandromyia seychellensis* Bezzi, 1923: 98. Holotype female [not male as published, Crosskey 1984: 272] (BMNH). Type locality: Seychelles, Mahé Is., Cascade Estate, ca. 1000ft.

##### Genus *MYCTEROMYIELLA* Mesnil, 1966

*MYCTEROMYIA* Mesnil, 1949b: 102. *Nomen nudum* (proposed after 1930 without designation of type species; no included species) (see Evenhuis and O’Hara 2008: 66).

*MYCTEROMYIA* Mesnil, 1950c: 107 (junior homonym of *Mycteromyia* Philippi, 1865). Type species: *Mycteromyia laetifica* Mesnil, 1950, by monotypy (see Evenhuis and O’Hara 2008: 66) [Australasian].

***MYCTEROMYIELLA*** Mesnil, 1966: 232 (*nomen novum* for *Mycteromyia* Mesnil, 1950).

Undescribed sp.: Angola (BMNH, Crosskey 1980b: 862, Crosskey 1984: 269).

##### Genus *NEMORILLOIDES* Brauer & Bergenstamm, 1891

***NEMORILLOIDES*** Brauer & Bergenstamm, 1891: 355 [also 1891: 51]. Type species: *Nemorilloides flaviventris* Brauer & Bergenstamm, 1891, by monotypy.

***carbonata*** Mesnil, 1952.—Afrotropical: D.R. Congo, South Africa.

*Nemorilloides carbonata* Mesnil, 1952a: 10. Holotype male (MRAC). Type locality: D.R. Congo, Nord-Kivu, Rutshuru, 1285m.

***flaviventris*** Brauer & Bergenstamm, 1891.—Afrotropical: South Africa.

*Nemorilloides flaviventris* Brauer & Bergenstamm, 1891: 356 [also 1891: 52]. Lectotype female (NHMW, not located by JEOH), by fixation of Townsend (1941: 111) (mention of “Ht female” from Cape of Good Hope in NHMW is regarded as a lectotype fixation). Type locality: South Africa, Western Cape, Cape of Good Hope [as “Cap b. sp.” = “Cap Bonae Spei”].

##### Genus *PARATRYPHERA* Brauer & Bergenstamm, 1891

***PARATRYPHERA*** Brauer & Bergenstamm, 1891: 328 [also 1891: 24]. Type species: *Paratryphera handlirschii* Brauer & Bergenstamm, 1891 (= *Chetina palpalis* Rondani, 1859), by monotypy [Palaearctic].

***sordida*** (Villeneuve, 1916).—Afrotropical: Botswana, Kenya, South Africa, Tanzania, Uganda, Yemen.

*Zenillia sordida* Villeneuve, 1916c: 485. Holotype male (SAMC, not located by JEOH). Type locality: South Africa, KwaZulu-Natal, Durban.

*sordia*. Incorrect subsequent spelling of *sordida* Villeneuve, 1916 (Curran 1927d: 333).

Note: *Paratryphera sordida* (Villeneuve, 1916) of current authors is likely a species complex but is treated here as a single species pending further study.

Possibly undescribed spp.: Kenya, South Africa (both records based on specimens in BMNH, Crosskey 1984: 270).

##### Genus *PHOROCEROSOMA* Townsend, 1927

***PHOROCEROSOMA*** Townsend, 1927c: 61. Type species: *Phorocerosoma forte* Townsend, 1927 (= *Masicera vicaria* Walker, 1856), by original designation [Oriental].

***aberrans*** Verbeke, 1962.—Afrotropical: Rwanda.

*Phorocerosoma aberrans* Verbeke, 1962a: 170. Holotype female (IRSNB). Type locality: Rwanda, near mouth of Sebeya River, Gisenyi [as “Kisenyi”].

Note: The name *Phorocerosoma aberrans* was made available by Verbeke (1962a: 170) in a key that was apparently intended to precede the full description by Verbeke (1962b: 32, as “*Phorocerosoma aberrans* n. sp.”). No specimens were mentioned in the first work but a single specimen, a female “Type”, was cited in the second. Since the nominal species was clearly based on a single specimen, we regard the “Type” as the holotype of *Phorocerosoma aberrans* by monotypy in Verbeke (1962a).

***albifacies*** Verbeke, 1962.—Afrotropical: Cameroon, D.R. Congo.

*Phorocerosoma albifacies* Verbeke, 1962a: 170. Lectotype female (IRSNB), by fixation of Verbeke (1962b: 32) (examination of “Type, 1♀” from Beni is regarded as a lectotype fixation). Type locality: D.R. Congo, Nord-Kivu, Beni.

Note: The name *Phorocerosoma albifacies* was made available by Verbeke (1962a: 170) in a key that was apparently intended to precede the full description by Verbeke (1962b: 30, as “*Phorocerosoma albifacies* n. sp.”). No specimens were mentioned in the first work but two females, “Type” and “Paratype”, were cited in the second. We regard the “Type” as the lectotype of *Phorocerosoma albifacies* by fixation of Verbeke (1962b: 32).

***caparti*** Verbeke, 1962.—Afrotropical: Burundi, D.R. Congo, Tanzania, Uganda.

*Phorocerosoma caparti* Verbeke, 1962a: 171. Lectotype male (IRSNB), by fixation of Verbeke (1962b: 24) (examination of “Type, 1♂” of *Phorocerosoma vicina* Verbeke from Rutshuru is regarded as a lectotype fixation for *Phorocerosoma caparti* Verbeke). Type locality: D.R. Congo, Nord-Kivu, Rutshuru.

*Phorocerosoma vicina* Verbeke, 1962b: 22 (junior objective synonym of *Phorocerosoma caparti* Verbeke, 1962; both names based on same name-bearing type). Holotype male (IRSNB). Type locality: D.R. Congo, Nord-Kivu, Rutshuru.

Note: The name *Phorocerosoma caparti* was made available by Verbeke (1962a: 171) in a key that was apparently intended to precede the full description by Verbeke (1962b: 22, as “*Phorocerosoma vicina* n. sp.”). No specimens were mentioned in the first work but a male “Type” and a series of “Paratypes” (of both sexes) were cited in the second. No explanation was given for proposing the name *Phorocerosoma vicina* in Verbeke (1962b) for what was named *Phorocerosoma caparti* in Verbeke (1962a). Verbeke (1962b) did not mention the name *Phorocerosoma caparti* and therefore we treat *Phorocerosoma vicina* as a separate nominal species (as did Crosskey 1980b: 862) rather than as a replacement name or incorrect subsequent spelling, with both *Phorocerosoma caparti* and *Phorocerosoma vicina* based on the same name-bearing type. We regard the “Type” (Verbeke 1962b: 24) as both the holotype of *Phorocerosoma vicina* and the lectotype of *Phorocerosoma caparti* (by fixation of Verbeke 1962b: 24), making the two names objective synonyms.

***echinum*** Verbeke, 1962.—Afrotropical: D.R. Congo.

*Phorocerosoma echina* Verbeke, 1962a: 170. Holotype male (MRAC). Type locality: D.R. Congo, Katanga, Parc National de l’Upemba [as “P.N.U.”], Rivière Lufira, [subtributary] Rivière Senze, Kaziba [as “Kaziba, affl. g. Senze, s.-affl. dr. Lufira”], 1140m.

Note: The name *Phorocerosoma echina* was made available by Verbeke (1962a: 170) in a key that was apparently intended to precede the full description by Verbeke (1962b: 29, as “*Phorocerosoma echina* n. sp.”). No specimens were mentioned in the first work but a single specimen, a male “Type”, was cited in the second. Since the nominal species was clearly based on a single specimen, we regard the “Type” as the holotype of *Phorocerosoma echina* by monotypy in Verbeke (1962a).

***elegans*** Verbeke, 1962.—Afrotropical: D.R. Congo.

*Phorocerosoma elegans* Verbeke, 1962a: 171. Holotype male (MRAC). Type locality: D.R. Congo, Orientale, Isiro [as “Paulis”].

Note: The name *Phorocerosoma elegans* was made available by Verbeke (1962a: 171) in a key that was apparently intended to precede the full description by Verbeke (1962b: 28, as “*Phorocerosoma elegans* n. sp.”). No specimens were mentioned in the first work but a single specimen, a male “Type”, was cited in the second. Since the nominal species was clearly based on a single specimen, we regard the “Type” as the holotype of *Phorocerosoma elegans* by monotypy in Verbeke (1962a).

***forcipatum*** Verbeke, 1962.—Afrotropical: D.R. Congo.

*Phorocerosoma forcipata* Verbeke, 1962a: 171. Lectotype male (IRSNB), by fixation of Verbeke (1962b: 27) (examination of “Type, 1♂” from Rutshuru is regarded as a lectotype fixation). Type locality: D.R. Congo, Nord-Kivu, Rutshuru.

Note: The name *Phorocerosoma forcipata* was made available by Verbeke (1962a: 171) in a key that was apparently intended to precede the full description by Verbeke (1962b: 25, as “*Phorocerosoma forcipata* n. sp.”). No specimens were mentioned in the first work but a male and female, as “Type” and “Paratype” respectively, were cited in the second. We regard the “Type” as the lectotype of *Phorocerosoma forcipata* by fixation of Verbeke (1962b: 27).

***pilipes*** (Villeneuve, 1916).—Afrotropical: D.R. Congo, Madagascar, Mauritius, Nigeria, Sierra Leone, South Africa, Uganda.

*Exorista pilipes* Villeneuve, 1916c: 483. Lectotype male (IRSNB), by designation of Verbeke (1962b: 21). Type locality: South Africa, KwaZulu-Natal, Durban.

*postulans* (Walker, 1861).—Misidentification, not Afrotropical [known from Palaearctic, Oriental and Australasian regions].

Note: An unknown species was recorded as *Phorocerosoma anomala* Baranov, 1936 [properly “*anomalum*” in this combination] (currently a synonym of *Phorocerosoma postulans* (Walker, 1861), see Crosskey 1966b: 108 and Sabrosky and Crosskey 1969: 49) from Kenya and Tanzania by Mesnil (1959: 4) and from “tropical Africa” by Crosskey (1973b: 144, 1976: 225). Misidentifications (not recorded from the Afrotropical Region by Crosskey 1980b, O’Hara et al. 2009: 87).

##### Genus *ZELINDOPSIS* Anonymous, 1946

*Zelindopsis* Villeneuve, 1943c: 101. *Nomen nudum* (proposed after 1930 without designation of type species from four included species) (see note and Evenhuis et al. 2008: 34).

***Zelindopsis*** Anonymous *in* Imperial Institute of Entomology, 1946: 208. Type species: *Zelindopsis duplaria* Villeneuve, 1943, by monotypy (see Evenhuis et al. 2008: 34).

Note: Villeneuve (1943c: 100) treated three species of *Zenillia* Robineau-Desvoidy, 1830 as forming “un petit groupe homogène”, including new species *Zenillia stativa*. Villeneuve (1943c: 101) then described a fourth species, *duplaria*, placing it and the preceding three species in his new genus *Zelindopsis*. *Zelindopsis* Villeneuve, 1943 is a *nomen nudum* because it was proposed after 1930 without designation of a type species from four included species (not three included species as stated by Evenhuis et al. 2008: 34).

***bicincta*** (Villeneuve, 1916).—Afrotropical: Ghana, Nigeria, South Africa, Tanzania.

*Zenillia bicinta* Villeneuve, 1916c: 487. Lectotype male (IRSNB), by fixation of Villeneuve (1943c: 101) (treatment of the single male syntype from Nigeria as the “type” is regarded as a lectotype fixation). Type locality: northern Nigeria.

*Zenillia bicincta denudata* Villeneuve, 1943c: 101. Holotype male (IRSNB) (this is also the single paralectotype of *Zenillia bicincta* Villeneuve, 1916). Type locality: Ghana, Aburi.

*Zenillia bicincta aristata* Villeneuve, 1943c: 101. Holotype male (IRSNB). Type locality: South Africa, “Colonie du Cap” (former Cape Province, corresponding to the present-day Western Cape, Eastern Cape, Northern Cape, and North West [in part] provinces).

*bicinta*. Incorrect original spelling of *bicincta* Villeneuve, 1916 (Villeneuve 1916c: 487).

Note: The specific epithet of *Zenillia bicincta* Villeneuve (1916c: 487) was originally published as *bicinta* but subsequent authors (e.g., Villeneuve 1943c, Mesnil 1959, Verbeke 1962a, Crosskey 1980b) used the spelling *bicincta*. The spelling *bicincta* is an incorrect subsequent spelling according to Article 33.3 of the *Code* (ICZN 1999) but because it is in prevailing usage and is attributed to Villeneuve (1943c), it is deemed to be the correct original spelling in compliance with Article 33.3.1.

***cornuta*** Verbeke, 1962.—Afrotropical: D.R. Congo.

*Zelindopsis cornuta* Verbeke, 1962a: 168, 169. Holotype male (IRSNB). Type locality: D.R. Congo, Nord-Kivu, Rutshuru.

***duplaria*** Villeneuve, 1943.—Afrotropical: Tanzania.

*Zelindopsis duplaria* Villeneuve, 1943c: 101. Holotype male (not located). Type locality: Tanzania.

Note: Villeneuve (1943c: 101) described *duplaria* and placed it in *Zelindopsis*. He did not use the combination *Zenillia duplaria* (cf. *Zenillia stativa* Villeneuve, 1943) and hence *duplaria* is treated as described in *Zelindopsis*.

***illita*** (Villeneuve, 1916).—Afrotropical: Burundi, South Africa, Tanzania, Uganda, Zimbabwe.

*Zenillia illita* Villeneuve, 1916c: 486 (as “*Zenillia* (*Pales*?) *illita*”). Holotype female (IRSNB). Type locality: South Africa, KwaZulu-Natal, Durban.

Note: Villeneuve (1916c) wrote in his description of *Zenillia illita* that the “type is a ♀” (p. 486) but further on wrote “Natal, 1♂, Durban, S. Afric. Museum [= SAMC]” (p. 487), evidently referring to the same specimen. Villeneuve (1943c: 100) later confirmed the sex of the holotype as female, writing “La femelle a seule été décrite”. Verbeke (1962b: 169) cited the type as a male from “Natal” in IRSNB. We confirm that the holotype is in IRSNB as stated by Verbeke, but is a female, not a male.

***nigripalpis*** Verbeke, 1962.—Afrotropical: D.R. Congo.

*Zelindopsis nigripalpis* Verbeke, 1962a: 169. Holotype male (IRSNB). Type locality: D.R. Congo, Nord-Kivu, Rutshuru.

***nigrocauda*** (Curran, 1927).—Afrotropical: D.R. Congo.

*Phorocera nigrocauda* Curran, 1927c: 10. Holotype male (AMNH). Type locality: D.R. Congo, Orientale, Kisangani [as “Stanleyville”].

***nitidicauda*** (Curran, 1940).—Afrotropical: South Africa.

*Phorocera nitidicauda* Curran, 1940: 7. Holotype male (SANC). Type locality: South Africa, Gauteng, Pretoria.

Note: *Phorocera nitidicauda* Curran, 1940 is “almost certainly” a synonym of *Zenillia illita* Villeneuve, 1916 according to Crosskey (1984: 270).

***nudapex*** (Curran, 1940).—Afrotropical: South Africa, Zimbabwe.

*Phorocera nudapex* Curran, 1940: 5. Holotype female (AMNH). Type locality: Zimbabwe, Nyanga [as “Inyanga”].

Note: *Phorocera nudapex* Curran, 1940 is “possibly” a synonym of *Zenillia illita* Villeneuve, 1916 according to Crosskey (1984: 270).

***stativa*** (Villeneuve, 1943).—Afrotropical: D.R. Congo.

*Zenillia stativa* Villeneuve, 1943c: 101. Holotype male (IRSNB). Type locality: D.R. Congo, Nord-Kivu, Mukule, 1800m [ca. 1°20′S 29°15′E].

Note: In an unusual nomenclatural action, Villeneuve (1943c: 101) described his new species *stativa* in *Zenillia* Robineau-Desvoidy, 1830 but three paragraphs later placed it in his new genus *Zelindopsis* along with three other species. We interpret *stativa* as intentionally described in the combination *Zenillia stativa* and then moved to *Zelindopsis*, rather than described as *Zelindopsis stativa*.

***villeneuvei*** Verbeke, 1962.—Afrotropical: D.R. Congo.

*Zelindopsis villeneuvei* Verbeke, 1962a: 168, 169. Holotype male (IRSNB). Type locality: D.R. Congo, northwest of Lake Tanganyika [as “N.W. Tanganika”] (not Tanzania as cited by Crosskey 1980b: 863, see note).

Note: The holotype of *Zelindopsis villeneuvei* Verbeke, 1962 was collected by [Rudolf] Grauer in 1910 from “N.W. Tanganika” according to both Verbeke (1962a: 169) and label data (holotype examined by PC). Grauer collected a wide variety of animals including insects, snakes, birds and mammals from “N.W. Tanganika” in 1910, as evidenced by numerous works citing his specimens. A few sources have interpreted “N.W. Tanganika” as Northwest Tanzania (including Crosskey 1980b: 863) but most have treated it as northwest (or northwest shore) of Lake Tanganyika; i.e., in D.R. Congo. One seemingly authoritative reference with the latter interpretation is Chapin (1928: 7, 8), and we accept this view.

***ugandana*** (Curran, 1940).—Afrotropical: Uganda.

*Phorocera ugandana* Curran, 1940: 3. Holotype male (BMNH). Type locality: Uganda, Lake Kibivera [not located].

***zenia*** (Curran, 1940).—Afrotropical: Uganda.

*Phorocera zenia* Curran, 1940: 10. Holotype male (BMNH). Type locality: Uganda, Kampala.

#### Tribe EXORISTINI

##### Genus *BESSA* Robineau-Desvoidy, 1863

***BESSA*** Robineau-Desvoidy, 1863b: 164. Type species: *Bessa secutrix* Robineau-Desvoidy, 1863 (= *Tachina selecta* Meigen, 1824), by original designation [Palaearctic].

***africana*** (Curran, 1941).—Afrotropical: Kenya (**new record**, MZUR [PC]), Zimbabwe.

*Kuwanimyia africana* Curran, 1941: 9. Holotype male (AMNH). Type locality: Zimbabwe, Harare [as “Salisbury”].

##### Genus *CHAETEXORISTA* Brauer & Bergenstamm, 1894

***CHAETEXORISTA*** Brauer & Bergenstamm, 1894: 616 [also 1895: 80]. Type species: *Chaetexorista javana* Brauer & Bergenstamm, 1894, by monotypy [Oriental].

*ISOPROSOPAEA* Villeneuve, 1938a: 1 (as subgenus of *Prosopea* Rondani, 1861, as “*Prosopaea* B. B.”). *Nomen nudum* (proposed after 1930 without designation of type species from two included species) (see Evenhuis et al. 2008: 16).

*ISOPROSOPAEA* Townsend, 1943: 336. Type species: *Prosopaea sororcula* Villeneuve, 1938, by original designation (see Evenhuis et al. 2008: 16 and Evenhuis et al. 2015: 149).

*HYGIA* Mesnil, 1949b: 104. *Nomen nudum* (proposed after 1930 without designation of type species; no included species) (see Evenhuis and O’Hara 2008: 66).

*HYGIA* Mesnil, 1952c: 222 (junior homonym of *Hygia* Uhler, 1861). Type species: *Blepharipoda eutachinoides* Baranov, 1932, by original designation (see Evenhuis and O’Hara 2008: 66) [Oriental].

*PARAPODOMYIA* Mesnil, 1952c: 235 (as subgenus of *Blepharella* Macquart, 1851). *Nomen nudum* (proposed after 1930 without designation of type species from two included species) (see O’Hara 1996: 127 and Evenhuis et al. 2008: 23).

*PARAPODOMYIA* Mesnil, 1956c: 560 (as full genus). Type species: *Blepharella claripennis* Mesnil, 1952, by original designation (see O’Hara 1996: 127 and Evenhuis et al. 2008: 23).

***claripennis*** (Mesnil, 1952).—Afrotropical: D.R. Congo.

*Blepharella claripennis* Mesnil, 1952c: 236. Holotype male (CNC). Type locality: D.R. Congo, Équateur, Eala.

Note: Mesnil (1952c: 236) proposed new species *Blepharella claripennis* in new subgenus *Parapodomyia*, but *Parapodomyia* Mesnil, 1952 is an unavailable name.

***dives*** (Villeneuve, 1938).—Afrotropical: Tanzania.

*Prosopaea dives* Villeneuve, 1938a: 1. Holotype female (not located). Type locality: Tanzania.

Note: Villeneuve (1938a: 1) proposed new species *Prosopaea dives* in new subgenus *Isoprosopaea*, but *Isoprosopaea* Villeneuve, 1938 is an unavailable name.

***langi*** (Curran, 1927).—Afrotropical: Angola, D.R. Congo, Ghana, Nigeria, Sierra Leone, Uganda, Zimbabwe.

*Podomyia langi* Curran, 1927a: 9. Holotype male (AMNH). Type locality: D.R. Congo, Orientale, Kisangani [as “Stanleyville”].

***ocellaris*** (Curran, 1927).—Afrotropical: D.R. Congo, Nigeria.

*Podomyia ocellaris* Curran, 1927a: 9. Holotype male (AMNH). Type locality: D.R. Congo, Orientale, Kisangani [as “Stanleyville”].

***sororcula*** (Villeneuve, 1938).—Afrotropical: Burundi (**new record**, MZUR [PC]), D.R. Congo.

*Prosopaea sororcula* Villeneuve, 1938a: 2. Holotype female (MRAC). Type locality: D.R. Congo, Nord-Kivu, Rutshuru.

Note: Villeneuve (1938a: 2) proposed new species *Prosopaea sororcula* in new subgenus *Isoprosopaea*, but *Isoprosopaea* Villeneuve, 1938 is an unavailable name.

##### Genus *CHAETORIA* Becker, 1908

***CHAETORIA*** Becker, 1908: 113. Type species: *Chaetoria stylata* Becker, 1908, by monotypy.

*CLISTORRHINIA* Bezzi *in* Bezzi & Lamb, 1926: 570. Type species: *Clistorrhinia aurifrons* Bezzi, 1926, by monotypy.

***aurifrons*** (Bezzi, 1926).—Afrotropical: Madagascar (**new record**, TAU [PC]), Mauritius (Rodrigues Is.).

*Clistorrhinia aurifrons* Bezzi *in* Bezzi & Lamb, 1926: 572. Lectotype male (BMNH), by fixation of Townsend (1940: 176) (mention of “Ht male” from Rodrigues Island in BMNH is regarded as a lectotype fixation). Type locality: Mauritius, Rodrigues Is.

***stylata*** Becker, 1908.—Afrotropical: Botswana, Mozambique, Nigeria, Senegal, U.A. Emirates, Yemen. Palaearctic: C. Asia, Europe (SC. Eur.), N. Africa (Canary Is., NW. Africa).

*Chaetoria stylata* Becker, 1908: 114. Lectotype female (ZMHB), by fixation of Townsend (1941: 211) (mention of “Ht” from Tenerife in “Liegnitz” [now Legnica (Poland), referring to Becker’s personal collection that is now in ZMHB] is regarded as a lectotype fixation). Type locality: Canary Islands, Tenerife.

##### Genus *CHETOGENA* Rondani, 1856

*SALIA* Robineau-Desvoidy, 1830: 108 (junior homonym of *Salia* Hübner, 1818). Type species: *Salia echinura* Robineau-Desvoidy, 1830 (= *Tachina obliquata* Fallén, 1810), by subsequent designation of Robineau-Desvoidy (1863a: 553) [Palaearctic].

***CHETOGENA*** Rondani, 1856: 68. Type species: *Salia rondaniana* Villeneuve, 1931, by fixation of O’Hara and Wood (2004: 145) under Article 70.3.2 of the *Code* (ICZN 1999), misidentified as *Tachina gramma* Meigen, 1824 in the original designation by Rondani (1856) [Palaearctic].

*SPOGGOSIA* Rondani, 1859: 182. Type species: *Spoggosia occlusa* Rondani, 1859 (= *Tachina obliquata* Fallén, 1810), by monotypy [Palaearctic].

*CHAETOGENA* Bezzi & Stein, 1907: 315. Unjustified emendation of *Chetogena* Rondani, 1856 (see O’Hara et al. 2011: 54, 259).

*STOMATOMYIA* Brauer & Bergenstamm, 1889: 98 [also 1890: 30]. Type species: *Chetogena filipalpis* Rondani, 1859, by subsequent designation of Brauer (1893: 483) [Palaearctic].

Note: Subgenera of *Chetogena* Rondani, 1856 are not recognized here because the subgeneric placements of the Afrotropical species require more study.

***acuminata*** Rondani, 1859.—Afrotropical: Cameroon, Nigeria, Senegal, Tanzania, U.A. Emirates, Yemen. Palaearctic: C. Asia, Europe (all except Scand.), Japan, M. East (Israel), Mongolia, N. Africa (Canary Is., Madeira), Pal. China, Russia (W. Siberia, E. Siberia, S. Far East), Transcaucasia. Oriental: Indonesia, Malaysia, Orien. China.

*Chetogena acuminata* Rondani, 1859: 180. Syntypes, males and females (MZF, Herting 1969: 189). Type localities: Italy, Apennines and fields near Parma.

*Stomatomyia acuminata approximata* Villeneuve *in* Frey, 1936: 145. Lectotype male (FMNHH), by designation of Herting (1983a: 2). Type locality: Canary Islands, Tenerife, Agua Mansa.

***cercosa*** Kugler, 1980.—Afrotropical: U.A. Emirates. Palaearctic: M. East (Israel).

*Chaetogena cercosa* Kugler, 1980a: 31. Holotype male (TAU). Type locality: Israel, Elat [also commonly as Eilat].

***echinata*** (Mesnil, 1939).—Afrotropical: Madagascar.

*Stomatomyia echinata* Mesnil, 1939c: 172. Syntypes, males and females (“nombreux exemplaires”) (MNHN). Type locality: Madagascar, Toliara, Bekily.

***nigrofasciata*** (Strobl, 1902).—Afrotropical: Kenya. Palaearctic: C. Asia, Europe (SE. Eur., Turkey), M. East (all), N. Africa (NW. Africa), Transcaucasia.

Phorocera (Parasetigena) nigrofasciata Strobl, 1902: 488. Holotype female (NMBA, Chvála 2008: 191). Type locality: Serbia, Niš.

*Stomatomyia repanda* Mesnil, 1939c: 171. Syntypes, 1 male and 1 female (MNHN). Type localities: Morocco, basin of Wadi Ouergha (Skel [not located]) and near Essaouira [as “Mogador”] (Bou Tazzert).

Note: The description of *Phorocera nigrofasciata* Strobl, 1902 was published first in Serbian (Strobl 1902: 488) and later in German (Strobl 1905: 548).

***setertia*** (Curran, 1940).—Afrotropical: Malawi, South Africa, Tanzania.

*Phorocera setertia* Curran, 1940: 8. Holotype male (SANC). Type locality: South Africa, Mpumalanga, Barberton.

***setosaria*** (Curran, 1940).—Afrotropical: Tanzania, Zimbabwe.

*Phorocera setosaria* Curran, 1940: 8. Holotype female (AMNH). Type locality: Zimbabwe, Harare [as “Salisbury”].

***setosina*** (Curran, 1940).—Afrotropical: South Africa, Tanzania, Uganda, Zimbabwe.

*Phorocera setosina* Curran, 1940: 9. Holotype male (BMNH). Type locality: Tanzania.

##### Genus *CRASSICORNIA* Kugler, 1980

***CRASSICORNIA*** Kugler, 1980a: 28 (as subgenus of *Exorista* Meigen, 1803). Type species: Exorista (Crassicornia) pilosa Kugler, 1980, by original designation.

***pilosa*** (Kugler, 1980).—Afrotropical: Ethiopia. Palaearctic: M. East (Israel).

Exorista (Crassicornia) pilosa Kugler, 1980a: 28. Holotype male (TAU). Type locality: Israel, Arava Valley, Hazeva.

##### Genus *EXORISTA* Meigen, 1803

###### Subgenus *EXORISTA* Meigen, 1803

***EXORISTA*** Meigen, 1803: 280. Type species: *Musca larvarum* Linnaeus, 1758 (as “*Musca larvarum* Fabr.”), by monotypy [Palaearctic].

###### Subgenus *EXORISTELLA* Herting, 1984

*Exoristella* Mesnil, 1946: 47 (as subgenus of *Exorista* Meigen, 1803). *Nomen nudum* (proposed after 1930 without designation of type species from two included species).

*Exoristella* Mesnil, 1960a: 565, 597 (as subgenus of *Exorista* Meigen, 1803). *Nomen nudum* (proposed after 1930 without designation of type species from three included species).

*Exoristella* Herting, 1984: 6 (as subgenus of *Exorista* Meigen, 1803). Type species: *Tachina glossatorum* Rondani, 1859, by original designation [Palaearctic].

Note: The nomenclatural history of *Exoristella* Mesnil was discussed by O’Hara (1996: 124) and Evenhuis et al. (2008: 13).

***duplaria*** (Villeneuve, 1916).—Afrotropical: Kenya, Malawi, Nigeria, South Africa, Tanzania, Uganda, Zambia.

*Tachina duplaria* Villeneuve, 1916c: 493. Syntypes, males and females (CNC, BMNH, SAMC [no specimens located in SAMC by JEOH]). Type localities: Malawi (Mt. Mulanje [as “Mt. Mlanje”]), Nigeria, and South Africa (KwaZulu-Natal, Durban).

###### Subgenus *PODOTACHINA* Brauer & Bergenstamm, 1891

*PODOTACHINA* Brauer & Bergenstamm, 1891: 350 [also 1891: 46]. Type species: *Tachina sorbillans* Wiedemann, 1830, by subsequent designation of Townsend (1916b: 8).

***atricans*** (Villeneuve, 1938).—Afrotropical: Malawi, Nigeria.

*Eutachina atricans* Villeneuve, 1938a: 3. Syntypes, 1 male and 3 females (CNC). Type locality: Malawi, Mt. Mulanje [as “Mt. Mlanje”].

***flavicans*** Mesnil, 1941.—Afrotropical: D.R. Congo.

*Exorista flavicans* Mesnil, 1941: 21. Holotype male (MNHN). Type locality: D.R. Congo, Sud-Kivu, Lake Kivu region, “Bulira” [probably Bulera, ca. 2°03′S 28°54′E].

***rubricans*** Mesnil, 1941.—Afrotropical: Djibouti.

*Exorista sorbillans rubricans* Mesnil, 1941: 21. Syntypes, three males (MNHN). Type locality: Djibouti, Obock [as “Obok”].

***sericans*** Mesnil, 1939.—Afrotropical: ?D.R. Congo, Madagascar.

*Exorista sericans* Mesnil, 1939b: 198. Holotype male (MNHN, not located by O’Hara 1996: 156). Type locality: Madagascar, Toliara, Bekily.

Note: Verbeke’s (1962b: 59) record of *Exorista sericans* Mesnil, 1939 from D.R. Congo needs confirmation.

***sorbillans*** (Wiedemann, 1830).—Afrotropical: Cameroon, D.R. Congo, Kenya, Malawi, Nigeria, Sierra Leone, Uganda. Palaearctic: C. Asia, Europe (W. Eur., E. Eur., SC. Eur., SE. Eur.), Japan, M. East (Israel), Mongolia, N. Africa (Canary Is.), Pal. China. Oriental: India, Indonesia, Nepal, Orien. China, Philippines, Ryukyu Is., Sri Lanka, Taiwan, Thailand, Vietnam. Australasian: Australia, N. Australasian.

*Tachina sorbillans* Wiedemann, 1830: 311. Syntypes, unspecified number and sex (3 males in NHMW). Type locality: Canary Islands, Tenerife.

Note: Wiedemann (1830: 312) described *Tachina sorbillans* from an unspecified number of specimens in “v. Winthem’s und meiner Sammlung”. Townsend (1932: 45) studied the “male Ht in Wien” and this statement about the “Ht” has been accepted as a lectotype fixation by subsequent authors (e.g., Crosskey 1976: 223, Herting 1984: 6, O’Hara et al. 2009: 93). However, an examination of the NHMW holdings (by JEOH) has revealed three male syntypes of *Tachina sorbillans*, two from “Coll. Winthem” (both with red “Type” labels) and one from “Coll. Wiedem.”. The “Ht” of Townsend (1932) cannot be recognized among the syntypes in NHMW, and hence his statement “male Ht in Wien” cannot be accepted as a lectotype fixation.

***tessellans*** Mesnil, 1939.—Afrotropical: D.R. Congo. Palaearctic: N. Africa (NW. Africa), M. East (Israel).

*Exorista tessellans* Mesnil, 1939b: 197. Syntypes, 1 male and 3 females (MNHN). Type locality: Algeria, El Goléa.

*tesselans*. Incorrect subsequent spelling of *tessellans* Mesnil, 1939 (Cerretti and Freidberg 2009: 12).

###### Subgenus *PTILOTACHINA* Brauer & Bergenstamm, 1891

*PTILOTACHINA* Brauer & Bergenstamm, 1891: 350 [also 1891: 46]. Type species: *Exorista florentina* Herting, 1975, by fixation of O’Hara et al. (2009: 94) under Article 70.3.2 of the *Code* (ICZN 1999), misidentified as *Tachina civilis* Rondani, 1859 in the fixation by monotypy of Brauer and Bergenstamm (1891) [Palaearctic].

***cardinalis*** Mesnil, 1939.—Afrotropical: Côte d’Ivoire.

*Exorista cardinalis* Mesnil, 1939b: 194. Syntypes, 2 males and 1 female (MNHN). Type locality: Côte d’Ivoire, Assinie [as “Assini”].

***ebneri*** (Villeneuve, 1922).—Afrotropical: Kenya, Senegal, Sudan. Palaearctic: M. East (Israel).

*Tachina ebneri* Villeneuve, 1922b: 62. Lectotype male (CNC), by fixation of Mesnil (1960a: 589) (mention of “♂ (Typus)” is regarded as a lectotype fixation following Cooper and O’Hara 1996: 73). Type locality: Sudan (Kordofan according to label data).

Note: Villeneuve was not always very precise when listing specimens belonging to his new species. For *Tachina ebneri*, he wrote at the end of the description that he had “obtenue en plusieurs exemplaires d’*Auchmophila cordofensis*, en juin” (Villeneuve 1922b: 62). If this statement is strictly interpreted, then specimens reared from this host that are not dated June are not part of the type series. In our view this was not Villeneuve’s intent, because he labelled another specimen (now in CNC) reared from this host on a different date as “Typ”. This specimen was seen by Mesnil (1960a: 589), who referred to it as “Typus” of *Tachina ebneri*. Cooper and O’Hara (1996: 73) interpreted Mesnil’s mention of “Typus” as a lectotype fixation and we follow this interpretation. The lectotype was collected from Kordofan, Sudan (misquoted as “Kondofan” by Cooper and O’Hara 1996: 73; Kordofan was a former province of Sudan that has since been divided into the states of North Kordofan and South Kordofan). A male in NHMW reared from “*Auchmophila kordofensis*” and dated June, from “el Obeid” (Sudan), also bears a Villeneuve “Typ.” label and is considered a paralectotype (examined by JEOH). It was not uncommon for Villeneuve to label more than one specimen in a type series as “Typ.”.

***elegantula*** Mesnil, 1939.—Afrotropical: Djibouti.

*Exorista elegantula* Mesnil, 1939b: 195. Holotype male (MNHN). Type locality: Djibouti, Obock [as “Obok”].

***neta*** (Curran, 1927).—Afrotropical: D.R. Congo, South Africa, Zimbabwe.

*Thrycolyga neta* Curran, 1927c: 2. Holotype male (AMNH). Type locality: D.R. Congo, Orientale, Kisangani [as “Stanleyville”].

*Tricholyga piligena* Villeneuve, 1938a: 3. Syntypes, 3 females (not located). Type localities: South Africa, KwaZulu-Natal, Wartburg and Zimbabwe, Harare [as “Salisbury”].

***niveipennis*** Mesnil, 1939.—Afrotropical: Mozambique.

*Exorista niveipennis* Mesnil, 1939b: 196. Holotype male (MNHN). Type locality: Mozambique, “Nova Choupanga” [near Chemba but not located].

***xanthaspis*** (Wiedemann, 1830).—Afrotropical: “widespread Afrotrop. Reg.” (Crosskey 1980b: 860), including Madagascar, Seychelles, Sudan, U.A. Emirates, Yemen. Palaearctic: C. Asia, Europe (all except British Is., Scand.), M. East (Israel), Mongolia, Pal. China, Russia (W. Russia, W. Siberia), Transcaucasia. Oriental: India, Indonesia, Orien. China, Ryukyu Is., Taiwan. Australasian: N. Australasian.

*Tachina xanthaspis* Wiedemann, 1830: 314. Syntypes, males and females (SMF, probably lost, Crosskey 1976: 223–224). Type locality: Nubia region [as “Nubien”, a region in southern Egypt and northern Sudan, recorded here as Sudan following Crosskey 1980b: 860].

*Tachina pyrrhocera* Wiedemann, 1830: 314. Type(s), female (SMF or lost). Type locality: Nubia region [as “Nubien”, a region in southern Egypt and northern Sudan, recorded here as Sudan following Crosskey 1980b: 860].

*Tachina fallax pseudofallax* Villeneuve, 1920a: 151. Syntypes, two males (CNC). Type locality: South Africa, Eastern Cape, Willowmore.

Larvaevora (Ptilotachina) fallax aethiopica Rohdendorf, 1931: 348. Holotype male (not located). Type locality: Sudan, Wad Medani.

*Tachina fallax* of authors (e.g., Villeneuve 1913c: 34), not Meigen, 1824. Misidentification (Crosskey 1980b: 860).

Note: The relative priority of *Tachina xanthaspis* Wiedemann, 1830 and *Tachina pyrrhocera* Wiedemann, 1830, when the two are treated as synonyms, was established by Crosskey (1980b: 860), as the First Reviser (Article 24.2.2 of the *Code*, ICZN 1999). *Exorista xanthaspis* (Wiedemann) of current authors is likely a species complex but is treated here as a single species pending further study.

###### Subgenus *SPIXOMYIA* Crosskey, 1967

*SCOTIELLA* Mesnil, 1940: 39 (as subgenus of *Exorista* Meigen, 1803) (junior homonym of *Scotiella* Delo, 1935). Type species: Exorista (Scotiella) bisetosa Mesnil, 1940, by original designation [Oriental].

*SPIXOMYIA* Crosskey, 1967a: 28 (*nomen novum* for *Scotiella* Mesnil, 1940).

***dasyops*** (Villeneuve, 1943).—Afrotropical: Nigeria.

*Sturmia dasyops* Villeneuve, 1943a: 40. Holotype male (CNC). Type locality: Nigeria, Degema.

###### Subgenus *TRICOLIGA* Rondani, 1856

*TRICOLIGA* Rondani, 1856: 68, 225. Type species: *Tricoliga nova* Rondani, 1856, by original designation (see O’Hara et al. 2011: 184 for an explanation of the correct spelling of this genus-group name) [Palaearctic].

*TRICOLYGA* Schiner, 1861: 456. Unjustified emendation of *Tricoliga* Rondani, 1856 (see O’Hara et al. 2011: 184, 268).

*THRYCOLYGA*. Incorrect original spelling of *Tricoliga* Rondani, 1856 (Rondani 1856: 68) (see O’Hara et al. 2011: 180, 184).

*TRICHOLYGA*. Incorrect subsequent spelling of *Tricoliga* Rondani, 1856 (Rondani 1865: 207, 208) (see O’Hara et al. 2011: 182).

***buccalis*** Mesnil, 1940.—Afrotropical: Madagascar.

*Exorista buccalis* Mesnil, 1940: 38. Holotype male (MNHN). Type locality: Madagascar, Toliara, Bekily.

###### Unplaced to subgenus

***abdominalis*** (Curran, 1927).—Afrotropical: D.R. Congo.

*Thrycolyga abdominalis* Curran, 1927a: 8. Holotype male (AMNH). Type locality: D.R. Congo, Orientale, Kisangani [as “Stanleyville”].

***africana*** (Rohdendorf, 1931).—Afrotropical: Nigeria, South Africa, Sudan, Zimbabwe.

*Tricholyga africana* Rohdendorf, 1931: 347. Holotype male (BMNH). Type locality: Sudan, Wad Medani.

***capensis*** (Macquart, 1855).—Afrotropical: South Africa.

*Masicera capensis* Macquart, 1855: 120 [also 1855: 100]. Lectotype male (BMNH), by fixation of Crosskey (1971: 273) (examination of “? holotype ♂” from “cap de Bonne-Espérance” in BMNH is regarded as a lectotype fixation). Type locality: South Africa, Western Cape, Cape of Good Hope [as “cap de Bonne-Espérance”].

***creole*** (Curran, 1927).—Afrotropical: D.R. Congo.

*Thrycolyga creole* Curran, 1927c: 1. Holotype male (AMNH). Type locality: D.R. Congo, Orientale, Kisangani [as “Stanleyville”].

*iniqua* (Brauer & Bergenstamm, 1891).

*Tricholyga iniqua* Brauer & Bergenstamm, 1891: 403, 431 [also 1891: 99, 127] (as “*iniqua* Mg.” on p. 99 [403]; as “*iniqua* C. Wth. litt. *Tricholyga*. Cap. [Cape of Good Hope]” on p. 127 [431]). *Nomen nudum*.

***sessitans*** (Curran, 1927).—Afrotropical: D.R. Congo, Malawi, Nigeria, Sierra Leone, South Africa, Zimbabwe.

*Thrycolyga sessitans* Curran, 1927c: 2. Holotype male (AMNH). Type locality: D.R. Congo, Orientale, Kisangani [as “Stanleyville”].

Undescribed spp.: “Many undescribed species, including at least 13 with distinct male genitalia that have been confused in collections under *Exorista sorbillans*” (BMNH, Crosskey 1984: 268).

##### Genus *NEOPHRYXE* Townsend, 1916

***NEOPHRYXE*** Townsend, 1916d: 318. Type species: *Neophryxe psychidis* Townsend, 1916, by original designation [Palaearctic].

***australe*** Cerretti, 2012.—Afrotropical: Namibia.

*Neophryxe australe* Cerretti, 2012: 318. Holotype male (NMNW). Type locality: Namibia, Caprivi, near Katima Mulilo, Salambala Forest (17°50′02″S 24°36′20″E).

***namibica*** Cerretti, 2012.—Afrotropical: Namibia.

*Neophryxe namibica* Cerretti, 2012: 320. Holotype male (NMNW). Type locality: Namibia, Okavango, near Rundu, Mile 46 (18°18′30″S 19°15′29″E).

##### Genus *PHORINIA* Robineau-Desvoidy, 1830

***PHORINIA*** Robineau-Desvoidy, 1830: 118. Type species: *Phorinia aurifrons* Robineau-Desvoidy, 1830, by subsequent designation of Robineau-Desvoidy (1863a: 491) [Palaearctic].

*BESSIOLA* Mesnil, 1960b: 630 (as subgenus of *Phorinia* Robineau-Desvoidy, 1830). Type species: *Bessa oblimata* Mesnil, 1944, by monotypy.

***atypica*** Curran, 1927.—Afrotropical: Cameroon, Ghana, Kenya, Malawi, South Africa, Sudan, Tanzania.

*Phorinia atypica* Curran, 1927d: 336. Holotype male (BMNH). Type locality: South Africa, KwaZulu-Natal, Durban.

***cinctella*** Mesnil, 1971.—Afrotropical: Uganda.

*Phorinia cinctella* Mesnil, 1971: 70. Holotype male (CNC). Type locality: Uganda, Kawanda [located a few kilometers north of Kampala].

***oblimata*** (Mesnil, 1944).—Afrotropical: Guinea.

*Bessa oblimata* Mesnil, 1944: 16. Holotype male (MNHN). Type locality: Guinea.

***pulverulenta*** (Karsch, 1886).—Afrotropical: Angola, D.R. Congo, Kenya, Malawi, Nigeria, Uganda, Zimbabwe.

*Phorocera pulverulenta* Karsch, 1886b: 341. Holotype, unspecified sex [male, examined by JEOH] (ZMHB). Type locality: Angola, Pungo Andongo.

***pumila*** Mesnil, 1971.—Afrotropical: Uganda.

*Phorinia pumila* Mesnil, 1971: 70. Holotype female (CNC, not located). Type locality: Uganda, Kampala.

***sadista*** (Curran, 1940).—Afrotropical: South Africa, Zimbabwe.

*Phorocera sadista* Curran, 1940: 4. Holotype female (AMNH). Type locality: South Africa, KwaZulu-Natal, Durban.

***verritus*** (Walker, 1849).—Afrotropical: “widespread W. Afr. to Ethiopia, E. Afr. & sthn Afr.” (Crosskey 1980b: 861), including Côte d’Ivoire, D.R. Congo, Guinea, South Africa.

*Tachina verritus* Walker, 1849: 774. Type(s), unspecified sex (1 female in BMNH according to BMNH database). Type locality: South Africa.

*Chetogena tricolor* Bigot, 1891: 377. Holotype male (BMNH, not lost as suspected by Crosskey 1971: 296). Type locality: Côte d’Ivoire, Assinie.

*verittus*. Incorrect subsequent spelling of *verritus* Walker, 1849 (Mesnil 1958: 251).

Undescribed sp.: Madagascar (TAU, examined by PC).

#### Tribe GONIINI

##### Genus *AGAEDIOXENIS* Villeneuve, 1939

*GAEDIOXENIS* Villeneuve, 1937: 206. *Nomen nudum* (proposed after 1930 without designation of type species from two included species).

*GAEDIOXENIS* Villeneuve, 1939: 1. *Nomen nudum* (proposed after 1930 without designation of type species from two included species).

***AGAEDIOXENIS*** Villeneuve, 1939: 2 (as subgenus of *Gaedioxenis* Villeneuve, 1937 [*nomen nudum*]). Type species: Gaedioxenis (Agaedioxenis) brevicornis Villeneuve, 1939, by monotypy

*GAEDIOXENIS* Townsend, 1943a: 335. Type species: *Gaedioxenis setifrons* Villeneuve, 1937, by original designation.

Note: Villeneuve (1937) described the genus *Gaedioxenis* with two new species, then two years later Villeneuve (1939) added another new species to the genus and created for it the new subgenus *Agaedioxenis*. The name *Gaedioxenis* was a *nomen nudum* in both publications but *Agaedioxenis* was validly proposed in the second. The valid name of the genus is thus *Agaedioxenis*, as explained in more detail by Cerretti et al. (2015: 502) in their revision of *Agaedioxenis* Villeneuve, 1939 and *Eugaedioxenis* Cerretti, O’Hara & Stireman, 2015.

***brevicornis*** (Villeneuve, 1939).—Afrotropical: South Africa, Zimbabwe.

Gaedioxenis (Agaedioxenis) brevicornis Villeneuve, 1939: 1. Holotype male (BMNH). Type locality: Zimbabwe, Mutare [as “Umtali”] District.

*Gaedioxenis propinqua* Villeneuve, 1939: 2. Holotype female (not located). Type locality: South Africa, KwaZulu-Natal.

Note: The relative priority of Gaedioxenis (Agaedioxenis) brevicornis Villeneuve, 1939 and *Gaedioxenis propinqua* Villeneuve, 1939, when the two are treated as synonyms, was established by Cerretti et al. (2015: 506), as the First Reviser (Article 24.2.2 of the *Code*, ICZN 1999).

***kirkspriggsi*** Cerretti, O’Hara & Stireman, 2015.—Afrotropical: South Africa.

*Agaedioxenis kirkspriggsi* Cerretti, O’Hara & Stireman *in* Cerretti et al., 2015: 507. Holotype male (NMB). Type locality: South Africa, Free State, Harrismith, Mooihekkop, ca. 1800m (28°10′50.0″S 29°10′51.1″E).

***setifrons*** (Villeneuve, 1937).—Afrotropical: South Africa.

*Gaedioxenis setifrons* Villeneuve, 1937a: 207. Holotype female (CNC). Type locality: South Africa, Western Cape, Stellenbosch.

***succulentus*** Cerretti, O’Hara & Stireman, 2015.—Afrotropical: South Africa.

*Agaedioxenis succulentus* Cerretti, O’Hara & Stireman *in* Cerretti et al., 2015: 507. Holotype male (MZUR). Type locality: South Africa, Western Cape, Ceres Bergfynbos Reserve, 459m (33°23′1.91″S 19°17′20.16″E).

***timidus*** Cerretti, O’Hara & Stireman, 2015.—Afrotropical: South Africa.

*Agaedioxenis timidus* Cerretti, O’Hara & Stireman *in* Cerretti et al., 2015: 508. Holotype male (CNC). Type locality: South Africa, Western Cape, Cape Town.

##### Genus *BLEPHARELLA* Macquart, 1851

***BLEPHARELLA*** Macquart, 1851b: 176 [also 1851b: 203]. Type species: *Blepharella lateralis* Macquart, 1851, by original designation [Oriental].

*PODOMYIA* Brauer & Bergenstamm, 1889: 96 [also 1890: 28]. Type species: *Eurigaster setosa* Doleschall, 1858 (= *Blepharella lateralis* Macquart, 1851), by monotypy [Oriental].

*CONGOCHRYSOSOMA* Townsend, 1916a: 174. Type species: *Congochrysosoma snyderi* Townsend, 1916, by original designation.

*PHRYXOSTURMIA* Townsend, 1927c: 68. Type species: *Phryxosturmia jacobsoni* Townsend, 1927 (= *Blepharella lateralis* Macquart, 1851), by original designation [Oriental].

*AFROSTURMIA* Curran, 1927f: 126. Type species: *Afrosturmia orbitalis* Curran, 1927, by original designation. **Syn. n.**

*APILIA* Malloch, 1930a: 345. Type species: *Apilia cilifera* Malloch, 1930 [= *Blepharella lateralis* Macquart, 1851], by original designation [Australasian].

*PUJOLINA* Mesnil, 1968b: 2. Type species: *Pujolina bicolor* Mesnil, 1968, by original designation.

Note: Macquart (1851b: 177 [also 1851b: 204]) noted, about his new genus *Blepharella*, “Le type est asiatique”. This statement is accepted as a type species designation for *Blepharella* of the single included species from India, *Blepharella lateralis* Macquart.

*Afrosturmia* Curran, 1927 was treated as a monotypic genus by Crosskey (1980b: 867) but is here placed in synonymy with *Blepharella* Macquart, 1851.

***abana*** (Curran, 1927).—Afrotropical: Angola, Tanzania.

*Sturmia abana* Curran, 1927f: 122. Holotype male (BMNH). Type locality: Tanzania, Morogoro.

***alacris*** (Curran, 1927).—Afrotropical: Malawi, Nigeria, Tanzania.

*Sturmia alacris* Curran, 1927f: 123. Holotype male (BMNH). Type locality: Tanzania, Morogoro.

***analis*** (Curran, 1927).—Afrotropical: D.R. Congo, Kenya, Somalia, Tanzania, Zimbabwe.

*Sturmia analis* Curran, 1927f: 120. Holotype male (BMNH). Type locality: Kenya, Narok [as “Narok, Masai Reserve”, ca. 1°5′S 35°52′E].

***arrogans*** (Curran, 1927).—Afrotropical: D.R. Congo.

*Sturmia arrogans* Curran, 1927c: 16. Holotype male (AMNH). Type locality: D.R. Congo, Orientale, Kisangani [as “Stanleyville”].

***atricauda*** Mesnil, 1970.—Afrotropical: Zimbabwe.

Blepharella (Congochrysosoma) atricauda Mesnil, 1970b: 97. Holotype male (CNC). Type locality: Zimbabwe, Hurungwe [as “Urungwe”], Gota Gota.

***aurifrons*** (Villeneuve, 1916).—Afrotropical: D.R. Congo, Kenya, Malawi, Sierra Leone, South Africa, Tanzania, Uganda.

Sturmia (Crossocosmia) aurifrons Villeneuve, 1916c: 475. Syntypes, males and females (SAMC [1 male examined by JEOH], other unspecified collections [as “etc.”]). Type localities: Malawi (Mt. Mulanje [as “Mt. Mlanje”]), Sierra Leone, South Africa (KwaZulu-Natal, Mfongosi [as “M’fongosi, Zululand”, ca. 28°43′S 30°49′E]), and Uganda.

***bicolor*** (Mesnil, 1968).—Afrotropical: C.A. Republic, D.R. Congo.

*Pujolina bicolor* Mesnil, 1968b: 3. Holotype female (MNHN). Type locality: C.A. Republic.

***carbonata*** Mesnil, 1952.—Afrotropical: D.R. Congo.

Blepharella (Blepharella) setigera carbonata Mesnil, 1952c: 235. Holotype, unspecified sex [male, see Cooper and O’Hara 1996: 18] (CNC). Type locality: D.R. Congo, Nord-Kivu, Kabasha [Escarpment], “Chambi” [probably Tshambi, ca. 0°44′S 29°13′E].

***chionaspis*** (Bezzi, 1908).—Afrotropical: D.R. Congo.

*Winthemia chionaspis* Bezzi, 1908c: 382. Holotype male (?IRSNB). Type locality: D.R. Congo, Orientale [as “Haut-Congo”].

***confusa*** Mesnil, 1952.—Afrotropical: South Africa.

Blepharella (Blepharella) setigera confusa Mesnil, 1952c: 235. Holotype male (CNC). Type locality: South Africa.

Note: Mesnil (1952c: 235) gave the type locality of *Blepharella setigera confusa* as South Africa. The holotype has a label with a place name but the name is partly obscured by a spot of black ink and is unreadable.

***erebiae*** Mesnil, 1970.—Afrotropical: Malawi.

Blepharella (Congochrysosoma) erebiae Mesnil, 1970b: 96. Holotype female (CNC). Type locality: Malawi, Mt. Mulanje [as “Mt. Mlanje”].

***fallaciosa*** Mesnil, 1970.—Afrotropical: Uganda.

Blepharella (Congochrysosoma) fallaciosa Mesnil, 1970b: 96. Holotype male (CNC). Type locality: Uganda, Entebbe.

***fascipes*** (Villeneuve, 1943).—Afrotropical: D.R. Congo, Ethiopia, South Africa.

*Sturmia fascipes* Villeneuve, 1943a: 37. Holotype male (CNC). Type locality: D.R. Congo, Katanga, Sankisia.

***fuscicosta*** (Curran, 1927).—Afrotropical: D.R. Congo, Ghana, Guinea, Malawi, Uganda.

Sturmia (Crossocosmia) fuscicosta Curran, 1927a: 10. Holotype male (AMNH). Type locality: D.R. Congo, Orientale, Kisangani [as “Stanleyville”].

***fuscipennis*** Mesnil, 1952.—Afrotropical: D.R. Congo.

Blepharella (Blepharella) fuscipennis Mesnil, 1952c: 235. Holotype male (CNC). Type locality: D.R. Congo, Orientale, Penghe [near Mambasa, 1°22′24″N 29°4′34″E].

***grandis*** (Curran, 1927).—Afrotropical: D.R. Congo.

*Sturmia grandis* Curran, 1927a: 13. Holotype male (AMNH). Type locality: D.R. Congo, Orientale, Kisangani [as “Stanleyville”].

***haemorrhoa*** Mesnil, 1970.—Afrotropical: Madagascar.

Blepharella (Congochrysosoma) haemorrhoa Mesnil, 1970b: 95. Holotype male (MNHN). Type locality: Madagascar, Toliara, Andronobe.

***hova*** Mesnil, 1952.—Afrotropical: Madagascar, South Africa.

Blepharella (Blepharella) hova Mesnil, 1952c: 235. Holotype male (MNHN). Type locality: Madagascar, “Merinon” [not located].

***imitator*** (Curran, 1927).—Afrotropical: D.R. Congo, Uganda.

*Sturmia imitator* Curran, 1927a: 13. Holotype male (AMNH). Type locality: D.R. Congo, Orientale, Kisangani [as “Stanleyville”].

***instabilis*** (Curran, 1927).—Afrotropical: Malawi, South Africa.

*Sturmia instabilis* Curran, 1927f: 124. Holotype male (SANC). Type locality: South Africa, KwaZulu-Natal, Port Shepstone.

***intensica*** (Curran, 1927).—Afrotropical: D.R. Congo.

Sturmia (Crossocosmia) intensica Curran, 1927a: 17. Lectotype male (AMNH), by designation of Arnaud (1963: 129). Type locality: D.R. Congo, Orientale, Kisangani [as “Stanleyville”].

***laetabilis*** (Curran, 1927).—Afrotropical: D.R. Congo, Ghana, Nigeria, Sierra Leone.

*Sturmia laetabilis* Curran, 1927f: 112, 114. Syntypes, 3 males and 2 females (AMNH). Type locality: D.R. Congo, Orientale, Kisangani [as “Stanleyville”].

Note: Authorship of *Sturmia laetabilis* was attributed to Villeneuve (1933: 279) by Crosskey (1980b: 868). However, the characters given for *Sturmia laetabilis* by Curran (1927f) in his key to the African species of *Sturmia* (pp. 112 [male], 114 [female]) validate the name from this work. Curran (1927f: 126) cited the author as “Villeneuve (in litt.?)”, but the descriptive details given for this species were his own: “I have several specimens from the Belgian Congo determined by Villeneuve, but have seen no description”. Curran (1928b) keyed *Sturmia laetabilis* (pp. 389, 391) and treated it in his text (p. 394), again with Villeneuve as author, and cited 3 males and 2 females from “Stanleyville”. These specimens are assumed to be the same as those examined by Curran (1927f) and thus are accepted as the original syntypes of *Sturmia laetabilis*. Villeneuve (1933: 279) gave characters to separate “*Sturmia rubricosa* n. sp.” and “*Sturmia laetabilis*” but interestingly did not name the latter as a new species nor cite it with an author’s name. No locality data was provided by Villeneuve for *Sturmia laetabilis*. The distribution of *Blepharella laetabilis* (Curran) is recognized here as the type locality in D.R. Congo and the three countries listed for the species by Crosskey (1980b): Ghana, Nigeria, and Sierra Leone.

***lodosi*** Mesnil, 1968.—Afrotropical: Ghana.

Blepharella (Congochrysosoma) lodosi Mesnil, 1968b: 1. Holotype male (CNC). Type locality: Ghana, Tafo [suburb of Kumasi].

***melita*** (Curran, 1927).—Afrotropical: D.R. Congo.

*Sturmia melita* Curran, 1927c: 12. Holotype male (AMNH). Type locality: D.R. Congo, Orientale, Kisangani [as “Stanleyville”].

***neglecta*** Mesnil, 1968.—Afrotropical: D.R. Congo.

Blepharella (Congochrysosoma) neglecta Mesnil, 1968b: 2. Holotype male (CNC). Type locality: D.R. Congo, Nord-Kivu, Walikale [ca. 1°25′S 28°00′E].

***oldi*** Mesnil, 1952.—Afrotropical: Malawi.

Blepharella (Blepharella) oldi Mesnil, 1952c: 235. Holotype male (CNC). Type locality: Malawi, “Ruo” (“Aluona Ruo Dist” according to label data, Cooper and O’Hara 1996: 18; likely somewhere in the Shire Valley of the former Ruo District, ca. 17°S 35°E [given as Ruo, Tanzania by O’Hara 1996: 150 and Cooper and O’Hara 1996: 18, in error]).

***orbitalis*** (Curran, 1927).—Afrotropical: Ghana. **Comb. n.**

*Afrosturmia orbitalis* Curran, 1927f: 127. Holotype male (BMNH). Type locality: Ghana, Ashanti.

Note: *Afrosturmia orbitalis* Curran, 1927 was treated as the sole species of *Afrosturmia* by Crosskey (1980b: 867) but is moved here to *Blepharella* Macquart, 1851.

***pellucida*** Mesnil, 1970.—Afrotropical: D.R. Congo.

Blepharella (Congochrysosoma) pellucida Mesnil, 1970b: 98. Holotype male (CNC). Type locality: D.R. Congo, Équateur, Lulonga [ca. 0°37′N 18°22′E].

***perfida*** Mesnil, 1970.—Afrotropical: D.R. Congo.

Blepharella (Congochrysosoma) perfida Mesnil, 1970b: 96. Holotype male (CNC). Type locality: D.R. Congo, Katanga, Kafakumba [ca. 9°41′N 23°46′E].

***picturata*** (Curran, 1927).—Afrotropical: Kenya, Uganda.

*Sturmia picturata* Curran, 1927f: 122. Holotype female (BMNH). Type locality: Uganda, “Kukedi” [not located].

***rex*** (Curran, 1927).—Afrotropical: D.R. Congo, Tanzania, Uganda.

*Sturmia rex* Curran, 1927a: 14. Holotype male (AMNH). Type locality: D.R. Congo, Orientale, Kisangani [as “Stanleyville”].

***rubricosa*** (Villeneuve, 1933).—Afrotropical: Malawi.

*Sturmia rubricosa* Villeneuve, 1933: 279. Syntypes, males (“plusieurs”) and 1 female (not located). Type locality: Malawi.

***ruficauda*** Mesnil, 1952.—Afrotropical: South Africa.

Blepharella (Blepharella) setigera ruficauda Mesnil, 1952c: 235. Holotype male (CNC). Type locality: South Africa, Gauteng, Sydenham.

***setifacies*** (Curran, 1927).—Afrotropical: D.R. Congo, Uganda.

*Sturmia setifacies* Curran, 1927c: 12. Holotype male (AMNH). Type locality: D.R. Congo, Orientale, Kisangani [as “Stanleyville”].

*Sturmia femineum* Curran, 1927c: 14. Holotype female (AMNH). Type locality: D.R. Congo, Orientale, Kisangani [as “Stanleyville”].

*Winthemia orbitalis* Villeneuve, 1934d: 68 (junior secondary homonym of *Afrosturmia orbitalis* Curran, 1927). Holotype male (CNC). Type locality: D.R. Congo, Nord-Kivu, “Moko Lesse” (“Moko” is “Moho” on the locality label of the holotype, Cooper and O’Hara 1996: 77) [Lesse at ca. 0°45′N 29°46′E, Moho (or Moko) is presumed to be nearby].

Blepharella (Congochrysosoma) erronea Mesnil, 1970b: 95 (*nomen novum* for *Winthemia orbitalis* Villeneuve, 1934).

Note: The relative priority of *Sturmia setifacies* Curran, 1927 and *Sturmia femineum* Curran, 1927, when the two are treated as synonyms, was established by Crosskey (1980b: 868), as the First Reviser (Article 24.2.2 of the *Code*, ICZN 1999).

***setigera*** (Corti, 1895).—Afrotropical: “widespread Afrotrop. Reg.” (Crosskey 1980b: 869), including D.R. Congo, Ethiopia, Kenya, Malawi, Nigeria, Sierra Leone, Uganda. Palaearctic: M. East (M. East [Iran, Zeegers and Majnon Jahromi 2015: 539]).

*Podomyia setigera* Corti, 1895: 135. Type(s), male (?MCSN). Type locality: Ethiopia, Jubba River, “Arussi Galla, Ganale Guddà” [most likely a valley of the upper Ganale River, a tributary of the Jubba River on the eastern edge of the Arussi and Bale Mountains, ca. 7°0′N 40°30′E].

*setigena*. Incorrect subsequent spelling of *setigera* Corti, 1895 (Zeegers and Majnon Jahromi 2015: 540, etc.).

***seydeli*** (Mesnil, 1949).—Afrotropical: D.R. Congo.

*Zygobothria seydeli* Mesnil, 1949a: 92. Holotype male (MRAC). Type locality: D.R. Congo, Katanga, Lubumbashi [as “Elisabethville”].

***snyderi*** (Townsend, 1916).—Afrotropical: D.R. Congo, Ghana, Guinea, Kenya, Malawi, Nigeria, Tanzania, Uganda.

*Congochrysosoma snyderi* Townsend, 1916a: 174. Holotype female (USNM). Type locality: D.R. Congo, Kasai-Occidental, Luebo.

*Sturmia currani* Villeneuve, 1933: 279 (named for *Sturmia versatilis* of Curran, 1927f, 1928b, not Villeneuve, 1910a). Syntypes, females (“plusieurs”) (not located). Type localities: D.R. Congo and Malawi.

*Sturmia versatilis* of Curran (1927f: 125, 1928b: 394), not Villeneuve, 1910. Misidentification (Villeneuve 1933: 279).

***vasta*** (Karsch, 1886).—Afrotropical: Angola, Uganda.

*Tachina vasta* Karsch, 1886b: 341. Holotype, unspecified sex [female, examined by JEOH] (ZMHB). Type locality: Angola, Pungo Andongo.

***versatilis*** (Villeneuve, 1910).—Afrotropical: D.R. Congo, Malawi, Nigeria, Sudan. Palaearctic: ?N. Africa (NE. Africa) (see note).

*Sturmia versatilis* Villeneuve, 1910a: 253. Type(s), male (1 male in CNC). Type locality: D.R. Congo (as “Congo”, p. 249).

*versatalis*. Incorrect subsequent spelling of *versatilis* Villeneuve, 1910 (Curran 1928b: 389).

Note: Villeneuve (1910a) described four species from “Congo”. Curran (1927f: 122) treated one of them (*Sturmia aureiventris* Villeneuve, 1910) as described from D.R. Congo (as “Belgian Congo”), and used “Belgian Congo” and “Congo” interchangeably in this work and some others. We think it likely that Villeneuve (1910a), like Curran, used “Congo” in the sense of present-day D.R. Congo. However, Crosskey (1980b) interpreted Villeneuve’s Congo as the present-day country of Congo. Villeneuve’s (1913c: 29) record of *Sturmia versatilis* Villeneuve, 1910 from Egypt, based on a male in BMNH, needs confirmation.

***vivax*** (Curran, 1927).—Afrotropical: D.R. Congo, Nigeria.

*Sturmia vivax* Curran, 1927a: 15. Holotype male (AMNH). Type locality: D.R. Congo, Orientale, Kisangani [as “Stanleyville”].

***vulnerata*** (Curran, 1927).—Afrotropical: D.R. Congo.

*Sturmia vulnerata* Curran, 1927c: 13. Holotype male (AMNH). Type locality: D.R. Congo, Orientale, Kisangani [as “Stanleyville”].

***xanthaspis*** Mesnil, 1970.—Afrotropical: South Africa.

Blepharella (Congochrysosoma) xanthaspis Mesnil, 1970b: 97. Holotype male (CNC). Type locality: South Africa, KwaZulu-Natal, Eshowe [as “Eshova”, misprint].

##### Genus *BLEPHARELLINA* Mesnil, 1952

*BLEPHARELLINA* Mesnil, 1949b: 104. *Nomen nudum* (proposed after 1930 without designation of type species; no included species) (see Evenhuis and O’Hara 2008: 65).

*BLEPHARELLINA* Mesnil, 1950c: 105 (as subgenus of *Blepharella* Macquart, 1851). *Nomen nudum* (proposed after 1930 without designation of type species; no included species) (see Evenhuis and O’Hara 2008: 65).

***BLEPHARELLINA*** Mesnil, 1952c: 234 (as subgenus of *Blepharella* Macquart, 1851). Type species: Blepharella (Blepharellina) picta Mesnil, 1952, by monotypy (see Evenhuis and O’Hara 2008: 65).

***picta*** (Mesnil, 1952).—Afrotropical: Nigeria.

Blepharella (Blepharellina) picta Mesnil, 1952: 234. Holotype, unspecified sex [female, see Cooper and O’Hara 1996: 18] (CNC). Type locality: Nigeria, Oshogbo.

##### Genus *BLEPHARIPA* Rondani, 1856

***BLEPHARIPA*** Rondani, 1856: 71. Type species: *Erycia ciliata* Macquart, 1834 (as “*Masicera ciliata* Macq.”) (= *Tachina pratensis* Meigen, 1824), by original designation.

*BLEPHARIPODA* Brauer & Bergenstamm, 1889: 96 [also 1890: 28] (junior homonym of *Blepharipoda* Randall, 1840). Type species: *Nemoraea scutellata* Robineau-Desvoidy, 1830 (= *Tachina pratensis* Meigen, 1824), by monotypy.

*pratensis* (Meigen, 1824).—Misidentification, not Afrotropical [known from Palaearctic Region].

Note: An unknown species was recorded as “*Sturmia scutellata*, Desvoidy” (originally described as *Nemoraea scutellata* Robineau-Desvoidy, 1830, currently a synonym of *Tachina pratensis* Meigen, 1824) from Uganda by Curran (1927f: 123). Misidentification (not recorded from the Afrotropical Region by Crosskey 1980b, Herting and Dely-Draskovits 1993: 249).

##### Genus *BRACHYCHAETOIDES* Mesnil, 1970

***BRACHYCHAETOIDES*** Mesnil, 1970b: 109 (as subgenus of *Chlorolydella* Townsend, 1933). Type species: Chlorolydella (Brachychaetoides) varipes Mesnil, 1970 (= *Archiclops africanum* Mesnil, 1968), by original designation.

Note: *Brachychaetoides* Mesnil, 1970 was treated as a synonym of *Chlorolydella* Townsend, 1933 by Crosskey (1980b: 877). It was later recognized as a genus by Crosskey (1984: 201, 295) with single species *Brachychaetoides africanum* (Mesnil, 1968).

***africanum*** (Mesnil, 1968).—Afrotropical: Tanzania.

*Archiclops africanum* Mesnil, 1968b: 6. Holotype male (SMNS). Type locality: Tanzania, southwest side of Mt. Kilimanjaro [as “Kilimandjaro”], 3500m.

Chlorolydella (Brachychaetoides) varipes Mesnil, 1970b: 109. Holotype male (MNHN). Type locality: Tanzania, Mt. Kilimanjaro [as “Kilimandjaro”], 2800–3000m.

Note: *Chlorolydella varipes* Mesnil, 1970 was synonymized with *Archiclops africanum* Mesnil, 1968 by Crosskey (1984: 201, 295). *Archiclops africanum* was earlier treated as a species of *Gymnophryxe* Villeneuve, 1922 by Crosskey (1980b: 878).

***violacea*** (Curran, 1927).—Afrotropical: Kenya. **Comb. n.**

*Campylochaeta violacea* Curran, 1927d: 337. Holotype male (BMNH). Type locality: Kenya, Kabete [ca. 1°16′S 36°43′E, near Nairobi].

Note: *Campylochaeta violacea* Curran, 1927 was treated as a species of *Chlorolydella* Townsend, 1933 by Crosskey (1980b: 877, 1984: 286) but is moved here to *Brachychaetoides* Mesnil, 1970.

Undescribed spp.: Kenya (TAU), Malawi (TAU), South Africa (MZUR, NMB) (examined by PC).

##### Genus *CADURCIA* Villeneuve, 1926

***CADURCIA*** Villeneuve, 1926c: 243. Type species: *Masicera casta* Rondani, 1861, by subsequent designation of Townsend (1936b: 256) [Palaearctic].

*ARGYROPHYLACOIDES* Townsend, 1933: 477. Type species: *Degeeria zetterstedtii* Karsch, 1886, by original designation.

***auratocauda*** (Curran, 1934).—Afrotropical: Côte d’Ivoire, D.R. Congo, Ghana, Nigeria, Sierra Leone.

*Sturmia auratocauda* Curran, 1934b: 2. Holotype male (BMNH). Type locality: Nigeria, Ibadan.

***borbonensis*** Villeneuve, 1926.—Afrotropical: Réunion.

*Cadurcia borbonensis* Villeneuve, 1926c: 245. Syntypes, 4 males (1 male in CNC, 1 male in NHMW). Type localities: Réunion and “un ♂ de la collection Strobl (d’où?)”.

***depressa*** Villeneuve, 1926.—Afrotropical: D.R. Congo.

*Cadurcia depressa* Villeneuve, 1926c: 244. Syntypes, 2 males (1 male in CNC). Type locality: D.R. Congo, Katanga, Kayombo.

***fascicauda*** (Curran, 1934).—Afrotropical: South Africa.

*Sturmia fascicauda* Curran, 1934b: 3. Holotype male (SANC). Type locality: South Africa, Eastern Cape, East London.

***lucens*** Villeneuve, 1926.—Afrotropical: Malawi, Mauritius, Nigeria, South Africa, Uganda.

*Cadurcia lucens* Villeneuve, 1926c: 244. Lectotype male (BMNH), by designation of Crosskey (1976: 265). Type locality: Nigeria, Ilorin.

*Masicera casta* of authors (e.g., Verbeke 1970: 272, as “*Cadurcia casta*”; Crosskey 1980b: 860, under “*Cadurcia lucens*”), not Rondani, 1861. Misidentification (see note).

Note: Crosskey (1980b: 869) included *Cadurcia vanderwulpi* Baranov, 1938 (described from India) in synonymy with *Cadurcia lucens* Villeneuve, 1926, but we have followed Herting and Dely-Draskovits (1993: 245) in treating the former as a valid name. Similarly, we have followed Herting and Dely-Draskovits (1993: 243) in treating *Cadurcia casta* (Rondani, 1861) as a strictly Palaearctic species and not as a questionable synonym of *Cadurcia lucens* as listed by Crosskey (1980b: 869). If *Cadurcia casta* and *Cadurcia lucens* are conspecific then the former name has priority.

***mesnili*** Verbeke, 1962.—Afrotropical: D.R. Congo.

*Cadurcia mesnili* Verbeke, 1962b: 53. Holotype male (IRSNB). Type locality: D.R. Congo, Nord-Kivu, Parc National des Virunga [as “P.N.A”, former Parc National Albert], Goma-Sake route, Buheno.

***plutellae*** van Emden, 1942.—Afrotropical: Kenya.

*Cadurcia plutellae* van Emden, 1942: 223. Holotype male (BMNH). Type locality: Kenya, Nairobi.

***semiviolacea*** Villeneuve, 1926.—Afrotropical: South Africa.

*Cadurcia semiviolacea* Villeneuve, 1926c: 245 (as “*semiviolacea* (B. B. i. litt.)”). Syntypes, 2 females (NHMW). Type locality: South Africa, Western Cape, Cape of Good Hope [as “Cap”].

Note: Villeneuve (1926c: 245) wrote that *Cadurcia semiviolacea* was “Représenté par 2 ♀♀ dans la collection v. Winthem du Muséum de Vienne”. Villeneuve began the next paragraph with “Ces deux ♂♂”, which was a lapsus for two females. The two female syntypes in NHMW were examined by JEOH.

***versicauda*** (Curran, 1934).—Afrotropical: Angola, South Africa, Tanzania.

*Sturmia versicauda* Curran, 1934b: 4. Holotype male (BMNH). Type locality: South Africa, KwaZulu-Natal, Weenen [as “Wernen”, ca. 28°51′S 30°4′E].

***vinsoni*** Mesnil, 1952.—Afrotropical: Mauritius.

*Cadurcia vinsoni* Mesnil, 1952c: 214. Holotype, unspecified sex (BMNH). Type locality: Mauritius, Chebel.

***zetterstedtii*** (Karsch, 1886).—Afrotropical: Angola, Congo, Guinea, Nigeria, Senegal, Yemen.

*Degeeria zetterstedtii* Karsch, 1886b: 342. Holotype, unspecified sex [female, examined by JEOH] (ZMHB). Type locality: Angola, Pungo Andongo.

*Sturmia albicauda* Curran, 1934b: 3. Holotype male (AMNH). Type locality: Congo, “on board ship off Loango”.

*albocauda*. Incorrect original spelling of *albicauda* Curran, 1934 (Curran 1934b: 1).

Note: There are two original spellings for *Sturmia albicauda* in Curran (1934b): *albocauda* in the key (p. 1) and *albicauda* in the species header (p. 3). The correct original spelling was selected as *albicauda* by Crosskey (1980b: 869), as the First Reviser (Article 24.2.3 of the *Code*, ICZN 1999).

Possibly undescribed spp.: Yemen, as “Cadurcia sp. 1 cf. fascicauda” and “*Cadurcia* sp. 2” (Zeegers 2007: 378).

##### Genus *CHAETOSTURMIA* Villeneuve, 1915

***CHAETOSTURMIA*** Villeneuve, 1915b: 193. Type species: *Chaetosturmia barbata* Villeneuve, 1915, by monotypy.

***barbata*** Villeneuve, 1915.—Afrotropical: Madagascar.

*Chaetosturmia barbata* Villeneuve, 1915b: 194. Holotype male (NHMW). Type locality: Madagascar.

##### Genus *CHLOROLYDELLA* Townsend, 1933

***CHLOROLYDELLA*** Townsend, 1933: 473. Type species: *Chlorolydella caffrariae* Townsend, 1933, by original designation.

*CHLOROPHRYNO* Townsend, 1933: 478. Type species: *Gymnochaeta glauca* Karsch, 1886 (as “*Gymnocheta glauca*”), by original designation.

Note: The relative priority of *Chlorolydella* Townsend, 1933 and *Chlorophryno* Townsend, 1933, when the two are treated as synonyms, was established by Mesnil (1954b: 347), as the First Reviser (Article 24.2.2 of the *Code*, ICZN 1999). Mesnil did not mention *Chlorophryno* but effectively synonymized it with *Chlorolydella* by placing its type species, *Gymnochaeta glauca* Karsch, 1886, in *Chlorolydella*.

***bequaerti*** (Curran, 1940).—Afrotropical: Uganda.

*Phorocera bequaerti* Curran, 1940: 6. Holotype female (AMNH). Type locality: Uganda, Behungi [as “Behunge” in error, Arnaud 1963: 124, ca. 1°17′S 29°48′E].

***caffrariae*** Townsend, 1933.—Afrotropical: South Africa, Tanzania, Uganda, Zimbabwe.

*Chlorolydella caffrariae* Townsend, 1933: 474. Holotype male (NHRS). Type locality: South Africa, “Caffraria” (also known as “Kaffraria”, a former region in Eastern Cape).

? *Stomatomya metallica* Villeneuve, 1916c: 475 (junior secondary homonym of *Campylochaeta metallica* Bezzi, 1908 and *Phorocera metallica* Becker, 1909). Syntypes, unspecified number and including at least 1 male (CNC, SAMC [not located by JEOH]). Type localities: South Africa (KwaZulu-Natal, Durban; KwaZulu-Natal, Mfongosi [as “M’fongosi, Zululand”]) and Zimbabwe (Harare [as “Salisbury”]).

***glauca*** (Karsch, 1886).—Afrotropical: Angola, Burundi, Eritrea, Kenya, South Africa (**new record**, NMDA [PC]), Tanzania, Uganda.

*Gymnochaeta glauca* Karsch, 1886b: 339. Syntypes, two specimens of unspecified sex [females, examined by JEOH] (ZMHB). Type locality: Angola, Pungo Andongo (not “West Tanganyika” [i.e., Tanzania] as cited by Townsend 1933: 478, in error).

*Campylochaeta metallica* Bezzi, 1908b: 57. Holotype male (not located, not among the labelled types of Bezzi in MSNM examined by Arnaud 1982). Type locality: Eritrea, near Adi Keyh [also as Adi Kaie and other spellings, published as “Adi Caiè”, ca. 14°51′N 39°22′E].

Note: Townsend (1933: 478) mentioned the “Female holotype” of *Gymnochaeta glauca* Karsch, 1886 in ZMHB but did not restrict the term holotype to one of the two females in the type series and hence did not fix a lectotype.

***metallica*** (Becker, 1909).—Afrotropical: Kenya.

*Phorocera metallica* Becker, 1909a: 117 (junior secondary homonym of *Campylochaeta metallica* Bezzi, 1908; not renamed while *Campylochaeta metallica* is in synonymy with *Chlorolydella glauca* (Karsch, 1886)). Holotype female (MNHN). Type locality: Kenya [as “Afrique orientale anglaise; Escarpment”, interpreted as Kenya by Crosskey 1980b: 877].

Note: The description of *Phorocera metallica* Becker, 1909 was repeated in Becker (1910a: 26) under the heading “*Phorocera metallica*, n. sp. ♀”.

***pallidipes*** (Curran, 1927).—Afrotropical: Kenya.

*Campylochaeta pallidipes* Curran, 1927d: 338. Holotype male (not located). Type locality: Kenya, Kabete [ca. 1°16′S 36°43′E, near Nairobi].

***schistacea*** Mesnil, 1955.—Afrotropical: Rwanda, South Africa.

*Chlorolydella schistacea* Mesnil, 1955: 365. Holotype, unspecified sex [male, see Cooper and O’Hara 1996: 24] (CNC). Type locality: Rwanda, Volcan Visoke [also known as Bisoke; published as “Bishoke”], Kibga, 2400m [ca. 1°29′S 29°31′E].

***trochanterata*** (Villeneuve, 1934).—Afrotropical: South Africa.

*Pales trochanterata* Villeneuve, 1934c: 408. Syntypes, 2 males (not located). Type locality: South Africa.

***venusta*** (Curran, 1928).—Afrotropical: Burundi, Kenya, Tanzania, Uganda.

*Phorocera venusta* Curran, 1928a: 238. Holotype male (BMNH). Type locality: Uganda, Rwenzori Range [as “Mount Ruwenzori”].

##### Genus *DOLICHOCOLON* Brauer & Bergenstamm, 1889

***DOLICHOCOLON*** Brauer & Bergenstamm, 1889: 100 [also 1890: 32]. Type species: *Dolichocolon paradoxum* Brauer & Bergenstamm, 1889, by monotypy.

Note: A world revision of *Dolichocolon* Brauer & Bergenstamm, 1889 was published by Cerretti and Shima (2011).

***africanum*** Mesnil, 1968.—Afrotropical: D.R. Congo, South Africa, Tanzania.

*Dolichocolon africanum* Mesnil, 1968c: 176. Holotype male (CNC). Type locality: D.R. Congo, Nord-Kivu, Rwindi, 1000m [ca. 0°47′S 29°17′E].

***basilewskyi*** Cerretti & Shima, 2011.—Afrotropical: Uganda.

*Dolichocolon basilewskyi* Cerretti & Shima, 2011: 557. Holotype male (MRAC). Type locality: Uganda, Bugiri, 1400m (1°04′N 33°43′E).

***bequaerti*** Cerretti & Shima, 2011.—Afrotropical: D.R. Congo.

*Dolichocolon bequaerti* Cerretti & Shima, 2011: 556. Holotype male (MRAC). Type locality: D.R. Congo, Katanga, Kunda (7°15′S 28°27′E).

***caudatum*** Cerretti & Shima, 2011.—Afrotropical: Senegal.

*Dolichocolon caudatum* Cerretti & Shima, 2011: 561. Holotype male (SMNS). Type locality: Senegal, Simenti (13°02′N 13°18'W), Maribor.

***crosskeyi*** Cerretti & Shima, 2011.—Afrotropical: Angola, Zimbabwe.

*Dolichocolon crosskeyi* Cerretti & Shima, 2011: 565. Holotype male (BMNH). Type locality: Zimbabwe, Chikurubi (17°47′S 31°12′E).

***elegans*** Cerretti & Shima, 2011.—Afrotropical: D.R. Congo.

*Dolichocolon elegans* Cerretti & Shima, 2011: 553. Holotype male (MRAC). Type locality: D.R. Congo, Katanga, Lubumbashi.

***meii*** Cerretti & Shima, 2011.—Afrotropical: Ethiopia.

*Dolichocolon meii* Cerretti & Shima, 2011: 554. Holotype male (MZUR). Type locality: Ethiopia, El Banno, 1250m (4°51′0.05″N 37°23′59.96″E).

***mesnili*** Cerretti & Shima, 2011.—Afrotropical: D.R. Congo.

*Dolichocolon mesnili* Cerretti & Shima, 2011: 560. Holotype male (CNC). Type locality: D.R. Congo, Katanga, Lubumbashi.

***paradoxum*** Brauer & Bergenstamm, 1889.—Afrotropical: D.R. Congo, Mozambique. Palaearctic: Europe (W. Eur., SW. Eur., SC. Eur., SE. Eur.), M. East (all), Transcaucasia.

*Dolichocolon paradoxum* Brauer & Bergenstamm, 1889: 100, 165 [also 1890: 32, 97]. Holotype male [not lectotype male as inferred by O’Hara et al. 2009: 106, see Cerretti and Shima 2011: 555] (NHMW). Type locality: Croatia, Dalmacija [as “Dalmatien”].

Note: Cerretti and Shima (2011: 555–556) redescribed *Dolichocolon paradoxum* Brauer & Bergenstamm, 1889 and reevaluated its distribution. These authors noted that *Dolichocolon paradoxum* had been misidentified from South Africa in Crosskey (1980b: 877) and its presence in eastern Asia as recorded in O’Hara et al. (2009: 106) and elsewhere is suspect (Cerretti and Shima 2011: 556).

***paravicinum*** Cerretti & Shima, 2011.—Afrotropical: Nigeria, South Africa, Yemen.

*Dolichocolon paravicinum* Cerretti & Shima, 2011: 571. Holotype male (RMNH). Type locality: Yemen, 12km northwest of Manākhah (15°04′19″N 43°44′27″E according to Zeegers 2009: 371).

Note: The holotype of *Dolichocolon paravicinum* Cerretti & Shima, 2011 is one of the specimens from Yemen that Zeegers (2007: 384) examined and cited as “*Dolichocolon* sp.”. Specimens of *Dolichocolon paravicinum* from Uganda were misidentified as *Dolichocolon vicinum* Mesnil, 1968 in Crosskey (1980b: 877). *Dolichocolon vicinum* is currently regarded as a strictly Oriental species (Cerretti and Shima 2011: 569–571).

***rude*** Cerretti & Shima, 2011.—Afrotropical: Cameroon, Côte d’Ivoire, D.R. Congo, South Africa.

*Dolichocolon rude* Cerretti & Shima, 2011: 558. Holotype male (NMDA). Type locality: Cameroon, Kassei (10°31′N 14°46′E).

##### Genus *ERYTHROCERA* Robineau-Desvoidy, 1849

*ERYTHROCERA* Robineau-Desvoidy, 1848: 186. *Nomen nudum* (no description or included species).

***ERYTHROCERA*** Robineau-Desvoidy, 1849b: 436. Type species: *Phryno nigripes* Robineau-Desvoidy, 1830, by subsequent designation of Robineau-Desvoidy (1863a: 600, as “*Erythrocera nigripes*, R.-D.”) [Palaearctic].

***doris*** (Curran, 1927).—Afrotropical: D.R. Congo.

*Sturmia doris* Curran, 1927c: 18 (as “*Sturmia* (?) *doris*”). Holotype male (AMNH). Type locality: D.R. Congo, Orientale, Kisangani [as “Stanleyville”].

*Sturmia dorina* Curran, 1927f: 126 (unnecessary *nomen novum* for *Sturmia doris* Curran, 1927).

Note: *Sturmia dorina* Curran, 1927 was proposed for “Sturmia? doris Curran (not Schiner)”. However, this was based on the misidentification of *Tachina doris* Meigen, 1824 by previous authors. Herting (1972: 5) established the true *Tachina doris* Meigen as a junior synonym of *Lydella stabulans* (Meigen, 1824) (see also Herting and Dely-Draskovits 1993: 205 [*doris* Meigen], 248 [*doris* of authors, not Meigen]).

***picta*** (Villeneuve, 1936).—Afrotropical: Nigeria.

Pexomyia (Erythrocera) picta Villeneuve, 1936a: 7. Holotype male (CNC). Type locality: Nigeria, Oshogbo.

***porcula*** Mesnil, 1952.—Afrotropical: Nigeria, Sierra Leone.

*Erythrocera porcula* Mesnil, 1952c: 252. Holotype female (not located). Type locality: northern Nigeria.

##### Genus *GONIA* Meigen, 1803

*SALMACIA* Meigen, 1800: 38. Name suppressed by ICZN (1963: 339).

***GONIA*** Meigen, 1803: 280. Type species: *Gonia bimaculata* Wiedemann, 1819, by subsequent designation of Sabrosky and Arnaud (1965: 1075).

***bimaculata*** Wiedemann, 1819.—Afrotropical: “widespread mainland Afrotrop. Reg. (excl. W. Afr.)” (Crosskey 1980b: 875), including Malawi, South Africa, Uganda, Yemen. Palaearctic: C. Asia, Europe (E. Eur., SW. Eur., SC. Eur., SE. Europe, Turkey), M. East (all), N. Africa (Canary Is., Madeira), Pal. China, Transcaucasia. Oriental: Orien. China.

*Gonia bimaculata* Wiedemann, 1819: 25. Type(s), female (1 female in NHMW, 1 syntype in ZMUC [Zimsen 1954: 23]). Type locality: South Africa, Western Cape, Cape of Good Hope [as “Prom. bon. sp.” = “Promontorium Bonae Spei”].

Note: *Gonia bimaculata* Wiedemann, 1819 was described from an unspecified number of females, but certainly more than one because Wiedemann (1930: 344) later wrote “In Westermann’s und meiner Sammlung”. A female in NHMW (examined by JEOH) is recognizable as a syntype by its “*bimaculata* Coll. Wiedem.” label and a second label giving the species name, locality (“C. b. sp.”), and collector (“Westermann”). A third label, blue with only “Typus” handwritten on it, appears to have been written by Villeneuve.

***rubriventris*** Macquart, 1851.—Afrotropical: South Africa.

*Gonia rubriventris* Macquart, 1851b: 150 [also 1851b: 177]. Lectotype female (BMNH), by fixation of Crosskey (1971: 270) (examination of “Holotype ♀” from Cape of Good Hope in BMNH is regarded as a lectotype fixation). Type locality: South Africa, Western Cape, Cape of Good Hope [as “Cap de Bonne-Espérance”].

Note: Crosskey (1984: 285) discussed the differences between *Gonia rubriventris* Macquart, 1851 and *Gonia bimaculata* Wiedemann, 1819 and suggested they are “possibly synonymous”.

##### Genus *GONIOPHTHALMUS* Villeneuve, 1910

***GONIOPHTHALMUS*** Villeneuve *in* Becker, 1910b: 145 [also 1910b: 15]. Type species: *Goniophthalmus simonyi* Villeneuve, 1910, by monotypy.

***halli*** Mesnil, 1956.—Afrotropical: Botswana, Cape Verde, Kenya, Namibia, Sudan, Tanzania, U.A. Emirates, Yemen, Zimbabwe. Palaearctic: M. East (all). Oriental: India.

*Goniophthalmus halli* Mesnil, 1956c: 548. Holotype male (published as BMNH but probably not deposited there according to Crosskey 1976: 244). Type locality: Zimbabwe, Mazoe.

***simonyi*** Villeneuve. 1910.—Afrotropical: Yemen.

*Goniophthalmus simonyi* Villeneuve *in* Becker, 19l0b: 145 [also 1910b: 15]. Lectotype male (NHMW), by fixation of Townsend (1941: 34) (mention of “Ht male” from “Ras Shoab, Sokotra” in NHMW is regarded as a lectotype fixation for the single male in the type series). Type locality: Yemen, Suquţrá [as “Sokotra”] (“Ras Shoab” according to Townsend 1941: 34 and label data of lectotype [examined by JEOH]).

Undescribed sp.: Kenya (MZUR, examined by PC).

##### Genus *HYSTRICEPHALA* Macquart, 1846

***HYSTRICEPHALA*** Macquart, 1846: 282 [also 1846: 154]. Type species: *Hystricephala nigra* Macquart, 1846, by monotypy.

***nigra*** Macquart, 1846.—Afrotropical: South Africa.

*Hystricephala nigra* Macquart, 1846: 283 [also 1846: 155]. Holotype male (“presumed lost”, Crosskey 1971: 272). Type locality: South Africa, “Cafrerie” (also as “Kaffraria”; probably referring to an area now in the southeastern part of Eastern Cape).

##### Genus *IGNEOMYIA* Mesnil, 1950

*IGNEOMYIA* Mesnil, 1949b: 103 (as subgenus of *Congochrysosoma* Townsend, 1916). *Nomen nudum* (proposed after 1930 without designation of type species; no included species) (see Evenhuis and O’Hara 2008: 66).

***IGNEOMYIA*** Mesnil, 1950c: 105, 108 (as subgenus of *Congochrysosoma* Townsend, 1916). Type species: Pexopsis (Ugimeigenia) ignea Mesnil, 1944, by monotypy (see Evenhuis and O’Hara 2008: 66).

***ferruginea*** Mesnil, 1970.—Afrotropical: Madagascar.

*Igneomyia ferruginea* Mesnil, 1970b: 107. Holotype male (MNHN). Type locality: Madagascar, Antsiranana, Montagne d’Ambre [Parc National, ca. 12°36′S 49°8′E].

***ignea*** (Mesnil, 1944).—Afrotropical: Madagascar.

Pexopsis (Ugimeigenia) ignea Mesnil, 1944: 10. Holotype male (MNHN). Type locality: Madagascar, Toliara, Bekily.

##### Genus *KUWANIMYIA* Townsend, 1916

***KUWANIMYIA*** Townsend, 1916d: 319. Type species: *Kuwanimyia conspersa* Townsend, 1916, by original designation [Palaearctic].

Note: *Kuwanimyia* Townsend, 1916 was revised by Cerretti (2009b).

***afra*** Cerretti, 2009.—Afrotropical: Namibia.

*Kuwanimyia afra* Cerretti, 2009b: 56. Holotype male (BMNH). Type locality: Namibia, 23 miles southwest of Grootfontein, Rietfontein.

***atra*** Cerretti, 2009.—Afrotropical: Namibia, Nigeria.

*Kuwanimyia atra* Cerretti, 2009b: 57. Holotype male (BMNH). Type locality: Nigeria, Samaru.

***capensis*** Cerretti, 2009.—Afrotropical: South Africa.

*Kuwanimyia capensis* Cerretti, 2009b: 58. Holotype female (NMDA). Type locality: South Africa, Eastern Cape, Fort Beaufort.

##### Genus *LYDELLINA* Villeneuve, 1916

***LYDELLINA*** Villeneuve, 1916c: 490. Type species: hereby fixed under Article 70.3.2 of the *Code* (ICZN 1999) as *Lydellina villeneuvei* Townsend, 1933, misidentified as *Masicera caffra* Macquart, 1846 in the fixation by monotypy of Villeneuve (1916c).

***anorbitalis*** Mesnil, 1970.—Afrotropical: Benin, Tanzania, Uganda.

*Lydellina anorbitalis* Mesnil, 1970b: 99. Holotype male (CNC). Type locality: Benin [as “Dahomey”], Agouagon [ca. 7°59′N 2°18′E].

***distincta*** Mesnil, 1970.—Afrotropical: Madagascar.

*Lydellina distincta* Mesnil, 1970b: 100. Holotype male (MNHN). Type locality: Madagascar, Toliara, Bekily.

***frontalis*** Mesnil, 1970.—Afrotropical: Ghana.

*Lydellina frontalis* Mesnil, 1970b: 100. Holotype male (CNC). Type locality: Ghana, Aburi.

***umbripennis*** Mesnil, 1970.—Afrotropical: D.R. Congo.

*Lydellina umbripennis* Mesnil, 1970b: 100. Holotype male (CNC). Type locality: D.R. Congo, Équateur, Eala.

***villeneuvei*** Townsend, 1933.—Afrotropical: D.R. Congo, Malawi, South Africa.

*Lydellina villeneuvei* Townsend, 1933: 469 (named for *caffra* of Villeneuve, 1916c, etc., not Macquart, 1846). Holotype female (SAMC). Type locality: South Africa, KwaZulu-Natal, Durban.

*Masicera caffra* of authors (e.g., Villeneuve 1916c: 490, Curran 1928b: 397, Verbeke 1962b: 50, all three as “*Lydellina caffra*”), not Macquart, 1846. Misidentification (Crosskey 1980b: 879).

##### Genus *MINTHOSOMA* Zeegers, 2007

***MINTHOSOMA*** Zeegers, 2007: 389. Type species: *Minthosoma janus* Zeegers, 2007, by original designation.

Note: We have followed Zeegers (2007: 389) in tentatively placing this genus in the Goniini (“genus is likely to be close to *Baumhaueria* [Meigen]”).

***janus*** Zeegers, 2007.—Afrotropical: Yemen.

*Minthosoma janus* Zeegers, 2007: 390. Holotype female (RMNH). Type locality: Yemen, Seyun (15°56′36″N 48°47′36″E).

##### Genus *MYXOGAEDIA* Mesnil, 1956

*PRETORIANA* Curran, 1938: 7 (junior homonym of *Pretoriana* Uvarov, 1922). Type species: *Pretoriana setosa* Curran, 1938, by original designation.

***MYXOGAEDIA*** Mesnil, 1956a: 497. Type species: *Myxarchiclops maculosus* Villeneuve, 1916, by original designation.

*GAUTENGICESA* Koçak & Kemal, 2010: 157 (*nomen novum* for *Pretoriana* Curran, 1938).

Note: *Myxogaedia* Mesnil, 1956 was recognized as the valid name for this genus by O’Hara (2011: 60–61) after *Gautengicesa* Koçak & Kemal, 2010 was proposed as a replacement name for *Pretoriana* Curran, 1938.

***maculosa*** (Villeneuve, 1916).—Afrotropical: South Africa.

*Myxarchiclops maculosus* Villeneuve, 1916c: 496 (as “*Myxarchiclops* (?) *maculosus*”). Holotype female (CNC). Type locality: South Africa, Northern Cape, Springbok [as “Springbokfontein”].

***setosa*** (Curran, 1938).—Afrotropical: South Africa.

*Pretoriana setosa* Curran, 1938: 7. Holotype male (SANC). Type locality: South Africa, Gauteng, Pretoria.

Undetermined sp. (nr. *Myxarchiclops maculosa* (Villeneuve)): Namibia (MZUR, examined by PC).

##### Genus *MYXOPHRYXE* Cerretti & O’Hara, gen. n.

***MYXOPHRYXE*** Cerretti & O’Hara, **gen. n.** Type species: *Phorocera longirostris* Villeneuve, 1938, by present designation.

Note: This new genus and the three new species below are described in the New Taxa of Afrotropical Tachinidae section.

***longirostris*** (Villeneuve, 1938).—Afrotropical: South Africa. **Comb. n.**

*Phorocera longirostris* Villeneuve, 1938c: 2. Holotype male (not located; male specimen in CNC labelled by Mesnil as “TYPE” and cited as such by Cooper and O’Hara 1996: 62 is not from the type locality and is not the holotype). Type locality: South Africa, “Colonie du Cap” (former Cape Province, corresponding to the present-day Western Cape, Eastern Cape, Northern Cape, and North West [in part] provinces).

*Phorocera majestica* Curran, 1940: 10. Holotype male (SANC). Type locality: South Africa, KwaZulu-Natal, New Hanover. **Syn. n.**

Note: *Phorocera longirostris* Villeneuve, 1938 and *Phorocera majestica* Curran, 1940 were treated as species of *Pretoriana* Curran, 1938 (the valid name of which is now *Myxogaedia* Mesnil, 1956) by Crosskey (1980b: 879). These nominal species are moved here to *Myxophryxe* gen. n., with *Phorocera longirostris* as the valid name and *Phorocera majestica* in synonymy. This species is redescribed in the New Taxa of Afrotropical Tachinidae section.

***murina*** Cerretti & O’Hara, **sp. n.**—Afrotropical: South Africa.

*Myxophryxe murina* Cerretti & O’Hara, **sp. n.** Holotype male (NMB). Type locality: South Africa, Western Cape, De Vasselot Natural Reserve (33°58.194′S 23°32.193′E).

***regalis*** Cerretti & O’Hara, **sp. n.**—Afrotropical: South Africa.

*Myxophryxe regalis* Cerretti & O’Hara, **sp. n.** Holotype male (NMB). Type locality: South Africa, KwaZulu-Natal, Royal Natal National Park, Thendele, 1600m (28°42.378′S 28°56.083′E).

***satanas*** Cerretti & O’Hara, **sp. n.**—Afrotropical: South Africa.

*Myxophryxe satanas* Cerretti & O’Hara, **sp. n.** Holotype male (MZUR). Type locality: South Africa, Western Cape, Gamkaskloof (Die Hel), 336m (33°21′49.60″S 21°37′40.97″E).

##### Genus *NEALSOMYIA* Mesnil, 1939

***NEALSOMYIA*** Mesnil, 1939d: 31. Type species: Exorista (Alsomyia) triseriella Villeneuve, 1929, by original designation [Palaearctic].

Note: A world revision of *Nealsomyia* Mesnil, 1939 was published by Cerretti (2005).

***chloronitens*** (Mesnil, 1977).—Afrotropical: Madagascar. **Comb. n.**

*Alsomyia chloronitens* Mesnil, 1977b: 187. Holotype male (MNHN). Type locality: Madagascar, Ambohitantely [Réserve Spéciale, ca. 18°10′S 47°17′E].

Note: *Alsomyia* Brauer & Bergenstamm, 1891 was recognized from the Afrotropical Region by Mesnil (1977b) based on his new species *Alsomyia chloronitens* Mesnil, 1977. This species is moved here to *Nealsomyia* Mesnil, 1939.

***clausa*** (Curran, 1940).—Afrotropical: Zimbabwe. **Comb. n.**

*Phorocera clausa* Curran, 1940: 9. Holotype male (AMNH). Type locality: Zimbabwe, Kadoma [as “Gatooma”].

Note: *Phorocera clausa* Curran, 1940 was treated as an unplaced species of “Goniinae” [= Exoristinae] by Crosskey (1980b: 881) but is moved here to *Nealsomyia* Mesnil, 1939 based on examination of the holotype.

***lindneri*** Mesnil, 1959.—Afrotropical: Tanzania.

*Nealsomyia lindneri* Mesnil, 1959: 12. Holotype male (SMNS). Type locality: Tanzania, Lake Victoria, Mugango.

***merzi*** Cerretti, 2005.—Afrotropical: Namibia.

*Nealsomyia merzi* Cerretti, 2005: 129. Holotype male (MHNG). Type locality: Namibia, Mount Erongo.

Undescribed sp.: South Africa (NMB, examined by PC).

##### Genus *PALES* Robineau-Desvoidy, 1830

***PALES*** Robineau-Desvoidy, 1830: 154 (not a junior homonym of *Pales* Meigen, 1800 [Tipulidae] because the work in which that name appeared was suppressed by ICZN 1963: 339). Type species: *Pales florea* Robineau-Desvoidy, 1830 (= *Tachina pavida* Meigen, 1824), by subsequent designation of Coquillett (1910: 582) [Palaearctic].

*CTENOPHOROCERA* Brauer & Bergenstamm, 1891: 342 [also 1891: 38]. Type species: *Ctenophorocera blepharipus* Brauer & Bergenstamm, 1891, by subsequent designation of Sharp (1893: 299).

*NEOPALES* Coquillett, 1910: 575 (*nomen novum* for *Pales* Robineau-Desvoidy, 1830; proposed prior to the suppression of *Pales* Meigen, 1800 by ICZN 1963: 339).

*MICROPALES* Villeneuve, 1927: 121. Type species: *Micropales seminitida* Villeneuve, 1927, by monotypy.

***aethiopica*** (Mesnil, 1950).—Afrotropical: D.R. Congo, South Africa, Sudan, Tanzania.

Ctenophorocera (Ctenophorocera) aethiopica Mesnil, 1950c: 124. Holotype male (CNC). Type locality: northwestern Tanzania, forest edge, 1800–2000m.

***blepharipa*** (Brauer & Bergenstamm, 1891).—Afrotropical: D.R. Congo, South Africa, Uganda.

*Ctenophorocera blepharipus* Brauer & Bergenstamm, 1891: 342 [also 1891: 38]. Type(s), male (NHMW, not located by JEOH). Type locality: South Africa, Western Cape, Cape of Good Hope [as “?(Cap oder Brasilien. Coll. Winth.)”].

Note: Aldrich (1927d: 24) examined a male in NHMW bearing “a large blue name label in Doctor Villeneuve’s writing” that he treated as the “type” of *Ctenophorocera blepharipus* Brauer & Bergenstamm, 1891. The specimen was also labelled “caffra. Coll. Winthem”, with “caffra” meaning “Caffraria”, a former region in Eastern Cape (also known as “Kaffraria”). This is a different locality from the two possible localities given in the original description (Cape of Good Hope or Brazil). Given the uncertainty about whether this specimen is a name-bearing type of *Ctenophorocera blepharipus*, Aldrich’s (1927d: 24) examination of the “type” is not accepted as a lectotype fixation. JEOH did not find a specimen in NHMW that matches the expected type data for *Ctenophorocera blepharipus*.

***coerulea*** (Jaennicke, 1867).—Afrotropical: “n.-e. Afr. to sthn Afr.” (Crosskey 1980b: 870), including Ethiopia, South Africa, Zimbabwe.

*Phorocera coerulea* Jaennicke, 1867: 382 [also 1868: 74]. Type(s), male (SMF). Type locality: Ethiopia, “Simen” (probably the Simien Mountains area).

*caerulea*. Incorrect subsequent spelling of *coerulea* Jaennicke, 1867 (Aldrich 1927: 23).

Note: Crosskey (1980b: 870) noted the possible presence of *Phorocera coerulea* Jaennicke, 1867 in the Oriental Region, probably based on Mesnil’s (1950c: 126) mention of “?Indien”. The presence of this species in the Oriental Region needs confirmation.

***coeruleonigra*** (Mesnil, 1950).—Afrotropical: Zimbabwe.

Ctenophorocera (Ctenophorocera) coerulea coeruleonigra Mesnil, 1950c: 126 (as “*coeruleo-nigra*”). Holotype male (CNC). Type locality: Zimbabwe, Mutare [as “Umtali”] District, Vumba Mountains.

***contristans*** Villeneuve, 1938.—Afrotropical: South Africa.

*Pales contristans* Villeneuve, 1938c: 1. Type(s), unspecified sex (1 male in CNC). Type locality: South Africa, “Colonie du Cap” ([former Cape Province], Algoa Bay, according to label data of CNC syntype, Cooper and O’Hara 1996: 57).

***corrupta*** (Curran, 1927).—Afrotropical: Uganda.

*Zenillia corrupta* Curran, 1927d: 331. Holotype male (BMNH). Type locality: Uganda, Jeza [ca. 0°22′N 32°17′E].

Note: Curran (1927d: 331) reported that the holotype of his new species *Zenillia corrupta* was reared from a syrphid (Diptera, Syrphidae). This is a dubious record, but if true would be the only known case of parasitism of a syrphid (larva?) by a tachinid.

***cuthbertsoni*** (Curran, 1940).—Afrotropical: Zimbabwe.

*Phorocera cuthbertsoni* Curran, 1940: 5. Holotype male (AMNH). Type locality: Zimbabwe, Nyanga [as “Inyanga”].

*cuthbersoni*. Incorrect subsequent spelling of *cuthbertsoni* Curran, 1940 (original usage not found but spelling listed by Crosskey 1980b: 870).

***divergens*** (Curran, 1928).—Afrotropical: Uganda.

*Phorocera divergens* Curran, 1928a: 237. Holotype male (BMNH). Type locality: Uganda, Kampala.

***experta*** (Brauer & Bergenstamm, 1891).—Afrotropical: South Africa.

*Ctenophorocera experta* Brauer & Bergenstamm, 1891: 342 [also 1891: 38] (as “*experta* Wd.”). Lectotype male (NHMW, not located by JEOH), by fixation of Townsend (1941: 98) (mention of “Ht male” from Cape of Good Hope in NHMW is regarded as a lectotype fixation). Type locality: South Africa, Western Cape, Cape of Good Hope [as “Cap b. sp.” = “Cap Bonae Spei”].

***gnu*** (Curran, 1940).—Afrotropical: Liberia, Nigeria, Rwanda.

*Phorocera gnu* Curran, 1940: 11. Holotype male (AMNH). Type locality: Liberia, Ganta.

***macrocephala*** (Mesnil, 1950).—Afrotropical: Kenya (**new record**, MZUR [PC]), Malawi, South Africa.

Ctenophorocera (Ctenophorocera) macrocephala Mesnil, 1950c: 123. Holotype male (CNC). Type locality: Malawi, Nyika Plateau.

***maculisquama*** (Mesnil, 1950).—Afrotropical: Zimbabwe.

Ctenophorocera (Ctenophorocera) coerulea maculisquama Mesnil, 1950c: 126. Holotype male (CNC). Type locality: Zimbabwe, Harare [as “Salisbury”].

Note: Crosskey (1980b: 870) noted the possible presence of *Ctenophorocera coerulea maculisquama* Mesnil, 1950 in the Oriental Region, probably based on Mesnil’s (1950c: 126) statement: “?. Ein defektes ♂ aus Indien”. The presence of this species in the Oriental Region needs confirmation.

***metro*** (Curran, 1940).—Afrotropical: Zambia, Zimbabwe.

*Phorocera metro* Curran, 1940: 12. Holotype male (AMNH). Type locality: border between Zambia and Zimbabwe, Victoria Falls.

***nigronitens*** Villeneuve, 1938.—Afrotropical: D.R. Congo, South Africa.

*Pales nigronitens* Villeneuve, 1938c: 1 (as “*nigro-nitens*”). Syntypes, males and females (?IRSNB). Type locality: D.R. Congo.

*Phorocera ethelia* Curran, 1940: 9. Holotype male (AMNH). Type locality: South Africa, KwaZulu-Natal, Durban.

***nyasa*** (Curran, 1940).—Afrotropical: Malawi, South Africa.

*Phorocera nyasa* Curran, 1940: 13. Holotype male (BMNH). Type locality: Malawi, Nsanje [as “Port Herald”].

***pauciseta*** (Mesnil, 1950).—Afrotropical: D.R. Congo.

Ctenophorocera (Ctenophorocera) pauciseta Mesnil, 1950c: 125. Holotype male (CNC). Type locality: D.R. Congo, Équateur, Eala (see O’Hara and Cooper 1996: 27 for label data).

Note: O’Hara (1996: 152) commented on the holotype of *Ctenophorocera pauciseta* Mesnil, 1950: “The type locality is stated as Kisantu but the specimen labelled as the holotype is from Eala. The length of the Eala specimen corresponds with the length given in the description, whereas the two CNC specimens from Kisantu are larger.”

***rubrica*** Villeneuve, 1932.—Afrotropical: Kenya, Tanzania.

*Pales rubrica* Villeneuve, 1932: 285. Holotype male (BMNH). Type locality: Kenya, Aberdare Mountains, 7300ft.

***rubriventris*** Bezzi, 1908.—Afrotropical: South Africa.

*Pales rubriventris* Bezzi, 1908a: 185. Holotype female (not located). Type locality: South Africa, Northern Cape, Steinkopf.

***ruficauda*** (Curran, 1927).—Afrotropical: D.R. Congo.

*Phorocera ruficauda* Curran, 1927c: 9. Holotype female (AMNH). Type locality: D.R. Congo, Orientale, Kisangani [as “Stanleyville”].

***rufolateralis*** (Curran, 1940).—Afrotropical: Kenya, Malawi, South Africa.

*Phorocera rufolateralis* Curran, 1940: 11. Holotype male (BMNH). Type locality: Malawi, Zomba.

***sarcophagaeformis*** (Jaennicke, 1867).—Afrotropical: Ethiopia, Kenya, Malawi, South Africa, Tanzania, Uganda.

*Phorocera sarcophagaeformis* Jaennicke, 1867: 381 [also 1868: 73]. Type(s), male (SMF). Type locality: Ethiopia, “Simen” (probably the Simien Mountains area).

***seminitida*** (Villeneuve, 1927).—Afrotropical: D.R. Congo, Malawi, Nigeria, Zimbabwe.

*Micropales seminitida* Villeneuve, 1927: 121. Lectotype male (BMNH), by fixation of Townsend (1941: 108) (mention of “Ht male” from Ibadan in BMNH is regarded as a lectotype fixation). Type locality: Nigeria, Ibadan.

***senex*** (Curran, 1927).—Afrotropical: D.R. Congo, Nigeria.

*Phorocera senex* Curran, 1927c: 10. Holotype male (AMNH). Type locality: D.R. Congo, Orientale, Kisangani [as “Stanleyville”].

***setigena*** (Curran, 1940).—Afrotropical: South Africa, Zimbabwe.

*Phorocera setigena* Curran, 1940: 11. Holotype male (BMNH). Type locality: South Africa, KwaZulu-Natal, Stella Bush [near Durban].

Note: Curran (1940: 11) cited the type locality of *Phorocera setigena* as “Marley, Stella Bush”, but Marley was the collector.

***somomyina*** (Karsch, 1886).—Afrotropical: Angola.

*Phorocera somomyina* Karsch, 1886b: 340. Holotype, unspecified sex (ZMHB, not located by JEOH). Type locality: Angola, Pungo Andongo.

***splendens*** Mesnil, 1970.—Afrotropical: Madagascar.

*Pales splendens* Mesnil, 1970b: 89. Holotype male (MNHN). Type locality: Madagascar, Toamasina, Moramanga.

***tessellans*** (Mesnil, 1950).—Afrotropical: South Africa.

Ctenophorocera (Ctenophorocera) tessellans Mesnil, 1950c: 123. Holotype male (CNC). Type locality: South Africa, KwaZulu-Natal.

***tetra*** (Curran, 1940).—Afrotropical: South Africa.

*Phorocera tetra* Curran, 1940: 12. Holotype female (SANC). Type locality: South Africa, Mpumalanga, Barberton.

##### Genus *PERLUCIDINA* Mesnil, 1952

*PERLUCIDINA* Mesnil, 1949b: 104 (as subgenus of *Tamaromyia* Mesnil, 1949). *Nomen nudum* (proposed after 1930 without designation of type species; no included species) (see Evenhuis and O’Hara 2008: 67).

***PERLUCIDINA*** Mesnil, 1952c: 223 (as subgenus of *Hygia* Mesnil, 1952 [not *Hygia* Uhler, 1861]). Type species: *Exorista perlucida* Karsch, 1886, by monotypy (see Evenhuis and O’Hara 2008: 67). **Status revived.**

Note: Crosskey (1980b: 869) synonymized *Perlucidina* Mesnil, 1952 with *Calozenillia* Townsend, 1927. We do not agree with this synonymy and here reinstate *Perlucidina* as a genus. The characters that distinguish *Perlucidina* will be given in the Tachinidae chapter of the *Manual of Afrotropical Diptera* (in prep.).

***africana*** (Jaennicke, 1867).—Afrotropical: Ethiopia. **Comb. n.**

*Exorista africana* Jaennicke, 1867: 384 [also 1868: 76]. Type(s), female (SMF). Type locality: Ethiopia, “Simen” (probably the Simien Mountains area).

Note: *Exorista africana* Jaennicke, 1867 was treated as a species of *Calozenillia* Townsend, 1927 by Crosskey (1980b: 869, 1984: 281) but is moved here to the newly revived genus *Perlucidina* Mesnil, 1952.

***perlucida*** (Karsch, 1886).—Afrotropical: Angola, D.R. Congo, Malawi, South Africa, Sudan, Uganda, Zambia. **Comb. revived.**

*Tachina dubia* Walker, 1853: 291 (junior primary homonym of *Tachina dubia* Fallén, 1810). Type(s), female (BMNH). Type locality: South Africa, Western Cape, Cape of Good Hope [as “Cape”].

*Exorista perlucida* Karsch, 1886b: 339. Holotype, unspecified sex [male, examined by JEOH] (ZMHB). Type locality: Angola, Pungo Andongo.

Note: *Exorista perlucida* Karsch, 1886b was treated as a species of *Calozenillia* Townsend, 1927 by Crosskey (1980b: 869, 1984: 281) but is moved here to the newly revived genus *Perlucidina* Mesnil, 1952.

##### Genus *PEXOPSIS* Brauer & Bergenstamm, 1889

***PEXOPSIS*** Brauer & Bergenstamm, 1889: 88 [also 1890: 20]. Type species: *Eurigaster tibialis* Robineau-Desvoidy, 1849 (as “*tibialis* Mg.”) (= *Tachina aprica* Meigen, 1824), by monotypy [Palaearctic].

***chapini*** (Curran, 1927).—Afrotropical: D.R. Congo, Kenya, Uganda.

*Sturmia chapini* Curran, 1927a: 11. Holotype male (AMNH). Type locality: D.R. Congo, Orientale, Kisangani [as “Stanleyville”].

***femoralis*** Bezzi, 1911.—Afrotropical: Malawi, Mozambique.

*Pexopsis femoralis* Bezzi, 1911: 59. Holotype female (USNM). Type locality: Mozambique, Maputo Province, “Umbelusi”.

***garambana*** Verbeke, 1962.—Afrotropical: D.R. Congo.

*Pexopsis garambana* Verbeke, 1962b: 51. Holotype female (IRSNB). Type locality: D.R. Congo, Orientale, Parc National de la Garamba [as “P.N.G.”].

***lindneri*** Mesnil, 1959.—Afrotropical: D.R. Congo, Tanzania.

*Pexopsis lindneri* Mesnil, 1959: 10. Holotype male (SMNS). Type locality: Tanzania, Pare Mountains, 1800m.

***pyrrhaspis*** Villeneuve, 1916.—Afrotropical: “widespread W. Afr., E. Afr. & sthn Afr.” (Crosskey 1980b: 873), including Kenya, Malawi, South Africa.

*Pexopsis pyrrhaspis* Villeneuve, 1916c: 492. Syntypes, 2 females (BMNH, SAMC). Type localities: Malawi and South Africa, “Cape Colony” (former Cape Province, corresponding to the present-day Western Cape, Eastern Cape, Northern Cape, and North West [in part] provinces).

***yemenensis*** Zeegers, 2007.—Afrotropical: Yemen.

*Pexopsis yemenensis* Zeegers, 2007: 393. Holotype male (RMNH). Type locality: Yemen, Wādī Lahīmah [as “Al Lahima”] (15°24′N 43°32′E).

##### Genus *PHYTOMYPTERINA* van Emden, 1960

***PHYTOMYPTERINA*** van Emden, 1960: 356. Type species: *Phytomypterina burtti* van Emden, 1960 (= *Phytomyptera rufescens* Villeneuve, 1936), by original designation.

***rufescens*** (Villeneuve, 1936).—Afrotropical: Mozambique (**new record**, MZUR [PC]), South Africa, Tanzania.

*Phytomyptera rufescens* Villeneuve, 1936a: 3. Holotype female (CNC). Type locality: South Africa (KwaZulu-Natal, Mfongosi [as “M fongosi Zulu L.”] according to label data, Cooper and O’Hara 1996: 63).

*Phytomypterina burtti* van Emden, 1960: 357. Holotype male (BMNH). Type locality: Tanzania, Singida.

##### Genus *PIMELIMYIA* Mesnil, 1949

***PIMELIMYIA*** Mesnil, 1949b: 104. Type species: *Sturmia russata* Villeneuve, 1934 (as “*Pimelimyia russata* Vill.”, p. 103), by monotypy (see Evenhuis and O’Hara 2008: 67).

***grossa*** Mesnil, 1959.—Afrotropical: Tanzania, Zimbabwe.

*Pimelimyia grossa* Mesnil, 1959: 10. Holotype female (SMNS). Type locality: Tanzania, Pare Mountains, Usangi.

***insularis*** (Villeneuve, 1915).—Afrotropical: Madagascar.

*Sturmia insularis* Villeneuve, 1915b: 193. Syntypes, 1 male and 1 female (male in NHMW, female not located). Type localities: Madagascar, female syntype from Antananarivo, Antananarivo [as “Tananarive”], male syntype (examined by JEOH) from “Mgdk.” [= Madagascar] without further locality data.

Note: *Sturmia insularis* Villeneuve, 1915 is probably misplaced in *Pimelimyia* Mesnil, 1949; it lacks a sexual patch on the underside of abdominal tergite 4 but is otherwise similar to species of *Blepharipa* Rondani, 1856 (a genus not recorded from the Afrotropical Region).

***natalensis*** (Curran, 1927).—Afrotropical: South Africa.

*Sturmia natalensis* Curran, 1927f: 121. Holotype female (BMNH). Type locality: South Africa, KwaZulu-Natal, Weenen [ca. 28°51′S 30°4′E].

***rufina*** (Curran, 1927).—Afrotropical: South Africa.

*Sturmia rufina* Curran, 1927f: 125. Holotype female (SANC). Type locality: South Africa, Gauteng, Pretoria.

***rufula*** (Villeneuve, 1943).—Afrotropical: “Afrique orientale” [East Africa], South Africa.

*Sturmia rufula* Villeneuve, 1943a: 38. Syntypes, 2 females (not located). Type locality: “Afrique orientale” [East Africa].

***russata*** (Villeneuve, 1943).—Afrotropical: “l’Afrique orientale” [East Africa], South Africa.

*Sturmia russata* Villeneuve, 1943a: 37. Syntypes, 2 males (not located). Type localities: South Africa and “l’Afrique orientale” [East Africa].

***semitestacea*** (Villeneuve, 1916).—Afrotropical: Malawi, South Africa, Tanzania, Zimbabwe.

Sturmia (Blepharipoda) semitestacea Villeneuve, 1916c: 477. Syntypes, 7 males and females (BMNH, SAMC [1 male examined by JEOH]). Type localities: Malawi and South Africa [latter inferred from deposition of specimens in “S. Afric. Museum”].

##### Genus *PROSOPODOPSIS* Townsend, 1926

***PROSOPODOPSIS*** Townsend, 1926b: 542. Type species: *Tachina fasciata* Wiedemann, 1830 (junior primary homonym of *Tachina fasciata* Fallén, 1820; = *Prosopaea appendiculata* de Meijere, 1910), by original designation [Oriental].

Note: Three undescribed species from Namibia, Nigeria and Uganda assigned to *Prosopodopsis* Townsend, 1926 by Crosskey (1980b: 880, 1984: 295) were placed elsewhere by Cerretti (2009b: 52).

***pulchricornis*** (Villeneuve, 1938).—Afrotropical: Mozambique, ?South Africa.

*Histochaeta pulchricornis* Villeneuve, 1938a: 3. Holotype male (CNC). Type locality: Africa (“Erosba Pan” according to label data [an unknown location], collected by the “S.W. Africa Mus. Exped.”); considered “sud-africaine très probablement” by Villeneuve (1938a: 3).

Note: *Histochaeta pulchricornis* Villeneuve, 1938 was treated as a species of *Chlorolydella* Townsend, 1933 by Crosskey (1980b: 877) but was moved to *Prosopodopsis* Townsend, 1926 by Crosskey (1984: 201, 295). This placement was upheld by Cerretti (2009b: 52).

Undescribed sp.: Nigeria (CNC, examined by PC).

Undetermined spp.: U.A. Emirates (Zeegers 2010), Yemen (Zeegers 2007: 394).

##### Genus *PSEUDALSOMYIA* Mesnil, 1968

***PSEUDALSOMYIA*** Mesnil, 1968c: 178. Type species: *Pseudalsomyia piligena* Mesnil, 1968, by original designation [Oriental].

***audisioi*** Cerretti, 2012.—Afrotropical: Kenya.

*Pseudalsomyia audisioi* Cerretti, 2012: 329. Holotype male (MZUR). Type locality: Kenya, Western, Kakamega Forest, 1600m (0°13′37.2″N 34°52′49.8″E).

##### Genus *PSEUDOGONIA* Brauer & Bergenstamm, 1889

***PSEUDOGONIA*** Brauer & Bergenstamm, 1889: 100 [also 1890: 32]. Type species: *Gonia cinerascens* Rondani, 1859 (= *Tachina rufifrons* Wiedemann, 1830), by monotypy [Palaearctic].

*GAEDIOGONIA* Townsend, 1927c: 71. Type species: *Gaediogonia jacobsoni* Townsend, 1927 (= *Tachina rufifrons* Wiedemann, 1830), by original designation [Oriental].

***fasciata*** (Wiedemann, 1819).—Afrotropical: South Africa, Zimbabwe. Palaearctic: Europe (SW. Eur.), N. Africa (Canary Is.).

*Gonia fasciata* Wiedemann, 1819: 25. Syntypes, female (2 syntypes in ZMUC, Zimsen 1954: 23). Type locality: South Africa, Western Cape, Cape of Good Hope [as “Prom. bon. sp.” = “Promontorium Bonae Spei”].

*Rhedia capensis* Robineau-Desvoidy, 1830: 77. Type(s), unspecified sex (MNHN or lost). Type locality: South Africa, Western Cape, Cape of Good Hope [as “cap de Bonne-Espérance”].

*Reaumuria lalandii* Robineau-Desvoidy, 1830: 80. Type(s), unspecified sex (MNHN or lost). Type locality: South Africa, Western Cape, Cape of Good Hope [as “cap de Bonne-Espérance”].

Note: *Gonia fasciata* Wiedemann, 1819 was described from an unspecified number of females, but certainly more than one because Wiedemann (1930: 344) later wrote, “In Westermann’s und meiner Sammlung”. Hence, the original type series is interpreted as consisting of syntypes.

***madagascariensis*** Villeneuve, 1915.—Afrotropical: Madagascar.

*Pseudogonia madagascariensis* Villeneuve, 1915b: 192. Lectotype female (CNC), by fixation of Cooper and O’Hara (1996: 66) (data on “Holotype ♀” from Tananarive in CNC is regarded as a lectotype fixation). Type locality: Madagascar, Antananarivo, Antananarivo [as “Tananarive”].

Note: There are eight paralectotypes, most or all of which females, of *Pseudogonia madagascariensis* Villeneuve, 1915 in NHMW (examined by JEOH).

***rufifrons*** (Wiedemann, 1830).—Afrotropical: “widespread Afrotrop. Reg.” (Crosskey 1980b: 875), including Cape Verde, Nigeria, South Africa, Tanzania, U.A. Emirates, Yemen. Palaearctic: C. Asia, Europe (all except British Is., Scand.), Japan, Kazakhstan, Korea (S. Korea), M. East (Israel), Mongolia, Pal. China, Russia (W. Russia, W. Siberia, S. Far East), Transcaucasia. Oriental: India, Indonesia, Malaysia, Myanmar, Orien. China, Pakistan, Philippines, Ryukyu Is., Taiwan, Thailand. Australasian: Australia, Hawaii, Melanesia, N. Australasian.

*Latreillia lalandii* Robineau-Desvoidy, 1830: l06 (junior secondary homonym of *Reaumuria lalandii* Robineau-Desvoidy, 1830). Type(s), unspecified sex (MNHN or lost). Type locality: South Africa, Western Cape, Cape of Good Hope [as “cap de Bonne-Espérance”].

*Tachina rufifrons* Wiedemann, 1830: 318. Lectotype female (ZMUC), by fixation of Crosskey (1966a: 677) (examination of “Holotype ♀” from China in ZMUC is regarded as a lectotype fixation). Type locality: China.

*Gonia cinerascens* Rondani, 1859: 34. Syntypes, unspecified number and including at least 1 male (MZF, Herting 1969: 192; 1 male and 7 females in MZF [examined by PC]). Type locality: Italy, hills near Parma.

*Gonia munroi* Curran, 1927d: 339. Holotype male (BMNH). Type locality: Tanzania.

*Gonia ritchiei* Cuthbertson & Munro, 1941: 109. *Nomen nudum*.

Note: The relative priority of *Reaumuria lalandii* Robineau-Desvoidy, 1830 and *Latreillia lalandii* Robineau-Desvoidy, 1830, when both are placed in the same genus, was established by Crosskey (1980b: 875), as the First Reviser (Article 24.2.2 of the *Code*, ICZN 1999). Since the latter name was given junior homonym status, it cannot replace *Tachina rufifrons* Wiedemann, 1830 as the valid name of the species even though it was published first; see dating of Robineau-Desvoidy (1830) and Wiedemann (1830) in References.

*Gonia cinerascens* Rondani, 1859 was probably described from both sexes but the original description only made specific mention of the male. Crosskey (1976: 244) reported three male and four female syntypes in MZF. An examination of the MZF holdings by PC discovered another syntype and a change to the reported sexes. The type series was found to consist of one male and seven females. The single male is *Gonia picea* (Robineau-Desvoidy, 1830) and the seven females conform to the present concept of *Gonia cinerascens* Rondani. When treated as a valid name, *Gonia cinerascens* has also been called *Isomera cinerascens* (Rondani) in the literature.

*Gonia munroi* Curran (1927d: 339) was “Described from 5♂♂, 8♀♀, from Tanganyika and South Africa. The type male and female are from Tanganyika ...”. We recognize the male from Tanzania as a designated holotype whereas Arnaud and Owen (1981: 209) treated all specimens as syntypes, writing “syntypes, 5 males and 8 females”.

***suspecta*** Villeneuve, 1915.—Afrotropical: Madagascar.

*Pseudogonia suspecta* Villeneuve, 1915b: 192 (as “*Pseudogonia suspecta* (n. sp.? vel n. var.?)”). Syntypes, 3 specimens of uncertain sex [“Par l’absence de soies orbitaires et surtout par la longueur des antennes, ils semblent bien être des ♂; néanmoins les griffes sont courtes comme chez les ♀.”] (NHMW, not located by JEOH). Type locality: Madagascar.

##### Genus *RAMONELLA* Kugler, 1980

*RAMONA* Kugler, 1980a: 40 (junior homonym of *Ramona* Casey, 1886). Type species: *Ramona mesnili* Kugler, 1980, by original designation.

***RAMONELLA*** Kugler, 1980b: 67 (*nomen novum* for *Ramona* Kugler, 1980).

***mesnili*** (Kugler, 1980).—Afrotropical: Yemen. Palaearctic: Europe (Turkey), M. East (Israel), N. Africa (Canary Is.).

*Ramona mesnili* Kugler, 1980a: 41. Holotype male (TAU). Type locality: Israel, Negev, Ramon.

##### Genus *RHYNCHOGONIA* Brauer & Bergenstamm, 1893

***RHYNCHOGONIA*** Brauer & Bergenstamm, 1893: 37, 104 [also 1893: 125, 192]. Type species: *Rhynchogonia algerica* Brauer & Bergenstamm, 1893, by monotypy.

***algerica*** Brauer & Bergenstamm, 1893.—Afrotropical: U.A. Emirates. Palaearctic: C. Asia, M. East (Israel), N. Africa (NW. Africa).

*Rhynchogonia algerica* Brauer & Bergenstamm, 1893: 105 [also 1893: 193]. Type(s), female (1 female in NHMW according to Herting 1974b: 135, not located by JEOH). Type locality: “Afrika” (Algeria, Biskra according to Herting 1974b: 135).

##### Genus *SCHEMBRIA* Rondani, 1861

***SCHEMBRIA*** Rondani, 1861b: 110. Type species: *Schembria meridionalis* Rondani, 1861, by monotypy [Palaearctic].

Note: *Schembria* Rondani, 1861 was first recognized from the Afrotropical Region by Crosskey (1984: 201, 287) based on an undescribed species from South Africa. That species was subsequently described by Barraclough (1991: 135) as *Schembria eldana*.

***eldana*** Barraclough, 1991.—Afrotropical: South Africa.

*Schembria eldana* Barraclough, 1991: 135. Holotype male (NMDA). Type locality: South Africa, KwaZulu-Natal, Lower Tugela River, Tongaat (29°35′S 31°08′E), Wewe Sugar Estate.

##### Genus *SIMOMA* Aldrich, 1926

***SIMOMA*** Aldrich, 1926b: 20. Type species: *Simoma grahami* Aldrich, 1926, by original designation. **New record.**

***grahami*** Aldrich, 1926.—Afrotropical: Namibia (**new record**, MZUR [PC]). Palaearctic: Japan, M. East (Israel), Pal. China. Oriental: India, Malaysia, Orien. China, Vietnam.

*Simoma grahami* Aldrich, 1926b: 21. Holotype male (USNM). Type locality: China, Sichuan, Suifu.

Note: *Simoma grahami* Aldrich, 1926 is newly recorded from the Afrotropical Region. O’Hara et al. (2009: 117), in a note about *Simoma grahami*, wrote: “This species may have been recorded from Japan in error (e.g., Crosskey 1976: 253, Herting 1984: 73)”. *Simoma grahami* has since been recorded from Honshu (Japan) by Shima (2014: 861) and PC examined a male in IRSNB from Tokyo (collected by Edme Gallois on 6 June 1909).

##### Genus *STIREMANIA* Cerretti & O’Hara, gen. n.

***STIREMANIA*** Cerretti & O’Hara, **gen. n.** Type species: *Stiremania karoo* Cerretti and O’Hara sp. n., by present designation.

Note: This new genus and the two new species below are described in the New Taxa of Afrotropical Tachinidae section.

***karoo*** Cerretti & O’Hara, **sp. n.**—Afrotropical: South Africa.

*Stiremania karoo* Cerretti & O’Hara, **sp. n.** Holotype male (MZUR). Type locality: South Africa, Western Cape, Gamkaskloof (Die Hel), 336m (33°22′5.90″S 21°37′19.43″E).

***robusta*** Cerretti & O’Hara, **sp. n.**—Afrotropical: South Africa.

*Stiremania robusta* Cerretti & O’Hara, **sp. n.** Holotype male (NMDA). Type locality: South Africa, Eastern Cape, Willowmore.

##### Genus *STURMIA* Robineau-Desvoidy, 1830

***STURMIA*** Robineau-Desvoidy, 1830: 171. Type species: *Sturmia vanessae* Robineau-Desvoidy, 1830 (= *Tachina bella* Meigen, 1824), by subsequent designation of Robineau-Desvoidy (1863a: 888) (earlier type fixations set aside by ICZN 2012: 242; see Evenhuis and Thompson 1990: 238 and O’Hara and Evenhuis 2011: 61) [Palaearctic].

*POLYCHNOMYIA* Bischof, 1904: 85. Type species: *Polychnomyia flavohalterata* Bischof, 1904 (= *Tachina convergens* Wiedemann, 1824), by monotypy.

*VERBEKEIA* Mesnil, 1959: 5. Type species: *Verbekeia lindneri* Mesnil, 1959, by monotypy.

***bellina*** Mesnil, 1944.—Afrotropical: Madagascar.

*Sturmia bellina* Mesnil, 1944: 10. Holotype male (MNHN). Type locality: Madagascar, Toliara, Bekily.

***convergens*** (Wiedemann, 1824).—Afrotropical: Ethiopia, Kenya, Malawi, Nigeria, Sierra Leone, South Africa, Tanzania, Uganda, Zambia, Zimbabwe. Oriental: India, Sri Lanka. Australasian: Australia, N. Australasian.

*Tachina convergens* Wiedemann, 1824: 43. Lectotype female (ZMUC), by designation of Crosskey (1963: 78). Type locality: “India orient.” (i.e., “East Indies”; interpreted as India by Crosskey 1963: 78 and Crosskey 1976: 242).

*Polychnomyia flavohalterata* Bischof, 1904: 86. Type(s), male (1 male in NHMW). Type locality: South Africa, Eastern Cape, Algoa Bay.

*Sturmia completa* Curran, 1927f: 119. Holotype male (SANC). Type locality: South Africa, Mpumalanga, White River [ca. 25°20′S 31°1′E].

*Tachina bella* of authors, not Meigen, 1824. Misidentification (Crosskey 1980b: 874).

Note: *Polychnomyia flavohalterata* Bischof, 1904 was described from one or more males. There are two specimens in NHMW, one male and one female, with label data corresponding to that published for the type(s) of *Polychnomyia flavohalterata* and both labelled as “Typ.” by Villeneuve (there is no Bischof det. label on either of these specimens). Since the original description mentioned only the male sex, only the male is a name-bearing type. This male also bears a Crosskey holotype label dated 1970, but Bischof did not give the number of specimens upon which his description was based and therefore this “holotype” is regarded as a syntype.

***lindneri*** (Mesnil, 1959).—Afrotropical: D.R. Congo, Nigeria, Tanzania, Uganda.

*Verbekeia lindneri* Mesnil, 1959: 5. Holotype male (SMNS). Type locality: Tanzania, Kware [ca. 3°17′S 37°9′E].

***profana*** (Karsch, 1888).—Afrotropical: “Ost-Afrika” [East Africa]. **Comb. n.**

*Degeeria profana* Karsch, 1888: 376. Holotype male [not female as published] (ZMHB). Type locality: “Ost-Afrika” [East Africa].

Note: *Degeeria profana* Karsch, 1888 was treated as an unplaced species of “Goniinae” [= Exoristinae] by Crosskey (1980b: 881) but is moved here to *Sturmia* Robineau-Desvoidy, 1830 based on examination of the holotype.

***rasa*** (Mesnil, 1959).—Afrotropical: Tanzania.

*Pimelimyia rasa* Mesnil, 1959: 8. Holotype male (SMNS). Type locality: Tanzania, “Ngaruka” [probably Engaruka, ca. 3°0′S 35°58′E].

***rasella*** (Mesnil, 1970).—Afrotropical: Madagascar.

*Pimelimyia rasella* Mesnil, 1970b: 100. Holotype male (MNHN). Type locality: Madagascar, Toliara, Sakaraha.

***velutina*** Mesnil, 1944.—Afrotropical: Madagascar.

*Sturmia velutina* Mesnil, 1944: 11. Holotype male (MNHN). Type locality: Madagascar.

##### Unplaced species of Goniini

***clarior*** Villeneuve, 1943.—Afrotropical: Zimbabwe.

*Sturmia russata clarior* Villeneuve, 1943a: 38. Holotype male (not located). Type locality: southern Zimbabwe.

***inimica*** Hesse, 1934.—Afrotropical: South Africa.

*Sturmia inimica* Hesse, 1934: 428. Holotype female (SAMC). Type locality: South Africa, Western Cape, Somerset West.

***rufiventris*** Curran, 1927.—Afrotropical: D.R. Congo. **Comb. n.**

*Ceromasia rufiventris* Curran, 1927c: 7. Holotype female (AMNH). Type locality: D.R. Congo, Orientale, Kisangani [as “Stanleyville”].

Note: *Ceromasia rufiventris* Curran, 1927 was placed in *Eurysthaea* Robineau-Desvoidy, 1863 by Crosskey (1980b: 878) as a new combination. The female holotype was examined by PC and could not be placed to genus within the Goniini but does not belong to *Eurysthaea*.

***vocalis*** Villeneuve, 1943.—Afrotropical: D.R. Congo.

*Sturmia vocalis* Villeneuve, 1943a: 36. Syntypes, 1 male and 1 female (not located). Type localities: D.R. Congo, Orientale, Kisangani [as “Stanleyville”] and Nord-Kivu, “Lessewoud” [assumed to be Lesse at ca. 0°45′N 29°46′E].

#### Tribe THRIXIONINI

##### Genus *THRIXION* Brauer & Bergenstamm, 1889

***THRIXION*** Brauer & Bergenstamm, 1889: 108 [also 1890: 40]. Type species: *Phytomyptera aberrans* Schiner, 1861, by monotypy [Palaearctic].

Undetermined sp.: Yemen, as “Thrixion cf. pilifrons Mesnil, 1963” (Zeegers 2007: 400).

#### Tribe WINTHEMIINI

##### Genus *HEMIWINTHEMIA* Villeneuve, 1938

***HEMIWINTHEMIA*** Villeneuve, 1938c: 4. Type species: *Hemiwinthemia calva* Villeneuve, 1938, by monotypy.

Note: Crosskey (1984: 274) considered *Hemiwinthemia* Villeneuve, 1938 “of dubious status and probably should not be maintained distinct from *Winthemia* [Robineau-Desvoidy, 1830]”.

***calva*** Villeneuve, 1938.—Afrotropical: D.R. Congo.

*Hemiwinthemia calva* Villeneuve, 1938c: 5. Holotype female (not located). Type locality: D.R. Congo, Katanga, Bukama.

##### Genus *NEMORILLA* Rondani, 1856

***NEMORILLA*** Rondani, 1856: 66. Type species: *Tachina maculosa* Meigen, 1824, by original designation [Palaearctic].

***afra*** Curran, 1939.—Afrotropical: Ghana, Mozambique, Nigeria, South Africa.

*Nemorilla afra* Curran, 1939: 3. Holotype male (SANC). Type locality: Mozambique, Maputo [as “Lourenco Marquis”].

*floralis* (Fallén, 1810).—Afrotropical: ?Eritrea. [Palaearctic.]

*Tachina floralis* Fallén, 1810: 287.

Note: Bezzi’s (1908b: 54) record of *Nemorilla floralis* (Fallén, 1810) (as *Nemorilla notabilis* (Meigen, 1824)) from Eritrea was likely based on a misidentification.

***nemorilloides*** (Bezzi, 1923).—Afrotropical: Seychelles.

*Exorista nemorilloides* Bezzi, 1923: 101. Syntypes, 1 male and 1 female (BMNH). Type locality: Seychelles, Silhouette Is., near coast.

Undetermined sp.: Yemen, as “Nemorilla cf. maculosa (Meigen, 1824)” (Zeegers 2007: 392).

##### Genus *OSSIDINGIA* Townsend, 1919

***OSSIDINGIA*** Townsend, 1919a: 179. Type species: *Ossidingia ornata* Townsend, 1919 (= *Tachina cruciata* Wiedemann, 1830), by original designation.

*JESUIMYIA* Townsend, 1926b: 541. Type species: *Tachina cruciata* Wiedemann, 1830, by original designation.

Note: *Ossidingia* Townsend, 1919 was treated as a synonym of *Nemorilla* Rondani, 1856 by Crosskey (1980b: 863) but was later recognized as a genus by Crosskey (1984: 201, 274).

***cruciata*** (Wiedemann, 1830).—Afrotropical: Burundi, Cameroon, D.R. Congo, Kenya, Malawi, Rwanda, South Africa, Tanzania, Uganda.

*Tachina cruciata* Wiedemann, 1830: 326. Syntypes, males and females (4 males and 1 female in NHMW, 2 syntypes in ZMUC [Zimsen 1954: 22]). Type locality: South Africa, Western Cape, Cape of Good Hope [as “Kap”].

*Tachina concisa* Walker, 1853: 280. Type(s), female (BMNH). Type locality: South Africa, Western Cape, Cape of Good Hope [as “Cape”].

*Tachina ornata* Walker, 1853: 282. Type(s), female (BMNH). Type locality: South Africa, Western Cape, Cape of Good Hope [as “Cape”].

*Ossidingia ornata* Townsend, 1919a: 179 (junior secondary homonym of *Tachina ornata* Walker, 1853). Holotype female (USNM). Type locality: Cameroon, Ossidinge [ca. 5°53′N 9°07′E].

##### Genus *SMIDTIA* Robineau-Desvoidy, 1830

***SMIDTIA*** Robineau-Desvoidy, 1830: 183. Type species: *Smidtia vernalis* Robineau-Desvoidy, 1830 (= *Tachina conspersa* Meigen, 1824), by subsequent designation of Desmarest *in* d’Orbigny (1848: 649) (see Evenhuis and Thompson 1990: 238) [Palaearctic].

*TIMAVIA* Robineau-Desvoidy, 1863a: 257. Type species: *Smidtia flavipalpis* Robineau-Desvoidy, 1848 (= *Tachina amoena* Meigen, 1824), by original designation [Palaearctic].

*OMOTOMA* Lioy, 1864: 1338. Type species: *Tachina amoena* Meigen, 1824, by subsequent designation of Townsend (1916b: 8).

*NEMOSTURMIA* Townsend, 1926a: 34. Type species: *Nemosturmia pilosa* Townsend, 1926 (= *Winthemia fumiferanae* Tothill, 1912), by original designation.

*HOMOTOMA* Bezzi & Stein, 1907: 257. Unjustified emendation of *Omotoma* Lioy, 1864 (junior homonym of *Homotoma* Guérin, 1844).

Note: Bezzi and Stein (1907: 257) emended the name *Omotoma* to *Homotoma* but treated the latter as a junior synonym of *Nemorilla* Rondani, 1856. *Homotoma* has been commonly cited in the literature as a justified emendation (e.g., Mesnil 1949b) or an unjustified emendation (e.g., Herting 1984: 37, O’Hara and Wood 2004: 205) but seemingly always as a junior synonym of another generic name. Unless *Homotoma* was used as a valid name before 1961, it is an unavailable name and thus an incorrect subsequent spelling of *Omotoma* (Article 11.6.1 of the *Code*, ICZN 1999).

***capensis*** (Schiner, 1868).—Afrotropical: South Africa.

*Nemoraea capensis* Schiner, 1868: 329. Holotype male (NHMW, not located by JEOH). Type locality: South Africa, Western Cape, Cape of Good Hope [as “Cap”].

*Hemiwinthemia francoisi* Verbeke, 1973: 4. Holotype female (IRSNB). Type locality: Western Cape, Ceres District, Michell’s Pass [also known as Mitchell’s Pass]. **Syn. n.**

Note: Shima (1996: 174) synonymized *Timavia* Robineau-Desvoidy, 1863 with *Smidtia* Robineau-Desvoidy, 1830 but did not consider the Afrotropical species *Nemoraea capensis* Schiner, 1868 (which Crosskey 1980b: 863 had assigned to *Timavia*) in his redefinition of *Smidtia*. The combination *Smidtia capensis* (Schiner) was first published by Cerretti et al. (2013: 27).

*Hemiwinthemia francoisi* Verbeke, 1973 was overlooked by Crosskey (1980b) but was recorded from the Afrotropical Region without study or change in genus by Crosskey (1984: 201). The synonymy here is based on study of the holotype by PC.

##### Genus *WINTHEMIA* Robineau-Desvoidy, 1830

***WINTHEMIA*** Robineau-Desvoidy, 1830: 173. Type species: *Musca quadripustulata* Fabricius, 1794, by subsequent designation of Desmarest *in* d’Orbigny (1849b: 301) (see Evenhuis and Thompson 1990: 239) [Palaearctic].

*WINTHEMYA* Rondani, 1859: 103. Unjustified emendation of *Winthemia* Robineau-Desvoidy, 1830 (see O’Hara et al. 2011: 188).

*CROSSOTOCNEMA* Bigot, 1885: cci [also 1886: cci]. Type species: *Crossotocnema javana* Bigot, 1885, by monotypy [Oriental].

*SERICOPHOROMYIA* Austen, 1909: 95. Type species: hereby fixed under Article 70.3.2 of the *Code* (ICZN 1999) as *Tachina quadrata* Wiedemann, 1830, misidentified as *Tachina dasyops* Wiedemann, 1824 in the original designation by Austen (1909).

*PSEUDOKEA* Townsend, 1927c: 69. Type species: *Pseudokea sumatrana* Townsend, 1927, by monotypy (see Evenhuis et al. 2015: 233) [Oriental].

*SERICOPHOROMYIOPS* Townsend, 1933: 470. Type species: *Tachina dasyops* Wiedemann, 1824, by original designation.

*WINTHEMIOLA* Mesnil, 1949b: 80 (as subgenus *Winthemia* Robineau-Desvoidy, 1830). Type species: Winthemia (Winthemiola) madecassa Mesnil, 1949, by monotypy.

*SERICOPHOROMYA*. Incorrect subsequent spelling of *Sericophoromyia* Austen, 1909 (Villeneuve 1916c: 480).

*WINTHEMYIA*. Incorrect subsequent spelling of *Winthemia* Robineau-Desvoidy, 1830 (Pantel 1910: 34, etc., Villeneuve 1910b: 305, Villeneuve 1913c: 32).

***amplipilosa*** (Curran, 1928).—Afrotropical: South Africa.

*Sericophoromyia amplipilosa* Curran, 1928a: 241. Holotype female (SANC; not BMNH, see Arnaud and Owen 1981: 234). Type locality: South Africa, Mpumalanga, Barberton.

***australis*** Mesnil, 1949.—Afrotropical: Réunion.

Winthemia (Crossotocnema) australis Mesnil, 1949b: 83. Holotype male (MNHN). Type locality: Réunion, Cilaos.

***candida*** Mesnil, 1977.—Afrotropical: Madagascar.

*Winthemia candida* Mesnil, 1977b: 173. Holotype male (MNHN). Type locality: Madagascar, Toliara, Sakaraha.

***claripilosa*** (Austen, 1909).—Afrotropical: Malawi, Tanzania, Uganda.

*Sericophoromyia claripilosa* Austen, 1909: 96. Holotype male (BMNH). Type locality: Uganda, east Rwenzori Range [as “E. Ruwenzori”], Mubuku Valley, 5000–7000ft.

*clarissima*. Incorrect subsequent spelling of *claripilosa* Austen, 1909 (original usage not found but spelling listed by Crosskey 1980b: 864).

***conformis*** (Curran, 1928).—Afrotropical: D.R. Congo, Kenya, Malawi, South Africa, Uganda.

*Sericophoromyia conformis* Curran, 1928a: 242. Holotype male (SANC). Type locality: South Africa, KwaZulu-Natal, Port Shepstone.

*Sericophoromyia sericea* Curran, 1928a: 240. Holotype male (BMNH). Type locality: Uganda, Rwenzori Range [as “Mount Ruwenzori”].

Note: The relative priority of *Sericophoromyia conformis* Curran, 1928 and *Sericophoromyia sericea* Curran, 1928, when the two are treated as synonyms, was established by Crosskey (1980b: 864), as the First Reviser (Article 24.2.2 of the *Code*, ICZN 1999).

***cylindrica*** (Villeneuve, 1938).—Afrotropical: D.R. Congo.

*Sericophoromyia cylindrica* Villeneuve, 1938c: 15. Syntypes, males and females (1 male in IRSNB, 1 male and 1 female in MRAC). Type localities: D.R. Congo, Équateur, Eala and Katanga, Lubumbashi [as “Elisabethville”].

***dasyops*** (Wiedemann, 1824).—Afrotropical: D.R. Congo, Ethiopia, Ghana, Kenya, Madagascar, Malawi, Mozambique, Nigeria, South Africa, Tanzania, Uganda, Yemen.

*Tachina dasyops* Wiedemann, 1824: 42. Lectotype male (ZMUC), by fixation of Townsend (1932: 47) (examination of “Male Ht” from “Cape Good Hope” in ZMUC [as “Westermann Coll.”] is regarded as a lectotype fixation). Type locality: South Africa, Western Cape, Cape of Good Hope [as “Prom. bon. sp.” = “Promontorium Bonae Spei”].

*Sericophoromyia marshalli* Villeneuve, 1915b: 195. Syntypes, males and females (not located). Type localities: Ghana (Aburi), Madagascar (Antananarivo, Antananarivo [as “Tananarive”]), Malawi (Mt. Mulanje [as “Mt. Mlanje”]), and Mozambique.

Note: Villeneuve (1916c: 480) described *Sericophoromyia marshalli* Villeneuve, 1915 a second time (spelling the generic name as “*Sericophoromya*”), explaining that the original description “was to be published abroad, but of which I heard nothing since the beginning of the war”. Townsend’s (1932: 47) mention of “male Ht” of *Sericophoromyia marshalli* in SAMC from “Natal” cannot be accepted as a lectotype fixation because Natal was not among the type localities listed by Villeneuve (1915b: 196). Villeneuve’s (1916c: 480) second description of *Sericophoromyia marshalli* included material from “Natal, Durban” in SAMC, but only material listed by Villeneuve (1915b) belongs to the type series of *Sericophoromyia marshalli*. Similarly, the “paratype” of *Sericophoromyia marshalli* in MSNM from South Africa examined by Arnaud (1982: 13) was not part of Villeneuve’s (1915b) type series.

***fasciculata*** Villeneuve, 1921.—Afrotropical: Ghana, Kenya, Malawi, Nigeria.

*Winthemia fasciculata* Villeneuve, 1921: 29. Syntypes, males and females (“Plusieurs individus des deux sexes”) (BMNH). Type localities: Ghana (Aburi) and Malawi (Mt. Mulanje [as “Mont Mlanjé”]).

***ignicornis*** Mesnil, 1977.—Afrotropical: Madagascar.

*Winthemia ignicornis* Mesnil, 1977b: 172. Holotype male (MNHN). Type locality: Madagascar, Ambohitantely [Réserve Spéciale, ca. 18°10′S 47°17′E].

***madecassa*** Mesnil, 1949.—Afrotropical: D.R. Congo, Madagascar.

Winthemia (Winthemiola) madecassa Mesnil, 1949b: 82. Holotype male (MNHN). Type locality: Madagascar, Toamasina, Rogez [ca. 18°48′S 48°37′E].

***masicerana*** (Villeneuve, 1937).—Afrotropical: Mauritius.

*Sericophoromyia masicerana* Villeneuve, 1937b: 1. Syntypes, 2 males (not located). Type locality: Mauritius.

***quadrata*** (Wiedemann, 1830).—Afrotropical: “widespread E. & sthn Afr.” (Crosskey 1980b: 864), including Cameroon, D.R. Congo, Ethiopia, Rwanda, Somalia, South Africa, Tanzania, Yemen.

*Tachina quadrata* Wiedemann, 1830: 318. Type(s), unspecified sex (2 syntypes in ZMUC, Zimsen 1954: 22). Type locality: South Africa, Western Cape, Cape of Good Hope [as “Kap”].

*Sericophoromyia lanuginosa* Speiser, 1910: 140. Holotype female (NHRS). Type locality: Tanzania, Mt. Kilimanjaro [as “Kilimandjaro”].

*Tachina dasyops* of Austen (1909: 95), not Wiedemann, 1824. Misidentification (Crosskey 1980b: 864).

Note: *Tachina dasyops* of Austen (1909: 95) was interpreted as synonymous with *Sericophoromyia amplipilosa* Curran, 1928 by Townsend (1932: 47), but the synonymy of Crosskey (1980b: 864) is followed here.

***ruficrura*** (Villeneuve, 1916).—Afrotropical: Ghana, Kenya, Malawi, Mozambique, Nigeria, Tanzania, Uganda.

*Sericophoromya ruficrura* Villeneuve, 1916c: 481. Syntypes, unspecified number and sex (BMNH). Type localities: Ghana (Aburi [as “Ahuri”]) and Malawi (Mt. Mulanje [as “Mt. Mlanje”]), and Mozambique.

***terrosa*** Villeneuve, 1913.—Afrotropical: Ghana, Nigeria, Uganda.

*Winthemyia terrosa* Villeneuve, 1913c: 32. Holotype female (BMNH). Type locality: Uganda, “Prot. Daro or Duro Forest, Toro” [Duro Forest not located; Toro is a kingdom in western Uganda that occupies a large area between Lake Albert and Lake Edward], 4000–4500ft.

Undescribed spp.: South Africa, Uganda (two undescribed species in BMNH and CNC with atypical features and thus of uncertain generic assignment, Crosskey 1984: 275).

#### Unplaced species of Exoristinae

***boscii*** Macquart, 1844.—Afrotropical: Mauritius.

*Lydella boscii* Macquart, 1844: 60 [also 1844: 217]. Type(s), male (“presumed lost”, Crosskey 1971: 272). Type locality: Mauritius [as “l’île de France”].

***brunnescens*** Becker, 1909.—Afrotropical: Kenya.

*Pseudophorocera brunnescens* Becker, 1909a: 117. Holotype male (MNHN). Type locality: Kenya [as “Afrique orientale anglaise; Escarpment”, interpreted as Kenya by Crosskey 1980b: 881].

Note: The description of *Pseudophorocera brunnescens* Becker, 1909 is repeated in Becker (1910a: 26) under the heading “*Pseudophorocera brunnescens*, n. sp. ♂”.

***caffra*** Macquart, 1846.—Afrotropical: South Africa.

*Masicera caffra* Macquart, 1846: 290 [also 1846: 162]. Type(s), female (“presumed lost”, Crosskey 1971: 273). Type locality: South Africa, “Cafrerie” (also as “Kaffraria”; probably referring to an area now in the southeastern part of Eastern Cape).

***echinaspis*** Bezzi, 1908.—Afrotropical: Eritrea.

*Exorista echinaspis* Bezzi, 1908b: 53. Syntypes, 2 males (not located, not among the labelled types of Bezzi in MSNM examined by Arnaud 1982). Type locality: Eritrea, near Adi Keyh [also as Adi Kaie and other spellings, published as “Adi Caiè”, ca. 14°51′N 39°22′E].

***excoriata*** Wiedemann, 1830.—Afrotropical: South Africa.

*Tachina excoriata* Wiedemann, 1830: 316. Type(s), male (NHMW, “Type” seen by Brauer and Bergenstamm 1891: 343 [also 1891: 39]; not located by JEOH in the Afrotropical portion of the collection but possibly placed elsewhere). Type locality: not given (cited as “?South Africa” by Crosskey 1980b: 881).

*excoricata*. Incorrect original spelling of *excoriata* Wiedemann, 1830 (Wiedemann 1830: 679).

Note: There are two original spellings for *Tachina excoriata* in Wiedemann (1830): *excoriata* in the text (p. 316) and *excoricata* in the index (p. 679). The correct original spelling was selected as *excoriata* by Bezzi (1911: 59), as the First Reviser (Article 24.2.3 of the *Code*, ICZN 1999). Bezzi (1911) examined a female of this species from Pretoria and this is probably the basis for Crosskey (1980b: 881) suggesting South Africa as the likely country of origin of the type(s) of *Tachina excoriata*.

***liliputiana*** Bezzi, 1923.—Afrotropical: Seychelles.

*Discochaeta liliputiana* Bezzi, 1923: 94. Holotype female (BMNH). Type locality: Seychelles, Mahé Is., Cascade Estate, ca. 800ft.

***polleniina*** Bezzi, 1908.—Afrotropical: Eritrea.

*Ctenophorocera polleniina* Bezzi, 1908b: 56. Syntypes, 2 females (not located, not among the labelled types of Bezzi in MSNM examined by Arnaud 1982). Type locality: Eritrea, near Adi Keyh [also as Adi Kaie and other spellings, published as “Adi Caiè”, ca. 14°51′N 39°22′E].

***pretoriensis*** Bezzi, 1911.—Afrotropical: South Africa.

*Archiclops pretoriensis* Bezzi, 1911: 61. Holotype female (USNM). Type locality: South Africa, Gauteng, Pretoria.

***setibarba*** Bezzi, 1908.—Afrotropical: Eritrea.

*Erynnia setibarba* Bezzi, 1908b: 55. Syntypes, 1 male and 1 female (not located, not among the labelled types of Bezzi in MSNM examined by Arnaud 1982). Type locality: Eritrea, Keren [ca. 15°47′N 38°27′E].

### Subfamily PHASIINAE (Fig. 5)

**Figure 5.**
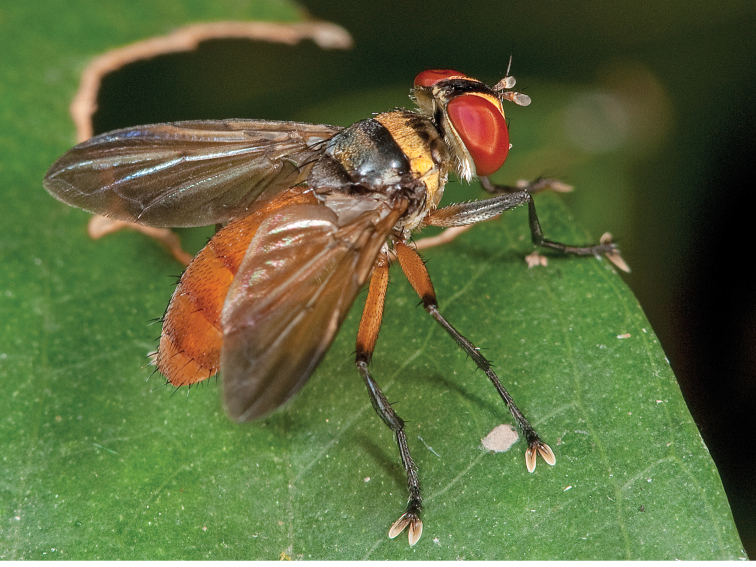
Live specimen of *Bogosia* sp. (Gymnosomatini, Phasiinae) from Magombera Forest near Mangula, Tanzania (image courtesy of S.A. Marshall).

#### Tribe CATHAROSIINI

##### Genus *CATHAROSIA* Rondani, 1868

***CATHAROSIA*** Rondani, 1868a: 46. Type species: *Thereva pygmaea* Fallén, 1815, by original designation [Palaearctic].

*ARCHIPHANIA* van Emden, 1945: 397. Type species: *Archiphania alutacea* van Emden, 1945, by monotypy. **Syn. revived.**

Note: *Archiphania* van Emden, 1945 was treated as a genus by Crosskey (1980b: 825) but was synonymized with *Catharosia* Rondani, 1868 by Crosskey (1984: 200). Zeegers (2007: 401) treated *Archiphania* as a genus but we agree with the synonymy of Crosskey (1984: 200).

***alutacea*** (van Emden, 1945).—Afrotropical: Angola, D.R. Congo, Kenya, Nigeria, Yemen.

*Archiphania alutacea* van Emden, 1945: 398. Holotype male (BMNH). Type locality: Kenya, Embu.

***capensis*** Verbeke, 1970.—Afrotropical: South Africa.

*Catharosia capensis* Verbeke, 1970: 295. Holotype male (MZLU). Type locality: South Africa, Western Cape, Cape Peninsula, Hout Bay, Skoorsteenkop.

***valescens*** Villeneuve, 1942.—Afrotropical: D.R. Congo (**new record**, IRSNB [Verbeke det.]), Kenya, South Africa, Zimbabwe.

*Catharosia valescens* Villeneuve, 1942a: 55. Holotype female (not located). Type locality: Zimbabwe, Hurungwe [as “Urungwe”], Gota Gota.

Undescribed sp.: Madagascar (CNC [as *Archiphania alutacea* van Emden, det. L.P. Mesnil], examined by PC).

##### Genus *LITOPHASIA* Girschner, 1887

***LITOPHASIA*** Girschner, 1887: 380. Type species: *Thereva hyalipennis* Fallén, 1815, by subsequent designation of Brauer (1893: 497) [Palaearctic].

*LITHOPHASIA*. Incorrect subsequent spelling of *Litophasia* Girschner, 1887 (Verbeke 1962a: 89, etc.).

***sulcifacies*** Dear, 1980.—Afrotropical: South Africa.

*Litophasia sulcifacies* Dear, 1980: 218. Holotype male (BMNH). Type locality: South Africa, Eastern Cape, Port Elizabeth.

Note: *Litophasia sulcifacies* Dear, 1980 was referred to as an “Undescribed sp.” from South Africa by Crosskey (1980b: 825).

Undescribed sp.: Tanzania (ZMUC, examined by PC).

#### Tribe CYLINDROMYIINI

##### Genus *BESSERIA* Robineau-Desvoidy, 1830

***BESSERIA*** Robineau-Desvoidy, 1830: 232. Type species: *Besseria reflexa* Robineau-Desvoidy, 1830, by monotypy [Palaearctic].

*APOSTROPHUS* Loew, 1871: 310, 311. Type species: *Apostrophus suspectus* Loew, 1871 (= *Actia zonaria* Loew, 1847), by subsequent designation of Coquillett (1910: 509) (see O’Hara and Wood 2004: 213) [Palaearctic].

*APOSTROPHUSIA* Townsend, 1933: 454. Type species: *Apostrophus anthophilus* Loew, 1871, by original designation [Palaearctic].

Note: The type species of *Apostrophus* Loew, 1871 was first designated by Coquillett (1910: 509), as discussed by Sabrosky and Arnaud (1965: 972) and O’Hara and Wood (2004: 213). The designation by Dupuis (1958: 693), which was followed by Crosskey (1980b: 826), Herting and Dely-Draskovits (1993: 433) and others, was later.

***caffra*** Villeneuve, 1920.—Afrotropical: South Africa.

*Besseria capensis* Brauer & Bergenstamm, 1891: 411 [also 1891: 107] (as “*capensis* S. litt. Cap b. sp. [Cape of Good Hope]”). *Nomen nudum*.

*Besseria caffra* Villeneuve, 1920a: 155. Syntypes, males and females (not located). Type locality: South Africa, Eastern Cape, Willowmore.

***excavata*** Herting, 1979.—Afrotropical: Madagascar.

*Besseria excavata* Herting, 1979a: 8. Holotype male (CNC). Type locality: Madagascar, Antananarivo, Antananarivo [as “Tananarive”].

***fossulata*** Bezzi, 1908.—Afrotropical: D.R. Congo, Madagascar, South Africa, Yemen. Palaearctic: M. East (M. East). **Status revived.**

*Besseria fossulata* Bezzi, 1908c: 383. Holotype male (IRSNB). Type locality: D.R. Congo, Bas-Congo, Banana to Boma.

*Besseria longicornis* Zeegers, 2007: 402. Holotype male (RMNH). Type locality: Yemen, 12km northwest of Manākhah (15°04′19″N 43°44′27″E). **Syn. n.**

Note: *Besseria fossulata* Bezzi, 1908 was treated as a synonym of *Besseria zonaria* (Loew, 1847) by Crosskey (1980b: 826) but is recognized here as a distinct species based on study of the holotype by PC. *Besseria longicornis* Zeegers, 2007 is newly recognized as a junior synonym of *Besseria fossulata*.

***oblita*** Herting, 1979.—Afrotropical: Namibia, South Africa.

*Besseria oblita* Herting, 1979a: 7. Holotype male (BMNH). Type locality: Namibia, Regenstein, 25km SSW of Windhoek.

***zonaria*** (Loew, 1847).—Afrotropical: Ethiopia, South Africa, Tanzania. Palaearctic: C. Asia, Europe (SW. Eur., SC. Eur., SE. Eur., Turkey), Kazakhstan, M. East (Israel), Russia (W. Russia).

*Actia zonaria* Loew, 1847: 275. Holotype male [published as “♀?”, examined by JEOH] (ZMHB). Type locality: Italy, Sicily, Siracusa [as “Syrakus”].

##### Genus *CATAPARIPROSOPA* Townsend, 1927

***CATAPARIPROSOPA*** Townsend, 1927b: 285. Type species: *Catapariprosopa curvicauda* Townsend, 1927, by original designation [Oriental].

*HEMIPHANIA* Villeneuve, 1937a: 205. Type species: *Hemiphania trispina* Villeneuve, 1937, by monotypy.

*PHANIOLA* Mesnil, 1978b: 285. Type species: *Phaniola cyanella* Mesnil, 1978, by original designation.

Note: *Phaniola* Mesnil, 1978 (with seven new species) and *Hemiphania cilipes* Mesnil, 1978 were published too late to be included in Crosskey’s (1980b) chapter on Afrotropical Tachinidae but were listed in the Appendix of the Afrotropical catalogue under “List of additional taxa since going to press” (Crosskey 1980a: 1224). *Hemiphania* was treated as a synonym of *Catapariprosopa* Townsend, 1927 by Crosskey (1980b: 826) and *Phaniola* was similarly treated by Crosskey (1984: 195).

A key to the two mainland Afrotropical species of *Catapariprosopa* Townsend, 1927 was given by Herting (1979b: 13) and a key to the species of Madagascar (as *Hemiphania* Villeneuve, 1937 and *Phaniola* Mesnil, 1978) was given by Mesnil (1978b: 286).

***cerina*** (Mesnil, 1978).—Afrotropical: Madagascar.

*Phaniola cerina* Mesnil, 1978b: 288. Holotype female (MNHN). Type locality: Madagascar, Toamasina, road from Anosibe An’ Ala [as “Anosibe”] to Moramanga, 840m.

***cilipes*** (Mesnil, 1978).—Afrotropical: Madagascar.

*Hemiphania cilipes* Mesnil, 1978b: 288. Holotype male (MNHN). Type locality: Madagascar, Antananarivo, Ambatolampy [ca. 19°23′S 47°26′E], “Andranotobaka” [not located], 1400m.

Note: *Hemiphania cilipes* Mesnil, 1978 was assigned to *Catapariprosopa* Townsend, 1927 in the Appendix to *Catalogue of the Diptera of the Afrotropical Region* (Crosskey 1980a: 1230).

***cultellifera*** (Mesnil, 1978).—Afrotropical: Madagascar.

*Phaniola cultellifera* Mesnil, 1978b: 288. Holotype male (MNHN). Type locality: Madagascar, Toamasina, Périnet [ca. 18°55′S 48°25′E].

***cumatilis*** (Mesnil, 1978).—Afrotropical: Madagascar.

*Phaniola cumatilis* Mesnil, 1978b: 287. Holotype female (MNHN). Type locality: Madagascar, Toamasina, Périnet [ca. 18°55′S 48°25′E].

***cyanella*** (Mesnil, 1978).—Afrotropical: Madagascar.

*Phaniola cyanella* Mesnil, 1978b: 287. Holotype male (MNHN). Type locality: Madagascar, Toamasina, Périnet [ca. 18°55′S 48°25′E].

***edwardsi*** (van Emden, 1945).—Afrotropical: D.R. Congo (**new record**, IRSNB [PC]), Kenya, Uganda.

*Phania edwardsi* van Emden, 1945: 402. Holotype male (BMNH). Type locality: Uganda, Rwenzori Range [as “Ruwenzori”], Kilembe, 4500ft.

***liturata*** (Mesnil, 1978).—Afrotropical: Madagascar.

*Phaniola liturata* Mesnil, 1978b: 287. Holotype male (MNHN). Type locality: Madagascar, Toamasina, Périnet [ca. 18°55′S 48°25′E].

***nigrapex*** (Mesnil, 1978).—Afrotropical: Madagascar.

*Phaniola nigrapex* Mesnil, 1978b: 288. Holotype female (MNHN). Type locality: Madagascar, Toamasina, Périnet [ca. 18°55′S 48°25′E].

***russipes*** (Mesnil, 1978).—Afrotropical: Madagascar.

*Phaniola russipes* Mesnil, 1978b: 288. Holotype female (MNHN). Type locality: Madagascar, Toamasina, Périnet [ca. 18°55′S 48°25′E].

***trispina*** (Villeneuve, 1937).—Afrotropical: Uganda.

*Hemiphania trispina* Villeneuve, 1937a: 205. Holotype male (CNC). Type locality: Uganda, Rwenzori Range [as “Ruwenzori”], 1800m.

##### Genus *CONOPOMIMA* Mesnil, 1978

***CONOPOMIMA*** Mesnil, 1978b: 289 Type species: *Conopomima bisetosa* Mesnil, 1978, by original designation.

Note: *Conopomima* Mesnil, 1978 was published too late to be included in Crosskey’s (1980b) chapter on Afrotropical Tachinidae but was listed in the Appendix of the Afrotropical catalogue under “List of additional taxa since going to press” (Crosskey 1980a: 1224).

***bisetosa*** Mesnil, 1978.—Afrotropical: Madagascar.

*Conopomima bisetosa* Mesnil, 1978b: 290. Holotype female (MNHN). Type locality: Madagascar, Toliara, near Tôlanaro [also commonly known as Taolagnaro or Fort Dauphin and published as the latter], “forêt d’Isaka”, 225m [Isaka not located].

##### Genus *CYLINDROMYIA* Meigen, 1803

***CYLINDROMYIA*** Meigen, 1803: 279. Type species: *Musca brassicaria* Fabricius, 1775, by monotypy [Palaearctic].

*OCYPTERA* Latreille, 1804: 195. Type species: *Musca brassicaria* Fabricius, 1775, by subsequent designation of Curtis (1837: 629).

*EXOGASTER* Rondani, 1856: 78. Type species: *Exogaster carinatus* Rondani, 1856 (= *Ocyptera rufifrons* Loew, 1844), by original designation (see O’Hara et al. 2011: 85) [Palaearctic].

*OCYPTERULA* Rondani, 1856: 78. Type species: *Ocyptera pusilla* Meigen, 1824, by original designation [Palaearctic].

*PLESIOCYPTERA* Brauer & Bergenstamm, 1893: 56 [also 1893: 144]. Type species: *Ocyptera bicolor* Wiedemann, 1819 (junior primary homonym of *Ocyptera bicolor* Olivier, 1811; = *Ocyptera rubida* Loew, 1854), by monotypy [Oriental].

*CONOPISOMA* Speiser, 1910: 144. Type species: *Conopisoma miraculum* Speiser, 1910, by original designation.

*FORMICOCYPTERA* Townsend, 1933: 451. Type species: *Ocyptera atrata* Fabricius, 1805, by original designation.

*CYLINDROMYA*. Incorrect subsequent spelling of *Cylindromyia* Meigen, 1803 (numerous works).

Note: Subgenera of *Cylindromyia* Meigen, 1803 are not recognized here because the subgeneric placements of the Afrotropical species require more study.

***aberrans*** (Villeneuve, 1936).—Afrotropical: D.R. Congo, Kenya, Uganda.

*Ocyptera aberrans* Villeneuve, 1936b: 2. Holotype female (CNC). Type locality: D.R. Congo, Nord-Kivu, “Moho Lesse” [Lesse at ca. 0°45′N 29°46′E, Moho is presumed to be nearby].

***atrata*** (Fabricius, 1805).—Afrotropical: D.R. Congo, Nigeria, Sierra Leone, Sudan, Uganda.

*Ocyptera atrata* Fabricius, 1805: 313. Lectotype male (ZMUC), by fixation of Townsend (1931: 389) (examination of “Male Ht” from Guinea in ZMUC [as “Copenhagen Fab. Coll.”] is regarded as a lectotype fixation). Type locality: “Guinea” (referring to West Africa).

***aurohumera*** (van Emden, 1945).—Afrotropical: Sudan.

*Ocyptera aurohumera* van Emden, 1945: 407. Holotype male (BMNH). Type locality: Sudan, Darfur, Meidob [as “Midob”], plain below Jabal [as “J.”] Kaboija [ca. 14°58′N 26°36′E].

***braueri*** O’Hara & Cerretti, **nom. n.**—Afrotropical: D.R. Congo, Ethiopia, Kenya, Mozambique (**new record**, JOS [PC]), Rwanda (**new record**, IRSNB [PC]), South Africa, Tanzania, Uganda, Yemen, Zimbabwe (see note).

*Ocyptera nigra* Brauer & Bergenstamm, 1891: 408 [also 1891: 104] (as “*nigra* Wd. ltt. Afrika”). *Nomen nudum*.

*Ocyptera nigra* Villeneuve, 1918: 504 (as “*nigra* Br. et Berg. (in litt.)”) (junior secondary homonym of *Glossidionophora nigra* Bigot, 1885). Holotype, unspecified sex [female, examined by JEOH] (NHMW). Type locality: not given (“Afrika” according to Brauer and Bergenstamm 1891: 408 [also 1891: 104] and data label of holotype).

*Cylindromyia braueri* O’Hara & Cerretti, **nom. n.** for *Ocyptera nigra* Villeneuve, 1918.

*Ocyptera nigra* of Crosskey (1980b: 827, as synonym of *Cylindromyia rufipes* (Meigen, 1824)), not Villeneuve, 1918. Misidentification (see note).

Note: *Ocyptera nigra* Villeneuve, 1918 is a junior secondary homonym of *Glossidionophora nigra* Bigot, 1885, the valid name of a Neotropical species of *Cylindromyia* (Guimarães 1976: 6). We hereby propose the new name *Cylindromyia braueri* to replace the preoccupied name *Ocyptera nigra* Villeneuve. The same type material applies to the new name. The specific epithet *braueri* is proposed in honour of the 19th Century dipterist Friedrich Brauer of the Naturhistorisches Museum in Vienna (NHMW) who, along with J.E. von Bergenstamm, first published a name for this species, albeit as a *nomen nudum*.

Although *Cylindromyia braueri*, as *Ocyptera nigra* Villeneuve, 1918, was treated by Crosskey (1980b: 827) as a synonym of *Ocyptera rufipes* Meigen, 1824, it was recognized as a distinct species earlier by both Curran (1934a: 130) and van Emden (1945: 410) and recently by Zeegers (2007: 403). Herting (1983b: 85), in his treatment of Palaearctic *Cylindromyia*, did not discuss *Cylindromyia nigra* but presumably considered it as separate from *Cylindromyia rufipes* because he did not record the latter from the Afrotropical Region. Similarly, Herting and Dely-Draskovits (1993: 429) did not record *Cylindromyia rufipes* from the Afrotropics. The distribution of *Cylindromyia braueri* given here is based on the records in Curran (1934a), van Emden (1945) and Zeegers (2007).

***completa*** Curran, 1927.—Afrotropical: D.R. Congo.

*Cylindromyia completa* Curran, 1927b: 3. Holotype male (AMNH). Type locality: D.R. Congo, Orientale, Faradje.

***deserta*** (Villeneuve, 1936).—Afrotropical: Nigeria.

*Ocyptera deserta* Villeneuve, 1936b: 2. Holotype male (CNC). Type locality: northern Nigeria.

***eronis*** Curran, 1927.—Afrotropical: Cape Verde, D.R. Congo, Ghana, Malawi, Somalia, South Africa, Uganda.

*Cylindromyia eronis* Curran, 1927b: 3. Holotype female (AMNH). Type locality: D.R. Congo, Orientale, Kisangani [as “Stanleyville”].

*Cylindromyia incerta* Curran, 1934a: 132. Holotype female (BMNH). Type locality: South Africa, KwaZulu-Natal, Durban.

? *Ocyptera cribrata* Villeneuve, 1936b: 3. Syntypes, 1 male and 1 female (1 female in CNC). Type localities: D.R. Congo and South Africa (Eastern Cape, Algoa Bay).

Note: *Cylindromyia eronis* Curran, 1927 was treated as a synonym of *Cylindromyia miracula* (Speiser, 1910) by Herting (1979b: 9) and as a distinct species by Crosskey (1980b: 826). It is recognized here as a species (with synonymy as given by Crosskey 1980b) based on examination of the holotype by PC.

***ethelia*** Curran, 1934.—Afrotropical: South Africa (**new record**, NMDA [PC]), Uganda.

*Cylindromyia ethelia* Curran, 1934a: 126. Holotype male (BMNH). Type locality: Uganda, Kampala.

***flavibasis*** (Villeneuve, 1916).—Afrotropical: Burundi (**new record**, IRSNB [PC]), D.R. Congo (**new record**, IRSNB [PC]), South Africa, Uganda (**new record**, NMDA [PC]), Zambia, Zimbabwe.

*Ocyptera flavibasis* Villeneuve, 1916c: 506. Syntypes, 2 males (SAMC, not located by JEOH). Type localities: South Africa, KwaZulu-Natal, Mfongosi [as “Zululand, M’Fongosi”] and “Transvaal” (a former province that occupied much of the northeastern part of the country and has since been subdivided into several provinces).

***hemimelaena*** (Bezzi, 1923).—Afrotropical: Seychelles.

*Exogaster hemimelaena* Bezzi, 1923: 92. Holotype male (BMNH). Type locality: Seychelles, Praslin Is.

***lavinia*** Curran, 1934.—Afrotropical: South Africa.

*Cylindromyia lavinia* Curran, 1934a: 129. Holotype female (SANC). Type locality: South Africa, Limpopo, Woodbush.

***marginalis*** (Wiedemann, 1824).—Afrotropical: South Africa.

*Ocyptera marginalis* Wiedemann, 1824: 41. Type(s), unspecified sex (1 syntype in ZMUC, Zimsen 1954: 21). Type locality: South Africa, Western Cape, Cape of Good Hope [as “Prom. bon. sp.” = “Promontorium Bonae Spei”].

Note: *Ocyptera marginalis* Wiedemann, 1824 was described from one or more specimens from Cape of Good Hope. There are two males in NHMW labelled “Cap b. sp” and “*marginalis* Coll. Winthem” (examined by JEOH). These are unlikely to be syntypes because Wiedemann cited the type(s) in “Museo Westerm.”, since incorporated into ZMUC.

***miracula*** (Speiser, 1910).—Afrotropical: D.R. Congo, Tanzania.

*Conopisoma miraculum* Speiser, 1910: 146. Holotype male (NHRS). Type locality: Tanzania, Mt. Kilimanjaro [as “Kilimandjaro”], Kibongoto [as “Kibonoto”].

*Cylindromyia insolitum* Curran, 1927b: 1. Holotype female [not male as published] (AMNH). Type locality: D.R. Congo, Orientale, Kisangani [as “Stanleyville”].

Note: See Herting (1979b: 8) for a redescription of *Conopisoma miraculum* Speiser, 1910.

***ocypteroides*** (Bezzi, 1908).—Afrotropical: Eritrea.

*Exogaster ocypteroides* Bezzi, 1908b: 67. Syntypes, 3 males and 1 female (MSNM [1 “type” according to Arnaud 1982: 13]). Type localities: Eritrea, Sabarguma [ca. 15°31′N 39°6′E] and near Adi Keyh [also as Adi Kaie and other spellings, published as “Adi Caiè”, ca. 14°51′N 39°22′E].

Note: Bezzi (1908b: 67) recorded two males of his new species *Exogaster ocypteroides* from Adi Caiè. He noted that one male and one female reported as “*Ocyptera* sp.” from Sabarguma by Bezzi (1901: 22) also belong to this new species. Since all four specimens apparently contributed to the description, they are all treated as syntypes.

***oxyphera*** (Villeneuve, 1926).—Afrotropical: South Africa.

*Ocyptera oxyphera* Villeneuve, 1926a: 192. Lectotype male (NHMW), by fixation of Curran (1934a: 136) (study and illustration of “type” from Algoa Bay in NHMW is regarded as a lectotype fixation). Type locality: South Africa, Eastern Cape, Algoa Bay.

*Cylindromyia oxyphora* Curran, 1934a: 136. Unjustified emendation of *Ocyptera oxyphera* Villeneuve, 1926.

***pedunculata*** Curran, 1927.—Afrotropical: D.R. Congo.

*Cylindromyia pedunculata* Curran, 1927b: 2. Holotype female (AMNH). Type locality: D.R. Congo, Orientale, Kisangani [as “Stanleyville”].

*pendunculata*. Incorrect subsequent spelling of *pedunculata* Curran, 1927 (Crosskey 1980b: 827).

***pictipennis*** (Macquart, 1835).—Afrotropical: “widespread W. Afr., E. Afr., sthn Afr.” (Crosskey 1980b: 827), including Cameroon, D.R. Congo, Ghana, Nigeria, Senegal, Sierra Leone, South Africa, Tanzania, Uganda, Zimbabwe.

*Ocyptera pictipennis* Macquart, 1835: 186. Lectotype male (MNHN, only one wing and part of thorax remaining according to Crosskey 1971: 280), by fixation of Crosskey (1971: 280) (examination of “Holotype ♂” from Senegal in MNHN is regarded as a lectotype fixation). Type locality: Senegal.

*Ocyptera picta* Walker, 1849: 695. Type(s), unspecified sex (1 female in BMNH according to BMNH database). Type locality: Sierra Leone.

*Ocyptera euprepia* Speiser, 1910: 143. Holotype male (NHRS). Type locality: Tanzania, Mt. Kilimanjaro [as “Kilimandjaro”], Kibongoto [as “Kibonoto”].

***rubida*** (Loew, 1854).—Afrotropical: Yemen. Palaearctic: C. Asia, Europe (SW. Eur., SC. Eur., SE. Eur.) M. East (Israel), N. Africa (NW. Africa). Oriental: India, Sri Lanka (Crosskey 1976: 170, as *Cylindromyia wiedemanni* Crosskey, 1976).

*Ocyptera bicolor* Wiedemann, 1819: 37 (junior primary homonym of *Ocyptera bicolor* Olivier, 1811). Lectotype male (ZMUC), by designation of Crosskey (1966a: 666). Type locality: “India or.” (i.e., “East Indies”; interpreted as India by Crosskey 1966a: 666 and Crosskey 1976: 170).

*Ocyptera rubida* Loew, 1854: 19. Lectotype male (ZMHB), by fixation of Herting (1983b: 82) (examination of “typ[us]” from Dalmatien in ZMHB is regarded as a lectotype fixation for the single syntype in ZMHB [examined by JEOH]). Type locality: Croatia, Dalmacija [as “Dalmatien”].

*Cylindromyia wiedemanni* Crosskey, 1976: 170, 264 (*nomen novum* for *Ocyptera bicolor* Wiedemann, 1819).

Note: Herting (1983b: 80) redescribed *Ocyptera rubida* Loew, 1854 and established *Ocyptera bicolor* Wiedemann, 1819 as a senior (but invalid) synonym.

***rufipes*** (Meigen, 1824).—Afrotropical: U.A. Emirates. Palaearctic: Europe (all except British Is., Scand.), M. East (all), Russia (W. Russia), Transcaucasia. Oriental: India, Pakistan.

*Ocyptera rufipes* Meigen, 1824: 215. Lectotype male (MNHN), by fixation of Crosskey (1976: 170) (examination of “Holotype” from France in MNHN is regarded as a lectotype fixation). Type locality: France.

***rufohumera*** O’Hara & Cerretti, **nom. n.**—Afrotropical: D.R. Congo, Zimbabwe.

*Ocyptera scapularis* Villeneuve, 1944: 145 (junior primary homonym of *Ocyptera scapularis* Loew, 1845). Syntypes, 2 males (1 male in CNC). Type localities: D.R. Congo and Zimbabwe (Vumba Mountains according to label data of CNC syntype, Cooper and O’Hara 1996: 56).

*Cylindromyia rufohumera* O’Hara & Cerretti, **nom. n.** for *Ocyptera scapularis* Villeneuve, 1944.

Note: *Ocyptera scapularis* Villeneuve, 1944 is a junior primary homonym of *Ocyptera scapularis* Loew, 1845, the valid name of a Palaearctic species of *Cylindromyia* (Cerretti 2010: 540). This was noted by Crosskey (1980a: 1230) in the Appendix to *Catalogue of the Diptera of the Afrotropical Region*, who wrote: “No replacement name is here proposed as such may not be needed when Afrotropical *Cylindromyia* species are fully revised ...”. We believe that renaming the Afrotropical species is in the best interests of nomenclatural stability and hereby propose the new name *Cylindromyia rufohumera* to replace the preoccupied name *Ocyptera scapularis* Villeneuve. The same type material applies to the new name. The specific epithet *rufohumera* is formed from the Latin words *rufus* (reddish) and *humerus* (shoulder), alluding to the reddish postpronotal lobes (“épaules”) mentioned in the original description and which likely inspired Villeneuve’s name *scapularis*.

***sensua*** Curran, 1934.—Afrotropical: Botswana, D.R. Congo (**new record**, IRSNB [Verbeke det.]), Tanzania.

*Cylindromyia sensua* Curran, 1934a: 133. Holotype female (BMNH). Type locality: Tanzania, Zanzibar, Pemba Island.

***soror*** (Wiedemann, 1830).—Afrotropical: D.R. Congo (**new record**, IRSNB [PC]), Kenya, Malawi, Nigeria, Réunion (**new record**, photo identification [PC]), South Africa, Sudan, Tanzania, Uganda, Yemen.

*Ocyptera soror* Wiedemann, 1830: 652. Syntypes, unspecified number and sex (2 males and 1 female in NHMW). Type locality: South Africa, Western Cape, Cape of Good Hope [as “Kap”].

*Cylindromyia snelli* Curran, 1934a: 129. Holotype female (BMNH). Type locality: Tanzania, Zanzibar, near “Mazi Moja” (possibly now as Mnazi Mmoja).

*Ocyptera linearis* Villeneuve, 1936b: 2. Lectotype male (IRSNB), by designation herein (see Lectotype Designations section). Type locality: D.R. Congo ([Équateur], Eala according to label data).

***xiphias*** (Bezzi, 1908).—Afrotropical: “widespread W. Afr. to E. Afr. & n.-e. Afr., sthn Afr.” (Crosskey 1980b: 827), including D.R. Congo, Eritrea, Kenya, Malawi, South Africa, Sudan, Tanzania, Uganda, Zambia, Zimbabwe.

*Ocyptera xiphias* Bezzi, 1908b: 65. Type(s), male (not located, not among the labelled types of Bezzi in MSNM examined by Arnaud 1982). Type locality: Eritrea, near Mendefera [as “Adi Ugri”, ca. 14°53′N 38°49′E].

Note: Curran (1934a: 137) was likely in error when he wrote for *Ocyptera xiphias* Bezzi, 1908: “Type in Munro collection; allotype in British Museum (Natural History)”. There are no Bezzi types of Tachinidae in SANC, where Curran types collected by Munro are housed (examined by JEOH). Similarly, we doubt that Munro would have been in possession of a name-bearing type of *Ocyptera xiphias* Bezzi. For these reasons we do not accept Curran’s (1934a: 137) mention of the “Type” of *Ocyptera xiphias* Bezzi, 1908 as a lectotype fixation.

Undescribed sp.: Madagascar (TAU, examined by PC).

##### Genus *PROLOPHOSIA* Townsend, 1933

***PROLOPHOSIA*** Townsend, 1933: 450. Type species: *Prolophosia petiolata* Townsend, 1933, by original designation.

***petiolata*** Townsend, 1933.—Afrotropical: Burundi, D.R. Congo (**new record**, IRSNB [PC]), Kenya, South Africa, Tanzania, Uganda.

*Prolophosia petiolata* Townsend, 1933: 450. Holotype male (NHRS). Type locality: South Africa, “Caffraria” (also known as “Kaffraria”, a former region in Eastern Cape).

*Cylindromyia atypica* Curran, 1934a: 140. Holotype male (BMNH). Type locality: Uganda, Kampala.

*Cylindromyia ugandana* Curran, 1934a: 141. Holotype male (BMNH). Type locality: Uganda, Kampala.

***retroflexa*** (Villeneuve, 1944).—Afrotropical: Uganda. **Comb. n.**

*Ocyptera retroflexa* Villeneuve, 1944: 145. Holotype female (CNC). Type locality: Uganda, Kampala.

Note: *Ocyptera retroflexa* Villeneuve, 1944 was treated as a species of *Cylindromyia* Meigen, 1803 by Crosskey (1980b: 827) but is moved here to *Prolophosia* Townsend, 1933 based on our study of the holotype.

Undescribed sp.: Burundi (MZUR, examined by PC).

#### Tribe GYMNOSOMATINI

##### Genus *BOGOSIA* Rondani, 1873

***BOGOSIA*** Rondani, 1873: 284. Type species: *Bogosia antinorii* Rondani, 1873, by monotypy.

*EPINEURA* Brauer & Bergenstamm, 1891: 388 [also 1891: 84]. Type species: *Phasia helva* Wiedemann, 1818, by subsequent designation of Townsend (1916b: 6).

*ENGELOBOGOSIA* Townsend, 1933: 449. Type species: *Bogosia engeli* Karsch, 1887 (= *Bogosia antinorii* Rondani, 1873), by original designation.

Note: A revision of *Bogosia* Rondani, 1873 was published by Barraclough (1985a). We agree with Tschorsnig (1985: 106) in assigning *Bogosia* to the Gymnosomatini.

***antinorii*** Rondani, 1873.—Afrotropical: Angola, D.R. Congo, Eritrea, Kenya, Madagascar, Malawi, South Africa, Tanzania, Zimbabwe.

*Bogosia antinorii* Rondani, 1873: 284. Lectotype female (MCSN), by fixation of Townsend (1938b: 12) (mention of “Ht” from “Bogos” in MCSN is regarded as a lectotype fixation). Type locality: Eritrea, “Bogos” (a former region).

*Bogosia engeli* Karsch, 1887: 4. Lectotype female (ZMHB, not located by Barraclough 1985a: 352 or by JEOH in 2014), by fixation of Townsend (1933: 450) (mention of “Female holotype” from Pungo Andongo [p. 449] in ZMHB is regarded as a lectotype fixation). Type locality: Angola, Pungo Andongo.

Note: Townsend’s (1931: 389) mention of “Ht in Genoa?, from Abyssinia” for *Bogosia antinorii* Rondani, 1873 is too vague to be accepted as a lectotype fixation. Neither Rondani (1873: 284) nor Townsend (1938b: 12) gave the sex of the name-bearing type of *Bogosia antinorii*, but Barraclough (1985a: 351) examined the “holotype” in MCSN and gave its sex as female. Townsend’s (1931: 388) examination of the “Female Ht” of *Bogosia engeli* Karsch, 1887 in ZMHB is not accepted as a lectotype fixation because he gave the type locality as “West Tanganyika” (i.e., West Tanzania); the type locality is Pungo Andongo in Angola, as cited correctly by Townsend (1933: 449). Townsend (1931: 388) either erred in citing the type locality or examined a non-type specimen.

***argentea*** Barraclough, 1985.—Afrotropical: Angola, South Africa, Zambia.

*Bogosia argentea* Barraclough, 1985a: 366. Holotype male (BMNH). Type locality: Angola, Chianga.

***bequaerti*** Villeneuve, 1913.—Afrotropical: Angola, Burundi, Cameroon, Congo, Côte d’Ivoire, D.R. Congo, Gabon, Ghana, Guinea, Kenya, Malawi, Mozambique, Nigeria, Rwanda, South Africa, Tanzania, Uganda, Zimbabwe.

*Bogosia bequaerti* Villeneuve, 1913c: 45. Holotype female (CNC). Type locality: D.R. Congo, Bas-Congo, Kibombo.

***curvaverpa*** Barraclough, 1985.—Afrotropical: Côte d’Ivoire.

*Bogosia curvaverpa* Barraclough, 1985a: 367. Holotype male (MNHN). Type locality: Côte d’Ivoire, Bouaké.

***grahami*** Barraclough, 1985.—Afrotropical: Ghana.

*Bogosia grahami* Barraclough, 1985a: 357. Holotype male (BMNH). Type locality: Ghana, Obuasi, Ashanti.

***helva*** (Wiedemann, 1818).—Afrotropical: D.R. Congo, Kenya, Malawi, Mozambique, South Africa, Tanzania, Uganda, Zimbabwe.

*Phasia helva* Wiedemann, 1818: 45. Lectotype male (NHMW), by fixation of Townsend (1931: 388) (examination of “Male Ht” from Cape of Good Hope in NHMW is regarded as a lectotype fixation). Type locality: South Africa, Western Cape, Cape of Good Hope.

*Phania taeniata* Wiedemann, 1824: 42. Lectotype female [Wiedemann cited only the male sex, presumably in error] (ZMUC), by designation of Barraclough (1985a: 360). Type locality: South Africa, Western Cape, Cape of Good Hope [as “Prom. bon. sp.” = “Promontorium Bonae Spei”].

*Epineura minor* Villeneuve, 1913c: 45. Lectotype male (MRAC), by fixation of Barraclough (1985a: 360, 363) (examination of “holotype ♂” from Bukama in MRAC [as “KMMA” = “Koninklijk Museum voor midden-Afrika ... Tervuren”) is regarded as a lectotype fixation). Type locality: D.R. Congo, Katanga, Bukama.

*Epineura pellucens* Villeneuve, 1918: 508. *Nomen nudum* (published in synonymy with *Phasia helva* Wiedemann, 1818).

*Bogosia similis* Villeneuve, 1926b: 64. Syntypes, unspecified number and sex (2 males in CNC, one from each type locality, Cooper and O’Hara 1996: 19). Type localities: D.R. Congo, Katanga (Kalemie [as “Albertville”]) and Nord-Kivu (Beni).

Note: There are four specimens in NHMW that we believe belong to the original type series of *Phasia helva* Wiedemann, 1818: three males and one damaged specimen of undetermined sex (examined by JEOH). One male is from “Prom. bon. spei” [= Cape of Good Hope] and “Coll. Wiedem.” and the other three specimens are from “Cap b. sp.” [= Cape of Good Hope] and “Coll. Winthem”. All four specimens have “Type” handwritten on the collection label but only one, the male from “Coll. Wiedem.”, additionally bears a small red “Type” label. This last specimen is accepted as the lectotype of *Phasia helva* by fixation of Townsend (1938b: 15).

The distribution of *Bogosia helva* (Wiedemann, 1818) given here follows the “Material examined” in Barraclough (1985a: 363–364). Van Emden (1945: 429) additionally recorded the species from Ghana, Nigeria and Sudan, based in part on material in BMNH that was later examined by Barraclough. It is possible that Barraclough (1985a) did not accept all of the identifications of *Bogosia helva* by van Emden (1945).

***inconspicua*** (Villeneuve, 1938).—Afrotropical: D.R. Congo.

*Epineura inconspicua* Villeneuve, 1938c: 16. Lectotype male (IRSNB), by designation of Barraclough (1985a: 356). Type locality: D.R. Congo, Nord-Kivu, Rutshuru.

***rogezensis*** Barraclough, 1985.—Afrotropical: Madagascar.

*Bogosia rogezensis* Barraclough, 1985a: 359. Holotype male (MNHN). Type locality: Madagascar, Toamasina, Rogez [ca. 18°48′S 48°37′E].

***rubens*** (Villeneuve, 1923).—Afrotropical: D.R. Congo, Nigeria, South Africa, Tanzania, Uganda, Zimbabwe.

*Epineura rubens* Villeneuve, 1923: 78. Lectotype male (BMNH), by fixation of Barraclough (1985a: 352, 355) (examination of “Holotype ♂” from Ibadan in BMNH is regarded as a lectotype fixation). Type locality: Nigeria, Ibadan.

***rufiventris*** Bigot, 1876.—Afrotropical: Cameroon, Congo, D.R. Congo, Malawi, South Africa, Tanzania, Zimbabwe.

*Bogosia rufiventris* Bigot, 1876: 399. Lectotype male (BMNH), by fixation of Crosskey (1971: 296) (examination of “Holotype ♂” from Natal in BMNH is regarded as a lectotype fixation). Type locality: South Africa, KwaZulu-Natal.

##### Genus *BOGOSIELLA* Villeneuve, 1923

***BOGOSIELLA*** Villeneuve, 1923: 78. Type species: *Bogosiella pomeroyi* Villeneuve, 1923, by monotypy. **Status revived.**

Note: Sun and Marshall (2003: 19) synonymized *Bogosiella* Villeneuve, 1923 with *Phasia* Latreille, 1804 but its single species, *Bogosiella pomeroyi* Villeneuve, 1923, is inexplicably missing from their work except for a brief mention of its membership in the *Phasia varicolor* (Curran, 1927) species group (p. 214, as “*pomeryi*”). We do not agree with this synonymy and here reinstate *Bogosiella* as a genus and classify it in the Gymnosomatini as first proposed by Tschorsnig (1985: 106). The characters that distinguish *Bogosiella* will be given in the Tachinidae chapter of the *Manual of Afrotropical Diptera* (in prep.).

***pomeroyi*** Villeneuve, 1923.—Afrotropical: “widespread W. Afr. to E. Afr. & sthn Afr.” (Crosskey 1980b: 825), including Côte d’Ivoire, D.R. Congo, Ghana, Kenya, Malawi, Nigeria, Sierra Leone, South Africa, Sudan, Uganda, Zimbabwe. **Comb. revived.**

*Musca fasciata* Fabricius, 1805: 299 (junior primary homonym of *Musca fasciata* Müller, 1764 and others). Lectotype male (ZMUC), by fixation of Townsend (1931: 388) (examination of “Male Ht” from Guinea in ZMUC [as “Copenhagen Fab. Coll.”] is regarded as a lectotype fixation). Type locality: “Guinea” (referring to West Africa).

*Bogosiella pomeroyi* Villeneuve, 1923: 79. Lectotype, unspecified sex (BMNH), by fixation of Townsend (1931: 388) (examination of “Ht” from South Nigeria in BMNH is regarded as a lectotype fixation). Type locality: Nigeria, Ibadan.

*Besseria atypica* Curran, 1933: 168. Holotype female (BMNH). Type locality: Nigeria, Ibadan.

*pomeryi*. Incorrect subsequent spelling of *pomeroyi* Villeneuve, 1923 (Sun and Marshall 2003: 214).

##### Genus *GYMNOSOMA* Meigen, 1803

*RHODOGYNE* Meigen, 1800: 39. Name suppressed by ICZN (1963: 339).

***GYMNOSOMA*** Meigen, 1803: 278. Type species: *Musca rotundata* Linnaeus, 1758 (as “*Musca rotundata* Fabr.”), by monotypy [Palaearctic].

***emdeni*** (Mesnil, 1950).—Afrotropical: Ethiopia, Kenya, Tanzania, Uganda, Zimbabwe.

*Rhodogyne emdeni* Mesnil, 1950d: 114. Holotype male (CNC). Type locality: Zimbabwe, Kadoma [as “Gatooma”].

*Musca rotundata* of van Emden (1945: 434, as “*Gymnosoma rotundatum*”), not Linnaeus, 1758. Misidentification (Crosskey 1980b: 825).

***fuscohalteratum*** van Emden, 1945.—Afrotropical: Malawi, Nigeria.

*Gymnosoma fuscohalteratum* van Emden, 1945: 434. Holotype male (BMNH). Type locality: Malawi, Thyolo [as “Cholo”].

##### Genus *TRICHOPODA* Berthold, 1827

***TRICHOPODA*** Berthold, 1827: 508 (as “Trichopode” (vernacular) by Latreille, 1825: 498, name first latinized in Berthold’s German translation of Latreille (1825)). Type species: *Thereva plumipes* Fabricius, 1805, by subsequent designation of Coquillett (1910: 616).

*TRICHIOPODA*. Incorrect subsequent spelling of *Trichopoda* Berthold, 1827 (Latreille 1829: 512).

Note: Two species of *Trichopoda* Berthold, 1827 native to the New World have been introduced into South Africa as potential biological control agents against the southern green stink bug, *Nezara viridula* (Linnaeus): *Trichopoda giacomellii* (Blanchard, 1966) and *Trichopoda pennipes* (Fabricius, 1781) (van den Berg et al. 1995, van den Berg and Greenland 1996, van den Berg and Greenland 1997). The establishment of neither species has been confirmed. *Trichopoda* was first treated in the Gymnosomatini by Tschorsnig (1985: 106).

There is an unconfirmed and doubtful report of *Trichopoda* sp. from Tanzania (Ngongolo et al. 2014). We were unsuccessful in contacting the senior author for more information about this record and consider it as most likely based on a misidentification. We tentatively record *Trichopoda* from the Afrotropical Region but note that confirmed species records are lacking.

*giacomellii* (Blanchard, 1966).—Afrotropical: ?South Africa. [Neotropical.]

*Trichopodopsis giacomellii* Blanchard, 1966: 75.

*pennipes* (Fabricius, 1781).—Afrotropical: ?South Africa. [Nearctic.]

*Musca pennipes* Fabricius, 1781: 450.

#### Tribe HERMYINI

Note: Crosskey (1980b: 826, 827) treated *Hermya* Robineau-Desvoidy, 1830 and *Paraclara* Bezzi, 1908 (as *Clara* Brauer & Bergenstamm, 1889, but see note below) as genera in the tribe Cylindromyiini. The Hermyini are currently recognized as a tribe (e.g., Herting 1984: 162, O’Hara et al. 2009: 130) and *Paraclara* is here transferred to it.

##### Genus *HERMYA* Robineau-Desvoidy, 1830

***HERMYA*** Robineau-Desvoidy, 1830: 226. Type species: *Hermya afra* Robineau-Desvoidy, 1830 (= *Ocyptera diabolus* Wiedemann, 1819), by subsequent designation of Townsend (1916b: 7).

*ORECTOCERA* van der Wulp, 1881: 39. Type species: *Tachina beelzebul* Wiedemann, 1830, by subsequent designation of Townsend (1936a: 75) [Oriental].

*PARAPHANIA* Brauer & Bergenstamm, 1889: 141 [also 1890: 73]. Type species: *Ocyptera diabolus* Wiedemann, 1819, by monotypy.

*LIANCOSMIA* Speiser, 1910: 156. Type species: *Liancosmia ditissima* Speiser, 1910, by monotypy.

*DEUTEROCLARA* Villeneuve, 1915b: 207. Type species: *Deuteroclara regalis* Villeneuve, 1915, by monotypy.

*HERMYIA* Bezzi & Stein, 1907: 566. Unjustified emendation of *Hermya* Robineau-Desvoidy, 1830 (see O’Hara et al. 2011: 23 for an explanation for why this spelling in Scudder 1882: 160 is not accepted as an unjustified emendation).

***albifacies*** Curran, 1941.—Afrotropical: D.R. Congo.

*Hermya albifacies* Curran, 1941: 5 (junior secondary homonym of *Pseudorectocera albifacies* Townsend, 1928; not renamed while *Pseudorectocera albifacies* is in synonymy with *Hermya beelzebul* (Wiedemann, 1830) [Oriental]). Holotype male (AMNH). Type locality: D.R. Congo, Orientale, Kisangani [as “Stanleyville”].

***confusa*** Curran, 1941.—Afrotropical: Cameroon, D.R. Congo, Madagascar, Nigeria, Uganda.

*Hermya confusa* Curran, 1941: 4. Holotype male (AMNH). Type locality: D.R. Congo, Orientale, Kisangani [as “Stanleyville”].

***diabolus*** (Wiedemann, 1819).—Afrotropical: “widespread trop. Afr. & sthn Afr.” (Crosskey 1980b: 827), including D.R. Congo, Ghana, Guinea, Kenya, Liberia, Malawi, Sierra Leone, South Africa, Sudan, Tanzania, Uganda, Zimbabwe.

*Ocyptera diabolus* Wiedemann, 1819: 26. Syntypes, males and females (3 syntypes in ZMUC, Zimsen 1954: 22). Type locality: South Africa, Western Cape, Cape of Good Hope [as “Prom. bon. sp.” = “Promontorium Bonae Spei”].

*Hermya afra* Robineau-Desvoidy, 1830: 227. Type(s), unspecified sex (originally in Dejean’s collection, the Diptera of which are mostly lost; Evenhuis et al. 2010: 238). Type locality: South Africa [as “Brésil”, in error according to Crosskey 1980b: 827].

*Hermya hottentota* Robineau-Desvoidy, 1830: 227. Type(s), unspecified sex (MNHN or lost). Type locality: South Africa, Western Cape, Cape of Good Hope [as “Cap de Bonne-Espérance”].

*Hermya pictipennis* Curran, 1941: 5. Holotype male (AMNH). Type locality: Uganda.

***ditissima*** (Speiser, 1910).—Afrotropical: “widespread W. Afr., E. & sthn Afr.” (Crosskey 1980b: 828), including Cameroon, D.R. Congo, Ghana, Kenya, South Africa, Tanzania, Uganda.

*Liancosmia ditissima* Speiser, 1910: 157. Holotype female [not male as published, Townsend 1931: 391] (NHRS). Type locality: Tanzania, Mt. Kilimanjaro [as “Kilimandjaro”], Kibongoto [as “Kibonoto”], 1300–1900m.

***nitida*** Curran, 1941.—Afrotropical: D.R. Congo, Kenya, Uganda.

*Hermya nitida* Curran, 1941: 4. Holotype male (AMNH). Type locality: D.R. Congo, Orientale, Kisangani [as “Stanleyville”].

***regalis*** (Villeneuve, 1915).—Afrotropical: Madagascar.

*Deuteroclara regalis* Villeneuve, 1915b: 208. Lectotype male (CNC), by fixation of Townsend (1938b: 102) (mention of “Ht male” from Tananarive in Rambouillet [Villeneuve’s personal collection, since dispersed] is regarded as a lectotype fixation). Type locality: Madagascar (Antananarivo, Antananarivo [as “Tananarive”] according to label data, Cooper and O’Hara 1996: 28).

***vittata*** Curran, 1941.—Afrotropical: Cameroon, D.R. Congo.

*Hermya vittata* Curran, 1941: 4. Holotype male (AMNH). Type locality: Cameroon, Edea [as “Eden” in error, Arnaud 1963: 116].

##### Genus *PARACLARA* Bezzi, 1908

*CLARA* Brauer & Bergenstamm, 1889: 141 [also 1890: 73] (junior homonym of *Clara* Gill, 1862). Type species: *Clara dimidiata* Brauer & Bergenstamm, 1889, by monotypy.

***PARACLARA*** Bezzi, 1908b: 86. Type species: *Paraclara magnifica* Bezzi, 1908, by monotypy.

Note: The valid name for this genus was given as *Clara* Brauer & Bergenstamm, 1889 by Crosskey (1980b: 826) but was corrected to *Paraclara* Bezzi, 1908 in the simultaneously published Appendix (Crosskey 1980a: 1230). *Paraclara* was treated in the Cylindromyiini by Crosskey (1980b: 826) but is moved here to the Hermyini, **comb. n.**

***dimidiata*** (Brauer & Bergenstamm, 1889).—Afrotropical: “widespread W. Afr. to Sudan & sthn Afr.” (Crosskey 1980b: 826), including D.R. Congo, Ghana, Malawi, Nigeria, Sierra Leone, South Africa.

*Clara dimidiata* Brauer & Bergenstamm, 1889: 141, 170 [also 1890: 73, 102]. Lectotype male (NHMW), by fixation of Townsend (1931: 390) (examination of “Male Ht” from Cape of Good Hope in NHMW is regarded as a lectotype fixation for the single male syntype in NHMW [examined by JEOH]). Type locality: “Patria?” (i.e., unknown; South Africa, Western Cape, Cape of Good Hope according to label data of male lectotype and female paralectotype in NHMW, as “Cap. b. sp.” [= “Cap Bonae Spei”]).

***magnifica*** Bezzi, 1908.—Afrotropical: “widespread W. Afr. to E. Afr.” (Crosskey 1980b: 826), including D.R. Congo, Eritrea, Kenya, Nigeria, South Africa, Sudan, Tanzania, Uganda, Yemen.

*Paraclara magnifica* Bezzi, 1908b: 86. Lectotype male (MSNM, Arnaud 1982: 12), by fixation of Townsend (1938b: 149) (mention of “Ht male” from Adi Ugri in MSNM is regarded as a lectotype fixation). Type locality: Eritrea, near Mendefera [as “Adi Ugri”, ca. 14°53′N 38°49′E].

#### Tribe IMITOMYIINI

##### Genus *IMITOMYIA* Townsend, 1912

*HIMANTOSTOMA* Loew, 1863b: 320, 321 (junior homonym of *Himantostoma* Agassiz, 1862). Type species: *Himantostoma sugens* Loew, 1863, by monotypy [Nearctic].

***IMITOMYIA*** Townsend, 1912: 49 (*nomen novum* for *Himantostoma* Loew, 1863).

*DIPLOPOTA* Bezzi, 1918: 272. Type species: *Himantostoma mochii* Bezzi, 1917, by original designation.

***kivuensis*** Verbeke, 1962.—Afrotropical: D.R. Congo.

*Imitomyia kivuensis* Verbeke, 1962a: 150. Holotype male (MRAC). Type locality: D.R. Congo, Nord-Kivu, Kibati [ca. 1°36′S 29°16′E].

***mochii*** (Bezzi, 1917).—Afrotropical: D.R. Congo, Eritrea, Kenya (**new record**, MZUR [PC]), South Africa, Tanzania, Uganda, Zimbabwe.

*Himantostoma mochii* Bezzi, 1917: 91. Syntypes, males and females (MSNM, Arnaud 1982: 12). Type locality: Eritrea, Ghinda [ca. 15°27′N 39°6′E].

***nitida*** (van Emden, 1945).—Afrotropical: D.R. Congo, Gambia, Ghana, Kenya, Nigeria, Tanzania, Uganda.

*Diplopota nitida* van Emden, 1945: 412. Holotype male (BMNH). Type locality: Tanzania, Kilosa [as “Kilossa”].

#### Tribe LEUCOSTOMATINI

Note: Crosskey (1980b: 828, 1984: 195) recognized the tribe Cinochirini but the members of this tribe have since been incorporated into the Leucostomatini (e.g., Herting 1984, Herting and Dely-Draskovits 1993, Cerretti 2010). The family-group name Leucostomatini has priority over Cinochirini (Sabrosky 1999).

##### Genus *APOMORPHOMYIA* Crosskey, 1984

***APOMORPHOMYIA*** Crosskey, 1984: 298. Type species: *Apomorphomyia lygaeidophaga* Crosskey, 1984, by original designation.

***lygaeidophaga*** Crosskey, 1984.—Afrotropical: South Africa.

*Apomorphomyia lygaeidophaga* Crosskey, 1984: 299. Holotype male (BMNH). Type locality: South Africa, Gauteng, Johannesburg, Frankenwald.

##### Genus *CAHENIA* Verbeke, 1960

***CAHENIA*** Verbeke, 1960: 340. Type species: *Cahenia mima* Verbeke, 1960, by original designation.

*MAPOLOMYIA* Verbeke, 1960: 343. Type species: *Mapolomyia connexa* Verbeke, 1960, by original designation.

Note: *Mapolomyia* Verbeke, 1960 was treated as a genus by Crosskey (1980b: 828) but was later synonymized with *Cahenia* Verbeke, 1960 by Crosskey (1984: 200). The relative priority of *Cahenia* Verbeke, 1960 and *Mapolomyia* Verbeke, 1960, when the two are treated as synonyms, was established by Crosskey (1984: 200), as the First Reviser (Article 24.2.2 of the *Code*, ICZN 1999).

***connexa*** (Verbeke, 1960).—Afrotropical: D.R. Congo.

*Mapolomyia connexa* Verbeke, 1960: 343. Holotype male (MRAC). Type locality: D.R. Congo, Orientale, Mapolo.

***mima*** Verbeke, 1960.—Afrotropical: D.R. Congo.

*Cahenia mima* Verbeke, 1960: 340. Holotype male (MRAC). Type locality: D.R. Congo, Orientale, Mapolo.

##### Genus *CALYPTROMYIA* Villeneuve, 1915

***CALYPTROMYIA*** Villeneuve, 1915a: 92. Type species: *Calyptromyia barbata* Villeneuve, 1915, by original designation [Oriental].

*CALYPTEROMYIA*. Incorrect subsequent spelling of *Calyptromyia* Villeneuve, 1915 (Hennig 1941: 189).

***stupenda*** Dear, 1981.—Afrotropical: Madagascar.

*Calyptromyia stupenda* Dear, 1981: 504. Holotype male (BMNH). Type locality: Madagascar, Toliara [as “Tulear”], Forêt de Zombitsy, 300m.

##### Genus *CLAIRVILLIOPS* Mesnil, 1959

***CLAIRVILLIOPS*** Mesnil, 1959: 29 (as subgenus of *Dionaea* Robineau-Desvoidy, 1830). Type species: Dionaea (Clairvilliops) inermis Mesnil, 1959 (= *Clairvillia breviforceps* van Emden, 1954), by monotypy.

Note: *Clairvilliops* Mesnil, 1959 was treated as a synonym of *Dionaea* Robineau-Desvoidy, 1830 by Crosskey (1980b: 828) but was moved into synonymy with *Clairvillia* Robineau-Desvoidy, 1830 by Crosskey (1984: 200). *Clairvilliops* was recognized as a genus by Herting (1984: 176) and subsequent authors (e.g., Herting and Dely-Draskovits 1993: 424, Tschorsnig and Richter 1998: 781, Shima 2014: 865).

***breviforceps*** (van Emden, 1954).—Afrotropical: D.R. Congo, Tanzania. Palaearctic: Japan. Oriental: Malaysia, Taiwan.

*Clairvillia breviforceps* van Emden, 1954: 549. Holotype female (MRAC). Type locality: D.R. Congo, Nord-Kivu, Rutshuru.

Dionaea (Clairvilliops) inermis Mesnil, 1959: 29. Holotype female (SMNS). Type locality: Tanzania, Pare Mountains, Usangi.

Note: *Dionaea inermis* Mesnil, 1959 was synonymized with *Clairvillia breviforceps* van Emden, 1954 by Crosskey (1984: 200, 236). This synonymy was followed by Herting (1984: 177, as pers. comm. from Crosskey).

##### Genus *DIONOMELIA* Kugler, 1978

***DIONOMELIA*** Kugler, 1978b: 346. Type species: *Dionomelia hennigi* Kugler, 1978, by original designation.

***hennigi*** Kugler, 1978.—Afrotropical: U.A. Emirates. Palaearctic: Europe (SW. Eur.), M. East (all).

*Dionomelia hennigi* Kugler, 1978b: 346. Holotype female (TAU). Type locality: Israel, Dead Sea area, ‘En Boqeq [ca. 31°12′N 35°22′E].

##### Genus *LEUCOSTOMA* Meigen, 1803

***LEUCOSTOMA*** Meigen, 1803: 279. Type species: *Ocyptera simplex* Fallén, 1815, by subsequent monotypy of Meigen (1824: 234).

***africanum*** Villeneuve, 1920.—Afrotropical: South Africa.

*Leucostoma africanum* Villeneuve, 1920a: 155. Syntypes, males and females (1 male and 1 female in CNC). Type locality: South Africa, Eastern Cape, Willowmore.

***engeddense*** Kugler, 1966.—Afrotropical: South Africa, U.A. Emirates. Palaearctic: Europe (SW. Eur., SC. Eur., SE. Eur., Turkey), M. East (all), N. Africa (Canary Is., Madeira, NW. Africa).

*Leucostoma engeddense* Kugler, 1966: 177. Holotype female (TAU). Type locality: Israel, ‘En Gedi [as “En-Geddi”, ca. 31°27′N 35°23′E].

***obsidianum*** (Wiedemann, 1830).—Afrotropical: Sudan, Yemen. Palaearctic: Europe (SC. Eur.), M. East (all), N. Africa (Canary Is.).

*Tachina obsidiana* Wiedemann, 1830: 341. Lectotype female (SMF), by fixation of Herting (1982: 12) (examination of “Type (♀)” from Nubien in SMF is regarded as a lectotype fixation). Type locality: Nubia region [as “Nubien”, a region in southern Egypt and northern Sudan, recorded here as Sudan following Crosskey 1980b: 829].

*Leucostoma marismortui* Kugler, 1966: 179 (as “*maris-mortui*”). Holotype female (TAU). Type locality: Israel, ‘En Gedi [as “En-Geddi”, ca. 31°27′N 35°23′E].

Note: Crosskey (1980b: 829) synonymized *Tachina obsidiana* Wiedemann, 1830 with *Ocyptera simplex* Fallén, 1815. Herting (1982: 12) examined the type of the former and recognized *Tachina obsidiana* as a distinct species of *Leucostoma* Meigen. Subsequent authors have followed Herting (1982); e.g., Báez et al. (1986: 14), Herting and Dely-Draskovits (1993: 420), Tschorsnig and Báez (2002: 229), Zeegers (2007: 404), and Cerretti and Freidberg (2009: 13).

***simplex*** (Fallén, 1815).—Afrotropical: Cape Verde, Sierra Leone. Palaearctic: C. Asia, Europe (all except Turkey), Kazakhstan, Mongolia, Russia (W. Russia, W. Siberia, E. Siberia, S. Far East), Transcaucasia. Australasian: Australia, Hawaii. Nearctic: widespread. Neotropical: possibly widespread.

*Ocyptera simplex* Fallén, 1815: 240. Holotype female [not syntypes of both sexes as cited by Herting 1984: 174] (NHRS). Type locality: Sweden, Småland, Kalmar Län.

*Tachina analis* of van Emden (1945: 394, as “*Leucostoma anale*”), not Meigen, 1824. Misidentification (Crosskey 1980b: 829).

*tetraptera* (Meigen, 1824).—Afrotropical: ?Botswana, ?Nigeria, ?South Africa. [Palaearctic.]

*Tachina tetraptera* Meigen, 1824: 290.

Note: Crosskey’s (1980b: 829) records of *Leucostoma tetraptera* (Meigen, 1824) from Botswana, Nigeria and South Africa were likely based on misidentifications. Crosskey (1980b) considered van Emden’s (1945: 394) records of *Leucostoma africanum* Villeneuve, 1920 from South Africa to be misidentifications of *Leucostoma tetraptera*.

#### Tribe PHASIINI

##### Genus *PHASIA* Latreille, 1804

***PHASIA*** Latreille, 1804: 195. Type species: *Conops subcoleoptratus* Linnaeus, 1767, by subsequent monotypy of Latreille (1805: 379); see rulings by ICZN (1970, 2006) [Palaearctic].

*ALOPHORA* Robineau-Desvoidy, 1830: 293. Type species: *Syrphus hemipterus* Fabricius, 1794, by subsequent designation of Robineau-Desvoidy (1863b: 226, as “*Thereva hemiptera* de Fabricius”) [Palaearctic].

*HYALOMYA* Robineau-Desvoidy, 1830: 298. Type species: *Phasia semicinerea* Meigen, 1824 (= *Phasia pusilla* Meigen, 1824), by subsequent designation of Westwood (1840: 140) [Palaearctic].

*HYALOMYIA* Macquart, 1834: 69 [also 1834: 205]. Unjustified emendation of *Hyalomya* Robineau-Desvoidy, 1830 (see Evenhuis et al. 2010: 90).

*HALOPHORA* Agassiz, 1846b: 171. Unjustified emendation of *Alophora* Robineau-Desvoidy, 1830 (see Evenhuis et al. 2010: 36).

*PARALOPHORA* Girschner, 1887: 412 (as subgenus of *Alophora* Robineau-Desvoidy, 1830). Type species: *Phasia pusilla* Meigen, 1824, by monotypy [Palaearctic].

*MORMONOMYIA* Brauer & Bergenstamm, 1891: 388 [also 1891: 84]. Type species: *Mormonomyia laniventris* Brauer & Bergenstamm, 1891 (= *Phasia argentifrons* Walker, 1849), by subsequent designation of Sharp (1893: 301, as “*laniventris*, Wd., ?n. sp.”).

*ALLOPHORA* Mik, 1894: 49. Unjustified emendation of *Alophora* Robineau-Desvoidy, 1830 (see Evenhuis et al. 2010: 36).

*ALOPHORELLA* Townsend, 1912: 45. Type species: *Thereva obesa* Fabricius, 1798, by original designation [Palaearctic].

*PARALLOPHORA*. Incorrect subsequent spelling of *Paralophora* Girschner, 1887 (e.g., Bezzi and Stein 1907: 583, Bezzi 1908b: 88, Mesnil 1953b: 176).

Note: The Afrotropical species of *Phasia* Latreille, 1804 were treated in a world revision of the genus by Sun and Marshall (2003). Subgenera were not recognized by Sun and Marshall (2003) and are not recognized here because the subgeneric placements of the Afrotropical species require more study.

***africana*** Sun, 2003.—Afrotropical: South Africa.

*Phasia africana* Sun *in* Sun & Marshall, 2003: 159. Holotype female (USNM). Type locality: South Africa, Eastern Cape, Willowmore.

***argentifrons*** Walker, 1849.—Afrotropical: Botswana, Ethiopia, Kenya, Madagascar, Malawi, Tanzania, South Africa, Uganda, Zimbabwe.

*Phasia argentifrons* Walker, 1849: 691. Lectotype male (BMNH), by fixation of Sun and Marshall (2003: 26) (examination of “Holotype ♂” from South Africa in BMNH is regarded as a lectotype fixation). Type locality: South Africa [as “Interior of South Africa”].

*Mormonomyia laniventris* Brauer & Bergenstamm, 1891: 388 [also 1891: 84] (as “*laniventris* Wd. litt. n.”). Lectotype male (NHMW, not located by JEOH), by fixation of Townsend (1938b: 58) (mention of “Ht male” from Cape of Good Hope in NHMW is regarded as a lectotype fixation). Type locality: South Africa, Western Cape, Cape of Good Hope [as “Cap b. sp.”].

Allophora (Phorantha) bathymyza Speiser, 1910: 158. Holotype female [not male as published, van Emden 1945: 433] (NHRS). Type locality: Tanzania, Mt. Meru, lowlands.

*Mormonomyia umbrosa* Villeneuve, 1935b: 252. Holotype male (CNC). Type locality: South Africa, Gauteng, Pretoria.

*Mormonomyia brunnicosa* Villeneuve, 1935b: 252. Holotype male (CNC). Type locality: South Africa, Eastern Cape, Port Elizabeth.

*Hyalomya munroi* Curran, 1936: 10. Holotype male (SANC). Type locality: South Africa, Western Cape, Muizenberg [suburb of Cape Town].

*Hyalomya victoria* Curran, 1936: 11. Holotype male (AMNH). Type locality: Zimbabwe, “Victoria” (probably Victoria Falls).

***cana*** Sun, 2003.—Afrotropical: D.R. Congo, South Africa, Tanzania, Zimbabwe.

*Phasia cana* Sun *in* Sun & Marshall, 2003: 164. Holotype female (BMNH). Type locality: South Africa, “Transvaal, 8km NE Lake Trkhardt ?” (probably near Trichardt in Mpumalanga [ca. 26°29′S 29°14′E] or near Louis Trichardt in Limpopo [ca. 23°3′S 29°55′E]).

***clavigralla*** Sun, 2003.—Afrotropical: Tanzania.

*Phasia clavigralla* Sun *in* Sun & Marshall, 2003: 169. Holotype female (BMNH). Type locality: Tanzania, “Kilosa District, Ilouga ARI” [not located].

***distincta*** Sun, 2003.—Afrotropical: South Africa.

*Phasia distincta* Sun *in* Sun & Marshall, 2003: 30. Holotype male (NMDA). Type locality: South Africa,”Transvaal” (a former province that occupied much of the northeastern part of the country and has since been subdivided into several provinces).

***jeanneli*** (Mesnil, 1953).—Afrotropical: Kenya, South Africa.

*Parallophora jeanneli* Mesnil, 1953b: 177. Holotype female (MNHN). Type locality: Kenya, east side of Mt. Elgon, Elgon Saw Mill, 2470m.

***mathisi*** Sun, 2003.—Afrotropical: Kenya, Seychelles.

*Phasia mathisi* Sun *in* Sun & Marshall, 2003: 196. Holotype female (USNM). Type locality: Seychelles, Aldabra Island Group, Picard (an abandoned settlement on West Is.).

***mesnili*** (Draber-Mońko, 1965).—Afrotropical: Yemen. Palaearctic: C. Asia, Europe (W. Eur., E. Eur., SW. Eur., SC. Eur., SE. Eur., Turkey), Kazakhstan, M. East (all), N. Africa (Canary Is., NW. Africa), Pal. China, Russia (W. Russia, W. Siberia, S. Far East), Transcaucasia.

Alophora (Hyalomyia) mesnili Draber-Mońko, 1965: 109. Holotype female (ZMUM). Type locality: Russia, Stalingradskaja Oblast’, Tinguta.

Alophora (Hyalomyia) theodori Draber-Mońko, 1965: 114 (named for *aethiopica* of Mesnil, 1953, not Bezzi, 1908, but misidentified; see note). Holotype female (HUJI). Type locality: Israel, ‘En Gedi [as “Eingedi”, ca. 31°27′N 35°23′E].

Note: Draber-Mońko (1965: 114) described *Alophora theodori* for *Allophora aethiopica* of Mesnil (1953b: 177, as *Parallophora aethiopica*), not Bezzi, 1908. However, Draber-Mońko misidentified *Alophora aethiopica* of Mesnil, which is currently interpreted as *Phasia venturii* (Draber-Mońko, 1965) (Sun and Marshall 2003: 155). The relative priority of *Alophora mesnili* Draber-Mońko, 1965 and *Alophora theodori* Draber-Mońko, 1965, when the two are treated as synonyms, was established by Ziegler (1994: 159), as the First Reviser (Article 24.2.2 of the *Code*, ICZN 1999).

***multisetosa*** (Villeneuve, 1923).—Afrotropical: Nigeria, Tanzania, Zimbabwe.

*Allophora multisetosa* Villeneuve, 1923: 81. Lectotype female (BMNH), by fixation of van Emden (1945: 432) (mention of “type” from Ibadan in BMNH is regarded as a lectotype fixation). Type locality: Nigeria, Ibadan.

***nasuta*** (Loew, 1852).—Afrotropical: Burundi, D.R. Congo (**new record**, IRSNB [PC]), Eritrea, Kenya, Lesotho, Mozambique, South Africa, Zimbabwe. Palaearctic: “N. Afr.” (Crosskey 1980b: 824, no published records found).

*Hyalomyia nasuta* Loew, 1852: 660 [also 1862: 26, full description]. Type(s), unspecified sex (1 female in ZMHB, examined by JEOH). Type locality: Mozambique (Inhambane according to Loew 1862: 26 and label data).

*Alophora capensis* Schiner, 1868: 337. Holotype male (NHMW). Type locality: South Africa, Western Cape, Cape of Good Hope [as “Cap”].

Allophora (Parallophora) aethiopica Bezzi, 1908b: 88. Holotype male (not located, not among the labelled types of Bezzi in MSNM examined by Arnaud 1982). Type locality: Eritrea, Sabarguma [ca. 15°31′N 39°6′E].

*Mormonomyia leucodes* Villeneuve, 1935b: 252. Holotype male (CNC). Type locality: South Africa (no additional locality data in description; holotype without locality data, Cooper and O’Hara 1996: 52).

***nasalis*** (Bezzi, 1908).—Afrotropical: D.R. Congo, Kenya, Nigeria, Rwanda (**new record**, IRSNB [PC]), South Africa, Tanzania, Zambia, Zimbabwe.

Allophora (Hyalomyia) nasalis Bezzi, 1908c: 384. Holotype female (?IRSNB). Type locality: D.R. Congo, Bas-Congo, Boma.

*Allophora nigeriensis* Villeneuve, 1923: 80. Lectotypev male (BMNH), by fixation of van Emden (1945: 432) (mention of “type” from Ibadan in BMNH is regarded as a lectotype fixation). Type locality: Nigeria, Ibadan.

*Hyalomya cuthbertsoni* Curran, 1936: 8. Holotype male (AMNH). Type locality: Zimbabwe, Kadoma [as “Gatooma”].

Note: Van Emden (1945: 432) referred to the “type” of *Allophora nigeriensis* Villeneuve, 1923 but did not give its sex. This specimen is presumed to be the male syntype examined by Sun and Marshall (2003: 180). Hence, the lectotype recognized here, which is the “type” of van Emden (1945) and the “syntype” of Sun and Marshall (2003), is a male.

***nigrofimbriata*** (Villeneuve, 1935).—Afrotropical: Botswana, D.R. Congo, Kenya, Malawi, Nigeria, South Africa, Tanzania, Uganda, Zimbabwe.

*Mormonomyia nigrofimbriata* Villeneuve, 1935b: 252. Holotype male (CNC). Type locality: South Africa, “Transvaal” ([North West], Klerksdorp according to label data, Cooper and O’Hara 1996: 52 [handwritten locality misinterpreted as “Kluksdorp”]).

*Mormonomyia claripennis* Villeneuve, 1935b: 253. Lectotype male (CNC), by fixation of Sun and Marshall (2003: 43) (examination of “Holotype ♂” from Nakuta in CNC is regarded as a lectotype fixation). Type locality: not given (Kenya, Nakuta according to Cooper and O’Hara 1996: 52).

*Mormonomyia fumosa* Villeneuve, 1935b: 253. Type(s), unspecified sex (1 male in CNC). Type locality: not given (CNC syntype from Zimbabwe, Bulawayo, Cooper and O’Hara 1996: 52).

*Hyalomya negator* Curran, 1936: 11. Holotype male (AMNH). Type locality: Zimbabwe, Matetsi [ca. 18°15′S 26°1′E].

Note: The relative priority of *Mormonomyia nigrofimbriata* Villeneuve, 1935, *Mormonomyia claripennis* Villeneuve, 1935 and *Mormonomyia fumosa* Villeneuve, 1935, when the three are treated as synonyms, was established by van Emden (1945: 433), as the First Reviser (Article 24.2.2 of the *Code*, ICZN 1999).

***nigromaculata*** Sun, 2003.—Afrotropical: South Africa.

*Phasia nigromaculata* Sun *in* Sun & Marshall, 2003: 44. Holotype female (NMDA). Type locality: South Africa, Western Cape, Ceres District, north of Gydo Pass, Clanwillam Road.

***subnitida*** Sun, 2003.—Afrotropical: South Africa.

*Phasia subnitida* Sun *in* Sun & Marshall, 2003: 188. Holotype male (AMNH). Type locality: South Africa, Mpumalanga, Kaapmuiden (25°33′S 31°20′E).

***transvaalensis*** Sun, 2003.—Afrotropical: South Africa.

*Phasia transvaalensis* Sun *in* Sun & Marshall, 2003: 111. Holotype male (BMNH). Type locality: South Africa, Gauteng, Johannesburg.

#### Tribe STRONGYGASTRINI

Note: The tribe Strongygastrini is newly recorded from the Afrotropical Region and *Rondaniooestrus* Villeneuve, 1916 is transferred here from the Rondaniooestrini. The family-group name Strongygastrini has priority over Rondaniooestrini (Sabrosky 1999).

##### Genus *RONDANIOOESTRUS* Villeneuve, 1916

***RONDANIOOESTRUS*** Villeneuve, 1916b: 465. Type species: *Rondaniooestrus apivorus* Villeneuve, 1916, by monotypy.

*RONDANIOESTRUS*. Incorrect subsequent spelling of *Rondaniooestrus* Villeneuve, 1916 (van Emden 1945: 411, etc.).

***apivorus*** Villeneuve, 1916.—Afrotropical: Kenya, South Africa, Tanzania (**new record**, NHMW [JEOH]), Uganda.

*Rondaniooestrus apivorus* Villeneuve, 1916b: 467. Holotype male (CNC). Type locality: South Africa, Eastern Cape, Port Elizabeth.

##### Unplaced species of Phasiinae

***marginata*** Macquart, 1851.—Afrotropical: Senegal.

*Elomyia marginata* Macquart, 1851b: 188 [also 1851b: 215]. Type(s), male (“presumed lost”, Crosskey 1971: 267). Type locality: Senegal.

### Subfamily TACHININAE (Fig. 6)

**Figure 6.**
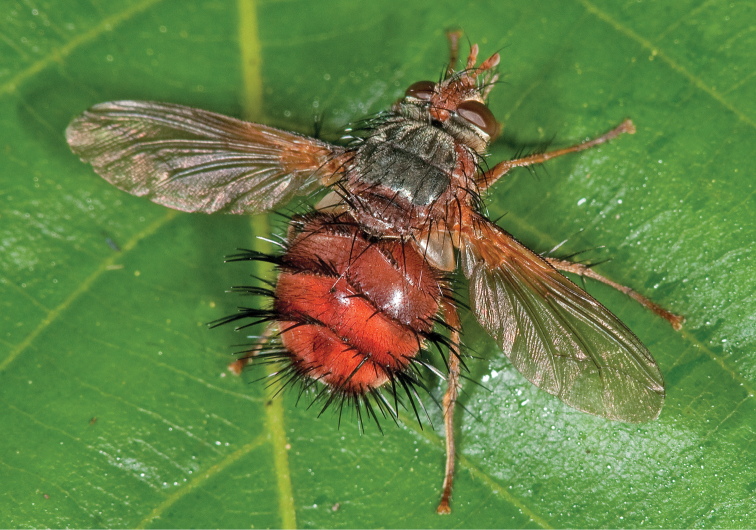
Live specimen of *Dejeania* sp. (Tachinini, Tachininae) from Mufindi, Tanzania (image courtesy of S.A. Marshall).

#### Tribe BIGONICHETINI

##### Genus *TRICHACTIA* Stein, 1924

*TRICHAETA* Becker, 1908: l18 (junior homonym of *Trichaeta* Swinhoe, 1892). Type species: *Trichaeta nubilinervis* Becker, 1908, by monotypy [Palaearctic].

***TRICHACTIA*** Stein, 1924: 138. Type species: *Thryptocera securicornis* Egger, 1865 (as “*Trichactia securricornis*”) (= *Tachina pictiventris* Zetterstedt, 1855), by monotypy [Palaearctic].

Undescribed sp. 1: South Africa (Crosskey 1980b: 840, Crosskey 1984: 248), Tanzania (TAU, examined by PC).

Undescribed sp. 2: South Africa (NMB, examined by PC).

Undescribed sp. 3: Ethiopia (TAU, examined by PC).

#### Tribe ERNESTIINI

##### Genus *BRACHELIA* Robineau-Desvoidy, 1830

***BRACHELIA*** Robineau-Desvoidy, 1830: 61. Type species: *Brachelia westermanni* Robineau-Desvoidy, 1830, by monotypy.

*PSEUDOLOEWIA* Brauer & Bergenstamm, 1889: 136 [also 1890: 68] (as “*Pseudolöwia*”). Type species: *Loewia sycophanta* Schiner, 1868 (= *Brachelia westermanni* Robineau-Desvoidy, 1830), by monotypy.

***leocrates*** (Walker, 1849).—Afrotropical: South Africa.

*Tachina leocrates* Walker, 1849: 745. Lectotype male (BMNH), by fixation of van Emden (1960: 404) (examination of male “type” from South Africa in BMNH is regarded as a lectotype fixation). Type locality: South Africa.

*Olivieria experrecta* Brauer & Bergenstamm, 1891: 407, 428 [also 1891: 103, 124] (as “*experrecta* Wd. litt. *Olivieria* Cap. [Cape of Good Hope]”). *Nomen nudum*.

*Eriothrix experrectus* Villeneuve, 1916c: 500 (as “*experrectus*, B. B. in Litt.”). Syntypes, 4 males (2 males in NHMW). Type locality: South Africa, Western Cape, Cape of Good Hope.

***minor*** Mesnil, 1968.—Afrotropical: South Africa.

*Brachelia minor* Mesnil, 1968b: 11. Holotype male (SMNS). Type locality: South Africa, Western Cape, Cape Town.

***westermanni*** Robineau-Desvoidy, 1830.—Afrotropical: South Africa.

*Brachelia westermanni* Robineau-Desvoidy, 1830: 62. Neotype male (ZMUC), by designation of Rognes et al. (2015: 476). Type locality: South Africa, Western Cape, Cape of Good Hope [as “cap de Bonne-Espérance”].

*Loewia sycophanta* Schiner, 1868: 323. Holotype male (NHMW). Type locality: South Africa, Western Cape, Cape of Good Hope [as “Cap”].

Note: *Brachelia westermanni* Robineau-Desvoidy, 1830 was treated as a junior synonym of *Tachina westermanni* Wiedemann, 1819 by van Emden (1960: 403) and Crosskey (1980b: 846). The original syntypic series of *Tachina westermanni* was mixed and comprised a species each of Tachinidae and Calliphoridae. Van Emden chose a tachinid syntype as lectotype, but Rognes et al. (2015) accepted an earlier lectotype fixation of a calliphorid syntype as valid. The valid name of the tachinid is therefore *Brachelia westermanni* Robineau-Desvoidy.

##### Genus *BRACHELIOPSIS* van Emden, 1960

***BRACHELIOPSIS*** van Emden, 1960: 405. Type species: *Bracheliopsis geniseta* van Emden, 1960, by original designation.

***geniseta*** van Emden, 1960.—Afrotropical: Kenya.

*Bracheliopsis geniseta* van Emden, 1960: 405. Holotype male (BMNH). Type locality: Kenya, Nairobi, Scott Agricultural Laboratories.

Undescribed sp.: South Africa (NMB, examined by PC).

##### Genus *GYMNOGLOSSA* Mik, 1898

***GYMNOGLOSSA*** Mik, 1898: 211. Type species: *Gymnoglossa transsylvanica* Mik, 1898, by monotypy [Palaearctic].

***munroi*** Curran, 1934.—Afrotropical: South Africa.

*Gymnoglossa munroi* Curran, 1934b: 25. Holotype male (SANC). Type locality: South Africa, Gauteng, Pretoria.

##### Genus *LINNAEMYA* Robineau-Desvoidy, 1830

***LINNAEMYA*** Robineau-Desvoidy, 1830: 52. Type species: *Linnaemya silvestris* Robineau-Desvoidy, 1830 (= *Tachina vulpina* Fallén, 1810), by subsequent designation of Robineau-Desvoidy (1863a: 131) (as *vulpina*, with *silvestris* in synonymy) [Palaearctic].

*MICROPALPIS* Macquart, 1834: 180 [also 1834: 316]. Type species: *Tachina vulpina* Fallén, 1810, by subsequent designation of d’Orbigny (1846: 200, as “*Micropalpus*”) (see Evenhuis and Thompson 1990: 237, as “*Micropalpus*”) [Palaearctic].

*LINNEMYIA* Macquart, 1835: 81. Unjustified emendation of *Linnaemya* Robineau-Desvoidy, 1830 (see Evenhuis et al. 2010: 100).

*ELACHIPALPUS* Rondani, 1850: 169. Type species: *Micropalpus longirostris* Macquart, 1845 (junior primary homonym of *Micropalpus longirostris* Macquart, 1844; = *Elachipalpus rondanii* Townsend, 1916, a probable junior synonym of *Micropalpus longirostris* Macquart, 1844), by original designation.

*TACHINOMIMA* Brauer & Bergenstamm, 1891: 383 [also 1891: 79]. Type species: *Tachinomima expetens* Brauer & Bergenstamm, 1891 (= *Micropalpus longirostris* Macquart, 1844), by monotypy [Palaearctic].

*LINNAEMYIA* Aldrich, 1905: 451. Unjustified emendation of *Linnaemya* Robineau-Desvoidy, 1830 (see Evenhuis et al. 2010: 100).

*HECATOEPALPUS* Townsend, 1933: 467. Type species: *Micropalpus prohecate* Speiser, 1910, by original designation.

*MICROPALPINUS* Enderlein, 1937: 441. Type species: *Micropalpus pallidus* Jaennicke, 1867, by original designation.

*GYMMANTIA* Enderlein, 1937: 441. Type species: *Micropalpus alboscutellatus* Speiser, 1910, by original designation.

*GYMNANTIA*. Incorrect subsequent spelling of *Gymmantia* Enderlein, 1937 (original usage not found but spelling listed by Crosskey 1980b: 846).

*LINNEMYA*. Incorrect subsequent spelling of *Linnaemya* Robineau-Desvoidy, 1830 (Robineau-Desvoidy 1863a: 130).

*MICROPALPUS*. Incorrect subsequent spelling of *Micropalpis* Macquart, 1834 (Macquart 1835: 80).

*TACHINOMINA*. Incorrect subsequent spelling of *Tachinomima* Brauer & Bergenstamm, 1891 (Villeneuve 1935a: 140).

Note: Subgenera of *Linnaemya* Robineau-Desvoidy, 1830 are not recognized here because the subgeneric placements of the Afrotropical species require more study.

***aculeata*** Curran, 1934.—Afrotropical: Burundi (**new record**, IRSNB [PC]), D.R. Congo, Ethiopia, Kenya, Malawi, Rwanda (**new record**, IRSNB [PC]), Sudan, Uganda.

*Linnaemya aculeatus* Curran, 1934b: 14 (as “*aculeatus* Villeneuve”). Holotype female (NHMW). Type locality: D.R. Congo, northwest of Lake Tanganyika [as “N.W. Tanganika”] (not Tanzania as cited by Crosskey 1980b: 846, see note).

Note: Curran (1934b: 14) attributed *Linnaemya aculeatus* to Villeneuve, but he himself was the first to describe the species. Curran based his description on “a specimen in the Vienna Museum, from northern Tanganyika, labelled as type of *Tachinomima aculeatus*”. Nearly 30 years later, van Emden (1960: 463) wrote, “I have seen 2♀ collected by Grauer in N.W. Tanganyika and belonging to the Vienna Museum. One of these was labelled by Villeneuve ‘*Micropalpus aculeatus* Villen. Typ.’”. JEOH found five specimens standing under the name *Linnaemya aculeatus* in NHMW, all from “N.W. Tanganika” and collected by Grauer (three collected in 1910, two without date but with additional locality information). Two of the females (both collected in 1910) bear blue Villeneuve determination labels and the inscription “*Micropalpus aculeatus* Typ. Villen.”. It is possible that one of these “Typ.” specimens was kept separate from the other until recent times and was not seen by either Curran or van Emden. Another possibility is that the specimen examined by Curran (the holotype of *Linnaemya aculeatus*) is neither of these “Typ.” specimens and is elsewhere in the collection or lost. This is considered possible because Curran referred to a specimen labelled as *Tachinomima aculeatus* (not *Micropalpus aculeatus*) and his description does not match exactly the two specimens labelled as “Typ.”; in particular, Curran wrote “Legs reddish, the tarsi black”, in contrast to the entirely yellow legs (including tarsi) of the “Typ.” specimens. The type locality of “N.W. Tanganika” is interpreted here as northwest of Lake Tanganyika in D.R. Congo; see note under *Zelindopsis villeneuvei* Verbeke, 1962.

***agilis*** Curran, 1934.—Afrotropical: Benin, D.R. Congo, Kenya, Malawi, Nigeria, South Africa, Tanzania, Uganda, Zimbabwe.

*Linnaemya agilis* Curran, 1934b: 8. Holotype male (BMNH). Type locality: Tanzania, Morogoro [as “Monogoro”].

Linnaemyia (Micropalpus) obscurior Villeneuve, 1934c: 409. Syntypes, males and females (1 male in CNC). Type locality: South Africa, KwaZulu-Natal, Durban.

Note: Villeneuve (1934c: 409) recorded *Linnaemyia obscurior* from several localities but restricted the type locality to Durban with the statement: “Durban (Natal): types ♂ et ♀”. Van Emden (1960: 424) erred in citing the “Type ♀” of *Micropalpus obscurior* from Kenya in BMNH, not only because the type locality was restricted to Durban by Villeneuve but also because Kenya was not mentioned in the original description.

***albifrons*** (Smith, 1870).—Afrotropical: “widespread W. Afr. to E. Afr., n.-e. Afr. & sthn Afr.” (Crosskey 1980b: 846), including Angola, Cameroon, D.R. Congo, Ethiopia, Ghana, Kenya, Malawi, Nigeria, Sierra Leone, Sudan, Tanzania, Uganda, Zimbabwe.

*Tachina albifrons* Smith *in* Dunning, 1870: 532. Lectotype, unspecified sex (BMNH), by fixation of van Emden (1960: 447) (mention of “type” from Kinsembo in BMNH is regarded as a lectotype fixation). Type locality: Angola, north of Ambriz, Kinsembo [also as Quizembo].

*Micropalpus affinis* Corti, 1895: 137. Type(s), male (?MCSN). Type locality: Ethiopia, Jubba River, “Arussi Galla, Ganale Guddà” [most likely a valley of the upper Ganale River, a tributary of the Jubba River on the eastern edge of the Arussi and Bale Mountains, ca. 7°0′N 40°30′E].

*Micropalpus alopecinus pelioticus* Speiser, 1914: 10. Holotype female (not located). Type locality: Cameroon, Soppo.

*Linnaemya aptus* Curran, 1934b: 19. Holotype male (BMNH). Type locality: Uganda, Bugoma [as “Bujoma”] Forest [ca. 1°16′N 30°57′E].

***alboscutellata*** (Speiser, 1910).—Afrotropical: “widespread W. Afr. & E. Afr., south to Malawi” (Crosskey 1980b: 846), including Cameroon, D.R. Congo, Ghana, Kenya, Malawi, Nigeria, Sierra Leone, South Africa, Tanzania, Uganda.

*Micropalpus alboscutellatus* Speiser, 1910: 138. Lectotype, unspecified sex (NHRS), by fixation of van Emden (1960: 430) (examination of “type” from Kibonoto in NHRS is regarded as a lectotype fixation). Type locality: Tanzania, Mt. Kilimanjaro [as “Kilimandjaro”], Kibongoto [as “Kibonoto”].

*alboscutatus*. Incorrect subsequent spelling of *alboscutellatus* Speiser, 1910 (Curran 1928b: 398).

***alopecina*** (Speiser, 1910).—Afrotropical: “widespread W. Afr., E. Afr. & sthn Afr.” (Crosskey 1980b: 846), including D.R. Congo, Ethiopia, Ghana, Kenya, Malawi, Nigeria, Sierra Leone, South Africa, Tanzania, Uganda.

*Micropalpus alopecinus* Speiser, 1910: 137. Lectotype female (NHRS), by fixation of van Emden (1960: 449) (examination of “type” from Meru in NHRS is regarded as a lectotype fixation). Type locality: Tanzania, Mt. Meru, 3000m.

Note: Speiser (1910: 137) described *Micropalpus alopecinus* from three females from the area of “Kilimandjaro” [now Kilimanjaro], with one female further restricted to Mt. Meru at 3000m. Van Emden (1960: 449) examined the “type” from “Kilimanjaro, Meru, 3000m” in NHRS and this specimen is accepted as the lectotype. Villeneuve (1914a: 439) had earlier remarked that he had examined the “type” of *Micropalpus alopecinus* but did not provide sufficient details about the specimen for his comment to qualify as a lectotype fixation.

*Linnaemyia conformis* Curran, 1927c: 19. Holotype female (AMNH). Type locality: D.R. Congo, Orientale, Kisangani [as “Stanleyville”].

*Linnaemya shillitoi* Curran, 1934b: 17. Holotype male (BMNH). Type locality: Uganda, Toro, Nyakasura [as “Makasura”, ca. 0°40′N 30°13′E].

*Linnaemya tarsalis* Curran, 1934b: 16 (as “*tarsalis* Villeneuve”). *Nomen nudum*.

***andrewesi*** van Emden, 1960.—Afrotropical: South Africa.

*Linnaemya andrewesi* van Emden, 1960: 450. Holotype male (BMNH). Type locality: South Africa, Eastern Cape, Ongeluksnek [ca. 30°20′S 28°15′E].

***angulicornis*** (Speiser, 1910).—Afrotropical: distribution uncertain but including D.R. Congo and Tanzania, not Palaearctic; given as “widespread W. Afr., n.-e. Afr., E. Afr. & sthn Afr.” by Crosskey (1980b: 847) but distribution confused with that of *Linnaemya neavei* Curran, 1934.

*Micropalpus angulicornis* Speiser, 1910: 138. Holotype male (NHRS). Type locality: Tanzania, Mt. Kilimanjaro [as “Kilimandjaro”].

*Linnaemyia breviseta* Villeneuve, 1941a: 109. Holotype female (CNC). Type locality: D.R. Congo, Sud-Kivu, Kabare.

Note: Van Emden (1960: 443) synonymized *Linnaemya luckmani* Curran, 1934, *Linnaemya neavei* Curran, 1934, and *Linnaemyia breviseta* Villeneuve, 1941 with *Micropalpus angulicornis* Speiser, 1910. This classification was followed by Crosskey (1980b: 847). Herting (1983a: 3–4) revised the “*Linnaemyia pallida* Komplex” and restored *Linnaemya neavei* to species status. *Linnaemya luckmani* was seemingly treated as a distinct species also, although similiarities between it and each of *Linnaemya neavei* and *Linnaemya pallida* (Jaennicke) were noted. Herting (1983a) did not mention *Linnaemya breviseta* and thus this name is kept in synonymy with *Linnaemya angulicornis* pending further study of the nominal species.

Although van Emden (1960) synonymized *Linnaemya breviseta* with *Linnaemya angulicornis* on page 443, he treated the name as valid in his key on page 414. The characters given for *Linnaemya breviseta* in the key do not fit the holotype and possibly refer to an undescribed species. This error might have been corrected had van Emden not died before the manuscript was submitted for publication.

***angustiforceps*** van Emden, 1960.—Afrotropical: Kenya.

*Linnaemya angustiforceps* van Emden, 1960: 458. Holotype male (BMNH). Type locality: Kenya, east side of forest in Aberdare Mountains, 7300ft.

***argyrozona*** van Emden, 1960.—Afrotropical: Kenya, Tanzania.

*Linnaemya argyrozona* van Emden, 1960: 454. Holotype male (BMNH). Type locality: Kenya, Mt. Kenya.

***assimilis*** (Macquart, 1847).—Afrotropical: Madagascar.

*Micropalpus assimilis* Macquart, 1847: 65 [also 1847: 81]. Type(s), female (lost, Crosskey 1971: 276). Type locality: Madagascar.

***aurantiaca*** Mesnil, 1952.—Afrotropical: D.R. Congo, Rwanda.

*Linnaemyia aurantiaca* Mesnil, 1952a: 6. Holotype male (MRAC). Type locality: D.R. Congo, Nord-Kivu, near Volcan Musule [ca. 1°23′S 29°33′E], “lac Kanyamenoni” [not located], 2300m.

*Linnaemyia aurantiaca endeni* Mesnil, 1955: 361. Holotype female (MRAC). Type locality: Rwanda, Kibuye [a former territory], Yanina [ca. 10km south of the city of Kibuye], 2300m.

*emdeni*. Incorrect subsequent spelling of *endeni* Mesnil, 1955 (van Emden 1960: 416).

***basilewskyi*** Mesnil, 1955.—Afrotropical: Rwanda, Uganda.

*Linnaemyia basilewskyi* Mesnil, 1955: 366. Holotype male (MRAC). Type locality: Rwanda, east slope of Volcan Muhabura [as “Muhavura”], 2100m [ca. 1°23′S 29°44′E].

***bequaerti*** Curran, 1934.—Afrotropical: D.R. Congo, Uganda.

*Linnaemya bequaerti* Curran, 1934b: 18. Holotype female (AMNH). Type locality: Uganda, Behungi [ca. 1°17′S 29°48′E].

Note: Curran (1934b: 19) recorded a paratype of *Linnaemya bequaerti* from “Tshibinda, Tanganyika”. As correctly noted by van Emden (1960: 464) and Crosskey (1980b: 847), Tshibinda is in D.R. Congo [as “Belgian Congo” and “Zaire”, respectively] not Tanzania.

***boxi*** van Emden, 1960.—Afrotropical: Ghana, Sierra Leone.

*Linnaemya boxi* van Emden, 1960: 435. Holotype female (BMNH). Type locality: Sierra Leone, “Jiama” (probably in Jaiama Bongor chiefdom in Bo District).

***brincki*** Verbeke, 1970.—Afrotropical: South Africa.

*Linnaemyia brincki* Verbeke, 1970: 290. Holotype male (MZLU). Type locality: South Africa, Western Cape, Cape Peninsula, Hout Bay, Skoorsteenkop.

***brunneoguttata*** van Emden, 1960.—Afrotropical: D.R. Congo, South Africa, Uganda.

*Linnaemya brunneoguttata* van Emden, 1960: 440. Holotype male (BMNH). Type locality: South Africa, KwaZulu-Natal, Durban.

***caffra*** (Villeneuve, 1916).—Afrotropical: D.R. Congo, Ethiopia, Kenya, Malawi, Rwanda, South Africa, Tanzania, Uganda, Zimbabwe.

*Micropalpus caffer* Villeneuve, 1916c: 471. Syntypes, unspecified number and including at least 1 male (BMNH, NHMW, SAMC [no syntypes located in NHMW or SAMC by JEOH]). Type localities: Malawi (Mt. Mulanje [as “Mt. Mlanje”]), South Africa (KwaZulu-Natal, Durban), and Tanzania (“Tanganyika region”).

*Linnaemya cuthbertsoni* Curran, 1934b: 21. Holotype male (AMNH). Type locality: Zimbabwe, Chirinda Forest [ca. 20°26′S 32°42′E].

***chorleyi*** van Emden, 1960.—Afrotropical: Kenya, Uganda.

*Linnaemya chorleyi* van Emden, 1960: 427. Holotype female (BMNH). Type locality: Uganda, mile 10 on Kampala–Entebbe Road.

***ciliata*** Mesnil, 1952.—Afrotropical: D.R. Congo, Kenya.

*Linnaemyia ciliata* Mesnil, 1952a: 4. Holotype female (MRAC). Type locality: D.R. Congo, Nord-Kivu, Rutshuru, 1285m.

*comta* (Fallén, 1810).—Misidentification, not Afrotropical [known from Palaearctic and Oriental regions and New World].

*Tachina comta* of van Emden (1960: 445, as “*Linnaemya comta*”) and Crosskey (1980b: 847, as *comta* with note “? *soror* Zimin misident.”), not Fallén, 1810. Misidentification (Herting and Dely-Draskovits 1993: 284).

Note: An unknown species was recorded as *Linnaemya comta* (originally described as *Tachina comta* Fallén, 1810) from Sudan by van Emden (1960: 445) and questionably by Crosskey (1980b: 847, with note “? *soror* Zimin misident.”). Misidentification (not recorded from the Afrotropical Region by Herting and Dely-Draskovits 1993: 284).

***conducens*** Villeneuve, 1941.—Afrotropical: Zimbabwe.

*Linnaemyia conducens* Villeneuve, 1941a: 109. Holotype male (BMNH). Type locality: Zimbabwe, Vumba Mountains.

***consobrina*** Villeneuve, 1941.—Afrotropical: Cameroon, South Africa, ?Uganda, Zimbabwe.

*Linnaemyia consobrina* Villeneuve, 1941a: 108. Holotype female (CNC). Type locality: Zimbabwe, Vumba Mountains.

***eburneola*** Villeneuve, 1935.—Afrotropical: Uganda.

*Linnaemyia eburneola* Villeneuve, 1935a: 141. Holotype female (IRSNB). Type locality: Uganda, Rwenzori Range [as “Ruwenzori”], 2300m.

***elgonica*** van Emden, 1960.—Afrotropical: Uganda.

*Linnaemya elgonica* van Emden, 1960: 452. Holotype female (BMNH). Type locality: Uganda, Mt. Elgon.

***ethelia*** Curran, 1934.—Afrotropical: Tanzania, Uganda.

*Linnaemya ethelia* Curran, 1934b: 14. Holotype female (BMNH). Type locality: Tanzania, Amani [ca. 5°7′S 38°38′E].

***flavitarsis*** van Emden, 1960.—Afrotropical: Burundi, Uganda.

*Linnaemya flavitarsis* van Emden, 1960: 456. Holotype male (BMNH). Type locality: Uganda, Semliki National Park [as “Bwamba Valley”, ca. 0°49′N 30°3′E].

*fulvitarsis*. Incorrect subsequent spelling of *flavitarsis* van Emden, 1960 (Crosskey 1980b: 847).

***fumipennis*** van Emden, 1960.—Afrotropical: Uganda.

*Linnaemya fumipennis* van Emden, 1960: 438. Holotype female (BMNH). Type locality: Uganda, west Rwenzori Range [as “W. Ruwenzori”], 8000–9000ft.

***geniseta*** van Emden, 1960.—Afrotropical: Eq. Guinea.

*Linnaemya geniseta* van Emden, 1960: 434. Holotype male (MNCN). Type locality: Equatorial Guinea, Evinayong [as “Ebinayong”].

***gowdeyi*** Curran, 1934.—Afrotropical: Uganda.

*Linnaemya gowdeyi* Curran, 1934b: 16. Holotype female (BMNH). Type locality: Uganda, Rwenzori Range [as “Mt. Ruwenzori”], 10,000ft.

***gracilipalpis*** van Emden, 1960.—Afrotropical: D.R. Congo, Kenya.

*Linnaemya gracilipalpis* van Emden, 1960: 429. Holotype male (BMNH). Type locality: Kenya, Nyeri.

***hirtifrons*** Mesnil, 1952.—Afrotropical: D.R. Congo, Uganda.

*Linnaemyia hirtifrons* Mesnil, 1952a: 5. Holotype female (not located). Type locality: D.R. Congo, Nord-Kivu, south of Lake Edward, “riv. Rwindi”, 1000m [this elevation suggests a location on the river near the town of Rwindi, ca. 0°47′S 29°17′E].

***ingrami*** Curran, 1934.—Afrotropical: “widespread E. Afr. & sthn Afr.” (Crosskey 1980b: 847), including D.R. Congo, Ethiopia, Ghana, Guinea, Kenya, Malawi, Sierra Leone, South Africa, Tanzania, Uganda, Zimbabwe.

*Linnaemya ingrami* Curran, 1934b: 23. Holotype male (BMNH). Type locality: Malawi, Mt. Mulanje [as “Mt. Mlanje”].

*Linnaemya andersoni* Curran, 1934b: 24. Holotype male (BMNH). Type locality: Kenya, Solai District, Sonje Valley (Laikipia Escarpment according to van Emden 1960: 453).

Note: The relative priority of *Linnaemya ingrami* Curran, 1934 and *Linnaemya andersoni* Curran, 1934, when the two are treated as synonyms, was established by van Emden (1960: 453), as the First Reviser (Article 24.2.2 of the *Code*, ICZN 1999).

***jocosa*** (Karsch, 1886).—Afrotropical: Angola, D.R. Congo, Malawi, Nigeria, Uganda.

*Micropalpus jocosus* Karsch, 1886b: 338. Holotype, unspecified sex [female, examined by JEOH] (ZMHB). Type locality: Angola, Pungo Andongo.

*Linnaemya nyasa* Curran, 1934b: 12. Holotype male (BMNH). Type locality: Malawi, Mt. Mulanje [as “Mt. Mlanje”].

*Linnaemya lamborni* Curran, 1934b: 13. Holotype female (BMNH). Type locality: Nigeria, Ibadan.

***keiseri*** Mesnil, 1977.—Afrotropical: Madagascar.

*Linnaemyia keiseri* Mesnil, 1977d: 327. Holotype male (MNHN). Type locality: Madagascar, Antananarivo, Belazao [ca. 19°53′S 46°58′E].

***latigena*** Kugler, 1977.—Afrotropical: U.A. Emirates. Palaearctic: M. East (Israel), N. Africa (NE. Africa).

*Linnaemyia latigena* Kugler, 1977: 3. Holotype male (TAU). Type locality: Egypt, Sinai, Bir Gifgafa Airfield [as “Refidim”, ca. 30°24′N 33°9′E].

***laxiceps*** (Villeneuve, 1916).—Afrotropical: ?Gabon, South Africa.

*Micropalpus analis* Macquart, 1855: 118 [also 1855: 98] (junior secondary homonym of *Linnaemya analis* Robineau-Desvoidy, 1830). Lectotype female (BMNH, Crosskey 1971: 276), by fixation of van Emden (1960: 462) (examination of “type” from “Gabon” in Collin collection [now BMNH] is regarded as a lectotype fixation). Type locality: “Gabon” (in error and probably South Africa according van Emden 1960: 462).

*Tachinomima laxiceps* Villeneuve, 1916c: 472. Lectotype female (SAMC), by fixation of van Emden (1960: 462) (examination of “type” from Cape Town in SAMC is regarded as a lectotype fixation). Type locality: South Africa, Western Cape, Cape Town.

***leucaspis*** van Emden, 1960.—Afrotropical: D.R. Congo.

*Linnaemya leucaspis* van Emden, 1960: 424. Holotype male (MRAC). Type locality: D.R. Congo, Orientale, Mongbwalu.

***lindneri*** Mesnil, 1968b.—Afrotropical: South Africa.

*Linnaemyia lindneri* Mesnil, 1968b: 11. Holotype male (SMNS). Type locality: South Africa, Western Cape, Cape Town.

***longirostris*** (Macquart, 1844).—Afrotropical: “widespread eastern Afr.” (Crosskey 1980b: 847), including D.R. Congo, Eritrea, Ethiopia, Kenya, Malawi, Rwanda, South Africa, Sudan, Tanzania, Uganda, Zambia, Zimbabwe.

*Micropalpus longirostris* Macquart, 1844: 46 [also 1844: 203]. Syntypes, male (lost, Crosskey 1971: 277). Type locality: South Africa, Western Cape, Cape of Good Hope [as “Cap”].

? *Micropalpus longirostris* Macquart, 1845: 273 (junior primary homonym of *Micropalpus longirostris* Macquart, 1844). Type(s), male (not located). Type locality: “France” (probably in error and more likely tropical Africa according to Herting 1984: 189–190, note 79).

*Micropalpus longirostris* Jaennicke, 1867: 389 [also 1868: 81] (junior primary homonym of *Micropalpus longirostris* Macquart, 1844) Type(s), female (SMF). Type locality: Ethiopia, “Simen” (probably the Simien Mountains area).

*Dejeania striata* Jaennicke, 1867: 394 [also 1868: 86]. Type(s), female (SMF). Type locality: Ethiopia, “Simen” (probably the Simien Mountains area).

*Tachinomima expetens* Brauer & Bergenstamm, 1891: 383 [also 1891: 79] (as “*Tachinomima* n. (*Tachina*) *expetens* Wd. litt.”). Lectotype male (NHMW), by fixation of Townsend (1939a: 215) (mention of “Ht male” from Cape of Good Hope in NHMW is regarded as a lectotype fixation). Type locality: South Africa, Western Cape, Cape of Good Hope [as “Cap b. sp.” = “Cap Bonae Spei”].

*Micropalpus salmacinus* Speiser, 1910: 136. Holotype female (NHRS). Type locality: Tanzania, Mt. Kilimanjaro [as “Kilimandjaro”].

? *Elachipalpus rondanii* Townsend, 1916b: 10 (*nomen novum* for *Micropalpus longirostris* Macquart, 1845).

Note: *Micropalpus longirostris* Macquart, 1844 was described from more than one male specimen. The type material was lost long before Townsend (1939a: 215) cited a “Ht male” from Cape of Good Hope in Newmarket [Verrall collection, which contained a portion of Macquart’s types] (Crosskey 1971: 277–278), thus nullifying a possible lectotype fixation.

Herting (1984: 100, 189–190 [note 79]) considered the type locality of “France” for *Micropalpus longirostris* Macquart, 1845 as probably wrong and more likely tropical Africa, suggesting also that “it is quite possible that *longirostris* Macquart 1843 [=1844] from the Cape and *longirostris* Macquart 1845 from ‘France’ are the same species” (p. 190). We have followed Herting (1980) in treating *Micropalpus longirostris* Macquart, 1845 and its replacement name *Elachipalpus rondanii* Townsend, 1916 as questional synonyms of *Micropalpus longirostris* Macquart, 1844.

There are two specimens, one of each sex and each labelled as “Cap” and “Coll. Winthem”, in NHMW that appear to be syntypes of *Tachinomima expetens* Brauer & Bergenstamm, 1891 (examined by JEOH). The single male syntype (bearing a second small label reading “*Micropalpus expotens* [sic] det. B.B”) is accepted as the lectotype fixed by Townsend (1939a: 215).

***luckmani*** Curran, 1934.—Afrotropical: Kenya.

*Linnaemya luckmani* Curran, 1934b: 11. Holotype female (AMNH). Type locality: Kenya, Narok [as “Ngare Narok, Masai Reserve”, ca. 1°5′S 35°52′E].

Note: *Linnaemya luckmani* Curran, 1934, was formerly treated as a synonym of *Linnaemya angulicornis* (Speiser, 1910). See note under *Linnaemya angulicornis* for further details. The holotype of *Linnaemya luckmani* was not listed among the tachinid types in AMNH by Arnaud (1963) but its presence there was recorded by Arnaud and Owen (1981: 217).

***luculenta*** Mesnil, 1977.—Afrotropical: Madagascar.

*Linnaemyia luculenta* Mesnil, 1977d: 328. Holotype male (MNHN). Type locality: Madagascar, Ambohitantely [Réserve Spéciale, ca. 18°10′S 47°17′E].

***maculipes*** (Villeneuve, 1920).—Afrotropical: South Africa.

*Tachinomima maculipes* Villeneuve, 1920a: 154 (as “*maculipes* n. sp. ?”). Syntypes, two females (1 female in NMDA). Type locality: South Africa, Eastern Cape, Willowmore.

*Tachinomima braunsi* Villeneuve, 1930a: 352. Holotype female (NMDA). Type locality: South Africa, Eastern Cape, Willowmore.

***masiceroides*** Villeneuve, 1935.—Afrotropical: Kenya.

Linnaemyia (Micropalpus) masiceroides Villeneuve, 1935a: 141. Holotype, sex uncertain [given as female in species header and as male at end of description] (not located). Type locality: Kenya, Marsabit Lake.

***multisetosa*** (Villeneuve, 1936).—Afrotropical: Kenya, Malawi, Tanzania, Uganda.

*Tachinomima multisetosa* Villeneuve, 1936a: 7. Lectotype female (BMNH), by fixation of van Emden (1960: 463) (mention of “type” from Fort Portal in BMNH is regarded as a lectotype fixation). Type locality: Uganda, Rwenzori Range [as “Kilimandjaro-Ruwenzori”], Fort Portal.

*Tachinomima multisetosa fasciata* Villeneuve, 1936a: 8. Holotype female (not located). Type locality: Uganda, Rwenzori Range [as “Kilimandjaro-Ruwenzori”], Fort Portal.

Note: Villeneuve (1936a: 7–8) described *Tachinomima multisetosa* from one or more specimens (only specifically mentioning the female sex) and “var. *fasciata*” from a single female. The type localities were given jointly as “Kilimandjaro-Ruwenzori: Fort Portal (Dr H.B. Owen)”. Cooper and O’Hara (1996: 73) accepted as the holotype of *Tachinomima multisetosa fasciata* a female in CNC from “Kilimandjaro versan [an error in transcription, label reads “versant”] sud-est” that Villeneuve had labelled as the type of “Tachinomima multisetosa var. albopilosa” (an unpublished name). In our opinion it is better to treat the type of *Tachinomima multisetosa fasciata* as not located and the specimen of “var. *albopilosa*” in CNC as an unpublished “variety” of Villeneuve’s.

***neavei*** Curran, 1934.—Afrotropical: distribution uncertain but including Mozambique; distribution confused with that of *Linnaemya angulicornis* (Speiser, 1910) by Crosskey (1980b: 847, *Linnaemya neavei* in synonymy with *Linnaemya angulicornis*). Palaearctic: Europe (SE. Eur., Turkey), M. East (all).

*Linnaemya neavei* Curran, 1934b: 10. Holotype male (BMNH). Type locality: Mozambique, east of Mt. Mulanje [as “Mt. Mlange”]).

*Micropalpus angulicornis* of van Emden (1960: 442, as “*Linnaemya angulicornis*”), not Speiser, 1910. Misidentification, in part (*Linnaemya angulicornis* with *Linnaemya neavei* Curran, *Linnaemya luckmani* Curran, and *Linnaemya breviseta* Villeneuve in synonymy).

*Micropalpus angulicornis* of Kugler (1980a: 50, as “*Linnaemyia angulicornis*”), not Speiser, 1910. Misidentification of specimen(s) from Israel (Herting 1983a: 4).

*Micropalpus vulpinoides* of Crosskey (1976: 204, as “*Linnaemya vulpinoides*”), not Baranov, 1932 [Oriental]. Misidentification of specimen(s) from Jordan (Herting 1983a: 4).

Note: *Linnaemya neavei* Curran, 1934, was formerly treated as a synonym of *Linnaemya angulicornis* (Speiser, 1910). See note under *Linnaemya angulicornis* for further details.

***nigribarba*** Mesnil, 1977.—Afrotropical: Madagascar.

*Linnaemyia nigribarba* Mesnil, 1977d: 328. Holotype male (MNHN). Type locality: Madgascar, Ambohitantely [Réserve Spéciale, ca. 18°10′S 47°17′E].

***nigritarsis*** van Emden, 1960.—Afrotropical: Kenya.

*Linnaemya nigritarsis* van Emden, 1960: 460. Holotype male (BMNH). Type locality: Kenya, west slopes of Mt. Kenya on Meru–Nyeri Road, 6000–8500 ft.

***pallida*** (Jaennicke, 1867).—Afrotropical: Eritrea, Ethiopia, South Africa.

*Micropalpus pallidus* Jaennicke, 1867: 388 [also 1868: 80]. Lectotype female (SMF), by fixation of Herting (1983a: 4) (mention of “Holotyp[us]” from Abyssinia in SMF is regarded as a lectotype fixation). Type locality: Ethiopia [as “Abyssinia”].

***parcesetosa*** (Villeneuve, 1916).—Afrotropical: “widespread W. Afr., E. Afr., sthn Afr.” (Crosskey 1980b: 848), including D.R. Congo, Ghana, Kenya, Malawi, Nigeria, Sierra Leone, South Africa, Tanzania, Uganda, Zambia.

*Micropalpus parcesetosus* Villeneuve, 1916c: 471. Lectotype male (SAMC), by fixation of van Emden (1960: 426, see also discussion under *Linnaemya sororcula* Villeneuve on p. 427) (mention of “typus” from Cape Town in SAMC is regarded as a lectotype fixation). Type locality: South Africa, Western Cape, Cape Town.

Note: Villeneuve (1916c: 472) gave one of the type localities of *Micropalpus parcesetosus* as “N.W. Rhodesia (Chilanga)”, which is a town south of Lusaka in present-day Zambia. Thus, Crosskey (1980b: 848) erred in citing “Rhodesia” (= Zimbabwe) among the countries of the type localities. Van Emden (1960: 426) noted that some of the localities recorded for *Linnaemya parcesetosa* by Curran (1934b: 10) pertain to *Linnaemya sororcula* Villeneuve, 1941, and the “recorded localities must therefore be disregarded”. The countries listed here for *Linnaemya parcesetosa* are based on the original type localities and records given by van Emden (1960: 426) and Crosskey (1980b: 848).

***pictipennis*** Curran, 1927.—Afrotropical: D.R. Congo.

*Linnaemyia pictipennis* Curran, 1927c: 19. Holotype female (AMNH). Type locality: D.R. Congo, Orientale, Kisangani [as “Stanleyville”].

***pilitarsis*** (Villeneuve, 1913).—Afrotropical: South Africa, Uganda, Zimbabwe.

*Tachinomima pilitarsis* Villeneuve, 1913c: 27 (as “*pilitarsis* (an n. spec.?)” on p. 27 but “est réellement une espèce nouvelle” in note added in proof on p. 46). Holotype male (BMNH). Type locality: Uganda, Ibanda.

Note: Villeneuve (1913c: 46) recorded a male of his new species *Tachinomima pilitarsis* in a note added in proof at the end of his paper, but it was not included in the description of the species and is therefore not a syntype.

***prohecate*** (Speiser, 1910).—Afrotropical: D.R. Congo, Kenya, Malawi, Tanzania, Uganda.

*Micropalpus prohecate* Speiser, 1910: 135. Syntypes, 2 females (NHRS). Type localities: Tanzania, Mt. Kilimanjaro [as “Kilimandjaro”] and one female further restricted to Mt. Kilimanjaro, Kibongoto [as “Kibonoto”], 2000–2500m.

***propleuralis*** van Emden, 1960.—Afrotropical: Kenya.

*Linnaemya propleuralis* van Emden, 1960: 432. Holotype male (BMNH). Type locality: Kenya, Aberdare Mountains, Mt. Kinangop, 8000ft.

***pulchella*** Villeneuve, 1934.—Afrotropical: Benin, Nigeria.

Linnaemyia (Micropalpus) pulchella Villeneuve, 1934c: 410. Lectotype female (BMNH), by fixation of van Emden (1960: 425) (mention of “type” from Oshogbo in BMNH is regarded as a lectotype fixation). Type locality: Nigeria, Oshogbo.

***rhodesiana*** Villeneuve, 1941.—Afrotropical: Kenya, Zimbabwe.

*Linnaemyia rhodesiana* Villeneuve, 1941a: 108. Holotype male (BMNH). Type locality: Zimbabwe, Harare [as “Salisbury”].

Note: Villeneuve (1941a: 108) designated a male from Salisbury as “Type” (= holotype) of *Linnaemyia rhodesiana*. The subsequent type designation by van Emden (1960: 432) of the same specimen was unnecessary.

***rudebecki*** Verbeke, 1970.—Afrotropical: South Africa.

*Linnaemyia rudebecki* Verbeke, 1970: 292. Holotype male (MZLU). Type locality: South Africa, Western Cape, Cape Peninsula, Hout Bay, Skoorsteenkop.

***setinervis*** Mesnil, 1952.—Afrotropical: D.R. Congo, Uganda, Zimbabwe.

*Linnaemyia setinervis* Mesnil, 1952a: 3. Holotype female (MRAC). Type locality: D.R. Congo, Nord-Kivu, Semliki Plain, 900–1000m [ca. 0°10′N 29°37′E].

***somerenana*** van Emden, 1960.—Afrotropical: Uganda.

*Linnaemya somerenana* van Emden, 1960: 445. Holotype male (BMNH). Type locality: Uganda, west Rwenzori Range [as “W. Ruwenzori”], 8000–9000ft.

***sororcula*** Villeneuve, 1941.—Afrotropical: D.R. Congo, Ghana, Kenya, South Africa, Tanzania, Uganda.

*Linnaemyia sororcula* Villeneuve, 1941a: 107. Syntypes, 2 females (1 female in CNC). Type localities: D.R. Congo, Équateur, Eala (CNC syntype) and unknown (“étiquette de localité illisible”).

***strigipes*** Curran, 1934.—Afrotropical: South Africa.

*Linnaemya strigipes* Curran, 1934b: 9. Holotype male (SANC). Type locality: South Africa, Eastern Cape, East London.

***succineiventris*** van Emden, 1960.—Afrotropical: Uganda.

*Linnaemya succineiventris* van Emden, 1960: 437. Holotype male (BMNH). Type locality: Uganda, Rwenzori Range [as “Ruwenzori”], Namwamba Valley, 6500ft.

***sulphurea*** (Villeneuve, 1935).—Afrotropical: Ethiopia.

*Tachinomina sulphurea* Villeneuve, 1935a: 140. Holotype female (BMNH). Type locality: southern Ethiopia (Abua according to van Emden 1960: 460).

***torensis*** Curran, 1934.—Afrotropical: Burundi, D.R. Congo, Rwanda, Uganda.

*Linnaemya torensis* Curran, 1934b: 18. Holotype male (BMNH). Type locality: Uganda, Toro, Nyakasura [as “Nyakasnea”, ca. 0°40′N 30°13′E].

*Linnaemyia patruelis* Mesnil, 1952a: 4. Holotype male (IRSNB). Type locality: Burundi, Bururi, 1900m.

***turbida*** (Brauer & Bergenstamm, 1893).—Afrotropical: D.R. Congo, Kenya, Malawi, South Africa, Tanzania, Uganda, Zambia.

*Erigone turbida* Brauer & Bergenstamm, 1893: 96 [also 1893: 184] (as “*turbida* Wd. Coll. Wth. litt.”). Lectotype female (NHMW), by fixation of Curran (1934b: 21) (examination of “type” from “Cape” is regarded as a lectotype fixation for the single syntype from “Cap b. sp.” in NHMW). Type locality: South Africa, Western Cape, Cape of Good Hope [as “Cap b. sp.” = “Cap Bonae Spei”].

Note: The female specimen in NHMW that is accepted as the lectotype of *Erigone turbida* Brauer & Bergenstamm, 1893 is from “Coll. Winthem” and bears a blue Villeneuve label that reads “*Micropalpus turbidus* Typ. B.B.” (examined by JEOH).

***variegata*** (Wiedemann, 1824).—Afrotropical: Burundi, D.R. Congo, Namibia, South Africa, Tanzania.

*Tachina variegata* Wiedemann, 1824: 42. Lectotype male (ZMUC), by fixation of van Emden (1960: 444) (see note). Type locality: South Africa, Western Cape, Cape of Good Hope [as “Prom. bon. sp.” = “Promontorium Bonae Spei”].

*Tachina vulpina* of Curran (1934b: 20, as “*Linnaemya vulpinus*”), not Fallén, 1810. Misidentification (Crosskey 1980b: 848).

Note: *Tachina variegata* Wiedemann, 1824 was described from one or more males. Curran (1934b: 21) examined the “type” but did not state its depository or where it was from. Van Emden (1960: 444) also examined the “type” and provided specifics about it that serve as a lectotype fixation (“type ‘Cape Good Hope, Dec. 1817, 311’ seen in Copenhagen Museum viii. 48”). There are additionally two males and one female in NHMW from “coll. Winthem” and collected from Cape of Good Hope (labelled as “Cap.” or “Cap. b. sp.”; examined by JEOH). These are unlikely to be syntypes because Wiedemann cited the type(s) in “Museo Westermanni”, since incorporated into ZMUC.

***victoria*** Curran, 1934.—Afrotropical: Madagascar, Nigeria, Tanzania, Uganda, Zimbabwe.

*Linnaemya victoria* Curran, 1934b: 16. Holotype male (AMNH). Type locality: Zimbabwe, Vumba.

***vittiventris*** van Emden, 1960.—Afrotropical: Kenya.

*Linnaemya vittiventris* van Emden, 1960: 441. Holotype female (BMNH). Type locality: Kenya, Aberdare Mountains, Mt. Kinangop, 8000ft.

Undetermined sp.: Yemen (Zeegers 2007: 409).

##### Genus *MARSHALLOMYIA* Fennah, 1960

***MARSHALLOMYIA*** Fennah *in* van Emden, 1960: 464. Type species: *Marshallomyia natalensis* Fennah, 1960, by original designation.

Note: The author of this genus and its type species is R.G. Fennah, not van Emden as generally recorded. The van Emden paper in which these descriptions appeared was published in 1960 after van Emden’s death. Fennah explained about the authorship of these names in a note on the first page of the paper: “The present annotator is responsible for the description of *Marshallomyia* and its single species, for the text figures and for following the original orthography of the generic names *Acemya*, *Linnaemya* and *Echinomya*”.

***natalensis*** Fennah, 1960.—Afrotropical: South Africa.

*Marshallomyia natalensis* Fennah *in* van Emden, 1960: 465. Holotype female (BMNH). Type locality: South Africa, KwaZulu-Natal, Ulundi, 5000–6500ft.

##### Genus *PLAGIOCOMA* Villeneuve, 1916

***PLAGIOCOMA*** Villeneuve, 1916c: 473. Type species: *Plagiocoma crassiseta* Villeneuve, 1916, by monotypy.

***crassiseta*** Villeneuve, 1916.—Afrotropical: South Africa.

*Plagiocoma crassiseta* Villeneuve, 1916c: 474. Holotype female (CNC). Type locality: South Africa, Eastern Cape, Port Elizabeth.

##### Genus *SCHIZOLINNAEA* van Emden, 1960

***SCHIZOLINNAEA*** van Emden, 1960: 407. Type species: *Schizolinnaea mirabilis* van Emden, 1960, by original designation.

Note: A diagnosis of *Schizolinnaea* van Emden, 1960 was published by Robertson and Barraclough (1992: 204).

***mirabilis*** van Emden, 1960.—Afrotropical: Kenya (**new record**, TAU [PC]), Malawi, Tanzania, Uganda, Zimbabwe.

*Schizolinnaea mirabilis* van Emden, 1960: 408. Holotype female (BMNH). Type locality: Uganda, Rwenzori Range [as “Ruwenzori”], Namwamba Valley, 6500ft.

Note: The male of *Schizolinnaea mirabilis* van Emden, 1960 was described for the first time by Robertson and Barraclough (1992: 205).

##### Genus *TRIXOCLEA* Villeneuve, 1916

***TRIXOCLEA*** Villeneuve, 1916c: 497. Type species: *Trixoclea metallica* Villeneuve, 1916, by monotypy.

***metallica*** Villeneuve, 1916.—Afrotropical: South Africa.

*Trixoclea metallica* Villeneuve, 1916c: 498. Holotype male (SAMC). Type locality: South Africa, KwaZulu-Natal, Mfongosi [as “Zululand, M’Fongosi”].

#### Tribe GLAUROCARINI

##### Genus *GLAUROCARA* Thomson, 1869

***GLAUROCARA*** Thomson, 1869: 518. Type species: *Glaurocara flava* Thomson, 1869, by monotypy.

*OESTROCHARIS* Villeneuve, 1927: 118. Type species: *Oestrocharis lutescens* Villeneuve, 1927 (= *Glaurocara flava* Thomson, 1869), by monotypy.

*OESTROCARA* Townsend, 1935: 104. Type species: *Semisuturia nitidiventris* Malloch, 1927, by original designation [Oriental].

*DYSOESTRUS* Villeneuve, 1937b: 2. Type species: *Dysoestrus obesus* Villeneuve, 1937, by monotypy.

Note: *Glaurocara* Thomson, 1869 was treated in the tribe Glaurocarini by Crosskey (1980b: 837). Tschorsnig (1985: 97) included the Glaurocarini in the Ormiini and Ziegler (1998: 192) agreed with this placement. We are doubtful of the monophyly of this group and follow the traditional placement of *Glaurocara* in the tribe Glaurocarini pending further study.

***flava*** Thomson, 1869.—Afrotropical: “widespread W. Afr., E. Afr. to sthn Afr.” (Crosskey 1980b: 837), including D.R. Congo, Kenya, Malawi, Mauritius, Réunion, South Africa, Tanzania.

*Glaurocara flava* Thomson, 1869: 519. Lectotype female (NHRS), by fixation of Townsend (1931: 386) (examination of “Female Ht” from Mauritius in NHRS is regarded as a lectotype fixation). Type locality: Mauritius.

*Oestrocharis lutescens* Villeneuve, 1927: 119. Holotype male (CNC). Type locality: South Africa, Eastern Cape, Willowmore.

Note: The immature stages of *Glaurocara flava* Thomson were described by Crosskey (1965).

***glauca*** Mesnil, 1978.—Afrotropical: Madagascar.

*Glaurocara glauca* Mesnil, 1978b: 281. Holotype female (MNHN). Type locality: Madagascar, Toamasina, Périnet [ca. 18°55′S 48°25′E].

***grandipennis*** Mesnil, 1978.—Afrotropical: Madagascar.

*Glaurocara grandipennis* Mesnil, 1978b: 281. Holotype male (MNHN). Type locality: Madagascar, Fianarantsoa, Andringitra-Ambalavao area, Anjavidilava, 2020m [ca. 22°10′S 46°58′E, within Parc National d’Andringitra].

***leleupi*** (Verbeke, 1960).—Afrotropical: Tanzania.

*Oestrocharis leleupi* Verbeke, 1960: 338. Holotype male (MRAC). Type locality: Tanzania, Uluguru Mountains, Mgeta, Bunduki, 1300m.

***livida*** Mesnil, 1978.—Afrotropical: Madagascar.

*Glaurocara livida* Mesnil, 1978b: 280. Holotype male (MNHN). Type locality: Madagascar, Antananarivo, Manjakatompo [ca. 19°21′S 47°18′E].

***nigrescens*** Mesnil, 1978.—Afrotropical: Madagascar.

*Glaurocara nigrescens* Mesnil, 1978b: 281. Holotype male (MNHN). Type locality: Madagascar, Toliara, Ambatolahy [ca. 19°54′S 45°23′E].

***obesa*** (Villeneuve, 1937).—Afrotropical: D.R. Congo.

*Dysoestrus obesus* Villeneuve, 1937b: 2. Holotype female (IRSNB). Type locality: D.R. Congo, Équateur, Eala.

***russea*** Mesnil, 1978.—Afrotropical: Madagascar.

*Glaurocara russea* Mesnil, 1978b: 280. Holotype male (MNHN). Type locality: Madagascar, Fianarantsoa, Ranomafana [Parc National, ca. 21°13′S 47°26′E].

***townsendi*** van Emden, 1960.—Afrotropical: D.R. Congo, Sierra Leone.

*Glaurocara townsendi* van Emden, 1960: 355. Holotype female (BMNH). Type locality: Sierra Leone.

***violacea*** Mesnil, 1978.—Afrotropical: Madagascar.

*Glaurocara violacea* Mesnil, 1978b: 281. Holotype female (MNHN). Type locality: Madagascar, Toamasina, Périnet [ca. 18°55′S 48°25′E].

#### Tribe GRAPHOGASTRINI

##### Genus *GRAPHOGASTER* Rondani, 1868

***GRAPHOGASTER*** Rondani, 1868a: 46. Type species: *Graphogaster vestitus* Rondani, 1868, by original designation (see O’Hara et al. 2011: 91).

Note: *Graphogaster* Rondani, 1868 was first recorded from the Afrotropical Region by Cerretti et al. (2013: 25) based on two specimens of an undescribed species from South Africa (see below).

Undescribed sp.: South Africa (MZUR, NMDA, Cerretti et al. 2013: 25).

##### Genus *PHYTOMYPTERA* Rondani, 1845

***PHYTOMYPTERA*** Rondani, 1845: 32, 33. Type species: *Phytomyptera nitidiventris* Rondani, 1845 (= *Tachina nigrina* Meigen, 1824), by monotypy [Palaearctic].

*ELFIA* Robineau-Desvoidy, 1849a: 158. *Nomen nudum* (no description or included species).

*ELFIA* Robineau-Desvoidy, 1850: 190. Type species: *Actia cingulata* Robineau-Desvoidy, 1830, by subsequent designation of Robineau-Desvoidy (1863a: 672) [Palaearctic].

***aurantia*** Barraclough, 1986.—Afrotropical: South Africa.

*Phytomyptera aurantia* Barraclough, 1986: 230. Holotype male (BMNH). Type locality: South Africa, Eastern Cape, East London.

***aurocrista*** (Barraclough, 1986).—Afrotropical: South Africa.

*Elfia aurocrista* Barraclough, 1986: 223. Holotype male (NMDA). Type locality: South Africa, Western Cape, Paarl District, Du Toits Kloof, 2000–3500ft.

***biseta*** (Barraclough, 1986).—Afrotropical: South Africa.

*Elfia biseta* Barraclough, 1986: 224. Holotype female (NMDA). Type locality: South Africa, Northern Cape, 25 miles SSW of Springbok, Messelpadpas, 1100ft.

***clavapalpa*** (Barraclough, 1986).—Afrotropical: South Africa.

*Elfia clavapalpa* Barraclough, 1986: 225. Holotype female (NMDA). Type locality: South Africa, Northern Cape, Calvinia District, Brandkop area.

***lacteipennis*** Villeneuve, 1934.—Afrotropical: U.A. Emirates. Palaearctic: Europe (W. Eur., E. Eur., SW. Eur., SE. Eur.), M. East (Israel), Mongolia, N. Africa (NE. Africa), Russia (W. Russia).

*Phytomyptera lacteipennis* Villeneuve, 1934d: 71. Lectotype female (CNC), by fixation of Cooper and O’Hara (1996: 63) (data on “Holotype ♀” from Suez in CNC is regarded as a lectotype fixation). Type locality: Egypt, Suez.

***longiarista*** O’Hara & Cerretti, **nom. n.**—Afrotropical: South Africa.

*Phytomyzoneura aristalis* Villeneuve, 1936a: 2 (junior secondary homonym of *Phasiostoma aristalis* Townsend, 1915). Holotype female (CNC). Type locality: South Africa, “Colonie du Cap” ([former Cape Province], “S. W. Distr Cape Col.” according to label data, Cooper and O’Hara 1996: 63; possibly referring to present-day Western Cape, Cape of Good Hope).

*Phytomyptera longiarista* O’Hara & Cerretti, **nom. n.** for *Phytomyzoneura aristalis* Villeneuve, 1936.

Note: *Phytomyzoneura aristalis* Villeneuve, 1936 is a junior secondary homonym of *Phasiostoma aristalis* Townsend, 1915, the valid name of a Nearctic species of *Phytomyptera* (O’Hara and Wood 2004: 254). We hereby propose the new name *Phytomyptera longiarista* to replace the preoccupied name *Phytomyzoneura aristalis* Villeneuve. The same type material applies to the new name. The specific epithet *longiarista* is formed from the Latin word *longus* (long) and arista, alluding to the elongate antenna mentioned in the original description and which likely inspired Villeneuve’s name *aristalis*.

***lunata*** Barraclough, 1986.—Afrotropical: Zimbabwe.

*Phytomyptera lunata* Barraclough, 1986: 232. Holotype male (BMNH). Type locality: Zimbabwe, Mutare [as “Umtali”] District, Vumba.

***maurokara*** (Barraclough, 1986).—Afrotropical: South Africa.

*Elfia maurokara* Barraclough, 1986: 227. Holotype male (NMDA). Type locality: South Africa, Western Cape, Wellington District, Bainskloof, 2000ft.

***mediaposita*** Barraclough, 1986.—Afrotropical: Namibia, South Africa.

*Phytomyptera mediaposita* Barraclough, 1986: 233. Holotype male (NMDA). Type locality: South Africa, Western Cape, north of Vanrhynsdorp, Knersvlakte.

***spinacrista*** Barraclough, 1986.—Afrotropical: Uganda.

*Phytomyptera spinacrista* Barraclough, 1986: 235. Holotype female (BMNH). Type locality: Uganda, Rwenzori Range [as “Ruwenzori Range”], Mahoma River, 6700ft.

***spinosovirilia*** (Barraclough, 1986).—Afrotropical: South Africa.

*Elfia spinosovirilia* Barraclough, 1986: 228. Holotype male (NMDA). Type locality: South Africa, Western Cape, Wellington District, Bainskloof, 2000ft.

***yemenensis*** Barraclough, 1986.—Afrotropical: Yemen.

*Phytomyptera yemenensis* Barraclough, 1986: 236. Holotype male (BMNH). Type locality: Yemen, 1 mile north of Ta‘izz, Usaifira, ca. 4500ft.

Undescribed sp.: Madagascar (TAU, examined by PC).

##### Genus *SARRORHINA* Villeneuve, 1936

***SARRORHINA*** Villeneuve, 1936a: 1. Type species: *Sarrorhina pupilla* Villeneuve, 1936, by monotypy.

*SARRHORINA*. Incorrect subsequent spelling of *Sarrorhina* Villeneuve, 1936 (Crosskey 1980b: 842).

Note: *Sarrorhina* Villeneuve, 1936 is moved here to the Graphogastrini from Crosskey’s (1980b: 842) placement in the Minthoini, **comb. n.**

***pupilla*** Villeneuve, 1936.—Afrotropical: South Africa.

*Sarrorhina pupilla* Villeneuve, 1936a: 2. Syntypes, 2 males and 1 female (CNC). Type locality: South Africa, 3600ft ([Western Cape], Winterhoek Mountains [as “Wind.hoek” and “Wint-hoek”], Tulbagh, according to label data, Cooper and O’Hara 1996: 68).

#### Tribe LESKIINI

##### Genus *AUSTROSOLIERIA* Cerretti & O’Hara, gen. n.

***AUSTROSOLIERIA*** Cerretti & O’Hara, **gen. n.** Type species: *Austrosolieria londti* Cerretti sp. n., by present designation.

Note: This new genus and the two new species below are described in the New Taxa of Afrotropical Tachinidae section.

***freidbergi*** Cerretti & O’Hara, **sp. n.**—Afrotropical: Malawi.

*Austrosolieria freidbergi* Cerretti & O’Hara, **sp. n.** Holotype female (TAU). Type locality: Malawi, Nyika National Park, 15km north of Chelinda (10°30.1′S 33°48.8′E).

***londti*** Cerretti & O’Hara, **sp. n.**—Afrotropical: South Africa.

*Austrosolieria londti* Cerretti & O’Hara, **sp. n.** Holotype male (NMDA). Type locality: South Africa, KwaZulu-Natal, Garden Castle Nature Reserve (29°44′51″S 29°12′36″E).

##### Genus *CLAUSICELLA* Rondani, 1856

***CLAUSICELLA*** Rondani, 1856: 61. Type species: *Clausicella suturata* Rondani, 1856 (as “*Claus: Suturata* Mihi”), by original designation (see O’Hara et al. 2011: 61) [Palaearctic].

*ISTOGLOSSA* Rondani, 1856: 77. Type species: *Istoglossa puella* Rondani, 1856, by original designation [Palaearctic].

*HISTOGLOSSA* Bezzi & Stein, 1907: 393. Unjustified emendation of *Istoglossa* Rondani, 1856 (see O’Hara et al. 2011: 101).

*HASMICA* Richter, 1972: 955. Type species: *Hasmica xanthocera* Richter, 1972, by original designation.

*PERISTOGLOSSA* Mesnil, 1973: 1127 (as subgenus of *Istoglossa* Rondani, 1856). Type species: Istoglossa (Peristoglossa) aurantiaca Mesnil, 1973, by original designation.

Note: The relative priority of *Clausicella* Rondani, 1856 and *Istoglossa* Rondani, 1856, when the two are treated as synonyms, was established by Brauer and Bergenstamm (1891: 445 [also 1891: 141]), as the First Reviser (Article 24.2.2 of the *Code*, ICZN 1999).

***aurantiaca*** (Mesnil, 1973).—Afrotropical: Senegal. Oriental: India.

Istoglossa (Peristoglossa) aurantiaca Mesnil, 1973: 1127. Holotype male (CNC). Type locality: Senegal, Bambey.

***xanthocera*** (Richter, 1972).—Afrotropical: U.A. Emirates. Palaearctic: C. Asia, Mongolia. Oriental: Pakistan.

*Hasmica xanthocera* Richter, 1972: 956. Holotype male (ZIN). Type locality: Mongolia, Bayanhongor aimak, Dzun-mod [likely referring to “Oase Dzun mod, cca 100 km S von Somon Schine zinst”, Marusik and Logunov 2006: 44].

*xanthomera*. Incorrect subsequent spelling of *xanthocera* Richter, 1972 (Zeegers 2010: 682).

Undescribed sp. 1: Namibia (MZUR, examined by PC).

Undescribed sp. 2: South Africa (NMDA, examined by PC).

##### Genus *COLOLESKIA* Villeneuve, 1939

***COLOLESKIA*** Villeneuve, 1939: 2. Type species: *Cololeskia pallida* Villeneuve, 1939, by monotypy.

***pallida*** Villeneuve, 1939.—Afrotropical: ?Kenya, ?Senegal, Zimbabwe.

*Cololeskia pallida* Villeneuve, 1939: 3. Holotype male (BMNH). Type locality: Zimbabwe, Hurungwe [as “Urungwe”], Gota Gota.

Note: Crosskey (1984: 255) recorded a male from Kenya and a female from Senegal belonging to *Cololeskia* Villeneuve, 1939 and possibly conspecific with *Cololeskia pallida* Villeneuve, 1939.

##### Genus *CYANOLESKIA* Mesnil, 1978

***CYANOLESKIA*** Mesnil, 1978a: 110. Type species: *Cyanoleskia leucohalterata* Mesnil, 1978, by original designation.

***leucohalterata*** Mesnil, 1978.—Afrotropical: Madagascar.

*Cyanoleskia leucohalterata* Mesnil, 1978a: 112. Holotype male (MNHN). Type locality: Madagascar, Antananarivo, Manjakatompo [ca. 19°21′S 47°18′E].

##### Genus *LESKIA* Robineau-Desvoidy, 1830

***LESKIA*** Robineau-Desvoidy, 1830: 100. Type species: *Leskia flavescens* Robineau-Desvoidy, 1830 (= *Tachina aurea* Fallén, 1820), by monotypy [Palaearctic].

Note: The following species are provisionally assigned to *Leskia* Robineau-Desvoidy, 1830 pending further study. A revision of these species may determine that some of them should be reassigned to *Fischeria* Robineau-Desvoidy, 1830 or *Solieria* Robineau-Desvoidy, 1849. Crosskey (1980b: 844) treated *Fischeria* and *Solieria* as synonyms of *Leskia* but all three are currently recognized as distinct genera (e.g., Herting and Dely-Draskovits 1993, Tschorsnig and Richter 1998).

***bwambana*** van Emden, 1960.—Afrotropical: Uganda.

*Leskia hirtula bwambana* van Emden, 1960: 391. Holotype female (BMNH). Type locality: Uganda, Semliki National Park [as “Bwamba Valley”, ca. 0°49′N 30°3′E].

***darwini*** van Emden, 1960.—Afrotropical: South Africa.

*Leskia darwini* van Emden, 1960: 391. Holotype male (BMNH). Type locality: South Africa, Western Cape, Cape of Good Hope.

***hirtula*** (Villeneuve, 1936).—Afrotropical: D.R. Congo, Ghana, Kenya, Malawi, Nigeria, Sierra Leone, South Africa, Uganda, Zimbabwe.

*Myiobia hirtula* Villeneuve, 1936a: 5. Lectotype female (BMNH), by designation of van Emden (1960: 390). Type locality: Nigeria, Osogbo [as “Oshogbe”].

*Fischeria capensis* Curran, 1941: 5. Holotype male (SANC). Type locality: South Africa, Eastern Cape, East London.

***lineata*** van Emden, 1960.—Afrotropical: D.R. Congo, Uganda.

*Leskia lineata* van Emden, 1960: 395. Holotype male (BMNH). Type locality: Uganda, Kampala.

***lineaticollis*** van Emden, 1960.—Afrotropical: Cameroon, South Africa, Uganda.

*Leskia lineaticollis* van Emden, 1960: 389. Holotype male (BMNH). Type locality: Uganda, Entebbe.

***longirostris*** (Villeneuve, 1937).—Afrotropical: South Africa.

*Myiobia longirostris* Villeneuve, 1937a: 205. Holotype female (CNC). Type locality: Western Cape, “près de Cape-Town” (Knysna according to label data, Cooper and O’Hara 1996: 53 [as “Knyzna C.C.”]).

***macilenta*** Mesnil, 1978.—Afrotropical: Madagascar.

*Leskia macilenta* Mesnil, 1978a: 110. Holotype male (MNHN). Type locality: Madagascar, Fianarantsoa, Ifanadiana [ca. 21°18′S 47°38′E].

***pallidithorax*** van Emden, 1960.—Afrotropical: Sudan.

*Leskia pallidithorax* van Emden, 1960: 394. Holotype male (BMNH). Type locality: Sudan, Delami.

***palliventris*** van Emden, 1960.—Afrotropical: South Africa.

*Leskia palliventris* van Emden, 1960: 397. Holotype male (BMNH). Type locality: South Africa, KwaZulu-Natal, south of Durban.

***pilipleura*** Mesnil, 1978.—Afrotropical: Madagascar.

*Leskia pilipleura* Mesnil, 1978a: 110. Holotype male (MNHN). Type locality: Madagascar, Toamasina, Périnet [ca. 18°55′S 48°25′E].

***pruinosa*** van Emden, 1960.—Afrotropical: Uganda.

*Leskia pruinosa* van Emden, 1960: 396. Holotype male (BMNH). Type locality: Uganda, Rwenzori Range [as “Ruwenzori”], Namwamba Valley, 6500ft.

***sappirina*** Mesnil, 1978.—Afrotropical: Madagascar.

*Leskia sappirina* Mesnil, 1978a: 109. Holotype female (MNHN). Type locality: Madagascar, Toamasina, Périnet [ca. 18°55′S 48°25′E].

***taylori*** van Emden, 1960.—Afrotropical: South Africa.

*Leskia taylori* van Emden, 1960: 392. Holotype male (BMNH). Type locality: South Africa, “Cape Province: Highlands”.

***villeneuvei*** van Emden, 1960.—Afrotropical: Angola, Botswana, Malawi, Nigeria, Uganda.

*Leskia bicolor villeneuvei* van Emden, 1960: 389. Syntypes, 3 males and 4 females (BMNH). Type localities: Botswana (Lobatse [as “Lobatsi”]), Malawi (Maiwale [ca. 14°27′S 35°18′E]), Nigeria (Oshogbo and Yaba [suburb of Lagos]), and Uganda (Semliki National Park [as “Bwamba Country”, ca. 0°49′N 30°3′E]).

*Fischeria bicolor* of Villeneuve (1913c: 36), not Robineau-Desvoidy, 1830. Misidentification (Crosskey 1980b: 845).

##### Genus *OCYPTEROMIMA* Townsend, 1916

***OCYPTEROMIMA*** Townsend, 1916a: 175. Type species: *Ocypteromima polita* Townsend, 1916, by original designation.

*PYRRHOSIELLA* Villeneuve, 1916c: 501. Type species: *Pyrrhosiella cingulata* Villeneuve, 1916 (= *Ocypteromima polita* Townsend, 1916), by monotypy.

*ASBOLEOLA* Villeneuve, 1916c: 503. Type species: *Asboleola elegans* Villeneuve, 1916, by subsequent designation of Townsend (1936b: 66).

Note: Townsend (1916a) was published on 1 February 1916 (Evenhuis 2003b: 40) and Villeneuve (1916c) was published on 8 December 1916 (dated from journal). Thus, new names in the former have priority over those in the latter.

***angustipennis*** (Villeneuve, 1916).—Afrotropical: D.R. Congo, Ghana, ?Nigeria, Sierra Leone, ?Uganda.

*Asboleola angustipennis* Villeneuve, 1916c: 504. Lectotype male (BMNH), by designation of van Emden (1960: 401). Type locality: Sierra Leone, Mendikama [ca. 7°48′N 10°51'W].

Note: Van Emden (1960: 401–402) treated *Asboleola elegans* Villeneuve, 1916 and *Asboleola angustipennis* Villeneuve, 1916 as subspecies of *Asboleola elegans*, thereby establishing, as the First Reviser, the relative priority of these names when the two are treated as synonyms (Article 24.2.2 of the *Code*, ICZN 1999). The two species or subspecies are partly separated geographically, with *Ocypteromima angustipennis* in the west and *Ocypteromima elegans* in the east and transitional forms of uncertain assignment in the middle. This uncertainty is reflected in the distributions given by Crosskey (1980b: 845) and here.

***elegans*** (Villeneuve, 1916).—Afrotropical: D.R. Congo, ?Kenya, Malawi.

*Asboleola elegans* Villeneuve, 1916c: 504. Lectotype male (BMNH), by fixation of Townsend (1939b: 209) (mention of “Ht male” from “Mount Mlanje” in BMNH is regarded as a lectotype fixation). Type locality: Malawi, Mt. Mulanje [as “Mt. Mlanje”].

Note: See note under *Ocypteromima angustipennis* (Villeneuve, 1916).

***polita*** Townsend, 1916.—Afrotropical: “widespread W. Afr. to E. Afr. & sthn Afr.” (Crosskey 1980b: 845), including Angola, D.R. Congo, Ghana, Kenya, Madagascar, Malawi, Mozambique, Nigeria, Sierra Leone, South Africa, Tanzania, Uganda.

*Ocypteromima polita* Townsend, 1916a: 175. Holotype female (USNM). Type locality: Mozambique, Maputo [as “Lorenzo Marques”].

*Pyrrhosiella cingulata* Villeneuve, 1916c: 503. Lectotype female (SAMC), by fixation of Townsend (1939b: 231) (mention of “Ht female” from Durban in SAMC is regarded as a lectotype fixation). Type locality: South Africa, KwaZulu-Natal, Durban.

Note: Van Emden (1960: 401) designated a female syntype of *Pyrrhosiella cingulata* Villeneuve, 1916 from Sierra Leone (Bendu) in BMNH as lectotype. However, Townsend’s (1939b: 231) lectotype fixation was earlier and has priority. There is a single female syntype from Durban in SAMC (examined by JEOH) and it is accepted as Townsend’s lectotype. There is also a female syntype from “Stella B” [former Stella Bush near Durban] in SAMC that Villeneuve labelled as “Typ.” but it was not published as the holotype and thus has the status of paralectotype. Villeneuve also labelled as “Typ.” a specimen in IRSNB from Oshogbo, Nigeria.

##### Genus *OXYMEDORIA* Villeneuve, 1916

***OXYMEDORIA*** Villeneuve, 1916c: 505. Type species: *Oxymedoria palpata* Villeneuve, 1916, by monotypy.

***palpata*** Villeneuve, 1916.—Afrotropical: Nigeria.

*Oxymedoria palpata* Villeneuve, 1916c: 506. Holotype female (BMNH). Type locality: Nigeria, Osogbo [as “Oshogbe”].

Undescribed sp.: D.R. Congo (BMNH, Crosskey 1984: 254).

##### Unplaced species of Leskiini

***stuckenbergi*** Verbeke, 1973.—Afrotropical: Mozambique. **Comb. n.**

*Hemiwinthemia stuckenbergi* Verbeke, 1973: 6. Holotype female (IRSNB). Type locality: Mozambique, Manica-Sofala District, Gorongosa [as “Gorongoza”] Mountain.

Note: *Hemiwinthemia stuckenbergi* Verbeke, 1973 was overlooked by Crosskey (1980b) but was recorded from the Afrotropical Region without study or change in genus by Crosskey (1984: 201). It is moved here based on examination of the holotype by PC. It cannot be placed to genus at the present time.

#### Tribe MACQUARTIINI

##### Genus *CHYULUELLA* van Emden, 1960

***CHYULUELLA*** van Emden, 1960: 321. Type species: *Chyuluella cribrata* van Emden, 1960, by original designation.

Note: *Chyuluella* van Emden, 1960 was treated as an unplaced genus of Tachinidae by Crosskey (1980b: 881) but was placed in Macquartiini by Crosskey (1984: 200, 250).

***cribrata*** van Emden, 1960.—Afrotropical: Kenya.

*Chyuluella cribrata* van Emden, 1960: 322. Holotype female (BMNH). Type locality: Kenya, Chyulu Hills, 4000ft.

##### Genus *MACQUARTIA* Robineau-Desvoidy, 1830

***MACQUARTIA*** Robineau-Desvoidy, 1830: 204. Type species: *Macquartia rubripes* Robineau-Desvoidy, 1830 (= *Tachina dispar* Fallén, 1820), by subsequent designation of Townsend (1916b: 7) [Palaearctic].

***aeneiventris*** van Emden, 1960.—Afrotropical: Uganda.

*Macquartia aeneiventris* van Emden, 1960: 327. Holotype female (BMNH). Type locality: Uganda, Kigezi District, Kanaba Gap, 7500ft [ca. 1°16′S 29°46′E].

***erythromera*** van Emden, 1960.—Afrotropical: D.R. Congo, Ethiopia, South Africa.

*Macquartia erythromera* van Emden, 1960: 328. Holotype male (BMNH). Type locality: southern Ethiopia, “Higo Samula”.

Note: According to a note by H. Scott *in* van Emden (1941: 224), printed labels in BMNH bearing the locality “Higo Samula” are in error. The name resulted from an unfortunate combination of two place names, “Higo” and “Samalu” (not “Samula”). Specimens from “Higo Samula” originate from either Higo or Samalu, both in southern Ethiopia and about 100 miles apart (see van Emden 1941: 224 for the specific locations of Higo and Samalu).

***nitidicollis*** van Emden, 1960.—Afrotropical: Kenya.

*Macquartia nitidicollis* van Emden, 1960: 328. Holotype male (BMNH). Type locality: Kenya, Jinja.

Note: Zeegers (2010: 683) recognized “Macquartia cf. nitidicollis van Emden” from U.A. Emirates.

***plumbella*** Villeneuve, 1942.—Afrotropical: Zimbabwe.

*Macquartia plumbella* Villeneuve, 1942a: 53. Holotype female (not located). Type locality: Zimbabwe, Harare [as “Salisbury”].

***rufipalpis*** (Curran, 1927).—Afrotropical: South Africa.

*Macroprosopa rufipalpis* Curran, 1927d: 340. Holotype male (SANC). Type locality: South Africa, Eastern Cape, Klipplaat.

***tessellata*** van Emden, 1960.—Afrotropical: South Africa.

*Macquartia tessellata* van Emden, 1960: 326. Holotype female (BMNH). Type locality: South Africa, Western Cape, Van Rhyns Pass [ca. 31°23′S 19°1′E].

***uniseriata*** van Emden, 1960.—Afrotropical: Rwanda.

*Macquartia uniseriata* van Emden, 1960: 330. Holotype male (MRAC). Type locality: Rwanda, Nkuli [ca. 1°35′S 29°31′E].

Note: Van Emden (1960: 331) gave the type locality of *Macquartia uniseriata* as “Belgian Congo: Gîte de Nkuli, Kusanda” and the collector as “L. Lippans”. The data label of the holotype in MRAC records the locality as “Ruanda: Gîte de Nkuli” and the collector as “L. Lippens”. Thus, the type locality is in Rwanda, not D.R. Congo as given by van Emden (1960: 331, as “Belgian Congo”) and repeated by Crosskey (1980b: 841, as “Zaire”).

##### Genus *PORPHYROMUS* van Emden, 1960

***PORPHYROMUS*** van Emden, 1960: 323. Type species: *Porphyromus caeruleiventris* van Emden, 1960, by original designation.

***caeruleiventris*** van Emden, 1960.—Afrotropical: Kenya.

*Porphyromus caeruleiventris* van Emden, 1960: 323. Holotype female (BMNH). Type locality: Kenya, Naivasha.

Undescribed sp.: South Africa (CNC, MZUR, Cerretti et al. 2013: 28).

#### Tribe MEGAPROSOPINI

Note: A key to the Afrotropical genera of the Megaprosopini (as Microphthalmini) was published by Barraclough (1996a: 124).

##### Genus *AMESIOMIMA* Mesnil, 1950

***AMESIOMIMA*** Mesnil, 1950a: 5. Type species: *Amesiomima fulvella* Mesnil, 1950, by monotypy.

***fulvella*** Mesnil, 1950.—Afrotropical: Rwanda.

*Amesiomima fulvella* Mesnil, 1950a: 5. Holotype female (MRAC). Type locality: Rwanda, foot of Volcan Karisimbi, Lac N’Gando, 2400m [ca. 1°35′S 29°24′E].

Note: The condition of the holotype of *Amesiomima fulvella* Mesnil, 1950 was discussed by Barraclough (1996a: 124).

##### Genus *CYRTOCLADIA* van Emden, 1947

***CYRTOCLADIA*** van Emden, 1947: 668. Type species: *Cyrtocladia unisetosa* van Emden, 1947, by monotypy.

***unisetosa*** van Emden, 1947: 669.—Afrotropical: Kenya, Tanzania.

*Cyrtocladia unisetosa* van Emden, 1947: 669. Holotype male (BMNH). Type locality: Kenya, east side of forest in Aberdare Mountains, 7300ft.

Note: The female specimen in BMNH upon which Crosskey (1980b: 840) based his record of *Cyrtocladia unisetosa* van Emden, 1947 from Angola was later regarded as an undescribed species by Crosskey (1984: 247).

Undescribed sp(p).: Angola (BMNH, Crosskey 1984: 247), Kenya (MZUR and TAU, examined by PC).

##### Genus *MICROPHTHALMA* Macquart, 1844

***MICROPHTHALMA*** Macquart, 1844: 84 [also 1844: 241]. Type species: *Microphthalma nigra* Macquart, 1844 (= *Tachina disjuncta* Wiedemann, 1824), by original designation [Nearctic].

*PRODEXILLA* Townsend, 1933: 461. Type species: *Prodexilla petiolata* Townsend, 1933 (= *Dexia posio* Walker, 1849), by original designation.

*AMESIOCLEA* Villeneuve, 1936a: 1. Type species: *Amesioclea cincta* Villeneuve, 1936 (= *Dexia posio* Walker, 1849), by monotypy.

*MICROPHTHALMIA*. Incorrect subsequent spelling of *Microphthalma* Macquart, 1844 (Adams *in* Williston 1908: 376).

*disjuncta* (Wiedemann, 1824).—Misidentification, not Afrotropical [known from Nearctic and Neotropical regions].

Note: An unknown species was recorded as *Microphthalma disjuncta* (originally described as *Tachina disjuncta* Wiedemann, 1824) from D.R. Congo by Villeneuve (1913c: 39). Misidentification (van Emden 1947: 672).

*europaea* Egger, 1860.—Misidentification, not Afrotropical [known from Palaearctic Region].

Note: An unknown species was recorded as *Microphthalma europaea* Egger, 1860 from D.R. Congo by Curran (1928b: 379). Misidentification (Crosskey 1980b: 840).

***flavipes*** Mesnil, 1950.—Afrotropical: D.R. Congo, Nigeria, Yemen.

*Microphthalma flavipes* Mesnil, 1950a: 4. Holotype female (MRAC). Type locality: D.R. Congo, Nord-Kivu, Volcan Nyamuragira, Mushumangabo, 2075m [ca. 1°26′S 29°16′E].

*Microphthalma nigeriensis* of van Emden (1947: 672), not Villeneuve, 1935. Misidentification (Crosskey 1980b: 840).

Note: Van Emden (1947: 672) included Kenya, Sierra Leone, South Africa, Tanzania, and Uganda in the distribution of “*Microphthalma europaea nigeriensis*” but Crosskey (1980b: 840) did not list these countries in the distribution of *Microphthalma flavipes* Mesnil, 1950.

***nigeriensis*** Villeneuve, 1935.—Afrotropical: Nigeria.

*Microphthalma europaea nigeriensis* Villeneuve, 1935a: 137. Holotype male (CNC). Type locality: Nigeria, Ikot Ekpene.

*nigerensis*. Incorrect subsequent spelling of *nigeriensis* Villeneuve, 1935 (original usage not found but spelling listed by Crosskey 1980b: 840).

***nox*** Zeegers, 2007.—Afrotropical: Yemen.

*Microphthalma nox* Zeegers, 2007: 413. Holotype male (RMNH). Type locality: Yemen, Al Kadan (15°14′52″N 43°15′16″E).

***posio*** (Walker, 1849).—Afrotropical: South Africa.

*Dexia posio* Walker, 1849: 844. Lectotype, unspecified sex [male according to BMNH database] (BMNH), by fixation of van Emden (1947: 671) (examination of “Walker’s type” from “Cape Province” in BMNH is regarded as a lectotype fixation). Type locality: South Africa, Western Cape, Cape of Good Hope [as “Cape”].

*Prodexilla petiolata* Townsend, 1933: 462. Holotype female (NHRS). Type locality: South Africa, Western Cape, Cape of Good Hope.

*Amesioclea cincta* Villeneuve, 1936a: 1. Syntypes, 2 males (1 male in CNC). Type locality: South Africa, “Colonie du Cap” ([Western Cape], Winterhoek Mountains, Tulbagh, 3600ft, according to label data of CNC syntype, Cooper and O’Hara 1996: 13 [Winterhoek Mountains cited as “Wint-hoek” but label has “Wint-hoeck”]).

***sejuncta*** (Walker, 1858).—Afrotropical: South Africa.

*Trixa sejuncta* Walker, 1858: 200 (as “Trixa? sejuncta”). Type(s), female (BMNH). Type locality: South Africa, Western Cape, Cape of Good Hope [as “Cape”].

*Microphthalma capensis* Schiner, 1868: 322. Syntypes, 1 male and 2 females (1 male in NHMW). Type locality: South Africa, Western Cape, Cape of Good Hope [as “Cap”].

##### Genus *MONTANOTHALMA* Barraclough, 1996

***MONTANOTHALMA*** Barraclough, 1996a: 125. Type species: *Montanothalma natalensis* Barraclough, 1996, by original designation.

***natalensis*** Barraclough, 1996.—Afrotropical: South Africa.

*Montanothalma natalensis* Barraclough, 1996a: 127. Holotype male (NMDA). Type locality: South Africa, KwaZulu-Natal, Natal Drakensberg, Forestry Reserve, Cathedral Peak, headwaters of Indumeni River, 8500–9200ft.

#### Tribe MINTHOINI

##### Genus *DYSHYPOSTENA* Villeneuve, 1939

***DYSHYPOSTENA*** Villeneuve, 1939: 4. Type species: *Dyshypostena tarsalis* Villeneuve, 1939, by monotypy. **Status revived.**

*KINANGOPANA* van Emden, 1960: 331. Type species: *Kinangopana edwardsi* van Emden, 1960, by original designation. **Syn. n.**

Note: Crosskey (1984: 252) treated *Kinangopana* van Emden, 1960 as a genus but noted the similarity between *Kinangopana edwardsi* van Emden, 1960 and *Dyshypostena tarsalis* Villeneuve, 1939 (*Dyshypostena* then in synonymy with *Sumpigaster* Macquart, 1855), adding: “If the two are treated in future as congeneric, then *Dyshypostena* will need to be recovered from synonymy with *Sumpigaster* and the name *Kinangopana* sunk as a synonym of *Dyshypostena*”. We agree that these two species are congeneric and have revised the classification accordingly. The characters that distinguish *Dyshypostena* will be given in the Tachinidae chapter of the *Manual of Afrotropical Diptera* (in prep.).

***edwardsi*** (van Emden, 1960).—Afrotropical: Kenya. **Comb. n.**

*Kinangopana edwardsi* van Emden, 1960: 331. Holotype male (BMNH). Type locality: Kenya, Aberdare Mountains, Mt. Kinangop, 8000ft.

Note: *Kinangopana edwardsi* van Emden, 1960 was treated as a species of *Kinangopana* van Emden, 1960 by Crosskey (1980b: 841, 1984: 252) but is moved here to *Dyshypostena* Villeneuve, 1939.

***tarsalis*** Villeneuve, 1939.—Afrotropical: D.R. Congo, Ghana, Tanzania, Zimbabwe. **Comb. revived.**

*Dyshypostena tarsalis* Villeneuve, 1939: 5. Syntypes, 2 females (not located). Type localities: D.R. Congo (Équateur, Eala) and Zimbabwe (Nyanga [as “Inyanga”]).

Note: *Dyshypostena tarsalis* Villeneuve, 1939 was treated as a species of *Sumpigaster* Macquart, 1855 by Crosskey (1980b: 842, 1984: 252) but is moved here to the newly revived genus *Dyshypostena* Villeneuve, 1939.

##### Genus *MESNILUS* Özdikmen, 2007

*ZIMINIOLA* Mesnil, 1978a: 112 (junior homonym of *Ziminiola* Gerasimov, 1930). Type species: *Ziminiola nigella* Mesnil, 1978, by original designation.

***MESNILUS*** Özdikmen, 2007: 166 (*nomen novum* for *Ziminiola* Mesnil, 1978).

***cyanella*** (Mesnil, 1978).—Afrotropical: Madagascar.

*Ziminiola cyanella* Mesnil, 1978a: 114. Holotype male (MNHN). Type locality: Madagascar, Antananarivo, Manjakatompo [ca. 19°21′S 47°18′E].

***hexachaeta*** (Mesnil, 1978).—Afrotropical: Madagascar.

*Ziminiola hexachaeta* Mesnil, 1978a: 114. Holotype female (MNHN). Type locality: Madagascar, Toamasina, Périnet [ca. 18°55′S 48°25′E].

***nigella*** (Mesnil, 1978).—Afrotropical: Madagascar.

*Ziminiola nigella* Mesnil, 1978a: 113. Holotype male (MNHN). Type locality: Madagascar, Antsiranana, Montagne d’Ambre [Parc National, ca. 12°36′S 49°8′E].

***plumosa*** (Mesnil, 1978).—Afrotropical: Madagascar.

*Ziminiola plumosa* Mesnil, 1978a: 114. Holotype female (MNHN). Type locality: Madagascar, Toliara, Sakaraha.

***prasina*** (Mesnil, 1978).—Afrotropical: Madagascar.

*Ziminiola prasina* Mesnil, 1978a: 114. Holotype male (MNHN). Type locality: Madagascar, Antananarivo, Manjakatompo [ca. 19°21′S 47°18′E].

***setosa*** (Mesnil, 1978).—Afrotropical: Madagascar.

*Ziminiola setosa* Mesnil, 1978a: 114. Holotype female (MNHN). Type locality: Madagascar, Toamasina, Moramanga.

##### Genus *MINTHO* Robineau-Desvoidy, 1830

***MINTHO*** Robineau-Desvoidy, 1830: 216. Type species: *Musca compressa* Fabricius, 1787, by subsequent designation of Rondani (1856: 79, as “*Dexia compressa* Meig.”).

***argentea*** Bezzi, 1908.—Afrotropical: “E. Afr., n.-e. Afr.” (Crosskey 1980b: 841), including Botswana, D.R. Congo, Eritrea, Ethiopia, Kenya, Sudan, Uganda.

*Mintho argentea* Bezzi, 1908b: 64. Syntypes, 3 males (MSNM, Arnaud 1982: 12). Type locality: Eritrea, near Adi Keyh [also as Adi Kaie and other spellings, published as “Adi Caiè”, ca. 14°51′N 39°22′E].

***compressa*** (Fabricius, 1787).—Afrotropical: “widespread mainland Afrotrop. Reg.” (Crosskey 1980b: 841, as *praeceps* Scopoli, 1763), including D.R. Congo, Eritrea, Kenya, Nigeria, Somalia, South Africa, Sudan, Tanzania, Yemen. Palaearctic: Europe (W. Eur., SW. Eur., SC. Eur., SE. Eur.), M. East (all), N. Africa (Canary Is., NW. Africa, NE. Africa), Transcaucasia.

*Musca compressa* Fabricius, 1787: 346. Type(s), unspecified sex (3 specimens in ZMUC [1 originally in ZMUK and only wing remaining], according to Zimsen 1964: 492). Type locality: Spain [as “Hispaniae”].

*Mintho capensis* Robineau-Desvoidy, 1830: 217. Type(s), unspecified sex (originally in Dejean’s collection, the Diptera of which are mostly lost; Evenhuis et al. 2010: 238). Type locality: South Africa, Western Cape, Cape of Good Hope [as “Cap de Bonne-Espérance”].

*Tachina isis* Wiedemann, 1830: 304. Syntypes, unspecified number and sex (NHMW [not searched for syntypes by JEOH], ZMHB [2 males examined by JEOH]). Type locality: Egypt.

*Dexia thala* Walker, 1849: 845. Type(s) female (1 female in BMNH according to BMNH database). Type locality: Morocco, Tangier.

*Dexia isapis* Walker, 1849: 848. Type(s) male (1 male in BMNH according to BMNH database). Type locality: Egypt.

*Musca praeceps* of Crosskey (1980b: 841), Zeegers (2007: 414), Dawah (2011: 7), etc. (as a distinct species and usually as senior synonym of *Musca compressa* Fabricius, 1787), not Scopoli, 1763. Misidentification (see note).

Note: Crosskey (1980b: 841) recognized *Musca praeceps* Scopoli, 1763 as a valid name with five synonyms, including *Musca compressa* Fabricius, 1787. Herting (1984: 132) and Herting and Dely-Draskovits (1993: 346) treated *Musca praeceps* Scopoli as a *nomen dubium* and recognized *Musca compressa* Fabricius as the valid name for *Musca praeceps* *sensu* Crosskey (1980b) and others. This interpretation has been followed here. *Tachina isis* Wiedemann, 1830, *Dexia thala* Walker, 1849, and *Dexia isapis* Walker, 1849 were all placed in synonymy with *Musca compressa* by Herting (1984) and Herting and Dely-Draskovits (1993). In the absence of evidence to the contrary, we assume that *Mintho capensis* Robineau-Desvoidy, 1830 should also join this list of synonyms because it was treated as a synonym of *Musca praeceps* (as was *Musca compressa*) by Crosskey (1980b). Further study may reveal that the widespread *Mintho compressa* (Fabricius) is a species complex.

***flavicoxa*** Bezzi, 1911.—Afrotropical: D.R. Congo, Ethiopia, Ghana, Kenya, Malawi, Namibia, Nigeria, Sierra Leone, South Africa, Tanzania, Uganda, Zimbabwe.

*Mintho flavicoxa* Bezzi, 1911: 63. Holotype male (USNM). Type locality: South Africa, Gauteng, Pretoria.

*Mintho lacera africa* Villeneuve, 1913c: 37. Lectotype female (BMNH), by fixation of van Emden (1960: 380) (mention of “type” from Ashanti in BMNH is regarded as a lectotype fixation). Type locality: Ghana, Ashanti.

##### Genus *MINTHODES* Brauer & Bergenstamm, 1889

***MINTHODES*** Brauer & Bergenstamm, 1889: 136 [also 1890: 68]. Type species: *Minthodes pictipennis* Brauer & Bergenstamm, 1889, by monotypy [Palaearctic].

***latifacies*** Herting, 1983.—Afrotropical: Yemen. Palaearctic: Europe (Turkey), M. East (all), Transcaucasia.

*Minthodes latifacies* Herting, 1983a: 5. Holotype male (TAU). Type locality: Syria, “Beit Djan” [not located].

*Minthodes pictipennis* of Kugler (1980a: 51), not Brauer & Bergenstamm, 1889. Misidentification (Herting 1983a: 5).

***rhodesiana*** Villeneuve, 1942.—Afrotropical: Zimbabwe.

*Minthodes rhodesiana* Villeneuve, 1942a: 54. Syntypes, 2 females (CNC). Type locality: Zimbabwe, Hurungwe [as “Urungwe”], Gota Gota.

##### Genus *PLESINA* Meigen, 1838

***PLESINA*** Meigen, 1838: 214. Type species: *Tachina phalerata* Meigen, 1824, by monotypy [Palaearctic].

*XANTHOPETIA* Townsend, 1933: 452. Type species: *Tachina fascipennis* Wiedemann, 1830, by original designation.

*KUGLERIA* Verbeke, 1970: 299 (junior homonym of *Kugleria* Bouwman, 1938). Type species: *Plesina fascipennis claripennis* Mesnil, by monotypy [Palaearctic].

***africana*** Kugler, 1978.—Afrotropical: Nigeria.

*Plesina africana* Kugler, 1978a: 91. Holotype male (BMNH). Type locality: Nigeria, between Kaduna and Keffi.

***fascipennis*** (Wiedemann, 1830).—Afrotropical: Sudan.

*Tachina fascipennis* Wiedemann, 1830: 342. Lectotype male (ZMHB, not located by JEOH), by fixation of Townsend (1932: 33) (examination of “Male Ht” from Nubia in ZMHB is regarded as a lectotype fixation). Type locality: Nubia region [as “Nubien”, a region in southern Egypt and northern Sudan, recorded here as Sudan following Crosskey 1980b: 830].

Note: The lectotype of *Tachina fascipennis* Wiedemann, 1830 was not found in ZMHB, but a male paralectotype in NHMW cited by Townsend (1932: 33, as “male Pt”) was examined by JEOH. A headless female paralectotype cited by Townsend (1932: 33, as “female At”) was not found in NHMW.

##### Genus *PSEUDOMINTHODES* Townsend, 1933

***PSEUDOMINTHODES*** Townsend, 1933: 455. Type species: *Pseudominthodes scutellaris* Townsend, 1933, by original designation.

***scutellaris*** Townsend, 1933.—Afrotropical: South Africa.

*Pseudominthodes scutellaris* Townsend, 1933: 455. Holotype male (NHRS). Type locality: South Africa, “Caffraria” (also known as “Kaffraria”, a former region in Eastern Cape).

##### Genus *ROSSIMYIOPS* Mesnil, 1953

***ROSSIMYIOPS*** Mesnil, 1953a: 145. Type species: *Rossimyiops whiteheadi* Mesnil, 1953, by monotypy.

*MESNILOMYIA* Kugler, 1972: 103. Type species: *Mesnilomyia magnifica* Kugler, 1972, by original designation [Palaearctic].

*PERSEDEA* Richter, 2001: 25. Type species: *Persedea exquisita* Richter, 2001, by original designation.

Note: *Rossimyiops* Mesnil, 1953 was revised and reassigned to the Minthoini by Cerretti et al. (2009).

***austrinus*** Cerretti, 2009.—Afrotropical: Namibia.

*Rossimyiops austrinus* Cerretti *in* Cerretti et al., 2009: 40. Holotype female (NMNW). Type locality: Namibia, Karibib District, Tsaobismund (22°22′40″S 15°44′58″E).

***exquisitus*** (Richter, 2001).—Afrotropical: Yemen. Palaearctic: M. East (M. East).

*Persedea exquisita* Richter, 2001: 28. Holotype female (BMNH). Type locality: Iran, Tehrān.

*Mesnilomyia rufipes* Zeegers, 2007: 411. Holotype female (RMNH). Type locality: Yemen, 12km northwest of Manākhah (15°04′19″N 43°44′27″E).

***subapertus*** (Herting, 1983).—Afrotropical: U.A. Emirates. Palaearctic: C. Asia, M. East (all).

*Mesnilomyia subaperta* Herting, 1983a: 5. Holotype male (SMNS). Type locality: Iran, Kermān, “Djiroft”, Anbarābād [ca. 28°29′N 57°51′E].

Note: Zeegers (2010: 6) included Uzbekistan in the distribution of *Mesnilomyia subaperta* Herting, 1983 and gave Ziegler (1991) as a reference. However, Ziegler (1991: 89) recorded this species from Turkmenistan and not Uzbekistan. Turkmenistan was correctly cited by Cerretti et al. (2009: 50).

***whiteheadi*** Mesnil, 1953.—Afrotropical: South Africa.

*Rossimyiops whiteheadi* Mesnil, 1953a: 145. Holotype male (NMDA). Type locality: South Africa, Eastern Cape, Grahamstown.

Undescribed spp.: Ethiopia (TAU, examined by PC), Nigeria (CNC).

##### Genus *SUMPIGASTER* Macquart, 1855

***SUMPIGASTER*** Macquart, 1855: 124 [also 1855: 104]. Type species: *Sumpigaster fasciatus* Macquart, 1855, by original designation [Australasian].

*MEGISTODEXIA* Townsend, 1933: 456. Type species: *Megistodexia diaristata* Townsend, 1933, by original designation.

*SYNEPLACA* Villeneuve, 1938c: 13. Type species: *Syneplaca ghesquierei* Villeneuve, 1938 (= *Megistodexia diaristata* Townsend, 1933), by monotypy.

*SYNHYPOSTENA* Villeneuve, 1939: 6. Type species: *Synhypostena pedestris* Villeneuve, 1939, by monotypy.

Note: Macquart (1855: 125 [also 1855: 105]) remarked about his new genus *Sumpigaster*, “Le type du genre est de l’Océanie”. This statement is accepted as a type species designation for *Sumpigaster* of the single included species from “l’Océanie. Moreton-Bay”, *Sumpigaster fasciatus* Macquart.

***brunnea*** (Mesnil, 1952).—Afrotropical: D.R. Congo.

*Synhypostena brunnea* Mesnil, 1952a: 10. Holotype male (MRAC). Type locality: D.R. Congo, Nord-Kivu, Volcan Nyamuragira [ca. 1°25′S 29°12′E], “Nyashebe” [not located], 1820m.

***diaristata*** (Townsend, 1933).—Afrotropical: D.R. Congo, Eq. Guinea, Ghana, Uganda.

*Megistodexia diaristata* Townsend, 1933: 456. Holotype male (ZMHB). Type locality: Equatorial Guinea, “Benito District”, “Ülleburg” [not located].

*Syneplaca ghesquierei* Villeneuve, 1938c: 13 (as “*ghesquièrei*”). Syntypes, males and females (1 male in CNC, 1 female in IRSNB). Type locality: D.R. Congo, Équateur, Eala.

***pedestris*** (Villeneuve, 1939).—Afrotropical: D.R. Congo, Ghana, Uganda.

*Synhypostena pedestris* Villeneuve, 1939: 7. Holotype female (not located). Type locality: D.R. Congo, Bas-Congo, Mayumbé [a highland area west of Rivière Congo].

***ruwenzorica*** (van Emden, 1960).—Afrotropical: Uganda.

*Synhypostena brunnea ruwenzorica* van Emden, 1960: 379. Holotype male (BMNH). Type locality: Uganda, Rwenzori Range [as “Ruwenzori”], Namwamba Valley, 6500ft.

##### Genus *TIPULIDOMIMA* Townsend, 1933

***TIPULIDOMIMA*** Townsend, 1933: 458. Type species: *Tipulidomima tessmanni* Townsend, 1933, by original designation.

***tessmanni*** Townsend, 1933.—Afrotropical: Eq. Guinea.

*Tipulidomima tessmanni* Townsend, 1933: 458. Holotype male (ZMHB). Type locality: Equatorial Guinea, “Benito District”, “Ülleburg” [not located].

##### Genus *XIPHOCHAETA* Mesnil, 1968

###### Subgenus *XIPHOCHAETA* Mesnil, 1968

***XIPHOCHAETA*** Mesnil, 1968a: 48. Type species: Xiphochaeta (Xiphochaeta) longicornis Mesnil, 1968, by original designation.

***atratula*** Mesnil, 1968.—Afrotropical: Madagascar.

Xiphochaeta (Xiphochaeta) atratula Mesnil, 1968a: 52. Holotype male (NHMB [“to be returned to MNHN”, O’Hara 1996: 133]). Type locality: Madagascar, Toamasina, Périnet [ca. 18°55′S 48°25′E].

***delicatula*** Mesnil, 1968.—Afrotropical: Madagascar.

Xiphochaeta (Xiphochaeta) delicatula Mesnil, 1968a: 52. Holotype male (NHMB [“to be returned to MNHN”, O’Hara 1996: 138]). Type locality: Madagascar, Toamasina, Périnet [ca. 18°55′S 48°25′E].

***heteronychia*** Mesnil, 1968.—Afrotropical: Madagascar.

Xiphochaeta (Xiphochaeta) heteronychia Mesnil, 1968a: 53. Holotype male (NHMB [“to be returned to MNHN”, O’Hara 1996: 142]). Type locality: Madagascar, Ambohitantely [Réserve Spéciale, ca. 18°10′S 47°17′E].

***longicornis*** Mesnil, 1968.—Afrotropical: Madagascar.

Xiphochaeta (Xiphochaeta) longicornis Mesnil, 1968a: 51. Holotype male (NHMB [“to be returned to MNHN”, O’Hara 1996: 145]). Type locality: Madagascar, Fianarantsoa, Vohiparara [within Parc National de Ranomafana, which is located at ca. 21°13′S 47°26′E].

***macronychia*** Mesnil, 1968.—Afrotropical: Madagascar.

Xiphochaeta (Xiphochaeta) macronychia Mesnil, 1968a: 51. Holotype male (NHMB [“to be returned to MNHN”, O’Hara 1996: 146]). Type locality: Madagascar, Ambohitantely [Réserve Spéciale, ca. 18°10′S 47°17′E].

***velutina*** Mesnil, 1968.—Afrotropical: Madagascar.

Xiphochaeta (Xiphochaeta) velutina Mesnil, 1968a: 52. Holotype male (NHMB [“to be returned to MNHN”, O’Hara 1996: 160]). Type locality: Madagascar, Fianarantsoa, Vohiparara [within Parc National de Ranomafana, which is located at ca. 21°13′S 47°26′E].

###### Subgenus *XIPHOCHAETINA* Mesnil, 1968

*XIPHOCHAETINA* Mesnil, 1968a: 49, 50 (as subgenus of *Xiphochaeta* Mesnil, 1968). Type species: Xiphochaeta (Xiphochaetina) paucibarba Mesnil, 1968, by original designation.

***nudicosta*** (Mesnil, 1978).—Afrotropical: Madagascar.

*Xiphochaetina nudicosta* Mesnil, 1978b: 279. Holotype male (MNHN, not located). Type locality: Madagascar, Toamasina, Périnet [ca. 18°55′S 48°25′E].

***paucibarba*** Mesnil, 1968.—Afrotropical: Madagascar.

Xiphochaeta (Xiphochaetina) paucibarba Mesnil, 1968a: 50. Holotype male (NHMB [“to be returned to MNHN”, O’Hara 1996: 152]). Type locality: Madagascar, Ambohitantely [Réserve Spéciale, ca. 18°10′S 47°17′E].

***reducta*** Mesnil, 1968.—Afrotropical: Madagascar.

Xiphochaeta (Xiphochaetina) reducta Mesnil, 1968a: 50. Holotype male (NHMB [“to be returned to MNHN”, O’Hara 1996: 155]). Type locality: Madagascar, Toamasina, Périnet [ca. 18°55′S 48°25′E].

#### Tribe NEAERINI

##### Genus *NEOPLECTOPS* Malloch, 1930

***NEOPLECTOPS*** Malloch, 1930b: 147. Type species: *Neoplectops nudibasis* Malloch, 1930, by original designation [Oriental].

*POINTELIA* Mesnil, 1956b: 77. Type species: *Craspedothrix veniseta* Stein, 1924 (= *Thryptocera pomonellae* Schnabl & Mokrzecki, 1903), by original designation [Palaearctic].

Note: Crosskey (1980b: 851) cited the type species of *Pointelia* Mesnil, 1956 as “*Pointelia veniseta* Mesnil, 1956”, in error.

***nudinerva*** (Mesnil, 1956).—Afrotropical: Côte d’Ivoire, Ghana, Malawi, Namibia (**new record**, MZUR [PC]), Nigeria.

*Pointelia nudinerva* Mesnil, 1956b: 78. Holotype male (CNC, not located by O’Hara 1996: 150). Type locality: Côte d’Ivoire, Adiopodoumé [also as Adiopo-Doumé, ca. 5°20′N 4°8'W].

Note: Mesnil (1956b: 78) wrote “Type dans ma collection” for *Pointelia nudinerva*. The type should be in CNC but has not been located there.

#### Tribe NEMORAEINI

##### Genus *NEMORAEA* Robineau-Desvoidy, 1830

***NEMORAEA*** Robineau-Desvoidy, 1830: 71. Type species: *Nemoraea bombylans* Robineau-Desvoidy, 1830 (= *Tachina pellucida* Meigen, 1824), by subsequent designation of Townsend (1916b: 8) [Palaearctic].

*CHAETOLYDELLA* Villeneuve, 1916c: 488. Type species: *Chaetolydella natalensis* Villeneuve, 1916, by monotypy.

*NEMOREA* Macquart, 1834: 165 [also 1834: 301]. Unjustified emendation of *Nemoraea* Robineau-Desvoidy, 1830 (see O’Hara et al. 2011: 126).

*NEMOROEA*. Incorrect subsequent spelling of *Nemoraea* Robineau-Desvoidy, 1830 (Macquart 1851b: 155 [also 1851b: 182]).

Note: *Hypotachina* Brauer & Bergenstamm, 1891 was listed as a synonym of *Nemoraea* Robineau-Desvoidy, 1830 by Crosskey (1980b: 843) but was reinstated as a genus endemic to the Neotropical Region by Wood and Zumbado (2010: 1405).

***bequaerti*** van Emden, 1960.—Afrotropical: D.R. Congo, ?Ghana, ?Nigeria.

*Nemoraea bequaerti* van Emden, 1960: 362. Holotype male (BMNH). Type locality: D.R. Congo, Katanga, near Lubumbashi [as “Elisabethville”], Rivière Kafubu.

***capensis*** (Robineau-Desvoidy, 1830).—Afrotropical: “widespread n.-e. Afr., E. Afr. & sthn Afr.” (Crosskey 1980b: 843), including D.R. Congo, Eritrea, Ethiopia, Malawi, South Africa, Nigeria, Zimbabwe.

*Meriania capensis* Robineau-Desvoidy, 1830: 71. Type(s), unspecified sex (MNHN or lost). Type locality: South Africa, Western Cape, Cape of Good Hope [as “cap de Bonne-Espérance”].

*Nemorea rufipes* Macquart, 1844: 54 [also 1844: 211] (as “*rufipes*, Guérin”). Lectotype male (MNHN), by fixation of Crosskey (1971: 280) (examination of “Holotype ♂” from “cap de Bonne-Espérance” in MNHN is regarded as a lectotype fixation). Type locality: South Africa, Western Cape, Cape of Good Hope [as “cap de Bonne-Espérance”].

***discoidalis*** Villeneuve, 1916.—Afrotropical: Burundi, D.R. Congo, Uganda.

*Nemoraea discoidalis* Villeneuve, 1916a: 198. Lectotype female (BMNH), by designation of van Emden (1960: 365). Type locality: Uganda, Tero [as “Jero”] Forest.

***fortuna*** Curran, 1936.—Afrotropical: D.R. Congo, Kenya, Uganda.

*Nemoraea fortuna* Curran, 1936: 14. Holotype male (AMNH). Type locality: D.R. Congo, Tshibinda.

Note: The type locality of *Nemoraea fortuna* Curran, 1936 was published as “Tshibinda, Tanganyika” and the holotype was similarly labelled (Arnaud 1963: 122). As explained by van Emden (1960: 363), this was due to an error in the labelling of Tshibinda material. The type locality of Tshibinda is in D.R. Congo.

***infoederata*** Villeneuve, 1916.—Afrotropical: D.R. Congo, Kenya, Uganda.

*Nemoraea infoederata* Villeneuve, 1916a: 199. Syntypes, 3 males and 1 female (1 male in CNC, Cooper and O’Hara 1996: 54). Type localities: Kenya (Aberdare Mountains, 7300ft) and Uganda (Rwenzori Range [as “Ruwenzori”], 2300m and 2500m).

Note: Van Emden (1960: 365) made the following remark about two syntypes of *Nemoraea infoederata* Villeneuve, 1916 that should be in BMNH: “Both the two typical males from the Aberdare Mts. and the female mentioned by Curran belong to the Commonwealth Institute of Entomology, but have not yet been returned”. These syntypes have not been located.

***intacta*** Villeneuve, 1916.—Afrotropical: Liberia, Nigeria. **Status revived.**

*Nemoraea miranda intacta* Villeneuve, 1916a: 201. Lectotype female (BMNH), by fixation of van Emden (1960: 364) (mention of “type” from Oshogbo in BMNH is regarded as a lectotype fixation). Type locality: Nigeria, Oshogbo.

Note: *Nemoraea miranda intacta* Villeneuve, 1916 was treated as a species by Curran (1936: 14, and recorded from Liberia) and later as a synonym of *Nemoraea miranda* Villeneuve, 1916 by van Emden (1960: 364) and Crosskey (1980b: 843). It is recognized here as a distinct species based on examination of the lectotype by PC.

***longicornis*** Villeneuve, 1916.—Afrotropical: Nigeria, Rwanda, Tanzania.

*Nemoraea longicornis* Villeneuve, 1916a: 201. Lectotype female (BMNH), by fixation of van Emden (1960: 363) (mention of “♀ type” from Oshogbo in BMNH is regarded as a lectotype fixation). Type locality: Nigeria, Oshogbo.

***mendax*** (Mesnil, 1978).—Afrotropical: Madagascar.

*Hypotachina mendax* Mesnil, 1978a: 108. Holotype male (MNHN). Type locality: Madagascar, Fianarantsoa, Ranomafana [Parc National, ca. 21°13′S 47°26′E].

***mira*** (Mesnil, 1978).—Afrotropical: Madagascar.

*Hypotachina mira* Mesnil, 1978a: 108. Holotype male (MNHN). Type locality: Madagascar, Antsiranana, Joffreville.

***miranda*** Villeneuve, 1916.—Afrotropical: Côte d’Ivoire, D.R. Congo, Ghana, Guinea, Kenya, Sierra Leone, Sudan, Uganda.

*Nemoraea miranda* Villeneuve, 1916a: 200. Lectotype male (BMNH), by designation of van Emden (1960: 364). Type locality: Ghana, Aburi.

***moerens*** Villeneuve, 1916.—Afrotropical: D.R. Congo, Malawi, Tanzania.

*Nemoraea moerens* Villeneuve, 1916a: 201. Lectotype male (BMNH), by fixation of van Emden (1960: 361) (mention of “♂ type” from W. Nyasa in BMNH is regarded as a lectotype fixation). Type locality: Malawi [as “W. Nyasa”].

***mutata*** Villeneuve, 1916.—Afrotropical: Uganda.

*Nemoraea miranda mutata* Villeneuve, 1916a: 201. Holotype female (not located; not returned to BMNH [as “Commonwealth Institute of Entomology”] according to van Emden 1960: 364). Type locality: Uganda, Entebbe.

***natalensis*** (Villeneuve, 1916).—Afrotropical: D.R. Congo, Lesotho, Malawi, South Africa, Zambia.

*Chaetolydella natalensis* Villeneuve, 1916c: 490. Syntypes, males and females (BMNH, MSNM [1 “cotype” according to Arnaud 1982: 12], SAMC [1 male and two females, examined by JEOH]). Type localities: Malawi (Mt. Mulanje [as “Mt. Mlanje”]) and South Africa (KwaZulu-Natal, Durban; Western Cape, Cape of Good Hope; “Transvaal” [a former province that occupied much of the northeastern part of the country and has since been subdivided into several provinces]).

Note: Townsend (1939a: 287) cited the “Ht” of *Chaetolydella natalensis* Villeneuve, 1916 from Durban in Rambouillet (Villeneuve’s personal collection, since dispersed). If a single type specimen from Durban is located then it could be accepted as the lectotype of *Chaetolydella natalensis* by fixation of Townsend (1939a: 287). Van Emden (1960: 359) accepted a male in BMNH labelled as “Typ.” by Villeneuve as the type, even though he noted that it is not from one of the type localities.

***paulla*** (Mesnil, 1978).—Afrotropical: Madagascar.

*Hypotachina paulla* Mesnil, 1978a: 107. Holotype male (MNHN). Type locality: Madagascar, Antsiranana, Montagne d’Ambre [Parc National, ca. 12°36′S 49°8′E].

***rubellana*** Villeneuve, 1913.—Afrotropical: Cameroon, D.R. Congo, Ethiopia, Kenya, Rwanda, South Africa, Uganda, Tanzania, Zimbabwe.

*Nemoraea rubellana* Villeneuve, 1913c: 28. Holotype male (BMNH). Type locality: Uganda, Lake George.

*Nemoraea completa* Curran, 1936: 16. Holotype male (BMNH). Type locality: Uganda, Entebbe.

*Nemoraea incerta* Curran, 1936: 17. Holotype male (AMNH). Type locality: Cameroon, Edea [as “Eden” in error, Arnaud 1963: 122].

***semiobscura*** Villeneuve, 1916.—Afrotropical: Kenya.

*Nemoraea discoidalis semiobscura* Villeneuve, 1916a: 199. Holotype male (not located; “The type has not yet been returned to the Commonwealth Institute of Entomology [= BMNH]” according to van Emden 1960: 366). Type locality: Kenya, Aberdare Mountains, 7300ft.

***vulgata*** (Mesnil, 1978).—Afrotropical: Madagascar.

*Hypotachina vulgata* Mesnil, 1978a: 108. Holotype male (MNHN). Type locality: Madagascar, Toamasina, Moramanga.

##### Tribe ORMIINI

###### Genus *AULACEPHALA* Macquart, 1851

***AULACEPHALA*** Macquart, 1851b: 138 [also 1851b: 165]. Type species: *Aulacephala maculithorax* Macquart, 1851, by monotypy.

*Aulacocephala* Gerstaecker, 1864: 1033. Unjustified emendation of *Aulacephala* Macquart, 1851.

*AULACOCEPHALOPSIS* Townsend, 1919a: 165. Type species: *Aulacocephala badia* Gerstaecker, 1864 (= *Aulacephala maculithorax* Macquart, 1851), by original designation.

***maculithorax*** Macquart, 1851.—Afrotropical: Botswana, Cameroon, C.A. Republic, D.R. Congo, Kenya, Liberia, ?Madagascar, Malawi, Mozambique, Nigeria, Sierra Leone, South Africa, Tanzania, Uganda, Zambia.

*Aulacephala maculithorax* Macquart, 1851b: 139 [also 1851b: 166]. Lectotype female (MNHN), by designation of Crosskey (1971: 264). Type locality: ?Madagascar.

*Aulacocephala badia* Gerstaecker, 1864: 1035. Holotype, unspecified sex [female, examined by JEOH] (ZMHB). Type locality: South Africa, “Caffraria” (also known as “Kaffraria”, a former region in Eastern Cape).

Note: Macquart (1851b: 139 [also 1851b: 166]) gave the type locality of *Aulacephala maculithorax* as Madagascar but there is speculation that this was an error for South Africa as reviewed by Nihei (2015: 9).

###### Genus *MEDIOSETIGER* Barraclough, 1983

***MEDIOSETIGER*** Barraclough, 1983: 431. Type species: *Mediosetiger microcephala* Barraclough, 1983, by original designation.

***microcephala*** Barraclough, 1983.—Afrotropical: South Africa.

*Mediosetiger microcephala* Barraclough, 1983: 432. Holotype female (NMDA). Type locality: South Africa, KwaZulu-Natal, Giant’s Castle Game Reserve, 5800ft.

Note: *Mediosetiger microcephala* Barraclough, 1983 was originally described from a single female specimen. It was redescribed from both sexes by Barraclough (1996b: 135).

###### Genus *THEROBIA* Brauer, 1862

***THEROBIA*** Brauer, 1862: 1231. Type species: *Trypoderma abdominalis* Wiedemann, 1830, by monotypy [Oriental].

*XYSTOMIMA* Villeneuve, 1914b: 438. Type species: *Xystomima maculipennis* Villeneuve, 1914, by monotypy.

*PLESIOOESTRUS* Villeneuve, 1914b: 439. Type species: *Plesiooestrus albifacies* Villeneuve, 1914, by monotypy.

*THEROBIOPSIS* Townsend, 1919a: 166. Type species: *Aulacephala braueri* Kertész, 1899, by original designation [Australasian].

*PROXYSTOMIMA* Villeneuve, 1925: 51. Type species: *Proxystomima claripennis* Villeneuve, 1925 (= *Plesiooestrus albifacies* Villeneuve, 1914), by monotypy.

*ORMIOMINDA* Paramonov, 1955: 125. Type species: *Ormiominda rieki* Paramonov, 1955, by original designation [Australasian].

*XISTOMIMA*. Incorrect original spelling of *Xystomima* Villeneuve, 1914 (Villeneuve 1914b: 438).

Note: There are two original spellings of *Xystomima* in Villeneuve (1914b): *Xystomima* (p. 438, etc.) and *Xistomima* (p. 441). The correct original spelling was selected as *Xystomima* by Crosskey (1966b: 103), as the First Reviser (Article 24.2.3 of the *Code*, ICZN 1999).

***albifacies*** (Villeneuve, 1914).—Afrotropical: D.R. Congo, Mozambique, Nigeria, Sierra Leone, Uganda.

*Plesiooestrus albifacies* Villeneuve, 1914b: 441. Holotype female (CNC). Type locality: D.R. Congo, Maniema, Kibombo.

*Proxystomima claripennis* Villeneuve, 1925: 51. Syntypes, 1 male and 1 female (CNC). Type localities: D.R. Congo (Rutshuru [as “Rutschuru” on locality label]) and Nigeria (Ilesha) (localities in parentheses from Cooper and O’Hara 1996: 64).

***bicolor*** (Séguy, 1933).—Afrotropical: Mozambique, Tanzania.

*Proxystomima bicolor* Séguy, 1933: 79. Type(s), male (MNHN). Type locality: Mozambique, “Nova-Choupanga” [near Chemba on Rio Zambezi according to van Emden (1945: 418)].

***leonidei*** (Mesnil, 1965).—Afrotropical: Yemen. Palaearctic: Europe (all except British Is., Scand.), Transcaucasia.

*Plesiooestrus leonidei* Mesnil, 1965: 262. Holotype male (CNC). Type locality: France, Bouches-du-Rhône, near Marseille, Massif de la Sainte-Baume.

***maculipennis*** (Villeneuve, 1914).—Afrotropical: D.R. Congo, Madagascar, Rwanda (**new record**, IRSNB [PC]), Sierra Leone, Uganda.

*Xystomima maculipennis* Villeneuve, 1914b: 441. Holotype female (IRSNB). Type locality: D.R. Congo, Kinshasa [as “Léopoldville”].

***melampodis*** (Séguy, 1969).—Afrotropical: Cameroon.

*Plesiooestrus melampodis* Séguy, 1969: 109. Holotype female (MNHN). Type locality: Cameroon, Yaoundé, Nkolbisson.

***minuta*** (Séguy, 1926).—Afrotropical: Madagascar.

*Proxystomima minuta* Séguy, 1926: 17. Type(s), male (MNHN). Type locality: Madagascar, Mahajanga, Analalava [District], Maromandia.

***tristis*** (Séguy, 1926).—Afrotropical: Eq. Guinea, Nigeria.

*Proxystomima tristis* Séguy, 1926: 17. Type(s), female (MNHN). Type locality: Eq. Guinea, Bioco [as “Fernando Po”].

***umbrinervis*** (Villeneuve, 1925).—Afrotropical: D.R. Congo (**new record**, IRSNB [Verbeke det.]), Mozambique, Rwanda (**new record**, IRSNB [Verbeke det.]), South Africa.

*Xystomima umbrinervis* Villeneuve, 1925: 50. Syntypes, 3 females (2 females in CNC). Type locality: South Africa, KwaZulu-Natal, Durban.

##### Tribe PALPOSTOMATINI

###### Genus *EUTRIXOPSIS* Townsend, 1919

***EUTRIXOPSIS*** Townsend, 1919a: 166. Type species: *Eutrixopsis javana* Townsend, 1919, by original designation [Oriental].

*PALPOSTOMOTRIXA* Townsend, 1927b: 277. Type species: *Palpostomotrixa paradoxa* Townsend, 1927, by original designation [Oriental].

*PARATAMICLEA* Villeneuve, 1936c: 1. Type species: *Paratamiclea pallida* Villeneuve, 1936, by monotypy.

*EUTRIXINA* Curran, 1938: 5. Type species: *Eutrixina fasciata* Curran, 1938 (= *Paratamiclea pallida* Villeneuve, 1936), by original designation.

***conica*** Zeegers, 2007.—Afrotropical: Yemen.

*Eutrixopsis conica* Zeegers, 2007: 407. Holotype male (RMNH). Type locality: Yemen, Ta‘izz (13°34′N 44°02′E).

***hova*** (Villeneuve, 1938).—Afrotropical: Madagascar.

*Paratamiclea pallida hova* Villeneuve, 1938a: 5. Syntypes, males and females (not located). Type locality: Madagascar, Toamasina, Toamasina [as “Tamatave”].

*Eutrixopsis regnardi* Verbeke, 1962a: 163. Holotype male (IRSNB). Type locality: Madagascar, Toamasina, Toamasina [as “Tamatave”].

***kufferathi*** Verbeke, 1962.—Afrotropical: D.R. Congo, ?Nigeria.

*Eutrixopsis kufferathi* Verbeke, 1962a: 162. Holotype male (IRSNB). Type locality: D.R. Congo, Nord-Kivu, Ituri, Lake Albert, Kasenye [as “Kasenyi”].

***pallida*** (Villeneuve, 1936).—Afrotropical: Kenya, Zimbabwe.

*Paratamiclea pallida* Villeneuve, 1936c: 1. Syntypes, 3 males (1 male in CNC). Type locality: Zimbabwe, Harare [as “Salisbury”].

*Eutrixina fasciata* Curran, 1938: 5. Holotype male (AMNH). Type locality: Zimbabwe, “Victoria” (probably Victoria Falls).

***petiolata*** Verbeke, 1962.—Afrotropical: D.R. Congo.

*Eutrixopsis petiolata* Verbeke, 1962a: 161, 163. Holotype male (IRSNB). Type locality: D.R. Congo, Orientale, Parc National de la Garamba, Ndelele.

***pinguis*** Mesnil, 1978.—Afrotropical: Madagascar.

*Eutrixopsis pinguis* Mesnil, 1978b: 283. Holotype female (MNHN). Type locality: Madagascar, Toamasina, Manompana.

###### Genus *HAMAXIA* Walker, 1860

***HAMAXIA*** Walker, 1860: 153. Type species: *Hamaxia incongrua* Walker, 1860, by monotypy [Australasian].

*OCHROMEIGENIA* Townsend, 1919b: 578. Type species: *Ochromeigenia ormioides* Townsend, 1919 (= *Hamaxia incongrua* Walker, 1860), by original designation [Australasian].

*HAMMAXIA*. Incorrect subsequent spelling of *Hamaxia* Walker, 1860 (Brauer and Bergenstamm 1891: 407 [also 1891: 103] and 1893: 143 [also 1893: 231]).

*HAMXIA*. Incorrect subsequent spelling of *Hamaxia* Walker, 1860 (Chao et al. 1998: 2040).

*incongrua* Walker, 1860.—Misidentification, not Afrotropical [known from Palaearctic, Oriental and Australasian regions].

Note: An unknown species was recorded as *Hamaxia incongrua* Walker, 1860 from Tanzania by Verbeke (1960: 335). This was probably the basis for Crosskey’s (1976: 184) record of the species (as *Palpostoma incongruum*) from “? E. Africa”. Crosskey (1980b) normally listed species misidentified from the Afrotropics and noted for each “Not Afrotropical”, but gave no entry for *Hamaxia incongrua*. We here confirm that there is no credible record of *Hamaxia incongrua* from the Afrotropical Region.

###### Genus *PALPOSTOMA* Robineau-Desvoidy, 1830

***PALPOSTOMA*** Robineau-Desvoidy, 1830: 429. Type species: *Palpostoma testacea* Robineau-Desvoidy, 1830, by monotypy [Australasian].

*AFROMEIGENIA* Curran, 1927f: 107. Type species: *Afromeigenia pallens* Curran, 1927, by original designation.

*HAMAXIOMIMA* Verbeke, 1962a: 154. Type species: *Hamaxiomima africana* Verbeke, 1962, by original designation.

***africanum*** (Verbeke, 1962).—Afrotropical: D.R. Congo, ?Mauritius.

*Hamaxiomima africana* Verbeke, 1962a: 158. Holotype male (IRSNB). Type locality: D.R. Congo, Nord-Kivu, Goma.

***cumatilis*** (Mesnil, 1978).—Afrotropical: Madagascar.

*Hamaxia cumatilis* Mesnil, 1978b: 282. Holotype male (MNHN). Type locality: Madagascar, Toamasina, route to Lakato [ca. 19°11′S 48°26′E], Ankasole [not located], 1130m.

***laticorne*** (Verbeke, 1962).—Afrotropical: D.R. Congo, Rwanda.

*Hamaxiomima laticornis* Verbeke, 1962a: 156. Holotype male (IRSNB). Type locality: D.R. Congo, Nord-Kivu, Goma.

***mutatum*** (Villeneuve, 1936).—Afrotropical: D.R. Congo, ?Kenya, South Africa, Tanzania.

*Hamaxia mutata* Villeneuve, 1936a: 6. Syntypes, 2 females (not located). Type locality: South Africa.

*Hamaxiomima picta* Verbeke, 1962a: 160. Holotype male (MRAC). Type locality: Tanzania, Handeni, 350m.

***pallens*** (Curran, 1927).—Afrotropical: D.R. Congo, Kenya, Nigeria, South Africa.

*Afromeigenia pallens* Curran, 1927f: 108. Holotype male (SANC). Type locality: South Africa, Eastern Cape, East London.

***pilosum*** (Verbeke, 1962).—Afrotropical: D.R. Congo.

*Hamaxiomima pilosa* Verbeke, 1962a: 155, 158. Holotype male (IRSNB). Type locality: D.R. Congo, Katanga, Parc National de l’Upemba [as “P.N.U.”], Rivière Lupiala [a tributary of Rivière Lufira], Munoi, 890m.

###### Genus *PERISTASISEA* Villeneuve, 1934

***PERISTASISEA*** Villeneuve, 1934b: 186. Type species: *Peristasisea luteola* Villeneuve, 1934, by original designation.

*HAMAXIOIDES* Mesnil, 1959: 26. Type species: *Hamaxioides mellea* Mesnil, 1959 (= *Peristasisea luteola* Villeneuve, 1934), by monotypy.

***luteola*** Villeneuve, 1934.—Afrotropical: D.R. Congo, Malawi, Nigeria (**new record**, CNC), Sudan, Tanzania, Uganda.

*Peristasisea luteola* Villeneuve, 1934b: 187. Lectotype male (IRSNB), by designation herein (see Lectotype Designations section). Type locality: Malawi.

*Hamaxioides mellea* Mesnil, 1959: 26. Holotype female (SMNS). Type locality: Tanzania, Makoa [probably near Moshi, ca. 3°21′S 37°19′E].

##### Tribe SIPHONINI

###### Genus *ACTIA* Robineau-Desvoidy, 1830

***ACTIA*** Robineau-Desvoidy, 1830: 85. Type species: *Roeselia lamia* Meigen, 1838, by designation under the Plenary Powers of ICZN (1987: 71) [Palaearctic].

***antiqua*** (Mesnil, 1954).—Afrotropical: D.R. Congo.

*Entomophaga antiqua* Mesnil, 1954a: 31. Holotype male (MRAC). Type locality: D.R. Congo, Orientale, Bambesa.

***chrysocera*** Bezzi, 1923.—Afrotropical: Seychelles.

*Actia chrysocera* Bezzi, 1923: 96. Holotype male (BMNH). Type locality: Seychelles, Longue Is.

***ciligera*** (Mesnil, 1954).—Afrotropical: D.R. Congo.

*Entomophaga ciligera* Mesnil, 1954a: 29. Holotype female (MRAC). Type locality: D.R. Congo, Nord-Kivu, Lake Kivu, N’Zulu, 1500m [east of Sake at ca. 1°37′S 29°06′E].

***cuthbertsoni*** Curran, 1933.—Afrotropical: Madagascar, Uganda, Zimbabwe.

*Actia cuthbertsoni* Curran, 1933: 162. Holotype male (AMNH). Type locality: Zimbabwe, Kadoma [as “Gatooma”].

***exsecta*** Villeneuve, 1936.—Afrotropical: Uganda.

*Actia exsecta* Villeneuve, 1936d: 416. Syntypes, 2 males (1 male in BMNH, 1 male in CNC). Type locality: Uganda, Kampala.

***fallax*** (Mesnil, 1954).—Afrotropical: D.R. Congo, Rwanda.

*Entomophaga fallax* Mesnil, 1954a: 29. Holotype female (MRAC). Type locality: D.R. Congo, Nord-Kivu, Volcan Mikeno, near Rweru, 2400m [ca. 1°29′S 29°24′E].

***gratiosa*** (Mesnil, 1954).—Afrotropical: D.R. Congo.

*Entomophaga gratiosa* Mesnil, 1954a: 34. Holotype male (MRAC). Type locality: D.R. Congo, Nord-Kivu, north of Lake Kivu, Goma [as “N’Goma”].

***hargreavesi*** Curran, 1933.—Afrotropical: Uganda.

*Actia hargreavesi* Curran, 1933: 160. Holotype female (BMNH). Type locality: Uganda, Kampala.

*Actia comitata* Villeneuve, 1936d: 416. Syntypes, 4 males and 2 females (1 male and 1 female in BMNH, 1 male in CNC). Type locality: Uganda, Kampala.

***linguata*** Mesnil, 1968.—Afrotropical: South Africa.

*Actia linguata* Mesnil, 1968b: 10. Holotype male (BMNH). Type locality: South Africa, Western Cape, Cape Town, Cape Point.

***longilingua*** (Mesnil, 1954).—Afrotropical: D.R. Congo.

*Entomophaga longilingua* Mesnil, 1954a: 36. Holotype male (MRAC). Type locality: D.R. Congo, Nord-Kivu, Rutshuru, 1285m.

***munroi*** Curran, 1927.—Afrotropical: D.R. Congo, South Africa.

*Actia munroi* Curran, 1927d: 322. Holotype female (SANC). Type locality: South Africa, Mpumalanga, Barberton.

***nigrapex*** Mesnil, 1977.—Afrotropical: Madagascar.

*Actia nigrapex* Mesnil, 1977a: 83. Holotype male (MNHN). Type locality: Madagascar, Antsiranana, Montagne d’Ambre [Parc National, ca. 12°36′S 49°8′E].

***nitidella*** Villeneuve, 1936.—Afrotropical: Kenya, Tanzania, Uganda.

*Actia nitidella* Villeneuve, 1936d: 417. Holotype female (BMNH). Type locality: Uganda, Kampala.

***pallens*** Curran, 1927.—Afrotropical: South Africa.

*Actia pallens* Curran, 1927d: 322. Holotype female (SANC). Type locality: South Africa, KwaZulu-Natal, Durban.

***picipalpis*** (Mesnil, 1954).—Afrotropical: D.R. Congo, Ghana, Kenya.

*Entomophaga picipalpis* Mesnil, 1954a: 33. Holotype female [not male as published] (MRAC). Type locality: D.R. Congo, Nord-Kivu, Rutshuru, “Lubirizi” [not located], 1285m.

***rejecta*** Bezzi, 1926.—Afrotropical: Mauritius.

*Actia rejecta* Bezzi *in* Bezzi & Lamb, 1926: 569. Holotype male [not female as published] (BMNH). Type locality: Mauritius, Rodrigues Is.

***rubiginosa*** (Mesnil, 1954).—Afrotropical: D.R. Congo.

*Entomophaga rubiginosa* Mesnil, 1954a: 35. Holotype male (MRAC). Type locality: D.R. Congo, Nord-Kivu, Mokoto, Burungu [as “Burunga”], 2000m [ca. 1°20′S 29°2′E].

***russula*** Mesnil, 1977.—Afrotropical: Madagascar.

*Actia russula* Mesnil, 1977a: 84. Holotype male (MNHN). Type locality: Madagascar, Antsiranana, Joffreville.

***triseta*** (Mesnil, 1954).—Afrotropical: D.R. Congo, Rwanda.

*Entomophaga triseta* Mesnil, 1954a: 32. Holotype male (MRAC). Type locality: D.R. Congo, Nord-Kivu, Volcan Mikeno, near Rweru, 2400m [ca. 1°29′S 29°24′E].

Note: Crosskey (1980b: 852) gave the type locality of *Entomophaga triseta* Mesnil, 1954 as within Rwanda and this was followed by O’Hara (1996: 159). See note under *Periscepsia rufitibia* (Villeneuve, 1938) for an explanation of the treatment of the type locality as within D.R. Congo.

***vulpina*** (Mesnil, 1954).—Afrotropical: D.R. Congo.

*Entomophaga vulpina* Mesnil, 1954a: 34. Holotype male (MRAC). Type locality: D.R. Congo, Orientale, Bambesa.

Undetermined spp.: Yemen, as “Actia sp. 1 cf. rubiginosa (Mesnil, 1954)” and “Actia sp. 2 cf. nitidella Villeneuve, 1936” (Zeegers 2007: 405).

###### Genus *CEROMYA* Robineau-Desvoidy, 1830

***CEROMYA*** Robineau-Desvoidy, 1830: 86. Type species: *Ceromya testacea* Robineau-Desvoidy, 1830 (= *Tachina bicolor* Meigen, 1824), by subsequent designation of Coquillett (1910: 520) [Palaearctic].

*CEROMYIA* Agassiz, 1846a: 7. Unjustified emendation of *Ceromya* Robineau-Desvoidy, 1830 (see Evenhuis et al. 2010: 54).

Note: The generic limits of *Ceromya* Robineau-Desvoidy, 1830 were revised and the Afrotropical species listed by O’Hara (1989).

***amicula*** Mesnil, 1954.—Afrotropical: D.R. Congo.

*Ceromyia amicula* Mesnil, 1954a: 40. Holotype male (MRAC). Type locality: D.R. Congo, Orientale, Bambesa.

***buccalis*** (Curran, 1933).—Afrotropical: Kenya, Zimbabwe.

*Actia buccalis* Curran, 1933: 163. Holotype male (AMNH). Type locality: Zimbabwe, Kadoma [as “Gatooma”].

***cibdela*** (Villeneuve, 1913).—Afrotropical: D.R. Congo, Mozambique, Nigeria, South Africa, Tanzania.

*Actia cibdela* Villeneuve, 1913c: 35. Lectotype male (CNC), by designation of O’Hara (1989: 55). Type locality: Nigeria, Oshogbo.

*cibdella*. Incorrect subsequent spelling of *cibdela* Villeneuve, 1913 (Curran 1927d: 323).

***femorata*** Mesnil, 1954.—Afrotropical: D.R. Congo, Ghana, Nigeria, Uganda.

*Ceromyia femorata* Mesnil, 1954a: 38. Holotype male (MRAC). Type locality: D.R. Congo, Orientale, Bambesa.

***languidula*** (Villeneuve, 1913).—Afrotropical: D.R. Congo, Nigeria, Uganda.

*Actia languidula* Villeneuve, 1913c: 36. Syntypes, unspecified number and sex (1 male in BMNH, 1 male in CNC, O’Hara 1989: 61). Type locality: Nigeria, Oshogbo.

***languidulina*** Mesnil, 1977.—Afrotropical: Madagascar.

*Ceromyia languidulina* Mesnil, 1977c: 178. Holotype female (MNHN). Type locality: Madagascar, Ambohitantely [Réserve Spéciale, ca. 18°10′S 47°17′E].

***lavinia*** (Curran, 1927).—Afrotropical: Cameroon, D.R. Congo, South Africa.

*Actia lavinia* Curran, 1927d: 324. Holotype female (SANC). Type locality: South Africa, KwaZulu-Natal, “Clan Syndicate” (probably Clan Syndicate Mill, ca. 29°23′S 30°29′E).

***luteicornis*** (Curran, 1933).—Afrotropical: Kenya, Mozambique, Nigeria, South Africa, Uganda, Zimbabwe.

*Actia luteicornis* Curran, 1933: 162. Holotype male (BMNH). Type locality: Zimbabwe.

***natalensis*** (Curran, 1927).—Afrotropical: South Africa.

*Actia natalensis* Curran, 1927d: 325. Holotype male (SANC). Type locality: South Africa, KwaZulu-Natal, Cramond [ca. 29°25′S 30°26′E].

***normula*** (Curran, 1927).—Afrotropical: D.R. Congo, South Africa.

*Actia normula* Curran, 1927d: 322. Holotype male (SANC). Type locality: South Africa, Eastern Cape, East London.

***similata*** Mesnil, 1954.—Afrotropical: D.R. Congo.

*Ceromyia varichaeta similata* Mesnil, 1954a: 39. Holotype female (MRAC). Type locality: D.R. Congo, Mushari [as “Musari”], Tshumba, 2100m [ca. 1°15′S 29°11′E].

***varichaeta*** (Curran, 1927).—Afrotropical: D.R. Congo, South Africa.

*Actia varichaeta* Curran, 1927c: 6. Holotype male (AMNH). Type locality: D.R. Congo, Orientale, Faradje.

Undetermined sp.: Yemen, as “Ceromya sp. 1 cf. cibdela (Villeneuve, 1913)” (Zeegers 2007: 406).

###### Genus *PERIBAEA* Robineau-Desvoidy, 1863

*HERBSTIA* Robineau-Desvoidy, 1851: 184 (junior homonym of *Herbstia* Edwards, 1834). Type species: *Herbstia tibialis* Robineau-Desvoidy, 1851, by monotypy. Placed on the Official Index of Rejected and Invalid Generic Names in Zoology by action of ICZN (1964: 343).

***PERIBAEA*** Robineau-Desvoidy, 1863a: 720. Type species: *Peribaea apicalis* Robineau-Desvoidy, 1863 (= *Herbstia tibialis* Robineau-Desvoidy, 1851), by subsequent designation of Coquillett (1910: 587) [Palaearctic].

*STROBLIOMYIA* Townsend, 1926a: 31. Type species: *Tryptocera fissicornis* Strobl, 1910 (as “*Thryptocera fissicornis*”) (= *Thryptocera setinervis* Thomson, 1869), by original designation [Palaearctic].

***annulata*** (Mesnil, 1954).—Afrotropical: D.R. Congo.

*Strobliomyia annulata* Mesnil, 1954a: 21. Holotype male (MRAC). Type locality: D.R. Congo, Nord-Kivu, “Rwankwi” [probably on road between Goma and Rutshuru at ca. 1°19′S 29°22′E].

***anthracina*** Mesnil, 1977.—Afrotropical: Madagascar.

*Peribaea anthracina* Mesnil, 1977a: 81. Holotype male (MNHN). Type locality: Madagascar, Antananarivo, Belazao [ca. 19°53′S 46°58′E].

***cervina*** (Mesnil, 1954).—Afrotropical: D.R. Congo; South Africa.

*Strobliomyia cervina* Mesnil, 1954a: 18. Holotype male (IRSNB). Type locality: D.R. Congo, Nord-Kivu, Rutshuru.

***clara*** (Mesnil, 1954).—Afrotropical: D.R. Congo.

*Strobliomyia clara* Mesnil, 1954a: 21. Holotype male (MRAC). Type locality: D.R. Congo, Katanga, Kalabi.

***compacta*** (Curran, 1927).—Afrotropical: South Africa.

*Actia compacta* Curran, 1927d: 324. Holotype male (SANC). Type locality: South Africa, Eastern Cape, East London.

***ferina*** (Mesnil, 1954).—Afrotropical: Rwanda.

*Strobliomyia ferina* Mesnil, 1954a: 17. Holotype male (MRAC). Type locality: Rwanda, Volcan Visoke [also known as Bisoke], Kibga, 2400m [ca. 1°29′S 29°31′E].

***gibbicornis*** (Mesnil, 1954).—Afrotropical: D.R. Congo.

*Strobliomyia gibbicornis* Mesnil, 1954a: 19. Holotype male (IRSNB). Type locality: D.R. Congo, Nord-Kivu, Rutshuru.

***jepsoni*** (Villeneuve, 1937).—Afrotropical: Mauritius.

*Strobliomyia jepsoni* Villeneuve, 1937d: 2. Holotype male (CNC). Type locality: Mauritius.

***lobata*** Mesnil, 1977.—Afrotropical: Madagascar.

*Peribaea lobata* Mesnil, 1977a: 80. Holotype male (MNHN). Type locality: Madagascar, Antananarivo, Manjakatompo [ca. 19°21′S 47°18′E].

***longiseta*** (Villeneuve, 1936).—Afrotropical: Uganda.

*Actia longiseta* Villeneuve, 1936d: 417. Holotype female (BMNH). Type locality: Uganda, Kampala.

***mitis*** (Curran, 1927).—Afrotropical: Kenya, South Africa.

*Actia mitis* Curran, 1927d: 323. Syntypes, 1 male and 1 female [as “Types, ♂♀”] (SANC). Type locality: South Africa, Mpumalanga, Barberton.

***modesta*** (Mesnil, 1954).—Afrotropical: D.R. Congo.

*Strobliomyia modesta* Mesnil, 1954a: 14. Holotype male (MRAC). Type locality: D.R. Congo, Nord-Kivu, Rutshuru, Rivière Musugereza, 1100m [ca. 1°05′S 29°27′E].

***orbata*** (Wiedemann, 1830).—Afrotropical: “W. Afr. to n.-e. Afr., E. Afr. & sthn Afr.” (Crosskey 1980b: 853), including D.R. Congo, U.A. Emirates, Uganda, Yemen. Palaearctic: Japan, M. East (all), N. Africa (NE. Africa), P. China. Oriental: India, Indonesia, Orien. China, Malaysia, Myanmar, Philippines, Ryukyu Is., Sri Lanka, Taiwan, Thailand. Australasian: Australia, Melanesia, Micronesia, N. Australasian.

*Tachina orbata* Wiedemann, 1830: 336. Neotype female (BMNH), by designation of Crosskey (1967c: 106) and confirmed by ruling of ICZN (1990). Type locality: India, Assam, Azra.

Gymnopareia (Actia) aegyptia Villeneuve, 1913a: 508. Lectotype male (BMNH), by designation of Crosskey (1966b: 108). Type locality: Egypt (“Dep. Agr. Egypt Qaliûb” according to Crosskey 1966b: 108).

*Actia nigripes* Curran, 1927c: 6. Holotype male (AMNH). Type locality: D.R. Congo, Bas-Congo, Boma.

*Strobliomyia sororcula* Mesnil, 1954a: 16. Holotype female (MRAC). Type locality: D.R. Congo, Nord-Kivu, Rutshuru, 1285m.

***palaestina*** (Villeneuve, 1934).—Afrotropical: U.A. Emirates, Yemen. Palaearctic: C. Asia, M. East (all), N. Africa (NE. Africa). Oriental: ?Orien. China.

*Actia palaestina* Villeneuve, 1934a: 57. Holotype female (SMNS). Type locality: Israel, Rehovot [as “Rehoboth”].

*Actia alipes* Villeneuve, 1942b: 134. Holotype female (CNC). Type locality: Egypt, Aswân [as “Assuan”].

Note: The single record of *Peribaea palaestina* (Villeneuve, 1934) from China (Yunnan) by Chao et al. (1998: 2047) has been questioned by Tachi and Shima (2002: 141).

***pulla*** Mesnil, 1977.—Afrotropical: Madagascar.

*Peribaea pulla* Mesnil, 1977a: 82. Holotype male (MNHN). Type locality: Madagascar, Mahajanga, Ambato Boeni.

***repanda*** (Mesnil, 1954).—Afrotropical: D.R. Congo.

*Strobliomyia repanda* Mesnil, 1954a: 16. Holotype male (MRAC). Type locality: D.R. Congo, Nord-Kivu, near Rwindi, Ndeko, 1082m [ca. 0°50′S 29°19′E].

***rubea*** Mesnil, 1977.—Afrotropical: Madagascar.

*Peribaea rubea* Mesnil, 1977a: 82. Holotype female (MNHN). Type locality: Madagascar, Antsiranana, Montagne d’Ambre [Parc National, ca. 12°36′S 49°8′E].

***spoliata*** (Bezzi, 1923).—Afrotropical: Seychelles.

*Actia spoliata* Bezzi, 1923: 95. Syntypes, 1 male and 1 female (BMNH). Type localities: Seychelles, Mahé Is. (Cascade Estate, ca. 800ft) and Marie Anne Is.

***suspecta*** (Malloch, 1924).—Afrotropical: Sudan, Tanzania, Uganda. Oriental: India.

*Actia suspecta* Malloch, 1924: 409. Holotype male [not female as published, Crosskey 1976: 214] (BMNH). Type locality: India, Bihar, Pusa.

*Actia nana* Curran, 1928a: 237. Holotype female (BMNH). Type locality: Uganda, Kampala.

***tibialis*** (Robineau-Desvoidy, 1851).—Afrotropical: ?D.R. Congo, ?Kenya, ?South Africa. Palaearctic: C. Asia, Europe (W. Eur., E. Eur., SW. Eur., SC. Eur., SE. Eur.), Japan, “Korea”, M. East (Israel), Mongolia, Pal. China, Russia (W. Russia, S. Far East), Transcaucasia. Oriental: Myanmar, Orien. China, Ryukyu Is., Taiwan.

*Herbstia tibialis* Robineau-Desvoidy, 1851: 185. Type(s), male (lost, Herting 1974a: 19). Type locality: not given (France, probably near Paris).

Note: There is some doubt as to whether *Peribaea tibialis* (Robineau-Desvoidy, 1851) is correctly identified from the Afrotropical Region.

***timida*** (Mesnil, 1954).—Afrotropical: D.R. Congo.

*Strobliomyia timida* Mesnil, 1954a: 18. Holotype male (MRAC). Type locality: D.R. Congo, Nord-Kivu, Rutshuru, 1285m.

***ugandana*** (Curran, 1933).—Afrotropical: Uganda.

*Actia ugandana* Curran, 1933: 161. Holotype male (BMNH). Type locality: Uganda, Kampala.

***vidua*** (Mesnil, 1954).—Afrotropical: D.R. Congo.

*Strobliomyia vidua* Mesnil, 1954a: 15. Holotype male (MRAC). Type locality: D.R. Congo, Nord-Kivu, Rutshuru, Rivière Kanzarue, 1200m [ca. 1°13′S 29°28′E].

Undetermined spp.: Yemen, as “Peribaea sp. 1 cf. repanda Mesnil, 1954” and “*Peribaea* sp. 2” (Zeegers 2007: 415).

###### Genus *SIPHONA* Meigen, 1803

Note: The generic and subgeneric limits of *Siphona* Meigen, 1803 were revised and the Afrotropical species listed by O’Hara (1989).

####### Subgenus *APHANTORHAPHOPSIS* Townsend, 1926

*APHANTORHAPHOPSIS* Townsend, 1926c: 34. Type species: *Aphantorhaphopsis orientalis* Townsend, 1926, by original designation [Oriental].

*ASIPHONA* Mesnil, 1954a: 9 (as subgenus of *Siphona* Meigen, 1803). Type species: *Thryptocera selecta* Pandellé, 1894, by original designation [Palaearctic].

***fera*** Mesnil, 1954.—Afrotropical: D.R. Congo.

Siphona (Asiphona) fera Mesnil, 1954a: 26. Holotype male (MRAC). Type locality: D.R. Congo, Nord-Kivu, near Rutshuru, “Nyongera” [not located but apparently close to Rutshuru; also given as part of the locality was “(Butumba)”], 1218m.

***nigronitens*** Mesnil, 1954.—Afrotropical: D.R. Congo, Madagascar.

Siphona (Asiphona) nigronitens Mesnil, 1954a: 25 (as “*nigro-nitens*”). Holotype male (MRAC). Type locality: D.R. Congo, Nord-Kivu, Rutshuru, Rivière Musugereza, 1100m [ca. 1°05′S 29°27′E].

***picturata*** (Mesnil, 1977).—Afrotropical: Madagascar.

*Asiphona picturata* Mesnil, 1977c: 179. Holotype male (MNHN). Type locality: Madagascar, Antananarivo, Belazao [ca. 19°53′S 46°58′E].

***pudica*** Mesnil, 1954.—Afrotropical: D.R. Congo.

Siphona (Asiphona) pudica Mesnil, 1954a: 27. Holotype male (IRSNB). Type locality: D.R. Congo, Équateur, Eala.

***speciosa*** Mesnil, 1954.—Afrotropical: D.R. Congo, Tanzania.

Siphona (Asiphona) speciosa Mesnil, 1954a: 28. Holotype male (MRAC). Type locality: D.R. Congo, Nord-Kivu, Rutshuru, 1285m.

***xanthosoma*** Mesnil, 1954.—Afrotropical: D.R. Congo.

Siphona (Asiphona) xanthosoma Mesnil, 1954a: 28. Holotype male (MRAC). Type locality: D.R. Congo, Nord-Kivu, Rwindi, 1000m [ca. 0°47′S 29°17′E].

Undescribed sp: Yemen, as “Ceranthia (Aphantorhaphopsis) sp. 1” (Zeegers 2007: 406).

Note: Zeegers (2007: 406) noted that his “Ceranthia (Aphantorhaphopsis) sp. 1” has a clavate maxillary palpus. This suggests that this species belongs to *Siphona* (*Aphantorhaphopsis* Townsend, 1926) and not *Siphona* (*Ceranthia* Robineau-Desvoidy, 1830). Although *Ceranthia* has been ranked as either a distinct genus or as a subgenus of *Siphona* Meigen, 1803, it is generally regarded as a monophyletic taxon characterized in part by a reduced non-clavate maxillary palpus (O’Hara 1989, Andersen 1996, Tachi and Shima 2005, Cerretti 2010).

####### Subgenus *CERANTHIA* Robineau-Desvoidy, 1830

*CERANTHIA* Robineau-Desvoidy, 1830: 88. Type species: *Ceranthia fulvipes* Robineau-Desvoidy, 1830 (= *Ceromya abdominalis* Robineau-Desvoidy, 1830), by subsequent designation of Robineau-Desvoidy (1863a: 685) [Palaearctic].

***lacrymans*** (Mesnil, 1954).—Afrotropical: Rwanda, Tanzania.

*Ceranthia lacrymans* Mesnil, 1954a: 24. Holotype male (MRAC). Type locality: Rwanda, south of Volcan Karisimbi, Rivière Bikwi, 3100m [ca. 1°32′S 29°30′E].

Note: Mesnil (1954a: 24) did not name the country of the type locality of *Ceranthia lacrymans* but for other species collected from the same locality (Rivière Bikwi) gave “Congo Belge: Ruanda” or simply “Congo Belge”. Rwanda did not achieve complete independence from Belgium (and “Congo Belge”) until 1962. Crosskey (1980b: 852) and O’Hara (1989: 102, 1996: 144) erred in citing “Zaire” [= D.R. Congo] rather than Rwanda as the country containing the type locality of *Ceranthia lacrymans*.

***livoricolor*** (Mesnil, 1977).—Afrotropical: Madagascar.

*Ceranthia livoricolor* Mesnil, 1977c: 178. Holotype female (MNHN). Type locality: Madagascar, Fianarantsoa, Andringitra-Ambalavao area, Anjavidilava, 2020m [ca. 22°10′S 46°58′E, within Parc National d’Andringitra].

***plorans*** (Mesnil, 1954).—Afrotropical: Rwanda.

*Ceranthia plorans* Mesnil, 1954a: 24. Holotype male (MRAC). Type locality: Rwanda, Volcan Sabyinyo [as “Sabinyo”], Rwebeya Valley, 3000m [ca. 1°24′S 29°36′E].

***scutellata*** (Mesnil, 1954).—Afrotropical: D.R. Congo, Rwanda, Tanzania.

*Ceranthia scutellata* Mesnil, 1954a: 22. Holotype male (MRAC). Type locality: D.R. Congo, Nord-Kivu, Volcan Mikeno, near Rweru, 2400m [ca. 1°29′S 29°24′E].

***terrosa*** (Mesnil, 1954).—Afrotropical: Rwanda.

*Ceranthia terrosa* Mesnil, 1954a: 23. Holotype male (MRAC). Type locality: Rwanda, Volcans Gahinga–Sabyinyo [latter as “Sabinyo”], “Kundhuru ya Tshuve”, 2600m [ca. 1°23′S 29°38′E].

####### Subgenus *SIPHONA* Meigen, 1803

*CROCUTA* Meigen, 1800: 39. Name suppressed by ICZN (1963: 339).

***SIPHONA*** Meigen, 1803: 281. Type species: *Musca geniculata* De Geer, 1776, by designation under the Plenary Powers of ICZN (1974: 157) [Palaearctic].

***abbreviata*** (Villeneuve, 1915).—Afrotropical: Madagascar, South Africa.

*Bucentes abbreviata* Villeneuve, 1915b: 199. Syntypes, 1 male and 2 females (2 females from Madagascar in NHMW). Type localities: Madagascar and South Africa.

***albocincta*** (Villeneuve, 1942).—Afrotropical: D.R. Congo, Tanzania.

*Bucentes albocincta* Villeneuve, 1942a: 55. Holotype female (CNC). Type locality: D.R. Congo, Nord Kivu, Mt. Nyiragongo, 2300m.

***amoena*** (Mesnil, 1952).—Afrotropical: D.R. Congo, Rwanda.

*Crocuta amoena* Mesnil, 1952b: 12. Holotype male (MRAC). Type locality: Rwanda, south of Volcan Karisimbi, Rivière Bikwi, 3000m [ca. 1°32′S 29°30′E].

Note: Mesnil (1952b: 12) cited the country of the type locality of *Crocuta amoena* as “Congo Belge” but for other species collected from the same locality (Rivière Bikwi) gave the country as “Congo Belge: Ruanda”. Rwanda did not achieve complete independence from Belgium (and “Congo Belge”) until 1962. Crosskey (1980b: 854) and O’Hara (1989: 114, 1996: 132) misinterpreted Mesnil’s “Congo Belge” as meaning D.R. Congo (as “Zaire”).

***amplicornis*** Mesnil, 1959.—Afrotropical: Tanzania.

*Siphona amplicornis* Mesnil, 1959: 21. Holotype male (SMNS). Type locality: Tanzania, west side of Mt. Kibo [one of the three peaks of Mt. Kilimanjaro], 2800m.

***angusta*** Mesnil, 1959.—Afrotropical: Tanzania.

*Siphona angusta* Mesnil, 1959: 22. Holotype male (SMNS). Type locality: Tanzania, west side of Mt. Kibo [one of the three peaks of Mt. Kilimanjaro], 2800m.

***antennalis*** (Mesnil, 1952).—Afrotropical: Zimbabwe.

*Crocuta antennalis* Mesnil, 1952b: 9. Holotype male (CNC). Type locality: Zimbabwe, Harare [as “Salisbury”].

***atricapilla*** Mesnil, 1959.—Afrotropical: Tanzania.

*Siphona atricapilla* Mesnil, 1959: 20. Holotype male (SMNS). Type locality: Tanzania, west side of Mt. Kibo [one of the three peaks of Mt. Kilimanjaro], 3500m.

***bevisi*** Curran, 1941.—Afrotropical: South Africa.

*Siphona bevisi* Curran, 1941: 7. Holotype male (AMNH). Type locality: South Africa, KwaZulu-Natal, Durban, Umbilo.

***bilineata*** (Mesnil, 1952).—Afrotropical: D.R. Congo, Rwanda.

*Crocuta bilineata* Mesnil, 1952b: 10. Holotype male (MRAC). Type locality: Rwanda, foot of Volcan Karisimbi, Nyabirehe [as “Niabirehe”], 2400m [ca. 1°32′S 29°30′E].

***capensis*** Curran, 1941.—Afrotropical: South Africa.

*Siphona capensis* Curran, 1941: 7. Holotype female (SANC). Type locality: South Africa, Eastern Cape, East London.

***cothurnata*** (Mesnil, 1952).—Afrotropical: Cameroon, D.R. Congo, Kenya, Rwanda.

*Crocuta cothurnata* Mesnil, 1952b: 17. Holotype male (MRAC). Type locality: Rwanda, Volcan Muhabura [as “Muhavura”], Burambi, 2325m [ca. 1°22′S 29°42′E].

***creberrima*** (Speiser, 1910).—Afrotropical: Tanzania.

*Crocuta creberrima* Speiser, 1910: 142. Syntypes, 58 males and females (MSNM [2 “cotypes” according to Arnaud 1982: 12], NHRS). Type locality: Tanzania, Mt. Kilimanjaro [as “Kilimandjaro”], Kiboscho, 3000–4000m.

***cuthbertsoni*** Curran, 1941.—Afrotropical: D.R. Congo, Rwanda, South Africa, Tanzania, Zimbabwe.

*Siphona cuthbertsoni* Curran, 1941: 7. Holotype male (AMNH). Type locality: Zimbabwe, Harare [as “Salisbury”].

*Crocuta janssensi* Mesnil, 1952b: 4. Holotype male (MRAC). Type locality: Rwanda, Volcan Visoke [also known as Bisoke], Kibga, 2400m [ca. 1°29′S 29°31′E].

Note: Mesnil (1952b: 4) cited the country for the type locality of *Crocuta janssensi* as “Congo Belge: ... Ruanda”. Rwanda did not achieve complete independence from Belgium (and “Congo Belge”) until 1962. Crosskey (1980b: 854) misinterpreted the country as “Zaire” [= D.R. Congo].

***fuliginea*** Mesnil, 1977.

***fuligineacerina*** Mesnil, 1977.—Afrotropical: Madagascar.

*Siphona fuliginea cerina* Mesnil, 1977a: 76. Holotype male (MNHN). Type locality: Madagascar, Antsiranana, Montagne d’Ambre [Parc National, ca. 12°36′S 49°8′E].

***fuligineafuliginea*** Mesnil, 1977.—Afrotropical: Madagascar.

*Siphona fuliginea* Mesnil, 1977a: 77. Holotype male (MNHN). Type locality: Madagascar, Toliara, Ambatolahy [ca. 19°54′S 45°23′E].

***fuliginearubea*** Mesnil, 1977.—Afrotropical: Madagascar.

*Siphona fuliginea rubea* Mesnil, 1977a: 77. Holotype male (MNHN). Type locality: Madagascar, Antananarivo, Manjakatompo [ca. 19°21′S 47°18′E].

***gracilis*** (Mesnil, 1952).—Afrotropical: D.R. Congo, Kenya, Rwanda, South Africa, Tanzania.

*Crocuta gracilis* Mesnil, 1952b: 13. Holotype male (MRAC). Type locality: Rwanda, Volcan Visoke [also known as Bisoke], Kibga, 2400m [ca. 1°29′S 29°31′E].

***infuscata*** (Mesnil, 1952).—Afrotropical: D.R. Congo.

*Crocuta unispina infuscata* Mesnil, 1952b: 14. Holotype male (MRAC). Type locality: D.R. Congo, Nord-Kivu, Bweza, Tshamugussa, 2250m [ca. 1°20′S 29°31′E].

***lindneri*** Mesnil, 1959.—Afrotropical: Tanzania.

*Siphona lindneri* Mesnil, 1959: 22. Holotype male (SMNS). Type locality: Tanzania, Msingi [ca. 4°20′S 34°34′E].

***melania*** (Bezzi, 1908).—Afrotropical: Eritrea.

*Bucentes melania* Bezzi, 1908b: 58. Holotype female (not located, not among the labelled types of Bezzi in MSNM examined by Arnaud 1982). Type locality: Eritrea, near Adi Keyh [also as Adi Kaie and other spellings, published as “Adi Caiè”, ca. 14°51′N 39°22′E].

***melanura*** Mesnil, 1959.—Afrotropical: Tanzania.

*Siphona melanura* Mesnil, 1959: 23. Holotype female (SMNS). Type locality: Tanzania, west side of Mt. Kibo [one of the three peaks of Mt. Kilimanjaro], 3500m.

***munroi*** Curran, 1941.—Afrotropical: South Africa.

*Siphona munroi* Curran, 1941: 6. Holotype female (SANC). Type locality: South Africa, Eastern Cape, Fort Jackson.

***murina*** (Mesnil, 1952).—Afrotropical: Cameroon, D.R. Congo, Tanzania, Uganda.

*Crocuta murina* Mesnil, 1952b: 15. Holotype male (MRAC). Type locality: D.R. Congo, Nord-Kivu, near Rutshuru, “Nyongera” [not located but apparently close to Rutshuru; also given as part of the locality was “(Butumba)”], 1218m.

***nigrohalterata*** Mesnil, 1959.—Afrotropical: Tanzania

*Siphona amplicornis nigrohalterata* Mesnil, 1959: 22. Holotype male (SMNS). Type locality: Tanzania, west side of Mt. Kibo [one of the three peaks of Mt. Kilimanjaro], 3500m.

***nigroseta*** Curran, 1941.—Afrotropical: South Africa.

*Siphona nigroseta* Curran, 1941: 8. Holotype female (SANC). Type locality: South Africa, Gauteng, Pretoria.

***obesa*** (Mesnil, 1952).—Afrotropical: D.R. Congo.

*Crocuta obesa* Mesnil, 1952b: 8. Holotype male (MRAC). Type locality: D.R. Congo, Nord-Kivu, Rwindi, 1000m [ca. 0°47′S 29°17′E].

Note: The type locality is not in South Africa as listed by Crosskey (1980b: 854).

***obscuripennis*** Curran, 1941.—Afrotropical: Zimbabwe.

*Siphona obscuripennis* Curran, 1941: 8. Holotype female (AMNH). Type locality: Zimbabwe, Vumba Mountains.

***patellipalpis*** (Mesnil, 1952).—Afrotropical: D.R. Congo.

*Crocuta patellipalpis* Mesnil, 1952b: 10. Holotype male (MRAC). Type locality: D.R. Congo, Nord-Kivu, Mt. Sesero, near Bitashimwa [as “Bitashimva”], 2000m [ca. 1°23′S 29°26′E].

***phantasma*** (Mesnil, 1952).—Afrotropical: Rwanda, Uganda.

*Crocuta phantasma* Mesnil, 1952b: 7. Holotype male (MRAC). Type locality: Rwanda, summit of Volcan Gahinga, 3475m.

Note: The summit of Volcan Gahinga is on the border between Rwanda and Uganda.

***pigra*** Mesnil, 1977.—Afrotropical: Madagascar.

*Siphona pigra* Mesnil, 1977a: 78. Holotype female (MNHN). Type locality: Madagascar, Toamasina, Moramanga.

***pretoriana*** O’Hara & Cerretti, **nom. n.**—Afrotropical: South Africa.

*Siphona laticornis* Curran, 1941: 9 (junior secondary homonym of *Actia laticornis* Malloch, 1930). Holotype male (SANC). Type locality: South Africa, Gauteng, Pretoria.

Siphona (Siphona) pretoriana O’Hara & Cerretti, **nom. n.** for *Siphona laticornis* Curran, 1941.

Note: *Siphona laticornis* Curran, 1941 is a junior secondary homonym of *Actia laticornis* Malloch, 1930, the valid name of an Oriental species of Siphona (Aphantorhaphopsis) (O’Hara 1989: 96). We hereby propose the new name Siphona (Siphona) pretoriana to replace the preoccupied name *Siphona laticornis* Curran. The same type material applies to the new name. The specific epithet *pretoriana* is based on the type locality of Pretoria, South Africa.

***reducta*** (Mesnil, 1952).

***reductaludicra*** Mesnil, 1977.—Afrotropical: Madagascar.

*Siphona reducta ludicra* Mesnil, 1977a: 78. Holotype male (MNHN). Type locality: Madagascar, Antananarivo, Manjakatompo, 1700m [ca. 19°21′S 47°18′E].

***reductareducta*** (Mesnil, 1952).—Afrotropical: D.R. Congo, Rwanda, South Africa.

*Crocuta reducta* Mesnil, 1952b: 18. Holotype male (MRAC). Type locality: D.R. Congo, Nord-Kivu, Bweza, Tshamugussa, 2250m [ca. 1°20′S 29°31′E].

***rubrapex*** Mesnil, 1977.—Afrotropical: Madagascar.

*Siphona rubrapex* Mesnil, 1977a: 79. Holotype female (MNHN). Type locality: Madagascar, Toamasina, Périnet [ca. 18°55′S 48°25′E].

***rubrica*** (Mesnil, 1952).—Afrotropical: D.R. Congo.

*Crocuta rubrica* Mesnil, 1952b: 11. Holotype male (MRAC). Type locality: D.R. Congo, Nord-Kivu, Rutshuru, 1285m.

***setinerva*** (Mesnil, 1952).—Afrotropical: D.R. Congo, Madagascar, Rwanda.

*Crocuta setinerva* Mesnil, 1952b: 16. Holotype male (MRAC). Type locality: Rwanda, Volcan Visoke [also known as Bisoke], Kibga, 2400m [ca. 1°29′S 29°31′E].

***simulans*** (Mesnil, 1952).—Afrotropical: D.R. Congo, Madagascar, Rwanda.

*Crocuta simulans* Mesnil, 1952b: 18. Holotype male (MRAC). Type locality: Rwanda, Volcans Gahinga–Sabyinyo [latter as “Sabinyo”], “Kundhuru-ya-Tshuve”, 2600m [ca. 1°23′S 29°38′E].

***sola*** Mesnil, 1959.—Afrotropical: Tanzania.

*Siphona sola* Mesnil, 1959: 21. Holotype male (SMNS). Type locality: Tanzania, Pare Mountains, Usangi.

***spinulosa*** (Mesnil, 1952).—Afrotropical: D.R. Congo.

*Crocuta spinulosa* Mesnil, 1952b: 12. Holotype male (MRAC). Type locality: D.R. Congo, Nord-Kivu, Parc National des Virunga [as “Parc Nat. Albert”], Ngesho, 1000m.

***trichaeta*** (Mesnil, 1952).—Afrotropical: D.R. Congo, Rwanda.

*Crocuta trichaeta* Mesnil, 1952b: 18. Holotype, unspecified sex [male, see O’Hara 1996: 159] (MRAC). Type locality: Rwanda, foot of Volcan Karisimbi, Lac N’Gando, 2400m [ca. 1°35′S 29°24′E].

***unispina*** (Mesnil, 1952).—Afrotropical: D.R. Congo, Kenya.

*Crocuta unispina unispina* Mesnil, 1952b: 14. Holotype male (MRAC). Type locality: D.R. Congo, Nord-Kivu, Rutshuru, Rivière Kanzarue [as “riv. Kanzaru”], 1200m [ca. 1°13′S 29°28′E].

***vittata*** Curran, 1941.—Afrotropical: Zimbabwe.

*Siphona vittata* Curran, 1941: 8. Holotype male (AMNH). Type locality: Zimbabwe, Harare [as “Salisbury”].

***vixen*** Curran, 1941.—Afrotropical: South Africa, Zimbabwe.

*Siphona vixen* Curran, 1941: 9. Holotype female (AMNH). Type locality: Zimbabwe, Harare [as “Salisbury”].

***wittei*** (Mesnil, 1952).—Afrotropical: Kenya, Rwanda, South Africa.

*Crocuta wittei* Mesnil, 1952b: 5. Holotype male (MRAC). Type locality: Rwanda, [south of] Volcan Karisimbi, Rivière Bikwi, 3100m [ca. 1°32′S 29°30′E].

Undetermined sp. of *Siphona* (*Siphona* Meigen, 1803): Yemen (Zeegers 2007: 416).

###### Unplaced species of Siphonini

***heterochaeta*** Bezzi, 1908.—Afrotropical: Eritrea.

*Actia heterochaeta* Bezzi, 1908b: 59. Syntypes, females (not located, not among the labelled types of Bezzi in MSNM examined by Arnaud 1982). Type locality: Eritrea, near Adi Keyh [also as Adi Kaie and other spellings, published as “Adi Caiè”, ca. 14°51′N 39°22′E].

Note: Villeneuve (1913c: 35) recorded *Actia heterochaeta* Bezzi, 1908 from “Oshogbo” (Nigeria) based on two specimens in BMNH. Crosskey (1980b: 855), who had access to the BMNH material, must have doubted Villeneuve’s identification because he listed the species as an “Unplaced species of Siphonini” and gave Nigeria as a questionable record. Given the uncertainty of the identity of *Actia heterochaeta*, we record this species from only the country of the type locality.

##### Tribe TACHININI

###### Genus *CHROMATOPHANIA* Brauer & Bergenstamm, 1889

***CHROMATOPHANIA*** Brauer & Bergenstamm, 1889: 141 [also 1890: 73]. Type species: *Gonia picta* Wiedemann, 1830, by monotypy.

***distinguenda*** Villeneuve, 1913.—Afrotropical: Burundi, D.R. Congo, Malawi, Uganda.

*Chromatophania distinguenda* Villeneuve, 1913c: 43. Lectotype male (BMNH), by fixation of van Emden (1960: 478) (mention of “type (♂)” from Uganda in BMNH is regarded as a lectotype fixation). Type locality: Uganda (Unyoro District according to van Emden 1960: 478).

*Chromatophania dubia* Curran, 1941: 10. Holotype female (BMNH). Type locality: Malawi, Mt. Mulanje [as “Mt. Mlanje”].

***emdeni*** Mesnil, 1952.—Afrotropical: D.R. Congo.

*Chromatophania emdeni* Mesnil, 1952a: 7. Holotype male (IRSNB). Type locality: D.R. Congo, Équateur, Eala.

***fenestrata*** Villeneuve, 1913.—Afrotropical: “widespread W. Afr. & E. Afr.” (Crosskey 1980b: 849), including Angola, Cameroon, D.R. Congo, Ghana, Kenya, Malawi, Nigeria, Sierra Leone, Tanzania, Uganda, Zambia, Zimbabwe.

*Chromatophania fenestrata* Villeneuve, 1913c: 42. Syntypes, males and females (BMNH, MSNM [2 “cotypes” according to Arnaud 1982: 12], NHMW). Type localities: D.R. Congo (Kibimbi; Bas-Congo, Kibombo; Lufubu), Malawi, Nigeria, Sierra Leone, Uganda.

Note: Van Emden’s (1960: 479) notation of “Sierra Leone: Pendembu, 11.viii.12 (J.J. Simpson), 1♂ (1 type)” is a reference to a syntype and is not a lectotype fixation. More than one specimen of *Chromatophania fenestrata* was marked as “Typ.” by Villeneuve (see van Emden 1960: 427; there is also one ♂ in NHMW from Panguma, Sierra Leone marked as “typ.” by Villeneuve [examined by JEOH]).

***picta*** (Wiedemann, 1830).—Afrotropical: Botswana, D.R. Congo, Ethiopia, Ghana, Kenya, Malawi, Mozambique, Nigeria, South Africa, Uganda, Zimbabwe.

*Gonia picta* Wiedemann, 1830: 345. Lectotype female (ZMUC), by designation of Townsend (1932: 24, as “Female Ht”, “the male is hereby excluded as a very distinct form from *picta*, and the species restricted to the female”). Type locality: South Africa, Western Cape, Cape of Good Hope [as “Kap”].

*Chromatophania picta dilatata* Villeneuve, 1937b: 4. Type(s), unspecified sex (“elle ne semble pas très rare”) (not located). Type locality: not given.

***versicolor*** (Karsch, 1879).—Afrotropical: Angola, Kenya, Tanzania, Togo.

*Echinomyia versicolor* Karsch, 1879: 380. Holotype female (ZMHB). Type locality: Angola, [Cabinda Province], “Chinchoxo” [not located].

###### Genus *DEJEANIA* Robineau-Desvoidy, 1830

***DEJEANIA*** Robineau-Desvoidy, 1830: 33. Type species: *Dejeania capensis* Robineau-Desvoidy, 1830 (= *Stomoxys bombylans* Fabricius, 1798), by subsequent designation of Coquillett (1910: 531).

*MELANOJEANIA* Townsend, 1933: 465. Type species: *Dejeania pertristis* Villeneuve, 1913, by original designation.

*DEJAENIA*. Incorrect subsequent spelling of *Dejeania* Robineau-Desvoidy, 1830 (Verbeke 1962b: 62).

***bombylans*** (Fabricius, 1798).—Afrotropical: Angola, Cameroon, Congo, D.R. Congo, Eritrea, Ethiopia, Kenya, Malawi, Mozamibique, Sierra Leone, South Africa, Sudan, Tanzania, Uganda, Zambia, Zimbabwe.

*Stomoxys bombylans* Fabricius, 1798: 568. Type(s), unspecified sex (lost, Zimsen 1964: 485). Type locality: not given.

*Dejeania capensis* Robineau-Desvoidy, 1830: 34. Type(s), unspecified sex (MNHN or lost). Type locality: South Africa, Western Cape, Cape of Good Hope [as “cap de Bonne-Espérance”].

*Dejeania variabilis* Jaennicke, 1867: 393 [also 1868: 85]. Type(s), female (SMF). Type locality: Ethiopia, “Simen” (probably the Simien Mountains area).

*Dejeania gowdeyi* Curran, 1928a: 244. Holotype male (BMNH). Type locality: Uganda, Masaka [as “Majaba”].

***hecate*** Karsch, 1886b: 337.—Afrotropical: Angola, Cameroon, D.R. Congo, Ethiopia, Kenya, Malawi, South Africa, Sudan, Tanzania, Uganda, Zimbabwe.

*Dejeania hecate* Karsch, 1886b: 337. Holotype, unspecified sex [female, examined by JEOH] (ZMHB). Type locality: Angola, Pungo Andongo.

*Dejeania crocea* Bigot, 1888: 77. Lectotype female (BMNH), by designation of Crosskey (1971: 297). Type locality: South Africa, Western Cape, Cape of Good Hope [as “Cap de Bonne-Espérance”].

*Dejeania ebria* Brauer, 1898: 499 (as “*Dejeania ebria* Coll. Winth. manuscript in M.C. – (*Tachina ebria*) Cap. [Cape of Good Hope]”). *Nomen nudum*.

*Dejeania wollastonii* Austen, 1909: 93. Lectotype, unspecified sex (BMNH), by fixation of van Emden (1960: 475) (mention of “type” from Ruwenzori in BMNH is regarded as a lectotype fixation). Type locality: Uganda, east Rwenzori Range [as “E. Ruwenzori”], Mubuku Valley, 5000–13,000ft.

*Dejeania wollastoni abyssinica* Villeneuve, 1913c: 25. Syntypes, 2 females (BMNH). Type locality: southern Ethiopia.

*Dejeania marshalli* Curran, 1928a: 243. Holotype female (BMNH). Type locality: Uganda, Rwenzori Range [as “Mount Ruwenzori”].

*wollastoni*. Incorrect subsequent spelling of *wollastonii* Austen, 1909 (e.g., Villeneuve 1913c: 25, Curran 1928a: 244, van Emden 1960: 475).

***longirostris*** van Emden, 1960.—Afrotropical: Ethiopia.

*Dejeania longirostris* van Emden, 1960: 470. Holotype male (BMNH). Type locality: Ethiopia, Jem-Jem Forest [ca. 72km due west of Ādīs Ābeba], nearly 9000ft.

*nigrapex* Villeneuve, 1916.—Not Afrotropical, *nomen dubium* [?New World].

*Dejeania nigrapex* Villeneuve, 1916c: 470.

Note: Villeneuve (1916c: 470) described *Dejeania nigrapex* from one male and one female (NHMW, not examined) and gave the type locality as “Cape of Good Hope”, South Africa. Van Emden (1960: 468) speculated from the description alone that the species is actually a “New World fly”. Crosskey (1980b) did not list the species, presumably accepting van Emden’s conclusion that it is not of Afrotropical origin. The true identity of *Dejeania nigrapex* Villeneuve, 1916, and hence the probable provenance of the syntypes, has not been determined.

***pertristis*** Villeneuve, 1913.—Afrotropical: Cameroon, D.R. Congo, Nigeria, Togo, Uganda.

*Dejeania pertristis* Villeneuve, 1913c: 25. Lectotype male (BMNH), by fixation of van Emden (1960: 470) (mention of “♂ type” from Entebbe in BMNH is regarded as a lectotype fixation). Type locality: Uganda, Entebbe.

*Dejeania anthracosphaera* Speiser, 1914: 8. Syntypes, unspecified number and including at least 1 female (not located). Type localities: Cameroon (Mt. Cameroon, Buea) and Togo (Bismarckburg, ca. 8°11′N 0°41′E).

*Dejeania certima* Curran, 1927c: 20. Holotype male (AMNH). Type locality: D.R. Congo, Orientale, Kisangani [as “Stanleyville”].

*pertristris*. Incorrect subsequent spelling of *pertristis* Villeneuve, 1913 (van Emden 1960: 469).

###### Genus *PARATACHINA* Brauer & Bergenstamm, 1891

***PARATACHINA*** Brauer & Bergenstamm, 1891: 382 [also 1891: 78]. Type species: *Paratachina ingens* Brauer & Bergenstamm, 1891 (= *Echinomyia obliqua* Loew, 1863), by monotypy.

***costae*** (Jaennicke, 1867).—Afrotropical: Ethiopia.

*Echinomyia costae* Jaennicke, 1867: 389 [also 1868: 81]. Type(s), female (SMF). Type locality: Ethiopia, “Simen” (probably the Simien Mountains area).

***obliqua*** (Loew, 1863).—Afrotropical: South Africa.

*Echinomyia obliqua* Loew, 1863a: 16. Type(s), male (not located). Type locality: South Africa, Free State, Bloemfontein.

Note: Townsend’s (1939a: 245) mention of “Ht” from Bloemfontein in ZMHB is not accepted as a lectotype fixation because there is no evidence that the name-bearing types of species described in Loew (1863a) were deposited in ZMHB. No name-bearing type of *Echinomyia obliqua* Loew, 1863 was found in ZMHB by JEOH.

*Paratachina ingens* Brauer & Bergenstamm, 1891: 382 [also 1891: 78] (as “*Paratachina ingens* Wd. Coll. Winth. litt.”). Lectotype male (NHMW), by fixation of Townsend (1939a: 245) (mention of “Ht male” from Cape of Good Hope in NHMW is regarded as a lectotype fixation for the only male of the two syntypes in NHMW [examined by JEOH]). Type locality: South Africa, Western Cape, Cape of Good Hope [as “Cap b. sp.” = “Cap Bonae Spei”].

Undescribed sp.: Madagascar (MRAC, examined by PC).

###### Genus *PELETERIA* Robineau-Desvoidy, 1830

***PELETERIA*** Robineau-Desvoidy, 1830: 39. Type species: *Peleteria abdominalis* Robineau-Desvoidy, 1830, by subsequent designation of Coquillett (1910: 586) [Palaearctic].

*CUPHOCERA* Macquart, 1845: 267. Type species: *Micropalpus ruficornis* Macquart, 1835, by original designation [Palaearctic].

*PELETIERIA* Bezzi, 1906: 54. Unjustified emendation of *Peleteria* Robineau-Desvoidy, 1830.

*ACUPHOCERA* Townsend, 1926c: 37. Type species: *Acuphocera sumatrensis* Townsend, 1926 (= *Tachina iavana* Wiedemann, 1819), by original designation.

*PLEROPELETERIA* Villeneuve, 1916c: 470 (as subgenus of *Dejeania* Robineau-Desvoidy, 1830). Type species: Dejeania (Pleropeleteria) peringueyi Villeneuve, 1916 (= *Tachina lithanthrax* Wiedemann, 1830), by monotypy.

*PELETIERIANA* Mesnil, 1970a: 951 (as subgenus of *Peleteria* Robineau-Desvoidy, 1830, as “*Peletieria*”). Type species: *Echinomyia rustica* Karsch, 1886, by original designation.

*CYPHOCERA*. Incorrect subsequent spelling of *Cuphocera* Macquart, 1845 (Rondani *in* Osculati 1850: 241, Rondani 1856: 63, 207, Rondani 1859: 60, 235, Villeneuve 1915b: 191).

Note: Subgenera of *Peleteria* Robineau-Desvoidy, 1830 are not recognized here because the subgeneric placements of the Afrotropical species require more study.

***iavana*** (Wiedemann, 1819).—Afrotropical: D.R. Congo, Ethiopia, Kenya, Madagascar, South Africa, Sudan, Tanzania, Zambia, Zimbabwe. Palaearctic: Europe (all except British Is., Scand.), Japan, Kazakhstan, “Korea”, M. East (all), N. Africa (NW. Africa), Pal. China, Russia (W. Russia, W. Siberia, E. Siberia, S. Far East), Transcaucasia. Oriental: India, Indonesia, Malaysia, Myanmar, Orien. China, Nepal, Philippines, Sri Lanka, Taiwan. Thailand. Australasian: Australia, Melanesia, N. Australasian.

*Musca varia* Fabricius, 1794: 327 (junior primary homonym of *Musca varia* Gmelin, 1790). Type(s), unspecified sex (probably lost [the single female in ZMUC was considered the “Female Ht” by Townsend (1932: 42) and the “Holotype ♀” by Crosskey (1973b: 134, 1976: 205) but see note in O’Hara et al. 2009: 171]). Type locality: “India orientali” (i.e., “East Indies”).

*Tachina iavana* Wiedemann, 1819: 24. Lectotype female (ZMUC), by designation of Crosskey (1966a: 673). Type locality: Indonesia, Jawa (Jakarta [as “Batavia”] according to Crosskey 1966a: 673).

*Echinomyia argyrocephala* Macquart, 1846: 272 [also 1846: 144]. Type(s), female (not located). Type locality: Algeria, Alger.

*Cuphocera rufiventris* Corti, 1895: 136. Type(s), female (?MCSN). Type locality: Ethiopia, Jubba River, “Cormoso” [not located].

*Cyphocera varia hova* Villeneuve, 1915b: 191. Syntypes, unspecified number and sex (not located). Type locality: Madagascar.

*Acuphocera sumatrensis* Townsend, 1926c: 37. Lectotype male (RMNH), by designation of Crosskey (1969: 90). Type locality: Indonesia, Sumatera, Bukittinggi [as “Fort de Kock”] 920m.

*Cuphocera javana* Crosskey, 1976: 205 (published in synonymy with *Cuphocera varia* (Fabricius, 1794)). Unjustified emendation of *Tachina iavana* Wiedemann, 1819.

*javana*. Incorrect subsequent spelling of *iavana* Wiedemann, 1819 (Wiedemann 1830: 288, many other works).

Note: Wiedemann (1819, 1824) described several species of Diptera with the specific epithet *iavana* or *iavanus* (see index in Evenhuis 1989a) and therefore this spelling used in the name *Tachina iavana* Wiedemann, 1819 was not a printer’s error. In a subsequent work, Wiedemann (1830) changed the spelling to *javana* in the text (p. 288) but not in the index (p. 679). Since Wiedemann (1830) used both spellings, we interpret the spelling *javana* therein as an incorrect subsequent spelling and not as an emendation (following Evenhuis and Greathead 1999: 544, not O’Hara et al. 2009: 171). Crosskey (1976: 205) cited both spellings and adopted *javana* as the proper spelling, thus creating an unjustified emendation according to Article 33.2.1 of the *Code* (ICZN 1999). An earlier unjustified emendation with this spelling may exist but our cursory search of the literature did not reveal one. Cantrell and Crosskey (1989: 761) reestablished *iavana* as the correct spelling and cited usage of *javana* as an “error for *iavana*”.

***lithanthrax*** (Wiedemann, 1830).—Afrotropical: South Africa.

*Tachina lithanthrax* Wiedemann, 1830: 283. Lectotype, unspecified sex (ZMUC), by fixation of van Emden (1960: 482) (examination of “type” from Cape of Good Hope in ZMUC is regarded as a lectotype fixation). Type locality: South Africa, Western Cape, Cape of Good Hope [as “Java”, in error according to van Emden 1960: 482].

Dejeania (Pleropeleteria) peringueyi Villeneuve, 1916c: 471. Holotype male (CNC). Type locality: South Africa, Western Cape, Cape Town.

*litanthrax*. Incorrect subsequent spelling of *lithanthrax* Wiedemann, 1830 (original usage not found but spelling listed by Crosskey 1980b: 850).

***longipalpis*** van Emden, 1960.—Afrotropical: Ethiopia.

*Peletieria longipalpis* van Emden, 1960: 483. Holotype female (BMNH). Type locality: Ethiopia, Mt. Zuqualla [as “Mt. Zuquala”], ca. 9000ft.

***mimica*** Villeneuve, 1913.—Afrotropical: D.R. Congo.

*Peleteria mimica* Villeneuve, 1913c: 26. Holotype male (CNC). Type locality: D.R. Congo, Katanga, Sankisia.

***ruficornis*** (Macquart, 1835).—Afrotropical: Angola, D.R. Congo, Ethiopia, Kenya, Madagascar, Malawi, Nigeria, Sierra Leone, South Africa, Tanzania, Uganda, U.A. Emirates, Yemen, Zimbabwe. Palaearctic: Europe (all except British Is., Turkey), Kazakhstan, M. East (Israel), N. Africa (Canary Is.), Russia (W. Russia).

*Micropalpus ruficornis* Macquart, 1835: 83. Type(s), unspecified sex (not located). Type locality: France, Bordeaux.

***rustica*** (Karsch, 1886).—Afrotropical: Angola, D.R. Congo, Namibia, Sierra Leone, South Africa, Zambia, Zimbabwe.

Echinomyia (Peleteria) rustica Karsch, 1886b: 338. Syntypes, 2 males and 1 female (1 female in ZMHB). Type locality: Angola, Pungo Andongo.

Undescribed sp.: Madagascar (TAU, examined by PC).

###### Genus *PLATYSCHINERIA* Villeneuve, 1942

***PLATYSCHINERIA*** Villeneuve, 1942a: 51. Type species: *Platyschineria cuthbertsoni* Villeneuve, 1942, by monotypy.

Note: *Platyschineria* Villeneuve, 1942 was treated as an unplaced genus of Tachinidae by Crosskey (1980b: 881) but was placed in Tachinini by Crosskey (1984: 200, 258, 260).

***cuthbertsoni*** Villeneuve, 1942.—Afrotropical: Kenya (**new record**, MZUR [PC]), South Africa, Tanzania, Zimbabwe.

*Platyschineria cuthbertsoni* Villeneuve, 1942a: 52. Holotype male (NMBZ). Type locality: Zimbabwe, Khami.

#### Unplaced species of Tachinidae

***calyptrata*** Zeegers, 2007.—Afrotropical: Yemen.

*Mesnilomyia calyptrata* Zeegers, 2007: 410. Holotype female (RMNH). Type locality: Yemen, 12km northwest of Manakhah (15°04′19″N 43°44′27″E).

Note: *Mesnilomyia calyptrata* Zeegers, 2007 was removed from *Mesnilomyia* Kugler, 1972 (now a synonym of *Rossimyiops* Mesnil, 1953) and left unplaced in Tachinidae by Cerretti et al. (2009: 53).

***dejeanii*** Robineau-Desvoidy, 1830.—Afrotropical: Mauritius.

*Dexia dejeanii* Robineau-Desvoidy, 1830: 312. Type(s), unspecified sex (originally in Dejean’s collection, the Diptera of which are mostly lost; Evenhuis et al. 2010: 238). Type locality: Mauritius [as “l’Ile de France”].

***imbuta*** Walker, 1853.—Afrotropical: South Africa.

*Tachina imbuta* Walker, 1853: 288. Type(s), male (BMNH). Type locality: South Africa, Western Cape, Cape of Good Hope [as “Cape”].

Note: Crosskey (1980b: 882) was in error in treating *Tachina imbuta* Walker, 1853 as a junior primary homonym of *Tachina imbuta* Wiedemann, 1830. *Tachina imbuta* of Wiedemann (1830: 302) was a redescription and new combination of *Ocyptera imbuta* Wiedemann, 1819 (from India) and not a description of a new species.

***marginella*** Wiedemann, 1830.—Afrotropical: Sudan.

*Tachina marginella* Wiedemann, 1830: 330. Type(s), female (SMF or lost). Type locality: Nubia region [as “Nubien”, a region in southern Egypt and northern Sudan, recorded here as Sudan following Crosskey 1980b: 882].

***media*** Meunier, 1905.—Afrotropical: Tanzania.

*Thryptocera media* Meunier, 1905b: 212. Holotype, unspecified sex (not located). Type locality: Tanzania, Zanzibar (in copal).

***multiciliata*** Meunier, 1905.—Afrotropical: Madagascar.

*Myobia multiciliata* Meunier, 1905a: 91. Holotype, unspecified sex (not located). Type locality: Madagascar (in copal).

### Lectotype designations

In the interests of nomenclatural stability, we have chosen to designate lectotypes for the nominal species below to fix their names to single specimens that we believe best represent the taxa described.

Label information is cited in a consistent matter. The exact wording and punctuation are given for each label, where recorded, with the data from each line separated by a diagonal slash and a space (/ ). Data from each label is enclosed in quotation marks. Additional information not appearing on a label is enclosed within square brackets after the quotation marks. Words are typed unless indicated otherwise. A semi-colon marks the end of a label.

#### *Degeeria crocea* Villeneuve, 1950: 3.

Described from two specimens, a male from Mt. Mulanje, Malawi [as “Nyasaland, Mt. Mlanje”] and a female from Molo, Kenya. The two syntypes are in IRSNB. They are conspecific and in good condition, and labelled as follows:

1. ♂: “Mt Mlanje,/ Nyasaland,/ 23.VIII 1913./ S. A. Neave.” [date, month and last number of year handprinted]; “Degeeria/ crocea/ Typ. Villen.” [Villeneuve’s handwritten det. label]; “TYPE” [red label with black lines around “TYPE”].

2. ♀: “AFR. OR. ANGL. (MAU-ESCARP^T^)/ MOLO/ ALLUAUD & JEANNEL/ Déc. 1911 - 2420^m^ - St. 19”; “♀”; “Degeeria” [handwritten by Villeneuve].

In the interests of nomenclatural stability and to restrict the name to a single specimen, the male syntype from Mt. Mulanje and labelled by Villeneuve as “Typ.” is hereby designated by PC as lectotype of *Degeeria crocea* Villeneuve, 1950. The lectotype has been provided with the following additional label: “Lectotype ♂/ Degeeria/ crocea Villeneuve,/ 1950/ P. Cerretti des./ 2014” [handprinted by PC].

The current combination for this species is *Medina crocea* (Villeneuve, 1950).

#### *Degeeria semirufa* Villeneuve, 1950: 6.

Described from two females from Mt. Mulanje, Malawi [as “Nyasaland, Mt. Mlanje”]. Villeneuve (1950: 6) cited only the date “29-IX” in his description but the two females in IRSNB, one collected on 29.IX.1913 and the other on 16.X.1913 (and labelled “Typ.”), are believed to be the two original syntypes. They are conspecific and in good condition, and labelled as follows:

1. ♀: “Mt. Mlanje,/ Nyasaland,/ 16.X.1913./ S. A. Neave.” [day and month handprinted]; “Degeeria/ semirufa/ Typ. ♀” [Villeneuve’s handwritten det. label]; “TYPE” [red label with black lines around “TYPE”].

2. ♀: “Mt. Mlanje,/ Nyasaland,/ 29.IX.1913./ S. A. Neave.” [day and month handprinted].

In the interests of nomenclatural stability and to restrict the name to a single specimen, the female syntype collected on 16.X.1913 and labelled by Villeneuve as “Typ.” is hereby designated by PC as lectotype of *Degeeria semirufa* Villeneuve, 1950. The lectotype has been provided with the following additional label: “Lectotype ♀/ Degeeria/ semirufa Villeneuve,/ 1950/ P. Cerretti des./ 2014” [handprinted by PC].

The current combination for this species is *Medina semirufa* (Villeneuve, 1950).

#### *Erycia brunnescens* Villeneuve, 1934d: 69.

Described from three females from the Rwenzori Range [as “Ruwenzori”] in Uganda, between 2300m and 3000m. Two of the three syntypes are in IRSNB; the third syntype has not been located. The syntypes in IRSNB are conspecific and in good condition, and labelled as follows:

1. ♀: “R 2500^m^/ 18.V.14” [handprinted]; “TYPE” [red label]; “Erycia/ Dr Villeneuve det./ brunnescens/ Typ. Villen.” [Villeneuve’s det. label, handwritten except for 2nd line]; “Thelairosoma/ brunnescens Villen./ L. Mesnil det. 1953” [Mesnil’s det. label, 1st and 2nd lines and “53” in last line handwritten].

2. ♀: “R 3000^m^ / 15.IV.14”; “Thelairosoma/ brunnescens Villen./ L. Mesnil det. 1953” [Mesnil’s det. label, 1st and 2nd lines and “53” in last line handwritten].

In the interests of nomenclatural stability and to restrict the name to a single specimen, the female syntype collected at 2500m and labelled by Villeneuve as “Typ.” is hereby designated by PC as lectotype of *Erycia brunnescens* Villeneuve, 1934. The lectotype has been provided with the following additional label: “Lectotype ♀/ *Erycia*/ *brunnescens* Villeneuve,/ 1934/ P. Cerretti des. 2014” [handprinted by PC].

The current combination for this species is *Thelairosoma brunnescens* (Villeneuve, 1934).

#### *Exorista oculata* Villeneuve, 1910a: 251.

Described from one or more males, with no locality other than “Congo” [= D.R. Congo], which is given in the first paragraph of the paper. There is a single male in IRSNB that is either the holotype or a syntype. We follow Recommendation 73F of the *Code* (ICZN 1999, “Avoidance of assumption of holotype”) in treating this specimen as a syntype of a hypothetically larger type series. Villeneuve frequently labelled more than one specimen as a type and therefore his “Typus” label on the male in IRSNB is no indication that this was the only specimen of the type series. The syntype is in good condition and labelled as follows:

1. ♂: “Coll. J. Villeneuve:/ Exorista/ oculata Vill./ R.M.H.N. Belg. 15.392” [2nd and 3rd lines handprinted]; “TYPE” [red label]; “Typus” [handwritten by Villeneuve on blue paper]; “Exorista/ oculata/ ♂ n. sp.” [Villeneuve’s handwritten det. label]; “1 Soie aux/ coxae post.” [handwritten].

In the interests of nomenclatural stability and to restrict the name to a single specimen, the single recognized syntype in IRSNB is hereby designated by PC as lectotype of *Exorista oculata* Villeneuve, 1910. The lectotype has been provided with the following additional label: “Lectotype ♂/ Exorista/ oculata Villeneuve,/ 1910/ P. Cerretti des. 2014” [handprinted by PC].

The current combination for this species is Carcelia (Carcelita) oculata (Villeneuve, 1910).

#### *Kiniatilla tricincta* Villeneuve, 1938c: 11.

Described from multiple females (of an unspecified number) from two localities in D.R. Congo: Kiniati in the Mayumbé area [a highland area west of Rivière Congo] of Bas-Congo, and “Beni à Lesse” [Lesse is located northeast of Beni at ca. 0°45′N 29°46′E] in Nord-Kivu. There are three conspecific specimens that we believe to be syntypes in IRSNB, one female and two males. Although Villeneuve did not mention males, the label data of the males in IRSNB match the published data and thus we assume Villeneuve erred in citing only females. The three syntypes are conspecific and in fair condition, and labelled as follows:

1. ♀: “MUSÉE DU CONGO/ Mayumbé : Kiniati/ 7-VI-1911/ R. Mayné”; “Kiniatilla/ tricincta/ n. sp. Villen.” [Villeneuve’s handwritten det. label]; “TYPE” [red label with black lines around “TYPE”].

2. ♂: “Musée du Congo/ Beni à Lesse/ fin VII-1911/ Dr. Murtula”.

3. ♂: “Musée du Congo/ Beni à Lesse/ fin VII-1911/ Dr. Murtula”; “Kiniatilla/ tricincta/ Villen.”; “Para-/ type”.

In the interests of nomenclatural stability and to restrict the name to a single specimen, the female syntype from Kiniati is hereby designated by PC as lectotype of *Kiniatilla tricincta* Villeneuve, 1938. The lectotype has been provided with the following additional label: “Lectotype ♀/ Kiniatilla/ tricincta Villeneuve,/ 1938/ P. Cerretti des./ 2014” [handprinted by PC].

The current combination for this species is *Kiniatilla tricincta* Villeneuve, 1938.

#### *Myxarchiclops caffer* Villeneuve, 1916c: 495.

Described from an unspecified number of males and females from South Africa from three localities: Cape Town (Western Cape; collected by L. Péringuey); “S. Western District” (Western Cape); and Mooi River (KwaZulu-Natal; collected by C. Wroughton). The depository for specimens from the first two localities was given as “S Afric. Museum”, which is now SAMC. Nine specimens from these localities that are believed to be syntypes have been located in CNC and SAMC and examined by JEOH. The depository for specimens from the last locality was given as “Entom. Res. Comm.” and should now be in BMNH (not examined).

There are five probable syntypes in SAMC, four males from Cape Town collected by Péringuey and one male from the locality given as “S. Western District” (bearing locality labels “S.W. Distr.” and “Cape Col.” and a Villeneuve det. label including “Typ.”). Of the four males from Cape Town, three were collected in 1913 and one in 1915; the last bears a Villeneuve det. label but not a “Typ.” inscription. Townsend (1941: 193) mentioned “Ht male” from Cape Town in SAMC but this statement was insufficient for a lectotype fixation because a single male was not selected from among the four males in SAMC from this locality.

There are four specimens in CNC collected from Cape Town by Péringuey: two males and one female collected in September 1913 and one male collected in 1915. Two of the specimens (one male and one female) from 1913 are double mounted on the same pin and bear a Villeneuve identification and type label. It is likely that Villeneuve had all four specimens at hand when he described *Myxarchiclops caffer* and therefore all four are treated here as syntypes (Cooper and O’Hara 1996: 54 recognized only the male and female on the same pin as syntypes). The four syntypes in CNC are conspecific and in good condition, and labelled as follows:

1. ♂: “♂ ♀” [handprinted on stiff card into which are inserted two small pins holding the male and female]; “Cape Town/ G. Peringuey/ Sep 1913” [‘Sep’ handwritten]; “Myxarchiclops/ caffer/ Typ. Villen./ Typ.” [handwritten]; “Myxarchiclops/ caffer Villen./ L.P. Mesnil det., 1969” [first two lines and ‘69’ handwritten]; “TYPE” [red label]”; “CNC Syntype/ Myxarchiclops caffer/ Villeneuve/ Label affixed 1994”; “EX/ L.-P. MESNIL/ COLLECTION 1970”.

2. ♀: Double-mounted with male (see labels above).

3. ♂ [double-mounted on stiff card]: “Cape Town/ G. Peringuey/ Sep 1913” [‘Sep’ handwritten]; “Myxarchiclops/ caffer Villen./ L.P. Mesnil det., 1969” [first two lines and ‘69’ handwritten]; “EX/ L.-P. MESNIL/ COLLECTION 1970”.

4. ♂ [double-mounted on foam]: “Cape Town/ Peringuey/ 1915”; “Myxarchiclops/ caffer Villen./ L.P. Mesnil det., 1969” [first two lines and ‘69’ handwritten]; “EX/ L.-P. MESNIL/ COLLECTION 1970”.

In the interests of nomenclatural stability and to restrict the name to a single specimen, the male syntype on the double mount in CNC is hereby designated by JEOH as lectotype of *Myxarchiclops caffer* Villeneuve, 1916. The lectotype has been provided with the following additional label: “LECTOTYPE/ Myxarchiclops/ caffer Villeneuve/ J.E. O’Hara/ designation 2015” [red label]. The remaining three syntypes in CNC have been labelled as paralectotypes. We have not labelled the paralectotypes in SAMC or examined the paralectotype(s) from Mooi River in BMNH.

The current combination for this species is *Myxarchiclops caffer* Villeneuve, 1916.

#### *Ocyptera linearis* Villeneuve, 1936b: 2.

Described from one or more specimens of unspecified sex from D.R. Congo. There is a single male in IRSNB that we believe to be the holotype or a syntype. It is not labelled as a type but bears a Villeneuve det. label reading “*Ocyptera linearis* Villen.”. We follow Recommendation 73F of the *Code* (ICZN 1999, “Avoidance of assumption of holotype”) in treating this specimen as a syntype of a hypothetically larger type series. The syntype is in good condition and labelled as follows:

1. ♂: “Congo-belge/ Eala-XI-1934/ J. Ghesquière” [only month handprinted]; “R. Mus. Hist. Nat./ Belg. 10482”; “Dr. J. Villeneuve det., 1936 :/ Ocyptera/ linearis Villen” [2nd and 3rd lines handprinted]; “Ocyptera/ linearis/ Villen.” [Villeneuve’s handwritten det. label].

In the interests of nomenclatural stability and to restrict the name to a single specimen, the single recognized syntype in IRSNB is hereby designated by PC as lectotype of *Ocyptera linearis* Villeneuve, 1936. The lectotype has been provided with the following additional label: “Lectotype ♂/ *Ocyptera*/ *linearis* Villeneuve,/ 1936/ P. Cerretti des./ 2014” [handprinted by PC].

This nominal species is currently a junior subjective synonym of *Cylindromyia soror* (Wiedemann, 1830).

#### *Peristasisea luteola* Villeneuve, 1934b: 187.

Described from one male and two females from Malawi (as “Nyasaland”). The only syntype located, the single male, is in IRSNB. It is in good condition and labelled as follows:

1. ♂: “Nyasaland/ 2.X.” [handprinted]; “TYPE” [red label]; “Coll. J. Villeneuve./ Peristasisea/ luteola ♂ Villen./ R.M.H.N.Belg. 15.392/ Typ.” [2nd and 3rd lines handwritten, ‘Typ.’ handwritten along left side of label]; “Peristasisea/ luteola ♂/ Typ. Villen.” [Villeneuve’s handwritten det. label].

In the interests of nomenclatural stability and to restrict the name to a single specimen, the single recognized syntype in IRSNB is hereby designated by PC as lectotype of *Peristasisea luteola* Villeneuve, 1934. The lectotype has been provided with the following additional label: “Lectotype ♂/ *Peristasisea*/ *luteola* Villeneuve,/ 1934/ P. Cerretti des./ 2014”.

The current combination for this species is *Peristasisea luteola* Villeneuve, 1934.

#### *Phorocera crassipalpis* Villeneuve, 1938c: 2.

Described from one male and one or more females from Bomputu in D.R. Congo. Two syntypes have been located, a male in MRAC and a female in CNC. The two syntypes are conspecific. The male syntype is in good condition except for badly damaged wings; the female syntype is in fair condition, missing both wings and numerous setae on dorsum of thorax and abdomen. The syntypes are labelled as follows:

1. ♂: “Coll. Mus. Congo/ Bomputu/ XII.1935/ J. Ghesquière”; “*Phorocera*/ *crassipalpis*/ typ. ♂ Villen.”; “HOLOTYPUS/ ♂”; “[QRcode] RMCA ENT/ 000012116” (MRAC).

2. ♀: “Congo-belge/ Bomputu-XII-1935/ J. Ghesquière/ 1075” [2nd and 4th lines handprinted]; “Parasite/ Lepido 1048” [handprinted]; “R. Mus. Hist. Nat./ Belg. 10482”; “Phorocera/ crassipalpis/ typ. ♀ Villen.” [Villeneuve’s handwritten det. label]; “P. crassipalpis/ Villen./ L.P. Mesnil det., 1969” [1st and 2nd lines and ‘69’ handwritten]; “CNC Syntype/ Phorocera crassipalpis/ Villeneuve/ Label affixed 1994” [yellow label]; “EX/ L.-P. MESNIL/ COLLECTION 1970” (CNC).

In the interests of nomenclatural stability and to restrict the name to a single specimen, the male syntype in MRAC is hereby designated by PC as lectotype of *Phorocera crassipalpis* Villeneuve, 1938. The lectotype has been provided with the following additional labels: “Lectotype ♂/ *Phorocera*/ *crassipalpis* Villeneuve 1938/ Cerretti des. 2014”; “*Carceliathrix*/ *crassipalpis* (Villeneuve, 1938)/ Cerretti det. 2014” [handprinted by PC].

*Phorocera crassipalpis* is designated as the type species of new genus *Carceliathrix* Cerretti & O’Hara, described below.

### New taxa of Afrotropical Tachinidae

Seven new genera are described below to accommodate five described and eight new species that do not fit within the generic concepts of other Tachinidae. They are described here to allow the catalogue above to more accurately reflect the known generic diversity of Afrotropical Tachinidae. A key to identify these genera within the broader context of all Afrotropical genera of Tachinidae will appear in the upcoming *Manual of Afrotropical Diptera* (as discussed in the Introduction). Morphological terms follow Cumming and Wood (in press) except vfor costal sections of the wing, which follow Cerretti (2010: 11, vol. 2). Photographic techniques were the same as described in Cerretti et al. (2015).

#### Dexiinae, Dexiini

##### Mesnilotrix

Taxon classificationAnimaliaDipteraTachinidae

Cerretti & O’Hara gen. n.

http://zoobank.org/004A6353-E074-4CA0-BC3E-953DE56816C0

Fig. 7

###### Type species.

*Dexiotrix empiformis* Mesnil, 1976, by present designation.

###### Etymology.

*Mesnilotrix* is a composite word formed from the surname of Louis Paul Mesnil (the author of the type species) and the suffix of the generic name *Dexiotrix* Villeneuve. The name alludes to the morphological external similarity of *empiformis* to members of *Dexiotrix* that led Mesnil to describe the species in *Dexiotrix*.

###### Diagnosis.

**Figure 7.**
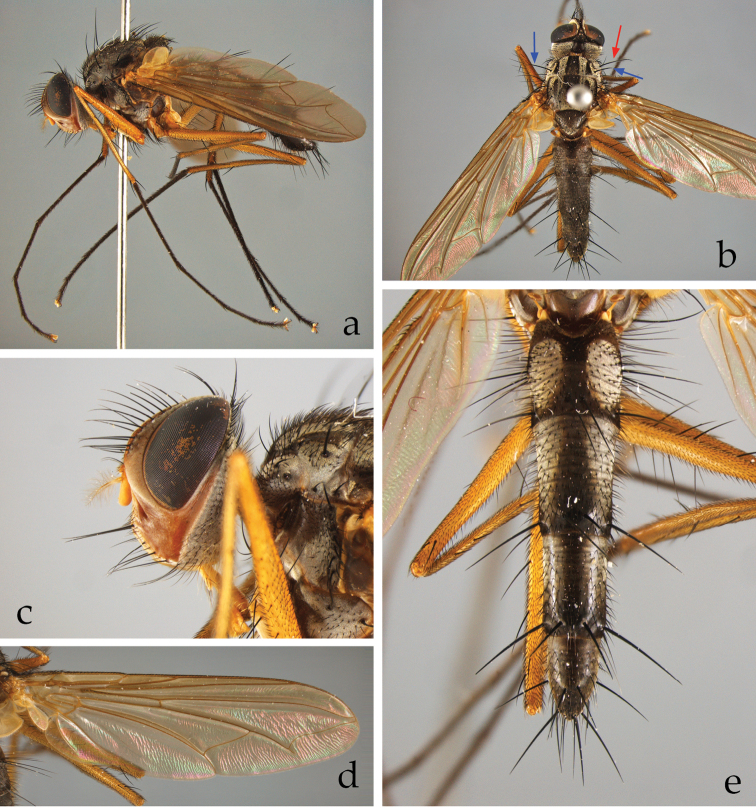
*Mesnilotrix empiformis* (Mesnil) (male holotype, MNHN) **a** habitus in lateral view **b** habitus in dorsal view (red arrow indicates lateral postpronotal seta, blue arrows indicate presutural supra-alar seta) **c** head in lateral view **d** wing **e** abdomen in dorsal view.

Compound eye bare. Antenna at most as long as height of gena. Arista plumose, with total width of arista and microtrichia 3.0–3.7 times as wide as width of postpedicel. Frontal setae descending to above level of upper margin of scape. Parafacial bare, about 2 times as wide as width of postpedicel. Facial ridge slightly concave, with fine decumbent setulae on lower 1/4 (or slightly more). Vibrissa arising distinctly above level of lower facial margin; subvibrissal ridge well developed with a row of 3–5 setae. Face concave with a small, narrow carina, not dividing antennae and not visible in laterial view. Genal dilation not developed. Upper occiput with several long black setulae not arranged in rows. Gena about 0.5 times as high as compound eye. Prementum about 2 times as long as wide. Palpus short, 2/3–3/4 as long as prementum, cylindrical (i.e., not inflated apically), with several long black setulae on apical 1/2. Proepisternum and prosternum bare. Postpronotum with 2–3 setae (if 3, then arranged in a line); lateral postpronotal seta enormously developed (Fig. 7b, red arrow). Scutum with 0 + 0–1 (i.e., 0 presutural and 0–1 postsutural) acrostichal setae; 3 + 3 dorsocentral setae; 0 + 2–3 intra-alar setae; 1 + 3 supra-alar setae (presutural supra-alar seta enormously developed [Fig. 7b, blue arrows], first postsutural supra-alar shorter than notopleural setae); 1 posthumeral seta; 2 notopleural setae. Two katepisternal setae. Scutellum with 3 pairs of marginal setae: one pair of crossed, sub-horizontal apical setae, one pair of strong, diverging subapical setae and one pair of weak, convergent basal setae (basal setae about 1/3 as long as subapical setae). Subscutellum conspicuously bulbous and at least as prominent as scutellum (Fig. 7a). Anterior and posterior lappets of metathoracic spiracle subequal in size (though not symmetrical). Legs exceptionally long and slender. Coxae, femora and tibiae yellow, tarsi dark brown. Medial anterior surface of fore coxa bare or predominantly bare. Fore tarsus about twice the length of fore tibia (Fig. 7a). Mid tibia with 1 anterodorsal seta. Hind tibia with 2 dorsal preapical setae and with preapical posteroventral seta undeveloped. Tegula and basicosta yellow. Second costal section setulose ventrally. Costal spine not developed. Cell r_4+5_ narrowly open at wing margin. Bend of vein M_1_ with a short appendix at most 3/4 as long as crossvein r-m (Fig. 7d). Abdomen unusually long and narrow (female unknown), slightly tapering towards apex (Fig. 7e). Mid-dorsal depression on abdominal syntergite 1+2 confined to anterior 1/3 (or less) of syntergite. Abdominal syntergite 1+2 and tergites 3 and 4 with 4 strong marginal setae (2 median, 2 lateral), without discal setae (Fig. 7a, e); tergite 5 with 4 strong discal and marginal setae (Fig. 7e). Male: Frons at its narrowest point about 0.3 times as wide as eye in dorsal view; inner vertical setae short and crossed; outer vertical seta not or barely distinguishable from postocular setae; upper reclinate orbital setae absent; fronto-orbital plate nearly bare, without proclinate orbital setae.

##### Mesnilotrix empiformis

Taxon classificationAnimaliaDipteraTachinidae

(Mesnil, 1976) comb. n.

Fig. 7

###### Type material examined.

Holotype ♂ of *Dexiotrix empiformis* Mesnil: “Madagascar Centre/ Ambohitantely 1600m/ det Ankazobe/ B. Stuckenberg”; “6.i.58”; “TYPE” [red label]; “*Dexiotrix*/ *empiformis* Mesn/ L.P. Mesnil det., 1975”; “*Mesnilotrix*/ *empiformis* (Mesnil, 1976)/ Cerretti, O’Hara & Wood det 2014” (MNHN). Paratype ♂: “Ambohitantely/ Tampoketsi 1600m/ Ankazobe/ 27-XII 56 R.E.” (MNHN).

###### Description.

See Mesnil (1976: 48, as *Dexiotrix empiformis*).

###### Remarks.

The dexiine genus *Dexiotrix* was erected by Villeneuve (1936e: 330) for the single species *Dexiotrix longipennis*, based on three females from Sichuan (China). The genus remained monotypic until Mesnil (1976: 48) described *Dexiotrix empiformis* based on three males from Madagascar, stressing that “tout en appartenant à une autre espèce, rentrent parfaitement dans le genre *Dexiotrix* Vill.” Mesnil did not provide details supporting this claim except to note that affinities between the faunas of Asia and Madagascar are well known. Nothing further was done on this group until Zhang and Shima (2005) redefined the dexiine genus *Trixa* Meigen to include *Dexiotrix* and the morphologically similar *Trixella* Mesnil.

Zhang and Shima (2005) formally assigned *empiformis* to the newly defined *Trixa* and included it in their key to the world species of *Trixa*. However, these authors did not examine specimens of *empiformis* and based their characterization of the species in the key on the original description of Mesnil (1976). This may be the reason why *empiformis* does not fully conform to their revised generic diagnosis of *Trixa*. For instance, *Mesnilotrix empiformis* possesses a narrow and concave face and a short, cylindrical palpus (Fig. 7c). Both these features are strikingly different from those shared by the remaining *Trixa* species *sensu* Zhang and Shima (2005), which have a broad and flat face, and a well-developed, “strongly inflated” palpus. *Mesnilotrix empiformis* is further characterized by: abdomen long, subcylindrical (Fig. 7e), and gently bent ventrally (Fig. 7a); lateral postpronotal seta and presutural supra-alar seta both enormously developed (Fig. 7b); and anterior and posterior lappets of metathoracic spiracle subequal in size. We therefore conclude that morphological evidence does not support the assignment of *empiformis* to *Trixa*.

Shape of the face, palpus, metathoracic spiracular lappets and abdomen are probably derived features that *Mesnilotrix empiformis* shares with the Malagasy endemic genus *Chaetodexia* Mesnil (Fig. 3), known from four species. Monophyly of *Chaetodexia* is supported by one probably derived character state in the male; i.e., the presence of a pair of strong median discal setae on abdominal tergites 3–5 which are subparallel, reclined at about 30° to horizontal and crossed (in lateral view) with the erect median marginal setae of the corresponding tergites (see Fig. 3). Moreover, all species of *Chaetodexia* possess strong apical and basal scutellar setae (in addition to an even stronger pair of subapical setae) and normally developed outer postpronotal and presutural supra-alar setae. *Mesnilotrix empiformis* differs by having abdominal tergites 3 and 4 without median discal setae (those on tergite 5 are erect), basal and apical scutellar setae strongly reduced in size (i.e., less than 1/2 the length of subapical setae) and, as mentioned above, outer postpronotal and presutural supra-alar setae both enormously developed. For these reasons we do not believe that *empiformis* should be assigned to the genus *Chaetodexia*, nor to any other named dexiine genus, and thus have chosen to erect the new genus *Mesnilotrix* for it.

#### Exoristinae, Blondeliini

##### Filistea

Taxon classificationAnimaliaDipteraTachinidae

Cerretti & O’Hara gen. n.

http://zoobank.org/DAB6C185-6871-4046-B856-8C1B5831CCCE

Figs 8 , 9

###### Type species.

*Viviania aureofasciata* Curran, 1927, by present designation.

###### Etymology.

The holotype of our new species *Filistea verbekei* below bears a label written by Verbeke identifying it by the unpublished generic name “*Filistea*”. We have chosen to use this name for our new genus, although we do not know its meaning or etymology. It is to be treated as a feminine noun.

###### Diagnosis.

An attractively patterned fly. Thoracic dorsum with a black submedian postsutural spot and two or four black presutural vittae standing out against the golden-microtomentose scutum. Ground colour of body black. Abdominal tergites 3 to 5 each with a distinct basal band of golden microtomentum strongly contrasting with black remainder. Wing almost entirely brown coloured. Compound eye bare. Ocellar setae well developed, proclinate. Frons 0.52–0.62 (male), 0.90–1.05 (female) times as wide as compound eye in dorsal view. Outer vertical seta not differentiated from postocular setae in both sexes. Two upper reclinate orbital setae (only 1 in a male from D.R. Congo). Male without, female with 2 proclinate orbital setae. Parafacial bare below lower frontal seta. Parafacial at its narrowest point 0.9–2.0 times as wide as width of postpedicel. Facial ridge straight or slightly concave, with short, fine, decumbent setulae on lower 1/5–1/4 of its length. Lower facial margin not visible in lateral view. Antenna arising above level of middle of eye height when head seen in lateral view. Postpedicel 2.2–2.9 times as long as pedicel. Arista apparently bare (i.e., longest microtrichia distinctly shorter than maximum basal diameter of arista), thickened on basal 1/4–1/3. First aristomere shorter than wide; second aristomere about as long as wide. Genal dilation well developed. Occiput flat to slightly concave. Lower occiput and postgena covered with pale setulae. Upper occiput with one or more rows of black occipital setulae. Vibrissa arising above level of lower facial margin. Palpus varying from cylindrical to slightly clavate. Prementum not more that 3.5 times as long as wide. Prosternum usually bare, rarely with 1–5 fine setulae laterally. Proepisternal depression bare. Proepisternal seta present. Postpronotum with 3 setae arranged in a line or in a shallow triangle. Scutum with 1 + 3 intra-alar setae; 2–3 + 3 dorsocentral setae; 3 + 3 acrostichal setae. First postsutural supra-alar seta shorter than first postsutural intra-alar seta, shorter than first postsutural dorsocentral seta and at most as long as notopleural setae. Katepimeron bare. Three katepisternal setae (2+1). Scutellum with 5 pairs of marginal setae: one pair of apical setae, crossed and sub-horizontal; one pair of subapical setae, well developed and divergent; two pairs of lateral setae (anterior pair shorter and less divergent than posterior pair); one pair of converging basal setae. Wing cell r_4+5_ narrowly open at wing margin. Mid tibia with 2 or more anterodorsal setae and a strong ventral seta. Hind coxa bare posterodorsally. Hind tibia with a row of anterodorsal setae irregular in length and thickness and 2 or 3 dorsal preapical setae. Mid-dorsal depression of abdominal syntergite 1+2 reaching posterior margin of syntergite. Syntergite 1+2 with 1 pair of median marginal setae; tergite 3 with one pair or a complete row of marginal setae; tergite 4 with a complete row of marginal setae. Tergites 3 and 4 with median discal setae.

###### Remarks.

The bare compound eye, the vibrissa arising far above lower facial margin, the presence of robust, crossed and horizontal apical scutellar setae, together with a usually bare prosternum and an unmodified oviscapt are the main character states that separate *Filistea* from the other Afrotropical Blondeliini. Moreover, the unique colour pattern of the body and the darkened wing membrane make *Filistea* easily identifiable among Afrotropical tachinids. We also examined all available keys to genera of other regions and compared our specimens with blondeliine descriptions and specimens in collections, paying special attention to those of the Palaearctic and Oriental regions, and did not find any basis for assigning *Filistea aureofasciata* and *Filistea verbekei* to a known genus. We thus erect a new genus for these two Afrotropical species.

**Figure 8.**
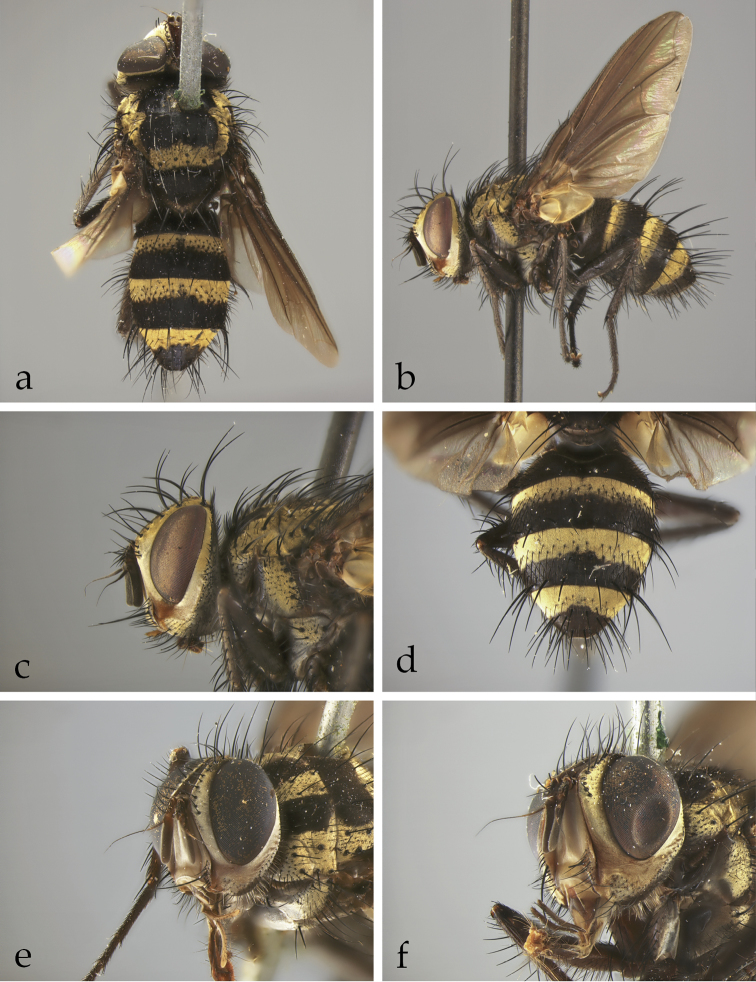
*Filistea* Cerretti & O’Hara, gen. n. **a** male habitus in dorsal view of *Filistea aureofasciata* (Curran) (Cameroon, ZMHB) **b–d** *Filistea verbekei* Cerretti & O’Hara, sp. n. (female paratype, Uganda, CNC) **b** habitus in lateral view **c** head in lateral view **d** abdomen in dorsal view **e** head in laterofrontal view of *Filistea verbekei* (male holotype, ZMHB) **f** male head in laterofrontal view of *Filistea aureofasciata* (Curran) (Cameroon, ZMHB).

**Figure 9.**
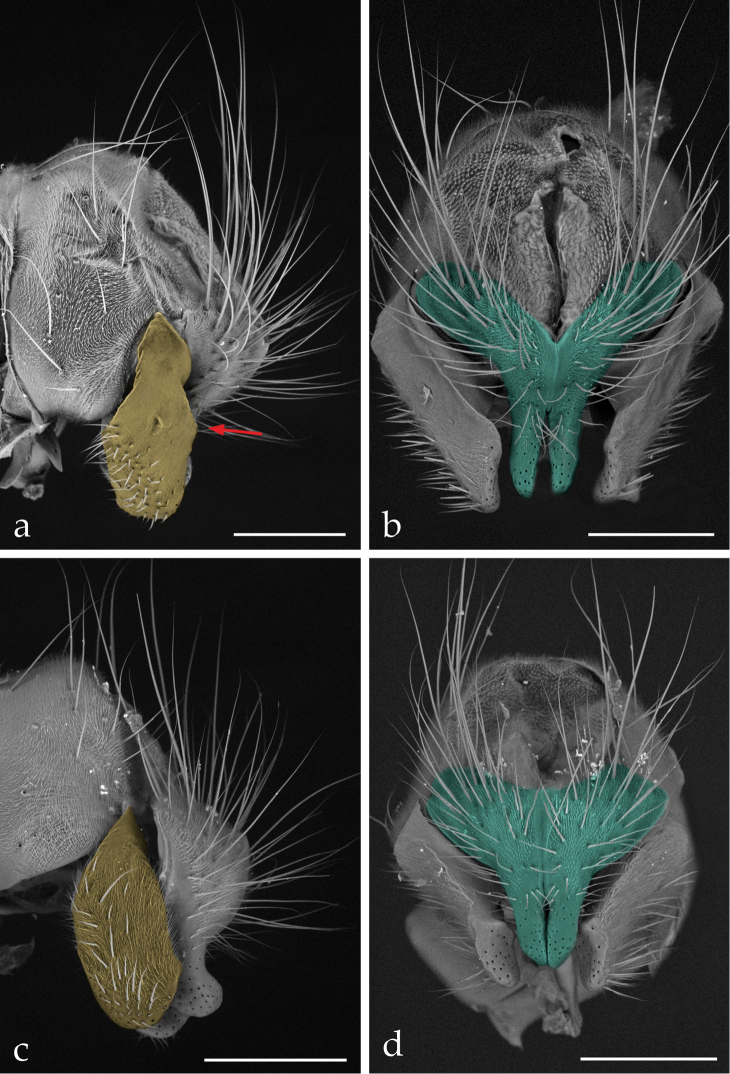
Epandrial complex of *Filistea* Cerretti & O’Hara, gen. n. **a–b** *Filistea aureofasciata* (Curran) (male, Cameroon, ZMHB) **a** lateral view (red arrow indicates a thickening along the posterior margin of surstylus) **b** posterior view **c–d** *Filistea verbekei* Cerretti & O’Hara, sp. n. (male holotype, ZMHB) **c** lateral view **d** posterior view. Colour coding: green = cerci; yellow = surstylus. Scale bars: 200 μm.

##### Filistea aureofasciata

Taxon classificationAnimaliaDipteraTachinidae

(Curran, 1927) comb. n.

Figs 8a, f , 9a–b

###### Type material examined.

Holotype ♂ of *Viviania aureofasciata* Curran: “Stanleyville, Cgo./ 25°10′E 0°30′N./ III.1915”; “Lang & Chapin/ Collectors”; “Taken from Bembex”; “*Viviania*/ TYPE/ *aureofasciata*/ Curran ♂/ No.”; “*Viviania*/ *aureofasciata*/ Det. Curran./ C.H. Curran” (AMNH).

###### Other material examined.

1♂: “3/5.96”; “N.Kamerun [Cameroon]/ Johann-Albrechtshöhe [Kumba, 4°38′N 9°28′E]/ L. Conradt S6”; “Zool. Mus./ Berlin” (ZMHB). 1♀: same data as male except “27/5.96” (ZMHB). 1 ♀: “Kayonza Forest/ Kigezi Dist[rict] UGANDA/ 2135 M. May 1972/ E. Babyetagara” (CNC).

###### Description.

For external morphology see Curran (1927: 8, as *Viviania aureofasciata*). A key to separate *Filistea aureofasciata* and *Filistea verbekei* is given below.

*Male terminalia* (Fig. 9a–b): Tergite 6 divided into two hemitergites. Sternite 6 asymmetrical and right side connection to segment 7 membranous. Posterior margin of sternite 5 with a deep median cleft. Epandrium short and convex. Cerci short in posterior view, distal 1/3 strongly bent posteriorly. Proximal 1/3 of cerci in posterior view very broad, cerci narrowing and slightly separated distally (Fig. 9b). Surstylus well developed, about as long as cerci, more or less lobe-like in lateral view, with posterior margin characterized by a broad thickening at about midlength (Fig. 9a, red arrow); surtylus not fused with epandrium. Lateral surface of surstylus without a thick cover of fine appressed setulae, posterodistal 1/2 of surstylus with several robust setae. Bacilliform sclerite rod-shaped and narrowly fused with surstylus anterobasally. Hypandrial arms not fused posteromedially. Pregonite well developed, sub-triangular, moderately hook-shaped distally. Postgonite distally rounded and gently bent anteriorly. Intermedium well developed. Ejaculatory apodeme present, small. Basal processes of basiphallus present. Epiphallus well developed and arising dorsally at sub-basal position of basiphallus. Ventral wall of distiphallus concave. Lateroventral region of distiphallus sclerotized. Medioventral ridge of distiphallus not developed. Extension of dorsal sclerite of distiphallus short.

###### Distribution.

Cameroon, D.R. Congo, Nigeria, Uganda.

##### Filistea verbekei

Taxon classificationAnimaliaDipteraTachinidae

Cerretti & O’Hara sp. n.

http://zoobank.org/C44D4695-466D-48F8-A969-A8FCF3E3FA90

Figs 8b–e , 9c–d

###### Type material.

Holotype ♂: “16/11.95”; “N.Kamerun [Cameroon]/ Johann-Albrechtshöhe [Kumba, 4°38′N 9°28′E]/ L. Conradt S6”; “Zool. Mus./ Berlin” (ZMHB). Paratypes. 1♂: “Congo Belge [D.R. Congo]: Kiwu/ Beni (poste)/ 18-VI-1953/ J. Verbeke.- KEA:”; “→ *Zenillia*/ *Filistea* ng./ *caparti* nsp.”; “N.gen. n-sp./ pris de/ *Bacromyiella*/ (Erythocerinae)” (IRSNB). 1♂: “Kamerun [Cameroon]/ Bidunbi/ 1-15V 05/ G. Teßmann S.G”; “207-02”; “Zool. Mus./ Berlin” (ZMHB). 1♂, 1♀: “Budongo Forest nr/ Lk Albert UGANDA/ Mar 20-31 1972/ H. Falke 915m. (CNC). 1♂, 1♀: “Entebbe, UGANDA/ 5.III.1972/ H. Falke/ In forest” (CNC). 1♂, 1♀: “Entebbe, UGANDA/ 7.II.1972/ H. Falke/ in Forest” (CNC). 1♂: “Nr Entebbe,/ UGANDA/ Jan.23-31,1973/ H. Falke, 1160m.” (CNC). 1♀: “Kampala UGANDA/ June 1-10, 1972/ 1150 M./ E. Babyetagara” (CNC). 1♀: “2659 4 M. NW/ of Agege Lagos/ State Nigeria/ 30 XII 73/ M.A. Cornes” (CNC). 1 ♀: “Cameroon/ Mt Cameroon/ 1000-1800 m/ 11-13.XI.1987/ Fini Kaplan” (TAU).

###### Etymology.

This species is named in honour of Jean Verbeke for his significant contributions to Afrotropical Tachinidae and for labelling our holotype of *Filistea verbekei* with the manuscript name we have chosen as the valid name of this genus.

###### Description.

*Body length*: 6–8 mm.

Male. *Colouration*: Head black or brownish-black in ground colour, covered with thick golden reflecting microtomentum. Antenna black. Palpus yellow. Tegula and basicosta black. Lower calypter smoky. Legs dark brown to black.

*Head* (Fig. 8b–c, e): Frons at its narrowest point 0.5–0.6 times as wide as compound eye in dorsal view. Parafacial at its narrowest point 0.9–1.5 times as wide as postpedicel. Vibrissa arising slightly above lower facial margin. Gena 0.27–0.37 times as high as compound eye. Postpedicel 2.3–2.8 times as long as pedicel.

*Thorax*: Anepimeral seta well developed. Anatergite bare below lower calypter. Posterior lappet of metathoracic spiracle visibly larger than anterior lappet. Medial margin of lower calypter more or less abutting lateral margin of scutellum. Second costal segment ventrally bare. Costal spine varying from slightly shorter than to 1.5 times as long as crossvein r-m. Base of R_4+5_ with a few short setulae. Fourth costal section longer than sixth. Section of M_1_ between r-m and dm-m varying from slightly longer to as long as section between dm-m and bend of M_1_. Medial anterior surface of fore coxa bare. Preapical anterodorsal seta of fore tibia distinctly shorter than preapical dorsal seta. Preapical posteroventral seta of hind tibia shorter than preapical anteroventral seta.

*Abdomen*: General setulae of tergites 3 to 5 erect. Tergite 5 about 0.8–1.0 times as long as tergite 4.

*Male terminalia* (Fig. 9c–d): As described for *Filistea aureofasciata* except: Surstylus in lateral view more or less parallel-sided in shape, distally rounded, with posterior margin straight (Fig. 9c). Lateral surface of surstylus covered with fine appressed setulae and with several robust setae along posterior 1/2.

Female differs from male as follows. Lower calypter yellowish-white. Frons at its narrowest point 0.9–1.1 times as wide as compound eye in dorsal view. Two proclinate orbital setae. General setulae of tergites 3 to 5 recumbent.

###### Distribution.

Cameroon, D.R. Congo, Nigeria, Uganda.

##### Key to species of *Filistea* gen. n.

**Table:** 

1	Palpus black. Abdominal tergite 3 usually with 2 median marginal setae (rarely 4). Male: parafacial at its narrowest point 1.8–2.0 times as wide as width of postpedicel; posterior margin of surstylus in lateral view characterized by a broadening at about midlength (Fig. 9a); lateral surface of surstylus without fine appressed setulae and with several short, robust setae on posterodistal 1/2 (Fig. 9a). Female: parafacial at its narrowest point 1.8–2.5 times as wide as width of postpedicel	***Filistea aureofasciata* (Curran)**
–	Palpus yellow. Abdominal tergite 3 with a complete row of median marginal setae. Male: parafacial at its narrowest point 0.9–1.8 times as wide as width of postpedicel; posterior margin of surstylus in lateral view straight (Fig. 9c); lateral surface of surstylus covered with fine appressed setulae and with several more robust setae along posterior 1/2 (Fig. 9c). Female: parafacial at its narrowest point 1.0–2.0 times as wide as width of postpedicel	***Filistea verbekei* sp. n.**

#### Exoristinae, Eryciini

##### Afrophylax

Taxon classificationAnimaliaDipteraTachinidae

Cerretti & O’Hara gen. n.

http://zoobank.org/083AFCF1-CD81-4CE4-BC3B-C8FCE22D1358

Fig. 10

###### Type species:

*Sturmia aureiventris* Villeneuve, 1910, by present designation.

###### Etymology.

*Afrophylax* is a composite word formed from *Afro* (African) and the suffix of the generic name *Argyrophylax* Townsend. The name alludes to the morphological external similarity between *Afrophylax* and *Argyrophylax* that led Mesnil (1950b) to assign *aureiventris* to *Argyrophylax*.

###### Diagnosis.

**Figure 10.**
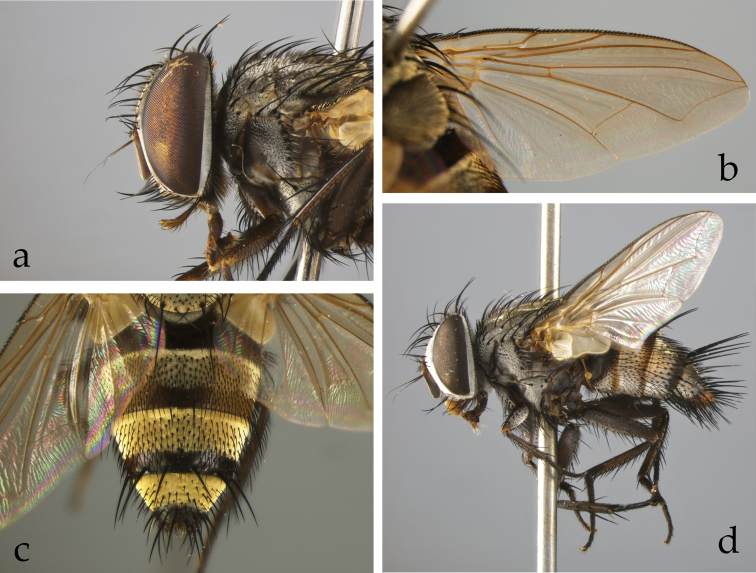
*Afrophylax* Cerretti & O’Hara, gen. n. **a–c** *Afrophylax aureiventris* (Villeneuve) (male, Nigeria, MZUR) **a** head in lateral view **b** wing **c** abdomen in dorsal view **d** female of *Afrophylax aureiventris*, habitus in lateral view (Uganda, TAU).

Compound eye bare. Ocellar setae well developed, proclinate. Male with 1 strong proclinate orbital seta arising on posterior 1/2–1/3 of fronto-orbital plate, female with 2 proclinate orbital setae. Parafacial bare below lower frontal seta. Parafacial very narrow, at its narrowest point 0.5–0.7 times as wide as width of postpedicel. Facial ridge straight or convex, with short, fine, decumbent setulae on lower 1/5 or less of its length. Lower facial margin not visible in lateral view. Antenna arising at about level of middle of eye height when head seen in lateral view. Postpedicel 2.9–3.9 times as long as pedicel. Arista apparently bare, thickened on basal 1/5. First aristomere shorter than wide; second aristomere about as long as wide. Genal dilation well developed, though very narrow as gena is reduced to a narrow strip in male. Gena slightly wider in female but not more than 0.06 times as high as compound eye. Occiput concave, covered with only pale hair-like setulae. Vibrissa arising at level of lower facial margin. Palpus slightly clavate in male, grossly clubbed in female. Prementum not more that 2.5 times as long as wide. Scutum and scutellum evenly covered with light silver and/or yellow reflecting microtomentum that is particularly bright when thorax is seen in anterodorsal view. Prosternum with at least 3 pairs of setulae along lateral margin. Proepisternal depression bare. Proepisternal seta present. Postpronotum with 3 setae arranged in a line (sometimes a fourth weak seta present in front of middle basal one). Scutum with 1 + 3 intra-alar setae; 3 + 4 dorsocentral setae; 3 presutural acrostichal setae. First postsutural supra-alar seta longer than notopleural setae and longer and stronger than first postsutural intra-alar seta. Katepimeron bare or with 1–3 setulae on anterior 1/4. Three katepisternal setae (1+2; i.e., ventral seta arising closer to posterior dorsal seta than to anterior dorsal seta) (Fig. 10d). Scutellum with 4 pairs of marginal setae and 1 pair of discal setae: apical scutellar setae weak (2/5–1/2 as long as subapical setae), crossed and sub-horizontal; lateral setae 2/5–2/3 as long as subapical setae. Wing cell r_4+5_ open at wing margin. Mid tibia with 1 strong anterodorsal seta and a strong submedian ventral seta. Hind coxa bare posterodorsally. Mid-dorsal depression of abdominal syntergite 1+2 reaching posterior margin of syntergite. Syntergite 1+2 and tergite 3 with 1 pair of median marginal setae (those on syntergite weak). Tergite 4 with a complete row of marginal setae. Tergites 3 and 4 without median discal setae, and with general setulae decumbent. Egg: macrotype, membranous, fully embryonated.

##### Afrophylax aureiventris

Taxon classificationAnimaliaDipteraTachinidae

(Villeneuve, 1910) comb. n.

Fig. 10

###### Type material examined.

Holotype ♂ of *Sturmia aureiventris* Villeneuve: “Sturmia/ aureiventris/ n. sp.” [handwritten]; “Coll. J. Villeneuve:/ Sturmia/ aureiventris Vill./ R.M.H.N. Belg. 15.392” [2nd and 3rd lines handprinted]; “Typus” [handwritten]; “TYPE” [red label] (MRAC).

###### Other material examined

[line breaks on labels not recorded]. 1♂: N.Kamerun [Cameroon], Johann-Albrechtshöhe [Kumba, 4°38′N 9°28′E] (ZMHB). 1 ♂: 54-57 Ikorodu, Lagos State, Nigeria, 1 IX 71, M.A. Coines (CNC). 1♂: Nigeria, Ife, 13-14 Sept 1977, S. Shinonaga. 2♂: same data but date 29-31 Aug 1977 (all in MZUR, ex H. Shima collection). 1♂, 1♀: Uganda, Impenetrable Forest, S.W. Uganda, 27.1.72, A. Freidberg (TAU).

###### Redescription.

*Body length*: 7.5–8.5 mm.

Male. *Colouration* (Fig. 10a, c): Head black or brownish-black in ground colour, covered with thick silver reflecting microtomentum. Scape and pedicel brownish-black; postpedicel mostly black, yellowish-brown at junction with pedicel. Palpus basally brown, shading into yellow apically. Postpronotum and notopleuron yellowish-brown in ground colour. Scutum mid-dorsally black, shading into yellowish around postpronotum, in front of scutellum and around transverse suture. The usual 4 dark presutural vittae of scutum very narrow and barely visible in posterodorsal view. Scutellum black basally, shading into yellowish on apical 1/2–3/4. Tegula black; basicosta varying from light brown to dark brown. Legs dark brown except for the brownish tibiae (colour is more pale at junction between femora and tibiae). Abdominal colouration distinctive (Fig. 10c), with conspicuous, sharply defined dark hind margins on tergites 3–5, basal parts of these tergites yellow microtomentose over pale ground colour (thus appearing golden-orange).

*Head* (Fig. 10a): Frons at its narrowest point 0.4–0.5 times as wide as compound eye in dorsal view. Inner vertical seta well developed, reclinate. Outer vertical seta short but distinct. Upper 3 frontal setae proclinate. Frontal setae descending to slightly above lower margin of pedicel. Fronto-orbital plate with erect, short, hair-like setulae. Two upper reclinate orbital setae (anterior one distinctly longer than second and slightly longer than ocellar seta). Parafacial at its narrowest point about 0.5 times as wide as postpedicel. Face and lower facial margin not visible in lateral view. Genal dilation well developed, though very narrow and visible only in ventral view. Gena very narrow, 0.02–0.04 times as high as compound eye. Postpedicel 3.0–3.9 times as long as pedicel. First and second aristomeres not longer than wide. Prementum 2–3 times as long as wide. Palpus sub-cylindrical to slightly enlarged distally.

*Thorax* (Fig. 10b): Anepimeral seta short but distinct. Anatergite bare below lower calypter. Posterior lappet of metathoracic spiracle visibly larger than anterior lappet. Medial margin of lower calypter more or less abutting lateral margin of scutellum. Wing membrane hyaline. Second costal segment ventrally bare. Costal spine not differentiated from other costal setulae. Vein R_1_ bare. Base of R_4+5_ with 2–4 short setulae. Fourth costal section longer than sixth. Section of M_1_ between crossveins r-m and dm-m clearly longer than section between dm-m and bend of M_1_. Medial anterior surface of fore coxa bare. Preapical anterodorsal seta of fore tibia distinctly shorter than preapical dorsal seta. Hind tibia with 2 dorsal preapical setae. Preapical posteroventral seta of hind tibia shorter than preapical anteroventral seta. Hind tibia with regular, comb-like row of anterodorsal setae.

*Abdomen* (Fig. 10c): Tergite 5 about 0.8–0.9 times as long as tergite 4.

*Male terminalia*: Not examined.

Female (Fig. 10d) differs from male as follows.

Frons at its narrowest point 0.76 times as wide as compound eye in dorsal view. Postpedicel about 3 times as long as pedicel. Gena 0.06 times as high as compound eye. Palpus grossly clubbed; i.e., its maximum diameter about 1.5 times as wide as fore tibia at midlength. Abdomen mostly black in ground colour. Egg: macrotype, membranous (Eryciini type).

###### Distribution.

Cameroon, D.R. Congo, Nigeria, Sierra Leone, Tanzania, Uganda.

###### Remarks.

Mesnil (1950b: 19–20) assigned two of Villeneuve’s Afrotropical species, *Sturmia aureiventris* Villeneuve, 1910 and *Carcelia nudioculata* Villeneuve, 1938, to *Argyrophylax* Brauer & Bergenstamm, 1889. Crosskey (1980b) did not recognize *Argyrophylax* from the Afrotropical Region, returning *Carcelia nudioculata* to *Carcelia* and treating *Sturmia aureiventris* as an unplaced species of “Carceliini”. Crosskey (1984: 277) keyed out *aureiventris* (as “‘Argyrophylax’ aureiventris”) separately in his key to genera of Carceliini and Anacamptomyiini. Although Crosskey noted in his key that the species does not belong to *Argyrophylax*, he did not suggest an alternative placement.

The genus *Argyrophylax* is widespread in the Neotropical, Oriental and Australasian regions and a few species reach the southern Nearctic and eastern Palaearctic regions. The type species of *Argyrophylax* (the New World species *Argyrophylax albincisus* (Wiedemann, 1830)), as well as other congeners of which the reproductive system has been examined, is characterized by a long and convoluted common oviduct retaining hundreds of microtype, plano-convex, fully embryonated eggs. Females of *Afrophylax aureiventris* have a different reproductive strategy and lay macrotype membranous eggs and cannot be assigned to *Argyrophylax*. Moreover, we have determined that this species does not fit within the limits of an existing tachinid genus (see diagnosis) and propose for it the new genus *Afrophylax*.

##### Carceliathrix

Taxon classificationAnimaliaDipteraTachinidae

Cerretti & O’Hara gen. n.

http://zoobank.org/C8625FED-27A0-4FA8-A088-1718F48F67EF

Fig. 11

###### Type species.

*Phorocera crassipalpis* Villeneuve, 1938, by present designation.

###### Etymology.

The compound name *Carceliathrix* is formed from the generic name *Carcelia* Robineau-Desvoidy and the Greek noun *thrix* (meaning hair). *Carceliathrix* resembles *Carcelia* in possessing a narrow gena and a setose posterodorsal margin of the hind coxa. The suffix *thrix* refers to the row of setae on the facial ridge.

###### Diagnosis.

**Figure 11.**
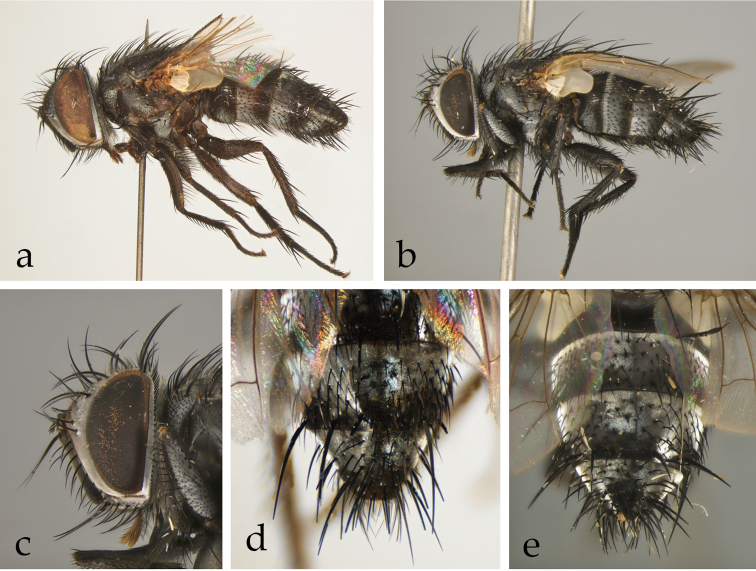
*Carceliathrix* Cerretti & O’Hara, gen. n. **a** male habitus in lateral view of *Carceliathrix crassipalpis* (Villeneuve) (lectotype, MRAC) **b** female habitus in lateral view of *Carceliathrix* sp. 2 (South Africa, NMB) **c** female head in lateral view of *Carceliathrix* sp. 2 **d** male abdomen in dorsal view of *Carceliathrix* sp. 1 (Namibia, NMNW) **e** female abdomen in dorsal view of *Carceliathrix* sp. 2.

Compound eye covered with thick, long ommatrichia (each ommatrichium clearly longer than diameter of 3 eye facets). Frontal vitta normally developed, about 1/2–2/3 as wide as fronto-orbital plate measured at midlength. Ocellar seta well developed, proclinate. No proclinate orbital setae in male, 2 in female. Parafacial bare. Facial ridge convex, with a row of strong, downcurved setae above vibrissa, on lower 1/2–2/3 of its length (Fig. 11a–c). Lower facial margin not visible in lateral view. Lower occiput and postgena covered with mostly pale hair-like setulae. Vibrissa arising at level of lower facial margin. Arista apparently bare; arista thickened on proximal 1/4–2/5. Palpus slightly clavate. Prosternum with at least 3 setulae along lateral margin. Proepisternal depression bare. Proepisternal seta present. Postpronotum with 4 setae, the 3 strongest arranged in a triangle. Scutum with 1 + 3 postsutural intra-alar setae; 3 + 4 dorsocentral setae. First postsutural supra-alar seta longer than notopleural setae and longer and stronger than first postsutural intra-alar seta. Katepimeron bare. Three katepisternal setae (2+1). Scutellum with 4 pairs of marginal setae and 1 pair of discal setae: apical setae crossed, horizontal or slightly tilted upwards by at most 30° to horizontal. Wing cell r_4+5_ open. Mid tibia with 1–5 anterodorsal setae and a strong submedian ventral seta. Hind coxa with 1 or more short setae arising posterodorsally (Fig. 11a–b). Mid-dorsal depression of abdominal syntergite 1+2 reaching posterior margin of syntergite. Syntergite 1+2 and tergite 3 with 1 pair of median marginal setae. Tergite 4 with a complete row of marginal setae. Tergites 3 and 4 with several robust, short median discal setae or setulae irregularly dispersed, sometimes barely distinguishable from general erect setulae.

###### Remarks.

A convex facial ridge characterized by having a row of strong, downcurved setae on lower 1/2−2/3 is the main, probably derived, character state that separates *Carceliathrix* from the widespread and speciose genus *Carcelia*. However, the compound eye covered with long ommatrichia, a narrow gena and setose facial ridge are traits shared by the anacamptomyiine genera *Anacamptomyia* and *Parapales* from which *Carceliathrix* is readily distinguished by having strong and proclinate ocellar setae, frontal vitta at least 1/2 as wide as width of fronto-orbital plate, postpronotum with the 3 strongest setae arranged in a triangle, and male without sexual patches on the abdominal tergites. We have determined that *Phorocera crassipalpis* Villeneuve and the two probably undescribed species from Namibia (sp. 1) and South Africa (sp. 2) listed below do not fit within the limits of an existing genus and propose for them the new genus *Carceliathrix*.

##### Carceliathrix crassipalpis

Taxon classificationAnimaliaDipteraTachinidae

(Villeneuve, 1938) comb. n.

Fig. 11a

###### Type material examined.

Lectotype ♂ (MRAC) and paralectotype ♀ (CNC), as designated above in Lectotype Designations section.

###### Redescription.

*Body length*: 5.6–7.0 mm.

Male. *Colouration*: Head black or brownish-black in ground colour, covered with silver reflecting microtomentum. Pedicel brownish-black, postpedicel black. Palpus proximally brown, shading into yellow distally. Scutum black in ground colour, with 4 dark presutural vittae, lateral pair triangular, median pair very narrow (about 1/7–1/6 as wide as microtomentose band between them). Scutellum mainly black, shading into reddish-brown apically. Tegula black; basicosta dark brown. Legs dark brown except for the brownish tibiae. Abdomen black, with bands of microtomentum covering about proximal half of tergites 3, 4 and 5.

*Head* (Fig. 11a): Frons at its narrowest point 0.9 times as wide as compound eye in dorsal view. Inner vertical seta well developed, reclinate. Outer vertical seta present but barely distinguishable from postocular setae. Frontal setae descending slightly below base of arista. Fronto-orbital plate with a few scattered hair-like setulae. Two upper reclinate orbital setae of approximately the same size. Parafacial at its narrowest point about 0.4 times as wide as postpedicel. Face and lower facial margin not visible in lateral view. Genal dilation well developed. Upper occiput without black setulae behind postocular row. Gena very narrow, about 0.1 times as high as compound eye. Postpedicel 4.0–5.5 times as long as pedicel. First and second aristomeres not longer than wide. Prementum 2–3 times as long as wide. Palpus slightly enlarged distally, dorsoventrally flattened.

*Thorax*: Scutum with 3 + 3 acrostichal setae; 3 posthumeral setae. Anepimeral seta well developed. Lateral scutellar setae about 3/5 as long as subapical setae. Anatergite bare below lower calypter. Posterior lappet of metathoracic spiracle visibly larger than anterior lappet. Medial margin of lower calypter more or less abutting lateral margin of scutellum. Wing membrane hyaline (both wings badly damaged in the lectotype). Second costal segment ventrally bare. Costal spine not differentiated from other costal setulae. Vein R_1_ bare. Base of R_4+5_ with 3 short setulae. Medial anterior surface of fore coxa bare. Preapical anterodorsal seta of fore tibia distinctly shorter than preapical dorsal seta. Hind tibia with 2 dorsal preapical setae. Preapical posteroventral seta of hind tibia shorter than preapical anteroventral seta. Anterodorsal setae of hind tibia irregular in length and thickness.

*Abdomen*: Tergite 5 about 0.8 times as long as tergite 4.

*Male terminalia*: Not examined.

Female differs from male as follows.

Outer vertical seta well developed. Wing features not examined (both wings missing in paralectotype).

###### Distribution.

D.R. Congo.

###### Remarks.

Villeneuve (1938c: 2) described *crassipalpis* within a broadly defined *Phorocera*. The species was left unplaced in the “Carceliini” (= Eryciini, in part) by Crosskey (1980b: 867) and was not keyed or discussed by Crosskey (1984). We recognize it as belonging to our new genus, *Carceliathrix*, and record below two additional species that we do not describe at this time due to the paucity of material. Based on present evidence this genus is known from these three species and is recorded from D.R. Congo, Namibia and South Africa.

##### Carceliathrix sp. 1

Taxon classificationAnimaliaDipteraTachinidae

Fig. 11d

###### Material examined.

1♂: “Namibia: RUNDU DIST./ Mile46/18°18′39″S 19°15′29″E/ 25–27.iii.2003/ A.H. Kirk-Spriggs/ Malaise traps”; “Namibian National/ Insect Collection,/ National Museum,/ P.O. Box 1203,/ Windhoek, Namibia” (NMNW).

##### Carceliathrix sp. 2

Taxon classificationAnimaliaDipteraTachinidae

Fig. 11b–c, e

###### Material examined.

1♀: “RSA: KZN, Ndumo Game R[eserve]./ main camp area at:/ 26°54.652′S 32°19.719′E/ 27-30.xi.2009/ A.H. Kirk-Spriggs”; Malaise traps/ broad-leafed deciduous/ woodland”; “Entomology Dept./ National Museum/ P.O. Box 266/ Bloemfontein 9300/ South Africa”; “BMSA(D)/ 13781” (NMB).

###### Remarks.

Females of *Carceliathrix* sp. 2 lay macrotype membranous eggs.

#### Exoristinae, Goniini

##### Myxophryxe

Taxon classificationAnimaliaDipteraTachinidae

Cerretti & O’Hara gen. n.

http://zoobank.org/BF6B3421-4A14-491B-81EB-433A4FAD7B26

Figs 12 , 13 , 14

###### Type species.

*Phorocera longirostris* Villeneuve, 1938, by present designation.

###### Etymology.

The compound name *Myxophryxe* derives from the prefix of the generic name *Myxogaedia* Villeneuve (to which *longirostris* was assigned before this revision) and the generic name *Phryxe* Robineau-Desvoidy, which is morphologically similar.

###### Diagnosis.

Compound eye covered with thick, long ommatrichia (longest ommatrichia longer than diameter of five eye facets). Ocellar setae well developed, proclinate. Frons 1.1–1.6 times as wide as compound eye in dorsal view. Parafacial bare or with a few short, fine setulae just below lower frontal seta. Parafacial flat or slightly convex, at its narrowest point 1.2–2.2 times as wide as width of postpedicel. Facial ridge straight or convex, with a row of strong, downcurved setae above vibrissa, on lower 4/5 or more of its length. Lower facial margin warped forward and slightly visible in lateral view. Postpedicel 3.9–6.3 times as long as pedicel. Arista apparently bare, thickened on basal 1/2–2/3. First aristomere shorter than wide; second aristomere about as long as wide. Genal dilation well developed. Gena in profile 0.25–0.50 times as high as compound eye. Lower occiput and postgena covered with mostly pale hair-like setulae. Upper occiput with one row of black occipital setulae. Vibrissa arising at level of lower facial margin. Palpus slightly clavate. Prementum varied. Prosternum with at least three long setulae along lateral margin. Proepisternal depression bare. Proepisternal seta present. Postpronotum with 4 or 5 setae, the 3 strongest basal ones arranged in a line. Scutum with 3 postsutural intra-alar setae; 3 + 4 dorsocentral setae; 3 presutural acrostichal setae. First postsutural supra-alar seta longer than notopleural setae and longer and stronger than first postsutural intra-alar seta. Katepimeron bare or with setulae on anterior 1/4–2/3. Three katepisternal setae (2+1). Scutellum with 4 pairs of marginal setae and 1 or 2 pairs of discal setae: apical scutellar setae crossed (sometimes converging and slightly crossed distally), sub-horizontal. Wing cell r_4+5_ open or closed at wing margin. Mid tibia with 2 anterodorsal setae (a short additional seta occasionally present) and a strong submedian ventral seta. Hind coxa bare posterodorsally. Mid-dorsal depression of abdominal syntergite 1+2 reaching posterior margin of syntergite. Syntergite 1+2 and tergite 3 with 1 pair of median marginal setae. Tergite 4 with a complete row of marginal setae. Tergites 3 and 4 without median discal setae (several robust, short median discal setae or setulae irregularly dispersed, sometimes barely distinguishable from general erect setulae).

###### Remarks.

As mentioned in the Classification section above, it is not always possible to ascertain whether a given genus belongs to the Goniini (microtype egg producers) or the Eryciini (macrotype egg producers) relying only on external morphological characters. This is especially true when only males are available for examination as has been the case for *Myxophryxe*. In spite of this, we propose here to tentatively assign *Myxophryxe* to the Goniini given the close morphological similarity of males to those of the goniine genus *Myxogaedia*. *Myxophryxe* is characterized by having the parafacial bare or with a few fine, short setulae below the lower frontal seta, arista thickened on basal 1/2–2/3, preapical anterodorsal seta of fore tibia varying from shorter to as long as preapical dorsal seta, and hind tibia with two or three dorsal preapical setae. In contrast, species of *Myxogaedia* have the parafacial with at least some strong, pro-medioclinate setae on upper 1/2, arista thickened on basal 4/5 to its whole length, preapical anterodorsal seta of fore tibia distinctly longer than preapical dorsal seta, and hind tibia with four or five dorsal preapical setae. Nevertheless, we cannot exclude that future investigation of the reproductive strategy of *Myxophryxe* species may change the current classification.

**Figure 12.**
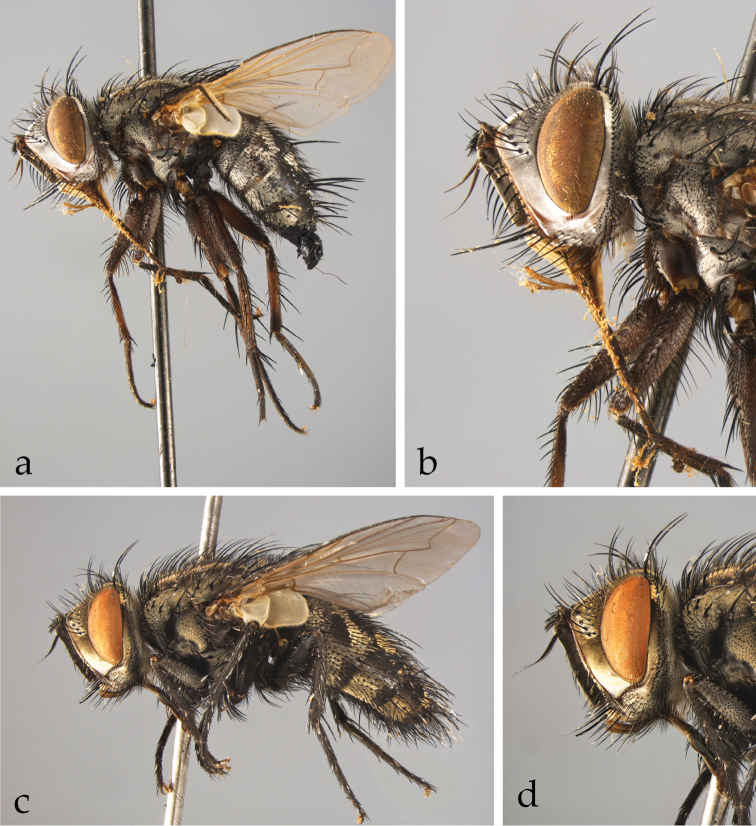
*Myxophryxe* Cerretti & O’Hara, gen. n. **a–b** *Myxophryxe longirostris* (Villeneuve) (male holotype of *Phorocera majestica* Curran, SANC) **a** habitus in lateral view **b** head in lateral view **c–d** *Myxophryxe murina* Cerretti & O’Hara, sp. n. (male holotype, NMB) **c** habitus in lateral view **d** head in lateral view.

**Figure 13.**
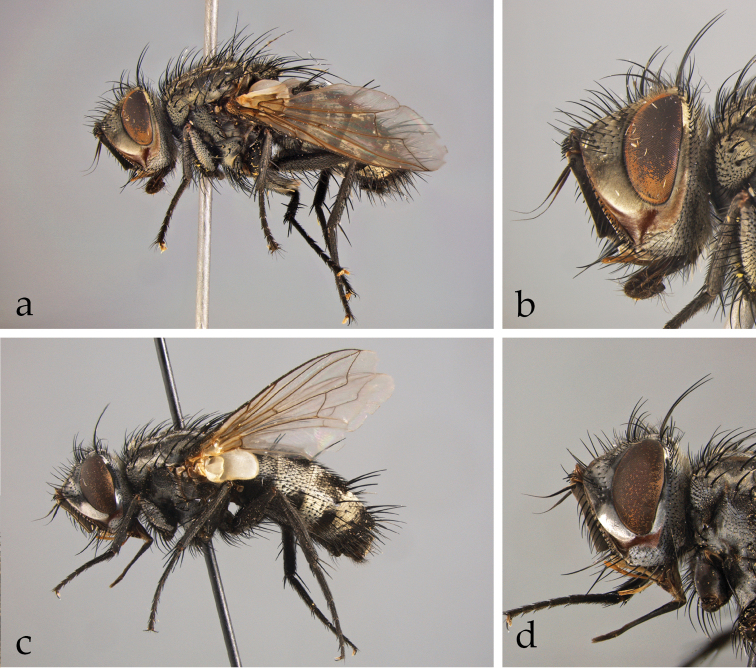
*Myxophryxe* Cerretti & O’Hara, gen. n. **a–b** *Myxophryxe regalis* Cerretti & O’Hara, sp. n. (male holotype, NMB) **a** habitus in lateral view **b** head in lateral view **c–d** *Myxophryxe satanas* Cerretti & O’Hara, sp. n. (male holotype, MZUR) **c** habitus in lateral view **d** head in lateral view.

**Figure 14.**
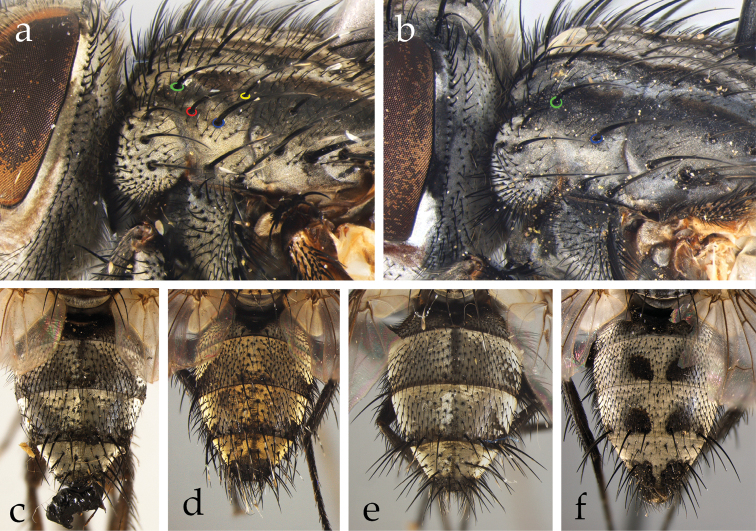
*Myxophryxe* Cerretti & O’Hara, gen. n. **a–b** head and scutum in dorsolateral view (colour coding of circles: green = base of inner posthumeral seta; red = base of outer posthumeral seta; blue = base of presutural supra-alar seta; yellow = base of presutural intra-alar seta) **a** *Myxophryxe regalis* Cerretti & O’Hara, sp. n. (male holotype, NMB) **b** *Myxophryxe satanas* Cerretti & O’Hara, sp. n. (male holotype, MZUR) **c–f** male abdomen in dorsal view **c** *Myxophryxe longirostris* (Villeneuve) (holotype of *Phorocera majestica* Curran, SANC) **d** *Myxophryxe murina* Cerretti & O’Hara, sp. n. (holotype, NMB) **e** *Myxophryxe regalis* **f** *Myxophryxe satanas*.

##### Myxophryxe longirostris

Taxon classificationAnimaliaDipteraTachinidae

(Villeneuve, 1938) comb. n.

Figs 12a–b , 13c

###### Type material examined.

Holotype ♂ of *Phorocera majestica* Curran: “New Hanover/ Natal N.29.14/ C.B. Hardenberg”; “Phorocera/ majestica/ Curran ♂/ Holotype” [red label]; “HOLOTYPE/ SANC/ TYPHO0059”; “Myxogaedia/ majestica (Curran)/ R.W. Crosskey det. 1964/ possibly same as/ longirostris Vill.” (SANC).

###### Other material examined.

1♂: “Marley/ n. 15/ 1824.[two illegible letters here]/ Krantz K [Krantzkloof]” [handwritten label]; “Chlorolydella/ longirostris Villen./ L.P. Mesnil det., 1969”; “TYPE” [red label]; “EX/ L.-P. MESNIL/ COLLECTION 1970” (CNC).

###### Redescription.

*Body length*: 8.1–9.6 mm.

Male. *Colouration* (Fig. 12a): Head ground colour black except genal groove, face, facial ridge and frontal vitta which are red. Head covered with whitish-grey reflecting microtomentum. Scape, pedicel and arista brownish-black; postpedicel black. Palpus yellow. Thorax black (only tip of scutellum dark red), covered with light grey reflecting microtomentum. Presutural area of scutum with 4, well defined, dark vittae; postsutural area of scutum, when viewed from behind, with 5 dark vittae, 3 vittae (i.e., lateral pair continuous with those on presutural area and 1 mid-dorsal) extending along entire length of postsutural area and 2 on anterior portion only and continuous with median pair on presutural area. Femora and tarsi black, tibiae mostly red but darkened ventrally near junction with femur and tarsus. Tegula black; basicosta reddish-brown. Wing membrane hyaline. Abdomen mostly black, entirely covered with dense, irregularly tessellate, grey, reflecting microtomentum.

*Head* (Fig. 12a–b): Frons 1.3–1.5 times as wide as compound eye in dorsal view. Inner and outer vertical setae long and robust (outer vertical seta lateroclinate). Ocellar seta strong, proclinate. Fronto-orbital plate with a row of 7–8 frontal setae and 2 irregular rows of medioclinate setulae lateral to frontal setae. Frontal setae descending slightly below level of base of arista. Two upper reclinate orbital setae. Proclinate orbital setae absent. Parafacial flat, at its narrowest point about 1.5 times as wide as width of postpedicel. Facial ridge straight, with 1 row of robust, erect setae on lower 5/6; longest setae of facial ridge distinctly longer that width of postpedicel. Face concave. Postpedicel about 5 times as long as pedicel. Arista apparently bare, thickened on basal 1/2–2/3. First aristomere shorter than wide; second aristomere about as long as wide. Genal dilation well developed. Gena in profile about 0.25 times as high as compound eye. Occiput slightly convex. Lower occiput and postgena almost entirely covered with fine, pale setae. Palpus narrow, sub-cylindrical, 0.7 times as long as postpedicel. Prementum slender, 0.7–0.8 times as long as height of head; labella narrow and apically pointed.

*Thorax*: Four postpronotal setae, the 3 strong, basal setae arranged in a straight line; 1 strong anterior seta arising between inner and mid basal setae. Scutum with 3 + 3 acrostichal setae; 3 + 4 dorsocentral setae; 1 + 3 intra-alar setae; 1 or 2 inner and 1 outer posthumeral setae (as in Fig. 14a); 1 + 3 supra-alar setae (first postsutural supra-alar seta longer than first postsutural dorsocentral seta and longer than notopleural setae); notopleuron with 2 strong setae, subequal in size; postalar callus with 2 or 3 setae (if 3, then 1 is weaker than notopleural setae). Anatergite bare. Prosternum with several long setulae on lateral margin. Proepisternal depression bare. Katepimeron with 3–5 relatively long setulae on anterior 1/2–3/4. Three katepisternal setae (2+1) (Fig. 12a). Anterior and posterior lappets of metathoracic spiracle unequal in size (posterior lappet larger, operculum-like). Scutellum with 1 pair of crossed apical setae (standing almost horizontal), about 2/3 as long as subapical setae; 1 pair of subapical setae, 1 pair of lateral setae, and 1 pair of basal setae (a second smaller pair present in the holotype of *Phorocera majestica* Curran); lateral and basal setae subequal in size; 1 or 2 pairs of widely separated discal setae.

*Legs*: Fore tibia with 2 posterior setae. Preapical anterodorsal seta of fore tibia distinctly shorter than preapical dorsal seta. Fore claws at most as long as fifth tarsomere. Mid tibia with 2 anterodorsal setae. Submedian ventral seta of mid tibia present. Hind tibia with several anterodorsal setae, irregular in size (i.e., not forming a regular comb-like row). Preapical posteroventral seta of hind tibia distinctly shorter than preapical anteroventral seta. Hind tibia with 2 dorsal preapical setae.

*Wing*: Costal spine virtually indistinguishable from general costal setulae. Vein R_4+5_ with 3 setulae at base. Bend of vein M_1_ nearly right-angled; wing membrane weakly creased for a short distance distal to bend in the holotype of *Phorocera majestica*. Second costal section ventrally with a few setulae (only 1 on one side, probably not constant). Fourth costal section longer than sixth. Section of M_1_ between crossveins r-m and dm-m clearly longer than section between dm-m and bend of M_1_. Section of M_1_ between dm-m and bend of M_1_ shorter than postangular section of M_1_. Cell r_4+5_ narrowly open at wing margin. Wing membrane uniformly covered with microscopic setulae.

*Abdomen* (Figs 12a, 14c): Ventral edges of syntergite 1+2 and tergites 3 and 4 entirely overlapping the corresponding sternites. Mid-dorsal depression of syntergite 1+2 extending to hind margin of syntergite. Syntergite 1+2 and tergite 3 with 1 pair of median marginal setae; tergite 4 with a complete row of regular marginal setae; tergite 5 covered with erect setae, not arranged in rows. General setulae of tergites 3 and 4 dorsolaterally decumbent, changing to slightly raised mid-dorsally. Tergites 3–5 without sexual patches. Tergite 5 about 0.8–0.9 times as long as tergite 4.

Female. Unknown.

###### Distribution.

South Africa.

###### Remarks.

The male holotype of *Phorocera longirostris* Villeneuve from the former Cape Colony of South Africa has not been located. Cooper and O’Hara (1996: 62) treated a male specimen in CNC from Krantzkloof, South Africa as the holotype because it was labelled as “TYPE” by Mesnil. It is possible that Villeneuve erred when noting the type locality and this specimen is truly the holotype, but an equally plausible explanation and the one accepted here is that the holotype is missing and Mesnil labelled another specimen from Villeneuve’s collection as the type. There are other missing Villeneuve types and in time some of them may yet be found. We have elected not to treat the holotype of *Phorocera longirostris* as lost and thus not to designate a neotype to replace it, but we do accept the CNC specimen as conspecific based on the original description and Mesnil’s labelling. We recommend its designation as the neotype of *Phorocera longirostris* if such action is deemed necessary for nomenclatural stability in the future. The holotype of *Phorocera majestica* Curran is conspecific with the CNC specimen of *Phorocera longirostris* and the two names are newly treated as synonyms.

##### Myxophryxe murina

Taxon classificationAnimaliaDipteraTachinidae

Cerretti & O’Hara sp. n.

http://zoobank.org/C5A75E51-A4E2-4DC7-8041-CC95F3DE00AF

Figs 12c–d , 14d

###### Type material.

Holotype ♂: “Malaise trap/ mature/ Fynbos”; “RSA [Republic of South Africa]: Western Cape/ de Vaselot [error for de Vasselot] Nat[ural]. Res[erve]. at:/ 33°58.194′S 23°32.193′E/ 24–27.i.2009/ A. Kirk-Spriggs, S. Otto”; “Entomology Dept./ National Museum/ P.O. Box 265/ Bloemfontein 9300/ South Africa”; “BMSA (D)/ 0544” (NMB). Paratype ♂: same data as holotype but “BMSA (D)/ 0543” (MZUR).

###### Etymology.

The species epithet derives from the Latin adjective *murinus*, meaning mouse-grey, referring to the colouration of the species.

###### Description.

*Body length*: 9.8–10.4 mm.

Male differs from that of *Myxophryxe longirostris* as follows:

*Colouration* (Figs 12c–d, 14d): Head ground colour black except genal groove and face, which are brownish-red. Microtomentum of head, thorax and abdomen yellowish-grey with golden reflections. Posterior 1/3 of scutellum reddish-brown. Antenna black. Palpus brown on proximal 2/3, shading into yellowish on distal 1/3. Legs black. Basicosta brownish-black. Abdomen black, entirely covered with dense, irregularly tessellate microtomentum.

*Head* (Fig. 12c–d): Frons 1.1–1.2 times as wide as compound eye in dorsal view. Fronto-orbital plate with a row of 7–8 frontal setae descending distinctly below level of base of arista. Parafacial slightly convex, at its narrowest point 1.2–1.3 times as wide as width of postpedicel. Facial ridge slightly convex, with 1 row of robust, erect setae on lower 3/4–4/5; longest setae of facial ridge about as long as width of postpedicel. Postpedicel 4.5–6.3 times as long as pedicel. Gena in profile 0.3–0.4 times as high as compound eye. Palpus slightly clubbed, 0.6–0.7 times as long as postpedicel. Prementum normal, 0.3–0.5 times as long as height of head (3.7–5.0 times as long as wide); labella normally developed and not pointed apically.

*Thorax*: Four or 5 postpronotal setae, 3 strong, basal setae arranged in a straight line; 1 strong anterior seta arising between inner and mid basal setae or in front of mid basal one; 1 smaller anterior seta (when present) arising in front of inner basal seta. Katepimeron with 1–3 short setulae on anterior 1/4.

*Legs*: Preapical anterodorsal seta of fore tibia about as long as preapical dorsal seta. Hind tibia with 3 dorsal preapical setae (mid-dorsal one distinctly shorter that anterodorsal preapical and posterodorsal preapical setae).

*Wing*: Costal spine well developed, at least as long as crossvein r-m. Second costal section ventrally bare.

*Abdomen* (Figs 12c, 14d): General setulae of tergites 3 and 4 slightly raised laterally and mid-dorsally. Tergite 5 0.9–1.0 times as long as tergite 4.

Female. Unknown.

###### Distribution.

South Africa.

##### Myxophryxe regalis

Taxon classificationAnimaliaDipteraTachinidae

Cerretti & O’Hara sp. n.

http://zoobank.org/A49A9629-494D-4C72-B390-67FD521BFDC7

Figs 13a–b , 14a, e

###### Type material.

Holotype ♂: “Malaise traps/ *Leucosedea* [error for *Leucosidea*] -/ dominated scrub”; “RSA [Republic of South Africa]: KZN [KwaZulu-Natal], Royal Natal N[ational]. P[ark]./ Thendele, 1600 m/ 28°42.378′S 28°56.083′E/ 15–17.ii.2010/ A.H. Kirk-Spriggs”; “Entomology Dept./ National Museum/ P.O. Box 265/ Bloemfontein 9300/ South Africa”; “BMSA (D)/ 20315” (NMB). Paratype ♂: same data as holotype but “BMSA (D)/ 20312” (MZUR).

###### Etymology.

The species epithet derives from the latin adjective *regalis*, meaning royal.

###### Description.

*Body length*: 8.5–9.6 mm.

Male differs from that of *Myxophryxe longirostris* as follows:

*Colouration* (Figs 13a–b, 14e): Head ground colour black except genal groove, face and facial ridge which are red; frontal vitta blackish-brown. Scape, pedicel and arista blackish. Palpus basally brown, shading into yellow on distal 1/2. Thorax and legs black. Basicosta blackish-brown.

*Head* (Fig. 13a–b): Frons about 1.3 times as wide as compound eye in dorsal view. Outer vertical seta weakly developed and not or only barely distinguishable from postocular setae. Two or 3 upper reclinate orbital setae. Fronto-orbital plate with a row of 9–10 frontal setae descending to about level of base of arista. Parafacial slightly convex, at its narrowest point 1.8–2.2 times as wide as width of postpedicel. Facial ridge convex, with 2 rows of robust, erect setae on lower 5/6 (lateral row consisting of shorter setae); longest setae of facial ridge distinctly shorter than width of postpedicel. Postpedicel 3.9–4.3 times as long as pedicel. Gena in profile 0.4–0.5 times as high as compound eye. Palpus narrow, sub-cylindrical or slightly clubbed, 0.7 times as long as postpedicel. Prementum normally developed, 0.3–0.4 times as long as height of head; labella normally developed, not pointed.

*Thorax*: Four postpronotal setae, 3 strong, basal setae arranged in a straight line; 1 anterior seta arising almost in front of inner basal seta. Katepimeron bare. Apical scutellar setae convergent or crossed only at tips.

*Legs*: Fore claws broken off on both specimens (pulvilli about as long as fifth tarsomere).

*Wing*: Costal spine about as long as crossvein r-m. Second costal section ventrally bare. Cell r_4+5_ open at wing margin.

*Abdomen* (Fig. 14e): Tergite 5 about 0.85–0.90 times as long as tergite 4.

Female. Unknown.

###### Distribution.

South Africa.

##### Myxophryxe satanas

Taxon classificationAnimaliaDipteraTachinidae

Cerretti & O’Hara sp. n.

http://zoobank.org/936FC9CB-EA99-4AA2-BF94-8F000F846182

Figs 13c–d , 14b, f

###### Type material.

Holotype ♂: “South Africa: Western Cape/ Gamkaskloof (Die Hel) at:/ 33°21′49.60″S 21°37′40.97″E/ 16–18.x.2012, 336 m/ P. Cerretti, J. Stireman, J. O’Hara,/ I. Winkler & A.H. Kirk-Spriggs”; “SA044” [voucher ID] (MZUR).

###### Remarks.

Fore and mid right legs were removed from the fresh specimen and stored in pure ethanol in a vial for DNA extraction and sequencing (preserved at Wright State University, OH, USA as part of the project “Phylogeny and Evolution of World Tachinidae (Diptera)” funded by the U.S. National Science Foundation, grant number DEB-1146269).

###### Etymology.

The species epithet derives from the Latin noun *Sătănās*, meaning devil, and is inspired by the type locality “Die Hel”.

###### Description.

*Body length*: 10.6 mm.

Male differs from that of *Myxophryxe longirostris* as follows:

*Colouration* (Figs 13c–d, 14b, f): Frontal vitta blackish-brown. Scape and pedicel yellowish-red, arista black. Thorax ground colour black (including scutellum). Legs entirely black, only a little reddish at junction between femora and tibiae. Basicosta blackish-brown. Abdomen entirely black, dorsally mostly covered with dense, non tessellate, whitish reflecting microtomentum with 2 sagittally symmetrical, large, black spots on posteromedian portions of tergites 3–5 including bases of median marginal setae; small dark spots present also around other marginal setae of tergite 4 (Fig. 14f); ventral surface of abdomen in posteroventral view largely shiny black, mostly whitish microtomentose in lateral view (Fig. 13c).

*Head* (Fig. 13c–d): Frons 1.6 times as wide as compound eye in dorsal view. Fronto-orbital plate with a row of 7–8 frontal setae and 1 irregular row of medioclinate short setulae lateral to frontal setae. Frontal setae descending below level of base of arista. Parafacial slightly convex, at its narrowest point 1.6 times as wide as width of postpedicel. Facial ridge slightly convex, with 1 or 2 rows of robust, erect setae on its whole length; longest setae of facial ridge distinctly longer than width of postpedicel. Face concave. Postpedicel 4.3 times as long as pedicel. Gena in profile about 0.4 times as high as compound eye. Palpus narrow, very slightly clubbed, 0.8 times as long as postpedicel. Prementum slender, about 0.6 times as long as height of head; labella narrow and apically pointed.

*Thorax*: Scutum with 0 + 3 intra-alar setae (first postsutural intra-alar very short, about 1/2 the length of second postsutural intra-alar seta); 1 posthumeral seta (i.e., outer posthumeral seta absent) (Fig. 14b). Katepimeron bare. Scutellum with 1 pair of crossed apical setae (standing almost horizontal), about 2/3–3/4 as long as subapical setae; 1 pair of widely separated discal setae.

*Legs*: Preapical anterodorsal seta of fore tibia about as long as preapical dorsal seta. Mid tibia with 2 strong anterodorsal setae, a third shorter anterodorsal seta present proximally. Hind tibia with 2 strong dorsal preapical setae subequal in size, and a third in anterodorsal position less than 1/2 as long as the others.

*Wing*: Vein R_1_ with 1 setula dorsally only on right wing. Second costal section ventrally bare.

*Abdomen* (Figs 13c, 14f): Tergite 5 with irregular rows of erect marginal and discal setae. General setulae of tergites 3 and 4 decumbent. Tergite 5 about as long as tergite 4.

Female. Unknown.

###### Distribution.

South Africa.

##### Key to males of the species of *Myxophryxe* gen. n.

**Table:** 

1	Outer posthumeral seta absent (Fig. 14b). Anterodorsal preapical setae of fore tibia about as long as preapical dorsal seta	**2**
–	Outer posthumeral seta present (Fig. 14a, red circle). Anterodorsal preapical setae of fore tibia distinctly shorter than preapical dorsal seta	**3**
2	Presutural intra-alar setae absent (Fig. 14b). Prementum long and slender, about 0.6 times as long as height of head; labella narrow and apically pointed (Fig. 13d). Abdomen dorsally mostly covered with dense, non tessellate, whitish reflecting microtomentum with 2 sagittally symmetrical, large, black spots on posteromedian portions of tergites 3–5 (Fig. 14f). Hind tibia with 2 dorsal preapical setae	***Myxophryxe satanas* sp. n.**
–	Presutural intra-alar seta present (as in Fig. 14a, yellow circle). Prementum normally developed, 0.3–0.5 times as long as height of head; labella normally developed and not pointed apically (Fig. 12d). Abdomen entirely covered with yellowish-grey reflecting microtomentum, irregularly tessellate (Fig. 14d). Hind tibia with 3 dorsal preapical setae	***Myxophryxe murina* sp. n.**
3	Facial ridge convex, with 2 rows of robust, erect setae (lateral row consisting of shorter setae); longest setae of facial ridge distinctly shorter than width of postpedicel (Fig. 13b). Parafacial slightly convex, at its narrowest point 1.8–2.2 times as wide as width of postpedicel. Prementum normally developed, 0.3–0.4 times as long as height of head; labella normally developed, not pointed (Fig. 13b). Gena in profile 0.4–0.5 times as high as compound eye	***Myxophryxe regalis* sp. n.**
–	Facial ridge straight, with one row of robust, erect setae; longest setae of facial ridge distinctly longer than width of postpedicel (Fig. 12b). Parafacial flat, at its narrowest point about 1.5 times as wide as width of postpedicel. Prementum slender, 0.7–0.8 times as long as height of head; labella narrow and apically pointed (Fig. 12b). Gena in profile about 0.25 times as high as compound eye	***Myxophryxe longirostris* (Villeneuve)**

##### Stiremania

Taxon classificationAnimaliaDipteraTachinidae

Cerretti & O’Hara gen. n.

http://zoobank.org/1BE107E1-FB25-410F-971C-9D59CECC596A

Figs 15 , 16

###### Type species.

*Stiremania karoo* Cerretti and O’Hara sp. n., by present designation.

###### Etymology.

Dedicated to our friend and colleague John O. Stireman III (Dayton, Ohio, USA).

###### Diagnosis.

**Figure 15.**
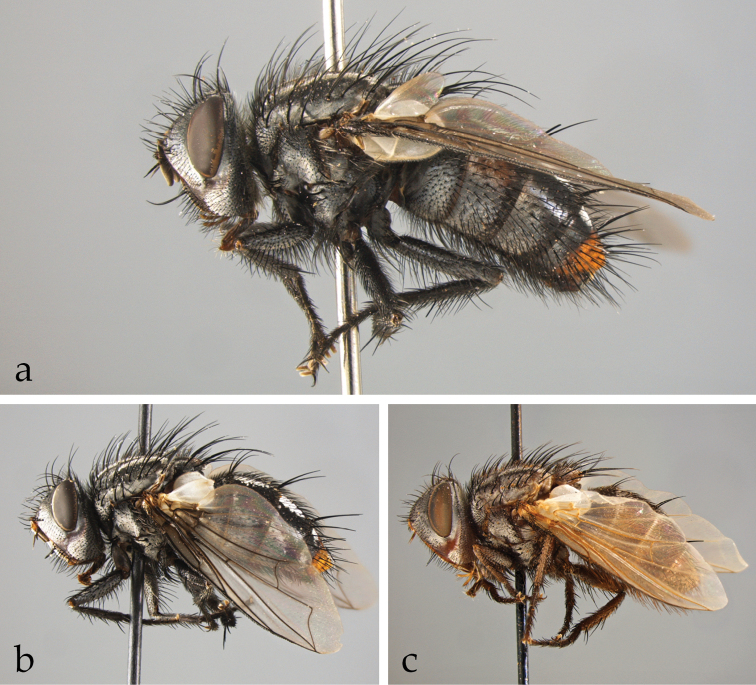
*Stiremania* Cerretti & O’Hara, gen. n. **a–b** habitus in lateral view of *Stiremania karoo* Cerretti & O’Hara, sp. n. **a** male holotype (MZUR) **b** female paratype (MZUR) **c** *Stiremania robusta* Cerretti & O’Hara, sp. n., habitus in lateral view (male holotype, NMDA).

**Figure 16.**
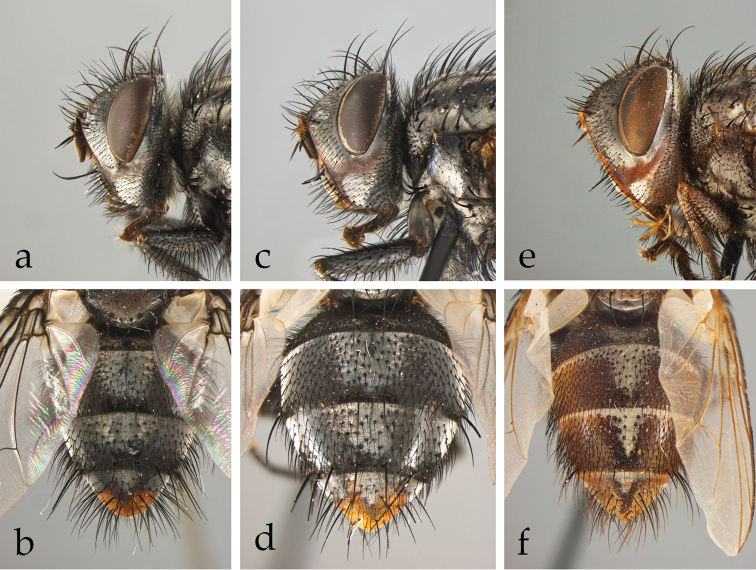
*Stiremania* Cerretti & O’Hara, gen. n. **a–b** *Stiremania karoo* Cerretti & O’Hara, sp. n. (male holotype, MZUR) **a** head in lateral view **b** abdomen in dorsal view **c–d** *Stiremania karoo* (female paratype, MZUR) **c** head in lateral view **d** abdomen in dorsal view **e–f** *Stiremania robusta* Cerretti & O’Hara, sp. n. (male holotype, NMDA) **e** head in lateral view **f** abdomen in dorsal view.

Compound eye nearly bare (scattered ommatrichia, when present, shorter than diameter of two eye facets). Ocellar seta well developed, proclinate. Frons broad, wider than compound eye in dorsal view. Two upper reclinate orbital setae. Parafacial broad, convex and entirely covered with short, black setulae. Face varying from moderately to deeply concave. Facial ridge straight or slightly concave, with fine, decumbent setae on lower 1/5 of its length. Lower facial margin not visible in lateral view. Lower occiput and postgena covered with mostly pale hair-like setulae. Vibrissa arising well above level of lower facial margin; subvibrissal ridge well developed, with a row of 4–5 subvibrissal setae subequal in size. Antenna short, at most as long as height of gena (Figs 15, 16a, c, e). Arista apparently bare; arista short and thickened on proximal 3/4. Palpus cylindrical. Prosternum with some setulae along lateral margin. Proepisternal depression bare. Proepisternal seta present. Postpronotum with 4 setae, the 3 strongest basal ones arranged in a line. Katepimeron bare. Three katepisternal setae (2+1). Three postsutural intra-alar setae. First postsutural supra-alar seta longer than notopleural setae and longer and stronger than first postsutural intra-alar seta. Four postsutural dorsocentral setae. Scutellum with 4 pairs of marginal setae and 1 pair of discal setae: apical scutellar setae crossed or sub-parallel, horizontal or slightly tilted upwards. Wing cell r_4+5_ closed at wing margin, short petiolate or M_1_ vein vanishing on membrane before reaching wing margin (Figs 15c, 16f). Wing membrane uniformly covered with microscopic setulae. Mid tibia with at least 3 anterodorsal setae and a strong submedian ventral seta. Hind coxa bare posterodorsally. Mid-dorsal depression of abdominal syntergite 1+2 reaching posterior margin of syntergite. Syntergite 1+2 and tergite 3 with 1 pair of median marginal setae. Tergite 4 with a complete row of marginal setae. Tergites 3 and 4 without median discal setae.

###### Remarks.

A robust body, broad head and wide parafacial covered with short setae make specimens of *Stiremania* easily mistaken for those of *Sturmiopsis* and *Pseudalsomyia*. However, *Sturmiopsis* is characterized by having the antenna distinctly longer than height of the gena, parafacial not wider than width of postpedicel, two katepisternal setae, and mid tibia with two anterodorsal setae. Also, females of *Sturmiopsis* species produce macrotype, membranous eggs, thus placing the genus in the tribe Eryciini. Females of *Stiremania karoo*, on the other hand, produce microtype, planoconvex eggs, which is the reproductive strategy of goniines. Within the Goniini, *Stiremania* is similar and perhaps closely related to *Pseudalsomyia* with which it shares, in addition to the character states it shares with both *Sturmiopsis* and *Pseudalsomyia*, a very short and narrow antenna and broadly convex parafacial. *Pseudalsomyia* differs from *Stiremania* by having one upper reclinate orbital seta, vibrissa almost indistinct from setae on facial ridge, two lateral scutellar setae, two katepisternal setae, mid tibia with one anterodorsal seta, and male possessing sexual patches on abdominal tergites 3 and 4. We have determined that the two new species described below do not fit within the limits of an existing genus and propose for them the new genus *Stiremania*.

##### Stiremania karoo

Taxon classificationAnimaliaDipteraTachinidae

Cerretti & O’Hara sp. n.

http://zoobank.org/7BADC63C-3B54-4783-B26C-47273E2A39B1

Figs 15a–b , 16a–d

###### Type material.

Holotype ♂: “South Africa: Western Cape/ Gamkaskloof (Die Hel) at:/ 33°22′5.90″S 21°37′19.43″E/ 17–18.x.2012, 336 m (hilltop)/ P. Cerretti, J. Stireman, J. O’Hara,/ I. Winkler & A.H. Kirk-Spriggs”; “SA033” [voucher ID] (MZUR). Paratype ♀: same data and depository as holotype.

###### Remarks.

The mid and hind right legs of the holotype and paratype were removed from the fresh specimens and stored in pure ethanol in a vial for DNA extraction and sequencing (preserved at Wright State University, OH, USA as part of the project “Phylogeny and Evolution of World Tachinidae (Diptera)” funded by the U.S. National Science Foundation, grant number DEB-1146269).

###### Etymology.

The specific epithet is a noun in apposition. Named after the Karoo region.

###### Description.

*Body length*: 8–9 mm.

Male. *Colouration* (Figs 15a, 16a–b): Head ground colour black except genal groove, which is dark brown. Head covered with grey microtomentum, more reflecting on parafacial than fronto-orbital plate. Antenna black. Palpus reddish-yellow. Thorax black (only apical 1/2–1/3 of scutellum dark red), covered with grey reflecting microtomentum. Presutural area of scutum with 4, not well defined, dark vittae; postsutural area of scutum, when viewed from behind, with 4 dark vittae, 2 vittae (i.e., lateral pair continuous with those on presutural area) extending along entire length of postsutural area and 2 on anterior portion only and continuous with median pair on presutural area. Legs black. Tegula and basicosta black. Wing membrane hyaline, veins brownish-black. Abdomen mostly black with posterior 1/2–3/4 of tergite 5 reddish-yellow (Fig. 16b), covered with irregularly tessellate grey reflecting microtomentum.

*Head* (Figs 15a, 16a): Frons 1.2 times as wide as compound eye in dorsal view. Inner vertical setae well developed, reclinate. Outer vertical seta not differentiated from postocular setae. Ocellar seta strong, proclinate. Fronto-orbital plate with a row of 8–10 frontal setae and several fine medioclinate setulae lateral to frontal setae. Frontal setae descending to about level of distal margin of pedicel. Two upper reclinate orbital setae. Proclinate orbital setae absent. Parafacial convex, at its narrowest point about 2.6 times as wide as width of postpedicel. Face moderately concave, antennae only partly hidden from view in profile (Figs 15a, 16a). Facial ridge concave, with a few decumbent setulae on lower 1/5. Postpedicel about 2.1 times as long as pedicel. Arista short, apparently bare, thickened on basal 4/5 to tip. First aristomere shorter than wide; second aristomere about as long as wide. Genal dilation well developed. Gena in profile about 0.6 times as high as compound eye. Occiput slightly convex. Upper occiput with 1 or 2 irregular rows of black setulae behind postocular row. Lower occiput and postgena almost entirely covered with fine, pale setae. Genal dilation with black setulae only. Palpus narrow, sub-cylindrical, 1.2–1.5 times as long as postpedicel, with setulae along whole length. Prementum short, about 0.2–0.3 times as long as height of head; labella normally developed.

*Thorax*: Four postpronotal setae, the 3 strong, basal setae arranged in a straight line; 1 strong anterior seta arising between inner and mid basal setae. Scutum with 3 + 3 acrostichal setae; 3 + 4 dorsocentral setae; 1 + 3 intra-alar setae; 1 or 2 inner and 1 outer posthumeral setae; 1 + 3 supra-alar setae (first postsutural supra-alar seta longer than first postsutural dorsocentral seta and longer than notopleural setae); notopleuron with 2 strong setae, subequal in size; postalar callus with 2 or 3 setae (if 3, then 1 is weaker than notopleural setae). Anatergite bare. Prosternum with several long setulae on lateral margin. Proepisternal depression bare. Katepimeron bare. Three katepisternal setae (2+1). Anterior and posterior lappets of metathoracic spiracle unequal in size (posterior lappet larger, operculum-like). Scutellum with 1 pair of crossed apical setae (standing almost horizontal), 1/2–2/3 as long as subapical setae; 1 pair of subapical setae, 1 or 2 pairs of lateral setae, and 1 pair of basal setae; lateral and apical setae subequal in size; 1 or 2 pairs of discal setae (medial pair convergent or apically crossed).

*Legs*: Fore tibia with 2 posterior setae. Preapical anterodorsal seta of fore tibia about 4/5 the length of preapical dorsal seta. Fore claws about 1.2 times as long as fifth tarsomere. Mid tibia with 3–5 anterodorsal setae (2 distinctly longer than the others). Submedian ventral seta of mid tibia present. Hind tibia with several anterodorsal setae, more or less regular in size, with 1 longer seta arising at about midlength. Preapical posteroventral seta of hind tibia distinctly shorter than preapical anteroventral seta. Hind tibia with 2 dorsal preapical setae.

*Wing*: Costal spine virtually indistinguishable from general costal setulae. Vein R_4+5_ with 4–5 setulae at base. Vein M_1_ complete (i.e., reaching wing margin). Bend of vein M_1_ obtuse-angled. Second costal section ventrally bare. Fourth costal section longer than sixth. Section of M_1_ between crossveins r-m and dm-m clearly longer than section between dm-m and bend of M_1_. Section of M_1_ between dm-m and bend of M_1_ shorter than postangular section of M_1_. Cell r_4+5_ closed at wing margin or short petiolate.

*Abdomen* (Figs 15a, 16b): Ventral edges of syntergite 1+2 and tergites 3 and 4 entirely overlapping the corresponding sternites. Syntergite 1+2 and tergite 3 with 1 pair of median marginal setae; tergite 4 with a complete row of regular marginal setae; reddish-yellow portion of tergite 5 covered with erect setae, not arranged in rows. General setulae of tergites 3 and 4 dorsolaterally decumbent, changing to slightly raised mid-dorsally. Tergites 3–5 without sexual patches. Tergite 5 about 0.8–0.9 times as long as tergite 4.

Female (Figs 15b, 16c–d) differs from male as follows. Scape and pedicel yellow. Frons 1.3 times as wide as compound eye in dorsal view. Fronto-orbital plate with 2 proclinate orbital setae. Parafacial convex, at its narrowest point about 3.0 times as wide as width of postpedicel. Postpedicel about 1.8 times as long as pedicel. Fore claws distinctly shorter than fifth tarsomere.

###### Distribution.

South Africa.

##### Stiremania robusta

Taxon classificationAnimaliaDipteraTachinidae

Cerretti & O’Hara sp. n.

http://zoobank.org/E99E8779-E219-4342-AF06-9C9D478E8139

Figs 15c , 16e–f

###### Type material.

Holotype ♂: “Capland/ Willowmore/ März 1935/ Dr. Brauns” (NMDA).

###### Etymology.

The specific epithet derives from the Latin adjective *robustus* meaning stout, alluding to the robustness of this species, mostly due to its thick, short legs.

###### Description.

*Body length*: 8–9 mm.

Male differs from that of *Stiremania karoo* as follows:

*Colouration* (Figs 15c, 16e–f): Fronto-orbital plate, parafacial and upper occiput blackish-brown; frontal vitta brown; lower occiput, postgena, gena, genal groove, facial ridge, and face yellowish-red in ground colour. Head covered with grey microtomentum, denser on parafacial than fronto-orbital plate. Antenna yellow. Palpus yellow. Thorax mostly brown especially on scutum, largely reddish-yellow on pleura. Legs mostly brownish-yellow. Basicosta yellow. Wing membrane hyaline, veins yellowish. Abdomen yellowish-red ventrally, laterally and dorsolaterally, shading into brown dorsomedially. Syntergite 1+2 microtomentose only on mid-dorsal depression; tergites 3–5 with a narrow basal band of whitish-grey reflecting microtomentum, which is medially expanded into a triangular prolongation almost reaching posterior margins of tergites (Fig. 16f).

*Head* (Figs 15c, 16e): Frontal setae descending to about level of middle of pedicel. Parafacial convex, at its narrowest point about 3–4 times as wide as width of postpedicel. Face deeply concave, antennae entirely hidden from view in profile. Facial ridge straight, with a few decumbent setulae on lower 1/5. Upper occiput with 1 irregular row of black setulae behind postocular row. Palpus sub-cylindrical, about as long as postpedicel, with several setulae along whole length. Prementum short, about 0.20 times as long as height of head.

*Thorax*: Two inner and 1 outer posthumeral setae. Apical scutellar setae erect and subparallel; 2 pairs of lateral scutellar setae.

*Legs*: Fore tibia with 2 posterior setae. Preapical anterodorsal seta of fore tibia about 2/3 the length of preapical dorsal seta. Fore claws about 0.8–0.9 times as long as fifth tarsomere. Preapical posteroventral seta of hind tibia well developed and about as long as preapical anteroventral seta.

*Wing*: Bend of vein M_1_ obtuse-angled; postangular section of M_1_ incomplete, being very faint from about halfway between bend and wing margin, then vanishing into the membrane and not reaching wing margin (Figs 15c, 16f). Section of M_1_ between crossveins r-m and dm-m clearly longer than section between dm-m and bend of M_1_. Section of M_1_ between dm-m and bend of M_1_ longer than postangular section of M_1_.

*Abdomen* (Fig. 16f): General setulae of tergites 3–5 dorsally short and decumbent; ventrally long and suberect. Tergites 4 and 5 with a symmetrical pair of sexual patches consisting of a carpet of curled microtrichia: in dorsal position on tergite 4, in dorsolateral position on tergite 5. Tergite 5 about 0.7 times as long as tergite 4.

Female. Unknown.

###### Distribution.

South Africa.

##### Key to species of *Stiremania* gen. n.

**Table:** 

1	Postpedicel black. Abdomen mostly black with posterior 1/2–3/4 of tergite 5 reddish-yellow (Figs 15a–b, 16b, d), covered with irregularly tessellate grey reflecting microtomentum. Basicosta black. Vein M_1_ complete (i.e., reaching wing margin) and postangular section of M_1_ normal, similar in thickness to adjacent veins. Male: Fore claws about 1.2 times as long as fifth tarsomere; abdominal tergites 4 and 5 without sexual patches	***Stiremania karoo* sp. n.**
–	Postpedicel yellow. Abdomen yellowish-red ventrally, laterally and dorsolaterally, shading into brown dorsomedially. Syntergite 1+2 microtomentose only on mid-dorsal depression; tergites 3–5 with a narrow band of whitish-grey reflecting microtomentum basally, medially expanding into a triangular prolongation (Fig. 16f). Basicosta yellow. Vein M_1_ incomplete, postangular section very faint from about halfway between bend and wing margin, then vanishing into the membrane (Figs 15c, 16f). Male: Fore claws about 0.8–0.9 times as long as fifth tarsomere; abdominal tergites 4 and 5 with sexual patches	***Stiremania robusta* sp. n.**

#### Tachininae, Leskiini

##### Austrosolieria

Taxon classificationAnimaliaDipteraTachinidae

Cerretti & O’Hara gen. n.

http://zoobank.org/BDA16828-0545-4EDB-B57A-6A0CA8746C63

Figs 17 , 18

###### Type species.

*Austrosolieria londti* Cerretti & O’Hara, sp. n., by present designation.

###### Etymology.

*Austrosolieria* is a composite word formed from the prefix of the Latin adjective *austrīnus*, meaning southern, and the generic name *Solieria* Robineau-Desvoidy, which is morphologically similar.

###### Diagnosis.

Compound eye bare. Ocellar setae well developed, proclinate. Frons 1.2–1.6 times as wide as compound eye in dorsal view. Parafacial bare, convex, at its narrowest point 1.1–1.3 times as wide as width of postpedicel. Facial ridge convex (slightly concave just above vibrissa), with 2–3 fine setulae on lower 1/6. Lower facial margin not warped forward and not visible in lateral view. Postpedicel sub-rectangular (more or less sharply pointed at apex in male), about 1.4–1.6 times as long as pedicel. Arista apparently bare; thickened on approximately basal 1/4. First aristomere shorter than wide; second aristomere about as long as wide. Genal dilation well developed, with several strong setae on anterior 1/2. Gena in profile 0.4–0.6 times as high as compound eye. Lower occiput and postgena covered with mostly pale hair-like setulae. Upper occiput with at least a few black occipital setulae. Vibrissa well developed, arising at level of lower facial margin. Prementum short and relatively narrow, palpus clubbed, well developed. Prosternum and proepisternal depression bare. Proepisternal seta present, well developed. Postpronotum with 2–5 setae. Katepimeron bare. Three katepisternal setae (2+1). Presutural intra-alar seta absent. Two or 3 postsutural intra-alar setae (if 2, then setae separated by a distance less than that between first seta and transverse suture). First postsutural supra-alar seta shorter than notopleural setae and first postsutural dorsocentral seta. Two or 3 presutural and 3 postsutural dorsocentral setae. Zero to 2 presutural acrostichal setae. Scutellum with 2 pairs of strong, slightly diverging marginal setae subequal in size: basal and subapical. Costal spine strong, 1.5–3.5 times as long as crossvein r-m (Fig. 18g–h). Second costal section setulose ventrally. Veins R_1_ and M_1_ bare. Base of vein R_4+5_ with 2–3 strong setulae or a tuft of setulae. Wing cell r_4+5_ closed at wing margin or nearly so. Bend of vein M_1_ obtuse. Fore tibia with 2 posterior setae. Preapical anterodorsal seta of fore tibia longer than preapical dorsal seta. Mid tibia with 2–4 anterodorsal setae and a strong submedian ventral seta. Preapical posteroventral seta of hind tibia at most 1/2 as long as preapical anteroventral seta. Hind coxa bare posterodorsally. Mid-dorsal depression of abdominal syntergite 1+2 reaching posterior margin of syntergite. Syntergite 1+2 and tergite 3 with 1 pair of median marginal setae. Tergites 4 and 5 with a complete row of marginal setae. Tergites 3–5 without median discal setae.

**Figure 17.**
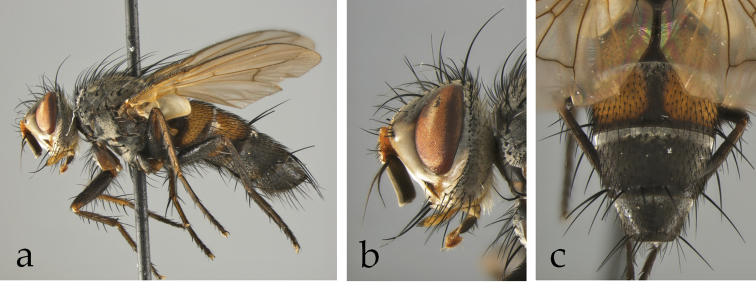
*Austrosolieria londti* Cerretti & O’Hara, sp. n. (male holotype, NMDA) **a** habitus in lateral view **b** head in lateral view **c** abdomen in dorsal view.

**Figure 18.**
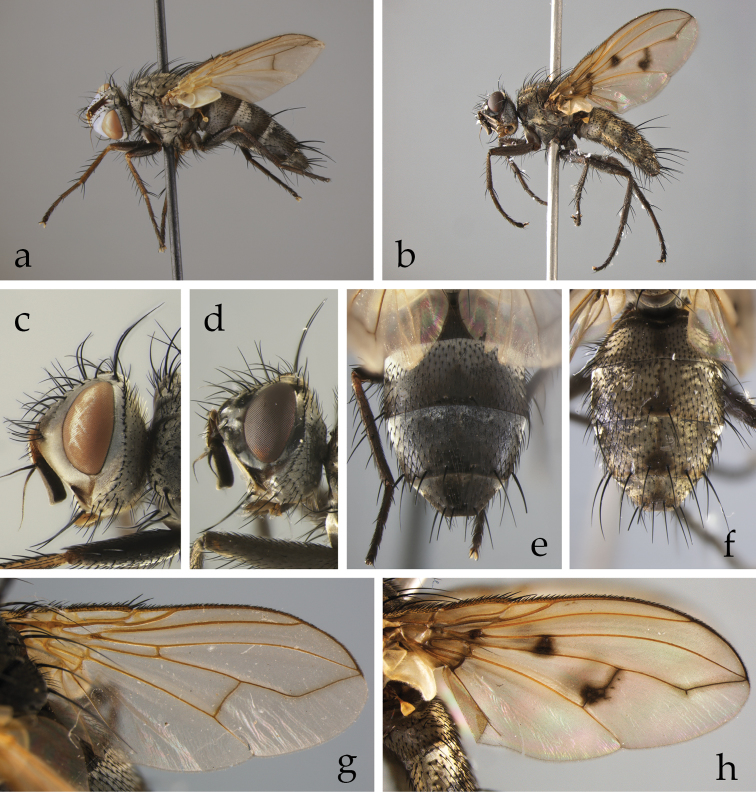
*Austrosolieria* Cerretti & O’Hara, gen. n. **a** *Austrosolieria londti* Cerretti & O’Hara, sp. n., habitus in lateral view (female paratype, NMDA) **b** *Austrosolieria freidbergi* Cerretti & O’Hara, sp. n., habitus in lateral view (female holotype, TAU) **c–d** head in lateral view **c** *Austrosolieria londti* **d** *Austrosolieria freidbergi* **e–f** abdomen in dorsal view **e** *Austrosolieria londti* **f** *Austrosolieria freidbergi* **g–h** wing **g** *Austrosolieria londti* **h** *Austrosolieria freidbergi*.

###### Remarks.

To our knowledge, species of *Austrosolieria* are not easily confused with those of any other Afrotropical genus. However, the habitus of *Austrosolieria* species and the combination of a wide frons, bare prosternum, three postsutural dorsocentral setae, two strong marginal scutellar setae (subapical and basal), hind tibia with short and weak preapical posteroventral seta, and costal spine well developed, are reminiscent of the Palaearctic genera *Solieria*, *Bithia* Robineau-Desvoidy, and, in part, *Clausicella* Rondani. *Austrosolieria* differs from these by having the lower facial margin not protruding and not visible in lateral view, fore tibia with preapical anteroventral seta distinctly longer than preapical dorsal seta, and postpedicel more or less sharply pointed at apex in male. We have determined that the two new species described below do not fit within the limits of an existing tachinid genus and propose for them the new genus *Austrosolieria*.

##### Austrosolieria freidbergi

Taxon classificationAnimaliaDipteraTachinidae

Cerretti & O’Hara sp. n.

http://zoobank.org/69EA1FA6-9FA5-4BD6-8EC3-BFF0743E1582

Fig. 18b, d, f, h

###### Type material.

Holotype ♀: 66076. MALAWI:/ Nyika National Park/ forest, 15km N Chelinda/ 10°30.1′S 33°48.8′E/ 29.xii.2009 2368m/ A. FREIDBERG (TAU).

###### Etymology.

Dedicated to our colleague Amnon Freidberg (TAU), who collected the holotype.

###### Description.

*Body length*: ca. 7 mm.

Female. *Colouration* (Fig. 18b, d, f, h): Head ground colour black. Head covered with grey, iridescent reflecting microtomentum: head when seen in anterodorsal view with a dark spot on upper parafacial between lowest frontal seta and compound eye margin; when seen in anteroventral view, parafacial appearing dark with two grey reflecting spots, one on lowest corner of parafacial and one between lowest frontal seta and compound eye margin (corresponding to dark spot visible in anterodorsal view). Antenna black. Palpus reddish-yellow. Thorax black in ground colour, with grey reflecting microtomentum. Presutural area of scutum with 3 broad dark vittae; postsutural area of scutum, when viewed from behind, more or less uniformly dark. Legs black. Tegula reddish-brown; basicosta yellow. Wing membrane mostly hyaline except for dark infuscations around crossveins r-m and dm-m, and a slightly smoky area along postangular section of M_1_. Abdomen black, covered with irregularly tessellate grey reflecting microtomentum.

*Head* (Fig. 18b, d): Frons about 1.6 times as wide as compound eye in dorsal view. Inner vertical seta well developed, reclinate. Outer vertical seta well developed. Ocellar seta proclinate. Fronto-orbital plate with a row of 7–8 frontal setae descending to about level of middle of pedicel. One weak upper lateroclinate orbital seta. Fronto-orbital plate with 2 proclinate orbital setae and a few short setulae lateral to frontal row. Parafacial convex, at its narrowest point about 1.2 times as wide as width of postpedicel. Face moderately concave, antennae not concealed from view in profile. Facial ridge concave, with 1–2 decumbent setulae just above vibrissa. Postpedicel subrectangular with dorsoapical tip pointed, about 1.5 times as long as pedicel. Arista thickened on basal 1/4–1/3. Genal dilation well developed with robust setae anteriorly. Gena in profile about 0.6 times as high as compound eye. Occiput convex. Upper occiput with 1 or 2 irregular rows of black setulae behind postocular row. Lower occiput and postgena with a few fine, pale setulae. Palpus strongly clubbed and covered with stout setulae; palpus about twice the length of prementum. Prementum short and labella reduced.

*Thorax*: Two postpronotal setae. Scutum with 1 + 0 acrostichal setae; 2 + 3 dorsocentral setae; 0 + 2 intra-alar setae (distance between postsutural intra-alar setae less than distance between anterior seta and transverse suture); 1 (inner) posthumeral seta; 1 + 3 supra-alar setae; notopleuron with 2 strong setae, subequal in size; postalar callus with 2 setae. Anatergite bare. Metathoracic spiracle small and rounded, anterior and posterior lappets subequal in size.

*Legs*: Fore tibia with 4 anterodorsal setae. Fifth fore tarsomere enlarged, ovoid. Fore claws shorter than fifth tarsomere. Mid tibia with 4 anterodorsal setae (median 2 distinctly longer than the others). Hind tibia with 2–4 (asymmetrical) anterodorsal setae, unequal in size. Hind tibia with 2 dorsal preapical setae.

*Wing*: Base of vein R_4+5_ with a tuft of 5–10 setulae. Bend of vein M_1_ obtuse, with a short appendix. Section of M_1_ between crossveins r-m and dm-m about as long as section between dm-m and bend of M_1_. Section of M_1_ between dm-m and bend of M_1_ longer than postangular section of M_1_. Cell r_4+5_ closed at wing margin. Wing membrane uniformly covered with microscopic setulae. Crossvein r-m with two stubs; crossvein dm-m not linear; i.e., developed into a sieve-like shape (Fig. 18h) [this may be teratological, though present in both wings].

###### Distribution.

South Africa.

##### Austrosolieria londti

Taxon classificationAnimaliaDipteraTachinidae

Cerretti & O’Hara sp. n.

http://zoobank.org/182132B1-26C0-4BF2-924E-5B797EF0335A

Figs 17 , 18a, c, e, g

###### Type material.

Holotype ♂: S[ou]TH AFRICA: K[wa]Z[ulu]-Natal/ Garden Castle Nat[ure] Res[erve]/ 29°44′51″S 29°12′36″E/ 25.i.2005 J.G.H. Londt/ 1790m Open grassland/ Resting on large rocks (NMDA). Paratype ♀: same data as holotype (NMDA).

###### Etymology.

Dedicated to our colleague Jason G.H. Londt (KwaZulu-Natal Museum, Pietermatizburg, South Africa), who collected the types.

###### Description.

*Body length*: ca. 8 mm.

Male. *Colouration* (Fig. 17): Fronto-orbital plate, occiput and genal dilation black in ground colour; frontal vitta dark brown; remainder of head pale yellow. Head covered with white to grey reflecting microtomentum. Antenna with scape and pedicel yellow, postpedicel mostly black except yellowish on inner basal portion. Palpus yellow. Thorax black in ground colour, with grey reflecting microtomentum. Presutural area of scutum with 4 dark vittae; median pair narrow, running straight from transverse suture to prothorax; lateral pair short, varying from subtriangular to subrectangular, not reaching posteriorly to transverse suture and ending anteriorly before base of posthumeral seta. Femora mostly dark brown to black but red apically and on distal third ventrally; tibiae yellow; tarsi proximally yellow shading into brown distally. Tegula reddish-brown; basicosta yellow. Wing membrane hyaline. Abdominal syntergite 1+2 and tergite 3 extensively red dorsolaterally and with a black median vitta; tergites 4 and 5 entirely black in ground colour. Tergites 3–5 with a narrow basal band of grey reflecting microtomentum.

*Head* (Fig. 17a, b): Frons about 1.2 times as wide as compound eye in dorsal view. Fronto-orbital plate with a row of 8–9 frontal setae descending to about level of middle of pedicel. One upper lateroclinate orbital seta, one upper medio-reclinate orbital seta. Fronto-orbital plate with 3–4 proclinate orbital setae and a few short setulae lateral to frontal row. Parafacial convex, at its narrowest point 1.2–1.3 times as wide as width of postpedicel. Postpedicel subrectangular with dorsoapical tip pointed, about 1.6 times as long as pedicel. Gena in profile about 0.4 times as high as compound eye. Upper occiput with 1 irregular row of black setulae behind postocular row. Palpus clubbed and covered with stout setulae; prementum short, about 2/3 the length of palpus.

*Thorax*: Four to 6 postpronotal setae, the 3 strongest basal setae arranged in a line. Scutum with 1–2 + 0 acrostichal setae; 3 + 3 dorsocentral setae; 0 + 2–3 intra-alar setae (if 2, then distance between postsutural intra-alar setae shorter than distance between anterior seta and transverse suture). Metathoracic spiracle small and rounded, posterior lappet slightly larger than anterior one.

*Legs*: Fore tibia with 4–7 anterodorsal setae. Fore claws about as long as fifth tarsomere. Mid tibia with 2–4 anterodorsal setae (median 2 distinctly longer than the others). Hind tibia with 6–8 anterodorsal setae, unequal in size.

*Wing*: Base of vein R_4+5_ with 2–3 strong setulae. Bend of vein M_1_ obtuse and rounded. Section of M_1_ between crossveins r-m and dm-m slightly longer than section between dm-m and bend of M_1_. Section of M_1_ between dm-m and bend of M_1_ longer than postangular section of M_1_. Crossveins r-m and dm-m normal.

Female differs from male as follows. Abdomen (Fig. 18e) mostly black in ground colour (dark brown laterally on syntergite 1+2), entirely covered with thick, iridescent, grey microtomentum. Frons about 1.4 times as wide as compound eye in dorsal view. Fronto-orbital plate with 2 proclinate orbital setae. Fifth fore tarsomere enlarged, ovoid; fore claws shorter than fifth tarsomere.

###### Distribution.

South Africa.

##### Key to species of *Austrosolieria* gen. n.

**Table:** 

1	Head ground colour black, covered with grey, iridescent reflecting microtomentum; head when seen in anterodorsal view showing a dark spot on upper parafacial (Fig. 18b, d). Antenna black. Presutural area of scutum with 3 broad dark vittae. Wing membrane with dark infuscations around crossveins r-m and dm-m, and a slightly smoky area along postangular section of M_1_ (Fig. 18h). Scutum with 2 presutural dorsocentral setae. Base of vein R_4+5_ with a tuft of 5–10 setulae. Bend of vein M_1_ with a short appendix (Fig. 18h). Female: Frons about 1.6 times as wide as compound eye in dorsal view	***Austrosolieria freidbergi* sp. n.**
–	Head ground colour not entirely black: anterior part of fronto-orbital plate, parafacial and face yellow; microtomentum of head non-iridescent (Figs 17a–b, 18a, c). Antennal scape and pedicel yellow. Presutural area of scutum with 4 dark vittae. Wing membrane hyaline (Fig. 18g). Scutum with 3 presutural dorsocentral setae. Base of vein R_4+5_ with 2–3 setulae. Bend of vein M_1_ without an appendix (Fig. 18g). Female: Frons about 1.4 times as wide as compound eye in dorsal view	***Austrosolieria londti* sp. n.**

## Supplementary Material

XML Treatment for Mesnilotrix

XML Treatment for Mesnilotrix empiformis

XML Treatment for Filistea

XML Treatment for Filistea aureofasciata

XML Treatment for Filistea verbekei

XML Treatment for Afrophylax

XML Treatment for Afrophylax aureiventris

XML Treatment for Carceliathrix

XML Treatment for Carceliathrix crassipalpis

XML Treatment for Carceliathrix sp. 1

XML Treatment for Carceliathrix sp. 2

XML Treatment for Myxophryxe

XML Treatment for Myxophryxe longirostris

XML Treatment for Myxophryxe murina

XML Treatment for Myxophryxe regalis

XML Treatment for Myxophryxe satanas

XML Treatment for Stiremania

XML Treatment for Stiremania karoo

XML Treatment for Stiremania robusta

XML Treatment for Austrosolieria

XML Treatment for Austrosolieria freidbergi

XML Treatment for Austrosolieria londti
